# Palladium-catalyzed asymmetric allylic 4-pyridinylation via electroreductive substitution reaction

**DOI:** 10.1038/s41467-022-33452-0

**Published:** 2022-09-26

**Authors:** Weijie Ding, Mengfan Li, Jinkun Fan, Xu Cheng

**Affiliations:** 1 Institute of Chemistry and Biomedical Sciences, Jiangsu Key Laboratory of Advanced Organic Materials, School of Chemistry and Chemical Engineering, Nanjing University, Nanjing, 210023 China; 2 State Key Laboratory of Elemento-organic Chemistry, Nankai University, Tianjin, 300071 China

**Keywords:** Asymmetric catalysis, Electrocatalysis, Synthetic chemistry methodology

## Abstract

The enantioselective pyridinylation is important for providing chiral compounds bearing heterocycles of pharmaceutical interests. 4-CN-pyrinde is extensively applied in the radical pyridinylation reaction, however, its’ enantioselective application is highly challenging. To achieve this goal, we propose an electrochemical catalytic activation of 4-CN-pyridine with a chiral transition metal complex instead of direct cathodic reduction. The chiral catalyst acts as the electron mediator and the transition metal catalysis in turn. The radical species from 4-CN-pyridine is captured via radical rebound by chiral catalyst, and undergoes enantioselective pyridinylation reaction. Here, we show the first method for catalytic asymmetric allylic 4-pyridinylation reactions using 4-CN-pyridine under electrochemical conditions.

## Abstract (web summary)

Controlling the enantioselectivity of radical reactions is a persistent challenge in organic synthesis. Here, the authors report a method to form asymmetric pyridine derivatives via the combination of chiral palladium catalysis and electrochemistry.

## Introduction

Controlling the enantioselectivity during catalytic electron-transfer reactions is a challenging task^1–8^. Electron transfer can lead to open-shell species that have different bonding characteristics than species generated by close-shell reactions involving chiral catalysts. Rationale enantioselective reactions that use the tremendous driving force of an electric potential are in high demand.

**Fig. 1:**
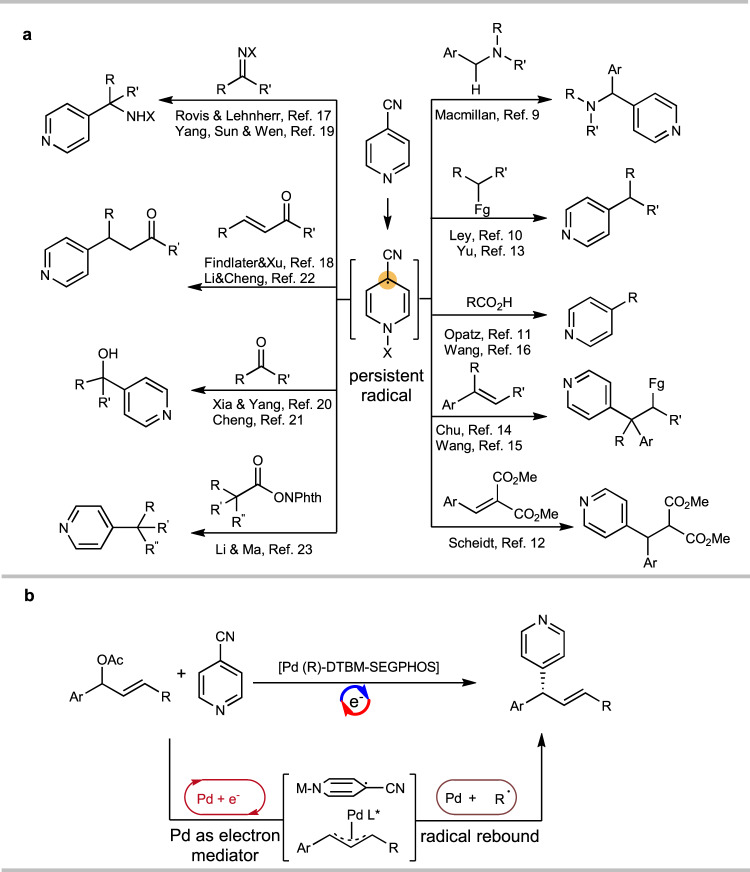
Use of 4-CN-pyridine in pyridinylation reactions to construct molecules with chiral centers. **a** Established chemistry with 4-CN-pyridine for racemic/achiral compounds. **b** This work: enantioselective electroreductive palladium-catalyzed pyridinylation via allylic substitution.

4-CN-pyridine is a typical reagent in open-shell chemistry. It is able to accept electrons under various conditions, and it has been shown to be a versatile pyridinylation reagent in photoredox catalysis^9–16^, electrochemistry^17–21^, and thermal chemistry^22,23^ to build molecules with C_sp3_ centers (Fig. 1a). In these reactions, it forms a persistent radical that undergoes C–C bond formation reactions with the substrates^22^. This chemistry can be used to build pyridine-bearing stereogenic centers, which are present in many pharmaceutical compounds^24–26^. Despite these highly important achievements, to date, the method to control the key open shell radical is a challenging task.^27^ In addition, our previous findings^22^ suggested the persistent radical derived from 4-CN-pyridine localizes the major spin density at its tertiary carbon center, which does not fall into the paradigm established for controlling the stereochemistry of radical reaction^28–39^. At this point, it would be highly desirable to develop an enantioselective pyridinylation reaction with 4-CN-pyridine, which can not only achieve an enantioselective model regulating persistent tertiary radical center, but also provide a class of chiral pyridine compounds. The protocol would provide the complementary to successfully enantioselective 4-pyrdinylation methods via 2-electron or single electron pathways.^40^

Asymmetric allylic alkylation is a powerful strategy for converting readily available allylic compounds to a diverse array of chiral products^41–55^. Notably, however, asymmetric allylic pyridinylation has not been achieved, partly because of the difficulty in generating and regulating the nucleophilic planar close-shell pyridinyl species. In this regard, we conceive a pathway involving the persistent radical would be a solution, in which an enantioselective catalysis design for both allyl partner and tertiary radical is the key.

Here, we show the first example for asymmetric allyl pyridinylation with 4-CN-pyridine driven by electrochemistry, in which the chiral Pd complex functions as both an electron-transfer mediator and chiral catalyst in the relay model to introduce persistent radical into the stereogenic center via a Pd-effected radical rebound,^56–59^ which has not been explored (Fig. 1b).^60–64^

## Results

### Reaction optimization

To begin our study, we chose cinnamyl acetate **1a** as a substrate for reactions with 4-CN-pyridine (**2a**) under constant-current electrolysis conditions in an undivided cell with a graphite felt (GF) cathode and a Zn anode (Table 1). When PdCl_2_ was the catalyst precursor and (*R*)-SEGPHOS (**L1**) was the chiral ligand, reaction in 5:1 (v/v) MeCN/MeOH at 35 °C and a current density of 5 mA/cm^3^ afforded a 8:1 mixture of desired branched (B) product **3a** and linear (L) product **3a’** (entry 1); the enantiomeric excess (ee) for **3a** was 70%. Changing to a single solvent (MeCN or DMF) decreased the yield, B/L ratio, and enantioselectivity (entries 2 and 3). If Et_4_NBF_4_ was used as supporting electrolyte, the reaction gave **3a** in 60% yield, with a B/L of 5:1 and 65% ee (entry 4). When (R)-DTBM-SEGPHOS (**L2**) was used as the ligand, the B/L ratio and the ee value increased to 13:1 and 93%, respectively; and the isolated yield of **3a** was 76% (entry 5). X-ray analysis of a hydroxylative derivative of **3a** indicated that it had a R configuration (Supplementary Fig. 162). Subsequently, chiral ligand (R)-BINAP (**L3**) and (R)-SPD (**L4**) were evaluated, and these experiments showed that a ligand with a small dihedral angle gave better results (entries 1, 6, and 7). (R)-DTBM-GARPHOS (**L5**, entry 8), which has a small dihedral angle, gave an ee similar to that obtained with **L2**. In contrast, trialkyl phosphine ligand **L6** gave a moderate yield and relatively low enantioselectivity (entry 9). Reactions involving (S, R)-Josiphos (**L7**) and monodentate ligand **L8** (entries 10 and 11, respectively) did not give measurable amounts of the desired product. When a stoichiometric metal reductant (Zn or Mg) was used instead of electricity, no reaction took place (entry 12). Finally, when Pd and chiral ligand were absent, no conversion of both **1a** and **2a** were observed (entry 13).

**Table 1:** Optimization of conditions for catalytic asymmetric electrochemical allylic 4-pyridinylation^a^


Entry	Deviation from initial conditions	Ligand	Yield	B/L	ee of 3a
1	None	L1	74	8:1	70%
2	MeCN only	L1	trace		
3	DMF only	L1	54	2:1	11%
4	Et_4_NBF_4_ instead of Et_4_NCl	L1	60	5:1	65%
5	None	L2	78 (76)^b^	13:1	93% (R)
6	None	L3	73	6:1	50%
7	None	L4	28	6:1	22%
8	None	L5	66	10:1	90%
9	None	L6	40	4:1	−77%
10	None	L7	trace		
11	None	L8	n. d.		
12	Zn or Mg instead of electricity	L1	n. r.		
13	No Pd catalyst	/	n. r.		

*GF* graphite felt, *ee* enantioselectivity, *NMR* nuclear magnetic resonance, *HPLC* high performance liquid chromatography, *DMF* N,N-dimethyl formamide, *B/L* branch/linear, *n. d.* not detected, *n. r*. no reaction.

^a^Standard conditions: **1a** (0.2 mmol), **2a** (0.6 mmol), PdCl_2_ (10 mol%), **L** (12 mol%), 5:1 (v/v) MeCN/MeOH, Et_4_NCl (0.1 M), Zn(+)|GF(-), 5 mA/cm^3^, 5 h, 35 °C; ^1^H NMR yields are reported; Ee values were determined by HPLC on a chiral stationary phase.

^b^Isolated yield.

### Substrate scope

**Fig. 2:**
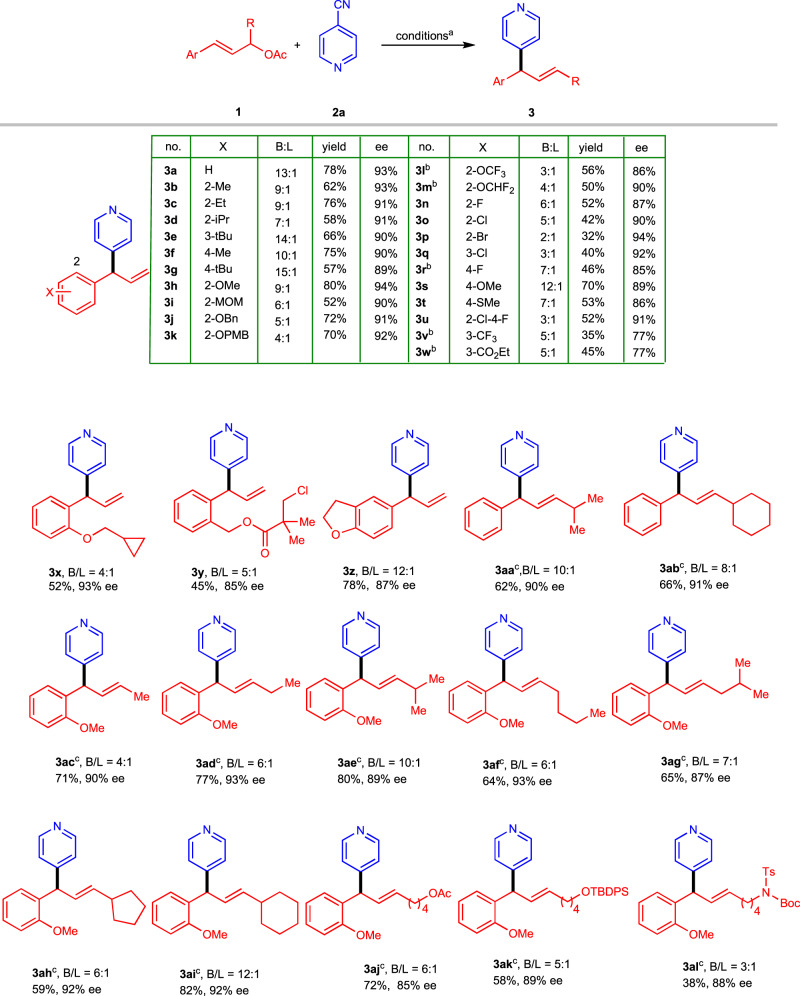
Electrochemical asymmetric allylic 4-pyridinylation of cinnamyl acetates 1. ^**a**^Conditions: **1** (0.2 mmol), **2a** (0.6 mmol), PdCl_2_ (10 mol%), **L2** (12 mol%), MeCN/MeOH 5:1 (v/v), Et_4_NCl (0.1 M), Zn(+)|GF(-), 5 mA/cm^3^, 5 h, 35 °C; isolated yields of branched product are reported; B/L and E/Z ratios were determined by ^1^H NMR analysis of crude reaction mixtures; ee values were determined by HPLC on a chiral stationary phase. ^**b**^Current density, 8 mA/cm^3^. ^**c**^only E configuration was detected. MOM, methoxylmethyl; PMB, 4-methoxybenzyl; TBDPS, t-butyldiphenylsilyl; Ts, tosyl; Boc, t-butyloxycarbonyl.

Using the optimized conditions (Table 1, entry 5), we explored the substrate scope of this asymmetric allylic pyridinylation reaction (Fig. 2). We began by systematically assessing the effect of the substituent (X) on the phenyl ring of cinnamyl acetate **1**. The steric bulk of the substituent did not markedly affect the regio- or stereoselectivity, as indicated by the results obtained for branched products **3b**–**3** **g** with good to excellent ees. Products **3h**–**3** **m**, all of which have an electron-donating alkoxide group at the ortho position, were obtained in moderate to good yields, with ee values typically around 90%. Next, the standard conditions were used to test a series of halogenated substrates, which gave products **3n**–**3r** with good to excellent enantioselectivities (85%–94%). We were pleased to find that even an aryl bromide moiety survived the reaction: product **3p** was obtained with 94% ee. Products **3** **s** and **3t**, which have para electron-donating groups, were obtained with 89% and 86% ee values, respectively; and dihalogenated product **3** **u** was obtained with 91% ee. Products **3** **v** and **3w**, which have electron-withdrawing substituents, had low yield due to low regioselectivity, and the enantioselectivies were moderate. Compounds with cyclopropyl, carboxylate, and dihydro-benzofuran moieties (**3x**–**3z**, respectively) were generated in acceptable yields with good to excellent ee values. A substrate with a secondary allyl acetate group afforded branched product **3aa** in 62% isolated yield with an ee of 90%; the internal alkene with the E configuration was the only detectable product. Moreover, products **3ab**–**3al** were generated smoothly with good to excellent enantioselectivities.

**Fig. 3:**
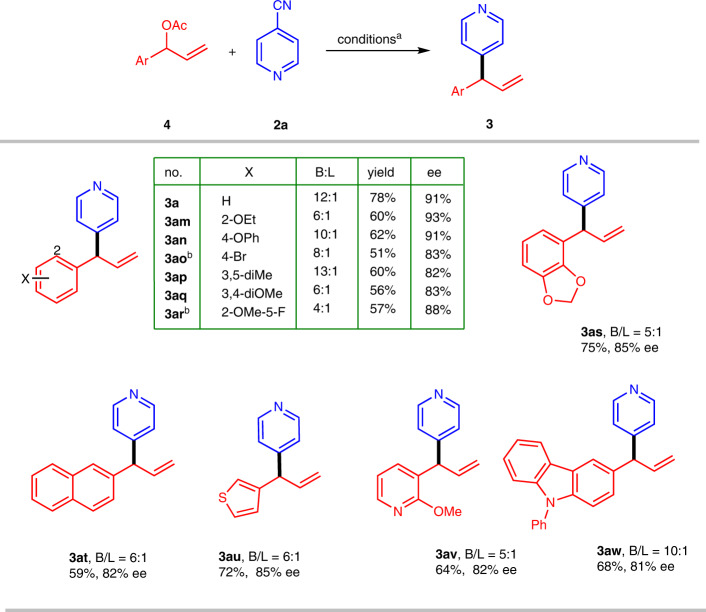
Electrochemical asymmetric allylic 4-pyridinylation of 4. ^**a**^Conditions: **4** (0.2 mmol), **2a** (0.6 mmol), PdCl_2_ (10 mol%), **L2** (12 mol%), MeCN/MeOH 5:1 (v/v), Et_4_NCl (0.1 M), Zn(+)|GF(-), 5 mA/cm^3^, 5 h, 35 °C; isolated yields of branched products are reported; B/L were determined by with ^1^H NMR analyses of crude reaction mixture; Ee values were determined by HPLC on a chiral stationary phase. ^**b**^Current density, 8 mA/cm^3^.

To further explore the scope, we prepared acetate **4a** from benzaldehyde and vinyl Grignard reagent and allowed it to react with **2a** under the standard conditions (Fig. 3). The outcome of this reaction was almost identical to that of the reaction of **1a** and **2a** (Fig. 2). We also tested several other 3-acetoxy-3-aryl propenes. Specifically, product **3am** and **3an**, which has an alkoxyl or phenoxyl group on the phenyl ring, was obtained with 93% and 91% ee. Other branched substrates also gave corresponding product **3ao**–**3at** in good enantioselectivities. Compound **3au**–**3aw** bearing two heterocycles were prepared with the same protocol in 81%–85% ee.

**Fig. 4:**
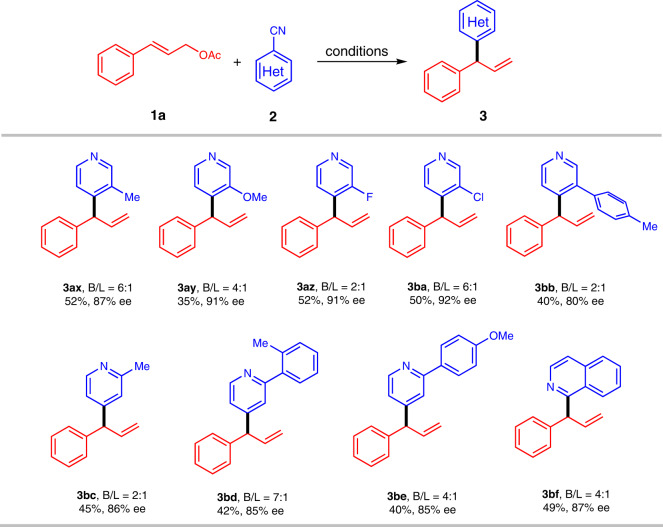
Electrochemical asymmetric allylic N-heteroarylation. Conditions: **1a** (0.2 mmol), **2** (0.6 mmol), PdCl_2_ (10 mol%), **L2** (12 mol%), 5:1 (v/v) MeCN/MeOH, Et_4_NCl (0.1 M), Zn(+)|GF(-), 8 mA/cm^3^, 3 h, 35 °C; isolated yields are reported; ee values were determined by HPLC on a chiral stationary phase.

Subsequently, we explored reactions of **1a** with different N-heterocycles **2** (Fig. 4). A broad scope of both electronic and sterically differentiated substituted on pyridine ring were accommodated well. However, the steric hindrance of 3-substituted substrate on pyridine ring has a certain effect on the enantioselectivity of the reaction. The greater the steric hindrance, the lower the corresponding enantioselectivity (**3ax**-**3bb**). The steric effect of pyridine 2-position only slightly influenced the enantioselectivity: products **3bc**–**3be** were obtained with 85–86% ee. Finally, reaction between 1-CN-isoquinoline and **1a** gave corresponding product **3bf** in 49% isolated yield with an ee of 87%.

**Fig. 5:**
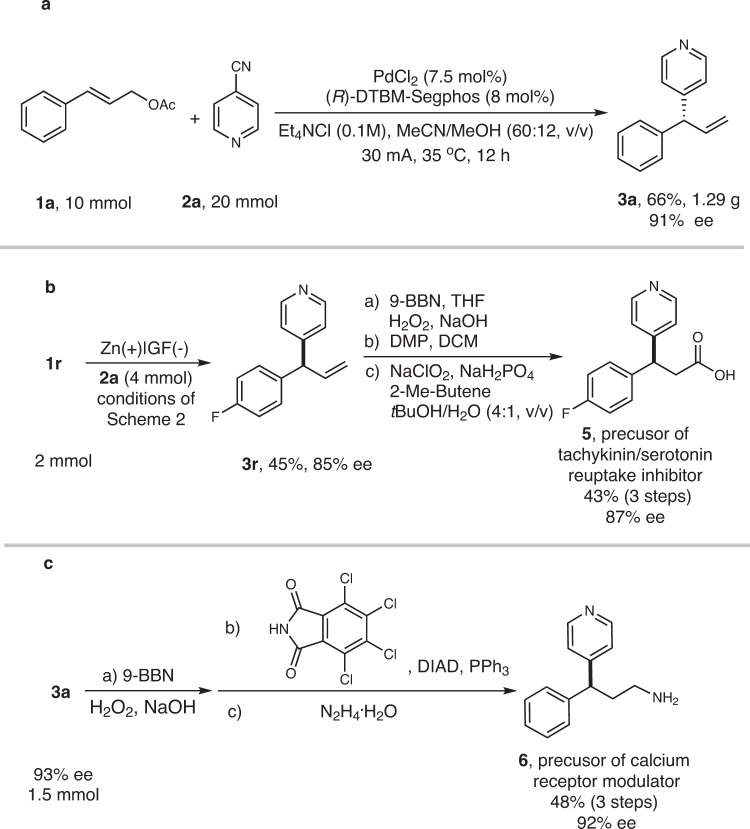
Use of enantioenriched allyl pyridines as pharmaceutical precursors. **a** Reaction at 10 mmol scale. **b** Synthesis of chiral precursor of tachykinin/serotonin reuptake inhibitor. **c** Synthesis of chiral precursor of calcium receptor modulator. 9-BBN, 9-borabicyclo[3.3.1]nonane; DMP: Dess-Martin periodinane; DIAD, diisopropyl azodicarboxylate.

Next, the reaction of **1a** and **2a** was conducted at 10 mmol scale under standard conditions. It was glad to find 1.29 g of **3a** was isolated in 66% yield with 91% ee (Fig. 5a). We demonstrated the synthetic utility of this method by carrying out a reaction between 2 mmol of **1r** and **2a** under the standard conditions; this reaction afforded desired product **3r** in 45% yield with 85% ee. Further elaboration of **3r** gave **5** (43% yield over three steps) with 87% ee. This product is an intermediate in a known procedure for the synthesis of tachykinin/serotonin-reuptake inhibitors (Fig. 5b)^65^. In addition, reaction of 2 mmol of **1a** gave product **3a** (75% yield, 93% ee), which was converted to amine **6**, an intermediate in the synthesis of a calcium receptor modulator^66^, with almost complete enantioretention (Fig. 5c).

### Study of mechanism

**Fig. 6:**
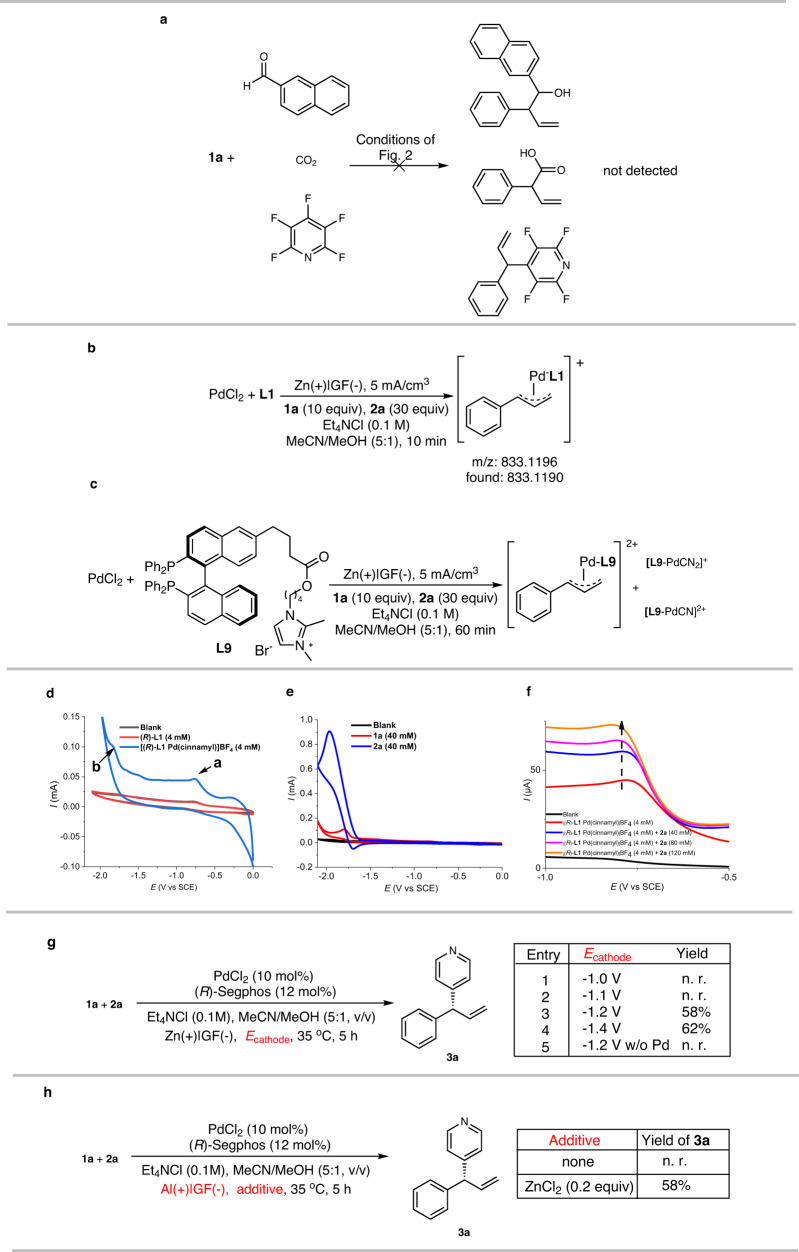
Control experiments to investigate reaction pathway. **a** Control experiment with reactive electrophiles. **b** High resolution mass spectrometry (HRMS) analysis of reaction mixture under standard conditions. **c** HRMS analysis of reaction mixture using positive charge-tagged BINAP as chiral ligand. **d** Cyclic voltammetry (CV) analysis of Pd-cinnamyl complex. **e** CV analysis of reactants **1a** and **2a**. **f** Catalytic current of Pd complex. **g** Experiment under controlled cathodic potential. **h** The role of Zn in the reaction. SCE, saturated calomel electrode.

Next, we conducted some control experiments to gain information about the reaction mechanism. At first, we used 2-naphthaldehyde, carbon dioxide, and perfluoropyridine as electrophiles instead of 4-CN-pyridine under otherwise identical conditions. These reactions did not afford the corresponding adducts, implying that allylic nucleophilic addition was not the predominant pathway (Fig. 6a). Then we measured the high-resolution mass spectrum of a reaction mixture containing **L1** under the standard conditions after 10 min of reaction time, which revealed that the major Pd species was an **L1**-Pd(cinnamyl) complex (Fig. 6b, more details in Supplementary Fig. 5). Furtherly, a positive charge tagged BINAP ligand was prepared and subjected to the standard reaction conditions. In addition to the allyl complex, the two Pd-CN rather than Pd-Cl complexes were identified by HRMS, suggesting the stronger coordinative ability of cyanide group (Fig. 6c, more details in Supplementary Fig. 6).

Therefore, we prepared an **L1**-Pd(cinnamyl)–BF_4_ complex and subjected it to cyclic voltammetry. Two significant reduction peaks were present in the voltammogram: one at −0.8 V and the other at −1.7 V vs SCE (labelled with a and b in Fig. 6d); these CV data are similar to data reported by Lei in their study on Pd(MeCN)_2_Cl_2_/P(p-Tol)_3_ in MeCN/*n*BuOH^67^. In addition, chiral phosphine ligand **L1** along did not undergo cathodic reduction even at −2.2 V vs SCE. This resulted suggested an open-shell Pd species might exist after the first cathodic electron transfer at −0.8 V. In comparison, cathodic electron transfer to cinnamyl acetate (**1a**) and 4-CN-pyridine (**2a**) took place at a cathode potential around −1.8 V vs SCE, which is more difficult than **L1**-Pd(cinnamyl)–BF_4_ (Fig. 6e). Subsequently, we studied the CV of the **L1**-Pd(cinnamyl)–BF_4_ complex at −0.8 V vs SCE using **2a** as an additive. A catalytic current around 10 μA scale (Fig. 6f) was detected, similar to the catalytic current observed in the reported Cu-catalyzed^68,69^ and Rh-catalyzed^70^ electrochemical asymmetric syntheses. This observation revealed that the reduced Pd species instantly transfers an electron to **2a** to generate intermediate **E**, which again accepts an electron from the cathode. In this manner, the Pd complex not only serves as a transition-metal catalyst but also mediates an electron transfer. To further verify the mediation, the reaction was conducted with controlled cathodic potential (Fig. 6g). It was found with −1.2 V vs SCE cathodic potential was adequate to effect the conversation to desired product along with pyridine as side product. On the other hand, if PdCl_2_ and phosphine ligand were removed, no reaction took place at −1.2 V vs SCE, and the substrate **1a** and pyridine **2a** were recovered almost quantitatively. In addition, the role of Zn in the reaction was examined. When the sacrificial anode was changed from Zn to Al, the reaction did not take place (Fig. 6h). In comparison, if 0.2 equiv of ZnCl_2_ was used as additive in the reaction with Al as anode, the reaction worked smoothly to give product **3a** in 58% yield.

**Fig. 7:**
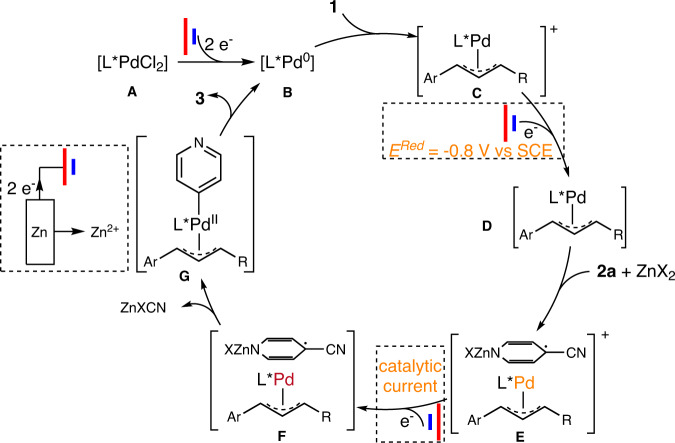
Plausible reaction pathway for electrochemical asymmetric allyl 4-pyridinylation. Precursor **A** is reduced to complex **B** and in turn to allyl intermediate **C**. **C** undergoes rebound of **2a** under cathodic reductions twice via **D** and **E** to give species **F** and in turn **G**. The reductive elimination of **G** furnishes the product and regenerates catalyst **B**.

On the basis of the above-described results, a plausible pathway is outlined in Fig. 7. First, PdCl_2_–diphosphine ligand complex **A** is reduced to [L*Pd^0^] complex **B**. Oxidative addition of cinnamyl acetate **1** to **B** gives rise to η^3^ allyl Pd^II^ complex **C**. This complex undergoes cathodic reduction at −0.8 V (vs SCE) to form Pd species **D**, which readily donates an electron to 4-CN-pyridine (**2a**), generating Pd species **E**; this single-electron transfer occurs readily because of the ability of **2a** to stabilize anionic radicals with zinc counter ion. Complex **E** quickly accepts another electron from the cathode to produce Pd species **F**, generating a catalytic current. Subsequently, η^3^ allyl pyridinyl Pd^II^ complex **G** forms from **F** via radical rebound and then undergoes reductive elimination to afford desired product **3** and regenerate Pd^0^ complex **B**. The Zn^2+^ from the sacrificial anode both activates **2a** and quenches the CN^−^ anion.

**Fig. 8:**
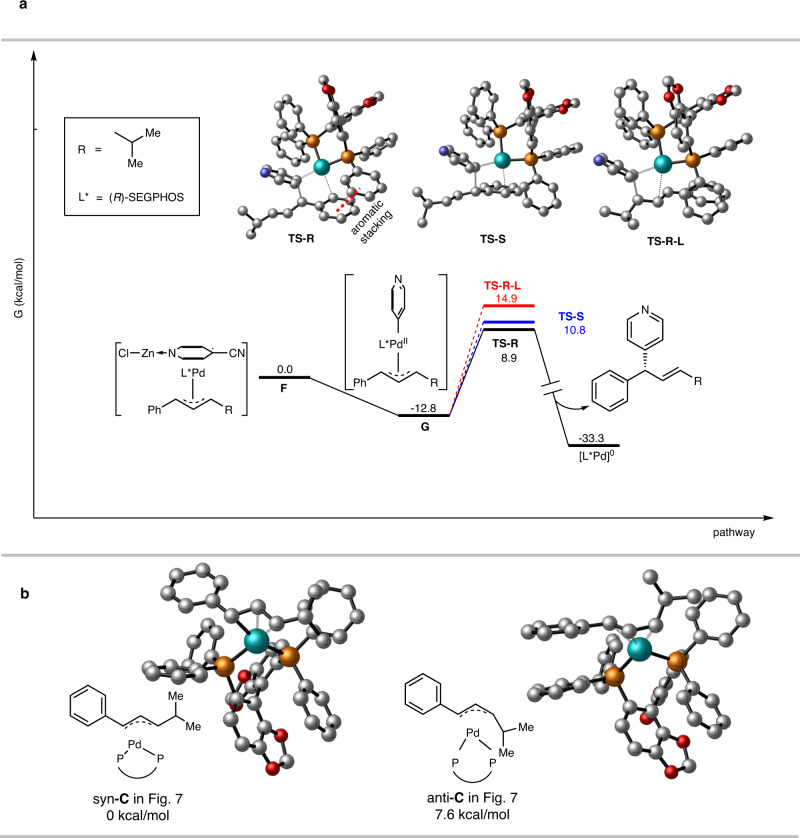
Computational profile of reaction species and selectivities. **a** Reaction profile of reaction pathway to determine the regio- and enantioselectivity. **b** The difference of energy between syn- and anti- isomers of intermediate **C**.

By using the reaction of **3aa** as the model, the regio-, and stereoselectivities were studied with density functional theory (DFT) computation study at B3LYP-3D level of theory (Fig. 8a). The free energy profile starting from intermediate **F** shown in Fig. 7. The atomic spin population analysis shows the single electron delocalizes on Pd and allyl group of intermediate **F**. (Supplementary Fig. 24). At first, the complex **F** undergoes the extrusion of Zn salt gives rise to complex **G** as exothermal process of −12.8 kcal/mol, since the formation of aromatic pyridinyl group. At this stage, the reductive elimination offers the product **3aa** and establishes regio- and enantioselectivity. To compare the regioselectivity, two transition state **TS-R** and **TS-R-L** corresponding to branch and linear products were located. In **TS-R**, the phenyl ring connected to allyl group participates in the complexation with Pd, leading to an aromatic stacking between this phenyl ring and one phenyl ring of chiral ligand. In comparison, in **TS-R-L** there is allyl group coordinating to Pd metal, spacing the phenyl groups and diminishing the aromatic interaction. This difference between **TS-R** and **TS-R-L** may result in the favor on branched product, though the 6.0 kcal/mol gap exceeds the experimental ratio (B/L = 8:1). Subsequently, the comparison of **TS-R** and **TS-S** corresponding to R and S products showed the aromatic stacking stabilization is absent in **TS-S**. As a result, **TS-R** is 1.9 kcal/mol lower than **TS-S** in terms of energy. Within the precision range of DFT calculation, this result is confirmed by the experimental data (86% ee). In this reaction, only alkene with E configuration was detected and isolated substrate **1ac** with either E or Z configuration (Supplementary Fig. 19). This observation was investigated with DFT calculation of intermediate **C** in Fig. 7, showing the syn-allyl-Pd-diphosphine complex is predominant over the interconvertible *anti*-counterpart with a stabilization of 7.6 kcal/mol due to steric effect (Fig. 8b). A ^31^P NMR tracking of reaction mixture also confirmed this predominance (Supplementary Fig. 21). In turn, the syn-intermediate can lead to product with E configuration.

In summary, we developed the first method for asymmetric allylic 4-pyridinylation reactions under electrochemical conditions. These reactions are catalyzed by a chiral diphosphine Pd complex and use 4-CN-pyridine as a pyridine source. The Pd complex not only acts as a chiral catalyst but also mediates electron transfer between the cathode and 4-CN-pyridine. More than 50 examples are reported, giving products with ee values as high as 94%; and the method was used to synthesize chiral pharmaceutical intermediates. Under these catalytic conditions, the 4-CN-pyridine is activated in solution below its reduction potential instead of at interface of electrode directly, avoiding the generation of uncontrollable species.

## Methods

### General procedure for the synthesis of 3

A 10 mL three-necked flask was charged with PdCl_2_ (0.02 mmol, 0.1 equiv), (R)-DTBM-SEGPHOS (0.024 mmol, 0.12 equiv) and a magnetic stir bar. The flask was evacuated and backfilled with argon for three times, and anhydrous MeCN (2 mL) was added via syringe. The mixture was stirred under room temperature for 30 min. Then the substrate **1** (0.2 mmol, 1.0 equiv), **2** (0.6 mmol, 3.0 equiv), Et_4_NCl (0.6 mmol, 3.0 equiv), anhydrous MeCN (3 mL) and MeOH (1 mL) was added. The flask was equipped with a rubber stopper, graphite felt (2 cm × 1 cm x 0.5 cm) as cathode, Zn (1.5 cm × 1 cm x 0.2 cm) as anode. The Zn anode attached to a platinum wire and graphite felt cathode attached to a titanium wire. The mixture was stirred under 35 °C and constant current electrolysis (5 mA). After the reaction completed (TLC or GC-MS analysis), the mixture was extracted with ethyl acetate. The organic layers were washed with brine, dried over Na_2_SO_4_, filtered and concentrated. The residue was purified by chromatography on silica gel to afford the desire product.

## Supplementary information

Supplementary Information

Description of Additional Supplementary Files

Supplementary Data 1

## Data Availability

The HPLC, NMR, HRMS and DFT compuation data generated in this study are provided in the Supplementary Information. Cartesian coordinates of the calculated structures are available from the Supplementary Data 1. Crystal structure data generated in this study is also available in the CCDC database under accession code 2118567.
