# Supplementary material for: Palladium-catalyzed asymmetric allylic 4-pyridinylation via electroreductive substitution reaction
Source: Nat Commun. 2022 Sep 26;13:5642. doi: 10.1038/s41467-022-33452-0 (PMC9512896; doi:10.1038/s41467-022-33452-0)
Supplement: Supplementary file 1 — Supplementary Information [file 41467_2022_33452_MOESM1_ESM.pdf]

Supplementary Information

Palladium-catalyzed Asymmetric Allylic 4-Pyridinylation via Electroreductive Substitution Reaction

Ding et al

1. Supplementary Methods....S1
1.1 General Information....S1
1.2 General procedures for the synthesis of the substrates....S1
1.3 General procedure for the synthesis of products 3....S4
1.4 Derivation of the product 3....S14
2. Supplementary Discussion....S18
2.1 Preparation of π-allylpalladium complex....S18
2.2 Synthesis of the charge-tagged diphosphate ligand L9....S19
2.3 HRMS analysis of reaction mixture....S25
2.4 Electrochemical analysis experiment....S26
2.5 NMR titration....S29
2.6 Control experiments....S31
2.7 Monitoring the reaction of (E)- or (Z)-1ac to 3ac....S32
2.8 Monitoring reaction of (E)- or (Z)-1ac forming allyl Pd-L1 complex....S34
2.9 The change of enantioselectivity in the reaction....S36
2.10 DFT computation study of reaction pathway....S37
2.11 Explore another persistent radical precursor....S40
3. Supplementary Notes....S42
3.1 Product characterization....S42
3.2 NMR spectra for new compounds....S124
3.3 X-ray single crystal data for the derivative compounds 3a....S229
4. Supplementary References....S239

1. Supplementary Methods

1.1 General Information

Unless otherwise noted, all reactions were carried out under argon atmosphere. All materials were obtained from commercial suppliers and used directly without further purification. Chiral ligands were purchased from Sinocompound or Bidepharm. Other chemical reagents were purchased from commercial sources and used without further purification. Flash chromatography utilized 300-400 mesh silica gel from Qingdao Haiyang Chemical Co., Ltd. Reactions were monitored by thin-layer chromatography (TLC) using 254 nm UV light to visualize the progress of the reactions. ^{1}H NMR and ^{13}C NMR spectra were recorded on Bruker Avance III 400 (400 MHz and 100 MHz). All ^{1}H NMR and ^{13}C NMR spectra are reported in parts per million (ppm) downfield of TMS. Spectra were reported as follows: chemical shift (δ ppm), multiplicity (s = singlet, d = doublet, t = triplet, q =quartet, m = multiplet), coupling constants (Hz) and integration. High-resolution mass spectra (HRMS) were obtained by ESI or EI source and a TOF detector mass spectrometer. High-pressure liquid chromatography (HPLC) was performed on Shimadzu LC-10ATvp or LC-20AT chromatographs using chiral columns as noted for each compound. Optical rotations were measured on an automatic polarimeter with $^{[\alpha]}D^{25}$ values reported in degrees; concentration (c) is in g/100 mL.

1.2 General procedures for the synthesis of the substrates

General procedure A:

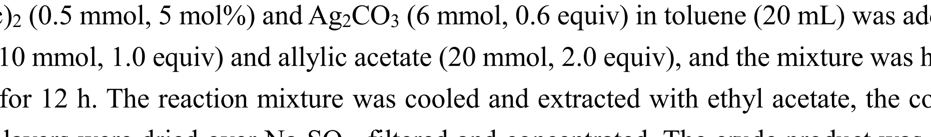

The allylic acetates were prepared following a literature-known procedure:[1] To a mixture of $Pd(OAc)_2$ (0.5 mmol, 5 mol%) and Ag_2CO_3 (6 mmol, 0.6 equiv) in toluene (20 mL) was added aryl iodide (10 mmol, 1.0 equiv) and allylic acetate (20 mmol, 2.0 equiv), and the mixture was heated at 100 ºC for 12 h. The reaction mixture was cooled and extracted with ethyl acetate, the combined organic layers were dried over Na_2SO_4, filtered and concentrated. The crude product was purified by column chromatography on a silica gel (petroleum ether/ethyl acetate) to afford the desired

product.

General procedure B:

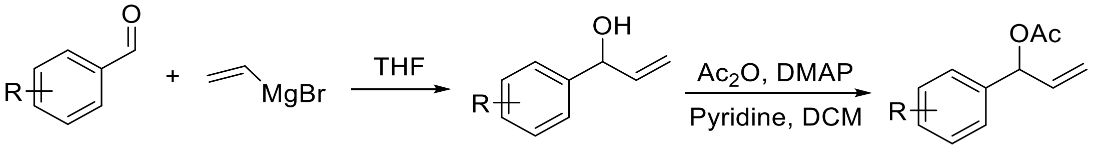

To a solution of aromatic aldehyde (10 mmol, 1.0 equiv) in anhydrous THF was added vinylmagnesium bromide (12 mmol, 1.2 equiv) at 0 °C under nitrogen. The reaction mixture was allowed to warm to room temperature, and then stirred for about 2 h (monitored by TLC). Until the aldehyde was completely consumed, the reaction was quenched by the addition of a saturated solution of NH_4Cl and diluted with ethyl acetate. The combined organic layers were washed with brine, dried over Na_2SO_4, filtered and concentrated. The residue was used in next step without further purification.

DMAP (1 mmol, 0.1 equiv), Ac_2O (12 mmol, 1.2 equiv), pyridine (15 mmol, 1.5 equiv) and DCM (20 ml) were added in the flask containing above residue, and then the solution was stirred for another 2 h at room temperature. Until the alcohol was completely consumed, the mixture was diluted with ethyl acetate. The organic layers were washed with brine, dried over Na_2SO_4, filtered and concentrated. The residue was purified by chromatography on silica gel to afford the desire product.

General procedure C:

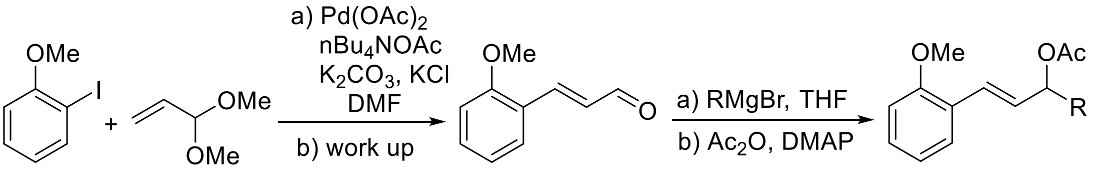

The substrate was prepared following a known procedure: [2] To a mixture of $Pd(OAc)_2$ (1 mmol, 5 mol%), *n*Bu_4NOAc (40 mmol, 2.0 equiv), K_2CO_3 (30mmol, 1.5 equiv), KCl (20 mmol, 1.0 equiv), and 2-Iodoanisole (20 mmol, 1.0 equiv) in DMF was added acrolein dimethyl acetal (60 mmol, 3.0 equiv). The mixture was allowed to heated to 90 °C, and stirred for 18 h. Until the starting material was completely consumed, the reaction was cooling to 0 °C, and quenched by the addition of a 2N HCl, the mixture was extracted with ethyl acetate/petroleum ether (1:1). The combined organic layers were washed with brine, dried over Na_2SO_4, filtered and concentrated. The residue was purified by chromatography on silica gel to afford the desire product as a yellow solid (1.56 g, 48%).

Then to a solution of above cinnamaldehyde (5 mmol, 1.0 equiv) in anhydrous THF was added the Grignard reagent (6 mmol, 1.2 equiv) at 0 °C under nitrogen. The reaction mixture was allowed to warm to room temperature, and stirred for about 2 h (monitored by TLC). Until the aldehyde was completely consumed, the reaction was quenched by the addition of a saturated solution of NH_4Cl and diluted with ethyl acetate. The combined organic layers were washed with brine, dried over Na_2SO_4, filtered and concentrated. The residue was purified by chromatography on silica gel to

afford the desire product.

General procedure D:

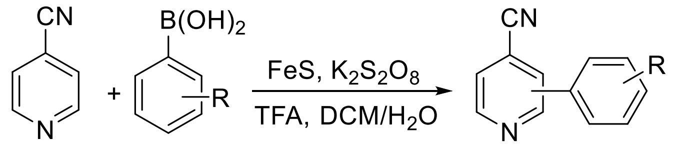

The substrate was prepared following a known procedure: [3] To a round-bottom flask equipped with magnetic stir bar was charged with 4-CN-pyridine (5 mmol, 1.0 equiv), TFA (5 mmol, 1.0 equiv), FeS (5 mmol, 1.0 equiv), arylboronic acids (7.5 mmol, 1.5 equiv), and $K_2S_2O_8$ (15 mmol, 3.0 equiv) in DCM / H_2O (20 mL: 20 mL). The mixture was allowed to stirring for 40 h at room temperature. The resulting solution was directly filtered through a pad of celite and washed with DCM (15 mL). The filtrate was washed with a saturated solution of $NaHCO_3$ (3 x 15 mL) and the aqueous layer was extracted again with DCM (3 x 15 mL). The organic layers were washed with brine, dried over Na_2SO_4, filtered and concentrated. The residue was purified by chromatography on silica gel to afford the desire product.

1.3 General procedure for the synthesis of products 3

1.3.1 Synthesis of racemic products 3

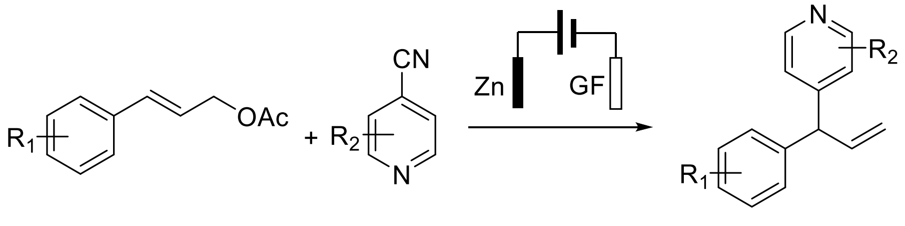

1 2 3

A 10 mL three-necked flask was charged with the substrate **1** (0.2 mmol, 1.0 equiv), **2** (0.6 mmol, 3.0 equiv), Pd(dppp)Cl_2 (0.02 mmol, 10 mol%), Et_4NCl (0.6 mmol, 3.0 equiv) and a magnetic stir bar. The flask was equipped with a rubber stopper, graphite felt (2 cm x 1 cm x 0.5 cm) as cathode, Zn (1.5 cm x 1 cm x 0.2 cm) as anode. The Zn anode attached to a platinum wire and graphite felt cathode attached to a titanium wire. The flask was evacuated and backfilled with argon for three times, and anhydrous MeCN (5 mL) and MeOH (1 mL) were added via syringe. The mixture was stirred under room temperature and constant current electrolysis (10 mA). After the reaction completed (TLC or GC-MS analysis, about 3 h), the mixture was extracted with ethyl acetate. The organic layers were washed with brine, dried over Na_2SO_4, filtered and concentrated. The residue was purified by chromatography on silica gel to afford the desire product.

1.3.2 Synthesis of chiral products 3

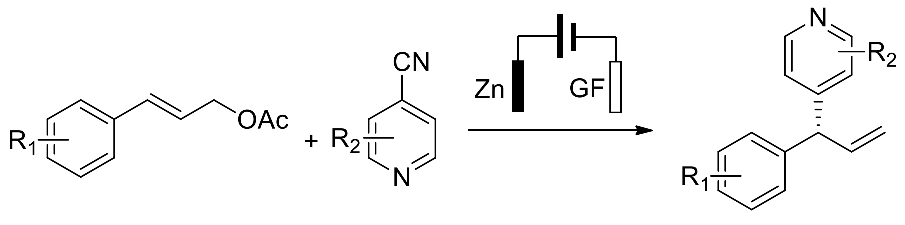

1 2 3

A 10 mL three-necked flask was charged with $PdCl_2$ (0.02 mmol, 0.1 equiv), (*R*)-DTBM-Segphos (0.024 mmol, 0.12 equiv) and a magnetic stir bar. The flask was evacuated and backfilled with argon for three times, and anhydrous MeCN (2 mL) was added via syringe. The mixture was stirred under room temperature for 30 min. Then the substrate **1** (0.2 mmol, 1.0 equiv), **2** (0.6 mmol, 3.0 equiv), Et_4NCl (0.6 mmol, 3.0 equiv), anhydrous MeCN (3 mL) and MeOH (1 mL) was added. The flask was equipped with a rubber stopper, graphite felt (2 cm x 1 cm x 0.5 cm) as cathode, Zn (1.5 cm x 1 cm x 0.2 cm) as anode. The Zn anode attached to a platinum wire and graphite felt cathode attached to a titanium wire. The mixture was stirred under 35 °C and constant current electrolysis (5 mA). After the reaction completed (TLC or GC-MS analysis), the mixture was extracted with ethyl acetate.

The organic layers were washed with brine, dried over Na_2SO_4, filtered and concentrated. The residue was purified by chromatography on silica gel to afford the desire product.

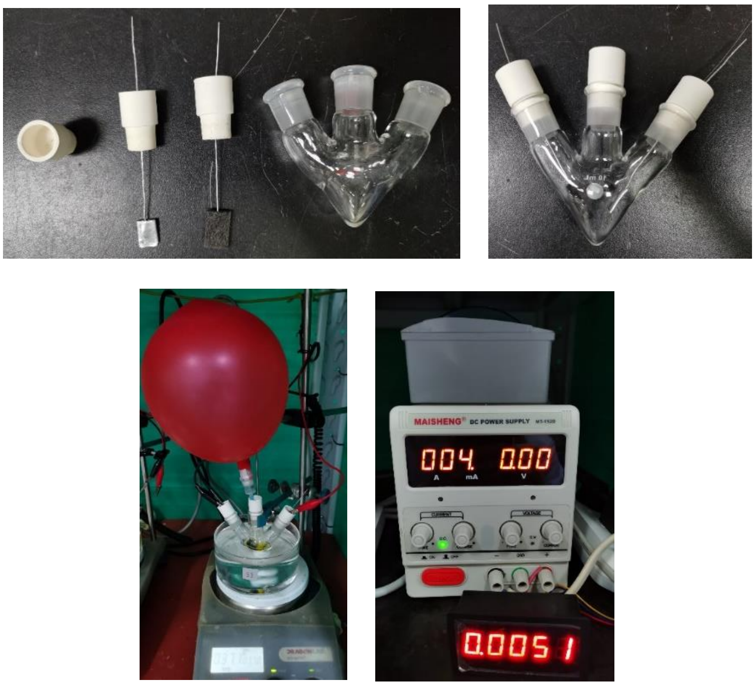

Supplementary Figure 1. **Reaction apparatus for > mmol scale reactions.**

1.3.3 Gram-scale preparation of 3a

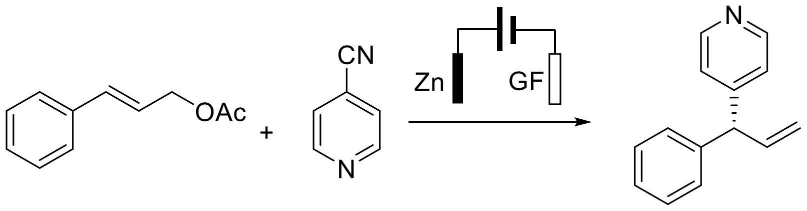

1a **2a** **3a**

2 mmol 4 mmol

A 50 mL two-necked flask was charged with $PdCl_2$ (7.5 mmol, 10 mol %), (*R*) - DTBM-Segphos (8 mmol, 12 mol %) and a magnetic stir bar. The flask was evacuated and backfilled with argon for three times, and anhydrous CH_3CN (10 mL) was added via syringe. The mixture was stirred under room temperature for 1 h. Then the substrate **1a** (2 mmol, 1.0 equiv), **2a** (4 mmol, 2.0 equiv), Et_4NCl (6 mmol, 3.0 equiv), anhydrous CH_3CN (10 mL) was added. The flask was equipped with a rubber stopper, graphite felt (4 cm x 2 cm x 1 cm) as cathode, Zn (3 cm x 1 cm x 0.3 cm) as anode. The Zn anode attached to a platinum wire and graphite felt cathode attached to a titanium wire. The mixture was stirred under 35 ^{o}C and constant current electrolysis (10 mA). After the reaction completed (TLC or GC-MS analysis, about 8 h), the mixture was extracted with ethyl acetate. The organic layers were washed with brine, dried over Na_2SO_4, filtered and concentrated. The residue was purified by chromatography on silica gel to afford the desire product **3a** (293 mg, 75%).

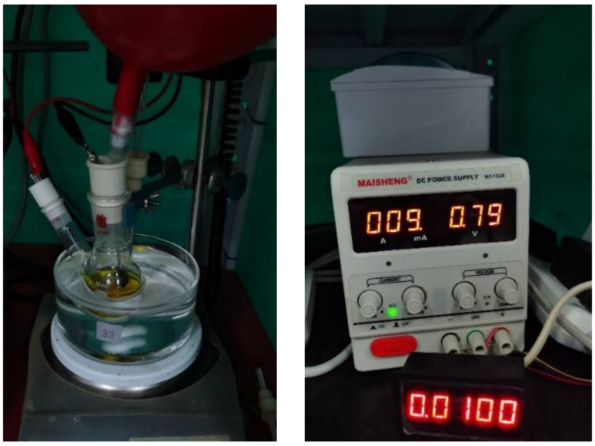

Supplementary Figure 2. **Reaction Setup of mmol scale reaction**

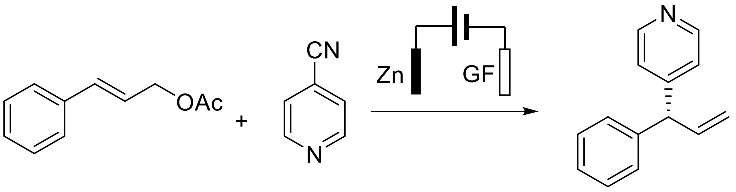

1a 10 mmol

2a 20 mmol

3a

A 100 mL three-necked flask was charged with $PdCl_2$ (132.8 mg, 0.75 mmol), (*R*) - DTBM-Segphos (0.94 g, 0.8 mmol) and a magnetic stir bar. The flask was evacuated and backfilled with argon for three times, and anhydrous CH_3CN (20 mL) was added via syringe. The mixture was stirred under room temperature for 1 h. Then the substrate **1a** (1.67 mL, 10 mmol), **2a** (2.08 g, 20 mmol), Et_4NCl (1.2 g, 7.2 mmol), MeOH (12 mL), and anhydrous CH_3CN (40 mL) was added. The flask was equipped with a rubber stopper, graphite felt (6 cm x 3 cm x 1.5 cm) as cathode, Zn (5 cm x 1.5 cm x 0.3 cm) as anode. The Zn anode attached to a platinum wire and graphite felt cathode attached to a titanium wire. The mixture was stirred under 35 ^{o}C and constant current electrolysis (30 mA). After the reaction completed (TLC or GC-MS analysis, about 12 h), the mixture was extracted with ethyl acetate. The organic layers were washed with brine, dried over Na_2SO_4, filtered and concentrated. The residue was purified by chromatography on silica gel to afford the desire product **3a** as a yellow oil (1.29 g, 66%).

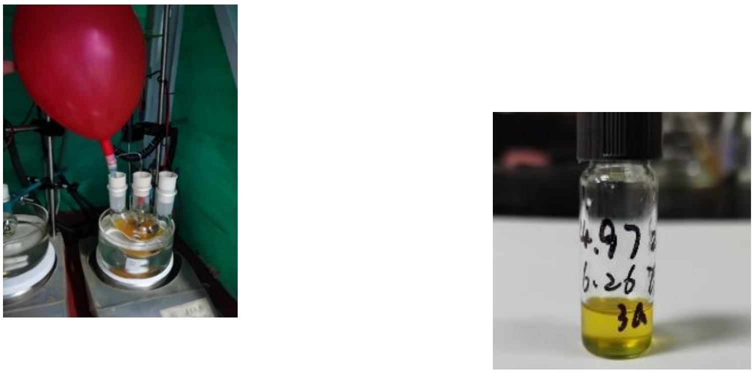

Supplementary Figure 3. **Reaction Setup of 10 mmol scale reaction**

1.3.4 Optimization of the conditions for 3a

Supplementary Table 1. **Screening of the solvent.**

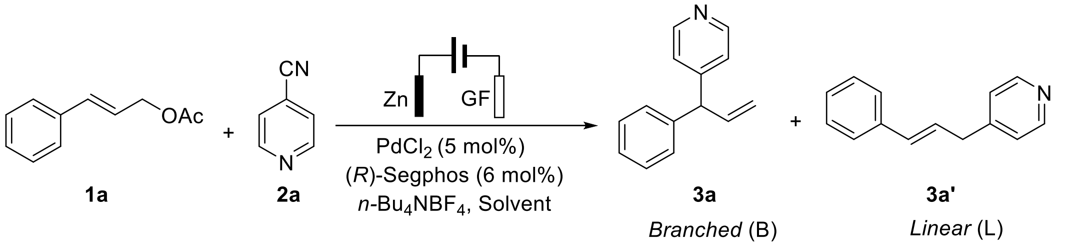

Entry[a]	Solvent	Yields (%)[b]	*ee* (%)[c]	B/L[b]
1	CH_3CN	trace	-	-
2	MeOH	12	36	6/1
3	EtOH	n.d.	-	-
4	DMF	54	11	2/1
5	DMSO	18	5	4/1
10	CH_3CN/THF (5/1)	trace	-	-
11	CH_3CN/MeOH (5/1)	60	33	6/1
12	CH_3CN/HFIP (5/1)	trace	-	-

[a] reaction condition: **1a** (0.2 mmol), **2a** (0.6 mmol), $PdCl_2$ (5 mol %), (*R*)-Segphos (6 mol %), *n*-Bu_4NBF_4 (0.1 mmol), solvent (6 mL) in an undivided cell with graphite felt (2 cm x 1 cm x 0.5 cm) as cathode, Zn (1.5 cm x 1 cm x 0.2 cm) as anode at rt in 20 mA for 2.5 h. [b] Yields were determined by ^{1}H NMR using CH_2Br_2 as an internal standard. [c] Enantiomeric excess (*ee*) values determined by HPLC on a chiral stationary phase.

Supplementary Table 2. **Screening of the electrode.**

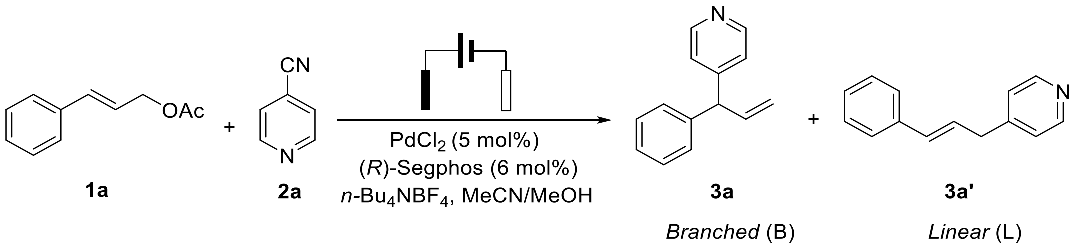

Entry[a]	Electrode	Yields (%)[b]	*ee* (%)[c]	B/L[b]
1	GF+/GF-	22		5/1
2	Pt+/GF-	trace	-	-
3	Pt+/Pt-	n.d.	-	-
4	Zn+/GF-	60	33	6/1
5	Zn+/Pt-	n.d.	-	-

[a] reaction condition: **1a** (0.2 mmol), **2a** (0.6 mmol), $PdCl_2$ (5 mol %), (*R*)-Segphos (6 mol %), MeCN/MeOH (5:1, v/v), *n*-Bu_4NBF_4 (0.1 mmol), in an undivided cell at rt in 20 mA for 2.5 h. [b] Yields were determined by ^{1}H NMR using CH_2Br_2 as an internal standard. [c] Enantiomeric excess (*ee*) values determined by HPLC on a chiral stationary phase.

Supplementary Table 3. **Screening of the temperature.**

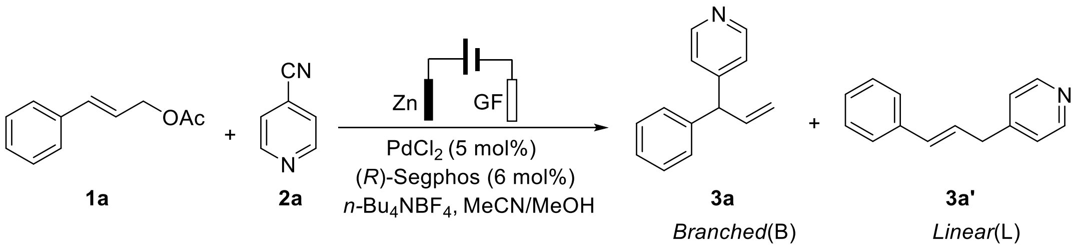

Entry	Temperature	NMR yields	*ee*%	B/L
1	25 °C	60	33	6/1
2	30 °C	70	45	6/1
3	35 °C	75	48	6/1
4	40 °C	61	48	5/1

[a] reaction condition: **1a** (0.2 mmol), **2a** (0.6 mmol), $PdCl_2$ (5 mol %), (*R*)-Segphos (6 mol %), MeCN/MeOH (5:1, v/v), *n*-Bu_4NBF_4 (0.1 mmol), in an undivided cell with graphite felt (2 cm x 1 cm x 0.5 cm) as cathode, Zn (1.5 cm x 1 cm x 0.2 cm) as anode in 20 mA for 2.5 h. [b] Yields were determined by ^1H NMR using CH_2Br_2 as an internal standard. [c] Enantiomeric excess (*ee*) values determined by HPLC on a chiral stationary phase.

Supplementary Table 4. **Evaluation of allylic compounds.**

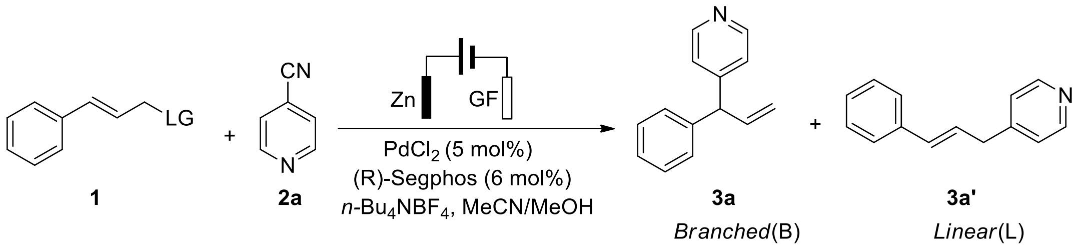

Entry [a]	"*LG*"	Yields (%)[b]	*ee* (%)[c]	B/L[b]
1	-OAc	75	48	6/1
2	-O-(C=NH)-CCl_3	trace	-	-
3	-$OCOCF_3$	n.d.	-	-
4	-OBoc	n.d.	-	-
5	-OBz	53	48	4/1

[a] reaction condition: **1** (0.2 mmol), **2a** (0.6 mmol), $PdCl_2$ (5 mol %), (*R*)-Segphos (6 mol %), MeCN/MeOH (5:1, v/v), *n*-Bu_4NBF_4 (0.1 mmol), in an undivided cell with graphite felt (2 cm x 1 cm x 0.5 cm) as cathode, Zn (1.5 cm x 1 cm x 0.2 cm) as anode at 35 °C in 20 mA for 2.5 h. [b] Yields were determined by ^1H NMR using CH_2Br_2 as an internal standard. [c] Enantiomeric excess (*ee*) values determined by HPLC on a chiral stationary phase.

Supplementary Table 5. **Screening of the electric current.**

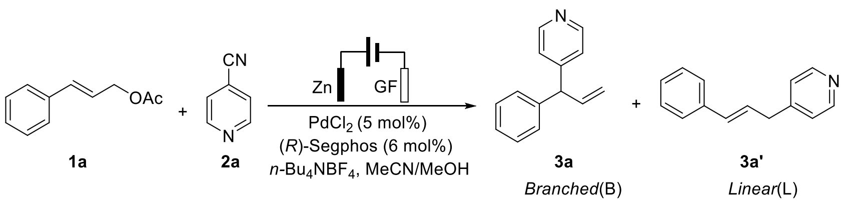

Entry [a]	I	Yields (%)[b]	*ee* (%)[c]	B/L[b]
1	24 mA	50	33	6/1
2	20 mA	75	48	6/1
3	10 mA	72	57	6/1
4	5 mA	62	63	6/1
5	2 mA	46	59	6/1

[a] reaction condition: **1a** (0.2 mmol), **2a** (0.6 mmol), $PdCl_2$ (5 mol %), (*R*)-Segphos (6 mol %), MeCN/MeOH (5:1, v/v), *n*-Bu_4NBF_4 (0.1 mmol), in an undivided cell with graphite felt (2 cm x 1 cm x 0.5 cm) as cathode, Zn (1.5 cm x 1 cm x 0.2 cm) as anode at 35 °C. [b] Yields were determined by ^{1}H NMR using CH_2Br_2 as an internal standard. [c] Enantiomeric excess (*ee*) values determined by HPLC on a chiral stationary phase.

Supplementary Table 6. **Screening of the Palladium catalysts.**

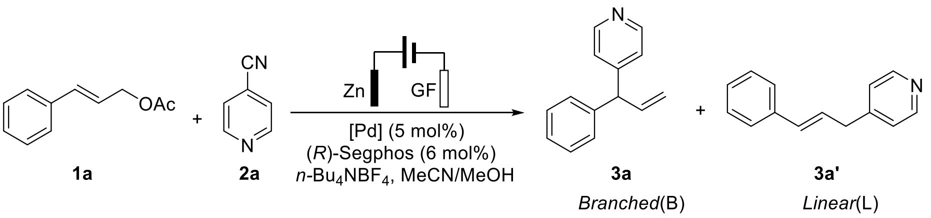

Entry [a]	[Pd]	Yields (%)[b]	*ee* (%)[c]	B/L[b]
1	$PdBr_2$	44	40	5/1
2	$Pd(OAc)_2$	18	43	4/1
3	$Pd_2(dba)_3$	38	45	4/1
4	$Pd(acac)_2$	63	60	5/1
5	$[PdCl(allyl)]_2$	68	57	5/1
6	$PdCl_2$	62	63	6/1
7	$Pd(TFA)_2$	53	58	5/1
8	$PdNO_3$	trace	-	-
9	**L1** $Pd(SbF_6)_2$	61	57	5/1
10	**L1** $Pd(BF_4)_2$	48	36	5/1
11	**L1** $Pd(OTf)_2$	23	48	4/1
12	**L1** $Pd(NTf)_2$	29	46	5/1
13	**L1** $Pd(BAr^F{}_4)_2$	73	58	6/1

[a] reaction condition: **1a** (0.2 mmol), **2a** (0.6 mmol), [Pd] (5 mol %), (*R*)-Segphos (6 mol %), MeCN/MeOH (5:1, v/v), *n*-Bu_4NBF_4 (0.1 mmol), in an undivided cell with graphite felt (2 cm x 1 cm x 0.5 cm) as cathode, Zn (1.5 cm x 1 cm x 0.2 cm) as anode at 35 °C in 5 mA. [b] Yields were determined by ^{1}H NMR using CH_2Br_2 as an internal standard. [c] Enantiomeric excess (*ee*) values determined by HPLC on a chiral stationary phase.

Supplementary Table 7. **Screening of the amount of 2a.**

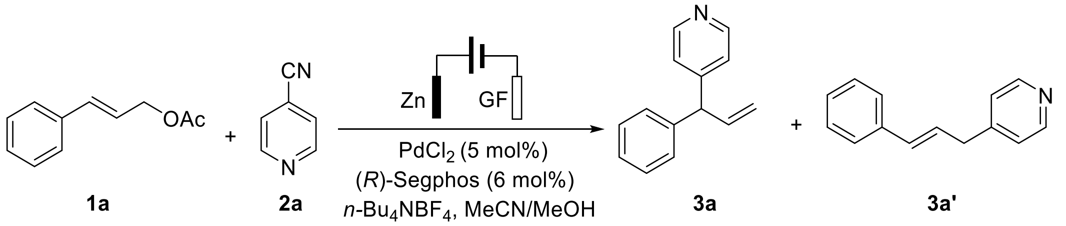

Entry [a]	x eq	Yields (%)[b]	*ee* (%)[c]	B/L[b]
1	1.5	65	55	6/1
2	3.0	62	63	6/1
3	5.0	61	62	5/1

[a] reaction condition: **1a** (0.2 mmol), **2a** (0.2x mmol), $PdCl_2$ (5 mol %), (*R*)-Segphos (6 mol %), MeCN/MeOH (5:1, v/v), *n*-Bu_4NBF_4 (0.1 mmol), in an undivided cell with graphite felt (2 cm x 1 cm x 0.5 cm) as cathode, Zn (1.5 cm x 1 cm x 0.2 cm) as anode at 35 °C in 5 mA. [b] Yields were determined by ^1H NMR using CH_2Br_2 as an internal standard. [c] Enantiomeric excess (ee) values determined by HPLC on a chiral stationary phase.

Supplementary Table 8. **Screening of the chiral ligands**.

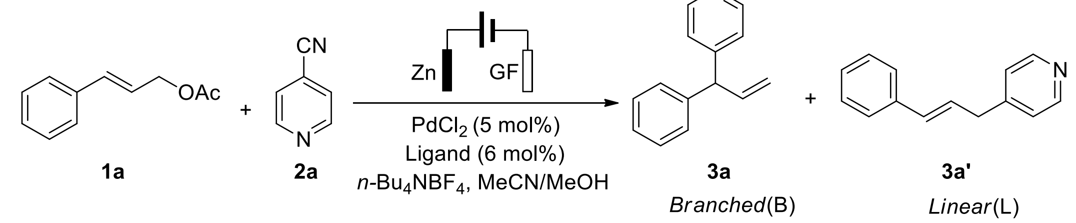

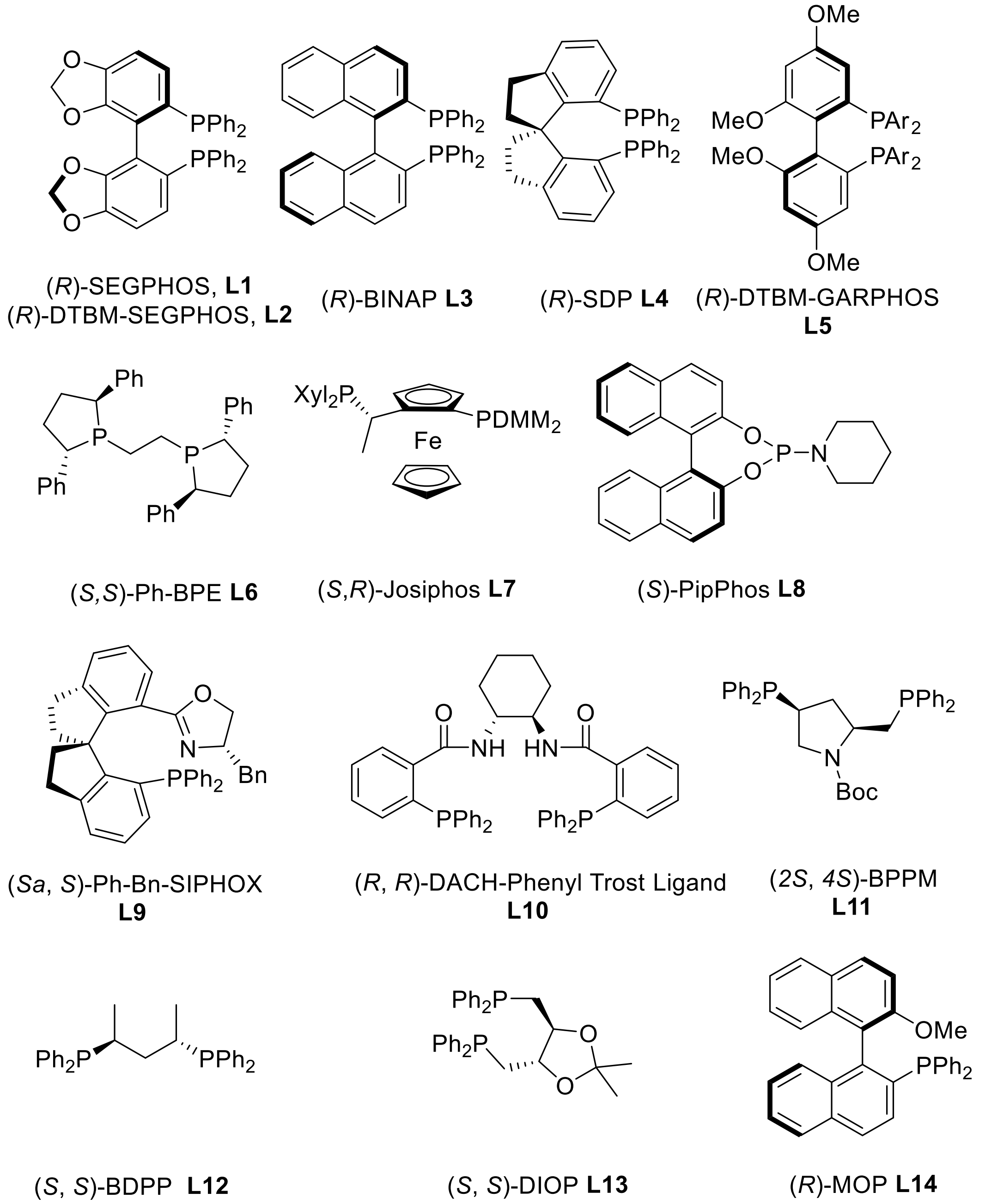

Entry[a]	Ligand	Yields (%)[b]	*ee* (%)[c]	B/L[b]
1	L1	62	63	6:1
2	L2	61	89	13/1
3	L3	73	50	6/1

4	L4	28	22	6/1
5	L5	66	90	10/1
6	L6	40	-77	4/1
7	L7	trace	-	-
8	L8	n.d.	-	-
9	L9	trace	-	-
10	L10	trace	-	-
11	L11	51	-10	3/1
12	L12	53	-8	5/1
13	L13	46	-11	4/1
14	L14	trace	-	-

[a] reaction condition: **1a** (0.2 mmol), **2a** (0.6 mmol), $PdCl_2$ (5 mol %), (*R*)-Segphos (6 mol %), MeCN/MeOH (5:1, v/v), *n*-Bu_4NBF_4 (0.1 mmol) in an undivided cell with graphite felt (2 cm x 1 cm x 0.5 cm) as cathode, Zn (1.5 cm x 1 cm x 0.2 cm) as anode at 35 °C in 5 mA. [b] Yields were determined by ^{1}H NMR using CH_2Br_2 as an internal standard. [c] Enantiomeric excess (*ee*) values determined by HPLC on a chiral stationary phase.

Supplementary Table 9**. Screening of the electrolyte.**

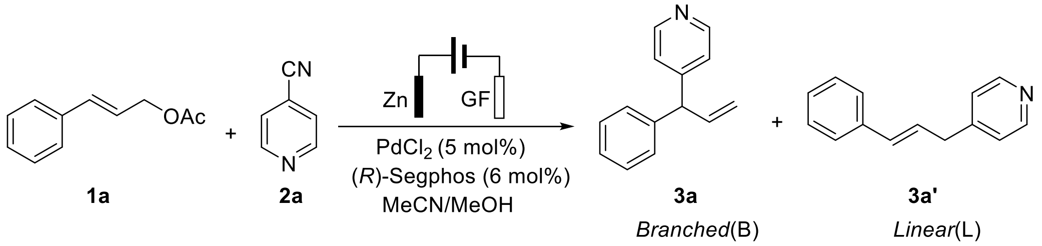

Entry [a]	Electrolyte	Yields (%)[b]	*ee* (%)[c]	B/L[b]
1	*n*-Bu_4NBF_4	62	63	6/1
2	*n*-Bu_4NPF_6	57	59	5/1
3	*n*-Bu_4NOAc	55	51	6/1
4	Et_4NCl	67	66	7/1
5	Et_4NBr	57	70	7/1
6	Et_4NBF_4	60	65	5/1
7	Et_4NClO_4	57	61	5/1
8	Et_4NOTs	56	59	5/1
9	*n*-Bu_4NBF_4 (1.0 equiv)	55	66	5/1
10	*n*-Bu_4NBF_4 (2.0 equiv)	61	63	4/1
11	Et_4NCl (1.0 equiv)	68	66	7/1
12	Et_4NCl (2.0 equiv)	70	67	8/1
13	Et_4NCl (3.0 equiv)	74	70	8/1

[a] reaction condition: **1a** (0.2 mmol), **2a** (0.2 mmol), $PdCl_2$ (5 mol %), **L** (6 mol %), MeCN/MeOH (5:1, v/v), electrolyte, in an undivided cell with graphite felt (2 cm x 1 cm x 0.5 cm) as cathode, Zn (1.5 cm x 1 cm x 0.2 cm) as anode at 35 °C in 5 mA. [b] Yields were determined by ^{1}H NMR using CH_2Br_2 as an internal standard. [c] Enantiomeric excess (*ee*) values determined by HPLC on a chiral stationary phase.

Supplementary Table 10. **Screening of the amount of $PdCl_2$.**

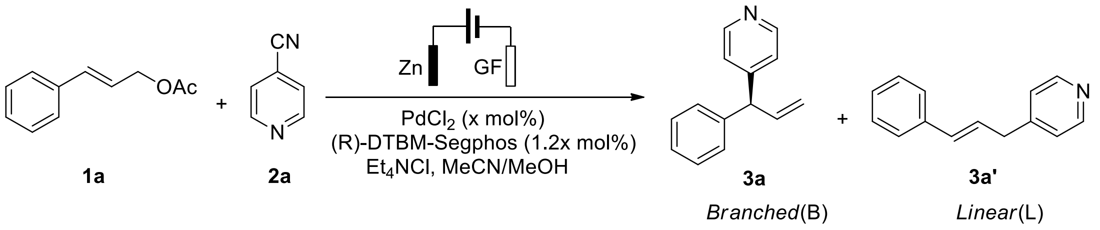

Entry[a]	x mol%	Yields (%)[b]	*ee* (%)[c]	B/L[b]
1	5	61	89	13/1
2	7.5	76	91	13/1
3	10	78	93	13/1

[a] reaction condition: **1a** (0.2 mmol), **2a** (0.6 mmol), $PdCl_2$ (x mol %), (R)-DTBM-Segphos (1.2x mol %), MeCN/MeOH (5:1, v/v), Et_4NCl (0.1 M), in an undivided cell with graphite felt (2 cm x 1 cm x 0.5 cm) as cathode, Zn (1.5 cm x 1 cm x 0.2 cm) as anode at 35 °C in 5 mA. [b] Yields were determined by ^1H NMR using CH_2Br_2 as an internal standard. [c] Enantiomeric excess (*ee*) values determined by HPLC on a chiral stationary phase.

Supplementary Table 11. **Further exploration of the electrode and additives.**

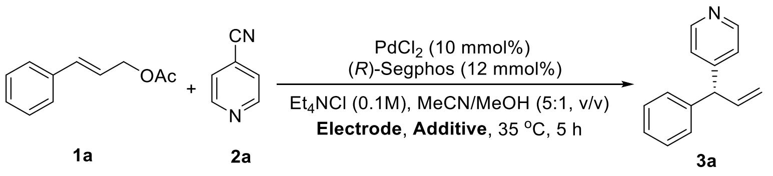

Entry	Electrode	Additive	Yield (%)
1	Fe+/C-	-	nd
2	Al+/C-	-	nd
3	Pt+/C-	-	nd
4	Mg+/C-	-	nd
5	C+/Pt-	-	nd
6	Fe+/C-	$ZnCl_2$ (3.0 equiv)	44
7	Al+/C-	$ZnCl_2$ (3.0 equiv)	68
8	Al+/C-	$ZnCl_2$ (0.2 equiv)	58

[a] reaction condition: **1a** (0.2 mmol), **2a** (0.6 mmol), $PdCl_2$ (10 mol %), (*R*)-Segphos (12 mol %), MeCN/MeOH (5:1, v/v), Et_4NCl (0.1 M), additive, in an undivided cell at 35 °C in 5 mA for 5h. [b] Yields were determined by ^1H NMR using CH_2Br_2 as an internal standard.

Supplementary Table 12. **Further exploration of the mode of electrolysis.**

When alternating current of square wave is applied with two graphite electrodes, the conversion did not take place. When the reaction mixture after AC condition (entry 3) was transferred to DC (Zn+/GF-) with a constant current of 5 mA for two hours, 40% of the target product **3a** was detected.

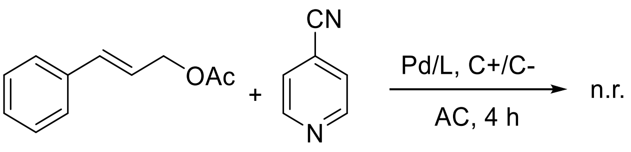

1a **2a**

Entry	AC	frequency	Yield
1	+2.5/-2.5	10	n.r.
2	+5/-5	50	n.r.
3	+5/-5	20	n.r.
4	+5/-5	5	n.r.

conditions: **1a** (0.2 mmol), **2a** (0.6 mmol), $PdCl_2$ (10 mol%), **L** (12 mol%), 5:1 (v/v) MeCN/MeOH 5 mL, Et_4NCl (0.1 M), 35 °C, square wave (50%/50%), the reactions were analyzed with GC-MS and ^1H NMR.

1.4 Derivation of the product 3

Procedure for the synthesis of carboxylic acid compound from 3r

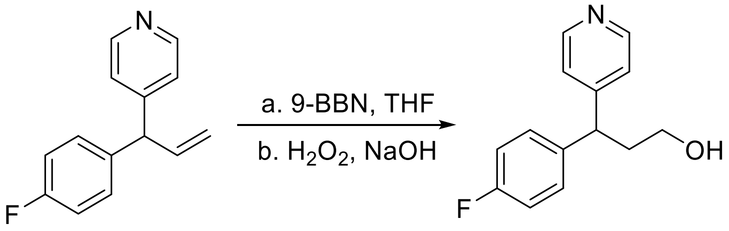

3r **S1**

0.5 M Borane tetrahydrofuran complex (4.0 mL, 2 mmol) was added to a solution of **3r** (213 mg, 1 mmol) in dried THF (5 mL) at 0 ºC under nitrogen. The mixture was allowed to warm to room temperature, and stirred for 1 h. Then 4.0 N NaOH (2.0 mL), followed by 30% H_2O_2 (2.0 mL) was then added to the reaction mixture and stirred for another 1 h. The mixture was extracted with ethyl acetate, the organic layers were washed with brine, dried over Na_2SO_4, filtered and concentrated. The residue was purified by chromatography on silica gel to afford the intermediate **S1** as a white solid (129 mg, 56%).

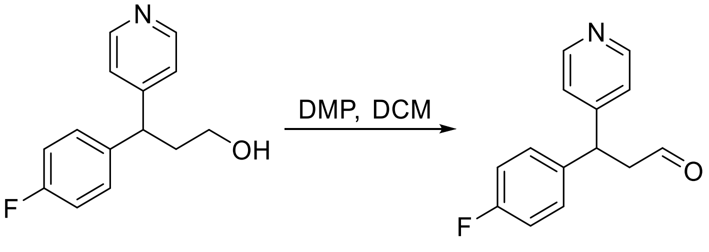

S1 **S2**

A flask was charged with **S1** (129 mg, 0.56 mmol), Dess-Martin's reagent (285 mg, 0.67 mmol), and DCM (5 mL) at 0 °C under nitrogen. The mixture was allowed to warm to room temperature, and stirred for 1 h. Until the alcohol was completely consumed, the mixture was extracted with DCM, the organic layers were washed with brine, dried over Na_2SO_4, filtered and concentrated. The residue was purified by chromatography on silica gel to afford the intermediate **S2** (115 mg, 90%) as a yellow oil.

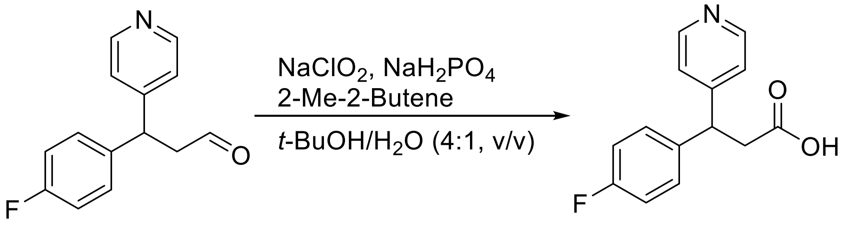

S2 **5**

The intermediate **S2** (45.8 mg, 0.2 mmol, 1.0 equiv) was dissolved in a mixture of tert-butyl alcohol (2 mL) and 2-methyl-2-butene (112 mg, 1.6 mmol, 8.0 equiv). The resulting solution was stirred and cooled to 0°C. A mixture of $NaClO_2$ (54.2 mg, 0.6 mmol, 3.0 equiv) and NaH_2PO_4 (165.6 mg, 1.2 mmol, 6.0 equiv) was dissolved in water (0.5 mL) and then added to the reaction. The mixture was stirred and monitored via TLC until complete consumption of the aldehyde was observed. The mixture was concentrated under vacuum, and dilute with ethyl acetate. The combined organic layers were washed with brine, dried over Na_2SO_4, filtered and concentrated. The residue was purified by flash chromatography to afford the desired product **5** as a white solid (49 mg, 86%).

Procedure for the synthesis of primary amine from 3a

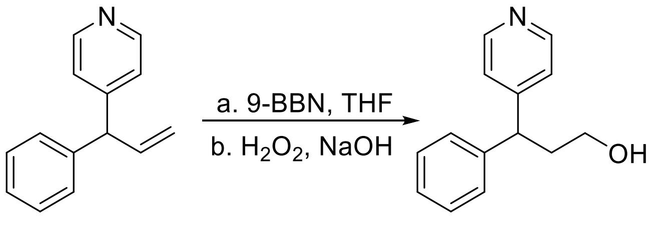

3a **S3**

0.5 M Borane tetrahydrofuran complex (4.0 mL, 2 mmol) was added to a solution of **3a** (195 mg, 1 mmol) in dried THF (5 mL) at 0 °C under nitrogen. The mixture was allowed to warm to room temperature, and stirred for 1 h. Then 4.0 N NaOH (2.0 mL), followed by 30% H_2O_2 (3.0 mL) was then added to the reaction mixture and stirred for another 1 h. The mixture was extracted with ethyl acetate, the organic layers were washed with brine, dried over Na_2SO_4, filtered and concentrated. The residue was purified by chromatography on silica gel to afford the intermediate **S3** as a white solid (145 mg, 68%).

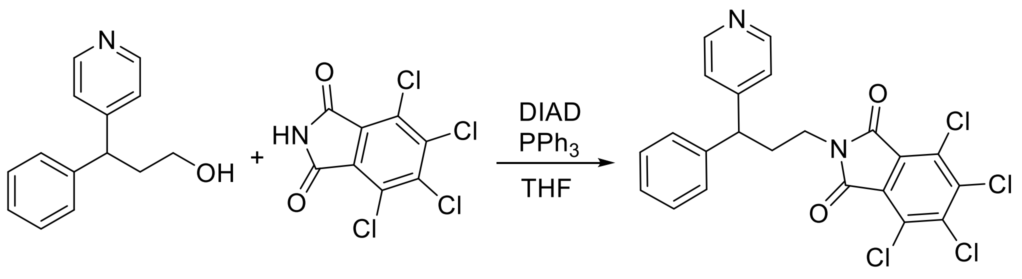

S3 **S4**

A flask was charged with **S3** (106.5 mg, 0.5 mmol), 4,5,6,7-tetrachloroisoindoline-1,3-dione (141.5 mg, 0.5 mmol), PPh_3 (196.5 mg, 0.75 mmol) and anhydrous THF (5 mL) at 0 ^{o}C under nitrogen. The mixture was allowed to warm to room temperature, and stirred for 30 min. DIAD (151.5 mg, 0.75 mmol) was then added and the mixture was stirred until the reaction was finished (monitored by TLC). The reaction was diluted with H_2O and the resulting mixture was extracted with ethyl acetate for three times. The combined organic layers were washed with brine, dried over Na_2SO_4, filtered and concentrated. The residue was purified by flash chromatography to afford the intermediate **S4** as white solid (192 mg, 90%).

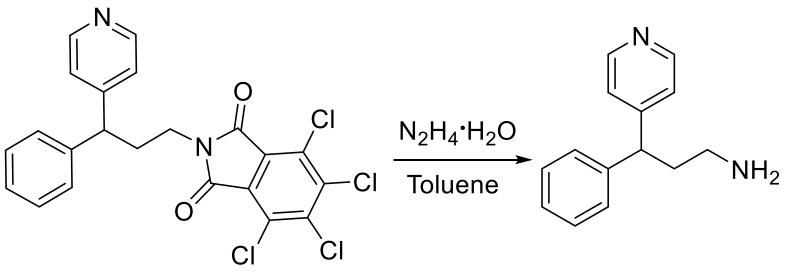

S4 **6**

A flask was charged with **S4** (192 mg), hydrazinium hydroxide (2 mL), and toluene (5 mL) at room temperature under nitrogen. The mixture was allowed to heated to 95 ^{o}C, and stirred for 2 h. Until the starting material **S4** was completely consume (monitored by TLC), the mixture was concentrated under vacuum. The residue was purified by chromatography on silica gel to afford the desire product **6** as yellow oil (70 mg, 78%).

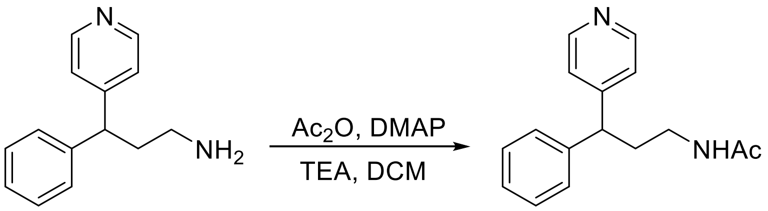

6 **S5**

A flask was charged with **6** (21 mg, 0.1 mmol), acetic acid (14 μL, 0.15 mmol), TEA (21 μL, 0.15 mmol), and DMAP (1.5 mg, 0.01 mmol) at room temperature under nitrogen. The mixture was allowed to stirred for 2 h. Until the starting material was completely consume (monitored by TLC), the mixture was concentrated under vacuum. The residue was purified by chromatography on silica gel to afford the desire product **S5** as colorless oil (22 mg, 89%).

2. Supplementary Discussion

2.1 Preparation of π-allylpalladium complex

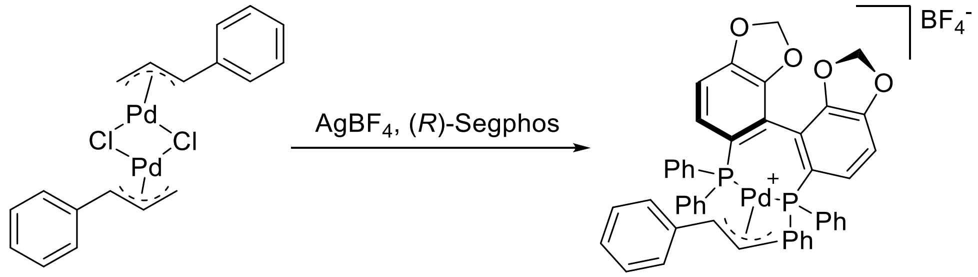

The substrate was prepared following a report procedure with a small modifications: [4-6] To a 50 mL of seal tube equipped with a stir bar was added [Pd(l-Phenylallyl)C1]$_2$ (196 mg, 0.38 mmol) and silver tetrafluoroborate (185 mg, 0.95 mmol). The flask was evacuated and backfilled with argon for three times, and CH_2Cl_2 (5 mL) was added via syringe. Then the mixture was allowed stirred for 1 h. Next, a solution of (R)-Segphos (464 mg, 0.76 mmol) in CH_2Cl_2 (10 mL) was added at room temperature. The mixture was allowed to stirring at room temperature for 2 h. The pale yellow suspension was centrifugalized, and the solution was concentrated under reduced pressure to give the desire product as a pale yellow powder (297 mg, 85%).

2.2 Synthesis of the charge-tagged diphosphate ligand L9

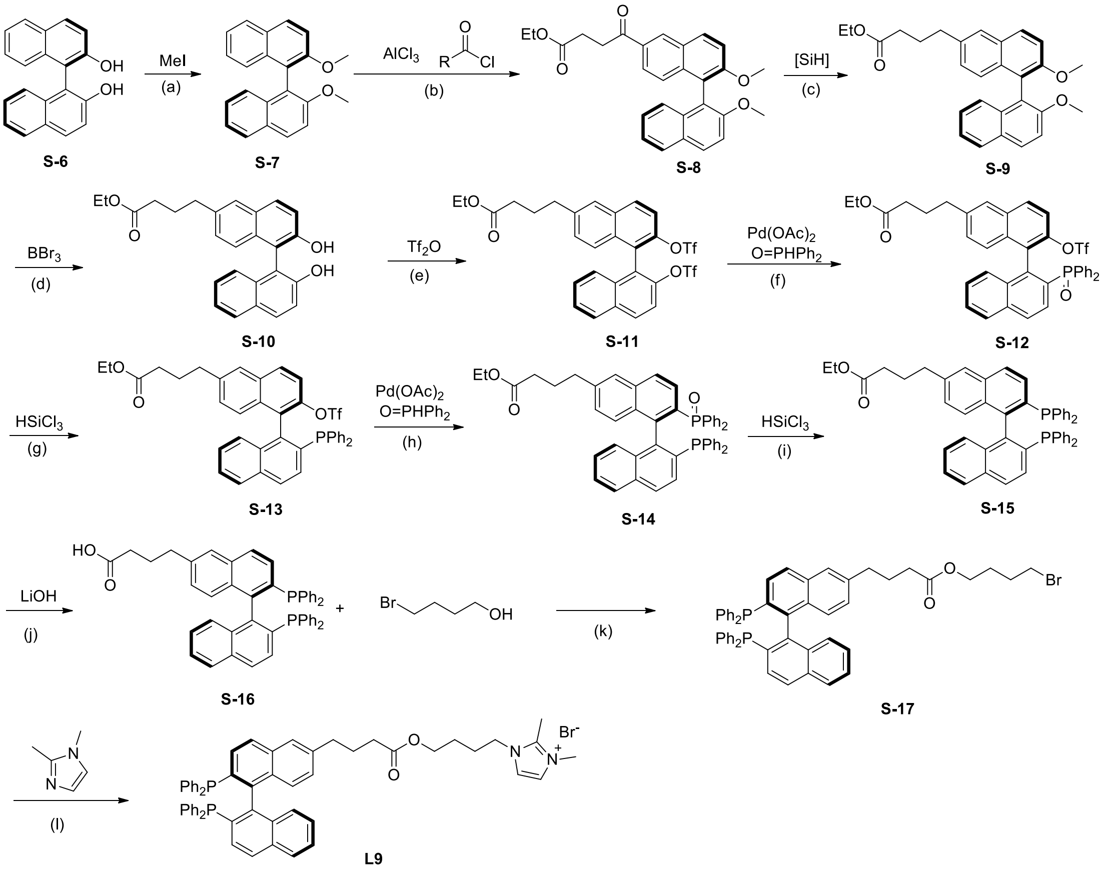

(a) MeI, acetone, reflux;(b) $AlCl_3$, ethyl 4-chloro-4-oxobutanoate, DCM;(c) Et_3SiH,CF_3COOH, DCM;(d) BBr_3,DCM; (e) Tf_2O, 2,6-lutidine, DMAP, DCM; (f) $Pd(OAc)_2$, dppb, diphenylphosphine oxide, iPr_2NEt, DMSO; (g) $HSiCl_3$, TEA,Toluene;(h) $Pd(OAc)_2$, dppb, diphenylphosphine oxide, iPr_2NEt, DMSO; (i) $HSiCl_3$, TEA,Toluene;(j) LiOH, THF/H_2O = 1:1; (k) EDCI, DMAP, iPr_2NEt, 4-bromobutan-1-ol, DCM;(l) 1,2-dimethyl-1*H*-imidazole, 115 ^oC, Toluene.

Supplementary Figure 4. **The synthesis of charge-tagged diphosphate ligand L9**

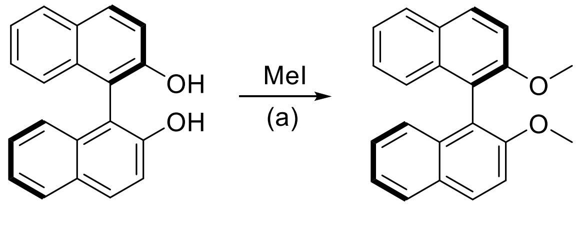

S6 **S7**

To a stirred solution of (R)-binaphthol (10 g, 35 mmol) in anhydrous acetone (200 mL) were added K_2CO_3 (14.5 g, 105 mmol) and methyl iodide (6.5 mL, 105 mmol). The mixture was heated at 60 ^oC for 24 h. After the reaction completed (TLC), the mixture was cooling to room temperature, the solution was concentrated under reduced pressure and the residual solids dissolved in CH_2Cl_2 and H_2O. The layers were separated, and the aqueous phase was extracted with CH_2Cl_2. The combined organic layers were washed with brine, dried over Na_2SO_4, filtered and concentrated.

The pale yellow solid which was purified with MeOH to afford the title product **S7** (9.9 g, 90%) as a white solid. ^{1}H NMR (400 MHz, Chloroform-*d*) δ 7.99 (d, *J* = 9.0 Hz, 2H), 7.88 (dd, *J* = 8.2, 1.2 Hz, 2H), 7.47 (d, *J* = 9.0 Hz, 2H), 7.33 (t, *J* = 7.4 Hz, 2H), 7.22 (t, *J* = 7.6 Hz, 2H), 7.12 (d, *J* = 8.4 Hz, 2H), 3.78 (s, 6H).

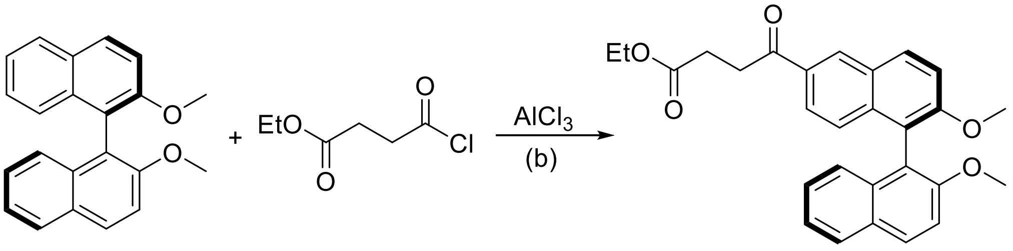

S7 **S8**

$AlCl_3$ (5.1 g, 37.8 mmol) was added in CH_2Cl_2 at 0 °C under nitrogen. Then **S7** (9.9 g, 31.5 mmol) was added in the solution. The red solution was stirred for 30 min, and added ethyl succinyl chloride (4.9 mL, 34.6 mmol). The resulting brown solution was allowed to warmed to room temperature, and stirred for 24 h. After the reaction completed (TLC), the mixture was cooling to 0 °C, and quenched with H_2O. The layers were separated, and the aqueous phase was extracted with CH_2Cl_2. The combined organic layers were washed with brine, dried over Na_2SO_4, filtered and concentrated. The residue was purified by chromatography on silica gel to afford the desire product **S8** as a yellow oil (8.3 g, 60%). ^{1}H NMR (400 MHz, Chloroform-*d*) δ 8.59 (s, 1H), 8.13 (d, *J* = 9.0 Hz, 1H), 8.02 (d, *J* = 9.0 Hz, 1H), 7.90 (d, *J* = 8.2 Hz, 1H), 7.81 (d, *J* = 8.9 Hz, 1H), 7.54 (d, *J* = 9.1 Hz, 1H), 7.48 (d, *J* = 9.0 Hz, 1H), 7.34 (d, *J* = 7.6 Hz, 1H), 7.25 (t, *J* = 7.6 Hz, 1H), 7.20 (d, *J* = 9.0 Hz, 1H), 7.10 (d, *J* = 8.5 Hz, 1H), 4.20 (q, *J* = 7.2 Hz, 2H), 3.80 (d, *J* = 13.6 Hz, 6H), 3.43 (t, *J* = 6.7 Hz, 2H), 2.82 (t, *J* = 6.7 Hz, 2H), 1.29 (q, *J* = 3.8 Hz, 3H).

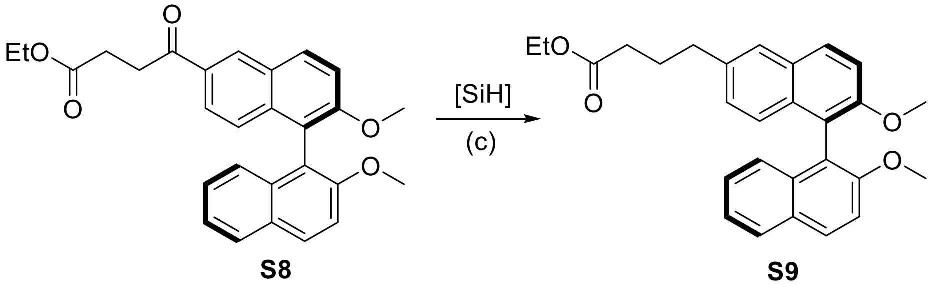

S8 (8.3 g, 18.9 mmol) was added in CH_2Cl_2 at 0 °C under nitrogen, trifluoroacetic acid (20 mL) and triethylsilane (8.0 mL). The mixture was allowed to warmed to room temperature, and stirred for 24 h. After the reaction completed (TLC), the mixture was cooling to 0 °C, and quenched with H_2O. Then neutralized with saturated aqueous Na_2CO_3, and the aqueous phase was extracted with CH_2Cl_2. The combined organic layers were washed with brine, dried over Na_2SO_4, filtered and concentrated. The residue was purified by chromatography on silica gel to afford the desire product **S9** as a pale yellow solid (7.3 g, 90%). ^{1}H NMR (400 MHz, Chloroform-*d*) δ 7.97 (d, *J* = 9.0 Hz, 1H), 7.90 (d, *J* = 9.0 Hz, 1H), 7.86 (d, *J* = 8.1 Hz, 1H), 7.64 (s, 1H), 7.45 (t, *J* = 8.9 Hz, 2H), 7.31 (ddd, *J* = 8.1, 6.7, 1.3 Hz, 1H), 7.21 (ddd, *J* = 8.1, 6.8, 1.3 Hz, 1H), 7.11 (d, *J* = 8.5 Hz, 1H), 7.09 –

7.01 (m, 2H), 4.12 (q, *J* = 7.1 Hz, 2H), 3.76 (d, *J* = 7.0 Hz, 6H), 2.76 (t, *J* = 7.5 Hz, 2H), 2.35 (t, *J* = 7.5 Hz, 2H), 2.01 (p, *J* = 7.7 Hz, 2H), 1.24 (t, *J* = 7.1 Hz, 3H).

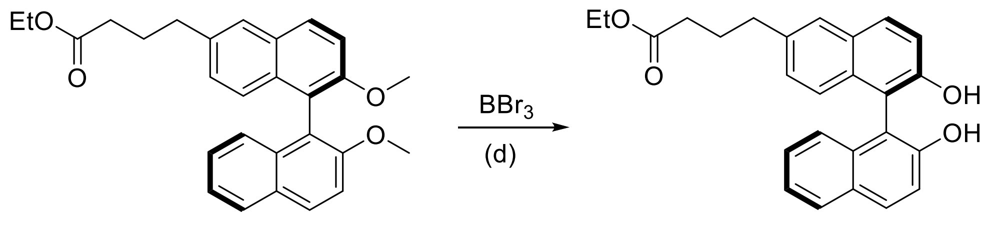

S9 **S10**

S9 (7.3 g, 17 mmol) was added in CH_2Cl_2 at -78 °C under nitrogen. BBr_3 (3.7 mL, 38.2 mmol) was added. The mixture was stirred over 8 h, and then allowed to warm to 0 °C. After the reaction completed (TLC), quenched with saturated aqueous $NaHCO_3$ and the aqueous phase was extracted with CH_2Cl_2. The layers were separated, and the aqueous phase was extracted with CH_2Cl_2. The combined organic layers were washed with brine, dried over Na_2SO_4, filtered and concentrated. The residue was purified by chromatography on silica gel to afford the desire product **S10** as a pale yellow solid (5.4 g, 80%). ^{1}H NMR (400 MHz, Chloroform-*d*) δ 7.95 (d, *J* = 8.9 Hz, 1H), 7.92 – 7.88 (m, 1H), 7.86 (d, *J* = 8.9 Hz, 1H), 7.67 (d, *J* = 1.7 Hz, 1H), 7.42 – 7.35 (m, 2H), 7.32 (t, *J* = 8.1 Hz, 2H), 7.22 – 7.14 (m, 2H), 7.11 (d, *J* = 8.6 Hz, 1H), 5.33 (d, *J* = 24.6 Hz, 2H), 4.12 (q, *J* = 7.1 Hz, 2H), 2.79 (t, *J* = 7.6 Hz, 2H), 2.35 (t, *J* = 7.5 Hz, 2H), 2.08 – 1.98 (m, 2H), 1.27 (t, *J* = 7.1 Hz, 3H).

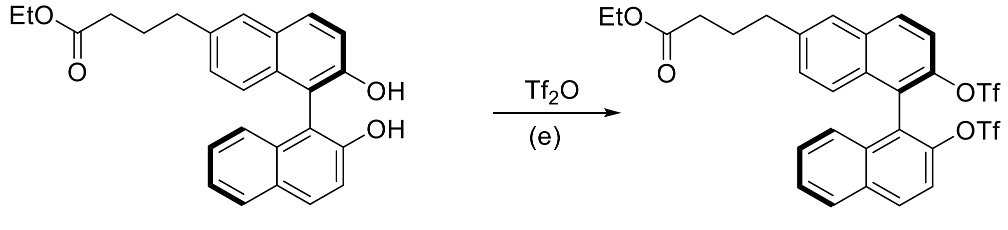

S10 **S11**

To a mixture of **S10** (5.4 g, 13.6 mmol), 2,6-lutidine (4.7 mL, 40.5 mmol), and DMAP (333.8 mg, 2.7 mmol) in CH_2Cl_2 at 0 °C was added trifluoromethanesulfonic anhydride (5.9 mL, 35.3 mmol). The mixture was allowed to warmed to room temperature, and stirred for 12 h. After the reaction completed (TLC), quenched with saturated aqueous $NaHCO_3$ and the aqueous phase was extracted with CH_2Cl_2. The layers were separated, and the aqueous phase was extracted with CH_2Cl_2. The combined organic layers were washed with 2 M aqueous HCl, brine, dried over Na_2SO_4, filtered and concentrated. The residue was purified by chromatography on silica gel to afford the desire product **S11** as a pale yellow oil (8.6 g, 95%). ^{1}H NMR (400 MHz, Chloroform-*d*) δ 8.16 (d, *J* = 9.1 Hz, 1H), 8.10 (d, *J* = 9.1 Hz, 1H), 8.03 (d, *J* = 8.2 Hz, 1H), 7.82 (s, 1H), 7.70 – 7.57 (m, 3H), 7.45 (t, *J* = 7.7 Hz, 1H), 7.35 – 7.26 (m, 2H), 7.23 (d, *J* = 9.0 Hz, 1H), 4.17 (q, *J* = 7.1 Hz, 2H), 2.87 (t, *J* = 7.6 Hz, 2H), 2.40 (t, *J* = 7.4 Hz, 2H), 2.15 – 2.05 (m, 2H), 1.28 (t, *J* = 7.1 Hz, 3H).

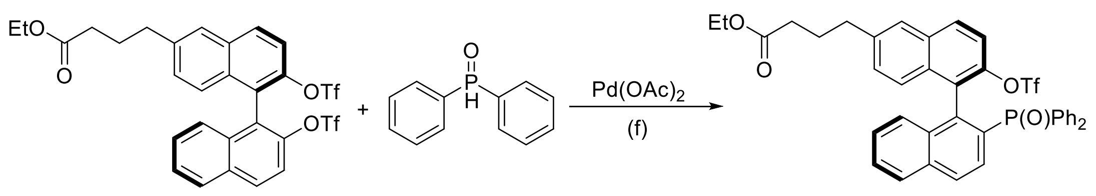

S11 **S12**

A solution of $Pd(OAc)_2$ (270.0 mg, 1.2 mmol) and dppp (639.0 mg, 1.5 mmol) in DMSO (8.0 mL) was stirred at room temperature for 1h under nitrogen. Subsequently, **S11** (4.0 g, 6.0 mmol), diphenylphosphine oxide (3.6 g, 18.0 mmol) and DIPEA (6.3 mL, 36.0 mmol) were added. The reaction was stirred at 100 °C and stirred for 8 h. The reaction was quenched with H_2O. The solution was extracted with CH_2Cl_2 (3 × 5 mL), washed with brine, and dried over Na_2SO_4, filtered and concentrated. The residue was purified by chromatography on silica gel to afford the desire product **S12** as yellow oil (3.5 g, 82%). ^{1}H NMR (400 MHz, Chloroform-*d*) δ 8.06 – 7.81 (m, 3H), 7.77 – 7.54 (m, 4H), 7.52 – 7.37 (m, 6H), 7.37 – 7.29 (m, 3H), 7.22 – 6.88 (m, 4H), 4.15 (dq, *J* = 14.3, 7.1 Hz, 2H), 2.80 (dt, *J* = 21.3, 7.6 Hz, 2H), 2.36 (dt, *J* = 12.4, 7.4 Hz, 2H), 2.11 – 1.94 (m, 2H), 1.28 (dt, *J* = 12.3, 7.1 Hz, 3H).

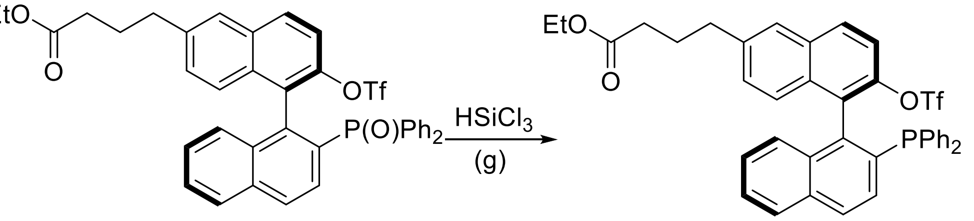

S12 **S13**

A solution of **S12** (3.5 g, 4.9 mmol) and Et_3N (6.6 mL, 47.2 mmol) in 30 mL toluene was stirred at 0 °C under nitrogen, then $HSiCl_3$ (3.4 mL, 33.7 mmol) was added, and allowed to heated to100 °C. The mixture was stirred for 12 h, after the reaction completed (TLC), quenched with saturated aqueous Na_2CO_3 and the aqueous phase was extracted with CH_2Cl_2. The layers were separated, and the aqueous phase was extracted with CH_2Cl_2. The combined organic layers were washed with brine, dried over Na_2SO_4, filtered and concentrated. The residue was purified by chromatography on silica gel to afford the desire product **S13** as colorless oil (2.3 g, 66%). ^{1}H NMR (400 MHz, Chloroform-*d*) δ 8.12 – 7.90 (m, 3H), 7.73 (d, *J* = 6.4 Hz, 1H), 7.59 – 7.46 (m, 3H), 7.40 – 7.27 (m, 6H), 7.26 – 7.18 (m, 2H), 7.15 (t, *J* = 7.6 Hz, 3H), 7.08 – 6.86 (m, 3H), 4.23 – 4.12 (m, 2H), 2.81 (dt, *J* = 15.9, 7.6 Hz, 2H), 2.37 (t, *J* = 7.5 Hz, 2H), 2.05 (ddd, *J* = 18.5, 8.5, 5.1 Hz, 2H), 1.29 (dt, *J* = 10.7, 7.1 Hz, 3H).

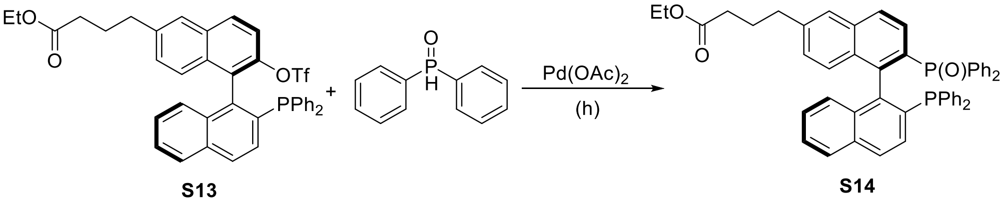

S13 **S14**

A solution of $Pd(OAc)_2$ (147.9 mg, 0.66 mmol) and dppp (351.6 mg, 0.82 mmol) in DMSO (6.0 mL) was stirred at room temperature for 1h under nitrogen. Subsequently, **S13** (2.3 g, 3.2 mmol), diphenylphosphine oxide (1.3 g, 6.6 mmol) and DIPEA (2.3 mL, 13.1 mmol) were added. The reaction was stirred at 100 °C and stirred for 8 h. The reaction was quenched with H_2O. The solution was extracted with CH_2Cl_2, washed with brine, and dried over Na_2SO_4, filtered and concentrated. The residue was purified by chromatography on silica gel to afford the desire product **S14** as colorless oil (1.8 g, 75%). ^{1}H NMR (400 MHz, Chloroform-*d*) δ 8.03 – 7.80 (m, 2H), 7.79 – 7.66 (m, 2H), 7.65 – 7.57 (m, 3H), 7.51 – 7.24 (m, 13H), 7.20 – 7.09 (m, 3H), 7.09 – 6.96 (m, 4H), 6.94 – 6.82 (m, 1H), 6.79 – 6.68 (m, 2H), 6.54 (s, 1H), 4.21 – 4.15 (m, 2H), 2.70 (q, *J* = 7.7 Hz, 2H), 2.33 (dt, *J* = 20.4, 7.5 Hz, 2H), 2.02 – 1.90 (m, 2H), 1.29 (s, 3H).

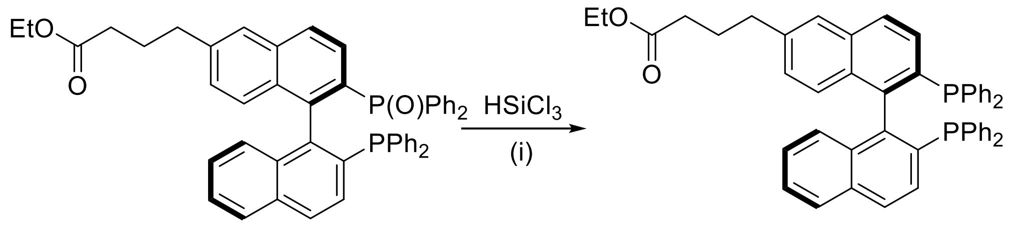

S14 **S15**

A solution of **S14** (1.8 g, 2.4 mmol) and Et_3N (1.8 mL, 12.6 mmol) in 20 mL toluene was stirred at 0 °C under nitrogen, then $HSiCl_3$ (0.9 mL, 9 mmol) was added, and allowed to heated to100 °C. The mixture was stirred for 12 h, after the reaction completed (TLC), quenched with saturated aqueous Na_2CO_3 and the aqueous phase was extracted with CH_2Cl_2. The layers were separated, and the aqueous phase was extracted with CH_2Cl_2. The combined organic layers were washed with brine, dried over Na_2SO_4, filtered and concentrated. The residue was purified by chromatography on silica gel to afford the desire product **S15** as colorless oil (1.3 g, 76%). ^{1}H NMR (400 MHz, Chloroform-*d*) δ 7.55 (d, *J* = 8.5 Hz, 1H), 7.49 (dd, *J* = 8.4, 4.0 Hz, 2H), 7.26 (s, 1H), 7.10 (dt, *J* = 8.5, 2.6 Hz, 2H), 7.01 (t, *J* = 7.4 Hz, 1H), 6.90 – 6.69 (m, 19H), 6.59 (t, *J* = 7.6 Hz, 1H), 6.52 (d, *J* = 8.4 Hz, 1H), 6.37 (s, 2H), 3.81 (q, *J* = 7.1 Hz, 2H), 2.37 (t, *J* = 7.5 Hz, 2H), 1.96 (t, *J* = 7.5 Hz, 2H), 1.67 – 1.56 (m, 2H), 0.93 (t, *J* = 7.1 Hz, 3H).

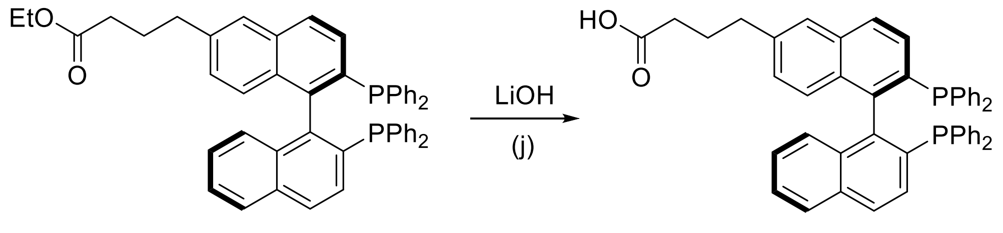

S15 **S16**

To a solution of **S15** (1.3 g, 1.8 mmol) in THF (8 mL) was added 8 mL of aqueous LiOH (2.2 g, 90.0 mmol) and the mixture heated at reflux for 20 h. After being cooled to room temperature, the solution was acidified to pH 1-3 with 2 M aqueous HCl and extracted with CH_2Cl_2. The layers were separated, and the aqueous phase was extracted with CH_2Cl_2. The combined organic layers

were washed with brine, dried over Na_2SO_4, filtered and concentrated. The residue was purified by chromatography on silica gel to afford the desire product **S16** as a white solid (1.1 g, 90%). ^1H NMR (400 MHz, Chloroform-*d*) δ 7.91 (d, *J* = 8.5 Hz, 1H), 7.85 (dd, *J* = 8.4, 3.2 Hz, 2H), 7.62 (s, 1H), 7.46 (dd, *J* = 8.5, 2.5 Hz, 2H), 7.37 (t, *J* = 7.2 Hz, 1H), 7.26 – 7.04 (m, 19H), 6.98 – 6.92 (m, 1H), 6.88 (d, *J* = 8.5 Hz, 1H), 6.73 (d, *J* = 2.1 Hz, 2H), 2.74 (t, *J* = 7.5 Hz, 2H), 2.36 (t, *J* = 7.5 Hz, 2H), 1.99 (p, *J* = 7.4 Hz, 2H).

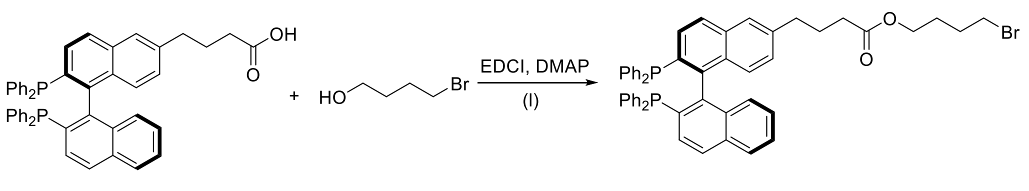

S16 **S17**

To a mixture of **S16** (200 mg, 0.3 mmol), 4-bromobutan-1-ol (32.8 uL, 0.36 mmol), DMAP (4.0 mg, 0.03 mmol) in CH_2Cl_2 at 0 ºC was added EDCI (86 mg, 0.45 mmol). The mixture was allowed to warmed to room temperature, and stirred for 1 h. Then DIPEA (0.26 mL, 1.5 mmol) was added, and stirred for 8h, after the reaction completed (TLC), diluted with CH_2Cl_2. The layers were separated, and the aqueous phase was extracted with CH_2Cl_2. The combined organic layers were washed with 2 M aqueous HCl, brine, dried over Na_2SO_4, filtered and concentrated. The residue was purified by chromatography on silica gel to afford the desire product **S17** as a colorless oil (189.5 mg, 75%).

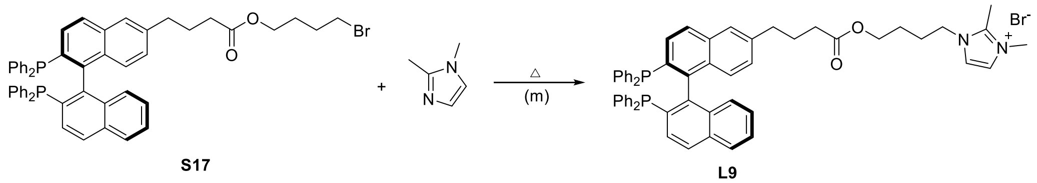

S17 **L9**

To a mixture of **S17** (189.5 mg, 0.23 mmol) in toluene was added 1,2-dimethyl-1H-imidazole (64.8 mg, 0.68 mmol) under nitrogen. The mixture was allowed to heated to 115Oc, and stirred for 12h. After the reaction completed (TLC), the mixture was directed removal of the solvent under reduced pressure. The residue was purified by chromatography on silica gel to afford the desire product **L9** as a pale yellow oil (95.1 mg, 45%). ^1H NMR (400 MHz, Chloroform-*d*) δ 7.88 (d, *J* = 8.5 Hz, 1H), 7.82 (d, *J* = 8.3 Hz, 2H), 7.60 (s, 1H), 7.51 (s, 2H), 7.44 (dt, *J* = 8.6, 2.2 Hz, 2H), 7.33 (d, *J* = 7.5 Hz, 1H), 7.28 – 7.21 (m, 1H), 7.20 – 7.02 (m, 19H), 6.90 (d, *J* = 7.2 Hz, 1H), 6.85 (s, 1H), 6.72 (s, 2H), 4.26 (t, *J* = 7.4 Hz, 2H), 4.11 (t, *J* = 6.2 Hz, 2H), 3.89 (s, 3H), 2.72 (d, *J* = 15.6 Hz, 5H), 2.31 (t, *J* = 7.6 Hz, 2H), 1.96 – 1.85 (m, 4H), 1.77 – 1.70 (m, 2H). ^{13}C NMR (101 MHz, Chloroform-*d*) δ 173.35, 143.84, 139.44, 134.23, 134.14, 134.03, 133.93, 133.30, 132.86, 132.69, 130.68, 130.37, 128.32, 128.28, 128.02, 127.98, 127.92, 127.61, 127.55, 127.50, 127.32, 126.92, 126.43, 126.35, 125.71, 122.85, 121.10, 63.06, 48.32, 35.93, 34.95, 33.41, 26.40, 26.04, 25.54, 10.75. ^{31}P NMR (162 MHz, Chloroform-*d*) δ -15.32 – -15.71 (m). **HRMS (ESI)** m/z: $[M]^+$ Calcd for $C_{57}H_{53}N_2O_2P_2^+$ 859.3577; Found 859.3582.

2.3 HRMS analysis of reaction mixture

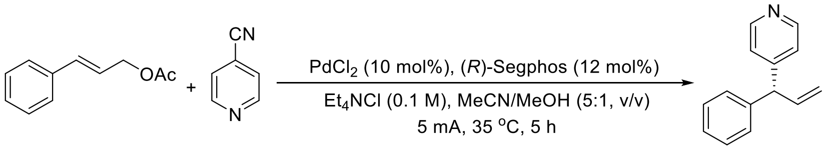

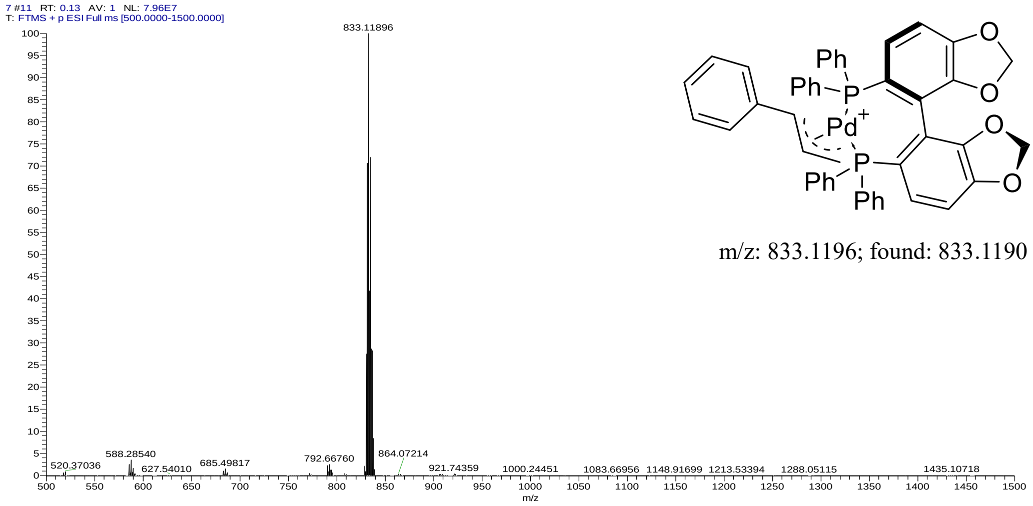

Supplementary Figure 5. **HRMS analysis of reaction mixture at 10th minute.**

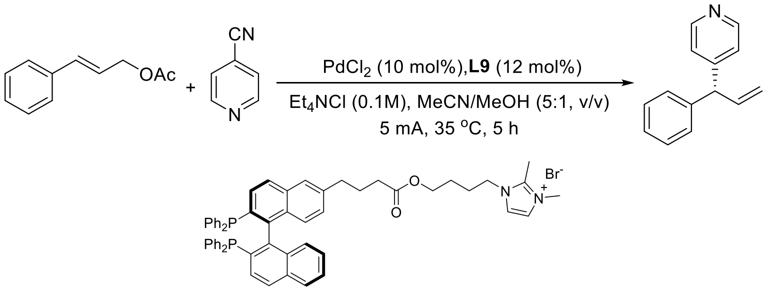

L9

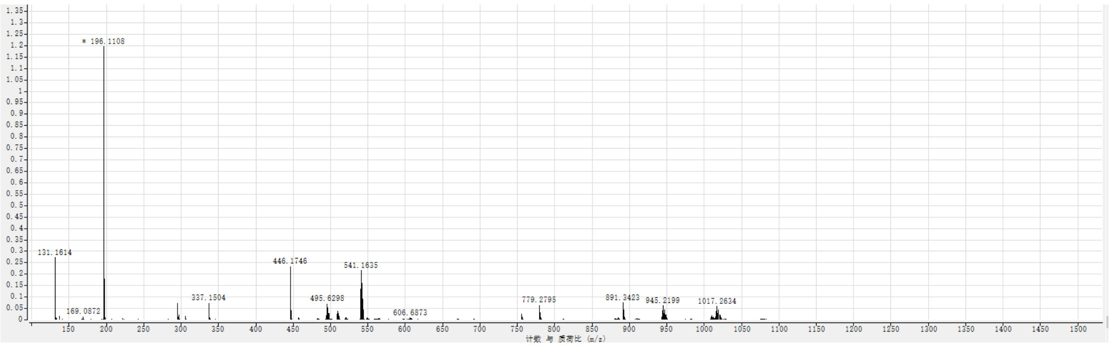

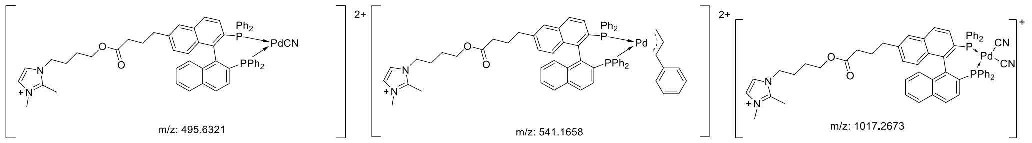

Supplementary Figure 6. **HRMS analysis of reaction mixture with charge-tagged ligand L9.**

2.4 Electrochemical analysis experiment

The electrochemical analyses were recorded using a glassy carbon working electrode, a Pt wire auxiliary electrode and a saturated calomel (SCE) reference electrode. The voltammograms were recorded at room temperature in MeCN at a substrate concentration of 0.04 mol/L and with 0.1 M Et_4NCl as supporting electrolyte. The scan rate was 50 mV/s.

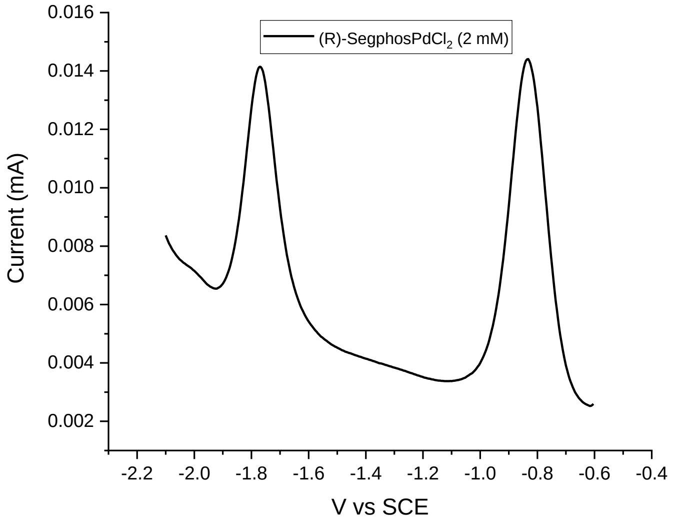

Supplementary Figure 7. **Square-wave voltammetry of (R)-SegphosPdCl$_2$ (2 mM) with 0.1 M of Et4NCl. The scan rate is 50 mV/s**

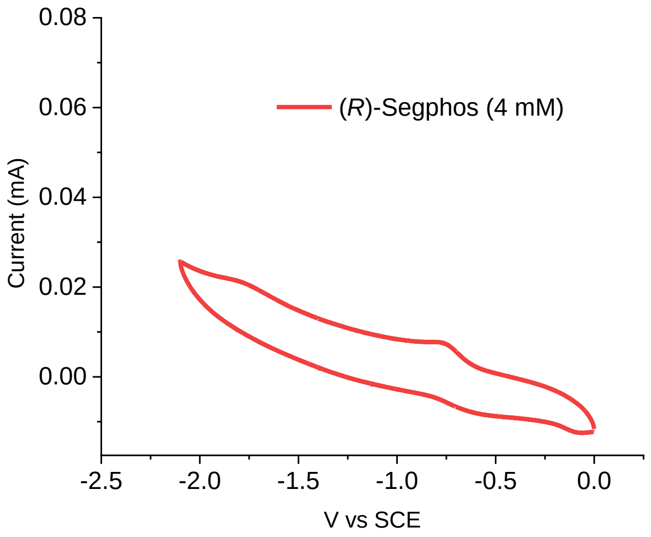

Supplementary Figure 8. **Cyclic voltammograms of (*R*)-L1 (4 mM) in MeCN (5 mL) with 0.1 M of Et_4NCl. The scan rate is 50 mV/s.**

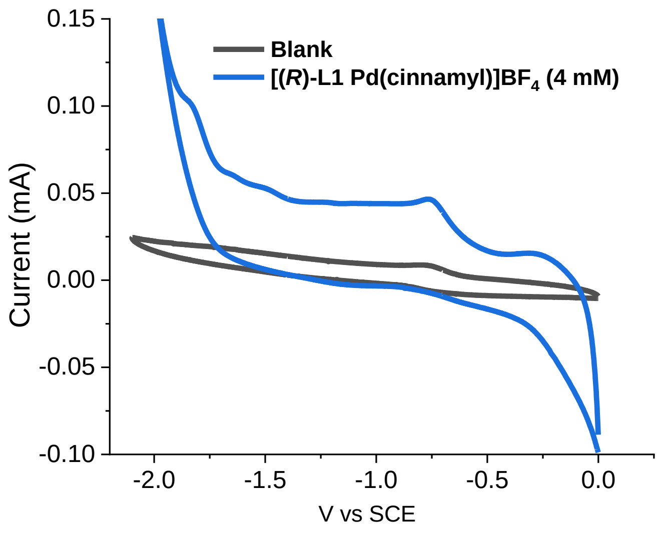

Supplementary Figure 9. **Cyclic voltammograms of [(*R*)-L1 Pd(cinnamyl)]BF_4 (4 mM) in MeCN (5 mL) with 0.1 M of Et_4NCl. The scan rate is 50 mV/s**.

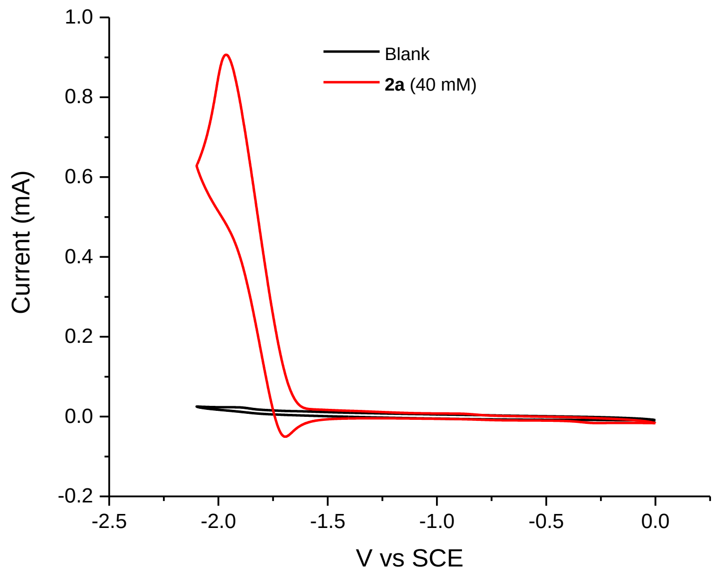

Supplementary Figure 10**. Cyclic voltammograms of 2a (40 mM) in MeCN (5 mL) with 0.1 M of Et_4NCl. The scan rate is 50 mV/s.**

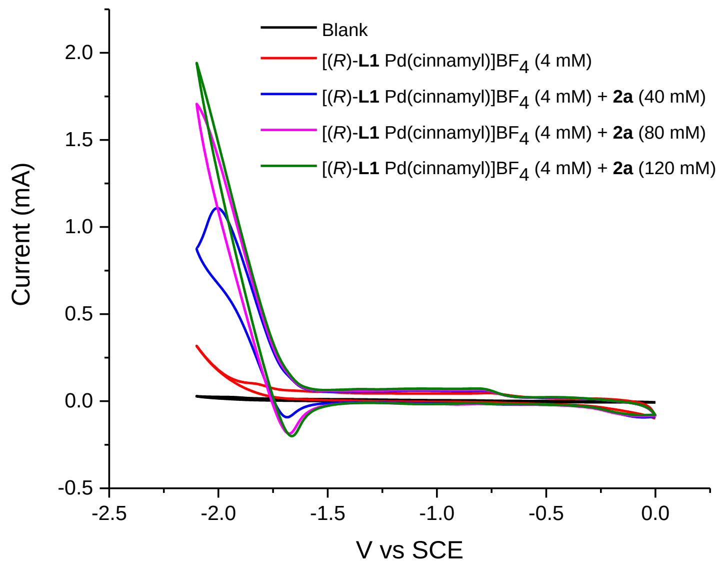

Supplementary Figure 11. **Cyclic voltammograms of [(*R*)-L1 Pd(cinnamyl)]BF_4 (4 mM) and various concentrations of 2a in MeCN (5 mL) with 0.1 M of Et_4NCl. The scan rate is 50 mV/s.**

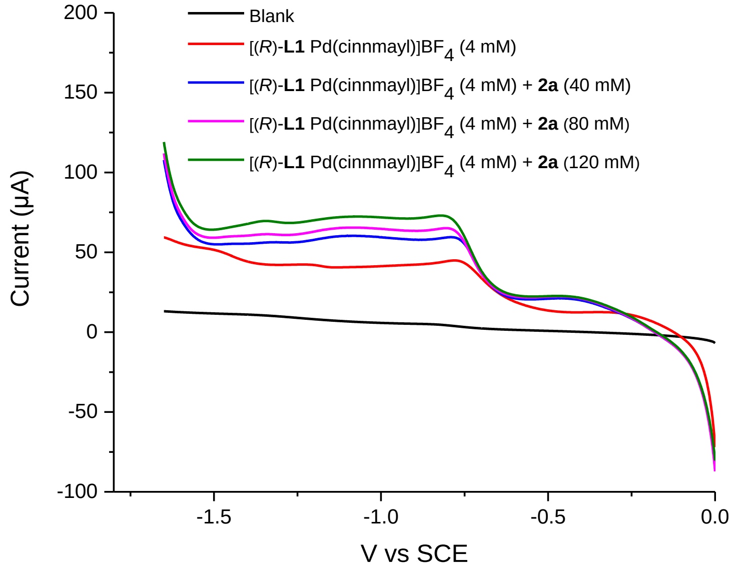

Supplementary Figure 12**. Detail View Profile (-1.75 – 0 V). Cyclic voltammograms of [(R)-L1 Pd(cinnamyl)]BF_4 (4 mM) and various concentrations of 2a in MeCN (5 mL) with 0.1 M of Et_4NCl. The scan rate is 50 mV/s.**

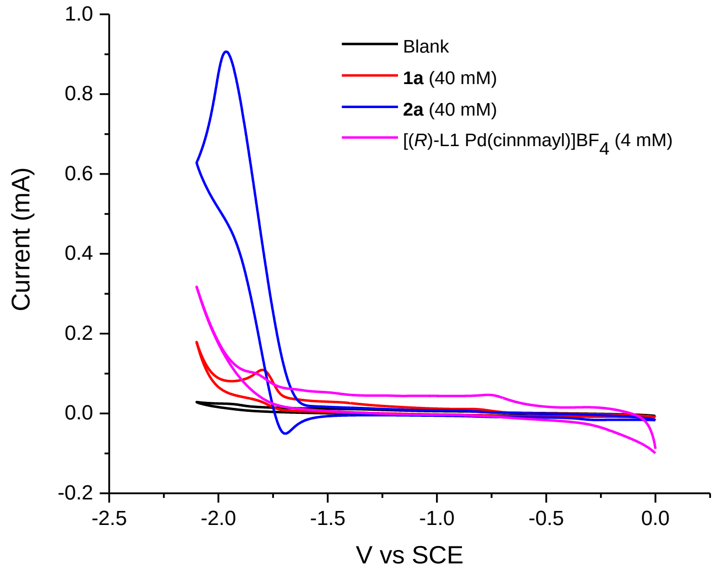

Supplementary Figure 13. **Cyclic voltammograms of several reaction mixtures in MeCN (5 mL) with 0.1 M of Et_4NCl. The scan rate is 50 mV/s.**

2.5 NMR titration

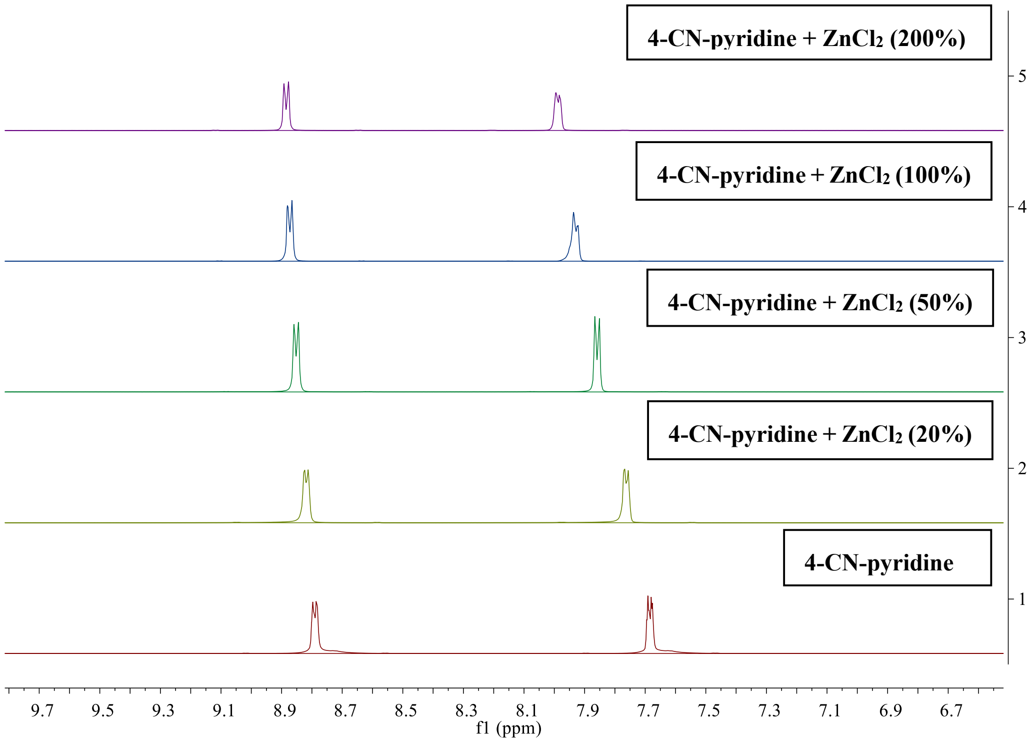

Supplementary Figure 14. **NMR titration of 2a with various amount of $ZnCl_2$ in CD_3CN/CD_3OD (5:1, v/v).**

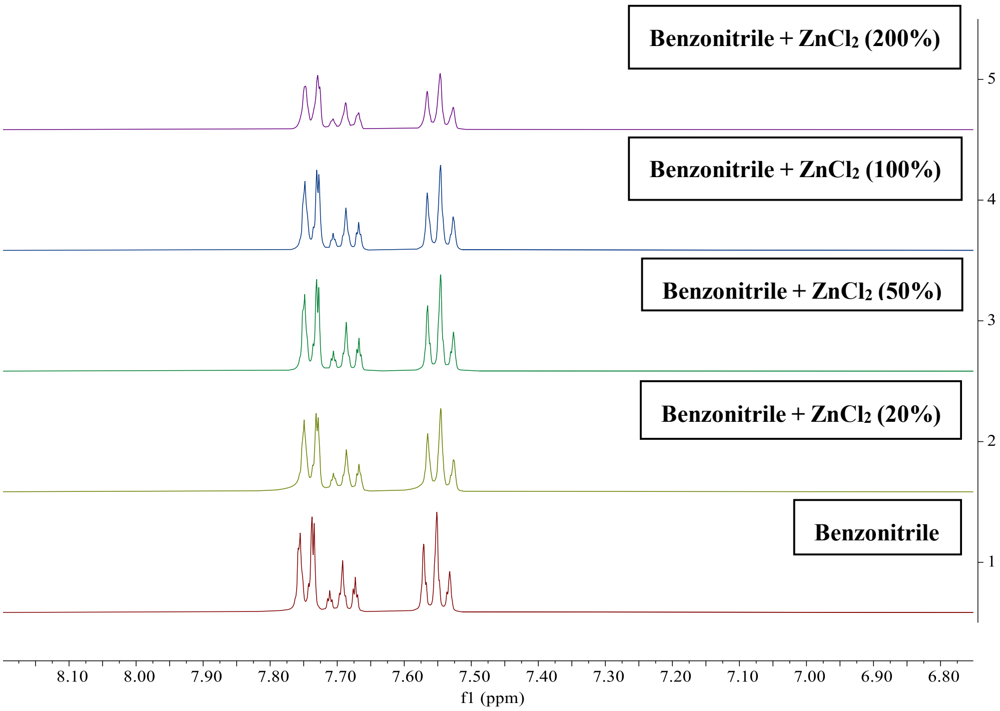

Supplementary Figure 15. **NMR titration of Benzonitrile with various amount of $ZnCl_2$ in CD_3CN/CD_3OD (5:1, v/v).**

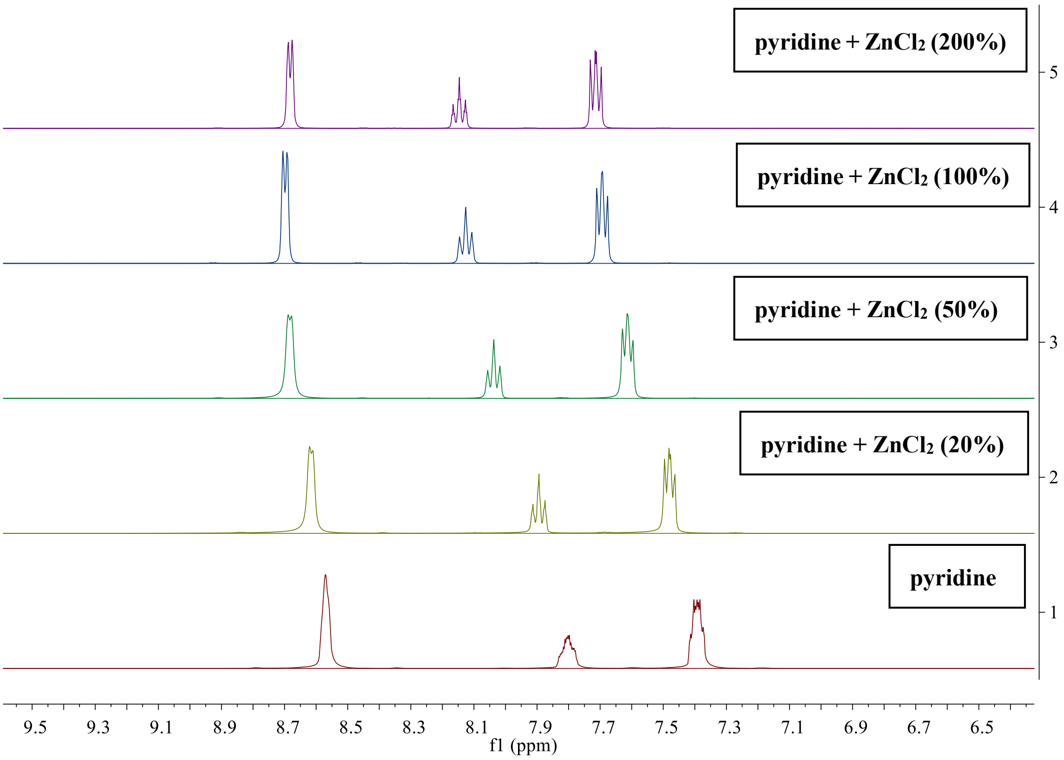

Supplementary Figure 16. **NMR titration of pyridine with various amount of $ZnCl_2$ in CD_3CN/CD_3OD (5:1, v/v).**

2.6 Control experiments

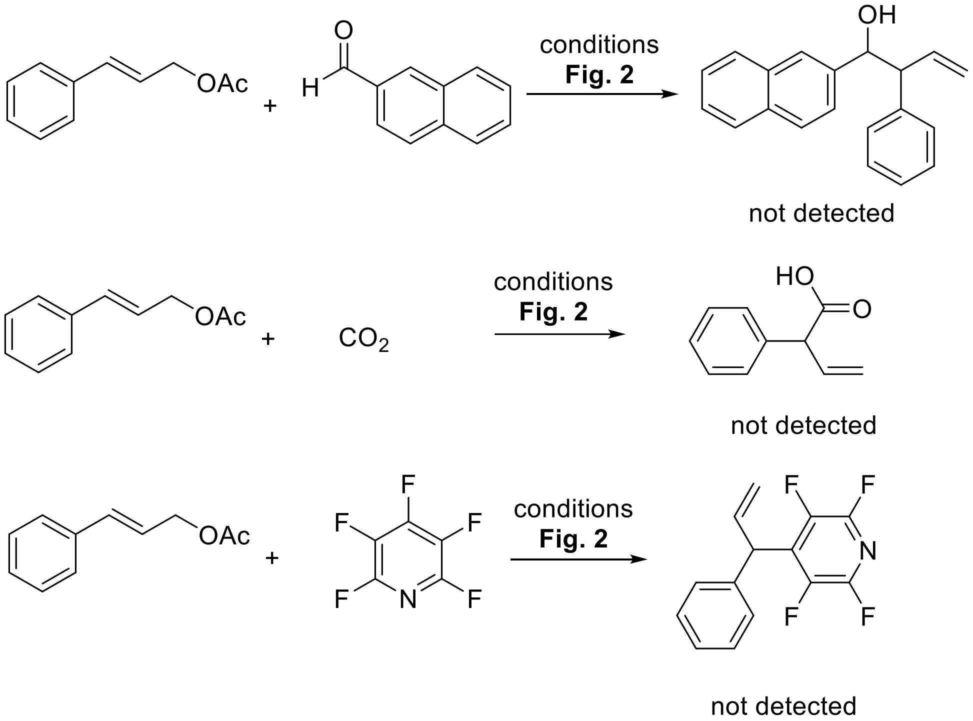

Supplementary Figure 17. **Control experiment with electrophile other than 2a**.

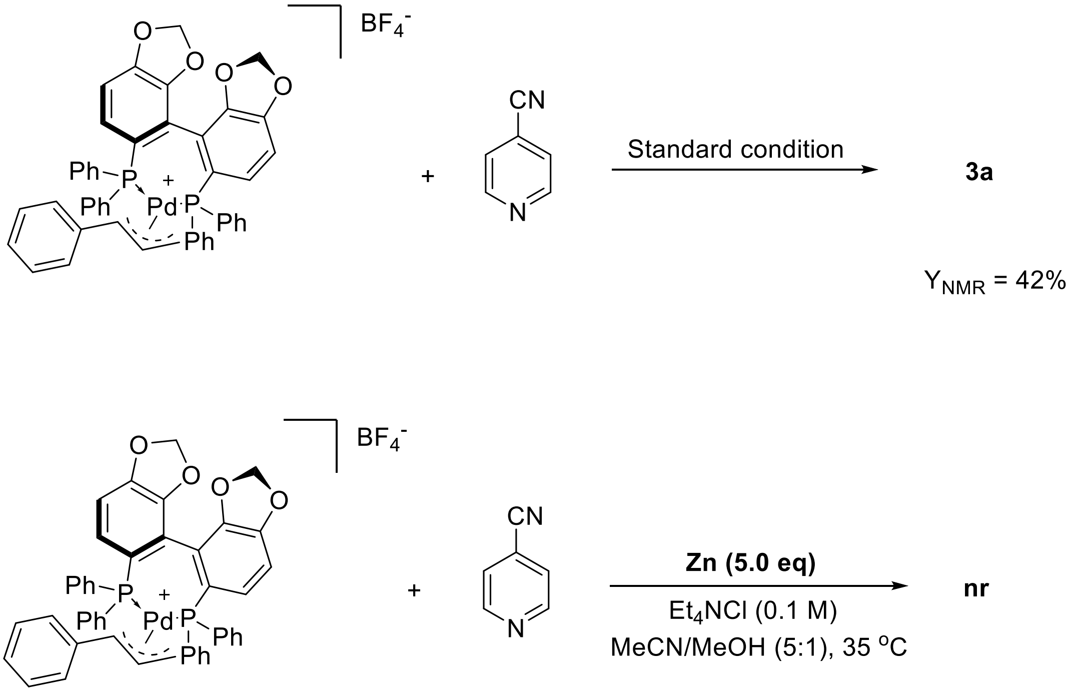

Supplementary Figure 18. **Control experiment of electroreduction and zinc reduction.**

Supplementary Table 13. **Controlled potential electrolysis experiment.**

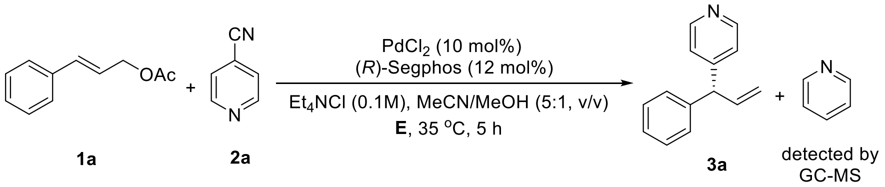

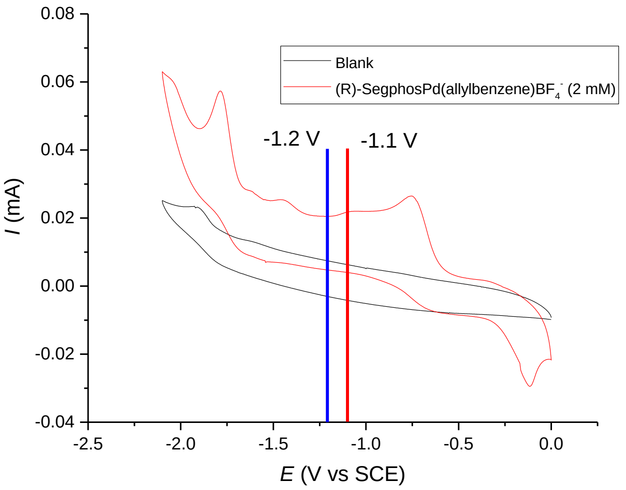

Entry	E	Yield
1	-1.0V	n.r.
2	-1.1V	n.r.
3	-1.2V	58%
4	-1.4V	62%
5	-1.2 V w/o Pd/L	n.r.

a reaction condition: **1a** (0.2 mmol), **2a** (0.6 mmol), $PdCl_2$ (10 mol %), (*R*)-Segphos (12 mol %), MeCN/MeOH (5:1, v/v), Et_4NCl (0.1 M), graphite felt as cathode, Zn as anode, and a saturated calomel (SCE) reference electrode, in an undivided cell and controlled potential electrolysis for 5 h at 35 °C. **b** Yields were determined by ^{1}H NMR using CH_2Br_2 as an internal standard.

In this reaction under standard conditions with constant cathode potential of -1.2 V vs SCE, pyridine was detected as the side product. If $PdCl_2$ and Segphos were absent, neither product **3a** nor pyridine was detected, and **1a** and **2a** were recovered almost quantitatively.

2.7 Monitoring the reaction of (E)- or (Z)-1ac to 3ac

Synthesis of (*Z*)-1ac

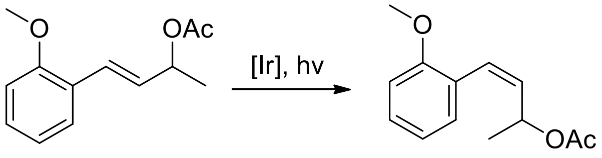

A dry tube with rubber septum was charged with $Ir(ppy)_3$ (3.9 mg, 0.006 mmol), (E)-4-(2-methoxyphenyl)but-3-en-2-yl acetate (660.8 mg, 3.0 mmol), DIPEA (52.2 μL, 0.3 mmol), and CH_3CN (15 mL) were added under Ar. The tube was placed in a light bath (Blue Light) and maintained at 30 °C. After the reaction completed (about 48 h, monitored by GC-MS), the mixture was directed removal of the solvent under reduced pressure. The residue was purified by chromatography on silica gel to afford the desire (Z)-4-(2-methoxyphenyl)but-3-en-2-yl acetate (*Z*)-**1ac** as colorless oil (594.7 mg, E/Z = 1:16, 90%).

Electroreduction coupling of (E)-/(Z)- allyl acetate with 4-cyanopyridine

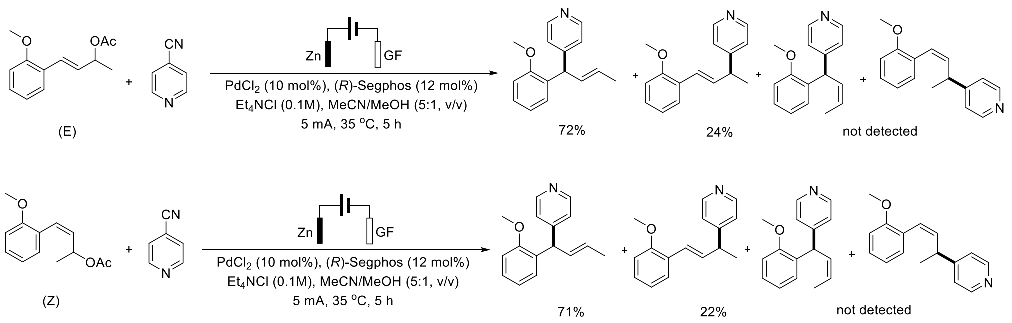

Supplementary Figure 19**. Electroreduction coupling of (E)-/(Z)- allyl acetate with 4-cyanopyridine**

Specific experimental procedure:

A 10 mL three-necked flask was charged with $PdCl_2$ (0.02 mmol, 10 mol%), (*R*)-Segphos (0.024 mmol, 12 mol%) and a magnetic stir bar. The flask was evacuated and backfilled with argon for three times, and anhydrous MeCN (2 mL) was added via syringe. The mixture was stirred under room temperature for 30 min. Then the substrate (E)-**1ac** or (Z)**-1ac** (0.2 mmol, 44.1 mg, 1.0 equiv), **2a** (0.6 mmol, 62.5 mg, 3.0 equiv), Et_4NCl (0.6 mmol, 99.4 mg, 3.0 equiv), anhydrous MeCN (3 mL) and MeOH (1 mL) was added. The graphite felt (2 cm x 1 cm x 0.5 cm) as cathode, Zn (1.5 cm x 1 cm x 0.2 cm) as anode. The Zn anode attached to a platinum wire and graphite felt cathode attached to a titanium wire. The mixture was stirred under 35 ºC and constant current electrolysis (5 mA). After 5 h, the mixture was extracted with ethyl acetate. The organic layers were washed with brine, dried over Na_2SO_4, filtered and concentrated. The residue was purified by chromatography on silica gel to afford the desire product as a pale yellow oil. From the analysis of the isolated product, it can be seen that both (Z)-substrates and (E)-substrates yielded (E)-products under our standard conditions. These results show the intermediate of reaction from E or Z substrate undergoes fast equilibrium as that reported previously. [8,9]

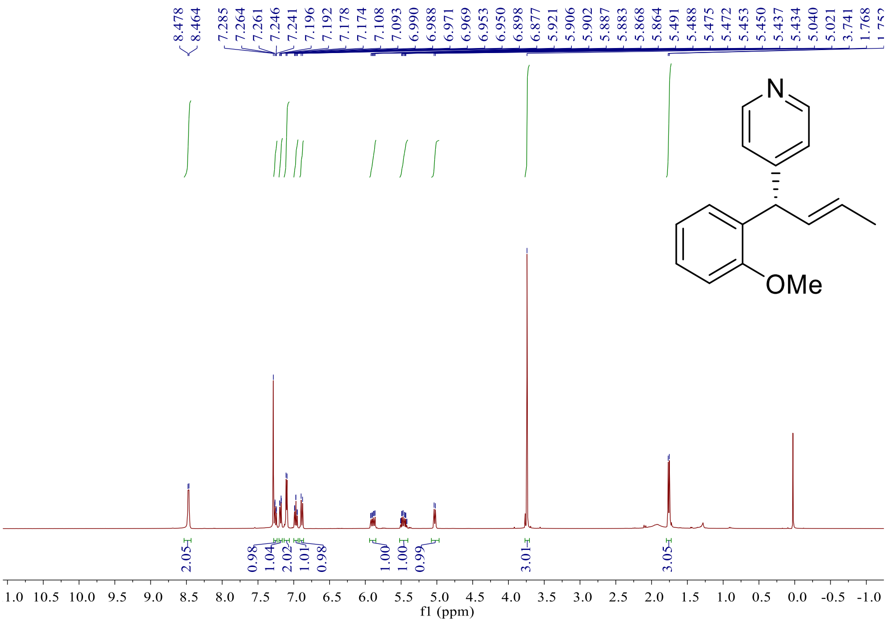

^{1}H NMR Spectrum of (*E*)-**3ac**

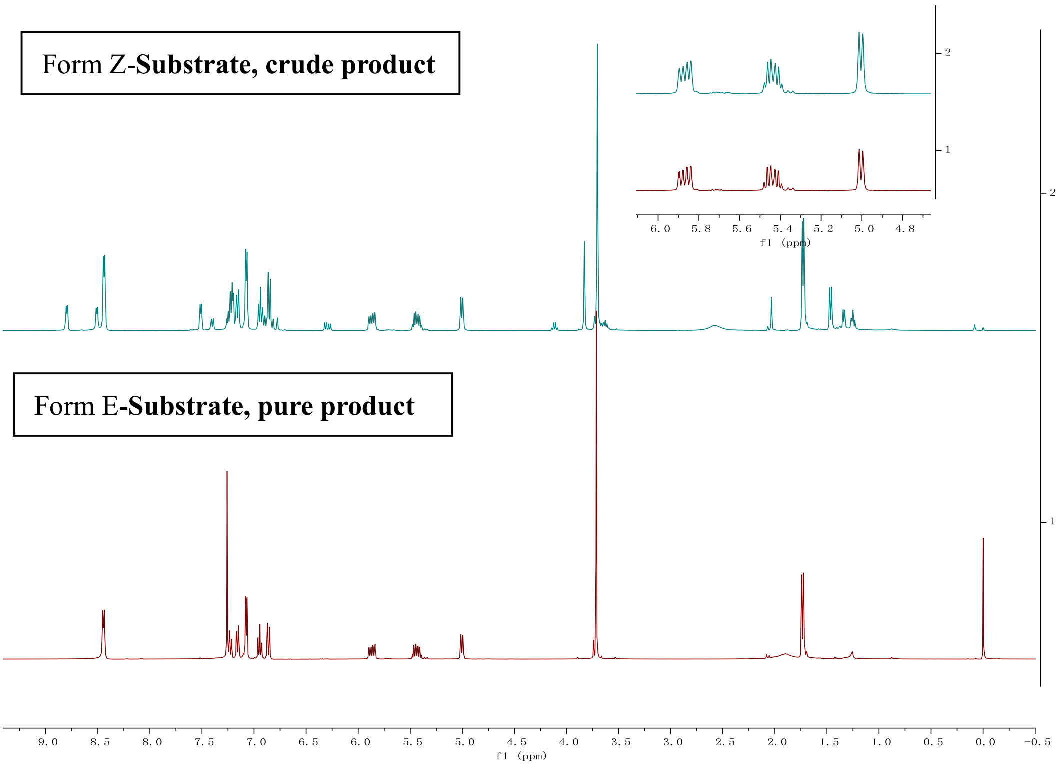

Supplementary Figure 20. **^{1}H NMR Spectrum of (E)-3ac from (E)- and (Z)-1ac**

2.8 Monitoring reaction of (E)- or (Z)-1ac forming allyl Pd-L1 complex

As that reported, [10] in BINAP Pd with unsymmetrical allyl (*syn*-allyl moiety in ring structure), two phosphine atoms show two sets of AX peaks respectively. Two reactions using (E)-**1ac** and

(Z)-**1ac** were carried out respectively, and both reactions gave the same product (E)-**3ac**. The reactions were tracked with ^{31}P NMR. At 10th, 30th, and 60th min, the reaction mixture from (*E*)-**1ac** and (*Z*)-**1ac** gave two sets of AX peaks. Meanwhile, some weak ^{31}P NMR was observed. These results suggest there is one predominant palladium species with one allyl configuration.

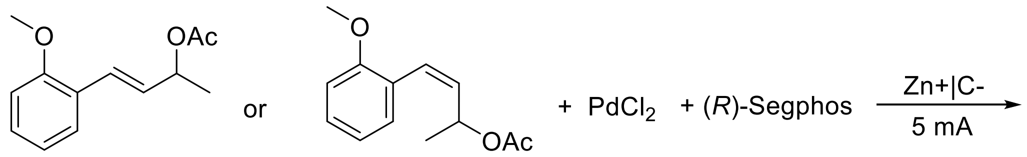

(*E*)-**1ac** (*Z*)-**1ac**

A 10 mL three-necked flask was charged with $PdCl_2$ (0.1 mmol, 17.7 mg, 1.0 equiv), (*R*)-Segphos (0.1 mmol, 61.1 mg, 1.0 equiv). The flask was evacuated and backfilled with argon for three times, and anhydrous MeCN (5 mL) was added via syringe. The mixture was stirred under room temperature for 1 h. Then the substrate (*E*)-**1ac** or (*Z*)-**1ac** (0.12 mmol, 26.4 mg, 1.2 equiv), Et_4NCl (0.6 mmol, 99.4 mg, 3.0 equiv) and MeOH (1 mL) was added. The flask was equipped with a rubber stopper, graphite felt (2 cm x 1 cm x 0.5 cm) as cathode, Zn (1.5 cm x 1 cm x 0.2 cm) as anode. The mixture was stirred under 35 °C and constant current electrolysis (5 mA). During the reaction, ^{31}P NMR was used to monitor the changes in the reaction process at 10 min, 30 min and 1 h, respectively. For each sampling, use a syringe to take 0.3 ml of the reaction system solution into an NMR tube, add 0.15 ml of deuterated acetonitrile, and scan 1024 times with NMR to obtain a corresponding ^{31}P NMR Spectrum. The corresponding data is shown below:

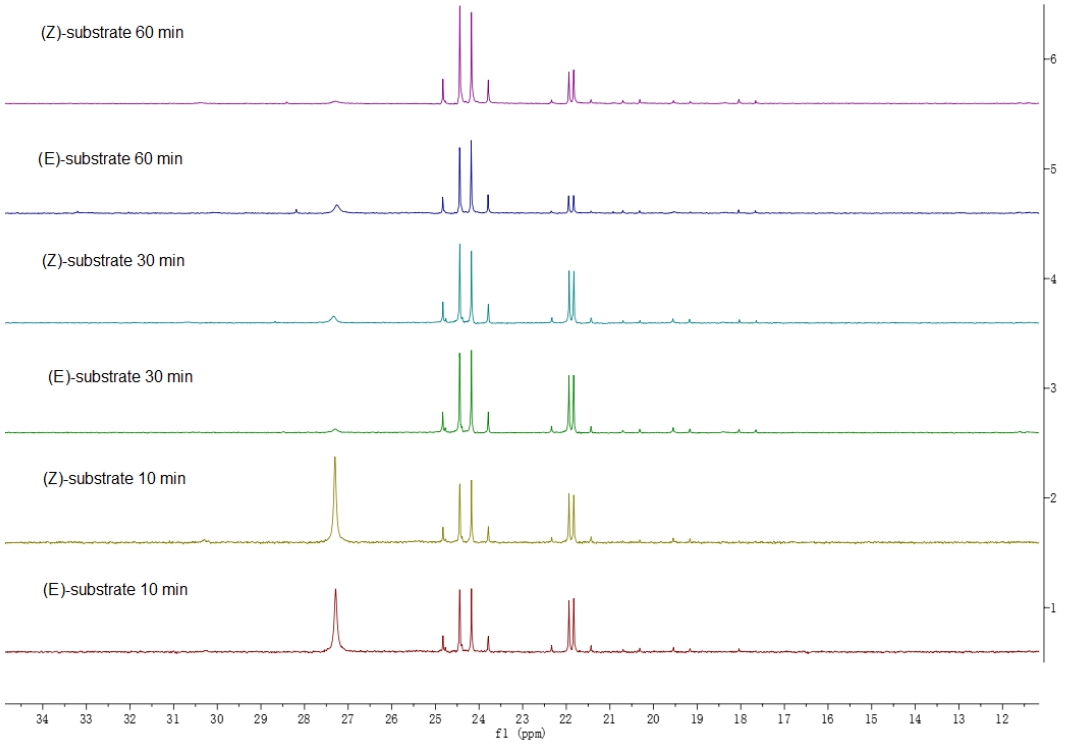

Supplementary Figure 21. ^{31}P NMR spectrum of π-allylpalladium complexes in CD_3CN

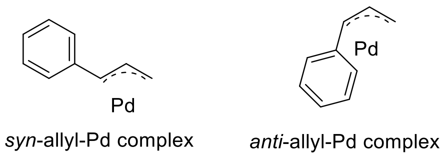

2.9 The change of enantioselectivity in the reaction

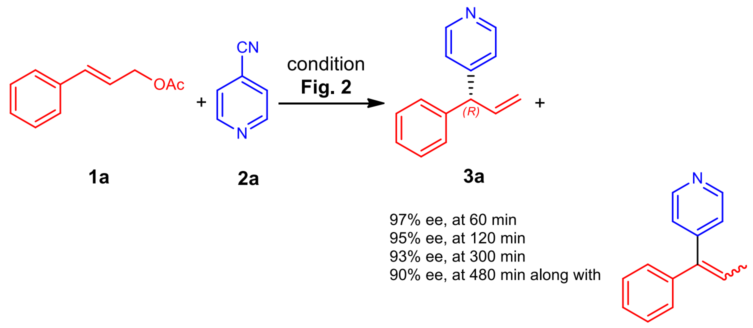

Supplementary Figure 22. Tracking the enantioselectiviity of product **3a**

The enantioselectivity of **3a** was tracked in model reaction. The initial ee of product **3a** is 97% at 60 min. Then, the ee dropped to 93% when the full conversion of **1a** achieved at 300 min. If the reaction was conducted with prolonged time, the ee dropped further to 90% along with the occurrence of by-product with alkene shifted to conjugation. This is possible due to the acidity of C-H of chiral center or the change of catalyst during the reaction, especially when the strong coordinative cyanide generates.

Analysis of Stereochemistry:

Enantiomeric excess: 97%, determined by HPLC (Daicel Chiralpak ASH, hexane/isopropanol = 98.5/1.5, flow rate 1.0 mL/min, T = 25 °C, 220nm): t_R= 8.985 min (minor), t_R= 9.930 min (major).

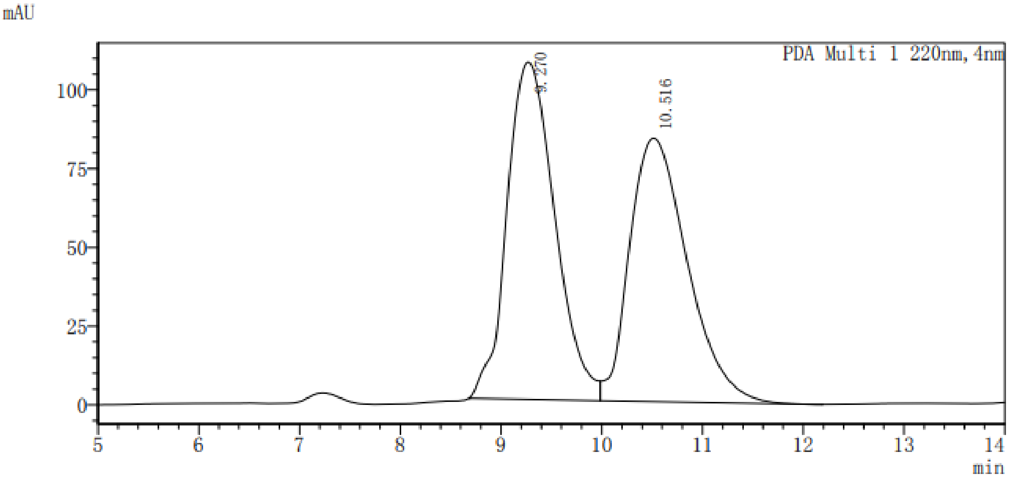

Peak	Ret Time[min]	Height [mAU]	Area[mAU*s]	Area %

1	9.270	107085	3540606	51.484
2	10.516	83618	3336523	48.516
Total		190704	6877129	100.000

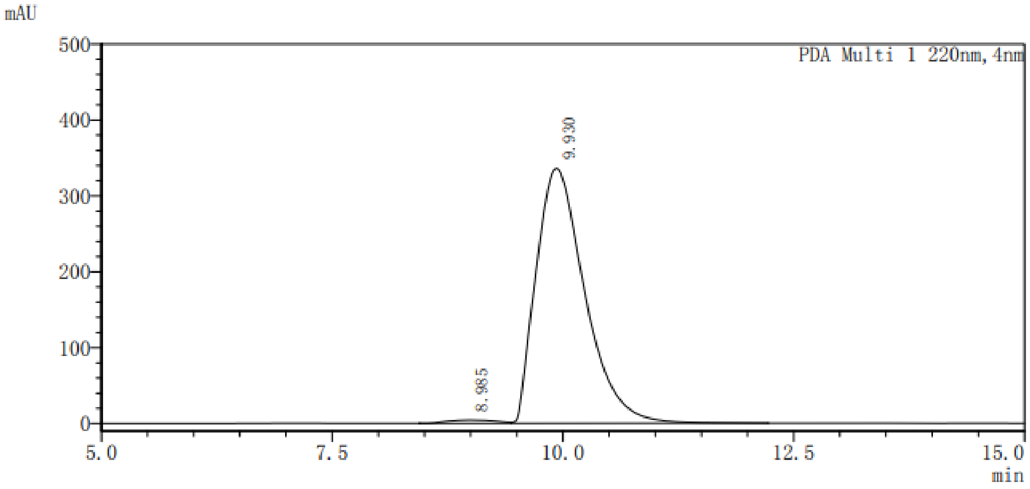

Peak	Ret Time[min]	Height [mAU]	Area[mAU*s]	Area %
1	8.985	4659	166631	1.333
2	9.930	336084	12329263	98.667
Total		340744	12495894	100.000

Supplementary Figure 23. **HPLC chromatography for 3a analysis of reaction mixture at 60th minute.**

2.10 DFT computation study of reaction pathway

Computational Details:

Density functional theory (DFT) calculations were carried out using Gaussian 16 programs C01 [10] on Cluster of High Performance Computer Center, Nanjing University throughout this manuscript. Geometric optimizations of the reactants, transition states, and products were performed using B3LYP hybrid functional[11] with Grimme's dispersion correction of D3 version (Becke-Johnson damping)[12]. The standard 6-31G* basis set[13,14] for all atoms was used. A effective core potential associated with the SDD basis set[15] was used on Pd. Harmonic vibration frequency calculations were performed for all stationary points to confirm them as a local minima (zero imaginary frequencies) or a transition state (one imaginary frequency). The thermochemical corrections for the Gibbs free energies were derived at 298.15 K. The intrinsic reaction coordinate (IRC) scheme[16,17] was applied for the calculations of the reaction coordinates to confirm whether or not the transition states were directly connected to the reactants and products. The single point energy (SP) calculations were performed on the optimized geometries with the larger def2-TZVPP basis set[18-20]. Approximate solvent effects were taken into consideration based on the SMD continuum solvation model[21] in all SP calculations. The Gibbs free energy of a solute in solution is given as

$$G_{soln} = E_{SP} + G_{corr}^{298.15K} + \Delta G_{1atm\rightarrow 1M}^{298.15K}$$

Summary of energy of species in reaction pathway

(All Values Are in Hartrees)

Species	E_sp	G_corr	1atm 1M	G
INT_C (*E*)	-3043.923943	0.718305	0.003019	-3043.202619
INT_C(*Z*)	-3043.912505	0.719064	0.003019	-3043.190422
INT-D	-3044.0236	0.710813	0.003019	-3043.309768
INT-E	-5624.380964	0.793854	0.003019	-5623.584091
INT-F	-5624.478448	0.788221	0.003019	-5623.687208
INT-G	-3291.796639	0.780824	0.003019	-3291.012796
TS-R	-3291.758353	0.780118	0.003019	-3290.975216
TS-R-L	-3291.754645	0.782976	0.003019	-3290.96865
TS-S	-3291.761288	0.780126	0.003019	-3290.978143
E_branch_product	-714.3523517	0.270118	0.003019	-714.0792147

Atomic spin population analysis

The summary of atomic spin populations at the red box positions of intermediate **E** (Fig. 7 in Main Article) is 0. The summary of atomic spin populations at the red box positions of intermediate **F** (Fig. 7 in Main Article) add up to about 1. These results imply the single electron delocalization on metal center and allyl ligand.

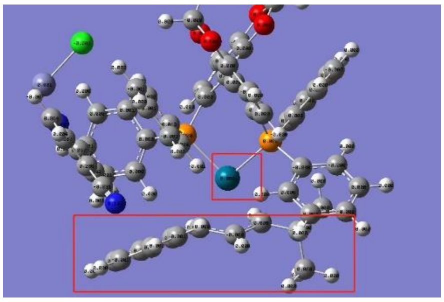

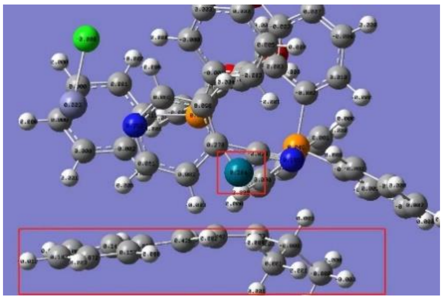

INT-E **INT-F**

Supplementary Figure 24. **Comparison of atomic spin population of intermediate E and F.**

atom number	atom	**INT-D**	**INT-E**	**INT-F**
1	Pd	0.254182	0.000408	0.204138
74	C	0.461575	0.000134	0.082978
75	C	-0.121168	-0.000037	-0.041811
76	C	0.175457	0.000171	0.42619
77	H	-0.024765	-0.000005	-0.003191
78	H	0.000419	-0.000001	-0.002007
79	H	-0.002813	-0.000022	-0.02425
80	C	-0.035245	0.000468	-0.074972
81	C	0.056808	-0.001125	0.156716
82	C	0.042551	0.000316	0.140763
83	C	-0.026308	0.001467	-0.07153
84	H	-0.002602	-0.00005	-0.008309
85	C	-0.023231	0.000514	-0.068471
86	H	-0.001889	-0.000053	-0.005736
87	C	0.062313	-0.000509	0.179818
88	H	0.001013	-0.000377	0.002952
89	H	0.000817	0.000182	0.00246
90	H	-0.004065	0.000209	-0.011657
91	C	-0.024447	-0.000015	-0.003375
92	H	0.017069	0	0.005642
93	C	0.012183	0.000008	0.001342
94	H	0.000297	0	-0.000141
95	H	0.000431	0	0.000897

	96	H	0.004304	0.000002	0.000649
	97	C	0.001785	0.000002	-0.000082
	98	H	-0.000204	0	0.000459
	99	H	-0.00054	0	0.000001
	100	H	-0.000135	0	-0.000104
Summary of atomic spin population			0.823792	0.001687	0.889369

2.11 Explore another persistent radical precursor

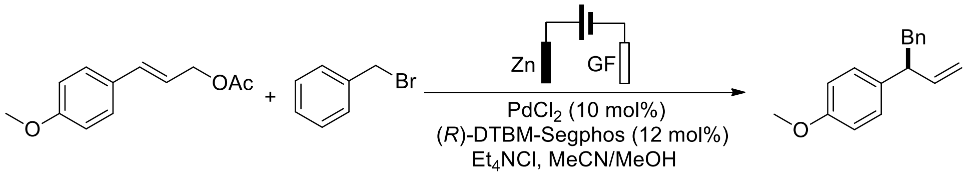

7a
32%, ee = 89%

Following the general procedure, the product **7a** was isolated by chromatography on silica gel (PE/EA = 100/1, eluent) as a colorless oil (32%, 15.2 mg); ^{1}H NMR (400 MHz, Chloroform-*d*) δ 7.28 (d, *J* = 7.9 Hz, 1H), 7.21 (dd, *J* = 7.9, 6.4 Hz, 2H), 7.18 – 7.12 (m, 1H), 7.08 – 7.04 (m, 3H), 6.85 – 6.80 (m, 2H), 6.01 (ddd, *J* = 17.2, 10.3, 7.2 Hz, 1H), 5.05 – 4.88 (m, 2H), 3.78 (s, 3H), 3.53 (q, *J* = 7.5 Hz, 1H), 3.06 – 2.93 (m, 2H).

Analysis of Stereochemistry:

Enantiomeric excess: 89%, determined by HPLC (Agela Technologies Venusil CJ, hexane/isopropanol = 99/1, flow rate 1.0 mL/min, T = 25 °C, 220nm): t_R= 14.00 min (minor), t_R= 15.09 min (major).

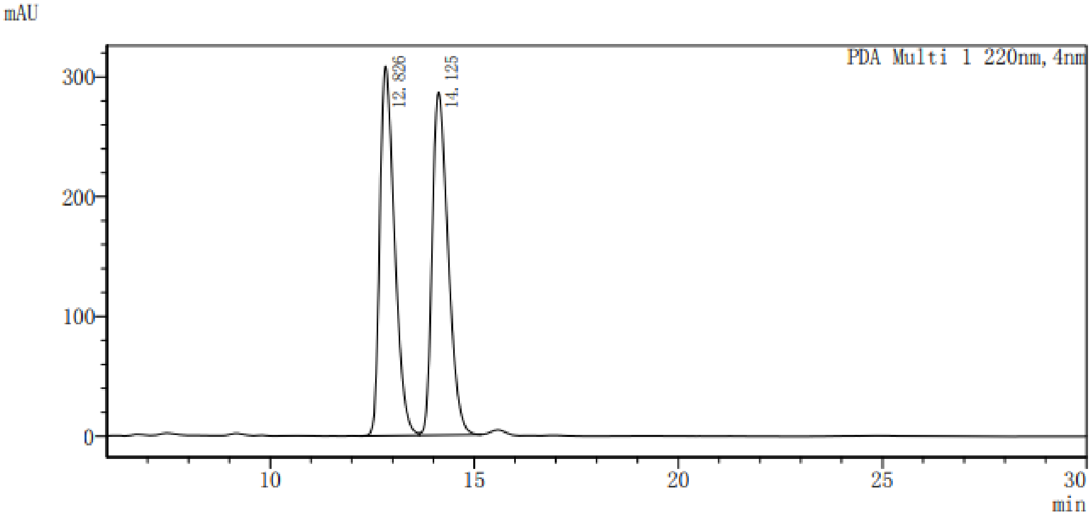

Peak	Ret Time[min]	Area[mAU*s]	Height [mAU]	Area %

1	12. 826	308334	7753264	49. 998
2	14. 125	286022	7753894	50. 002
Total		594356	15507158	100. 000

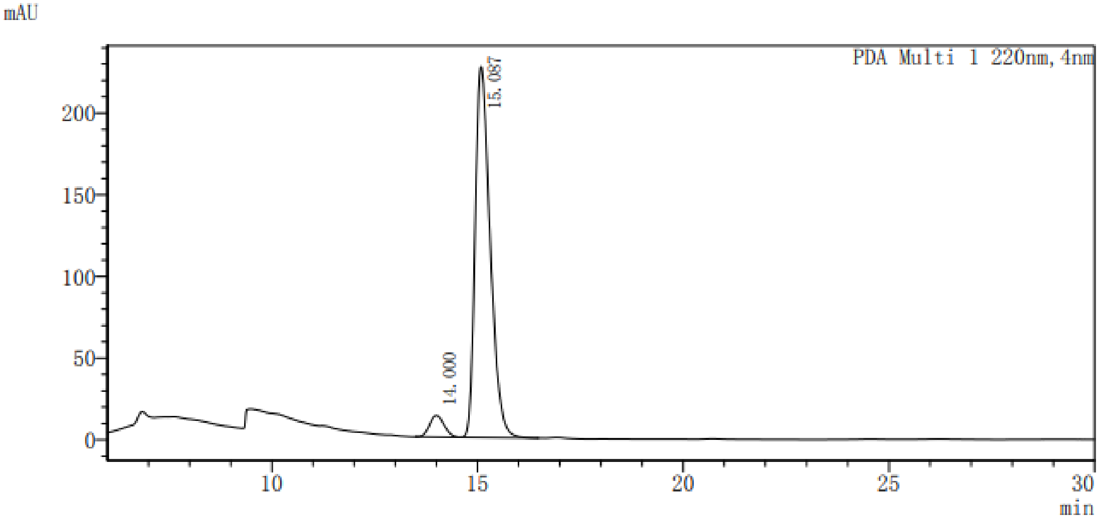

Peak	Ret Time[min]	Area[mAU*s]	Height [mAU]	Area %
1	14. 000	13202	325525	5. 334
2	15. 087	226985	5777158	94. 666
Total		240187	6102682	100. 000

HPLC chromatography for **7a**

3. Supplementary Notes

3.1 Product characterization

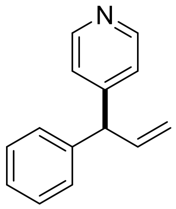

(R)-4-(1-phenylallyl)pyridine **(3a)**:

Following the general procedure, the product **3a** was isolated by chromatography on silica gel (PE/EA = 10/1 ~ 5/1, eluent) as a pale yellow oil (78%, 30.5 mg); $[\alpha]_D^{25}$ = 30.5 (c 0.8, $CHCl_3$); ^{1}H NMR (400 MHz, $CDCl_3$) δ 8.56 (d, *J* = 3.9 Hz, 2H), 7.33 (t, *J* = 7.4 Hz, 2H), 7.27 (s, 1H), 7.16 (dd, *J* = 5.8, 2.9 Hz, 4H), 6.23 (ddd, *J* = 17.3, 10.2, 7.3 Hz, 1H), 5.30 (d, *J* = 10.2 Hz, 1H), 5.03 (d, *J* = 17.1 Hz, 1H), 4.71 (d, *J* = 7.2 Hz, 1H). ^{13}C NMR (100 MHz, $CDCl_3$) δ 153.24, 149.62, 141.21, 138.62, 128.75, 128.53, 127.04, 124.20, 117.78, 54.36. **HRMS (ESI)** m/z: $[M+Na]^+$ Calcd for $C_{14}H_{13}NNa$ 218.0946; Found 218.0944;

Analysis of Stereochemistry:

Enantiomeric excess: 93%, determined by HPLC (Daicel Chiralpak ASH, hexane/isopropanol = 98.5/1.5, flow rate 1.0 mL/min, T = 25 °C, 220nm): t_R= 16.58 min (minor), t_R= 19.50 min (major).

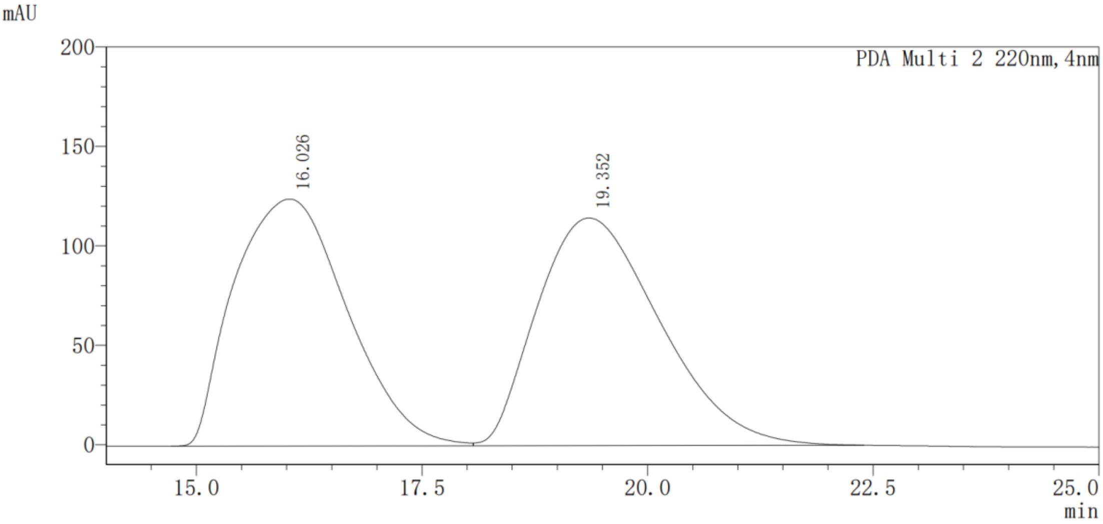

Peak	Ret Time[min]	Area[mAU*s]	Height [mAU]	Area %
1	16.026	10818894	124105	49.997
2	19.352	10820033	114458	50.003
Total		21638927	238563	100.000

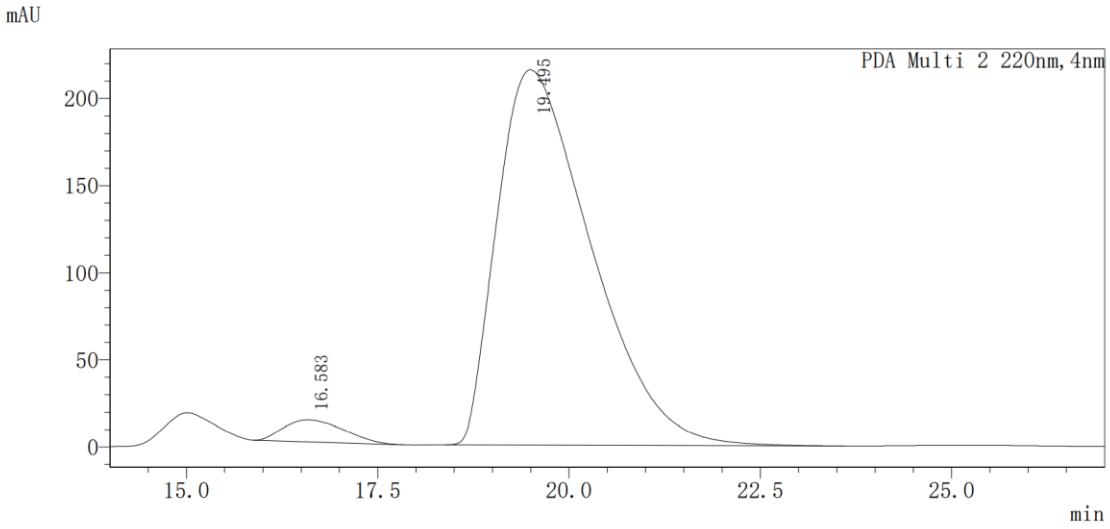

Peak	Ret Time[min]	Area[mAU*s]	Height [mAU]	Area %
1	16.583	695340	12664	3.640
2	19.495	18407674	215388	96.360
Total		19103014	228052	100.000

Supplementary Figure 25. **HPLC chromatography for 3a**

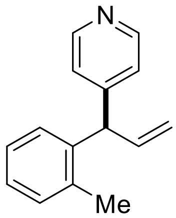

(R)-4-(1-(o-tolyl)allyl)pyridine (**3b**)

Following the general procedure, the product **3b** was isolated by chromatography on silica gel (PE/EA = 10/1 ~ 5/1, eluent) as a pale yellow oil (62%, 26.0 mg); $[\alpha]_D^{25}$ = 28.5 (c 1.2, $CHCl_3$); ^{1}H NMR (400 MHz, $CDCl_3$) δ 8.51 (d, *J* = 3.2 Hz, 2H), 7.19 (s, 3H), 7.09 (d, *J* = 19.2 Hz, 3H), 6.27 - 6.19 (m, 1H), 5.29 (d, *J* = 10.0 Hz, 1H), 4.91 - 4.88 (m, 2H), 2.23 (s, 3H). ^{13}C NMR (100 MHz, $CDCl_3$) δ 151.48, 149.73, 139.48, 138.66, 136.28, 130.68, 128.32, 126.96, 126.17, 124.09, 120.43, 117.50, 50.44, 19.63. **HRMS (ESI)** m/z: $[M+H]^+$ Calcd for $C_{15}H_{16}N$ 210.1283; Found 210.1279.

Analysis of Stereochemistry:

Enantiomeric excess: 93%, determined by HPLC (Agela Technologies Venusil CJ hexane/isopropanol = 99/1, flow rate 0.5 mL/min, T = 25 °C, 220nm): t_R= 34.62 min (minor), t_R= 41.39 min (major).

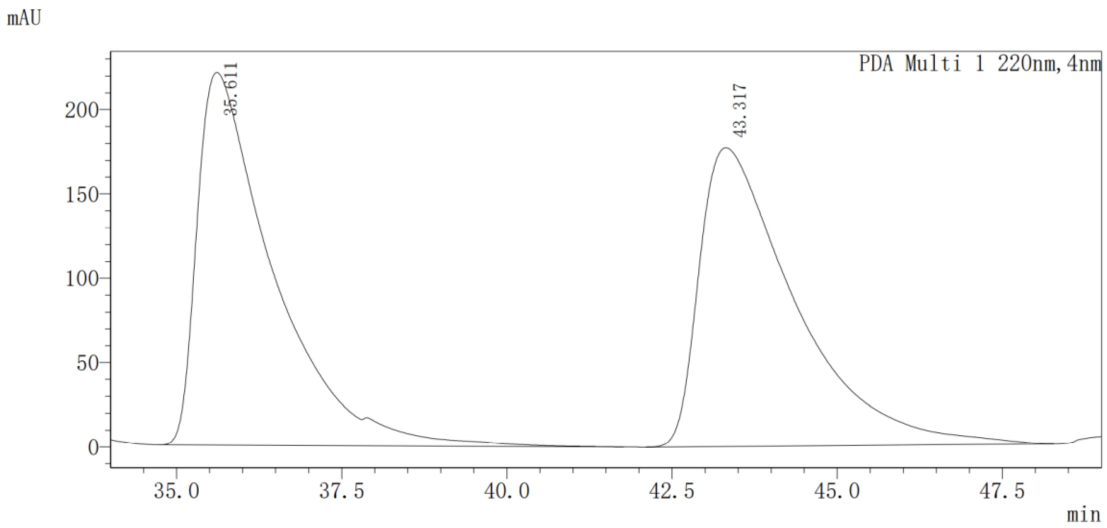

Peak	Ret Time[min]	Area[mAU*s]	Height [mAU]	Area %
1	35. 611	17790887	221109	49. 892
2	43. 317	17867813	177314	50. 108
Total		35658700	398423	100. 000

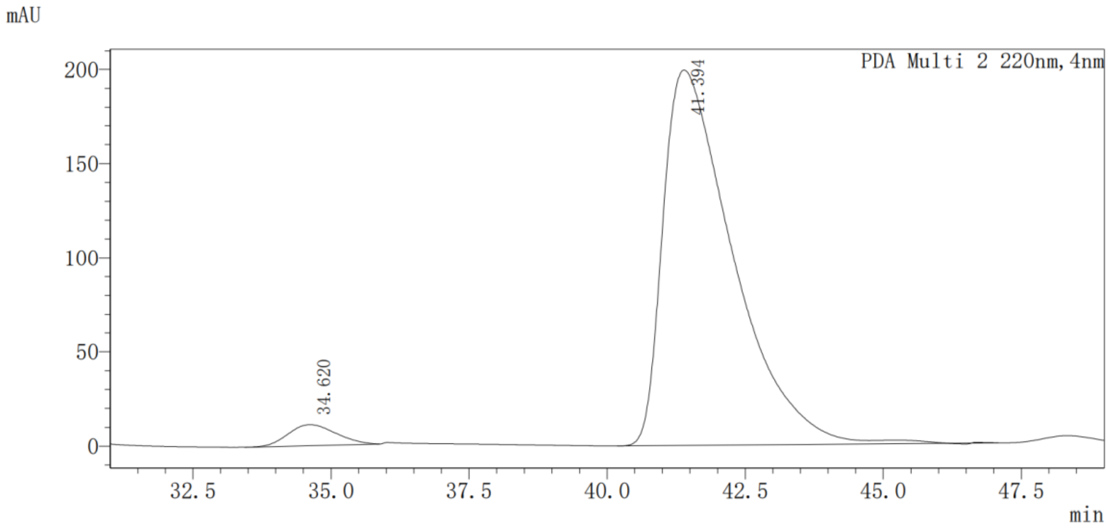

Peak	Ret Time[min]	Area[mAU*s]	Height [mAU]	Area %
1	34. 620	670612	11160	3. 665
2	41. 394	17626692	199198	96. 335
Total		18297304	210359	100. 000

Supplementary Figure 26**. HPLC chromatography for 3b**

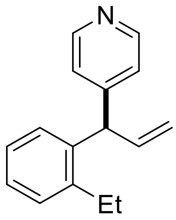

(R)-4-(1-(2-ethylphenyl)allyl)pyridine (3c)

Following the general procedure, the product **3c** was isolated by chromatography on silica gel (PE/EA = 10/1 ~ 5/1, eluent) as a pale yellow oil (76%, 33.9 mg); $[\alpha]_D^{25}$ = 33.6 (c 0.4, $CHCl_3$); ^1H NMR (400 MHz, $CDCl_3$) δ 8.50 (s, 2H), 7.24 - 7.21 (m, 2H), 7.20 - 7.15 (m, 1H), 7.08 (dd, *J* = 6.5, 1.9 Hz, 3H), 6.29 - 6.17 (m, 1H), 5.29 (dt, *J* = 10.2, 1.3 Hz, 1H), 4.96 (d, *J* = 6.6 Hz, 1H), 4.91 (dt, *J* = 17.1, 1.4 Hz, 1H), 2.70 - 2.52 (m, 2H), 1.16 (t, *J* = 7.6 Hz, 3H). ^{13}C NMR (100 MHz, $CDCl_3$) δ 152.22, 149.70, 142.14, 139.21, 138.83, 128.93, 128.77, 127.18, 126.12, 124.04, 117.51, 49.67, 25.64, 15.18. **HRMS (ESI)** m/z: $[M+H]^+$ Calcd for $C_{16}H_{18}N$ 224.1439; Found 224.1433.

Analysis of Stereochemistry:

Enantiomeric excess: 91%, determined by HPLC (Agela Technologies Venusil CJ hexane/isopropanol = 99/1, flow rate 1.0 mL/min, T = 25 °C, 220nm): t_R= 11.20 min (minor), t_R= 13.61 min (major).

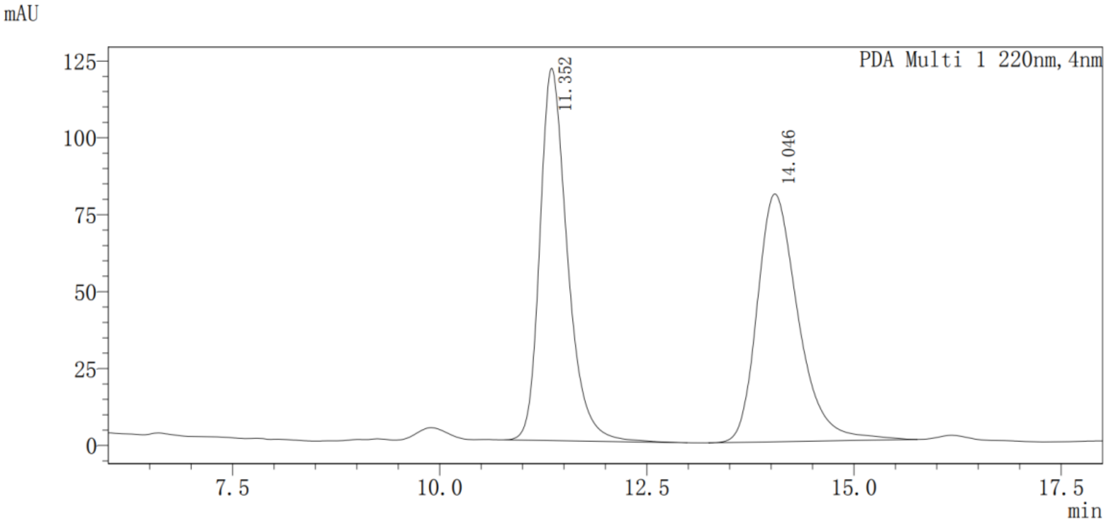

Peak	Ret Time[min]	Area[mAU*s]	Height [mAU]	Area %
1	11.352	2805818	121121	50.303
2	14.046	2772057	80526	49.697
Total		5577876	201647	100.000

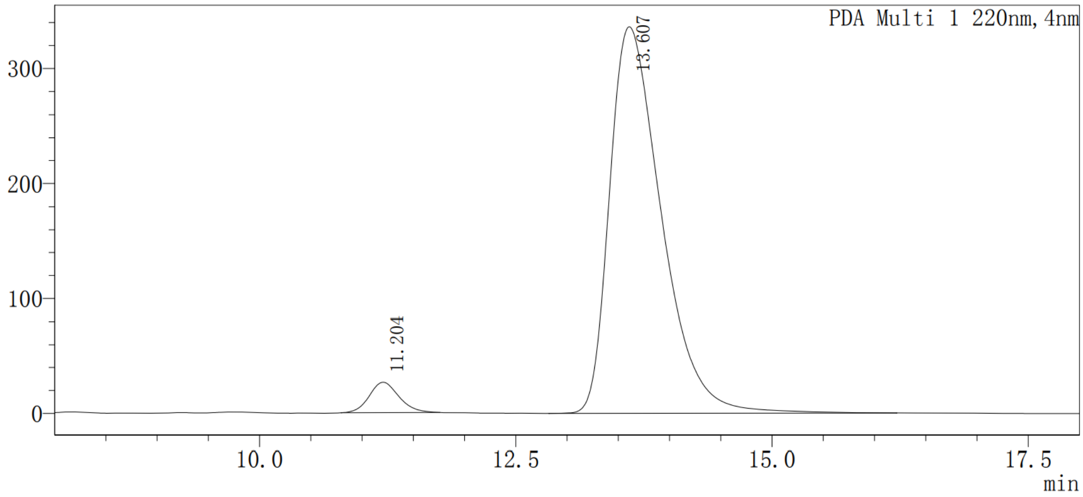

Peak Ret Time[min] Area[mAU*s] Height [mAU] Area %

1	11.204	534400	26466	4.318
2	13.607	11841209	336155	95.682
Total		12375610	362621	100.000

Supplementary Figure 27. **HPLC chromatography for 3c**

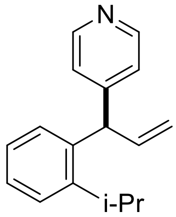

(R)-4-(1-(2-isopropylphenyl)allyl)pyridine **(3d)**

Following the general procedure, the product **3d** was isolated by chromatography on silica gel (PE/EA = 10/1 ~ 5/1, eluent) as a pale yellow oil (58%, 27.5 mg); $[\alpha]_D^{25}$ = 23.6 (c 1.0, $CHCl_3$); ^{1}H NMR (400 MHz, $CDCl_3$) δ 8.49 (d, *J* = 6.0 Hz, 2H), 7.32 (dd, *J* = 7.6, 1.4 Hz, 1H), 7.28 (d, *J* = 1.2 Hz, 1H), 7.18 - 7.14 (m, 1H), 7.08 - 7.06 (m, 3H), 6.29 - 6.21 (m, 1H), 5.30 (dt, *J* = 10.2, 1.3 Hz, 1H), 5.04 (d, *J* = 6.4 Hz, 1H), 4.91 (dt, *J* = 17.2, 1.2 Hz, 1H), 3.11 (dt, *J* = 13.8, 6.8 Hz, 1H), 1.20 (d, *J* = 6.8 Hz, 3H), 1.07 (d, *J* = 6.8 Hz, 3H). ^{13}C NMR (100 MHz, $CDCl_3$) δ 152.49, 149.66, 146.86, 139.34, 137.96, 128.87, 127.36, 125.90, 125.87, 124.02, 117.62, 49.64, 28.71, 23.87, 23.81. **HRMS (ESI)** m/z: $[M+H]^+$ Calcd for $C_{17}H_{20}N$ 238.1596; Found 238.1590.

Analysis of Stereochemistry:

Enantiomeric excess: 91%, determined by HPLC (Daicel Chiralpak OZH hexane/isopropanol = 99/1, flow rate 1.0 mL/min, T = 25 °C, 220nm): t_R = 17.35 min (minor), t_R = 19.31 min (major).

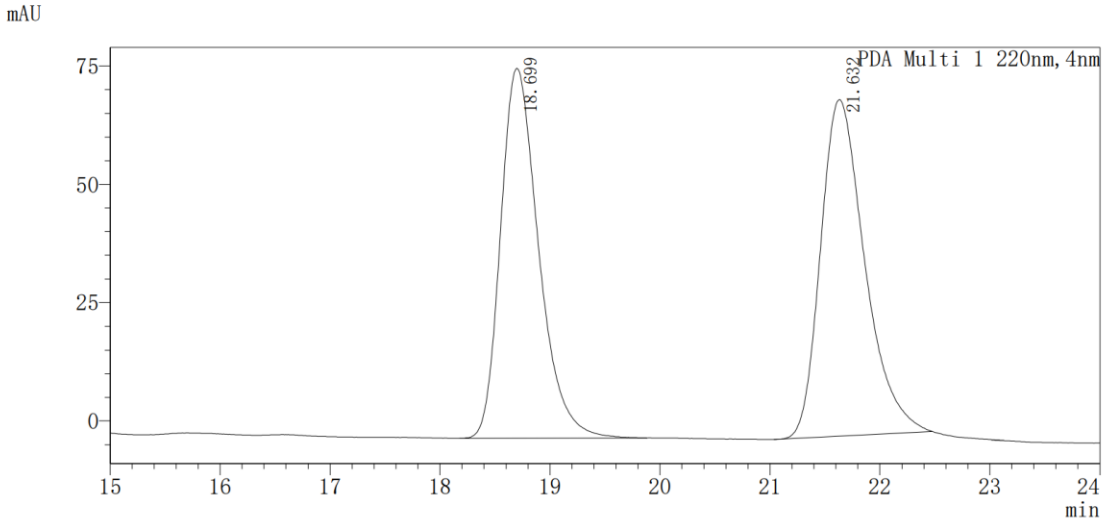

Peak	Ret Time[min]	Area[mAU*s]	Height [mAU]	Area %
1	18.699	1882510	78227	48.646
2	21.632	1987269	71171	51.354
Total		3869779	149398	100.000

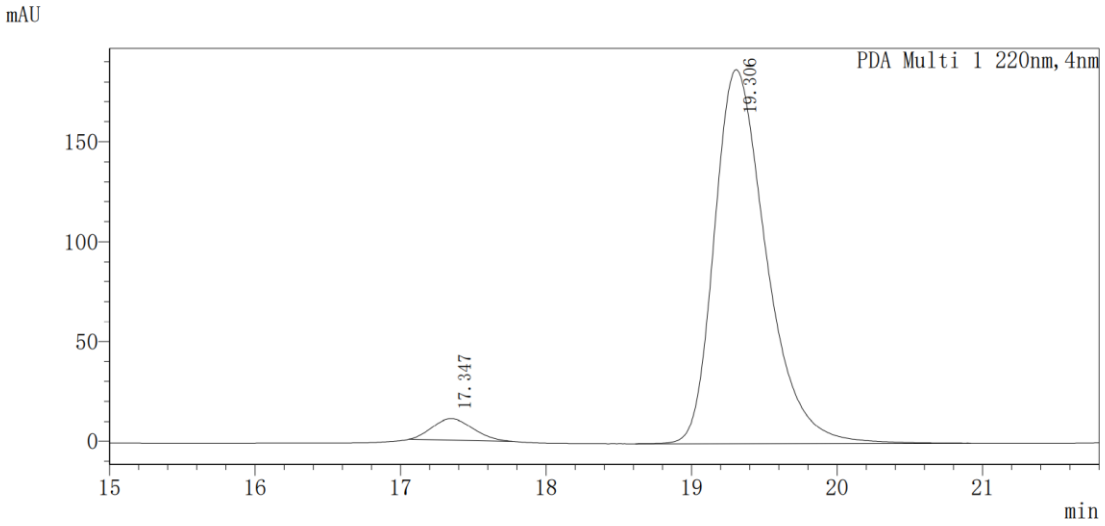

Peak	Ret Time[min]	Area[mAU*s]	Height [mAU]	Area %
1	17.347	211483	10836	4.351
2	19.306	4649563	187425	95.649
Total		4861046	198261	100.000

Supplementary Figure 28. **HPLC chromatography for 3d**

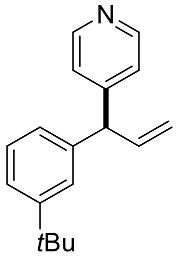

(R)-4-(1-(3-(tert-butyl)phenyl)allyl)pyridine **(3e)**

Following the general procedure, the product **3e** was isolated by chromatography on silica gel (PE/EA = 10/1 ~ 5/1, eluent) as a yellow oil (66%, 33.1 mg); $[\alpha]_D^{25}$ = 35.8 (c 1.5, $CHCl_3$); ^{1}H NMR (400 MHz, $CDCl_3$) δ 8.54 (d, J = 5.6 Hz, 2H), 7.36 (d, J = 8.0 Hz, 2H), 7.15 - 7.11 (m, 4H), 6.31 - 6.22 (m, 1H), 5.29 (d, J = 10.0 Hz, 1H), 5.06 (d, J = 16.8 Hz, 1H), 4.69 (d, J = 7.2 Hz, 1H), 1.33 (s, 9H). ^{13}C NMR (100 MHz, $CDCl_3$) δ 152.24, 149.79, 149.76, 139.07, 138.37, 128.06, 125.54, 123.83, 117.27, 53.91, 34.42, 31.32. **HRMS (ESI)** m/z: $[M+H]^+$ Calcd for $C_{18}H_{22}N$ 252.1752; Found 252.1748.

Analysis of Stereochemistry:

Enantiomeric excess: 90%, determined by HPLC (Daicel Chiralpak ADH hexane/isopropanol = 99/1, flow rate 1.0 mL/min, T = 25 °C, 220nm): t_R = 12.46 min (minor), t_R = 13.84 min (major).

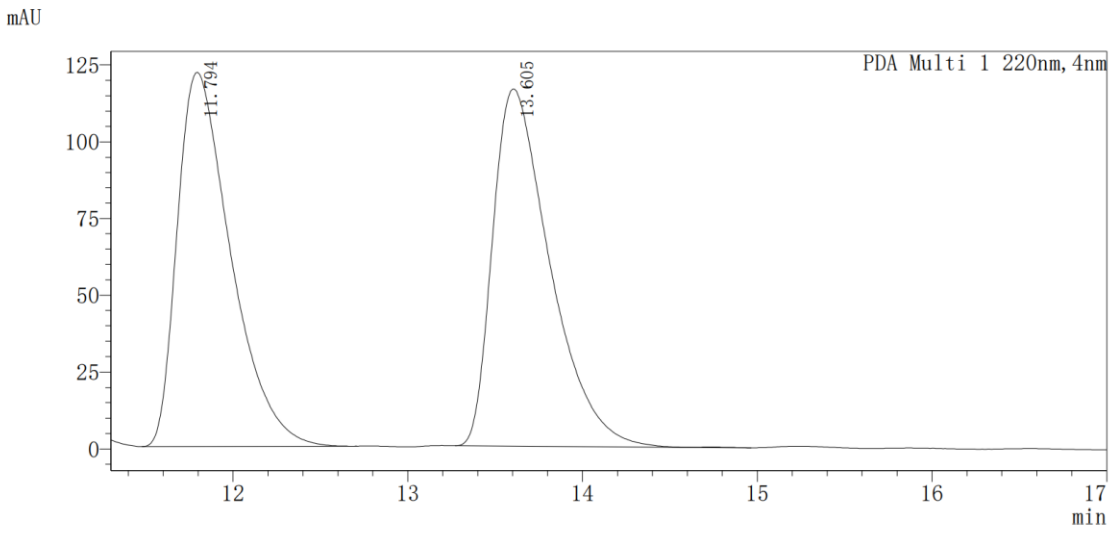

Peak	Ret Time[min]	Area[mAU*s]	Height [mAU]	Area %
1	11. 794	2591391	121760	49. 399
2	13. 605	2654399	116328	50. 601
Total		5245790	238088	100. 000

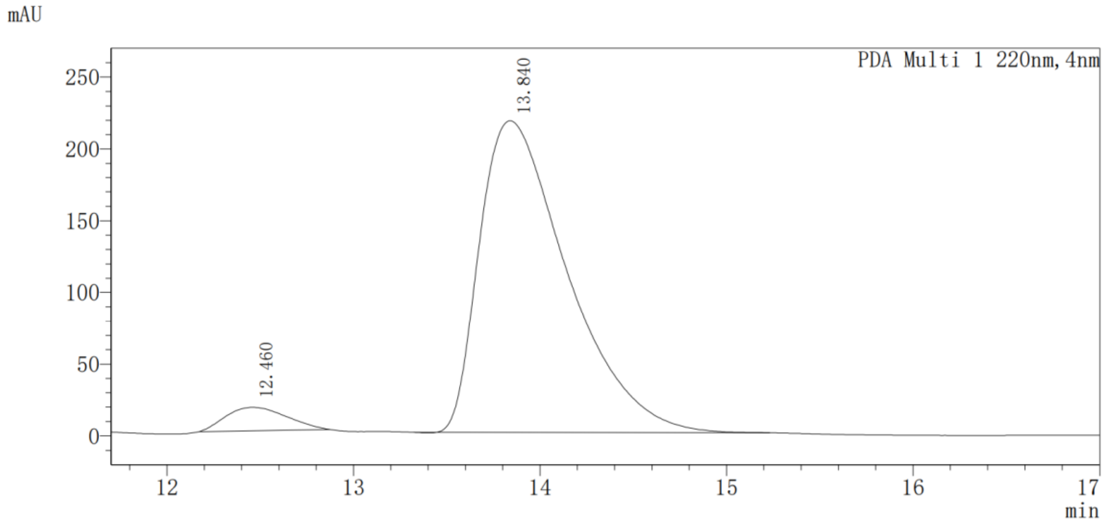

Peak	Ret Time[min]	Area[mAU*s]	Height [mAU]	Area %
1	12. 460	371734	16392	4. 964
2	13. 840	7116344	217286	95. 036
Total		7488078	233678	100. 000

Supplementary Figure 29**. HPLC chromatography for 3e**

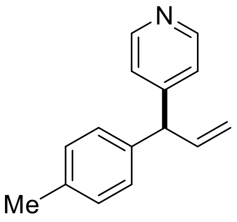

(R)-4-(1-(p-tolyl)allyl)pyridine (**3f**)

Following the general procedure, the product **3f** was isolated by chromatography on silica gel (PE/EA = 10/1 ~ 5/1, eluent) as a yellow oil (75%, 31.3 mg); $[\alpha]_D^{25}$ = 19.2 (c 1.3, $CHCl_3$); ^{1}H NMR (400 MHz, $CDCl_3$) δ 8.53 (dd, *J* = 4.6, 1.4 Hz, 2H), 7.18 - 7.11 (m, 4H), 7.07 (d, *J* = 8.1 Hz, 2H), 6.26 (ddd, *J* = 17.2, 10.2, 7.2 Hz, 1H), 5.29 (dt, *J* = 10.2, 1.2 Hz, 1H), 5.04 (dt, *J* = 17.1, 1.3 Hz, 1H), 4.68 (d, *J* = 7.2 Hz, 1H), 2.36 (s, 3H). ^{13}C NMR (100 MHz, $CDCl_3$) δ 152.39, 149.75, 139.07, 138.51, 136.56, 129.36, 128.38, 123.80, 117.31, 53.97, 20.98. **HRMS (ESI)** m/z: $[M+H]^+$ Calcd for $C_{15}H_{16}N$ 210.1283; Found 210.1281.

Analysis of Stereochemistry:

Enantiomeric excess: 90%, determined by HPLC (Daicel Chiralpak IA hexane/isopropanol = 99.5/0.5, flow rate 1.0 mL/min, T = 25 °C, 220nm): t_R = 29.53 min (minor), t_R = 31.70 min (major).

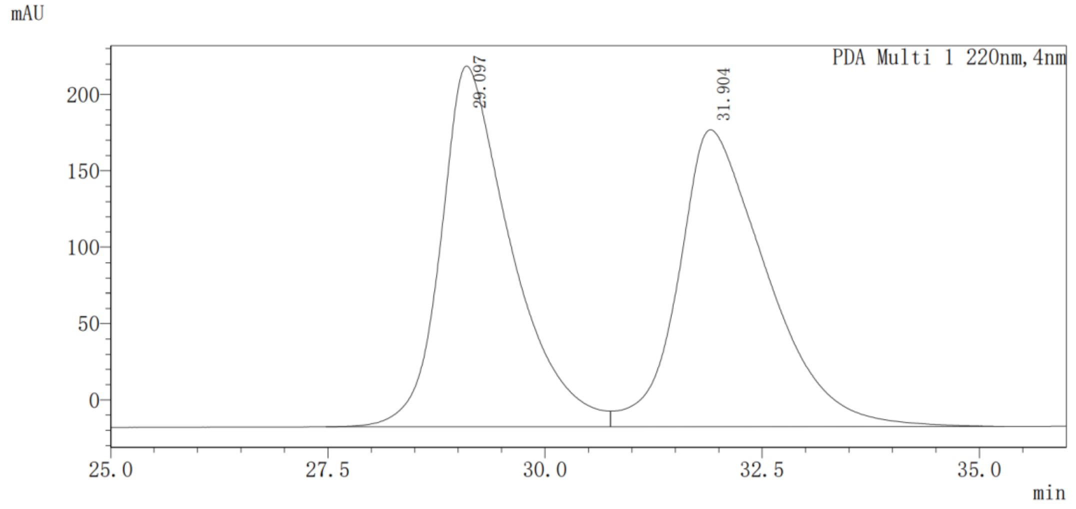

Peak	Ret Time[min]	Area[mAU*s]	Height [mAU]	Area %
1	29.097	13390112	236310	49.246
2	31.904	13799982	194343	50.754
Total		27190094	430653	100.000

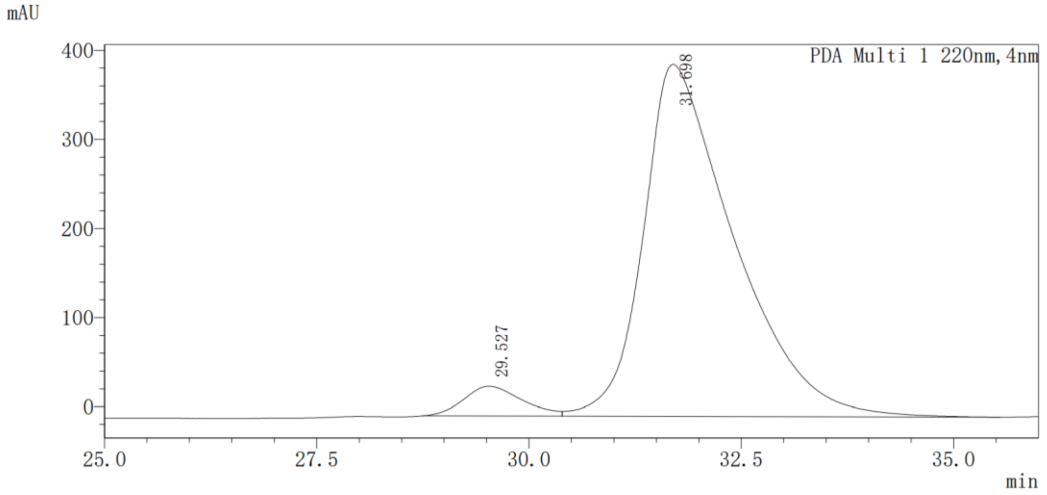

Peak	Ret Time[min]	Area[mAU*s]	Height [mAU]	Area %

1	29.527	1597233	33182	5.156
2	31.698	29380675	395093	94.844
Total		30977908	428274	100.000

Supplementary Figure 30. **HPLC chromatography for 3f**

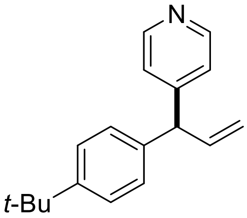

(R)-4-(1-(4-(tert-butyl)phenyl)allyl)pyridine **(3g)**

Following the general procedure, the product **3g** was isolated by chromatography on silica gel (PE/EA = 10/1 ~ 5/1, eluent) as a pale yellow oil (57%, 28.6 mg); $[\alpha]_D^{25}$ = 22.2(c 0.8, $CHCl_3$); ^{1}H NMR (400 MHz, $CDCl_3$) δ 8.51 (d, *J* = 5.2 Hz, 2H), 7.34 (d, *J* = 8.3 Hz, 2H), 7.13 (d, *J* = 5.9 Hz, 2H), 7.09 (d, *J* = 8.4 Hz, 2H), 6.24 (ddd, *J* = 17.3, 10.1, 7.4 Hz, 1H), 5.27 (d, *J* = 10.2 Hz, 1H), 5.03 (d, *J* = 17.1 Hz, 1H), 4.67 (d, *J* = 7.3 Hz, 1H), 1.31 (s, 9H). ^{13}C NMR (100 MHz, $CDCl_3$) δ 152.60, 149.84, 149.53, 139.05, 138.33, 128.08, 125.57, 123.93, 117.30, 53.97, 34.44, 31.33. **HRMS (ESI)** m/z: $[M+H]^+$ Calcd for $C_{18}H_{22}N$ 252.1752; Found 252.1747.

Analysis of Stereochemistry:

Enantiomeric excess: 89%, determined by HPLC (Agela Technologies Venusil CJ hexane/isopropanol = 99/1, flow rate 1.0 mL/min, T = 25 °C, 220nm): t_R = 14.43 min (major), t_R = 22.14 min (minor).

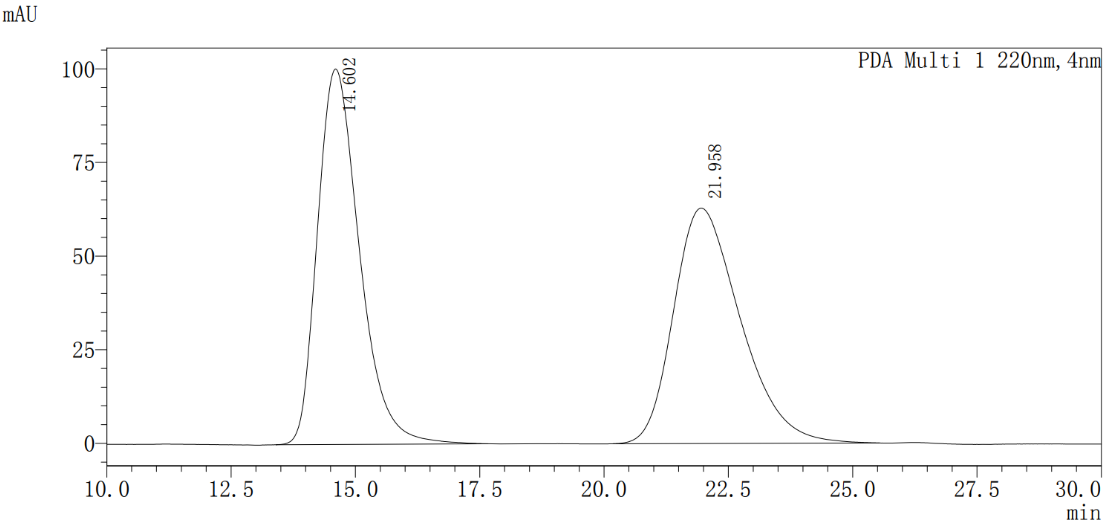

Peak	Ret Time[min]	Area[mAU*s]	Height [mAU]	Area %
1	14.602	5897232	100261	50.067
2	21.958	5881333	62867	49.933
Total		11778565	163128	100.000

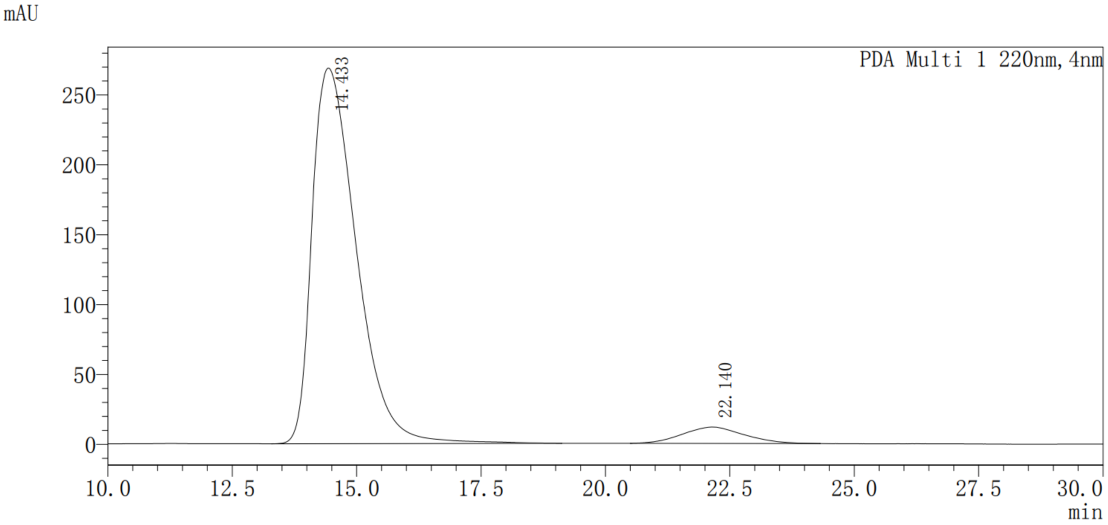

Peak	Ret Time[min]	Area[mAU*s]	Height [mAU]	Area %
1	14.433	16658174	268847	94.337
2	22.140	999916	11681	5.663
Total		17658091	280528	100.000

Supplementary Figure 31. **HPLC chromatography for 3g**

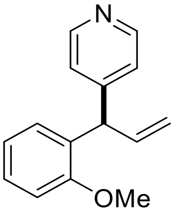

(R)-4-(1-(2-methoxyphenyl)allyl)pyridine **(3h)**

Following the general procedure, the product **3h** was isolated by chromatography on silica gel (PE/EA = 10/1 ~ 5/1, eluent) as a yellow oil (80%, 36.0 mg); $[\alpha]_D^{25}$ = 18.8 (c 1.6, $CHCl_3$); ^{1}H NMR (400 MHz, $CDCl_3$) δ 8.50 (d, *J* = 6.0 Hz, 2H), 7.30 - 7.26 (m, 1H), 7.17 (dd, *J* = 7.6, 1.6 Hz, 1H), 7.12 (d, *J* = 6.0 Hz, 2H), 6.98 (t, *J* = 6.8, 1H), 6.90 (d, *J* = 8.4 Hz, 1H), 6.32 - 6.23 (m, 1H), 5.28 (d, *J* = 10.4 Hz, 1H), 5.10 (d, *J* = 6.8 Hz, 1H), 4.98 (dd, *J* = 17.2, 1.4 Hz, 1H), 3.75 (s, 3H). ^{13}C NMR (100 MHz, $CDCl_3$) δ 156.79, 152.42, 149.44, 138.54, 129.91, 129.09, 128.18, 123.87, 120.57, 117.26, 110.82, 55.36, 47.45. **HRMS (ESI)** m/z: $[M+H]^+$ Calcd for $C_{15}H_{16}NO$ 226.1232; Found 226.1233.

Analysis of Stereochemistry:

Enantiomeric excess: 94%, determined by HPLC (Daicel Chiralpak ADH hexane/isopropanol = 99/1, flow rate 1.0 mL/min, T = 25 °C, 220nm): t_R = 20.15 min (major), t_R = 22.46 min (minor).

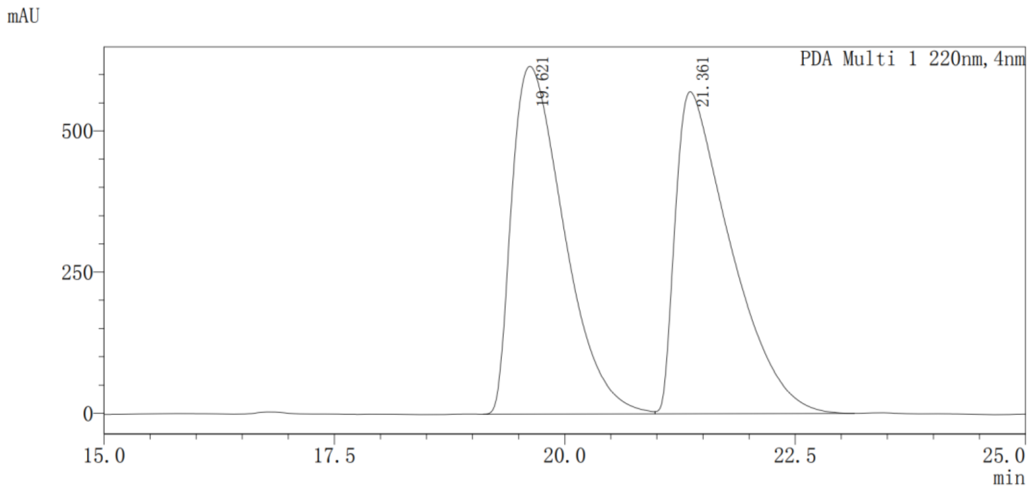

Peak	Ret Time[min]	Area[mAU*s]	Height [mAU]	Area %
1	19.621	24385644	616307	50.508
2	21.361	23895588	571118	49.492
Total		48281232	1187424	100.000

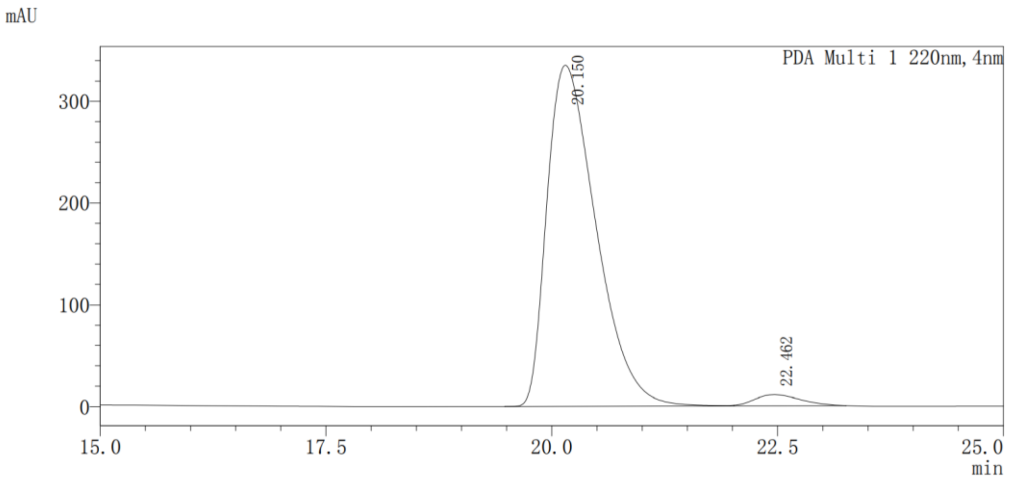

Peak	Ret Time[min]	Area[mAU*s]	Height [mAU]	Area %
1	20.150	12674831	335029	97.068
2	22.462	382868	10869	2.932
Total		13057699	345898	100.000

Supplementary Figure 32. **HPLC chromatography for 3h**

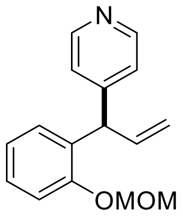

***(R)-4-(1-(2-(methoxymethoxy)phenyl)allyl)pyridine* (3i)**

Following the general procedure, the product **3i** was isolated by chromatography on silica gel (PE/EA = 10/1 ~ 5/1, eluent) as a yellow oil (52%, 26.5 mg); $[\alpha]_D^{25}$ = 14.1 (c 0.7, $CHCl_3$); ^1H NMR (400 MHz, $CDCl_3$) δ 8.50 (d, *J* = 5.8 Hz, 2H), 7.26 (dd, *J* = 12.3, 4.9 Hz, 1H), 7.20 (d, *J* = 7.5 Hz, 1H), 7.11 (dd, *J* = 10.4, 7.1 Hz, 3H), 7.03 (t, *J* = 7.4 Hz, 1H), 6.34 - 6.22 (m, 1H), 5.30 (d, *J* =

10.2 Hz, 1H), 5.10 (dd, J = 18.3, 6.8 Hz, 3H), 5.00 (d, J = 17.2 Hz, 1H), 3.26 (s, 3H). ^{13}C NMR (100 MHz, $CDCl_3$) δ 154.35, 152.24, 149.53, 138.44, 130.48, 129.23, 128.21, 123.78, 121.69, 117.39, 113.97, 93.98, 55.87, 47.90. **HRMS (ESI)** m/z: $[M+H]^+$ Calcd for $C_{16}H_{18}NO_2$ 256.1338; Found 256.1337.

Analysis of Stereochemistry:

Enantiomeric excess: 90%, determined by HPLC (Daicel Chiralpak ADH hexane/isopropanol = 99.5/0.5, flow rate 1.0 mL/min, T = 25 °C, 220nm): t_R= 40.37 min (minor), t_R= 45.31 min (major).

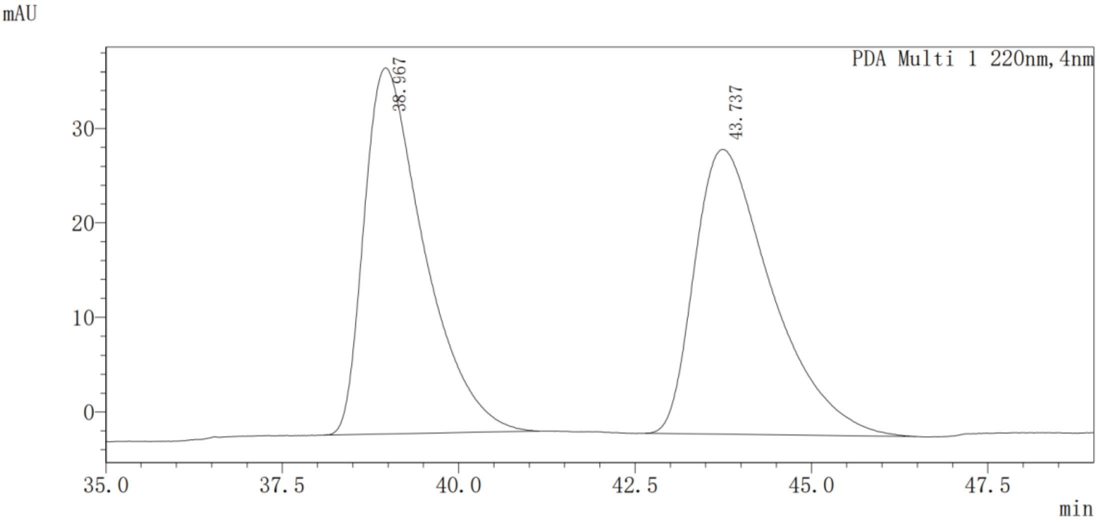

Peak	Ret Time[min]	Area[mAU*s]	Height [mAU]	Area %
1	38. 967	2290797	38738	49. 887
2	43. 737	2301201	30136	50. 113
Total		4591998	68875	100. 000

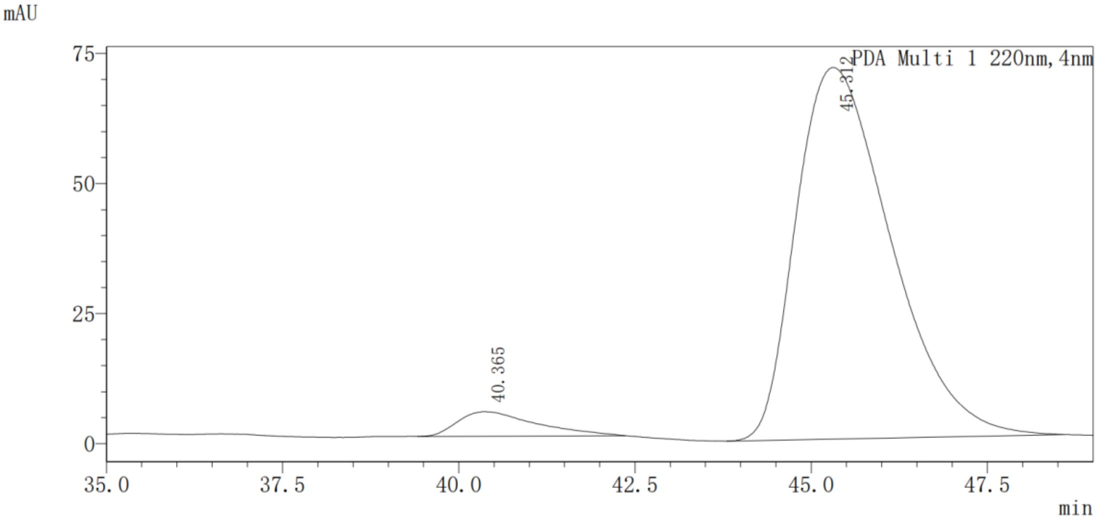

Peak	Ret Time[min]	Area[mAU*s]	Height [mAU]	Area %
1	40. 365	367155	4741	5. 193
2	45. 312	6703240	71439	94. 807
Total		7070395	76179	100. 000

Supplementary Figure 33**. HPLC chromatography for 3i**

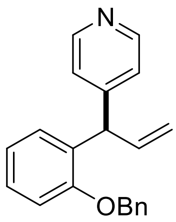

(R)-4-(1-(2-(benzyloxy)phenyl)allyl)pyridine **(3j)**

Following the general procedure, the product **3j** was isolated by chromatography on silica gel (PE/EA = 10/1 ~ 5/1, eluent) as a yellow oil (72%, 43.3 mg); $[\alpha]_D^{25}$ = 23.8 (c 0.8, $CHCl_3$); ^{1}H NMR (400 MHz, $CDCl_3$) δ 8.49 (d, *J* = 5.1 Hz, 2H), 7.34 (d, *J* = 6.2 Hz, 3H), 7.28 - 7.22 (m, 2H), 7.18 (d, *J* = 7.0 Hz, 2H), 7.08 (d, *J* = 5.1 Hz, 2H), 7.01 (t, *J* = 7.4 Hz, 1H), 6.96 (d, *J* = 8.1 Hz, 1H), 6.29 (ddd, *J* = 17.2, 9.9, 7.2 Hz, 1H), 5.29 (d, *J* = 10.2 Hz, 1H), 5.12 (d, *J* = 6.8 Hz, 1H), 5.05 - 4.93 (m, 3H). ^{13}C NMR (100 MHz, $CDCl_3$) δ 155.92, 152.23, 149.49, 138.50, 136.73, 130.35, 129.23, 128.41, 128.18, 127.86, 127.28, 123.88, 120.85, 117.34, 112.01, 70.04, 47.98. **HRMS (ESI)** m/z: $[M+H]^+$ Calcd for $C_{21}H_{20}NO$ 302.1545; Found 302.1545.

Analysis of Stereochemistry:

Enantiomeric excess: 91%, determined by HPLC (Agela Technologies Venusil CJ, hexane/isopropanol = 98.5/1.5, flow rate 1.0 mL/min, T = 25 °C, 220nm): t_R = 30.41 min (major), t_R = 38.98 min (minor).

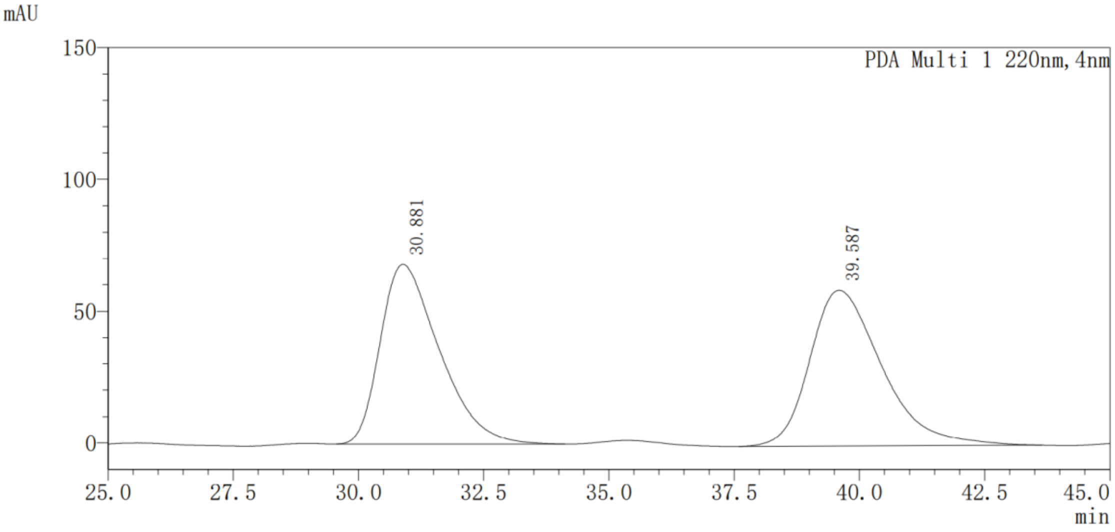

Peak	Ret Time[min]	Area[mAU*s]	Height [mAU]	Area %
1	30.881	5608792	68255	48.581
2	39.587	5936533	59152	51.419
Total		11545325	127407	100.000

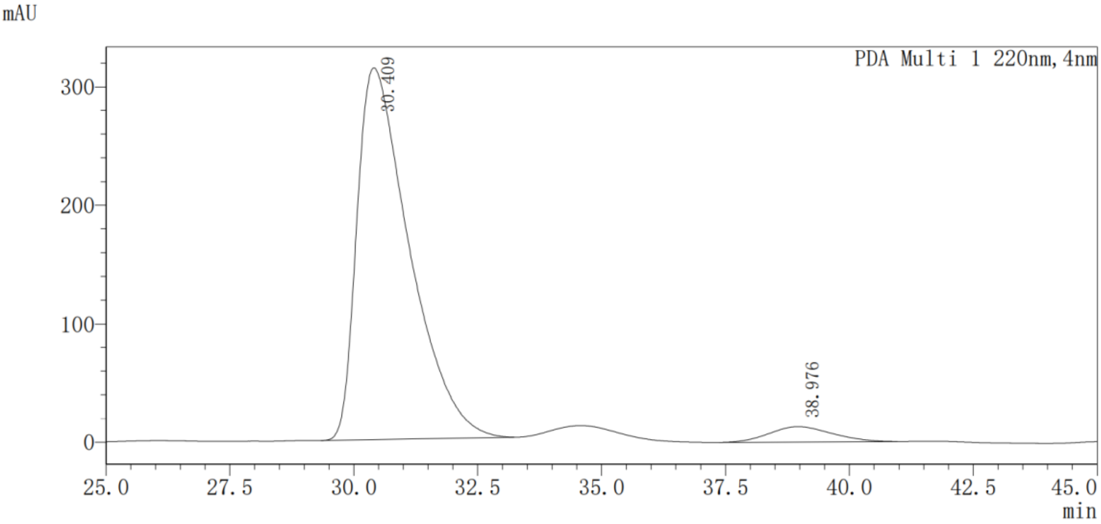

Peak	Ret Time[min]	Area[mAU*s]	Height [mAU]	Area %
1	30.409	23117021	313680	95.310
2	38.976	1137445	12922	4.690
Total		24254466	326602	100.000

Supplementary Figure 34. **HPLC chromatography for 3j**

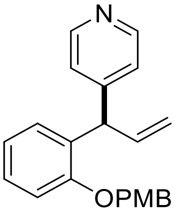

(R)-4-(1-(2-((4-methoxybenzyl)oxy)phenyl)allyl)pyridine **(3k)**

Following the general procedure, the product **3k** was isolated by chromatography on silica gel (PE/EA = 10/1 ~ 5/1, eluent) as a yellow oil (70%, 46.3 mg); $[\alpha]_D^{25}$ = 17.6 (c 1.1, $CHCl_3$); ^{1}H NMR (400 MHz, $CDCl_3$) δ 8.47 (d, *J* = 5.9 Hz, 2H), 7.24 (ddd, *J* = 12.2, 7.6, 1.5 Hz, 2H), 7.07 (dd, *J* = 10.6, 7.3 Hz, 4H), 6.98 (ddd, *J* = 11.7, 9.2, 4.5 Hz, 2H), 6.90 - 6.85 (m, 2H), 6.28 (ddd, *J* = 17.2, 10.2, 7.0 Hz, 1H), 5.27 (dt, *J* = 10.2, 1.2 Hz, 1H), 5.08 (d, *J* = 7.0 Hz, 1H), 4.98 (dt, *J* = 17.2, 1.4 Hz, 1H), 4.91 (q, *J* = 11.1 Hz, 2H), 3.83 (s, 3H). ^{13}C NMR (100 MHz, $CDCl_3$) δ 159.32, 155.95, 152.33, 149.37, 138.48, 130.32, 129.13, 128.97, 128.74, 128.14, 123.90, 120.72, 117.30, 113.77, 112.02, 69.79, 55.23, 47.99. **HRMS (ESI)** m/z: $[M+H]^+$ Calcd for $C_{22}H_{22}NO_2$ 332.1651; Found 332.1645.

Analysis of Stereochemistry:

Enantiomeric excess: 92%, determined by HPLC (Daicel Chiralpak OZH hexane/isopropanol = 90/10, flow rate 1.0 mL/min, T = 25 °C, 220nm): t_R= 13.71 min (major), t_R= 17.42 min (minor).

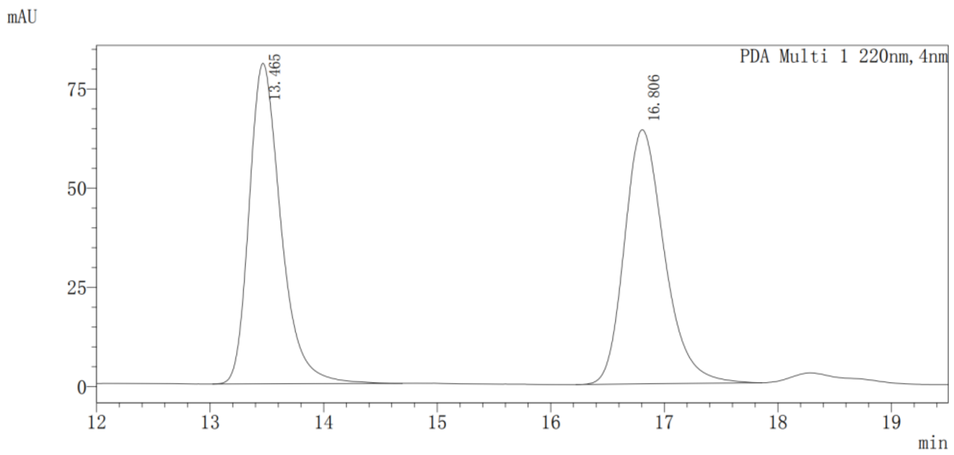

Peak	Ret Time[min]	Area[mAU*s]	Height [mAU]	Area %
1	13.465	1572531	80751	50.012
2	16.806	1571758	64025	49.988
Total		3144289	144776	100.000

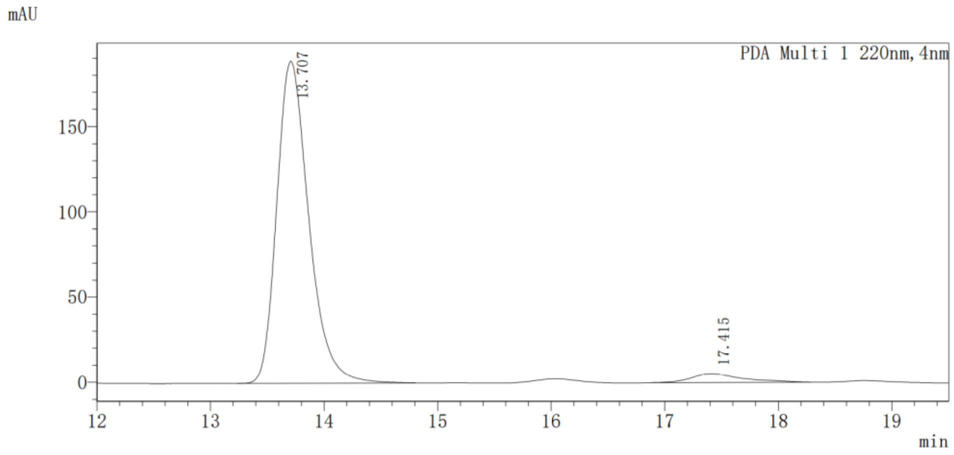

Peak	Ret Time[min]	Area[mAU*s]	Height [mAU]	Area %
1	13.707	3699229	188793	95.756
2	17.415	163968	5245	4.244
Total		3863198	194038	100.000

Supplementary Figure 35. **HPLC chromatography for 3k**

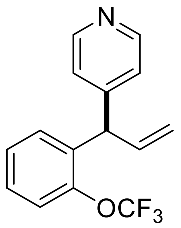

***(R)-4-(1-(2-(trifluoromethoxy)phenyl)allyl)pyridine* (3l)**

Following the general procedure, the product **3l** was isolated by chromatography on silica gel (PE/EA = 10/1 ~ 5/1, eluent) as a pale yellow oil (56%, 31.2 mg); $[\alpha]_D^{25}$ = 27.1 (c 0.9, $CHCl_3$); ^1H NMR (400 MHz, $CDCl_3$) δ 8.51 (dd, *J* = 4.5, 1.6 Hz, 2H), 7.31 (dd, *J* = 7.0, 2.4 Hz, 1H), 7.29 – 7.26 (m, 2H), 7.22 (dd, *J* = 8.0, 2.0 Hz, 1H), 7.13 - 7.06 (m, 2H), 6.24 - 6.15 (m, 1H), 5.33 (dt, *J* = 10.2, 1.2 Hz, 1H), 5.07 (d, *J* = 6.8 Hz, 1H), 5.00 (dt, *J* = 17.1, 1.3 Hz, 1H). ^{13}C NMR (100 MHz,

$CDCl_3$) δ 150.84, 149.87, 137.42, 133.88, 130.14, 128.46, 126.85, 123.67, 120.33, 118.33, 47.25.
HRMS (ESI) m/z: $[M+H]^+$ Calcd for $C_{15}H_{13}F_3NO$ 280.0949; Found 280.0949.

Analysis of Stereochemistry:

Enantiomeric excess: 86%, determined by HPLC (Daicel Chiralpak OZH hexane/isopropanol = 99.5/0.5, flow rate 1.0 mL/min, T = 25 °C, 220nm): t_R= 27.49 min (minor), t_R= 29.06 min (major).

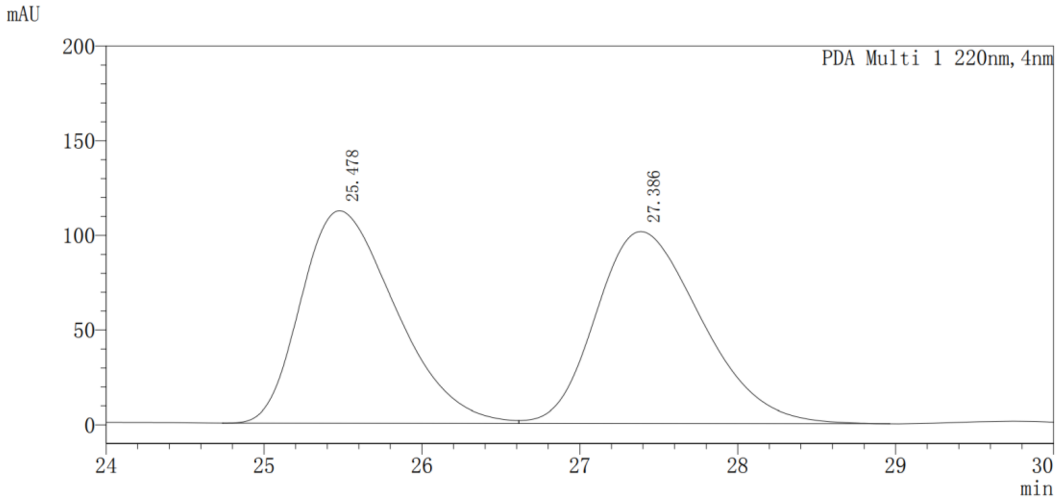

Peak	Ret Time[min]	Area[mAU*s]	Height [mAU]	Area %
1	25.478	4668158	112041	49.844
2	27.386	4697296	101236	50.156
Total		9365454	213277	100.000

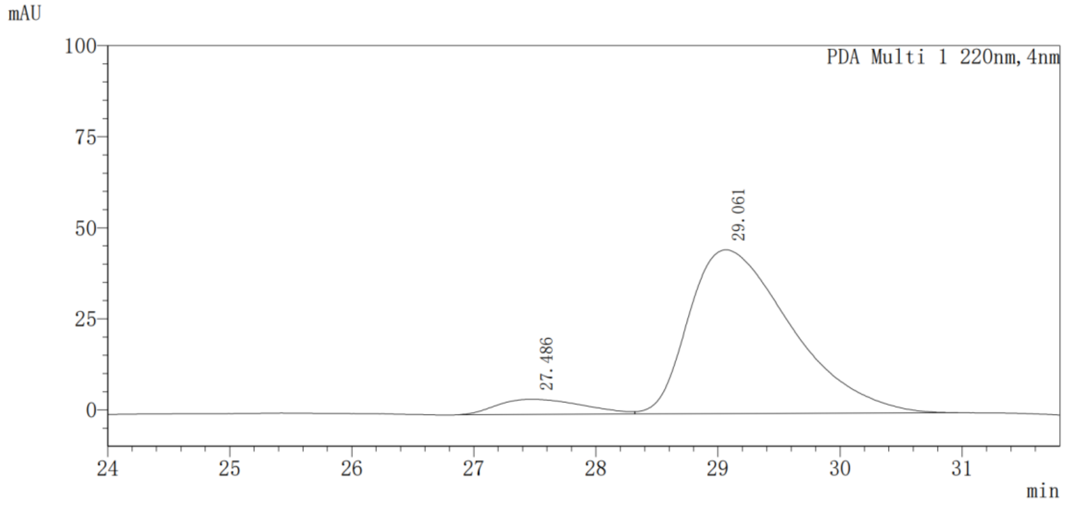

Peak	Ret Time[min]	Area[mAU*s]	Height [mAU]	Area %
1	27.486	203844	4155	7.202
2	29.061	2626688	44989	92.798
Total		2830532	49144	100.000

Supplementary Figure 36. **HPLC chromatography for 3l**

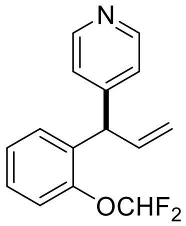

(R)-4-(1-(2-(difluoromethoxy)phenyl)allyl)pyridine **(3m)**

Following the general procedure, the product **3m** was isolated by chromatography on silica gel (PE/EA = 10/1 ~ 5/1, eluent) as a yellow oil (50%, 26.1 mg); $[\alpha]_D^{25}$ = 30.5 (c 1.4, $CHCl_3$); ^{1}H NMR (400 MHz, $CDCl_3$) δ 8.53 (d, J = 5.8 Hz, 2H), 7.32 (dd, J = 8.6, 4.3 Hz, 1H), 7.23 (d, J = 4.2 Hz, 2H), 7.17 - 7.10 (m, 3H), 6.37 (t, J = 74.0 Hz, 1H), 6.30 - 6.20 (m, 1H), 5.34 (d, J = 10.2 Hz, 1H), 5.13 (d, J = 6.8 Hz, 1H), 5.01 (d, J = 17.1 Hz, 1H). ^{13}C NMR (100 MHz, $CDCl_3$) δ 151.21, 149.78, 137.78, 132.65 (d, J_{C-F} = 101.5 Hz), 130.01, 129.61 (d, J_{C-F} = 41.4 Hz), 128.46, 125.63, 123.74, 118.92, 118.10, 116.17, 113.59, 47.36.^{19}F NMR (376 MHz, $CDCl_3$) δ -79.82. **HRMS (ESI)** m/z: $[M+H]^+$ Calcd for $C_{15}H_{14}F_2NO$ 262.1043; Found 262.1039.

Analysis of Stereochemistry:

Enantiomeric excess: 90%, determined by HPLC (Daicel Chiralpak IA hexane/isopropanol = 99 /1, flow rate 1.0 mL/min, T = 25 °C, 220nm): tR= 23.95 min (major), tR= 27.21 min (minor).

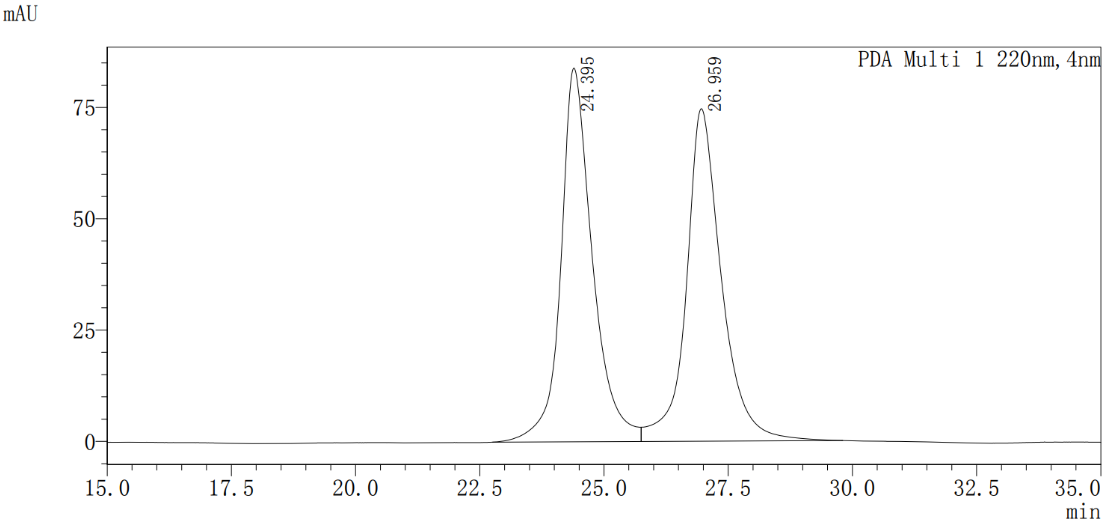

Peak	Ret Time[min]	Area[mAU*s]	Height [mAU]	Area %
1	24. 395	83935	3720699	50. 211
2	26. 959	74680	3689433	49. 789
Total		158614	7410133	100. 000

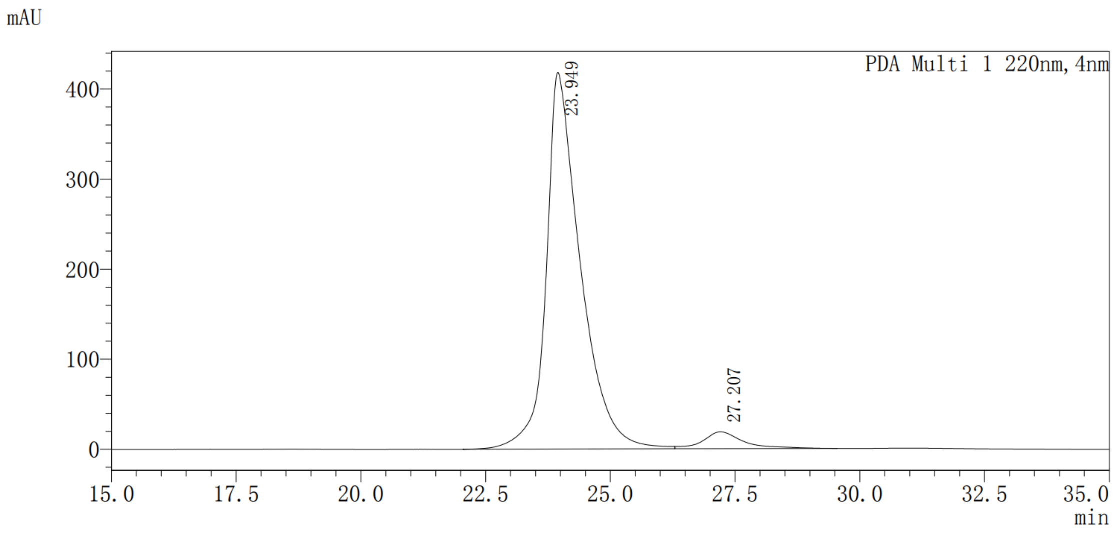

Peak	Ret Time[min]	Area[mAU*s]	Height [mAU]	Area %
1	23.949	418169	19949206	94.983
2	27.207	18745	1053630	5.017
Total		436914	21002837	100.000

Supplementary Figure 37**. HPLC chromatography for 3m**

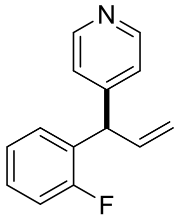

(R)-4-(1-(2-fluorophenyl)allyl)pyridine **(3n)**

Following the general procedure, the product **3n** was isolated by chromatography on silica gel (PE/EA = 10/1 ~ 5/1, eluent) as a yellow oil (52%, 22.1 mg); $[\alpha]_D^{25}$ = 19.5 (c 0.9, $CHCl_3$); ^{1}H NMR (400 MHz, $CDCl_3$) δ 8.52 (d, J = 5.8 Hz, 2H), 7.26 - 7.23 (m, 1H), 7.21 - 7.15 (m, 1H), 7.15 - 7.10 (m, 3H), 7.08 - 7.01 (m, 1H), 6.29 - 6.19 (m, 1H), 5.32 (dt, J = 10.2, 1.1 Hz, 1H), 5.04 (d, J = 17.2 Hz, 1H), 5.00 (d, J = 7.0 Hz, 1H). ^{13}C NMR (100 MHz, $CDCl_3$) δ 149.83, 137.37, 129.79, 129.75, 128.84, 128.76, 124.32, 124.28, 123.61, 118.09, 115.84, 115.62, 47.27. **HRMS (ESI)** m/z: $[M+H]^+$ Calcd for $C_{14}H_{13}FN$ 214.1032; Found 214.1031.

Analysis of Stereochemistry:

Enantiomeric excess: 87%, determined by HPLC (Agela Technologies Venusil CJ hexane/isopropanol = 99/1, flow rate 1.0 mL/min, T = 25 °C, 220nm): t_R= 13.12 min (minor), t_R= 14.18 min (major).

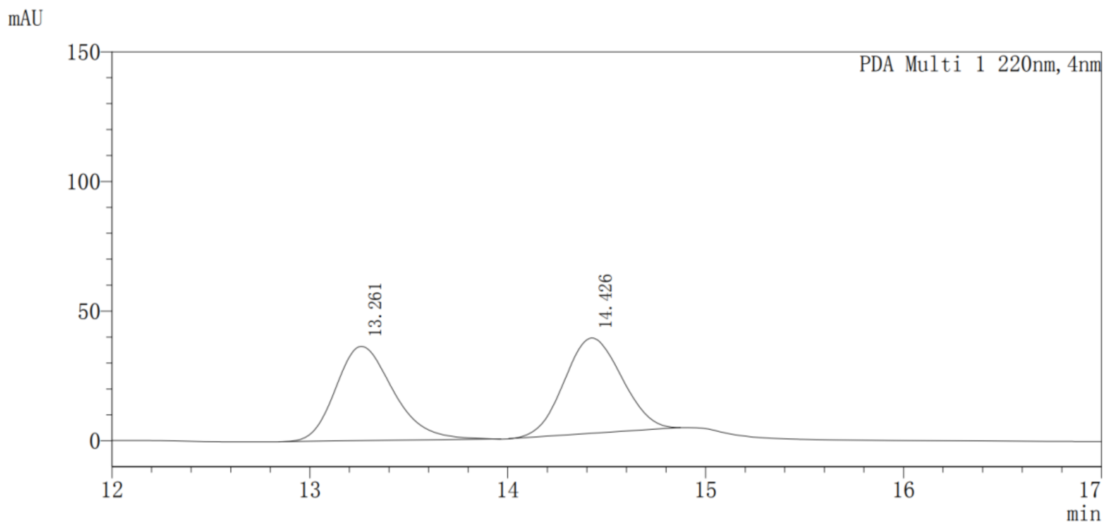

Peak	Ret Time[min]	Area[mAU*s]	Height [mAU]	Area %
1	13. 261	736864	36362	49. 733
2	14. 426	744779	36676	50. 267
Total		1481643	73038	100. 000

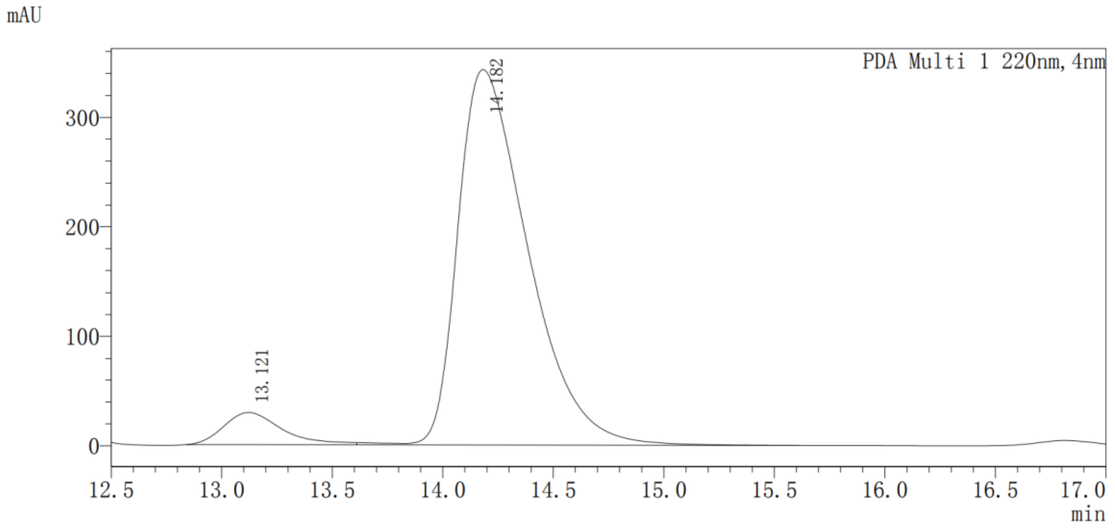

Peak	Ret Time[min]	Area[mAU*s]	Height [mAU]	Area %
1	13. 121	542045	29398	6. 671
2	14. 182	7583747	342691	93. 329
Total		8125792	372089	100. 000

Supplementary Figure 38. **HPLC chromatography for 3n**

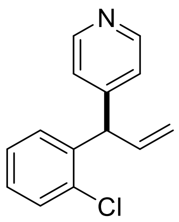

(R)-4-(1-(2-chlorophenyl)allyl)pyridine **(3o)**

Following the general procedure, the product **3o** was isolated by chromatography on silica gel (PE/EA = 10/1 ~ 5/1, eluent) as a yellow oil (42%, 19.2 mg); $[\alpha]_D^{25}$ = 24.9 (c 0.8, $CHCl_3$); ^{1}H NMR (400 MHz, $CDCl_3$) δ 8.51 (dd, *J* = 4.6, 1.5 Hz, 2H), 7.39 (dd, *J* = 7.6, 1.6 Hz, 1H), 7.28 - 7.26 (m, 1H), 7.25 - 7.22 (m, 1H), 7.19 (dd, *J* = 7.2, 1.9 Hz, 1H), 7.09 (d, *J* = 6.0 Hz, 2H), 6.21 (ddd, *J* = 17.1, 10.2, 6.6 Hz, 1H), 5.33 (dd, *J* = 10.2, 1.2 Hz, 1H), 5.20 (d, *J* = 6.5 Hz, 1H), 4.97 (dd, *J* = 17.2, 1.2 Hz, 1H). ^{13}C NMR (100 MHz, $CDCl_3$) δ 150.78, 149.74, 138.99, 137.64, 134.27, 129.95, 128.35, 127.01, 123.98, 118.30, 50.40. **HRMS (ESI)** m/z: $[M+H]^+$ Calcd for $C_{14}H_{13}ClN$ 230.0737; Found 230.0739.

Analysis of Stereochemistry:

Enantiomeric excess: 90%, determined by HPLC (Agela Technologies Venusil CJ hexane/isopropanol = 99/1, flow rate 1.0 mL/min, T = 25 °C, 220nm): tR= 14.10 min (minor), tR= 15.44 min (major).

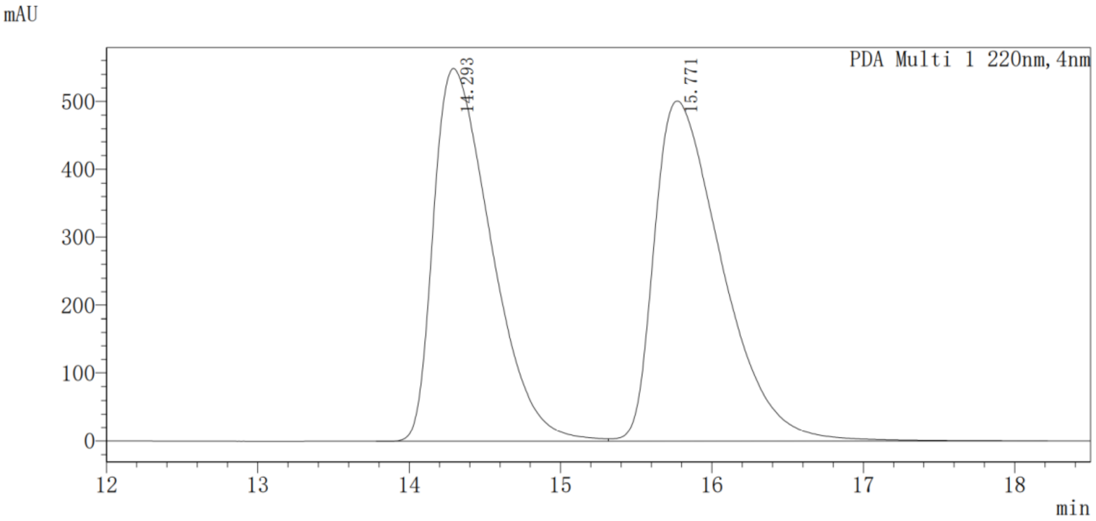

Peak	Ret Time[min]	Area[mAU*s]	Height [mAU]	Area %
1	14.293	14537023	548815	48.230
2	15.771	15603819	501052	51.770
Total		30140842	1049868	100.000

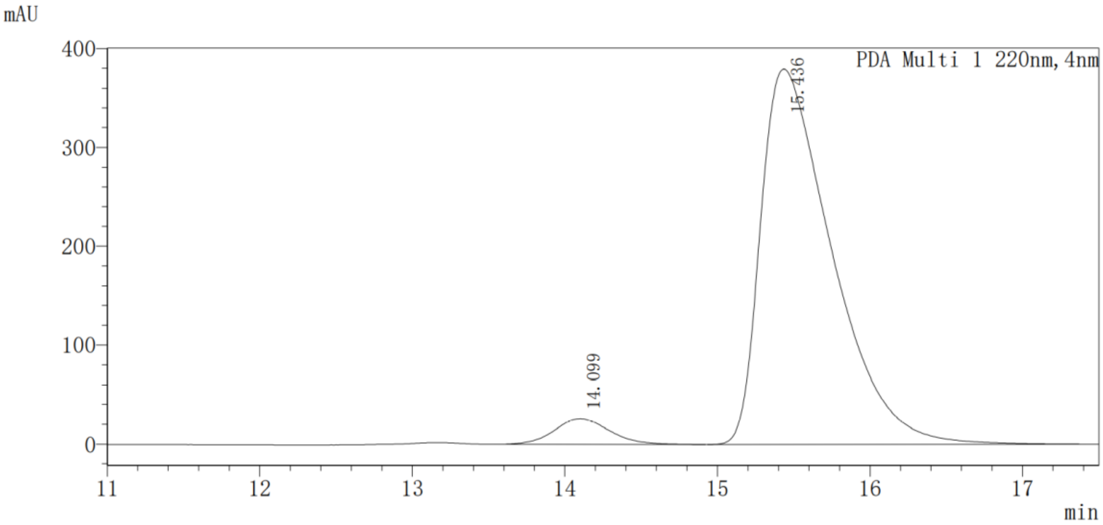

Peak	Ret Time[min]	Area[mAU*s]	Height [mAU]	Area %
1	14.099	657169	25960	5.070
2	15.436	12304055	379490	94.930
Total		12961224	405450	100.000

Supplementary Figure 39**. HPLC chromatography for 3o**

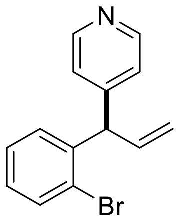

(R)-4-(1-(2-bromophenyl)allyl)pyridine **(3p)**

Following the general procedure, the product **3p** was isolated by chromatography on silica gel (PE/EA = 10/1 ~ 5/1, eluent) as a yellow oil (32%, 17.5 mg); $[\alpha]_D^{25}$ = 41.4 (c 1.0, $CHCl_3$); ^{1}H NMR (400 MHz, $CDCl_3$) δ 8.52 (d, J = 5.6 Hz, 2H), 7.59 (d, J = 8.0 Hz, 1H), 7.30 (t, J = 7.21 Hz, 1H), 7.18 (dd, J = 7.8, 1.6 Hz, 1H), 7.16 - 7.11 (m, 1H), 7.09 (d, J = 6.0 Hz, 2H), 6.25 - 6.16 (m, 1H), 5.34 (d, J = 10.3 Hz, 1H), 5.20 (d, J = 6.4 Hz, 1H), 4.96 (d, J = 17.2 Hz, 1H). ^{13}C NMR (100 MHz, $CDCl_3$) δ 150.71, 149.79, 140.65, 137.79, 133.31, 130.18, 128.62, 127.65, 125.12, 124.06, 118.35, 52.90. **HRMS (ESI)** m/z: $[M+H]^+$ Calcd for $C_{14}H_{13}BrN$ 274.0231; Found 274.0231.

Analysis of Stereochemistry:

Enantiomeric excess: 94%, determined by HPLC (Agela Technologies Venusil CJ hexane/isopropanol = 99/1, flow rate 1.0 mL/min, T = 25 °C, 220nm): t_R= 15.27 min (minor), t_R= 17.55 min (major).

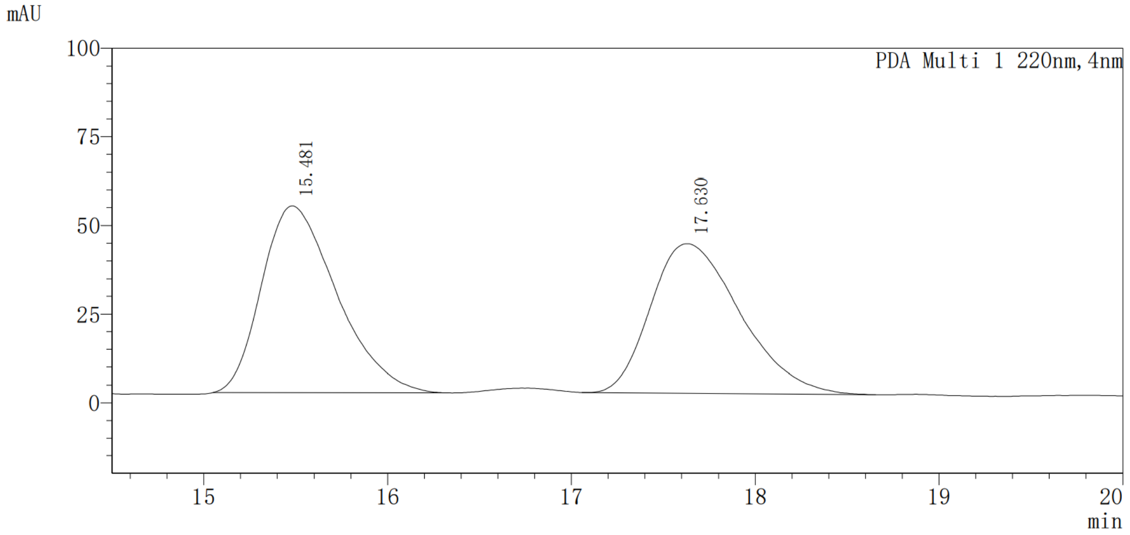

Peak	Ret Time[min]	Area[mAU*s]	Height [mAU]	Area %
1	15.481	1496370	52637	51.144
2	17.630	1429422	42220	48.856
Total		2925793	94858	100.000

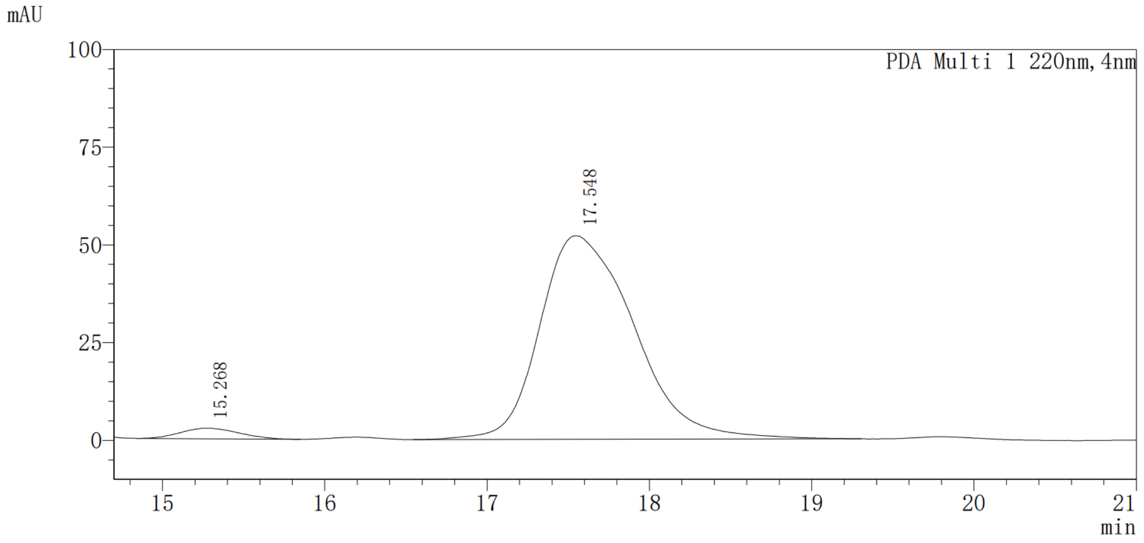

Peak	Ret Time[min]	Area[mAU*s]	Height [mAU]	Area %
1	15. 268	68256	2725	3. 142
2	17. 548	2104040	52100	96. 858
Total		2172297	54826	100. 000

Supplementary Figure 40**. HPLC chromatography for 3p**

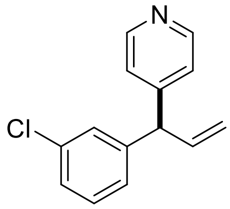

(R)-4-(1-(3-chlorophenyl)allyl)pyridine **(3q)**

Following the general procedure, the product **3q** was isolated by chromatography on silica gel (PE/EA = 10/1 ~ 5/1, eluent) as a yellow oil (40%, 18.3 mg); $[\alpha]_D^{25}$ = 17.2 (c 1.3, $CHCl_3$); ^{1}H NMR (400 MHz, $CDCl_3$) δ 8.53 (d, *J* = 6.0 Hz, 2H), 7.26 - 7.22 (m, 2H), 7.16 (d, *J* = 2.0 Hz, 1H), 7.10 (dd, *J* = 4.8, 1.4 Hz, 2H), 7.05 - 7.03 (m, 1H), 6.25 - 6.16 (m, 1H), 5.32 (dt, *J* = 10.0, 1.2 Hz, 1 H), 5.04 (dt, *J* = 17.2, 1.2 Hz, 1H), 4.67 (d, *J* = 7.2 Hz, 1H). ^{13}C NMR (100 MHz, $CDCl_3$) δ 151.36, 149.95, 143.50, 138.14, 134.61, 129.92, 128.68, 127.20, 126.75, 123.76, 118.20, 53.94. **HRMS (ESI)** m/z: $[M+H]^+$ Calcd for $C_{14}H_{13}ClN$ 230.0737; Found 230.0737.

Analysis of Stereochemistry:

Enantiomeric excess: 92%, determined by HPLC (Daicel Chiralpak ADH, hexane/isopropanol = 98/2, flow rate 1.0 mL/min, T = 25 °C, 220nm): t_R= 13.07 min (major), t_R= 14.06 min (minor)

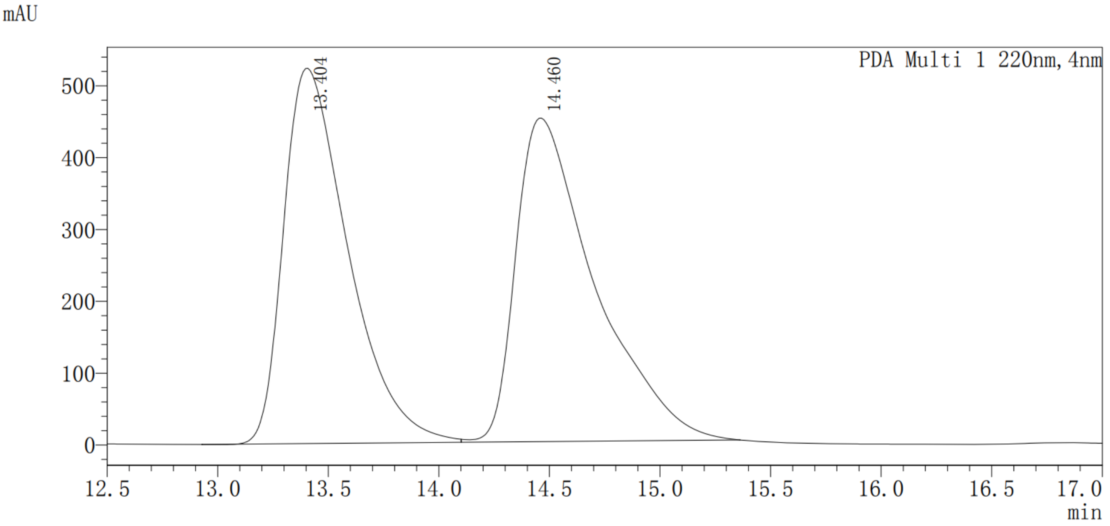

Peak	Ret Time[min]	Area[mAU*s]	Height [mAU]	Area %
1	13.404	10607894	522289	48.768
2	14.460	11143790	450255	51.232
Total		21751684	972544	100.000

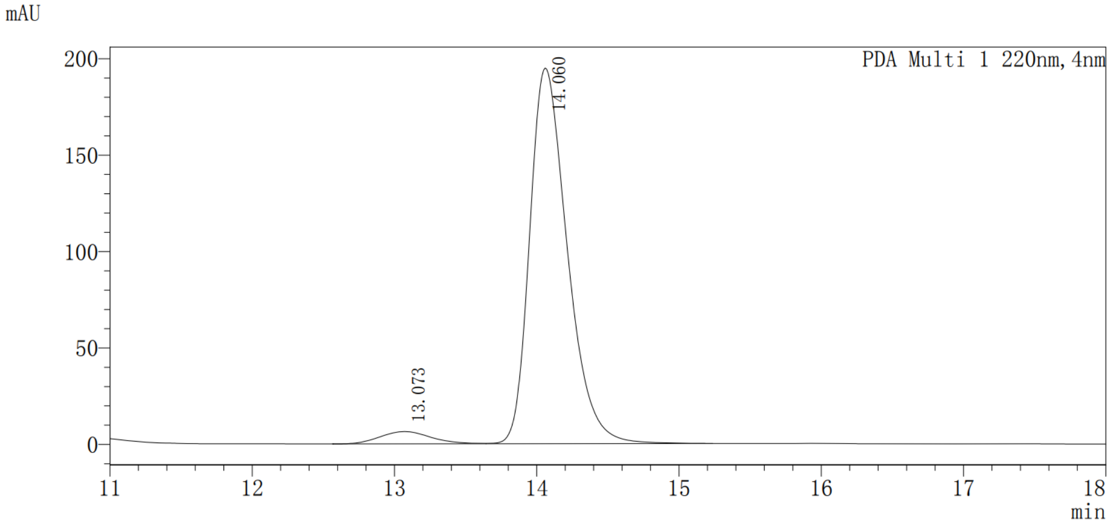

Peak	Ret Time[min]	Area[mAU*s]	Height [mAU]	Area %
1	13.073	6395	159401	4.263
2	14.060	194814	3579518	95.737
Total		201209	3738918	100.000

Supplementary Figure 41. **HPLC chromatography for 3q**

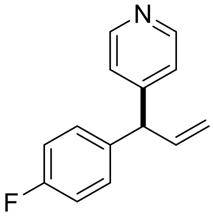

(R)-4-(1-(4-fluorophenyl)allyl)pyridine **(3r)**

Following the general procedure, the product **3r** was isolated by chromatography on silica gel (PE/EA = 10/1 ~ 5/1, eluent) as a yellow oil (46%, 19.6 mg); $[\alpha]_D^{25}$ = 16.6 (c 0.7, $CHCl_3$); ^1H NMR

(400 MHz, $CDCl_3$) δ 8.52 (d, J = 5.2 Hz, 2H), 7.14 - 7.09 (m, 4H), 7.03 - 6.99 (m, 2H), 6.25 -6.17 (m, 1H), 5.31 - 5.28 (m, 1H), 5.04 - 4.99 (m, 1H), 4.68 (d, J = 7.2 Hz, 1H). ^{13}C NMR (100 MHz, $CDCl_3$) δ 163.02, 160.58(d, J_{C-F} = 244.3 Hz), 151.90, 149.90, 138.74, 137.21, 137.17(d, J_{C-F} = 4.0 Hz),130.08, 130.00(d, J_{C-F} = 8.0 Hz), 123.75, 117.74, 115.63, 115.42(d, J_{C-F} = 21.0 Hz), 53.52. **HRMS (ESI)** m/z: $[M+H]^+$ Calcd for $C_{14}H_{13}FN$ 214.1032; Found 214.1031.

Analysis of Stereochemistry:

Enantiomeric excess: 85%, determined by HPLC (Daicel Chiralpak ADH hexane/isopropanol = 99/1, flow rate 1.0 mL/min, T = 25 °C, 220nm): t_R= 26.43 min (major), t_R= 28.77 min (minor).

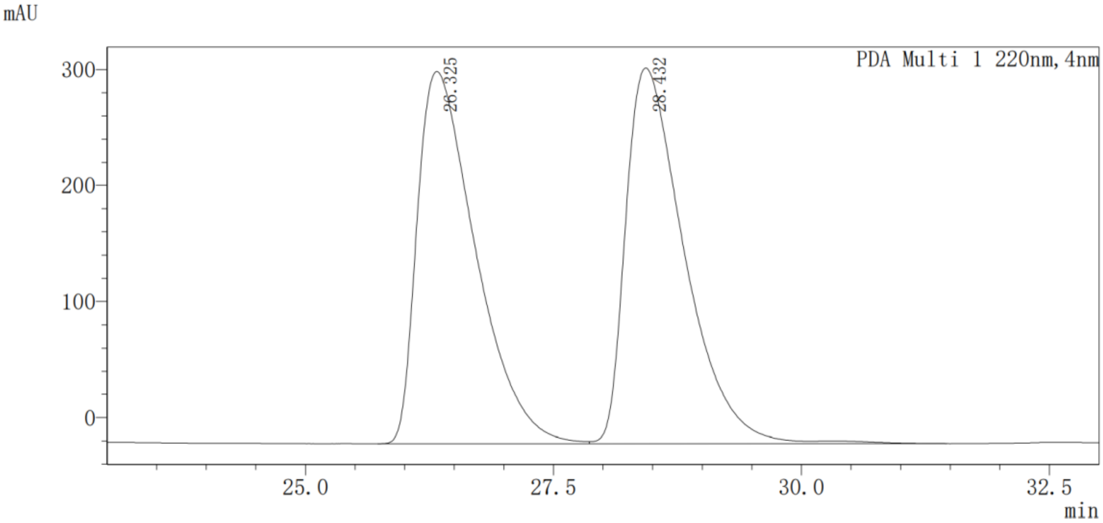

Peak	Ret Time[min]	Area[mAU*s]	Height [mAU]	Area %
1	26. 325	13112932	320729	49. 583
2	28. 432	13333509	323618	50. 417
Total		26446442	644348	100. 000

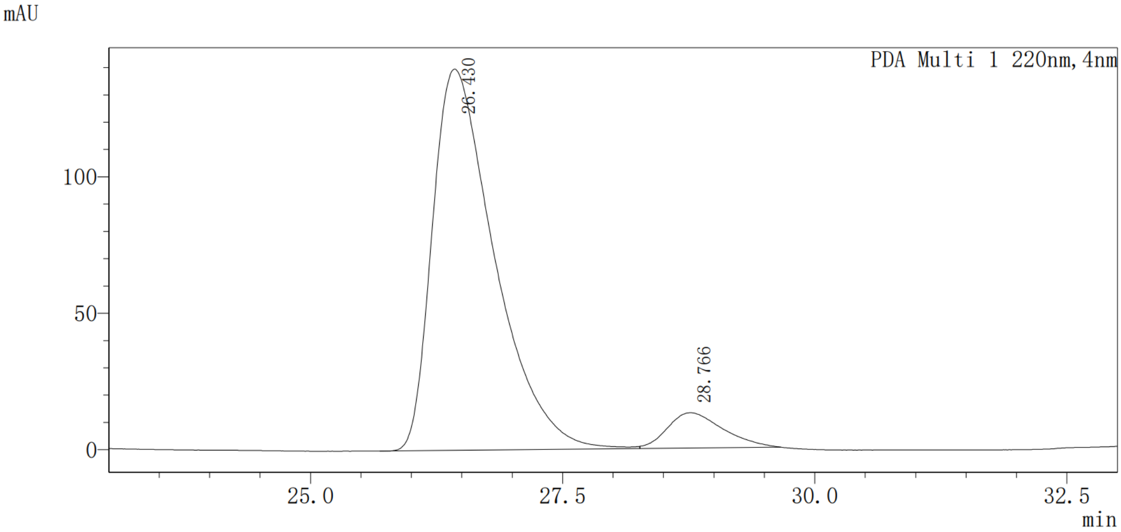

Peak	Ret Time[min]	Area[mAU*s]	Height [mAU]	Area %
1	26. 430	5998690	139703	92. 361
2	28. 766	496157	12952	7. 639
Total		6494847	152655	100. 000

Supplementary Figure 42. **HPLC chromatography for 3r**

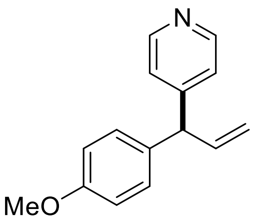

***(R)-4-(1-(4-methoxyphenyl)allyl)pyridine* (3s)**

Following the general procedure, the product **3s** was isolated by chromatography on silica gel (PE/EA = 10/1 ~ 5/1, eluent) as a yellow oil (70%, 31.5 mg); $[\alpha]_D^{25}$ = 31.8 (c 1.3, $CHCl_3$); ^{1}H NMR (400 MHz, $CDCl_3$) δ 8.50 (d, *J* = 6.0 Hz, 2H), 7.11 - 7.06 (m, 4H), 6.86 (d, *J* = 8.8 Hz, 2H), 6.27 - 6.16 (m, 1H), 5.26 (d, *J* = 10.4 Hz, 1H), 5.00 (d, *J* = 16.8 Hz, 1H), 4.65 (d, *J* = 7.2 Hz, 1H), 3.79 (s, 3H). ^{13}C NMR (100 MHz, $CDCl_3$) δ 158.50, 152.47, 149.71, 139.15, 133.53, 129.48, 123.77, 117.21, 114.06, 76.68, 55.23, 53.48. **HRMS (ESI)** m/z: $[M+H]^+$ Calcd For $C_{15}H_{16}NO$: 226.1232; Found: 226.1227.

Analysis of Stereochemistry:

Enantiomeric excess: 89%, determined by HPLC (Daicel Chiralpak ADH hexane/isopropanol = 99/1, flow rate 1.0 mL/min, T = 25 °C, 220nm): t_R= 51.00 min (major), t_R= 57.74 min (minor).

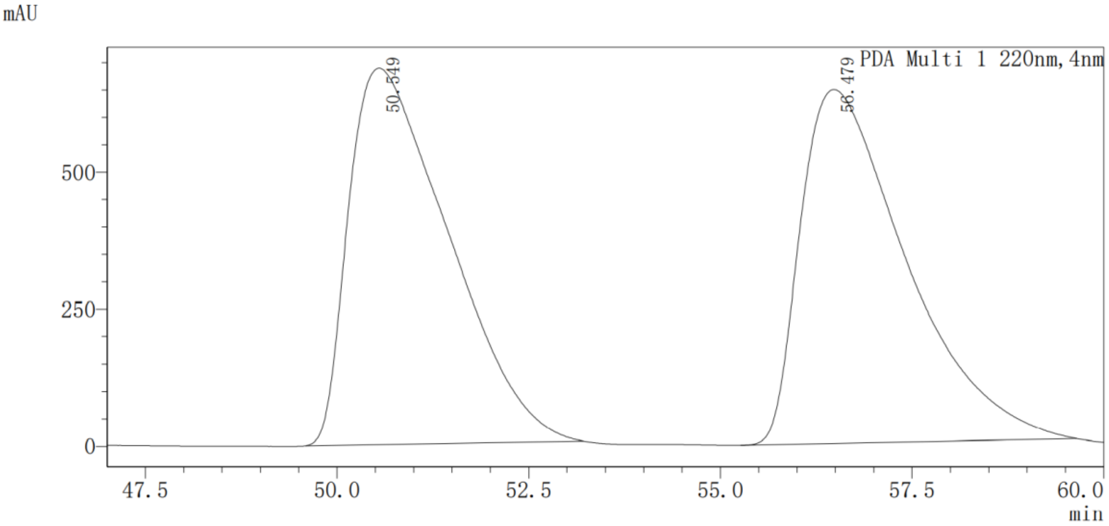

Peak	Ret Time[min]	Area[mAU*s]	Height [mAU]	Area %
1	50. 549	62125612	686503	50. 044
2	56. 479	62016797	645827	49. 956
Total		124142409	1332330	100. 000

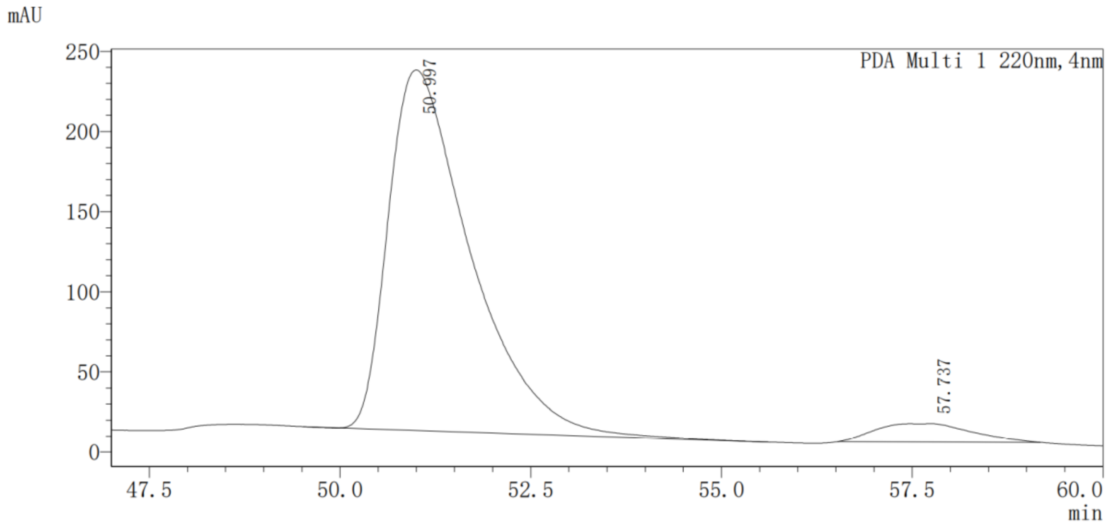

Peak	Ret Time[min]	Area[mAU*s]	Height [mAU]	Area %
1	50.997	16839001	224688	94.320
2	57.737	1014031	11590	5.680
Total		17853033	236277	100.000

Supplementary Figure 43. **HPLC chromatography for 3s**

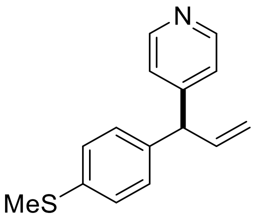

(R)-4-(1-(4-(methylthio)phenyl)allyl)pyridine **(3t)**

Following the general procedure, the product **3t** was isolated by chromatography on silica gel (PE/EA = 10/1 ~ 5/1, eluent) as a yellow oil (53%, 25.5 mg); $[\alpha]_D^{25}$ = 29.2 (c 0.8, $CHCl_3$); ^{1}H NMR (400 MHz, $CDCl_3$) δ 8.51 (d, J = 5.9 Hz, 2H), 7.24 - 7.21 (m, 1H), 7.21 - 7.19 (m, 1H), 7.11 (d, J = 6.0 Hz, 2H), 7.09 - 7.05 (m, 2H), 6.21 (ddd, J = 17.2, 10.2, 7.2 Hz, 1H), 5.28 (dt, J = 10.2, 1.1 Hz, 1H), 5.02 (dt, J = 17.1, 1.3 Hz, 1H), 4.66 (d, J = 7.2 Hz, 1H), 2.47 (s, 3H). ^{13}C NMR (100 MHz, $CDCl_3$) δ 152.13, 149.69, 138.70, 138.35, 137.12, 129.00, 126.97, 123.82, 117.65, 53.78, 15.90. **HRMS (ESI)** m/z: $[M+H]^+$ Calcd For $C_{15}H_{16}NS$: 242.1003; Found: 242.1005.

Analysis of Stereochemistry:

Enantiomeric excess: 86%, determined by HPLC (Daicel Chiralpak ADH hexane/isopropanol = 98.5/1.5, flow rate 1.0 mL/min, T = 25 °C, 220nm): t_R= 34.06 min (major), t_R= 37.72 min (minor).

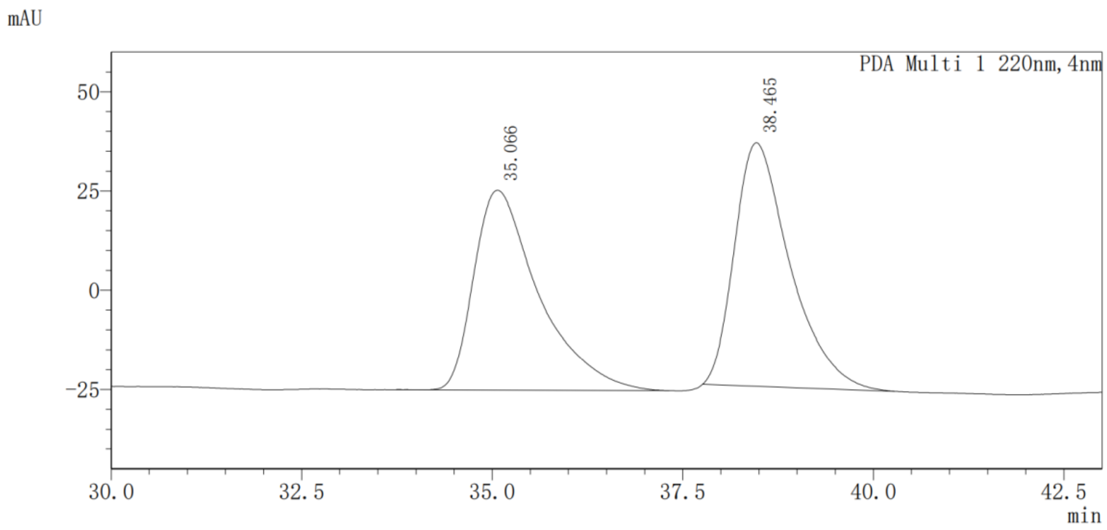

Peak	Ret Time[min]	Area[mAU*s]	Height [mAU]	Area %
1	35. 066	2982744	50350	48. 918
2	38. 465	3114665	61349	51. 082
Total		6097409	111699	100. 000

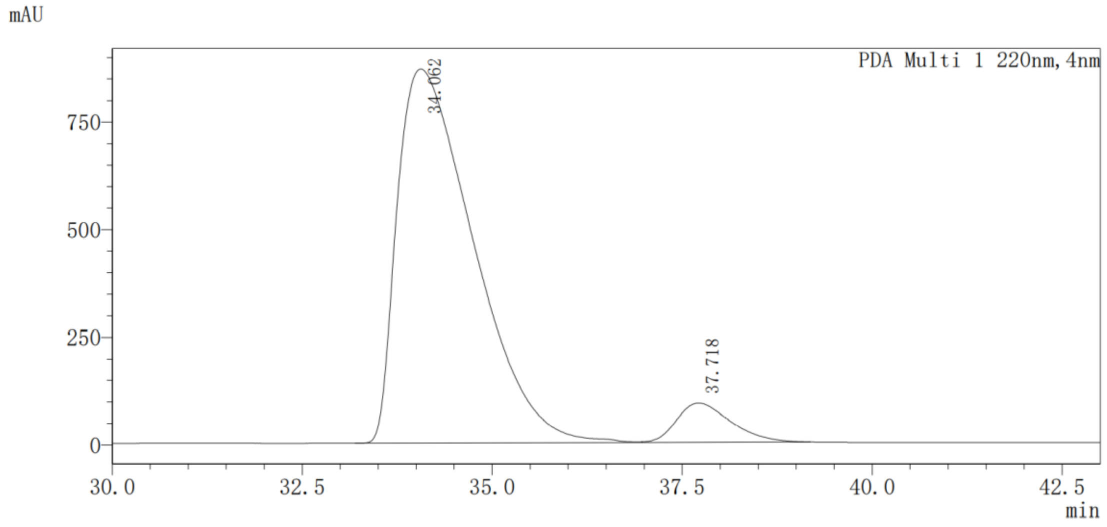

Peak	Ret Time[min]	Area[mAU*s]	Height [mAU]	Area %
1	34. 062	60598503	868331	93. 031
2	37. 718	4539778	91477	6. 969
Total		65138281	959808	100. 000

Supplementary Figure 44**. HPLC chromatography for 3t**

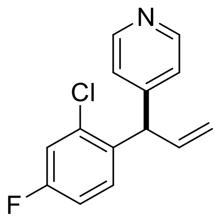

***(R)-4-(1-(2-chloro-4-fluorophenyl)allyl)pyridine* (3u)**

Following the general procedure, the product **3t** was isolated by chromatography on silica gel (PE/EA = 10/1 ~ 5/1, eluent) as a yellow oil (52%, 25.7 mg); $[\alpha]_D^{25}$ = 33.8 (c 0.7, $CHCl_3$); ^{1}H NMR (400 MHz, $CDCl_3$) δ 8.52 (dd, *J* = 4.4, 1.6 Hz, 2H), 7.17 - 7.13 (m, 2H), 7.06 (dd, *J* = 4.8, 1.6 Hz, 2H), 7.00 - 6.95 (m, 1H), 6.21 - 6.13 (m, 1H), 5.33 (d, *J* = 10.0 Hz, 1H), 5.14 (d, *J* = 6.4 Hz, 1H), 4.94 (d, *J* = 17.2 Hz, 1H). ^{13}C NMR (100 MHz, $CDCl_3$) δ 162.64, 160.16 (d, $J_{C\text{-}F}$ = 248 Hz), 150.40, 149.86, 137.43, 134.98, 134.94 (d, $J_{C\text{-}F}$ = 4.0 Hz), 134.87, 134.77, 130.90, 130.82 (d, $J_{C\text{-}F}$ = 8.0 Hz), 123.83, 118.46, 117.35, 117.11 (d, $J_{C\text{-}F}$ = 24 Hz), 114.33, 114.12, 49.76. **HRMS (ESI)** m/z: $[M+H]^+$ Calcd For $C_{14}H_{12}ClFN$: 248.0642; Found: 248.0643.

Analysis of Stereochemistry:

Enantiomeric excess: 90%, determined by HPLC (Daicel Chiralpak OZH hexane/isopropanol = 98/2, flow rate 1.0 mL/min, T = 25 °C, 220nm): t_R= 17.63 min (major), t_R= 18.77 min (minor).

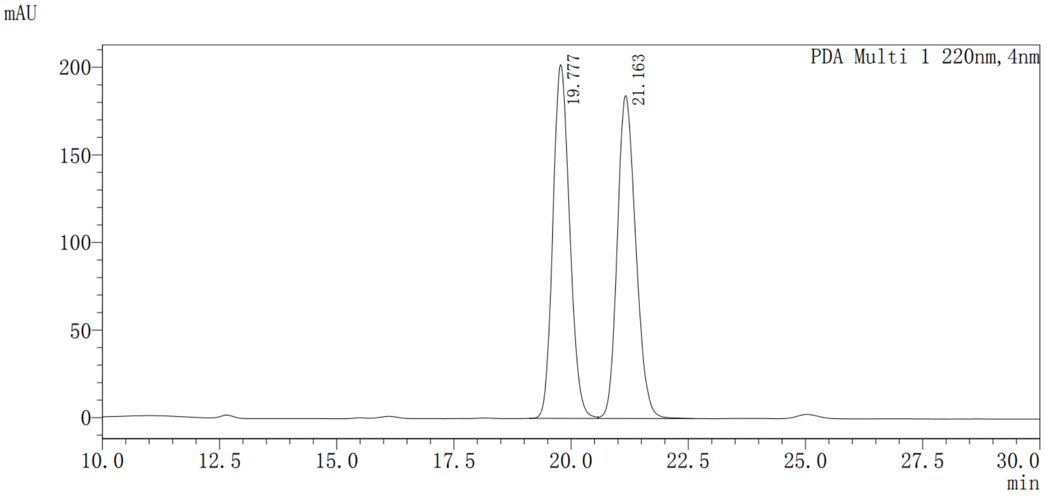

Peak	Ret Time[min]	Area[mAU*s]	Height [mAU]	Area %
1	19.777	5035282	201910	49.870
2	21.163	5061518	184283	50.130
Total		10096800	386193	100.000

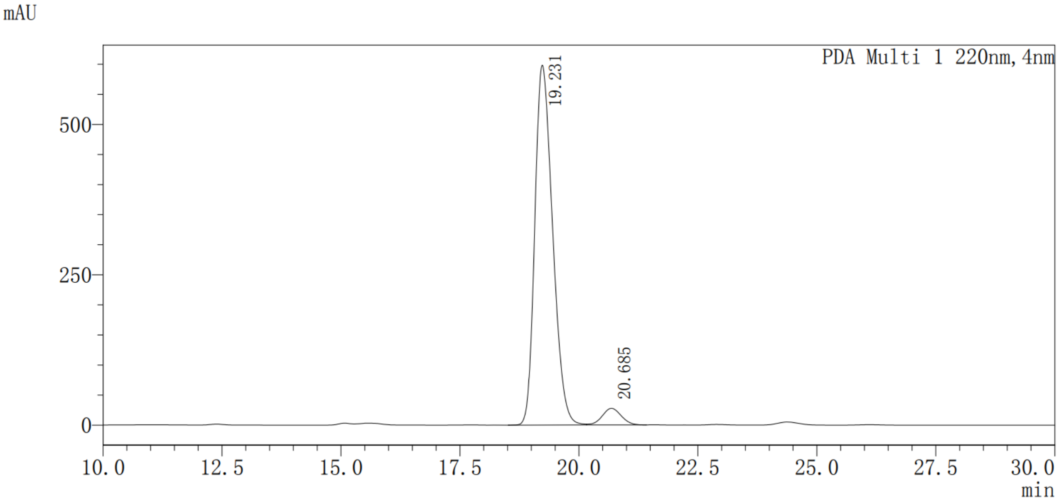

Peak	Ret Time[min]	Area[mAU*s]	Height [mAU]	Area %

1	19. 231	15569793	598370	95. 305
2	20. 685	766958	27639	4. 695
Total		16336751	626009	100. 000

Supplementary Figure 45**. HPLC chromatography for 3u**

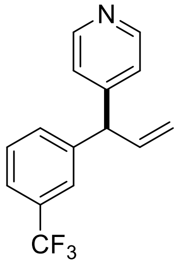

(*R*)-4-(1-(3-(trifluoromethyl)phenyl)allyl)pyridine (3v)

Following the general procedure, the product **3v** was isolated by chromatography on silica gel (PE/EA = 10/1 ~ 5/1, eluent) as a yellow oil (35%, 18.4 mg); $[\alpha]_D^{25}$ = 26.7 (c 0.6, $CHCl_3$); ^{1}H NMR (400 MHz, $CDCl_3$) δ 8.55 (d, *J* = 5.2 Hz, 2H), 7.53 (d, *J* = 7.8 Hz, 1H), 7.45 (dd, *J* = 9.0, 6.2 Hz, 2H), 7.33 (d, *J* = 7.7 Hz, 1H), 7.11 (d, *J* = 6.0 Hz, 2H), 6.23 (ddd, *J* = 17.3, 10.2, 7.2 Hz, 1H), 5.35 (d, *J* = 10.2 Hz, 1H), 5.05 (d, *J* = 17.1 Hz, 1H), 4.77 (d, *J* = 7.2 Hz, 1H). ^{13}C NMR (100 MHz, $CDCl_3$) δ 151.38, 149.79, 142.40, 137.97, 131.94, 129.18, 125.24 (q, *J* = 3.8 Hz), 124.41, 123.62, 118.50, 54.04. ^{19}F NMR (376 MHz, $CDCl_3$) δ -62.59. **HRMS (ESI)** m/z: $[M+H]^+$ Calcd For $C_{15}H_{13}F_3N$: 264.1000; Found: 264.0996.

Analysis of Stereochemistry:

Enantiomeric excess: 77%, determined by HPLC (Agela Technologies Venusil CJ hexane/isopropanol = 99/1, flow rate 1.0 mL/min, T = 25 °C, 220nm): t_R= 14.03 min (major), t_R= 15.33 min (minor).

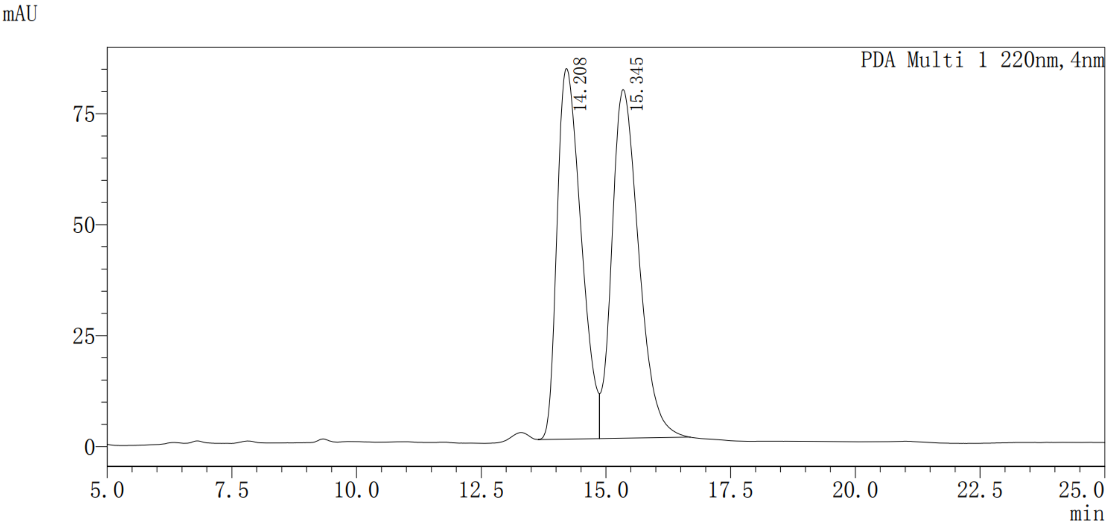

Peak	Ret Time[min]	Height [mAU]	Area[mAU*s]	Area %
1	14. 208	83506	2802149	48. 612
2	15. 345	78541	2962177	51. 388
Total		162047	5764326	100. 000

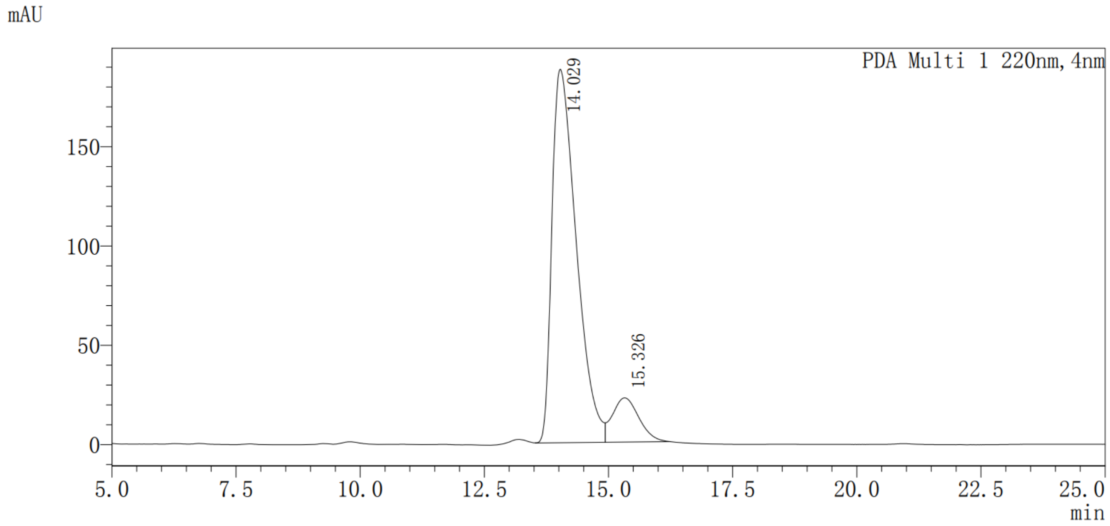

Peak	Ret Time[min]	Height [mAU]	Area[mAU*s]	Area %
1	14.029	188026	6423054	88.548
2	15.326	22298	830728	11.452
Total		210325	7253782	100.000

Supplementary Figure 46. **HPLC chromatography for 3v**

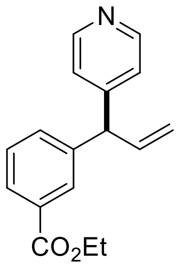

ethyl (*R*)-3-(1-(pyridin-4-yl)allyl)benzoate (3w)

Following the general procedure, the product **3x** was isolated by chromatography on silica gel (PE/EA = 10/1 ~ 5/1, eluent) as a yellow oil (45%, 24.0 mg); $[\alpha]_D^{25}$ = 18.7 (c 1.1, $CHCl_3$); ^{1}H NMR (400 MHz, $CDCl_3$) δ 8.55 (d, *J* = 5.7 Hz, 2H), 7.97 (d, *J* = 7.6 Hz, 1H), 7.90 (s, 1H), 7.43 (t, *J* = 7.7 Hz, 1H), 7.36 (d, *J* = 7.7 Hz, 1H), 7.13 (d, *J* = 6.0 Hz, 2H), 6.28 (s, 1H), 5.35 (d, *J* = 10.2 Hz, 1H), 5.07 (d, *J* = 17.1 Hz, 1H), 4.78 (d, *J* = 7.2 Hz, 1H), 4.39 (q, *J* = 7.1 Hz, 2H), 1.41 (t, *J* = 7.1 Hz, 3H). ^{13}C NMR (100 MHz, $CDCl_3$) δ 166.39, 151.61, 149.89, 141.87, 138.35, 132.93, 131.08, 129.63, 128.75, 128.22, 123.78, 118.13, 61.09, 54.11, 14.32. **HRMS (ESI)** m/z: $[M+H]^+$ Calcd For $C_{17}H_{18}NO_2$: 268.1338; Found: 268.1339.

Analysis of Stereochemistry:

Enantiomeric excess: 77%, determined by HPLC (Agela Technologies Venusil CJ hexane/isopropanol = 95/5, flow rate 1.0 mL/min, T = 25 °C, 220nm): t_R= 20.15 min (major), t_R= 25.53 min (minor).

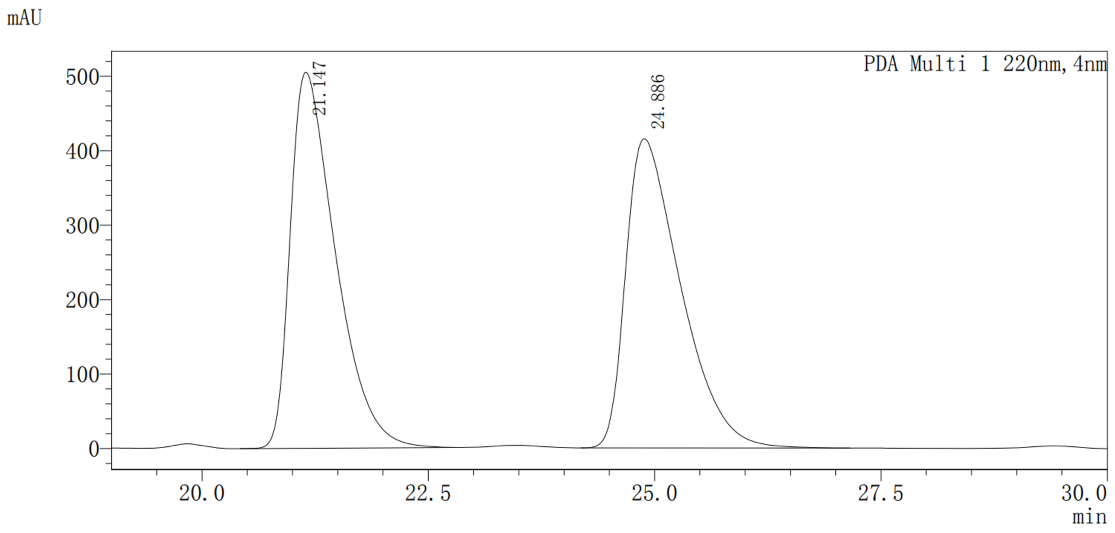

Peak	Ret Time[min]	Height [mAU]	Area[mAU*s]	Area %
1	21.147	505019	17572135	49.981
2	24.886	415326	17585531	50.019
Total		920345	35157666	100.000

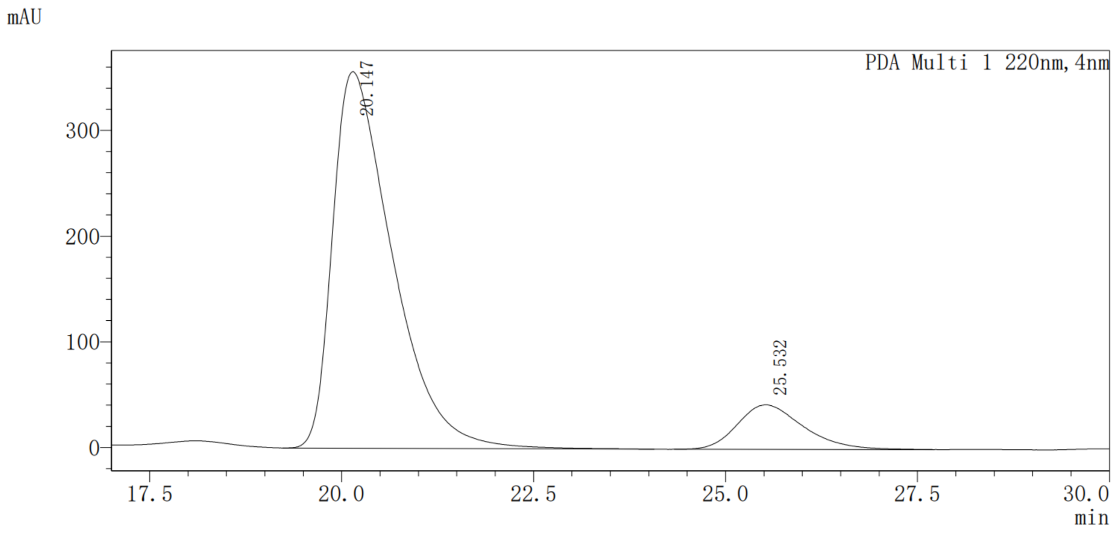

Peak	Ret Time[min]	Height [mAU]	Area[mAU*s]	Area %
1	20.147	356414	19353184	88.470
2	25.532	42145	2522147	11.530
Total		398559	21875332	100.000

Supplementary Figure 47. **HPLC chromatography for 3w**

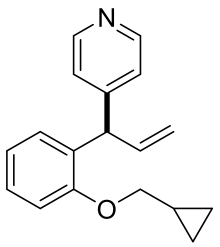

***(R)-4-(1-(2-(cyclopropylmethoxy)phenyl)allyl)pyridine* (3x)**

Following the general procedure, the product **3x** was isolated by chromatography on silica gel (PE/EA = 10/1 ~ 5/1, eluent) as a yellow oil (52%, 27.6 mg); $[\alpha]_D^{25}$ = 16.4 (c 0.9, $CHCl_3$); ^1H NMR

(400 MHz, $CDCl_3$) δ 8.49 (d, *J* = 5.7 Hz, 2H), 7.23 (dd, *J* = 13.6, 7.2 Hz, 2H), 7.14 (d, *J* = 5.5 Hz, 2H), 6.96 (t, *J* = 7.4 Hz, 1H), 6.83 (d, *J* = 8.1 Hz, 1H), 6.31 (ddd, *J* = 17.2, 10.1, 7.1 Hz, 1H), 5.29 (d, *J* = 10.2 Hz, 1H), 5.06 (dd, *J* = 33.0, 12.0 Hz, 2H), 3.73 (ddd, *J* = 32.0, 9.8, 7.0 Hz, 2H), 1.18 - 1.02 (m, 1H), 0.54 (d, *J* = 7.9 Hz, 2H), 0.19 (dd, *J* = 7.1, 5.5 Hz, 2H). ^{13}C NMR (100 MHz, $CDCl_3$) δ 156.23, 152.43, 149.44, 138.61, 130.40, 129.00, 128.08, 123.84, 120.44, 117.17, 111.82, 72.48, 48.13, 10.12, 2.91. **HRMS (ESI)** m/z: $[M+H]^+$ Calcd For $C_{18}H_{20}NO$: 266.1545; Found: 266.1542.

Analysis of Stereochemistry:

Enantiomeric excess: 92%, determined by HPLC (Daicel Chiralpak OZH hexane/isopropanol = 98/2, flow rate 1.0 mL/min, T = 25 °C, 220nm): t_R= 28.09 min (major), t_R= 36.66 min (minor).

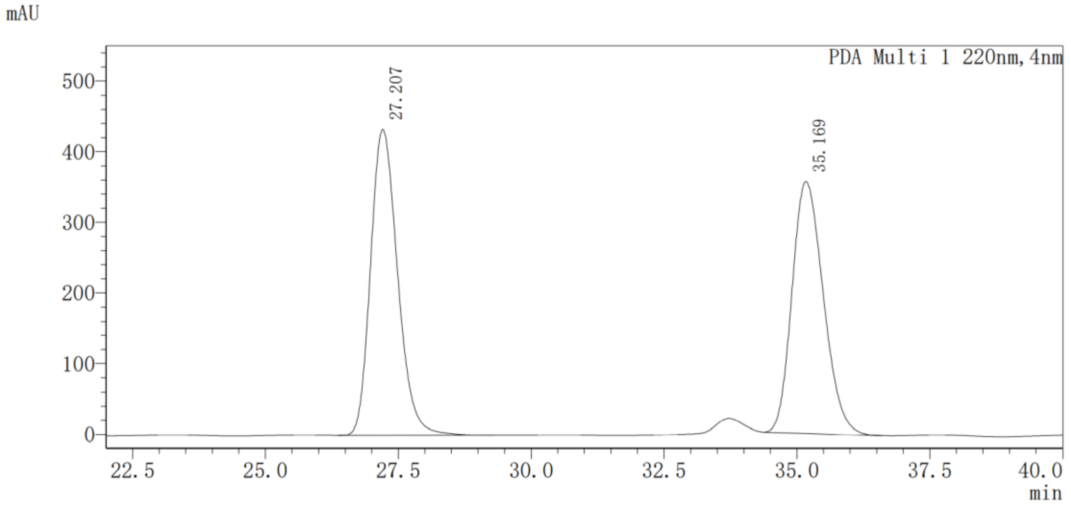

Peak	Ret Time[min]	Area[mAU*s]	Height [mAU]	Area %
1	27.207	15368753	433407	51.209
2	35.169	14643350	356627	48.791
Total		30012104	790034	100.000

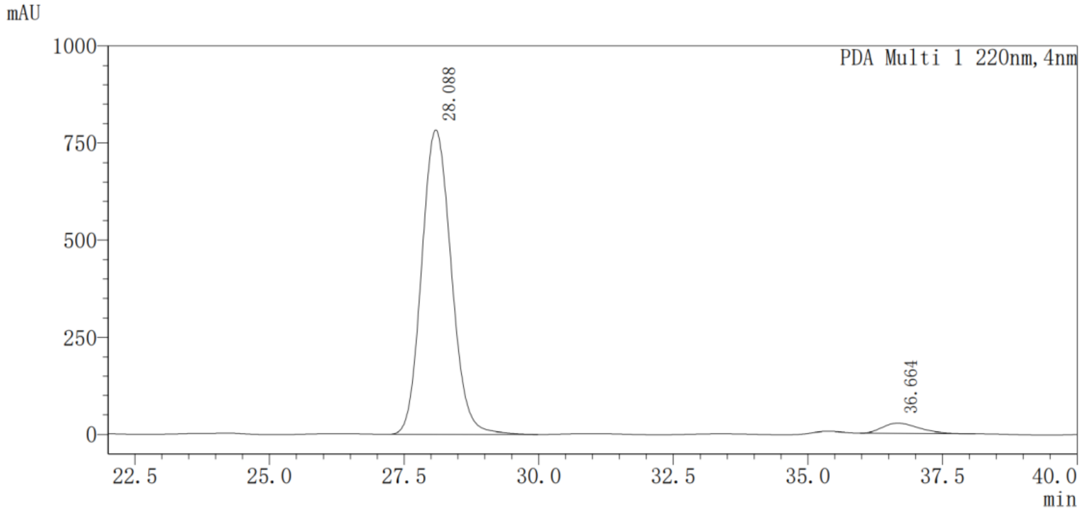

Peak	Ret Time[min]	Area[mAU*s]	Height [mAU]	Area %
1	28. 088	29945253	783261	96. 212
2	36. 664	1179043	25506	3. 788
Total		31124296	808767	100. 000

Supplementary Figure 48**. HPLC chromatography for 3x**

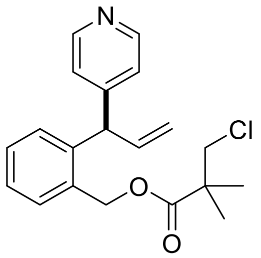

(*R*)-2-(1-(pyridin-4-yl)allyl)benzyl 3-chloro-2,2-dimethylpropanoate (3y)

Following the general procedure, the product **3y** was isolated by chromatography on silica gel (PE/EA = 5/1 ~ 3/1, eluent) as a yellow oil (45%, 31.0 mg); $[\alpha]_D^{25}$ = 25.3 (c 0.7, $CHCl_3$); ^{1}H NMR (400 MHz, $CDCl_3$) δ 8.51 (d, *J* = 5.8 Hz, 2H), 7.40 (dd, *J* = 7.3, 1.5 Hz, 1H), 7.34 (dd, *J* = 7.5, 1.7 Hz, 1H), 7.30 (dd, *J* = 7.4, 1.5 Hz, 1H), 7.14 (dd, *J* = 7.6, 1.3 Hz, 1H), 7.08 (d, *J* = 5.9 Hz, 2H), 6.22 (ddd, *J* = 17.0, 10.2, 6.6 Hz, 1H), 5.34 – 5.29 (m, 1H), 5.11 (q, *J* = 12.6 Hz, 2H), 5.04 (d, *J* = 6.6 Hz, 1H), 4.92 (dt, *J* = 17.2, 1.3 Hz, 1H), 3.59 – 3.50 (m, 2H), 1.25 (d, *J* = 1.4 Hz, 6H). ^{13}C NMR (101 MHz, $CDCl_3$) δ 174.76, 151.42, 149.87, 140.19, 138.55, 133.73, 130.42, 129.15, 129.00, 127.31, 123.98, 118.04, 64.65, 51.96, 49.49, 44.78, 23.23, 23.22. **HRMS (ESI)** m/z: $[M+H]^+$ Calcd For $C_{18}H_{20}NO$: 344.1417; Found: 344.1412.

Analysis of Stereochemistry:

Enantiomeric excess: 85%, determined by HPLC (Daicel Chiralpak ADH, hexane/isopropanol = 95/5, flow rate 1.0 mL/min, T = 25 °C, 220nm): t_R= 13.07 min (minor), t_R= 14.83 min (major).

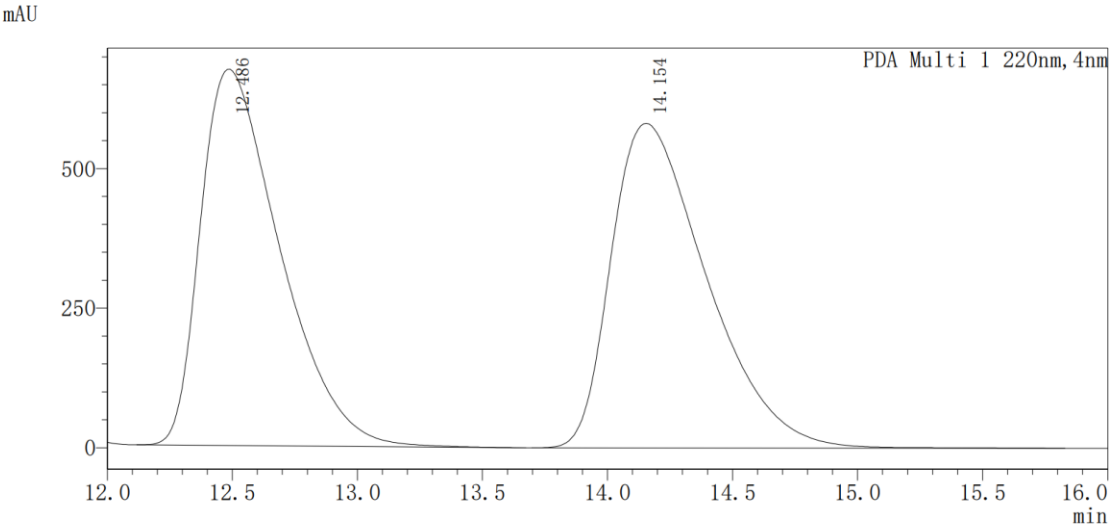

Peak	Ret Time[min]	Area[mAU*s]	Height [mAU]	Area %

1	12.486	14921410	673928	49.149
2	14.154	15438408	581438	50.851
Total		30359818	1255366	100.000

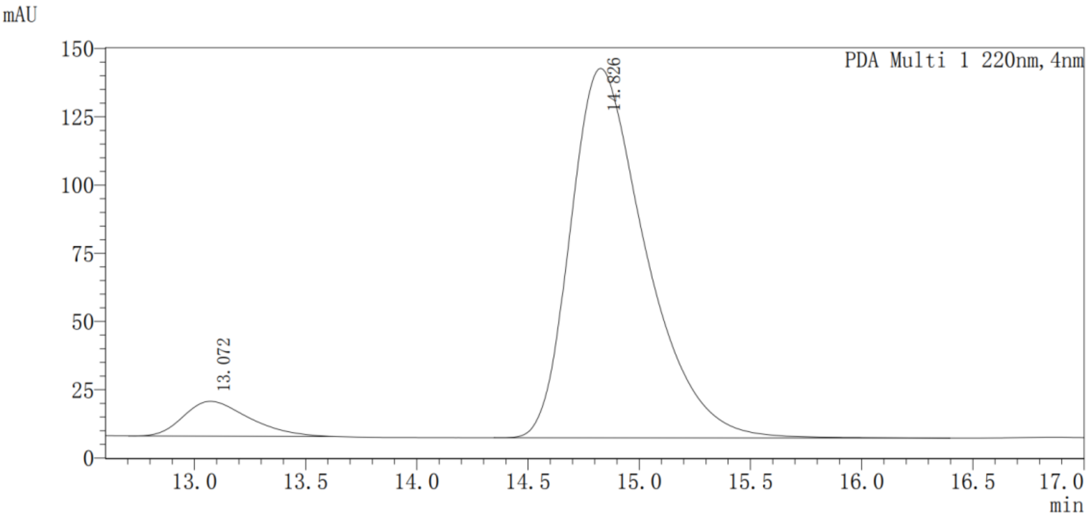

Peak	Ret Time[min]	Area[mAU*s]	Height [mAU]	Area %
1	13.072	262283	12772	7.585
2	14.826	3195518	135404	92.415
Total		3457801	148176	100.000

Supplementary Figure 49**. HPLC chromatography for 3y**

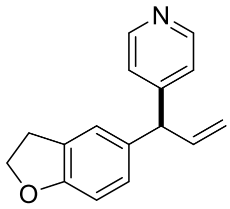

(R)-4-(1-(2,3-dihydrobenzofuran-5-yl)allyl)pyridine **(3z)**

Following the general procedure, the product **3z** was isolated by chromatography on silica gel (PE/EA = 10/1 ~ 5/1, eluent) as a yellow oil (78%, 37.3 mg); $[\alpha]_D^{25}$ = 18.2 (c 0.7, $CHCl_3$); ^{1}H NMR (400 MHz, $CDCl_3$) δ 8.53 (dd, *J* = 4.5, 1.6 Hz, 2H), 7.18 (dd, *J* = 4.7, 1.1 Hz, 2H), 7.13 (d, *J* = 5.2 Hz, 2H) 6.87 - 6.75 (m, 2H), 6.68 (dd, *J* = 7.8, 1.0 Hz, 1H), 6.28 (ddd, *J* = 17.2, 10.2, 7.3 Hz, 1H), 5.93 (dd, *J* = 2.6, 1.4 Hz, 2H), 5.32 (dt, *J* = 10.2, 1.2 Hz, 1H), 5.11 (dt, *J* = 17.1, 1.3 Hz, 1H), 4.83 (d, *J* = 7.3 Hz, 1H). ^{13}C NMR (100 MHz, $CDCl_3$) δ 151.15, 149.60, 147.50, 145.22, 137.21, 123.59, 123.00, 121.85, 121.48, 117.74, 107.53, 100.75, 48.60. **HRMS (ESI)** m/z: $[M+H]^+$ Calcd For $C_{16}H_{16}NO$: 238.1232; Found:238.1236.

Analysis of Stereochemistry:

Enantiomeric excess: 85%, determined by HPLC (Daicel Chiralpak OZH hexane/isopropanol = 98/2, flow rate 1.0 mL/min, T = 25 °C, 220nm): t_R= 33.90 min (major), t_R= 35.46 min (minor).

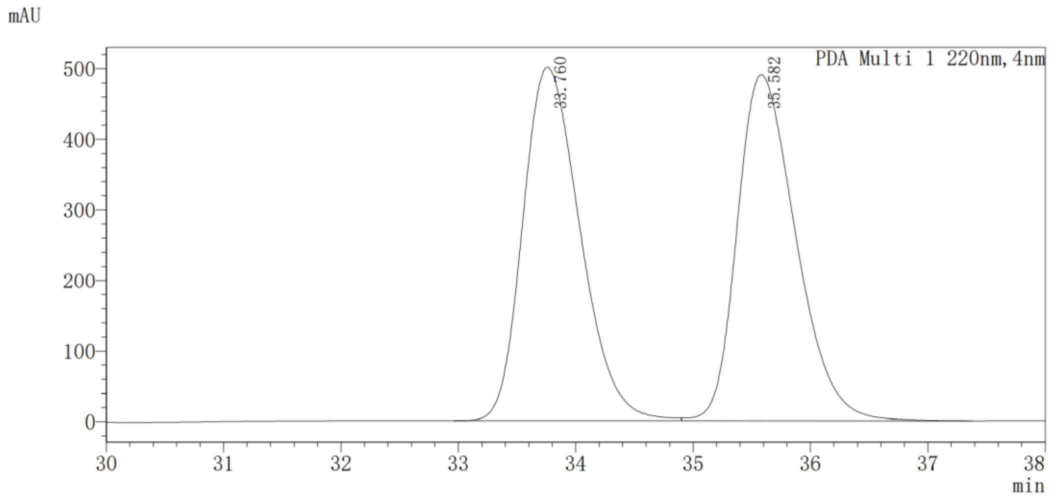

Peak	Ret Time[min]	Area[mAU*s]	Height [mAU]	Area %
1	33. 760	16997504	500582	50. 002
2	35. 582	16996423	490265	49. 998
Total		33993927	990847	100. 000

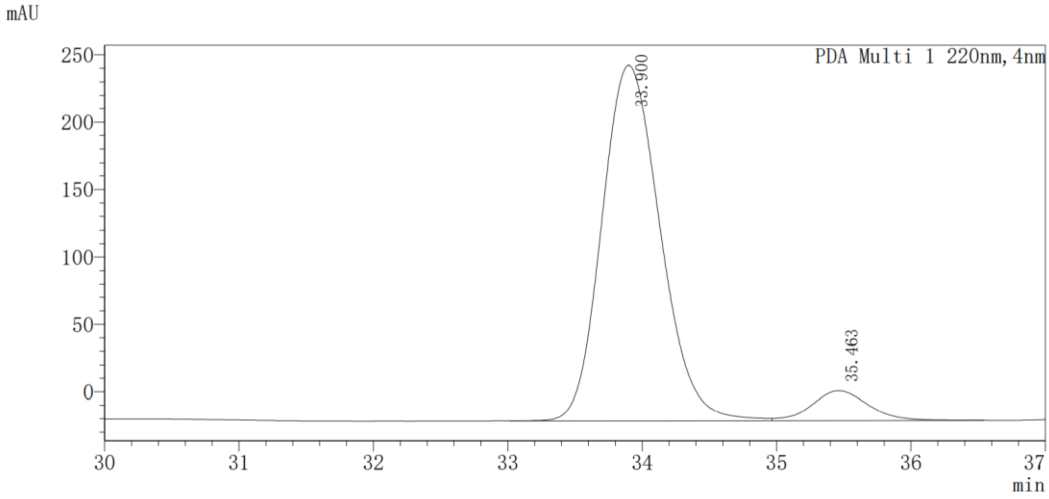

Peak	Ret Time[min]	Area[mAU*s]	Height [mAU]	Area %
1	33. 900	7956876	263853	92. 522
2	35. 463	643066	22177	7. 478
Total		8599942	286030	100. 000

Supplementary Figure 50. **HPLC chromatography for 3z**

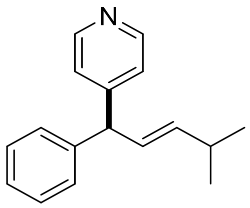

(R, E)-4-(4-methyl-1-phenylpent-2-en-1-yl)pyridine **(3aa)**

Following the general procedure, the product **3aa** was isolated by chromatography on silica gel (PE/EA = 10/1 ~ 5/1, eluent) as a pale yellow oil (62%, 29.4 mg); $[\alpha]_D^{25}$ = 23.3 (c 1.3, $CHCl_3$); ^1H NMR (400 MHz, $CDCl_3$) δ 8.52 (d, *J* = 5.2 Hz, 2H), 7.34 (t, *J* = 7.4 Hz, 2H), 7.27 (d, *J* = 8.0 Hz,

1H), 7.15 (dd, J = 22.8, 6.4 Hz, 4 H), 5.81 (dd, J = 15.6, 7.6 Hz, 1H), 5.48 (dd, J = 15.2, 6.8 Hz, 1H), 4.65 (d, J = 8.0 Hz, 1H), 2.37 (dd, J = 13.6, 6.8 Hz, 1H), 1.03 (d, J = 6.8 Hz, 6H). ^{13}C NMR (100 MHz, $CDCl_3$) δ 153.16, 149.70, 142.48, 140.73, 128.55, 128.41, 127.65, 126.66, 123.76, 53.21, 31.05, 22.36. **HRMS (ESI)** m/z: $[M+Na]^+$ Calcd For $C_{17}H_{19}NNa$: 260.1415; Found:260.1417.

Analysis of Stereochemistry:

Enantiomeric excess: 90%, determined by HPLC (Agela Technologies Venusil CJ hexane/isopropanol = 99/1, flow rate 1.0 mL/min, T = 25 °C, 220nm): t_R= 9.13 min (minor), t_R= 10.45 min (major).

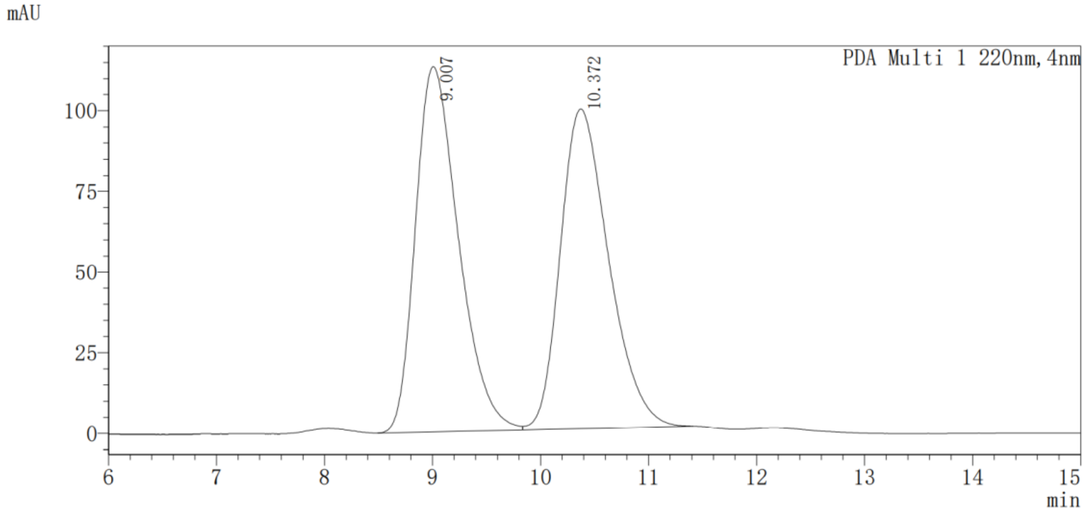

Peak	Ret Time[min]	Area[mAU*s]	Height [mAU]	Area %
1	9.007	3141800	113225	50.491
2	10.372	3080732	99074	49.509
Total		6222532	212299	100.000

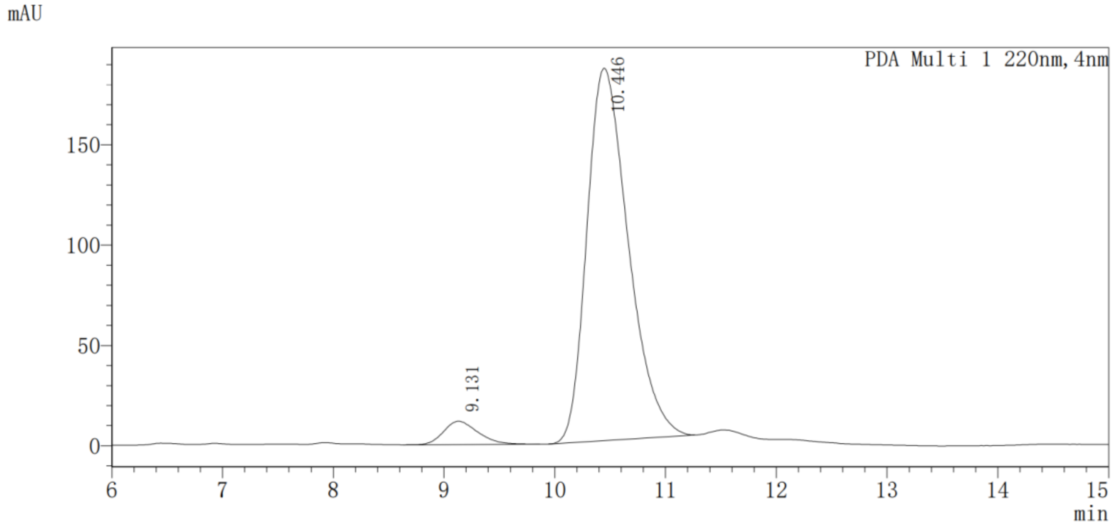

Peak	Ret Time[min]	Area[mAU*s]	Height [mAU]	Area %
1	9.131	253459	11659	4.985
2	10.446	4830577	185608	95.015
Total		5084037	197267	100.000

Supplementary Figure 51**. HPLC chromatography for 3aa**

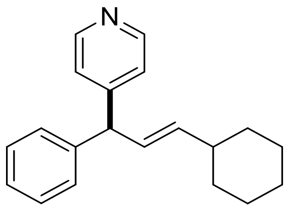

(R, E)-4-(3-cyclohexyl-1-phenylallyl)pyridine **(3ab)**

Following the general procedure, the product **3ab** was isolated by chromatography on silica gel (PE/EA = 10/1 ~ 5/1, eluent) as a yellow oil (66%, 36.6 mg); $[\alpha]_D^{25}$ = 12.7 (c 1.3, $CHCl_3$); ^{1}H NMR (400 MHz, $CDCl_3$) δ 8.62 (s, 2H), 7.33 (t, *J* = 7.3 Hz, 2H), 7.26 (dd, *J* = 11.7, 4.3 Hz, 1H), 7.17 (d, *J* = 7.2 Hz, 3H), 5.81 (ddd, *J* = 15.4, 7.7, 1.1 Hz, 1H), 5.46 (dd, *J* = 15.4, 6.7 Hz, 1H), 4.63 (d, *J* = 7.7 Hz, 1H), 2.04 (ddd, *J* = 14.4, 8.7, 4.9 Hz, 2H), 1.82 - 1.70 (m, 4H), 1.67 (d, *J* = 12.2 Hz, 1H), 1.29 (dd, *J* = 24.2, 11.5 Hz, 2H), 1.22 - 1.06 (m, 3H). ^{13}C NMR (100 MHz, $CDCl_3$) δ 153.17, 149.64, 142.56, 139.62, 128.57, 128.45, 128.18, 126.67, 53.42, 40.66, 32.95, 26.13, 26.00. **HRMS (ESI)** m/z: $[M+Na]^+$ Calcd For $C_{20}H_{23}NNa$: 300.1728; Found:300.1726.

Analysis of Stereochemistry:

Enantiomeric excess: 90%, determined by HPLC (Daicel Chiralpak ADH hexane/isopropanol = 99/1, flow rate 1.0 mL/min, T = 25 °C, 220nm): t_R= 19.08 min (minor), t_R= 23.07 min (major).

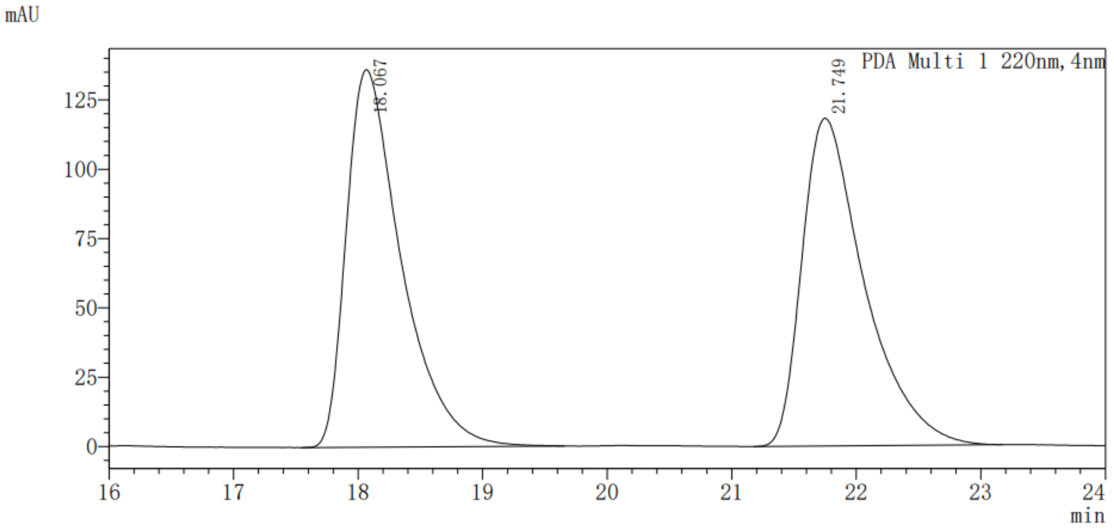

Peak	Ret Time[min]	Area[mAU*s]	Height [mAU]	Area %
1	18.067	136133	4181280	50.319
2	21.749	118215	4128314	49.681
Total		254348	8309594	100.000

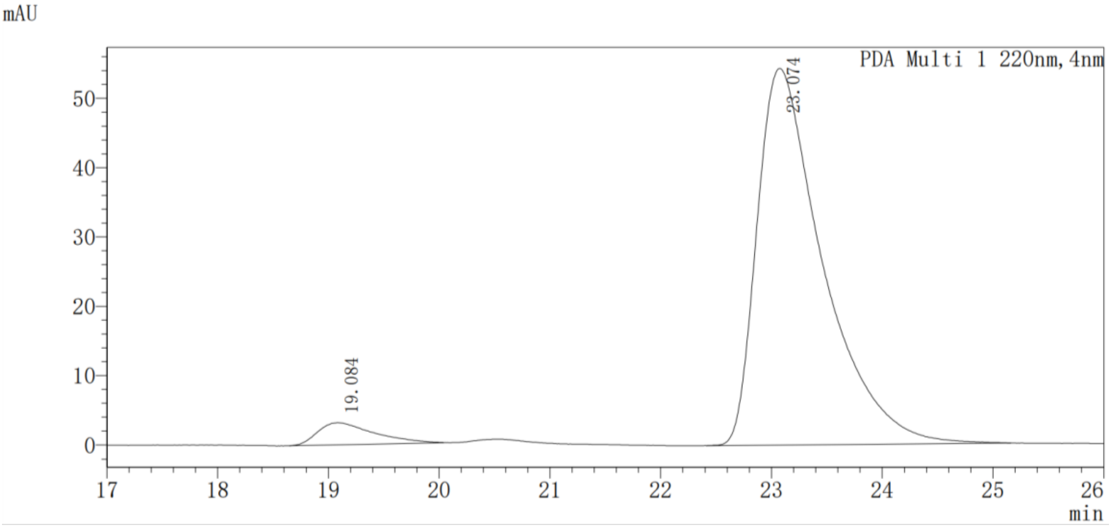

Peak	Ret Time[min]	Area[mAU*s]	Height [mAU]	Area %
1	19. 084	3198	112361	4. 782
2	23. 074	54338	2237178	95. 218
Total		57535	2349539	100. 000

Supplementary Figure 52**. HPLC chromatography for 3ab**

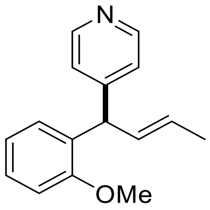

(R, E)-4-(1-(2-methoxyphenyl)but-2-en-1-yl)pyridine **(3ac)**

Following the general procedure, the product **3ac** was isolated by chromatography on silica gel (PE/EA = 10/1 ~ 5/1, eluent) as a yellow oil (71%, 33.9 mg); $[\alpha]_D^{25}$ = 33.5 (c 0.9, $CHCl_3$); ^{1}H NMR (400 MHz, $CDCl_3$) δ 8.47 (d, *J* = 5.7 Hz, 2H), 7.25 (dd, *J* = 7.7, 1.3 Hz, 1H), 7.18 (dd, *J* = 7.5, 1.6 Hz, 1H), 7.10 (d, *J* = 6.0 Hz, 2H), 6.97 (td, *J* = 7.5, 0.9 Hz, 1H), 6.89 (d, *J* = 8.2 Hz, 1H), 5.89 (ddd, *J* = 15.2, 7.6, 1.6 Hz, 1H), 5.46 (dqd, *J* = 12.9, 6.4, 1.1 Hz, 1H), 5.03 (d, *J* = 7.5 Hz, 1H), 3.74 (s, 3H), 1.76 (d, *J* = 6.5 Hz, 3H). ^{13}C NMR (100 MHz, $CDCl_3$) δ 156.83, 153.45, 149.38, 131.39, 130.97, 129.08, 128.00, 127.98, 123.67, 120.61, 110.94, 55.40, 46.69, 17.97. **HRMS (ESI)** m/z: $[M+H]^+$ Calcd For $C_{16}H_{18}NO$: 240.1388; Found:240.1385.

Analysis of Stereochemistry:

Enantiomeric excess: 90%, determined by HPLC (Daicel Chiralpak OZH hexane/isopropanol = 98/2, flow rate 1.0 mL/min, T = 25 °C, 220nm): t_R= 19.38 min (major), t_R= 22.22 min (minor).

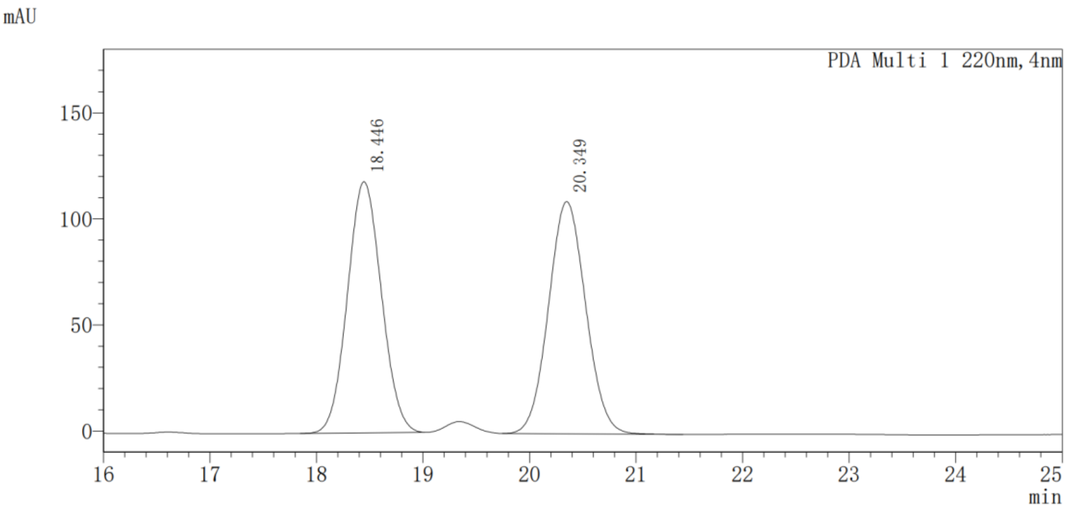

Peak	Ret Time[min]	Area[mAU*s]	Height [mAU]	Area %
1	18. 446	2631080	118446	49. 698
2	20. 349	2663027	109507	50. 302
Total		5294106	227954	100. 000

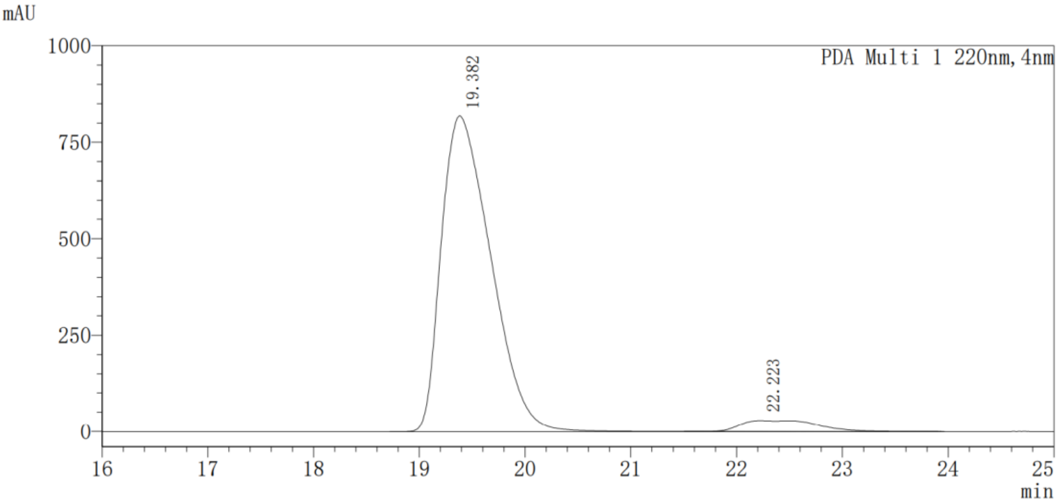

Peak	Ret Time[min]	Area[mAU*s]	Height [mAU]	Area %
1	19. 382	26672461	819529	94. 835
2	22. 223	1452772	28156	5. 165
Total		28125233	847685	100. 000

Supplementary Figure 53**. HPLC chromatography for 3ac**

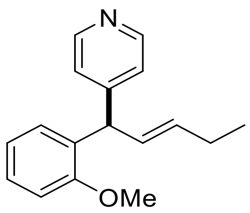

(R, E)-4-(1-(2-methoxyphenyl)pent-2-en-1-yl)pyridine **(3ad)**

Following the general procedure, the product **3ac** was isolated by chromatography on silica gel (PE/EA = 10/1 ~ 5/1, eluent) as a yellow oil (77%, 38.9 mg); $[\alpha]_D^{25}$ = 15.4 (c 1.5, $CHCl_3$); ^{1}H NMR

(400 MHz, $CDCl_3$) δ 8.45 (d, J = 4.8 Hz, 2H), 7.23 (d, J = 7.7 Hz, 1H), 7.17 (d, J = 7.1 Hz, 1H), 7.08 (d, J = 5.0 Hz, 2H), 6.95 (t, J = 7.4 Hz, 1H), 6.86 (d, J = 8.1 Hz, 1H), 5.84 (dd, J = 15.4, 7.5 Hz, 1H), 5.52 - 5.43 (m, 1H), 5.01 (d, J = 7.3 Hz, 1H), 3.71 (s, 3H), 2.14 - 2.04 (m, 2H), 1.00 (t, J = 7.4 Hz, 3H). ^{13}C NMR (100 MHz, $CDCl_3$) δ 156.80, 153.38, 149.40, 134.98, 130.98, 129.19, 129.01, 127.91, 123.64, 120.55, 110.87, 55.34, 46.52, 25.55, 13.62. **HRMS (ESI)** m/z: $[M+H]^+$ Calcd For $C_{17}H_{20}NO$: 254.1545; Found:254.1549.

Analysis of Stereochemistry:

Enantiomeric excess: 93%, determined by HPLC (Daicel Chiralpak ADH hexane/isopropanol = 99/1, flow rate 1.0 mL/min, T = 25 °C, 220nm): t_R= 18.66 min (major), t_R= 19.35 min (minor).

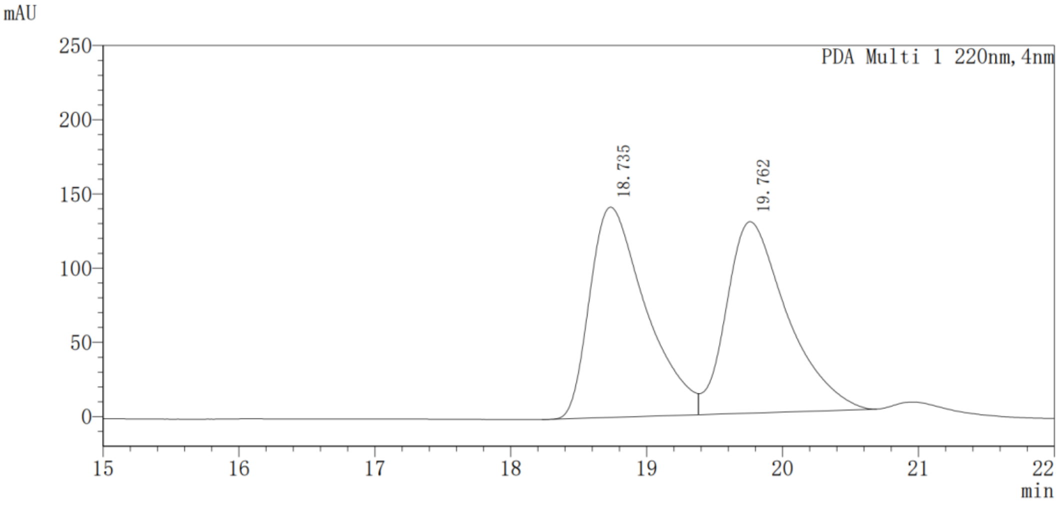

Peak	Ret Time[min]	Area[mAU*s]	Height [mAU]	Area %
1	18. 735	4064414	141596	50. 304
2	19. 762	4015346	128846	49. 696
Total		8079759	270442	100. 000

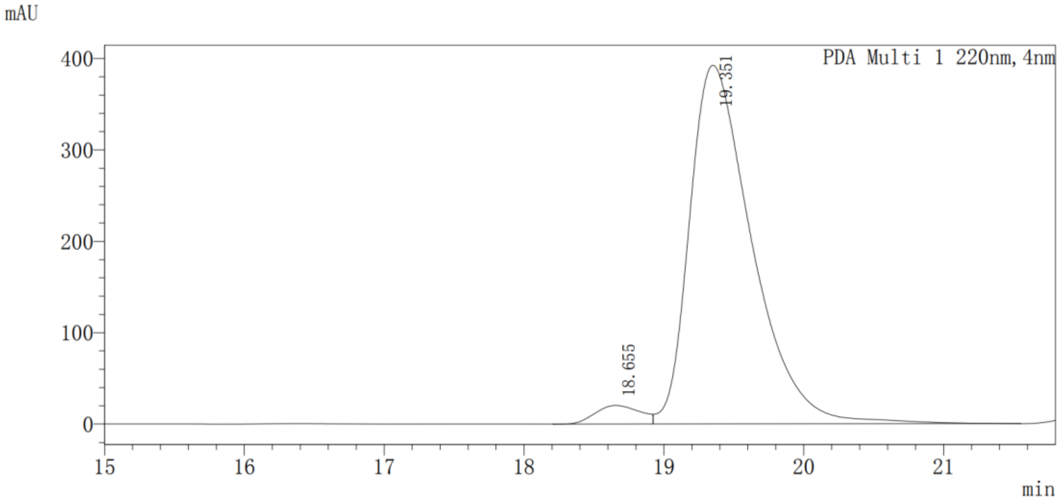

Peak	Ret Time[min]	Area[mAU*s]	Height [mAU]	Area %
1	18. 655	458406	20313	3. 624
2	19. 351	12192445	392528	96. 376
Total		12650852	412841	100. 000

Supplementary Figure 54. HPLC chromatography for **3ad**

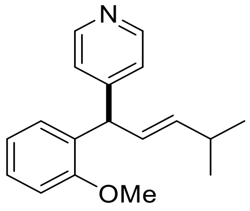

(R, E)-4-(1-(2-methoxyphenyl)-4-methylpent-2-en-1-yl)pyridine **(3ae)**

Following the general procedure, the product **3ae** was isolated by chromatography on silica gel (PE/EA = 10/1 ~ 5/1, eluent) as a yellow oil (80%, 42.7 mg); $[\alpha]_D^{25}$ = 27.0 (c 1.2, $CHCl_3$); ^1H NMR (400 MHz, $CDCl_3$) δ 8.45 (d, *J* = 6.0 Hz, 2H), 7.23 (dd, *J* = 7.7, 1.3 Hz, 1H), 7.16 (dd, *J* = 7.5, 1.4 Hz, 1H), 7.06 (d, *J* = 5.6 Hz, 2H), 6.95 (dd, *J* = 7.9, 7.0 Hz, 1H), 6.86 (d, *J* = 7.8 Hz, 1H), 5.79 (ddd, *J* = 15.4, 7.5, 1.2 Hz, 1H), 5.40 (ddd, *J* = 15.4, 6.6, 1.0 Hz, 1H), 4.99 (d, *J* = 7.4 Hz, 1H), 3.71 (s, 3H), 2.34 (td, *J* = 13.4, 6.7 Hz, 1H), 1.00 (t, *J* = 6.4 Hz, 6H). ^{13}C NMR (100 MHz, $CDCl_3$) δ 156.87, 153.50, 149.40, 140.48, 131.08, 129.00, 127.93, 127.29, 123.68, 120.58, 110.91, 55.37, 46.49, 31.09, 22.45. **HRMS (ESI)** m/z: $[M+H]^+$ Calcd For $C_{18}H_{22}NO$: 268.1701; Found:268.1700.

Analysis of Stereochemistry:

Enantiomeric excess: 89%, determined by HPLC (Daicel Chiralpak OZH hexane/isopropanol = 98/2, flow rate 1.0 mL/min, T = 25 °C, 220nm): t_R= 14.22 min (major), t_R= 16.88 min (minor).

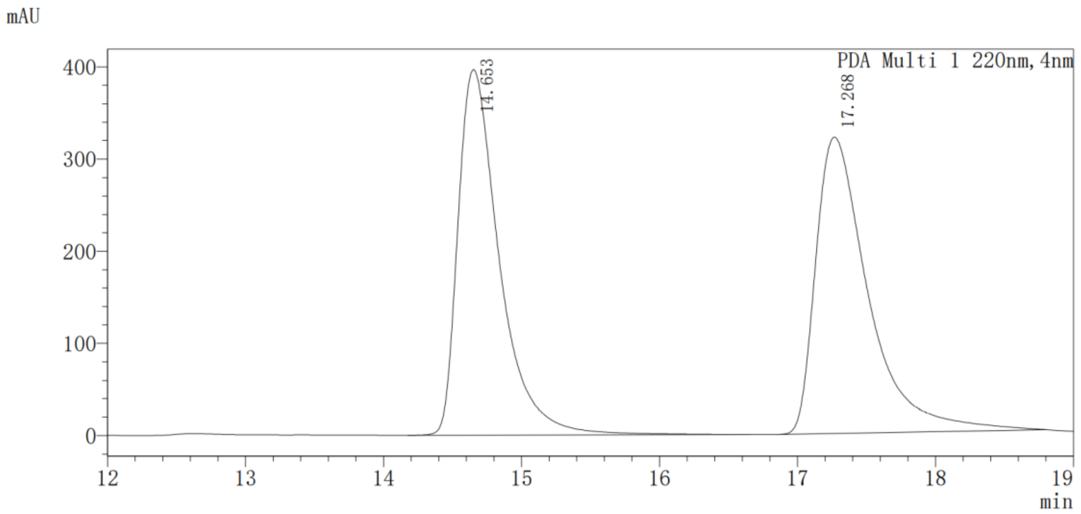

Peak	Ret Time[min]	Area[mAU*s]	Height [mAU]	Area %
1	14.653	396740	8274973	49.176
2	17.268	321675	8552159	50.824
Total		718415	16827132	100.000

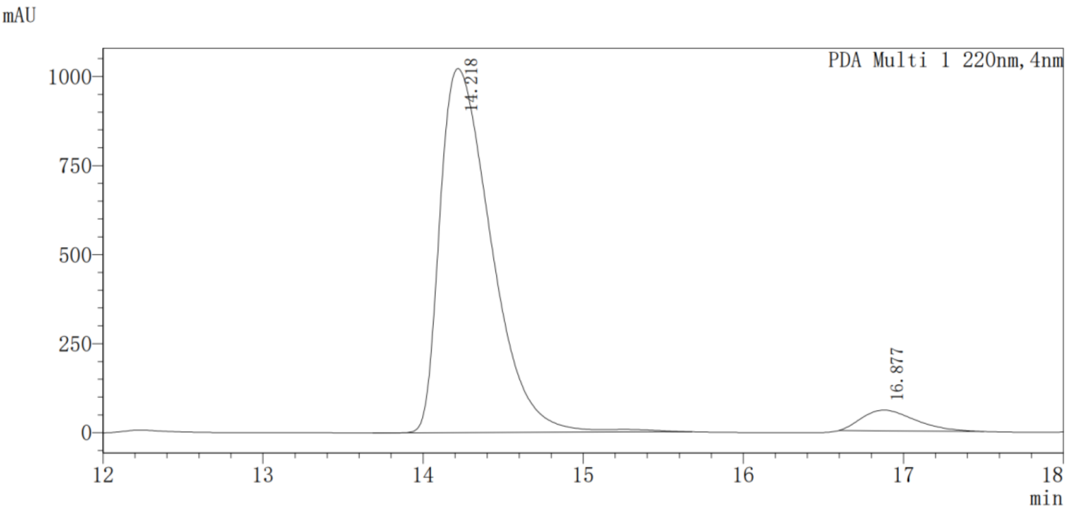

Peak	Ret Time[min]	Area[mAU*s]	Height [mAU]	Area %
1	14. 218	22749792	1021901	94. 374
2	16. 877	1356320	58088	5. 626
Total		24106112	1079989	100. 000

Supplementary Figure 55. **HPLC chromatography for 3ae**

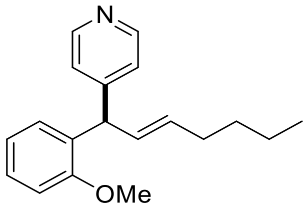

***(R, E)-4-(1-(2-methoxyphenyl)hept-2-en-1-yl)pyridine* (3af)**

Following the general procedure, the product **3af** was isolated by chromatography on silica gel (PE/EA = 10/1 ~ 5/1, eluent) as a yellow oil (64%, 36.0 mg); $[\alpha]_D^{25}$ = 28.6 (c 1.5, $CHCl_3$); ^{1}H NMR (400 MHz, $CDCl_3$) δ 8.45 (d, *J* = 5.9 Hz, 2H), 7.23 (d, *J* = 8.1 Hz, 1H), 7.19 - 7.14 (m, 1H), 7.07 (d, *J* = 5.8 Hz, 2H), 6.94 (t, *J* = 7.5 Hz, 1H), 6.86 (d, *J* = 8.1 Hz, 1H), 5.84 (dd, *J* = 15.3, 7.5 Hz, 1H), 5.47 - 5.35 (m, 1H), 5.01 (d, *J* = 7.4 Hz, 1H), 3.71 (s, 3H), 2.07 (dd, *J* = 13.6, 6.8 Hz, 2H), 1.34 (dt, *J* = 13.1, 6.1 Hz, 4H), 0.89 (t, *J* = 7.0 Hz, 3H). ^{13}C NMR (100 MHz, $CDCl_3$) δ 156.81, 153.40, 149.41, 133.52, 130.99, 130.11, 129.02, 127.91, 123.65, 120.56, 110.85, 55.33, 46.62, 32.22, 31.45, 22.19, 13.86. **HRMS (ESI)** m/z: $[M+H]^+$ Calcd For $C_{19}H_{24}NO$: 282.1858; Found:282.1859.

Analysis of Stereochemistry:

Enantiomeric excess: 93%, determined by HPLC (Daicel Chiralpak OZH hexane/isopropanol = 99/1, flow rate 1.0 mL/min, T = 25 °C, 220nm): t_R= 21.89 min (major), t_R= 26.44 min (minor).

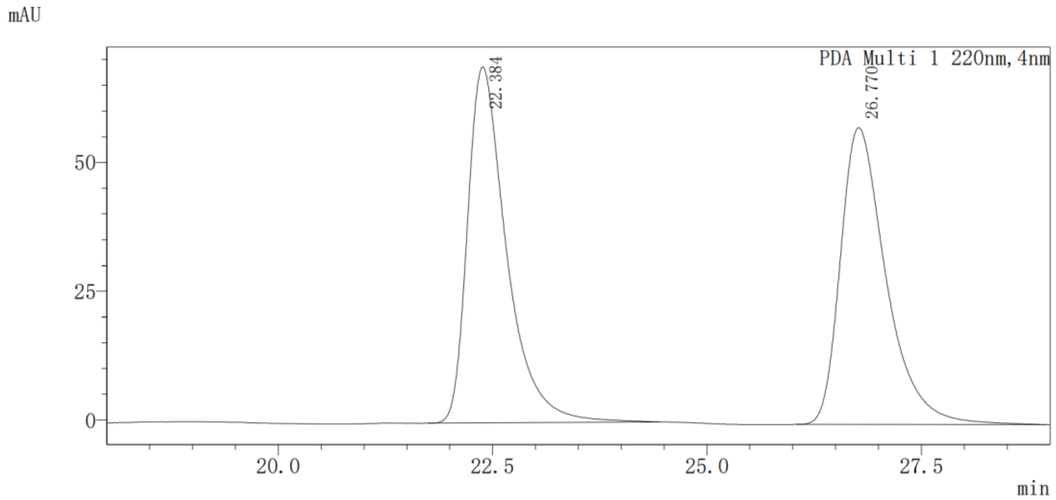

Peak	Ret Time[min]	Area[mAU*s]	Height [mAU]	Area %
1	22.384	2194478	69087	50.671
2	26.770	2136359	57607	49.329
Total		4330837	126694	100.000

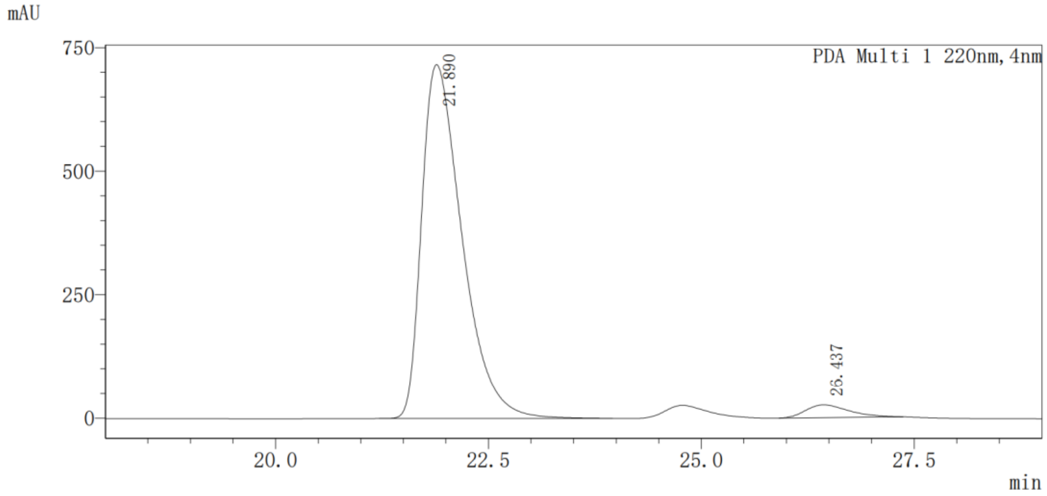

Peak	Ret Time[min]	Area[mAU*s]	Height [mAU]	Area %
1	21.890	23609489	716102	96.271
2	26.437	914450	25728	3.729
Total		24523939	741830	100.000

Supplementary Figure 56. **HPLC chromatography for 3af**

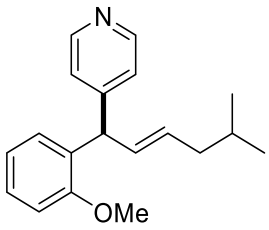

***(R, E)-4-(1-(2-methoxyphenyl)-5-methylhex-2-en-1-yl)pyridine* (3ag)**

Following the general procedure, the product **3ag** was isolated by chromatography on silica gel (PE/EA = 10/1 ~ 5/1, eluent) as a yellow oil (65%, 36.5 mg); $[\alpha]_D^{25}$ = 26.4 (c 0.5, $CHCl_3$); ^1H NMR (400 MHz, $CDCl_3$) δ 8.48 (d, *J* = 6.0 Hz, 2H), 7.28 - 7.23 (m, 1H), 7.19 (d, *J* = 7.5 Hz, 1H), 7.10

(d, J = 5.9 Hz, 2H), 6.97 (t, J = 7.5 Hz, 1H), 6.88 (d, J = 8.2 Hz, 1H), 5.85 (dd, J = 15.3, 7.5 Hz, 1H), 5.48 - 5.37 (m, 1H), 5.05 (d, J = 7.4 Hz, 1H), 3.74 (s, 3H), 1.99 (t, J = 6.9 Hz, 2H), 1.64 (td, J = 13.3, 6.7 Hz, 1H), 0.90 (dd, J = 6.6, 3.6 Hz, 6H). ^{13}C NMR (100 MHz, $CDCl_3$) δ 156.81, 153.40, 149.44, 149.41, 132.24, 131.29, 130.96, 129.02, 127.92, 123.67, 120.56, 110.81, 55.32, 55.30, 46.71, 41.92, 28.36, 22.29, 22.27. **HRMS (ESI)** m/z: $[M+H]^+$ Calcd For $C_{19}H_{24}NO$: 282.1858; Found:282.1855.

Analysis of Stereochemistry:

Enantiomeric excess: 87 %, determined by HPLC (Daicel Chiralpak OZH hexane/isopropanol = 98/2, flow rate 1.0 mL/min, T = 25 °C, 220nm): t_R= 13.80 min (major), t_R= 15.59 min (minor).

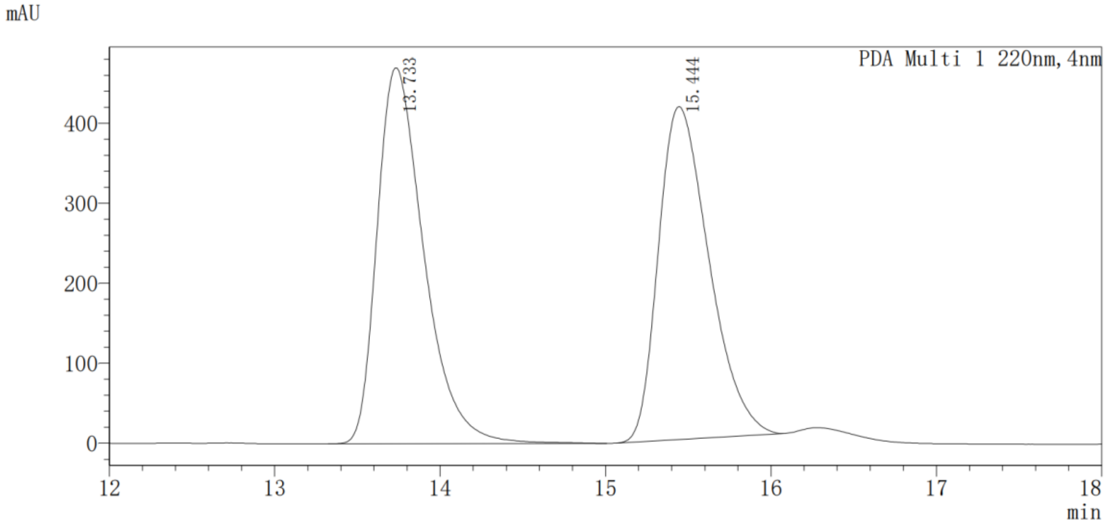

Peak	Ret Time[min]	Area[mAU*s]	Height [mAU]	Area %
1	13.733	9246897	469954	51.110
2	15.444	8845404	416430	48.890
Total		18092301	886384	100.000

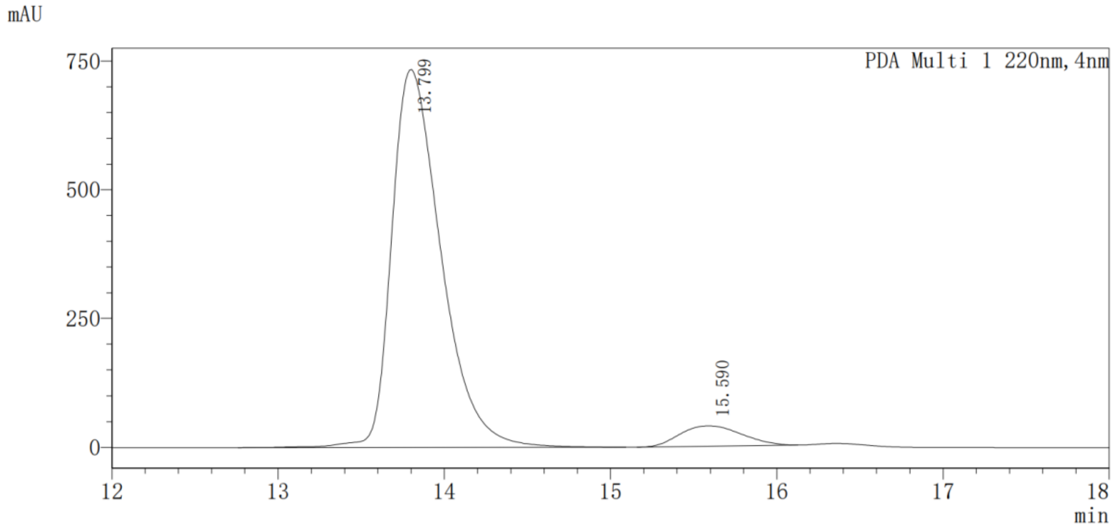

Peak	Ret Time[min]	Area[mAU*s]	Height [mAU]	Area %
1	13.799	15155565	733833	93.677
2	15.590	1022984	39687	6.323
Total		16178549	773520	100.000

Supplementary Figure 57. **HPLC chromatography for 3ag**

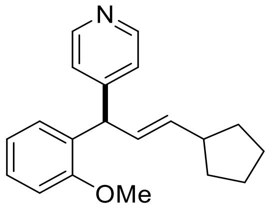

(R, E)-4-(3-cyclopentyl-1-(2-methoxyphenyl)allyl)pyridine **(3ah)**

Following the general procedure, the product **3ah** was isolated by chromatography on silica gel (PE/EA = 10/1 ~ 5/1, eluent) as a yellow oil (59%, 34.6 mg); $[\alpha]_D^{25}$ = 25.1 (c 1.0, $CHCl_3$); ^{1}H NMR (400 MHz, $CDCl_3$) δ 8.44 (s, 2H), 7.26 - 7.21 (m, 1H), 7.19 - 7.13 (m, 1H), 7.07 (d, *J* = 5.4 Hz, 2H), 6.95 (t, *J* = 7.4 Hz, 1H), 6.86 (d, *J* = 8.2 Hz, 1H), 5.81 (dd, *J* = 15.3, 7.5 Hz, 1H), 5.41 (dd, *J* = 15.3, 7.5 Hz, 1H), 4.99 (d, *J* = 7.5 Hz, 1H), 3.71 (s, 3H), 2.48 (dd, *J* = 15.9, 7.9 Hz, 1H), 1.80 - 1.71 (m, 3H), 1.58 (ddd, *J* = 11.7, 10.1, 5.0 Hz, 3H), 1.27 (dd, *J* = 12.2, 4.1 Hz, 2H). ^{13}C NMR (100 MHz, $CDCl_3$) δ 156.90, 153.59, 149.37, 138.09, 131.11, 129.05, 128.23, 127.94, 123.73, 120.62, 110.95, 55.39, 46.61, 43.20, 33.10, 33.02, 25.10. **HRMS (ESI)** m/z: $[M+H]^+$ Calcd For $C_{20}H_{24}NO$: 294.1858; Found:294.1859.

Analysis of Stereochemistry:

Enantiomeric excess: 92%, determined by HPLC (Daicel Chiralpak ADH hexane/isopropanol = 99/1, flow rate 1.0 mL/min, T = 25 °C, 220nm): t_R= 21.46 min (minor), t_R= 23.07 min (major).

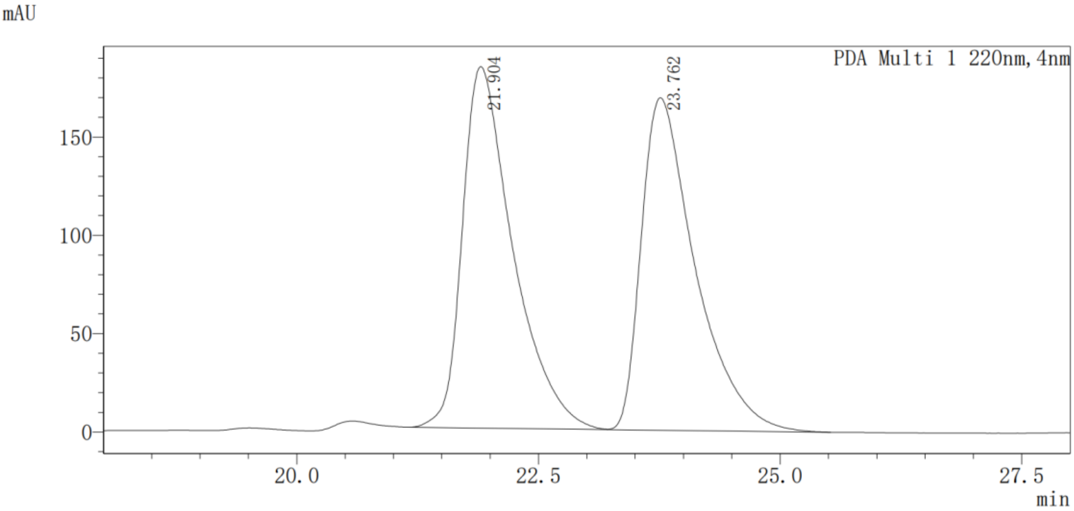

Peak Ret Time[min] Area[mAU*s] Height [mAU] Area %

1	21.904	183729	6683401	50.439
2	23.762	169019	6566978	49.561
Total		352748	13250379	100.000

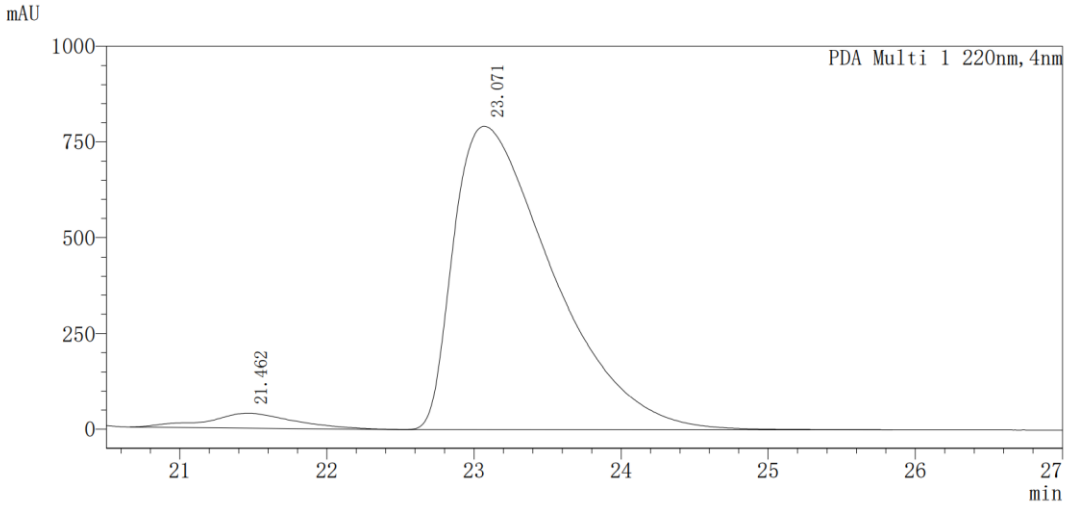

Peak	Ret Time[min]	Area[mAU*s]	Height [mAU]	Area %
1	21.462	1606968	38922	4.148
2	23.071	37132823	791687	95.852
Total		38739792	830609	100.000

Supplementary Figure 58. **HPLC chromatography for 3ah**

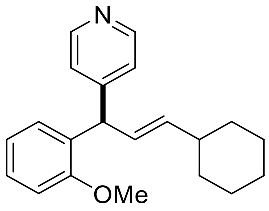

(R, E)-4-(3-cyclohexyl-1-(2-methoxyphenyl)allyl)pyridine **(3ai)**

Following the general procedure, the product **3ai** was isolated by chromatography on silica gel (PE/EA = 10/1 ~ 5/1, eluent) as a yellow oil (82%, 50.3 mg); $[\alpha]_D^{25}$ = 26.0 (c 0.9, $CHCl_3$); ^{1}H NMR (400 MHz, $CDCl_3$) δ 8.44 (d, J = 5.6 Hz, 2H), 7.26 - 7.20 (m, 1H), 7.16 (dd, J = 7.5, 1.6 Hz, 1H), 7.07 (d, J = 5.9 Hz, 2H), 6.94 (dd, J = 7.4, 6.7 Hz, 1H), 6.86 (d, J = 8.2 Hz, 1H), 5.79 (ddd, J = 15.5, 7.6, 1.1 Hz, 1H), 5.38 (dd, J = 15.5, 6.7 Hz, 1H), 4.98 (d, J = 7.5 Hz, 1H), 3.71 (s, 3H), 2.00 (dt, J = 10.6, 7.2 Hz, 1H), 1.72 - 1.61 (m, 5H), 1.29 - 1.21 (m, 2H), 1.20 - 1.04 (m, 3H). ^{13}C NMR (100 MHz, $CDCl_3$) δ 156.89, 153.69, 149.30, 139.36, 131.12, 129.03, 127.94, 127.74, 123.73, 120.61, 110.95, 46.67, 40.71, 33.03, 33.00, 26.17, 26.03. **HRMS (ESI)** m/z: $[M+H]^+$ Calcd For $C_{21}H_{26}NO$: 308.2014; Found:308.2019.

Analysis of Stereochemistry:

Enantiomeric excess: 92%, determined by HPLC (Daicel Chiralpak OZH hexane/isopropanol = 98/2, flow rate 1.0 mL/min, T = 25 °C, 220nm): t_R= 16.15 min (major), t_R= 19.13 min (minor).

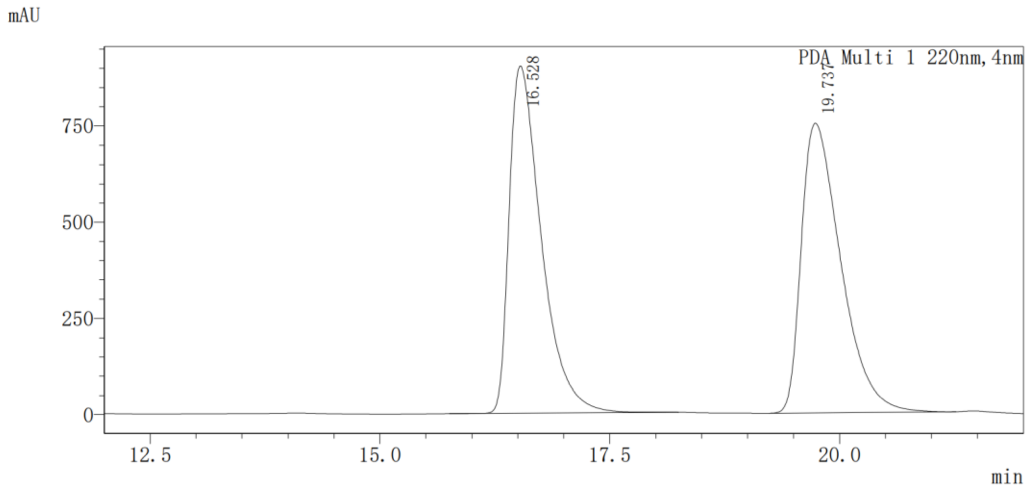

Peak	Ret Time[min]	Area[mAU*s]	Height [mAU]	Area %
1	16. 528	21981729	902312	49. 377
2	19. 737	22536803	752990	50. 623
Total		44518532	1655302	100. 000

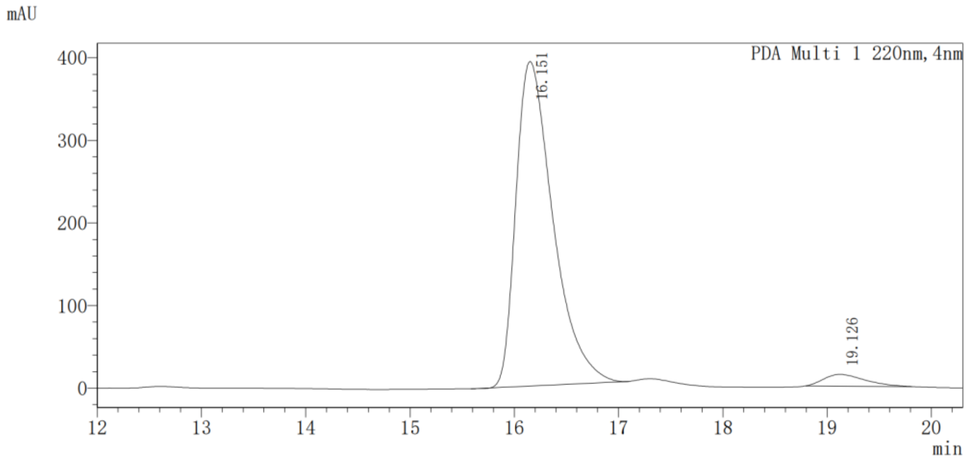

Peak	Ret Time[min]	Area[mAU*s]	Height [mAU]	Area %
1	16. 151	9716329	392835	96. 153
2	19. 126	388712	14120	3. 847
Total		10105040	406955	100. 000

Supplementary Figure 59. **HPLC chromatography for 3ai**

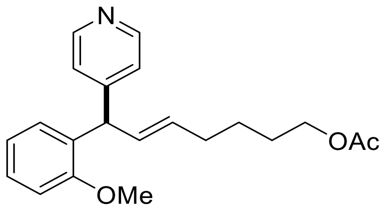

***(R, E)-7-(2-methoxyphenyl)-7-(pyridin-4-yl)hept-5-en-1-yl acetate* (3aj)**

Following the general procedure, the product **3aj** was isolated by chromatography on silica gel (PE/EA = 5/1 ~ 3/1, eluent) as a yellow oil (72%, 48.8 mg); $[\alpha]_D^{25}$ = 27.6 (c 1.2, $CHCl_3$); ^{1}H NMR (400 MHz, $CDCl_3$) δ 8.47 (d, J = 5.4 Hz, 2H), 7.25 (d, J = 7.9 Hz, 1H), 7.17 (d, J = 6.7 Hz, 1H),

7.09 (d, *J* = 5.5 Hz, 2H), 6.96 (t, *J* = 7.5 Hz, 1H), 6.88 (d, *J* = 8.1 Hz, 1H), 5.88 (dd, *J* = 15.3, 7.5 Hz, 1H), 5.43 (dd, *J* = 14.7, 7.3 Hz, 1H), 5.03 (d, *J* = 7.3 Hz, 1H), 4.07 (t, *J* = 6.6 Hz, 2H), 3.73 (s, 3H), 2.13 (dd, *J* = 14.2, 7.1 Hz, 2H), 2.05 (d, *J* = 7.2 Hz, 3H), 1.63 (dd, *J* = 14.7, 6.9 Hz, 2H), 1.49 - 1.42 (m, 2H). ^{13}C NMR (100 MHz, $CDCl_3$) δ 171.10, 156.77, 153.23, 149.38, 132.70, 130.80, 130.76, 128.97, 127.98, 123.64, 120.56, 110.85, 64.30, 55.32, 46.59, 32.06, 28.08, 25.59, 20.93. **HRMS (ESI)** m/z: $[M+H]^+$ Calcd For $C_{21}H_{26}NO_3$: 340.1913; Found:340.1910.

Analysis of Stereochemistry:

Enantiomeric excess: 85%, determined by HPLC (Daicel Chiralpak OZH hexane/isopropanol = 92/8, flow rate 1.0 mL/min, T = 25 °C, 220nm): t_R= 24.43 min (major), t_R= 27.91 min (minor).

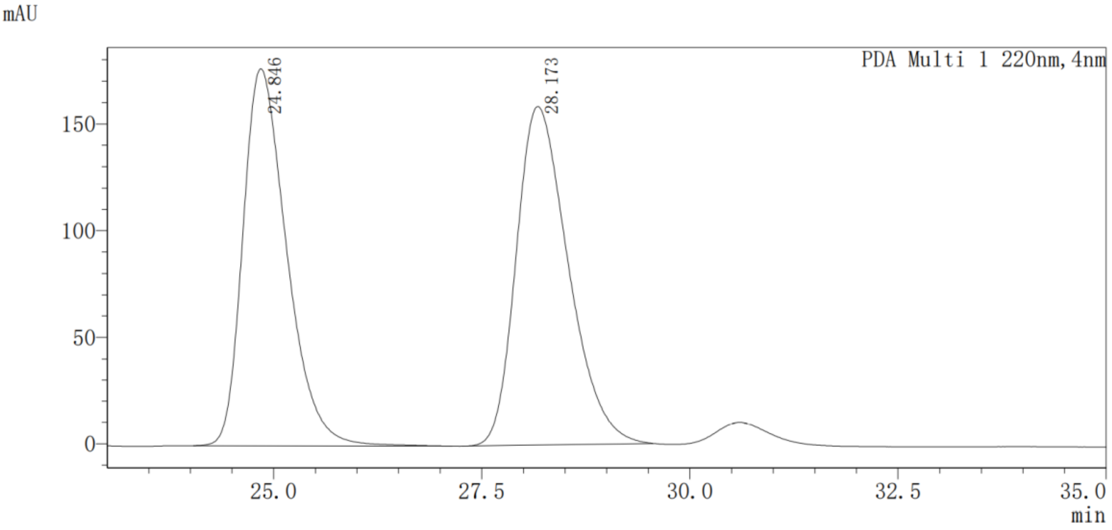

Peak	Ret Time[min]	Area[mAU*s]	Height [mAU]	Area %
1	24.846	6478959	176752	48.389
2	28.173	6910315	158832	51.611
Total		13389274	335584	100.000

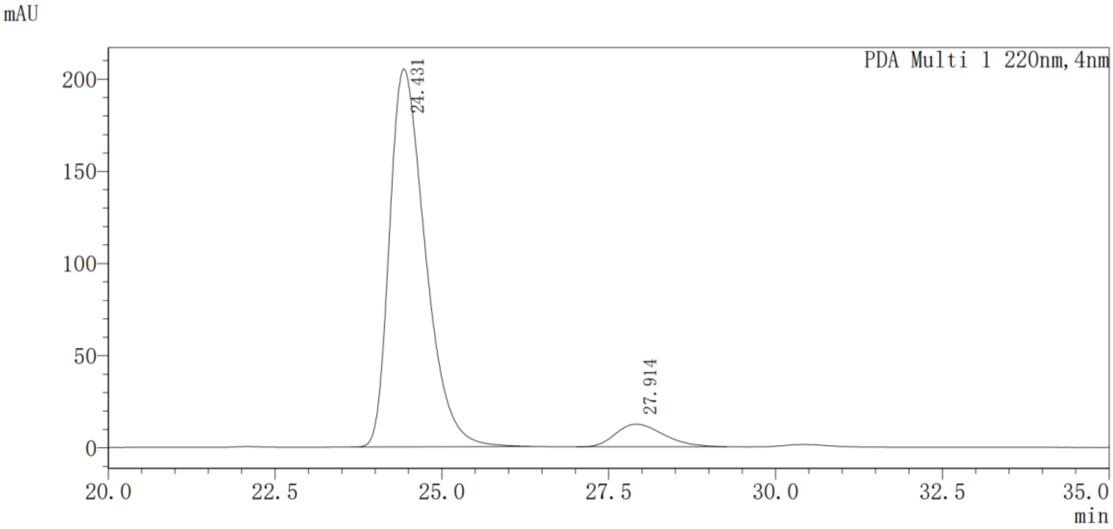

Peak	Ret Time[min]	Area[mAU*s]	Height [mAU]	Area %
1	24.431	7645731	205005	92.600
2	27.914	611009	12229	7.400
Total		8256740	217234	100.000

Supplementary Figure 60. **HPLC chromatography for 3aj**

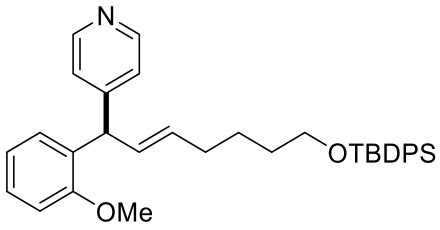

(R, E)-4-(7-((tert-butyldiphenylsilyl)oxy)-1-(2-methoxyphenyl)hept-2-en-1-yl)pyridine **(3ak)**

Following the general procedure, the product **3ak** was isolated by chromatography on silica gel (PE/EA = 10/1 ~ 5/1, eluent) as a pale yellow oil (58%, 62.1 mg); $[\alpha]_D^{25}$ = 25.8 (c 1.6, $CHCl_3$); ^1H NMR (400 MHz, $CDCl_3$) δ 8.49 (d, J = 4.9 Hz, 2H), 7.71 (d, J = 7.0 Hz, 4H), 7.42 (dt, J = 13.7, 6.9 Hz, 6H), 7.27 (d, J = 9.0 Hz, 1H), 7.20 (d, J = 7.5 Hz, 1H), 7.11 (d, J = 5.0 Hz, 2H), 6.98 (t, J = 7.5 Hz, 1H), 6.90 (d, J = 8.1 Hz, 1H), 5.87 (dd, J = 15.0, 7.4 Hz, 1H), 5.51 - 5.40 (m, 1H), 5.05 (d, J = 7.4 Hz, 1H), 3.75 (s, 3H), 3.70 (t, J = 6.3 Hz, 2H), 2.11 (dd, J = 13.9, 6.8 Hz, 2H), 1.66 - 1.58 (m, 2H), 1.50 (dt, J = 14.3, 7.2 Hz, 2H), 1.09 (s, 9H). ^{13}C NMR (100 MHz, $CDCl_3$) δ 156.75, 153.31, 149.38, 135.50, 134.03, 133.27, 130.33, 129.46, 128.99, 127.92, 127.53, 123.64, 120.55, 110.80, 63.69, 55.30, 46.58, 32.18, 32.01, 26.84, 25.48, 19.18. **HRMS (ESI)** m/z: $[M+H]^+$ Calcd For $C_{35}H_{42}NO_2Si$: 536.2985; Found:536.2983.

Analysis of Stereochemistry:

Enantiomeric excess: 89%, determined by HPLC (Daicel Chiralpak ADH hexane/isopropanol = 99/1, flow rate 0.5 mL/min, T = 25 °C, 220nm): t_R= 42.54 min (major), t_R= 49.07 min (minor).

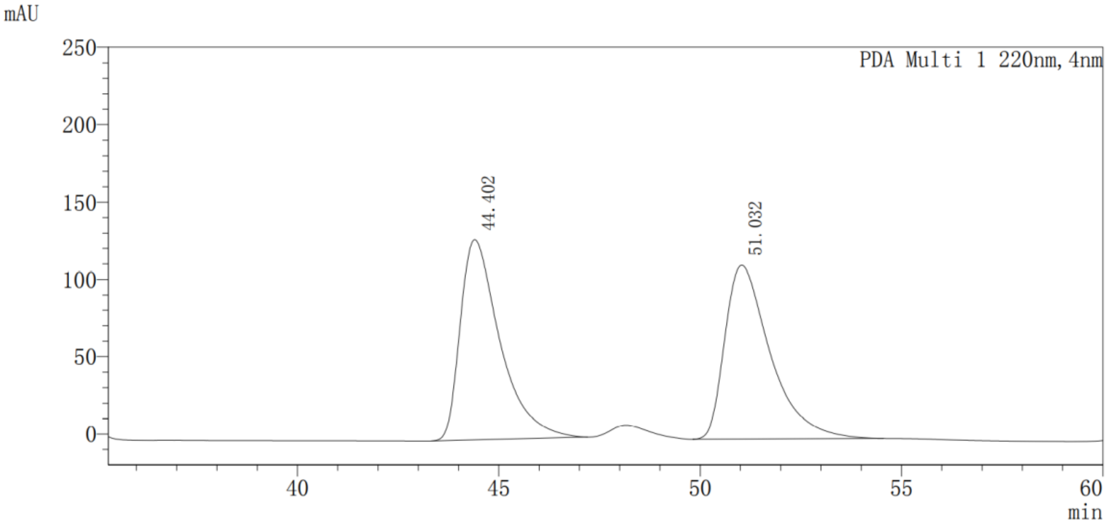

Peak	Ret Time[min]	Area[mAU*s]	Height [mAU]	Area %
1	44.402	8892836	129400	50.154
2	51.032	8838072	112464	49.846
Total		17730909	241864	100.000

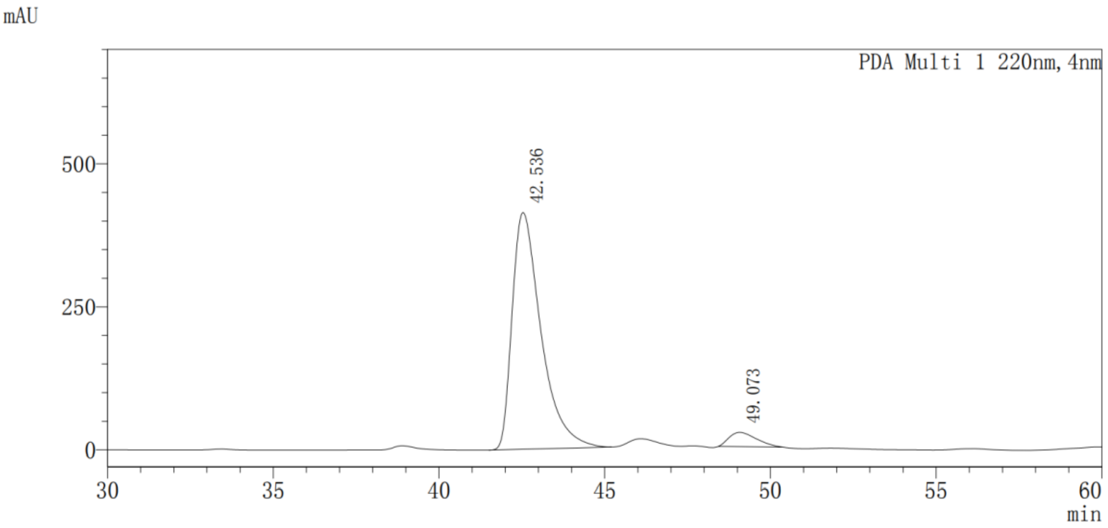

Peak	Ret Time[min]	Area[mAU*s]	Height [mAU]	Area %
1	42.536	25090864	413853	94.586
2	49.073	1436059	24900	5.414
Total		26526923	438753	100.000

Supplementary Figure 61. **HPLC chromatography for 3ak**

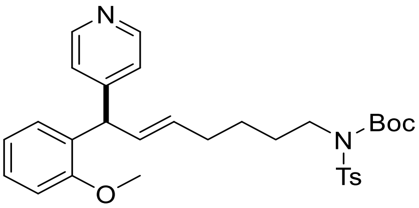

tert-butyl (R, E)-(7-(2-methoxyphenyl)-7-(pyridin-4-yl)hept-5-en-1-yl)(tosyl)carbamate (3al)

Following the general procedure, the product **3al** was isolated by chromatography on silica gel (PE/EA = 3/1 ~ 1/1, eluent) as a yellow oil (38%, 41.8 mg); $[\alpha]_D^{25}$ = 17.6 (c 0.9, $CHCl_3$); ^{1}H NMR (400 MHz, $CDCl_3$) δ 8.44 (d, *J* = 4.7 Hz, 2H), 7.75 (d, *J* = 7.7 Hz, 2H), 7.28 (d, *J* = 8.3 Hz, 2H), 7.22 (d, *J* = 7.5 Hz, 1H), 7.15 (d, *J* = 7.5 Hz, 1H), 7.08 (d, *J* = 5.0 Hz, 2H), 6.94 (t, *J* = 7.4 Hz, 1H), 6.86 (d, *J* = 8.2 Hz, 1H), 5.88 (dd, *J* = 15.3, 7.4 Hz, 1H), 5.43 (dd, *J* = 14.5, 7.4 Hz, 1H), 5.02 (d, *J* = 7.2 Hz, 1H), 3.84 - 3.79 (m, 2H), 3.72 (d, *J* = 0.7 Hz, 3H), 2.43 (s, 3H), 2.14 (dd, *J* = 13.9, 6.9 Hz, 2H), 1.76 (dd, *J* = 14.8, 7.8 Hz, 2H), 1.45 (dd, *J* = 15.0, 7.2 Hz, 2H), 1.31 (s, 9H). ^{13}C NMR (100 MHz, $CDCl_3$) δ 156.82, 153.36, 150.99, 149.38, 143.97, 137.64, 132.82, 130.86, 130.78, 129.19,

129.07, 127.96, 127.74, 123.69, 120.63, 110.88, 84.03, 55.37, 46.98, 46.64, 32.06, 29.72, 27.86, 26.36, 21.54. **HRMS (ESI)** m/z: $[M+Na]^+$ Calcd For $C_{31}H_{38}N_2O_5SNa$: 573.2399; Found:573.2394.

Analysis of Stereochemistry:

Enantiomeric excess: 88%, determined by HPLC (Daicel Chiralpak ADH hexane/isopropanol = 90/10, flow rate 1.0 mL/min, T = 25 °C, 220nm): t_R= 20.81 min (minor), t_R= 24.50 min (major).

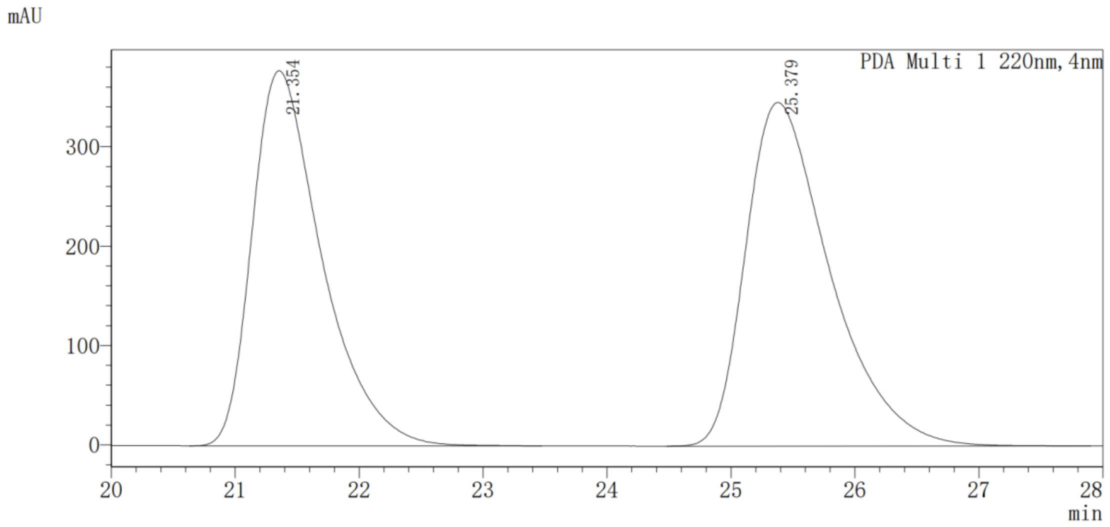

Peak	Ret Time[min]	Area[mAU*s]	Height [mAU]	Area %
1	21.354	14780506	377369	47.432
2	25.379	16380713	345649	52.568
Total		31161219	723019	100.000

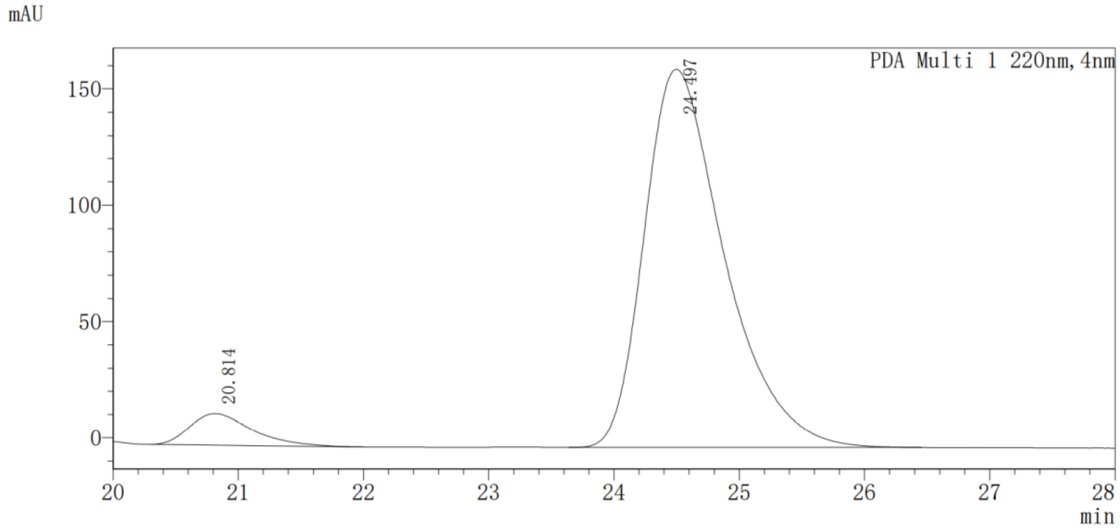

Peak	Ret Time[min]	Area[mAU*s]	Height [mAU]	Area %
1	20.814	473615	13588	6.141
2	24.497	7239327	162649	93.859
Total		7712942	176237	100.000

Supplementary Figure 62**. HPLC chromatography for 3al**

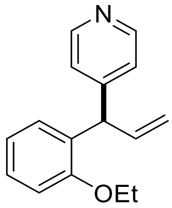

(R)-4-(1-(2-ethoxyphenyl)allyl)pyridine **(3am)**

Following the general procedure, the product **3am** was isolated by chromatography on silica gel (PE/EA = 10/1 ~ 5/1, eluent) as a pale yellow oil (60%, 28.7 mg); $[\alpha]_D^{25}$ = 22.7 (c 0.98, $CHCl_3$); ^{1}H NMR (400 MHz, $CDCl_3$) δ 8.49 (dd, *J* = 4.6, 1.4 Hz, 2H), 7.25 (td, *J* = 8.1, 1.7 Hz, 1H), 7.20 (dd, *J* = 7.6, 1.5 Hz, 1H), 7.16 - 7.07 (m, 2H), 6.96 (td, *J* = 7.5, 0.9 Hz, 1H), 6.86 (d, *J* = 8.1 Hz, 1H), 6.29 (ddd, *J* = 17.2, 10.2, 7.1 Hz, 1H), 5.32 - 5.20 (m, 1H), 5.02 (ddd, *J* = 17.2, 12.3, 4.2 Hz, 2H), 4.01 - 3.86 (m, 2H), 1.27 (t, *J* = 7.0 Hz, 3H). ^{13}C NMR (100 MHz, $CDCl_3$) δ 156.19, 152.54, 149.38, 138.58, 130.19, 129.00, 128.11, 123.84, 120.40, 117.18, 111.67, 63.55, 47.97, 14.63. **HRMS (ESI)** m/z: $[M+H]^+$ Calcd For $C_{16}H_{18}NO$: 240.1388; Found: 240.1387.

Analysis of Stereochemistry:

Enantiomeric excess: 93%, determined by HPLC (Agela Technologies Venusil CO hexane/isopropanol = 98.5/1.5, flow rate 1.0 mL/min, T = 25 °C, 220nm): t_R= 13.79 min (major), t_R= 15.82 min (minor).

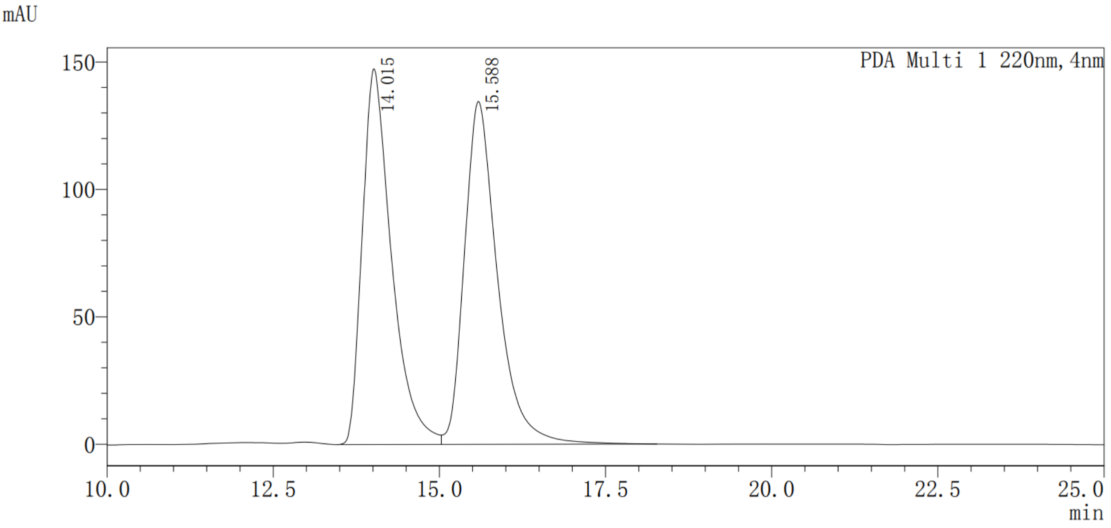

Peak	Ret Time[min]	Area[mAU*s]	Height [mAU]	Area %
1	14.015	4515617	147419	49.149
2	15.588	4671968	134546	50.851
Total		9187585	281965	100.000

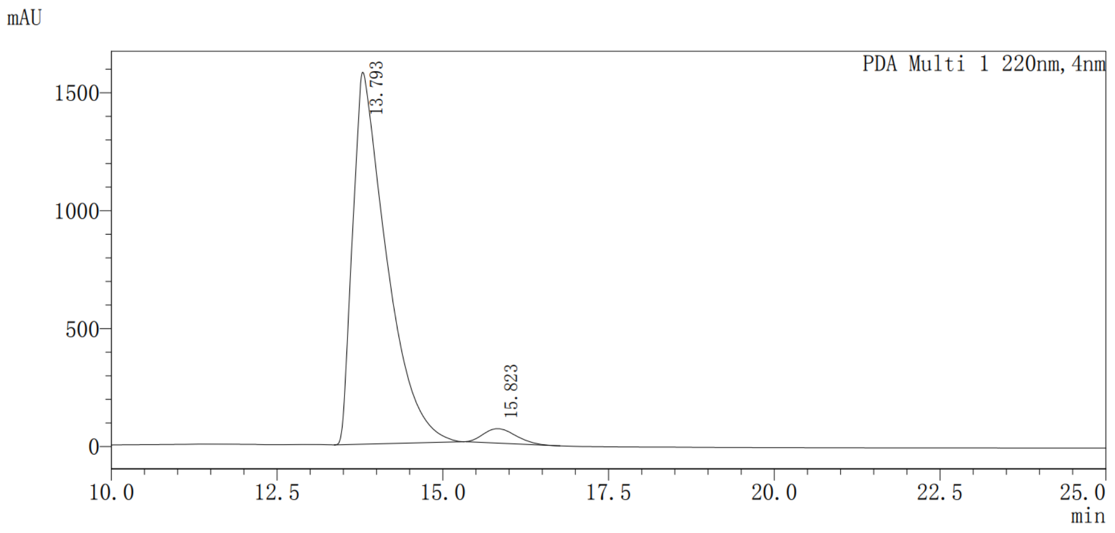

Peak	Ret Time[min]	Area[mAU*s]	Height [mAU]	Area %
1	13.793	56081769	1577164	96.406
2	15.823	2090620	61881	3.594
Total		58172389	1639045	100.000

Supplementary Figure 63. **HPLC chromatography for 3am**

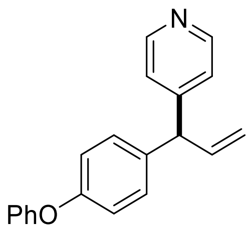

(*R*)-4-(1-(4-phenoxyphenyl)allyl)pyridine (3an)

Following the general procedure, the product **3an** was isolated by chromatography on silica gel (PE/EA = 10/1 ~ 5/1, eluent) as a pale yellow oil (62%, 35.6 mg); $[\alpha]_D^{25}$ = 8.8 (c 0.6, $CHCl_3$); ^{1}H NMR (400 MHz, Chloroform-*d*) δ 8.65 – 8.52 (m, 2H), 7.36 (dd, *J* = 8.6, 7.3 Hz, 2H), 7.20 – 7.10 (m, 5H), 7.07 – 7.01 (m, 2H), 7.01 – 6.95 (m, 2H), 6.26 (ddd, *J* = 17.2, 10.2, 7.2 Hz, 1H), 5.32 (dd, *J* = 10.2, 1.3 Hz, 1H), 5.06 (dt, *J* = 17.0, 1.5 Hz, 1H), 4.71 (d, *J* = 7.2 Hz, 1H). ^{13}C NMR (100 MHz, Chloroform-*d*) δ 157.00, 156.19, 152.12, 149.82, 138.90, 136.15, 129.77, 129.73, 123.78, 123.35, 118.93, 118.85, 117.52, 53.59. **HRMS (ESI)** m/z: $[M+H]^+$ Calcd for $C_{20}H_{18}NO$ 288.1388; Found 288.1390.

Analysis of Stereochemistry:

Enantiomeric excess: 91%, determined by HPLC (Agela Technologies Venusil CJ hexane/isopropanol = 95/5, flow rate 1.0 mL/min, T = 25 °C, 220nm): t_R= 30.83 min (major), t_R= 33.38 min (minor).

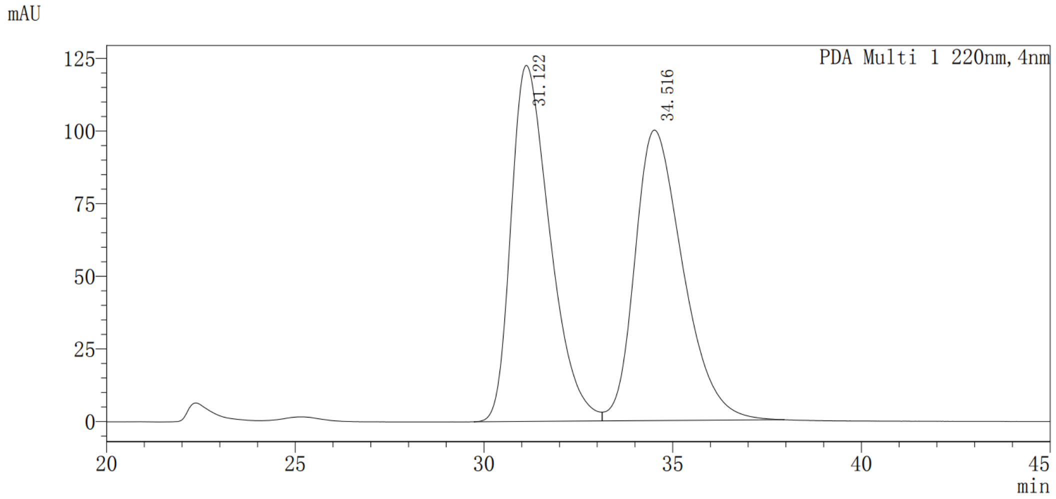

Peak	Ret Time[min]	Area[mAU*s]	Height [mAU]	Area %
1	31.122	8779905	122567	49.712
2	34.516	8881775	100014	50.288
Total		17661680	222580	100.000

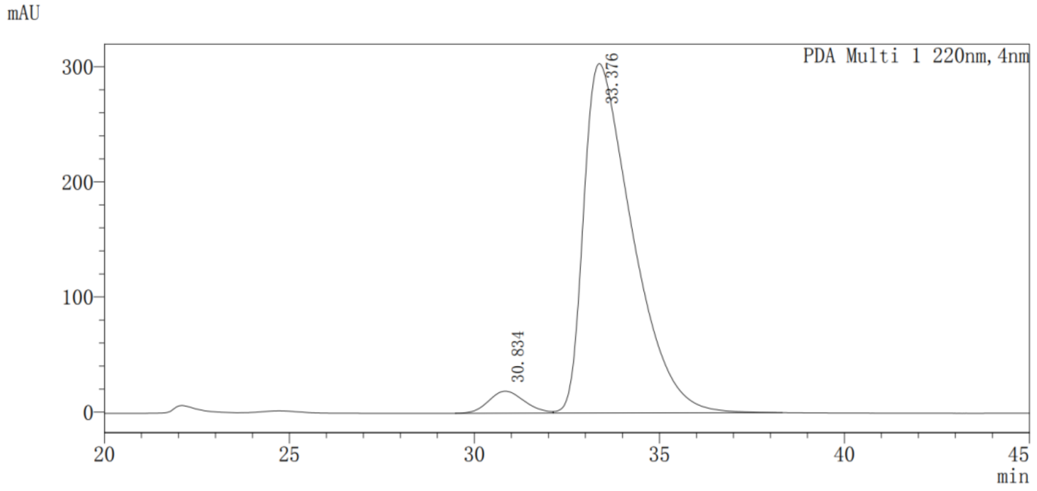

Peak	Ret Time[min]	Area[mAU*s]	Height [mAU]	Area %
1	30.834	1281943	19152	4.404
2	33.376	27829359	303444	95.596
Total		29111303	322596	100.000

Supplementary Figure 64. **HPLC chromatography for 3an**

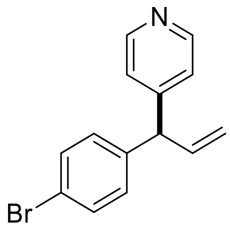

(R)-4-(1-(4-bromophenyl)allyl)pyridine **(3ao)**

Following the general procedure, the product **3ao** was isolated by chromatography on silica gel (PE/EA = 10/1 ~ 5/1, eluent) as a yellow oil (51%, 27.8 mg); $[\alpha]_D^{25}$ = 9.8 (c 0.9 $CHCl_3$); ^1H NMR (400 MHz, $CDCl_3$) δ 8.52 (d, *J* = 5.0 Hz, 2H), 7.44 (d, *J* = 8.1 Hz, 2H), 7.06 (dd, *J* = 19.3, 6.6 Hz, 4H), 6.19 (ddd, *J* = 17.2, 10.0, 7.4 Hz, 1H), 5.30 (d, *J* = 10.2 Hz, 1H), 5.02 (d, *J* = 17.1 Hz, 1H), 4.65 (d, *J* = 7.0 Hz, 1H). ^{13}C NMR (100 MHz, $CDCl_3$) δ 151.40, 149.93, 140.46, 138.28, 131.76, 130.24, 123.68, 120.90, 118.01, 53.64. **HRMS (ESI)** m/z: $[M+H]^+$ Calcd for $C_{14}H_{13}BrN$ 274.0231; Found 274.0231.

Analysis of Stereochemistry:

Enantiomeric excess: 83%, determined by HPLC (Agela Technologies Venusil CJ hexane/isopropanol = 98/2, flow rate 1.0 mL/min, T = 25 °C, 220nm): t_R= 22.89 min (major), t_R= 27.94 min (minor).

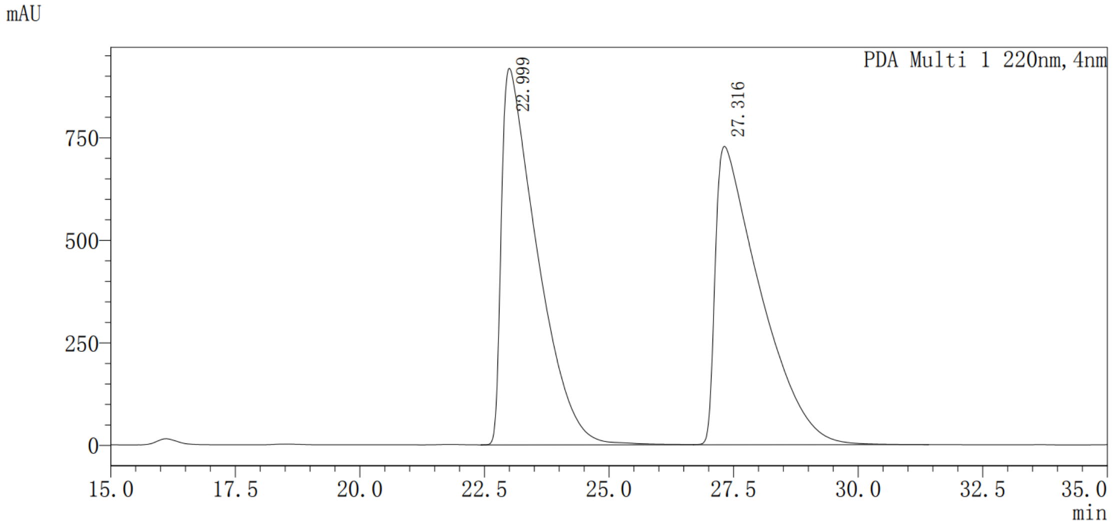

Peak	Ret Time[min]	Area[mAU*s]	Height [mAU]	Area %
1	22.999	45800331	917674	50.060
2	27.316	45689689	727226	49.940
Total		91490020	1644900	100.000

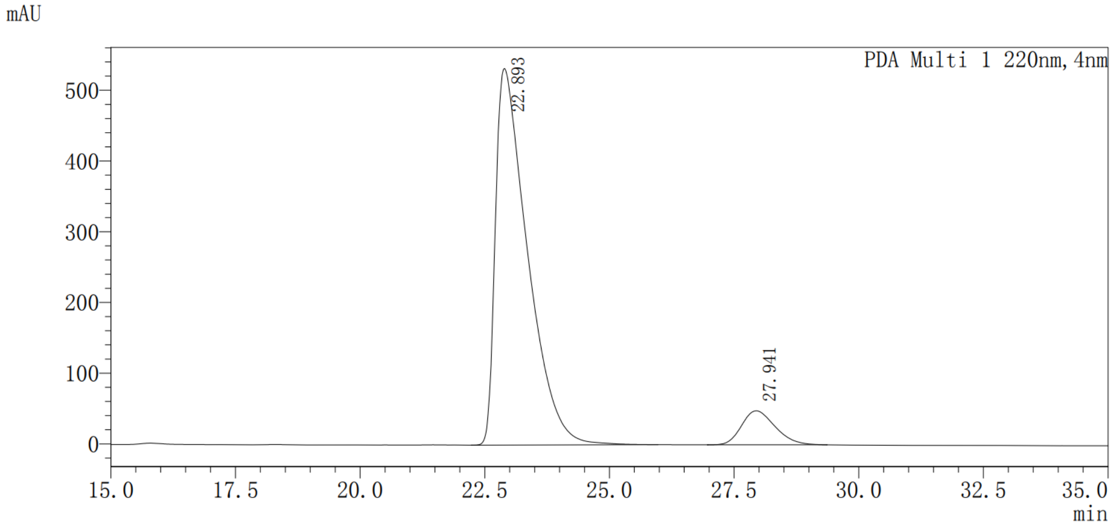

Peak	Ret Time[min]	Area[mAU*s]	Height [mAU]	Area %

1	22.893	23820869	532466	91.349
2	27.941	2255983	48116	8.651
Total		26076851	580581	100.000

Supplementary Figure 65. **HPLC chromatography for 3ao**

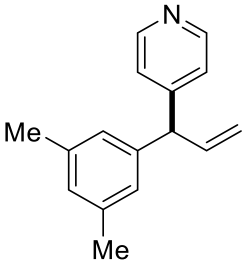

(R)-4-(1-(3,5-dimethylphenyl)allyl)pyridine **(3ap)**

Following the general procedure, the product **3ap** was isolated by chromatography on silica gel (PE/EA = 10/1 ~ 5/1, eluent) as a pale yellow oil (60%, 26.8 mg); $[\alpha]_D^{25}$ = 21.7 (c 0.7, $CHCl_3$); ^{1}H NMR (400 MHz, $CDCl_3$) δ 8.51 (d, J = 4.8 Hz, 2H), 7.12 (d, J = 5.0 Hz, 2H), 6.89 (s, 1H), 6.78 (s, 2H), 6.24 (ddd, J = 17.2, 9.9, 7.7 Hz, 1H), 5.27 (d, J = 10.1 Hz, 1H), 5.03 (d, J = 17.1 Hz, 1H), 4.61 (d, J = 7.2 Hz, 1H), 2.28 (s, 6H). ^{13}C NMR (100 MHz, $CDCl_3$) δ 152.32, 149.73, 141.41, 139.05, 138.18, 128.54, 126.27, 123.81, 117.25, 54.26, 21.28. **HRMS (ESI)** m/z: $[M+H]^+$ Calcd for $C_{16}H_{18}N$ 224.1439; Found 224.1439.

Analysis of Stereochemistry:

Enantiomeric excess: 82%, determined by HPLC (Daicel Chiralpak OZH hexane/isopropanol = 99/1, flow rate 1.0 mL/min, T = 25 °C, 220nm): t_R= 18.88 min (major), t_R= 20.07 min (minor).

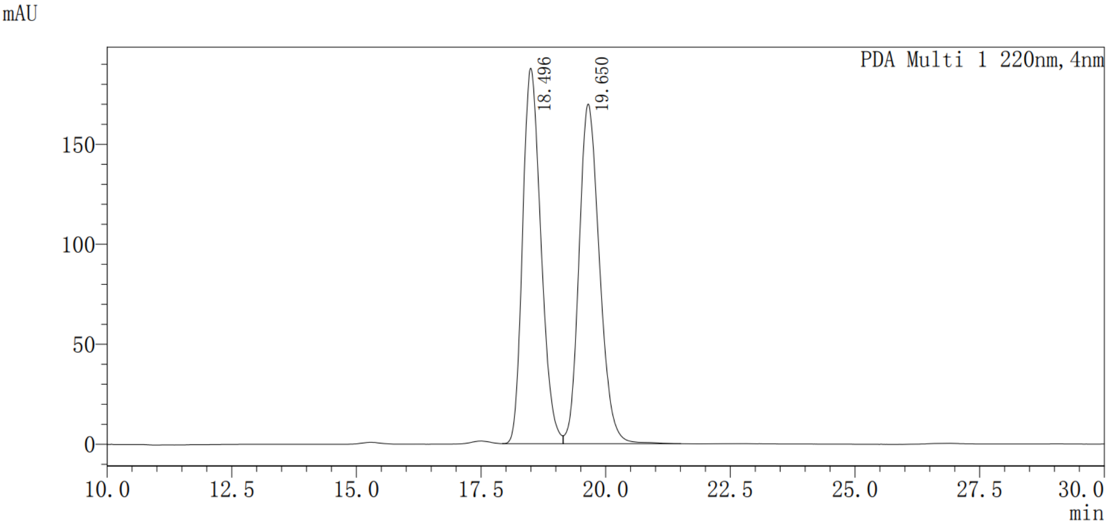

Peak	Ret Time[min]	Area[mAU*s]	Height [mAU]	Area %
1	18.496	4815014	187802	49.734
2	19.650	4866546	169899	50.266
Total		9681560	357701	100.000

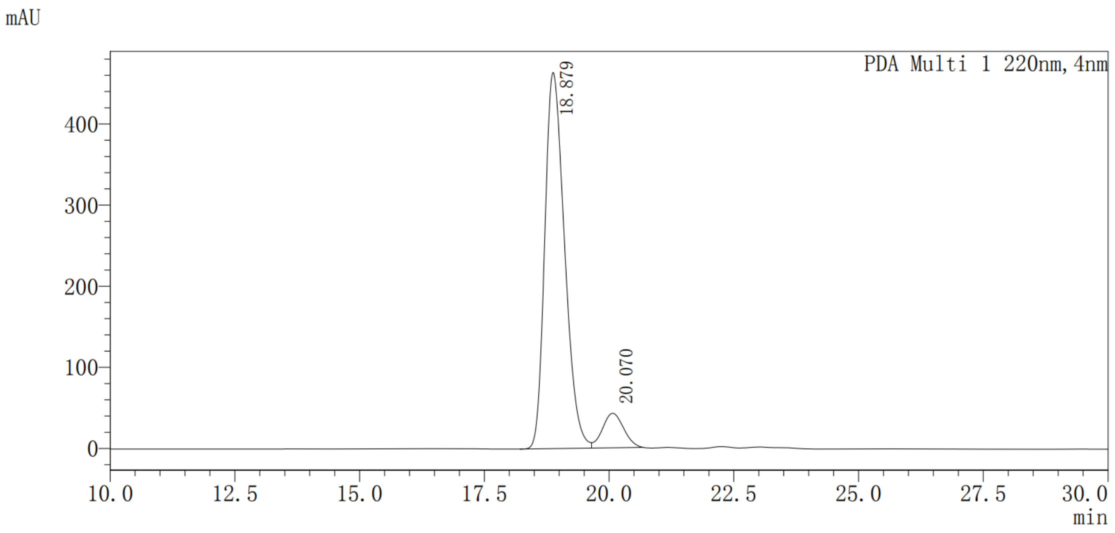

Peak	Ret Time[min]	Area[mAU*s]	Height [mAU]	Area %
1	18.879	12795628	463771	91.081
2	20.070	1252974	42557	8.919
Total		14048602	506328	100.000

Supplementary Figure 66. **HPLC chromatography for 3ap**

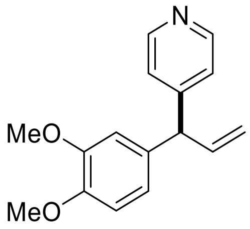

(*R*)-4-(1-(3,4-dimethoxyphenyl)allyl)pyridine (3aq)

Following the general procedure, the product **3aq** was isolated by chromatography on silica gel (PE/EA = 10/1 ~ 5/1, eluent) as a pale yellow oil (56%, 28.6 mg); $[\alpha]_D^{25}$ = 19.3 (c 0.5, $CHCl_3$); ^1H NMR (400 MHz, Chloroform-*d*) δ 8.54 – 8.49 (m, 2H), 7.14 – 7.07 (m, 2H), 6.82 (d, *J* = 8.2 Hz, 1H), 6.70 (dd, *J* = 8.3, 2.1 Hz, 1H), 6.65 (d, *J* = 2.0 Hz, 1H), 6.22 (ddd, *J* = 17.3, 10.2, 7.2 Hz, 1H), 5.28 (dt, *J* = 10.2, 1.3 Hz, 1H), 5.02 (dt, *J* = 17.1, 1.4 Hz, 1H), 4.64 (d, *J* = 7.2 Hz, 1H), 3.86 (s, 3H), 3.82 (s, 3H). ^{13}C NMR (100 MHz, Chloroform-*d*) δ 152.30, 149.74, 149.06, 147.98, 138.99, 133.93, 123.77, 120.57, 117.38, 111.78, 111.21, 55.88, 55.84, 53.82. **HRMS (ESI)** m/z: $[M+H]^+$ Calcd for $C_{16}H_{18}NO_2$ 256.1338; Found 256.1342.

Analysis of Stereochemistry:

Enantiomeric excess: 83%, determined by HPLC (Daicel Chiralpak ADH hexane/isopropanol = 95/5, flow rate 1.0 mL/min, T = 25 °C, 220nm): t_R= 32.62 min (major), t_R= 39.62 min (minor).

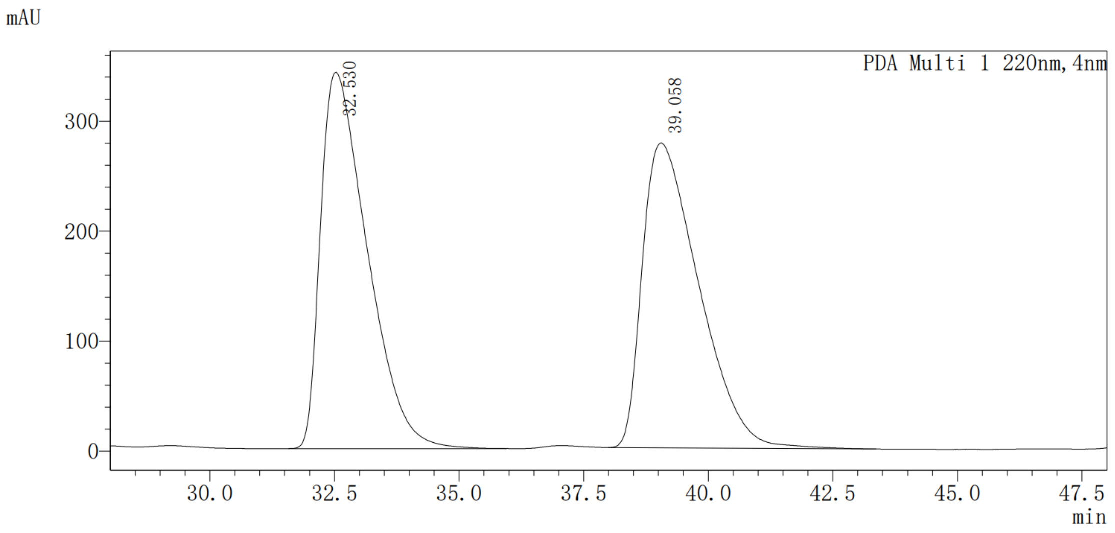

Peak	Ret Time[min]	Area[mAU*s]	Height [mAU]	Area %
1	32.530	22272131	342217	50.425
2	39.058	21896854	277244	49.575
Total		44168985	619462	100.000

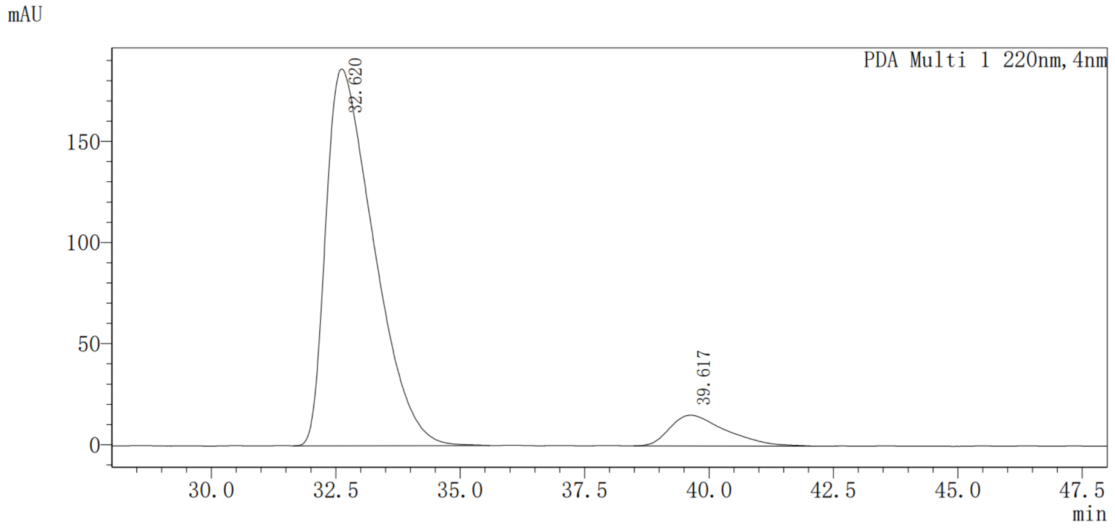

Peak	Ret Time[min]	Area[mAU*s]	Height [mAU]	Area %
1	32.620	12480246	186287	91.301
2	39.617	1189024	15232	8.699
Total		13669271	201520	100.000

Supplementary Figure 67. **HPLC chromatography for 3aq**

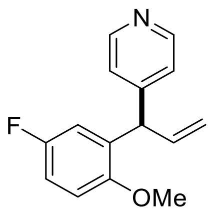

(R)-4-(1-(5-fluoro-2-methoxyphenyl)allyl)pyridine **(3ar)**

Following the general procedure, the product **3ar** was isolated by chromatography on silica gel (PE/EA = 1/1, eluent) as a colorless oil (57%, 27.7 mg); a pale yellow oil; $[\alpha]_D^{25}$ = 25.7 (c 0.6, $CHCl_3$); ^{1}H NMR (400 MHz, $CDCl_3$) δ 8.48 (d, *J* = 5.8 Hz, 2H), 7.07 (dd, *J* = 4.7, 1.3 Hz, 2H), 6.96 – 6.90 (m, 1H), 6.87 (dd, *J* = 9.2, 3.1 Hz, 1H), 6.79 (dd, *J* = 8.9, 4.5 Hz, 1H), 6.19 (ddd, *J* = 17.1, 10.2, 6.9 Hz, 1H), 5.28 (dt, *J* = 10.2, 1.2 Hz, 1H), 5.05 (d, *J* = 6.8 Hz, 1H), 4.97 (dt, *J* = 17.2, 1.4 Hz, 1H), 3.70 (s, 3H). ^{13}C NMR (100 MHz, $CDCl_3$) δ 158.19, 155.81, 154.41 (d, J_{C-F} = 279.7 Hz), 152.99, 151.55, 149.60, 137.92, 131.67 (d, J_{C-F} = 6.7 Hz), 123.74, 117.82, 116.19, 115.96, 114.06, 113.83, 111.73 (d, J_{C-F} = 8.3 Hz), 55.98, 47.30 (d, J_{C-F} = 1.2 Hz). ^{19}F NMR (376 MHz, $CDCl_3$) δ -123.35 (s). **HRMS (ESI)** m/z: $[M+H]^+$ Calcd For $C_{15}H_{15}FNO$: 244.1138; Found: 244.1138.

Analysis of Stereochemistry:

Enantiomeric excess: 88%, determined by HPLC (Daicel Chiralpak OZH, hexane/isopropanol = 98/2, flow rate 1.0 mL/min, T = 25 °C, 220nm): t_R= 31.31 min (major), t_R= 41.99 min (minor).

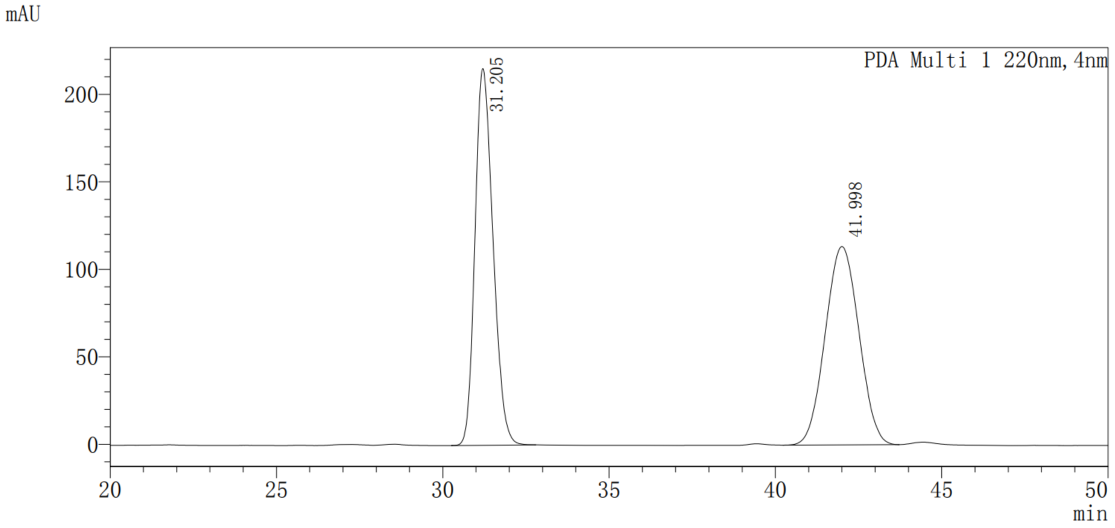

Peak	Ret Time[min]	Height [mAU]	Area[mAU*s]	Area %
1	31.205	215252	8119611	50.432
2	41.998	113352	7980404	49.568
Total		328605	16100015	100.000

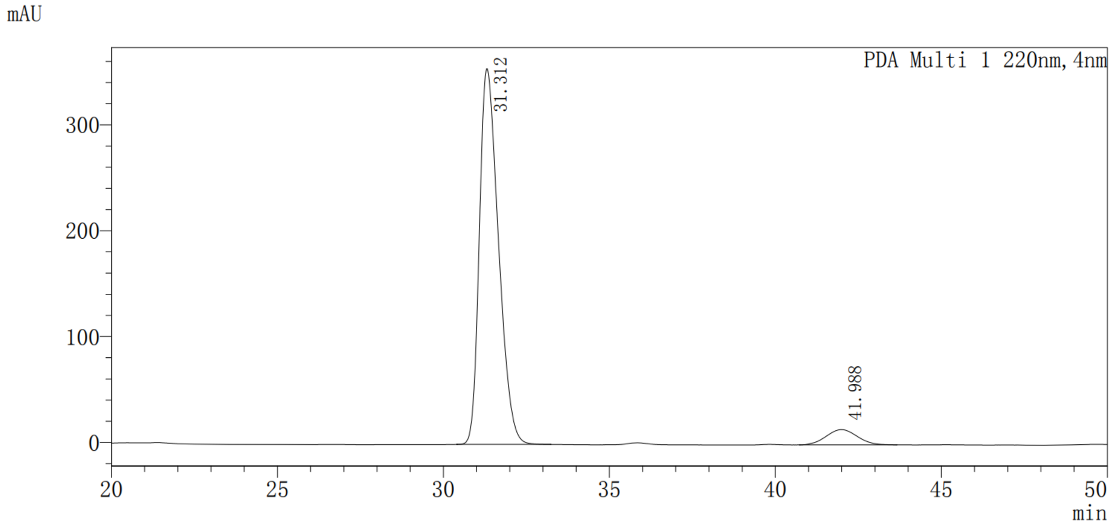

Peak	Ret Time[min]	Height [mAU]	Area[mAU*s]	Area %
1	31. 312	355040	14190469	93. 772
2	41. 988	14440	942543	6. 228
Total		369480	15133013	100. 000

Supplementary Figure 68. **HPLC chromatography for 3ar**

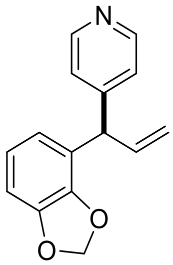

(R)-4-(1-(benzo[d][1,3]dioxol-4-yl)allyl)pyridine **(3as)**

Following the general procedure, the product **3as** was isolated by chromatography on silica gel (PE/EA = 10/1 ~ 5/1, eluent) as a yellow oil (75%, 35.9 mg); $[\alpha]_D^{25}$ = 37.4 (c 1.1, $CHCl_3$); ^1H NMR (400 MHz, $CDCl_3$) δ 8.53 (dd, *J* = 4.5, 1.6 Hz, 2H), 7.18 (dd, *J* = 4.7, 1.1 Hz, 2H), 6.87 - 6.75 (m, 2H), 6.68 (dd, *J* = 7.8, 1.0 Hz, 1H), 6.28 (ddd, *J* = 17.2, 10.2, 7.3 Hz, 1H), 5.93 (dd, *J* = 2.6, 1.4 Hz, 2H), 5.32 (dt, *J* = 10.2, 1.2 Hz, 1H), 5.11 (dt, *J* = 17.1, 1.3 Hz, 1H), 4.83 (d, *J* = 7.3 Hz, 1H). ^{13}C NMR (100 MHz, $CDCl_3$) δ 151.15, 149.60, 147.50, 145.22, 137.21, 123.59, 123.00, 121.85, 121.48, 117.74, 107.53, 100.75, 48.60. **HRMS (ESI)** m/z: $[M+H]^+$ Calcd For $C_{15}H_{14}NO_2$: 240.1025; Found: 240.1024.

Analysis of Stereochemistry:

Enantiomeric excess: 85%, determined by HPLC (Daicel Chiralpak OZH hexane/isopropanol = 98/2, flow rate 1.0 mL/min, T = 25 °C, 220nm): t_R= 33.90 min (major), t_R= 35.46 min (minor).

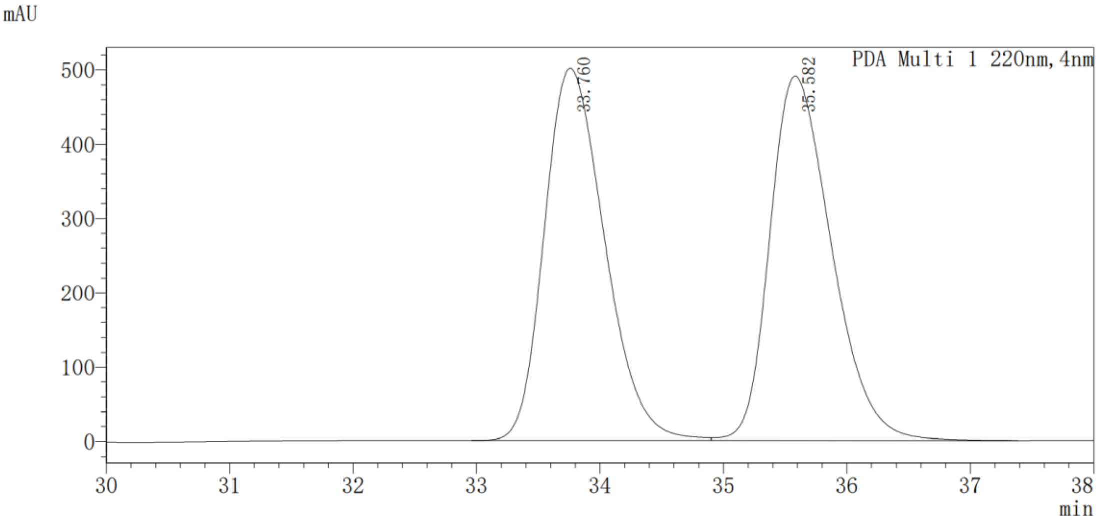

Peak	Ret Time[min]	Area[mAU*s]	Height [mAU]	Area %

1	33. 760	16997504	500582	50. 002
2	35. 582	16996423	490265	49. 998
Total		33993927	990847	100. 000

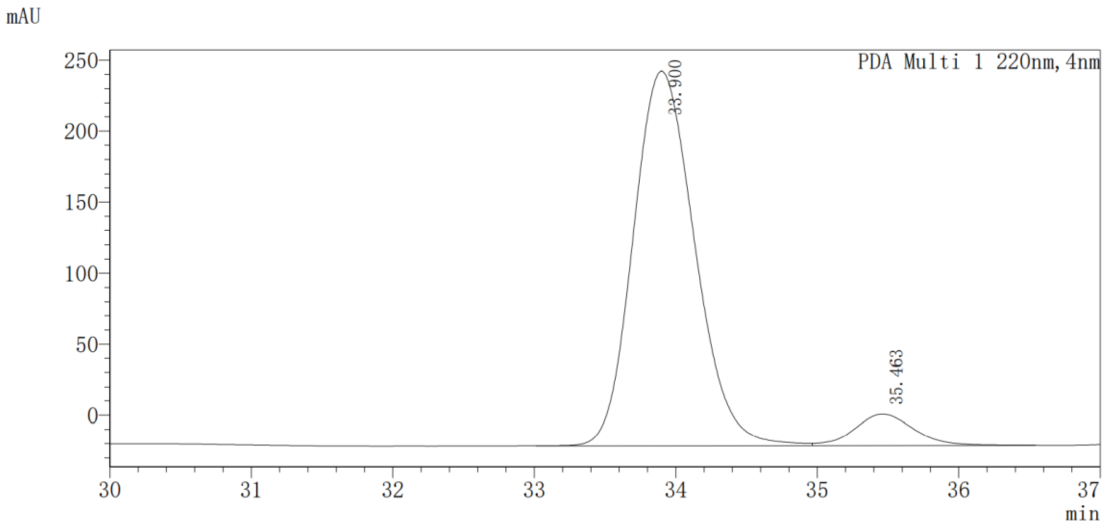

Peak	Ret Time[min]	Area[mAU*s]	Height [mAU]	Area %
1	33. 900	7956876	263853	92. 522
2	35. 463	643066	22177	7. 478
Total		8599942	286030	100. 000

Supplementary Figure 69. **HPLC chromatography for 3as**

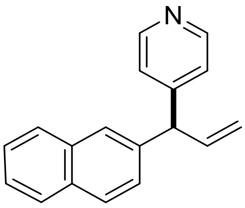

(R)-4-(1-(naphthalen-2-yl)allyl)pyridine **(3at)**

Following the general procedure, the product **3at** was isolated by chromatography on silica gel (PE/EA = 10/1 ~ 5/1, eluent) as a pale yellow oil (59%, 28.9 mg); $[\alpha]_D^{25}$ = 18.9 (c 0.8, $CHCl_3$); ^1H NMR (400 MHz, $CDCl_3$) δ 8.53 (d, J = 6.0 Hz, 2H), 7.80 (dd, J = 11.3, 7.1 Hz, 3H), 7.64 (s, 1H), 7.52 - 7.43 (m, 2H), 7.27 (d, J = 1.6 Hz, 1H) 7.16 (d, J = 6.0 Hz, 2H), 6.34 (ddd, J = 17.3, 10.2, 7.2 Hz, 1H), 5.35 (d, J = 10.2 Hz, 1H), 5.09 (d, J = 17.1 Hz, 1H), 4.87 (d, J = 7.2 Hz, 1H). ^{13}C NMR (100 MHz, $CDCl_3$) δ 151.97, 149.85, 138.92, 138.73, 133.47, 132.41, 128.38, 127.76, 127.62, 127.04, 126.82, 126.26, 125.92, 123.94, 117.83, 54.38. **HRMS (ESI)** m/z: $[M+H]^+$ Calcd For $C_{18}H_{16}N$: 246.1283; Found: 246.1286.

Analysis of Stereochemistry:

Enantiomeric excess: 82%, determined by HPLC (Daicel Chiralpak ADH, hexane/isopropanol = 98/2, flow rate 1.0 mL/min, T = 25 °C, 220nm): t_R= 30.54 min (major), t_R= 35.00 min (minor).

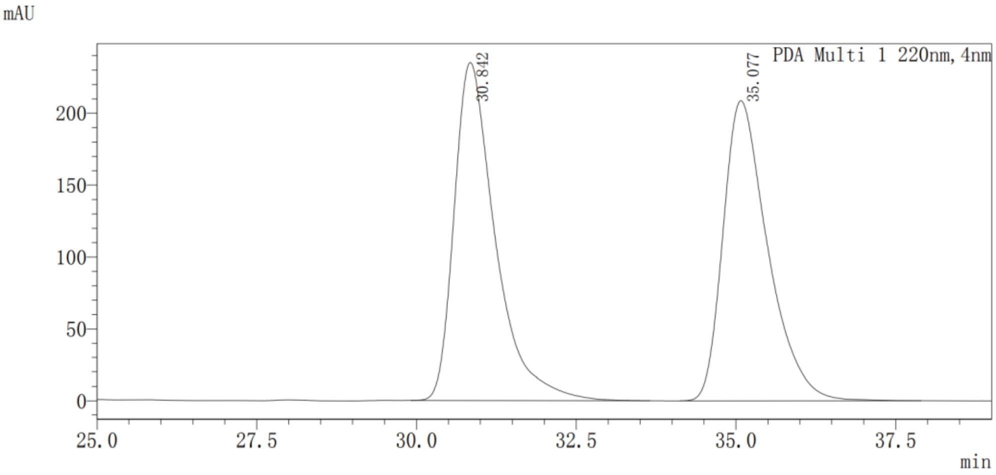

Peak	Ret Time[min]	Area[mAU*s]	Height [mAU]	Area %
1	30. 842	10533455	235058	50. 905
2	35. 077	10158742	208713	49. 095
Total		20692197	443771	100. 000

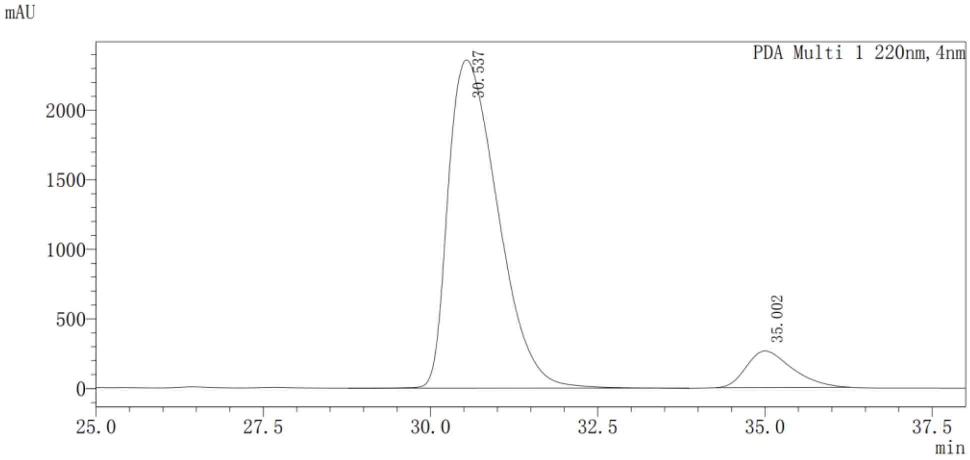

Peak	Ret Time[min]	Area[mAU*s]	Height [mAU]	Area %
1	30. 537	121855276	2360386	90. 725
2	35. 002	12458176	262269	9. 275
Total		134313452	2622655	100. 000

Supplementary Figure 70**. HPLC chromatography for 3at**

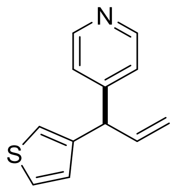

(*R*)-4-(1-(thiophen-3-yl)allyl)pyridine (3au)

Following the general procedure, the product **3au** was isolated by chromatography on silica gel (PE/EA = 10/1 ~ 5/1, eluent) as a pale yellow oil (72%, 28.9 mg); $[\alpha]_D^{25}$ = 19.5 (c 1.4, $CHCl_3$); ^{1}H NMR (400 MHz, $CDCl_3$) δ 8.52 (d, *J* = 6.0 Hz, 2H), 7.30 (dd, *J* = 5.0, 2.9 Hz, 1H), 7.12 (d, *J* = 6.1 Hz, 2H), 6.99 – 6.95 (m, 1H), 6.85 (dd, *J* = 5.0, 1.3 Hz, 1H), 6.21 (ddd, *J* = 17.3, 10.1, 7.4 Hz, 1H), 5.26 (d, *J* = 10.1 Hz, 1H), 5.06 (d, *J* = 17.0 Hz, 1H), 4.74 (d, *J* = 7.3 Hz, 1H). ^{13}C NMR (100 MHz,

$CDCl_3$) δ 151.85, 149.86, 142.02, 138.59, 127.70, 126.09, 123.59, 121.99, 117.17, 50.09. **HRMS (ESI)** m/z: $[M+H]^+$ Calcd For $C_{12}H_{12}NS$: 202.0690; Found: 202.0688.

Analysis of Stereochemistry:

Enantiomeric excess: 85%, determined by HPLC (Daicel Chiralpak ADH, hexane/isopropanol = 98/2, flow rate 1.0 mL/min, T = 25 °C, 220nm): t_R= 17.88 min (major), t_R= 19.01 min (minor).

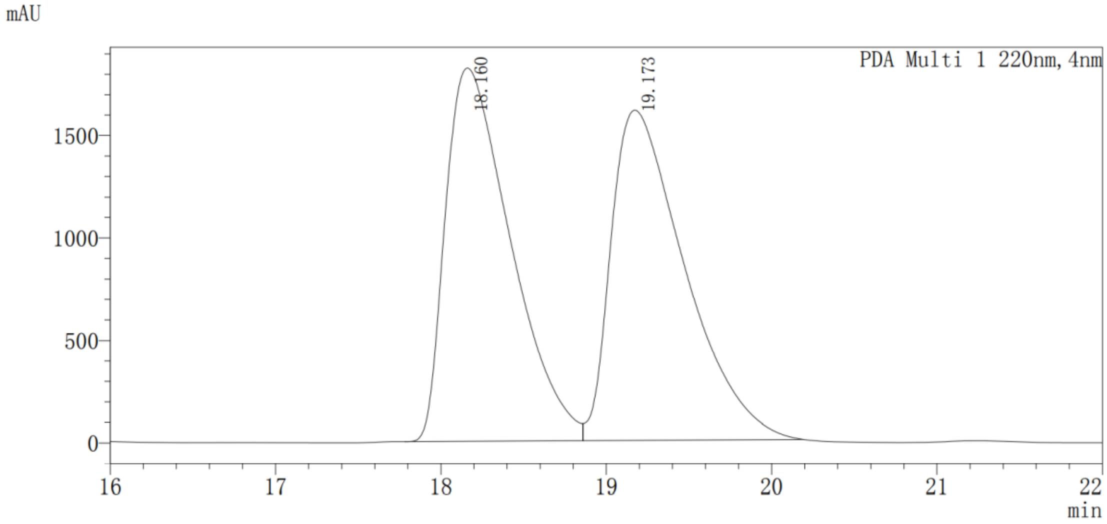

Peak	Ret Time[min]	Area[mAU*s]	Height [mAU]	Area %
1	18. 160	49918257	1822098	49. 999
2	19. 173	49920409	1611767	50. 001
Total		99838665	3433865	100. 000

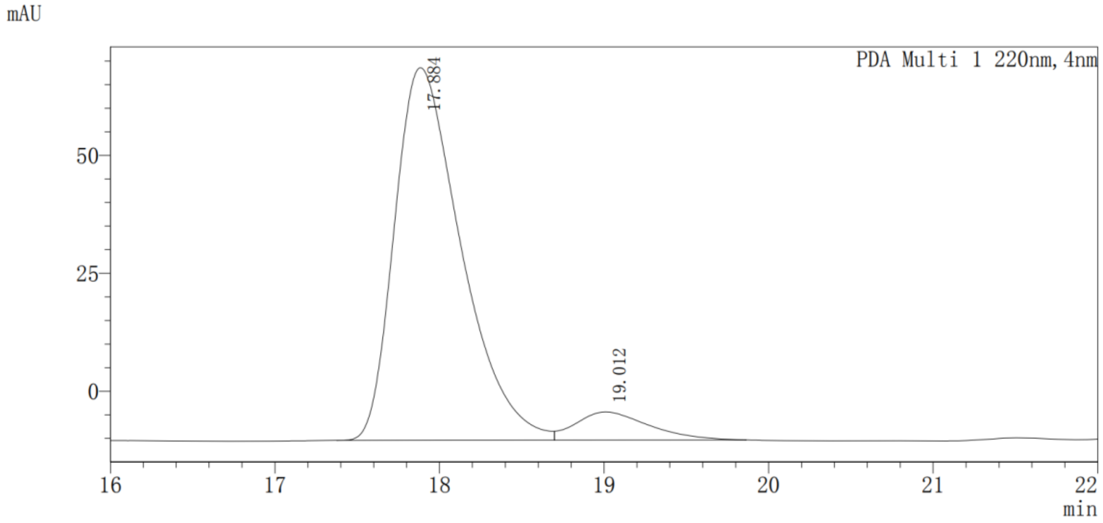

Peak	Ret Time[min]	Area[mAU*s]	Height [mAU]	Area %
1	17. 884	2225292	78979	92. 347
2	19. 012	184423	5962	7. 653
Total		2409715	84941	100. 000

Supplementary Figure 71**. HPLC chromatography for 3au**

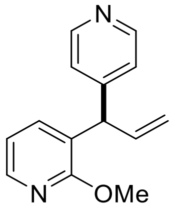

(R)-2-methoxy-3-(1-(pyridin-4-yl)allyl)pyridine **(3av)**

Following the general procedure, the product **3av** was isolated by chromatography on silica gel (PE/EA = 3/1 ~ 1/1, eluent) as a yellow oil (64%, 28.9 mg); $[\alpha]_D^{25}$ = 17.7 (c 0.9, $CHCl_3$); ^{1}H NMR (400 MHz, $CDCl_3$) δ 8.49 (d, *J* = 5.8 Hz, 2H), 8.09 (dd, *J* = 5.0, 1.7 Hz, 1H), 7.41 (dd, *J* = 7.3, 1.7 Hz, 1H), 7.09 (d, *J* = 5.9 Hz, 2H), 6.88 (dd, *J* = 7.3, 5.0 Hz, 1H), 6.20 (ddd, *J* = 17.1, 10.2, 7.0 Hz, 1H), 5.29 (d, *J* = 10.2 Hz, 1H), 4.97 (t, *J* = 11.5 Hz, 2H), 3.87 (s, 3H). ^{13}C NMR (100 MHz, $CDCl_3$) δ 161.40, 151.42, 149.37, 146.67, 145.45, 137.51, 137.33, 123.80, 117.95, 116.74, 53.41, 47.51. **HRMS (ESI)** m/z: $[M+H]^+$ Calcd For $C_{14}H_{15}N_2O$: 227.1184; Found: 227.1184.

Analysis of Stereochemistry:

Enantiomeric excess: 82%, determined by HPLC (Agela Technologies Venusil CJ, hexane/isopropanol = 97/3, flow rate 1.0 mL/min, T = 25 °C, 220nm): t_R= 12.39 min (major), t_R= 17.78 min (minor).

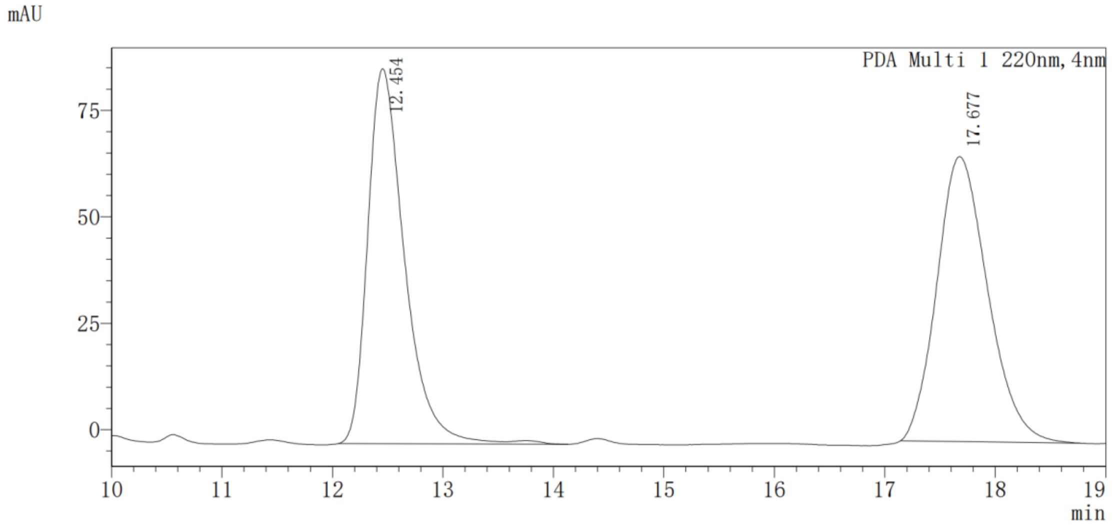

Peak	Ret Time[min]	Area[mAU*s]	Height [mAU]	Area %
1	12. 454	2049097	88030	48. 844
2	17. 677	2146072	66949	51. 156
Total		4195168	154978	100. 000

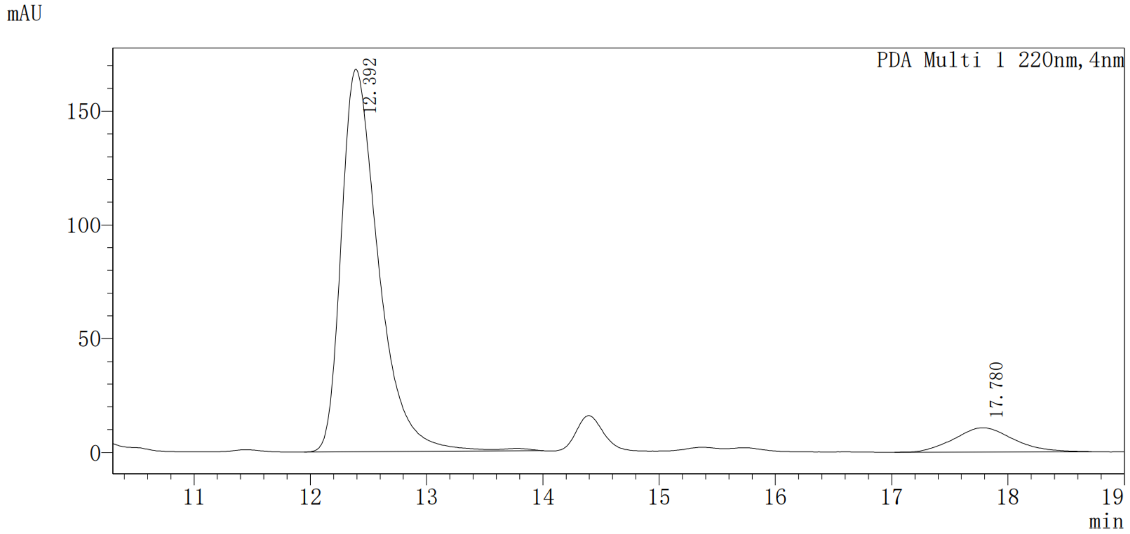

Peak	Ret Time[min]	Area[mAU*s]	Height [mAU]	Area %
1	12. 392	3751521	168129	90. 868
2	17. 780	377006	10618	9. 132
Total		4128527	178747	100. 000

Supplementary Figure 72. **HPLC chromatography for 3av**

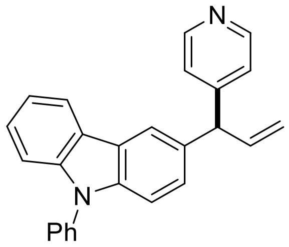

(R)-9-phenyl-3-(1-(pyridin-4-yl)allyl)-9H-carbazole **(3aw)**

Following the general procedure, the product **3aw**s isolated by chromatography on silica gel (PE/EA = 10/1 ~ 5/1, eluent) as a pale yellow oil (68%, 48.9 mg); $[\alpha]_D^{25}$ = 12.4 (c 1.2, $CHCl_3$); ^{1}H NMR (400 MHz, Chloroform-*d*) δ 8.63 – 8.50 (m, 2H), 8.14 (d, *J* = 7.8 Hz, 1H), 7.98 (d, *J* = 1.8 Hz, 1H), 7.68 – 7.54 (m, 4H), 7.54 – 7.46 (m, 1H), 7.44 (d, *J* = 4.0 Hz, 2H), 7.39 (d, *J* = 8.5 Hz, 1H), 7.31 (dt, *J* = 8.9, 4.5 Hz, 1H), 7.22 (dd, *J* = 7.1, 3.4 Hz, 3H), 6.42 (ddd, *J* = 17.2, 10.2, 7.1 Hz, 1H), 5.37 (d, *J* = 10.2 Hz, 1H), 5.12 (d, *J* = 17.1 Hz, 1H), 4.94 (d, *J* = 7.1 Hz, 1H). ^{13}C NMR (100 MHz, Chloroform-*d*) δ 152.83, 149.74, 141.18, 139.84, 139.52, 137.57, 133.10, 129.85, 127.46, 126.98, 126.61, 126.09, 123.92, 123.56, 123.05, 120.27, 120.02, 119.96, 117.33, 109.96, 109.84, 54.26. **HRMS (ESI)** m/z: $[M+H]^+$ Calcd for $C_{26}H_{21}N_2$ 361.1705; Found 361.1704.

Analysis of Stereochemistry:

Enantiomeric excess: 81%, determined by HPLC (Daicel Chiralpak ASH hexane/isopropanol = 97/3, flow rate 1.0 mL/min, T = 25 °C, 220nm): t_R= 16.50 min (major), t_R= 20.43 min (minor).

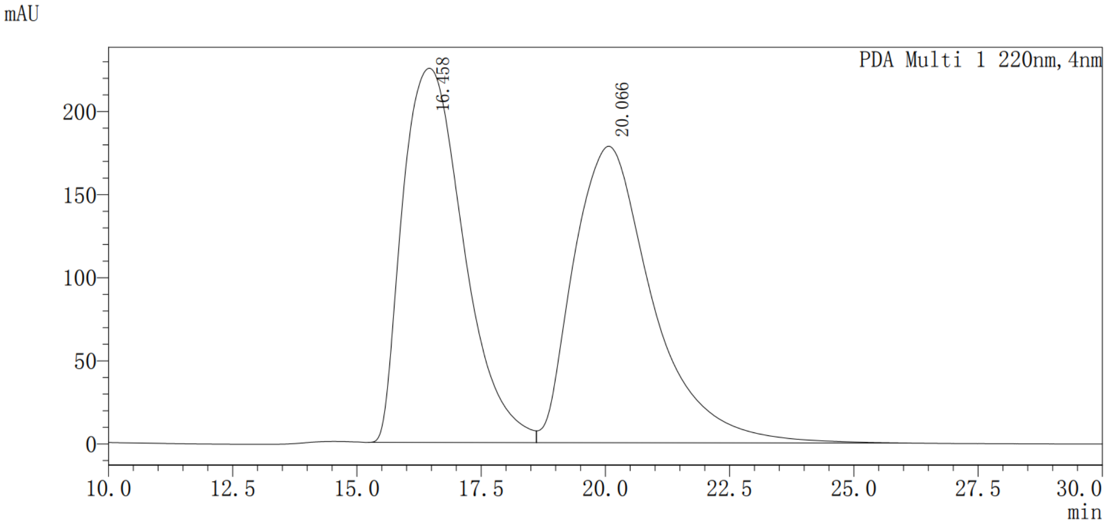

Peak	Ret Time[min]	Area[mAU*s]	Height [mAU]	Area %
1	16. 458	19304580	225169	49. 411
2	20. 066	19764445	178368	50. 589
Total		39069025	403537	100. 000

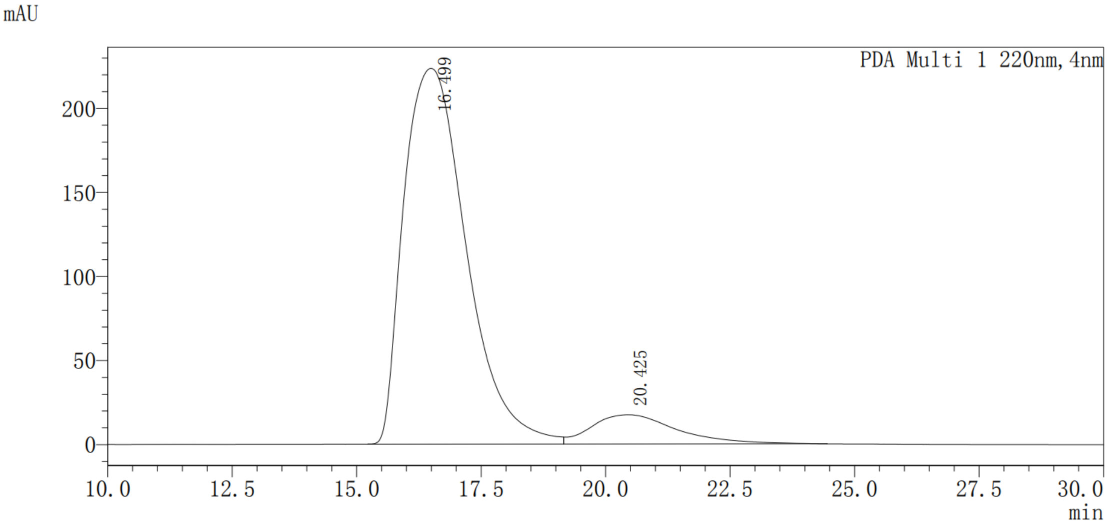

Peak	Ret Time[min]	Area[mAU*s]	Height [mAU]	Area %
1	16. 499	19552859	223523	90. 409
2	20. 425	2074179	17297	9. 591
Total		21627038	240820	100. 000

Supplementary Figure 73**. HPLC chromatography for 3aw**

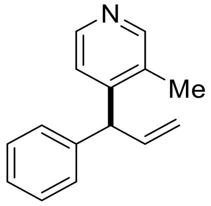

(R)-3-methyl-4-(1-phenylallyl)pyridine **(3ax)**

Following the general procedure, the product **3ax** was isolated by chromatography on silica gel (PE/EA = 10/1 ~ 5/1, eluent) as a yellow oil (52%, 21.7 mg); $[\alpha]_D^{25}$ = 18.7 (c 1.2, $CHCl_3$); ^{1}H NMR (400 MHz, $CDCl_3$) δ 8.39 (d, J = 5.0 Hz, 1H), 8.36 (s, 1H), 7.33 - 7.28 (m, 2H), 7.24 (s, 1H), 7.11 (d, J = 7.3 Hz, 2H), 7.04 (d, J = 5.0 Hz, 1H), 6.23 (ddd, J = 16.9, 10.1, 6.7 Hz, 1H), 5.28 (d, J = 10.2 Hz, 1H), 4.89 (d, J = 16.7 Hz, 2H), 2.22 (s, 3H). ^{13}C NMR (100 MHz, $CDCl_3$) δ 150.98, 150.07, 147.67, 140.67, 138.60, 128.77, 128.56, 126.80, 126.46, 123.05, 117.47, 50.75, 16.38. **HRMS (ESI)** m/z: $[M+H]^+$ Calcd For $C_{15}H_{16}N$: 210.1283; Found: 210.1287.

Analysis of Stereochemistry:

Enantiomeric excess: 87%, determined by HPLC (Daicel Chiralpak ADH, hexane/isopropanol = 98/2, flow rate 1.0 mL/min, T = 25 °C, 220nm): t_R= 13.49 min (minor), t_R= 16.48 min (major).

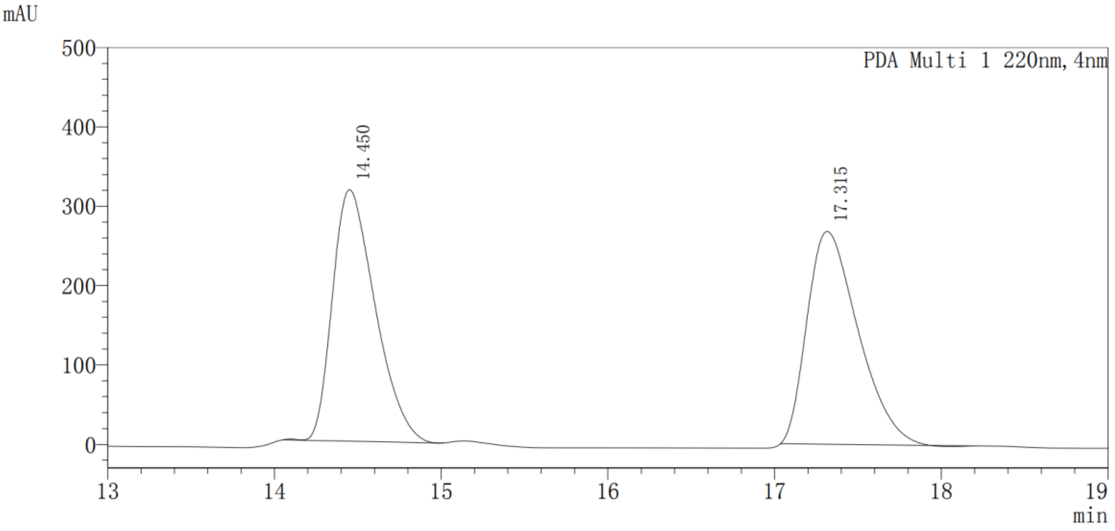

Peak	Ret Time[min]	Area[mAU*s]	Height [mAU]	Area %
1	14.450	5637155	316619	49.763
2	17.315	5690766	267906	50.237
Total		11327921	584525	100.000

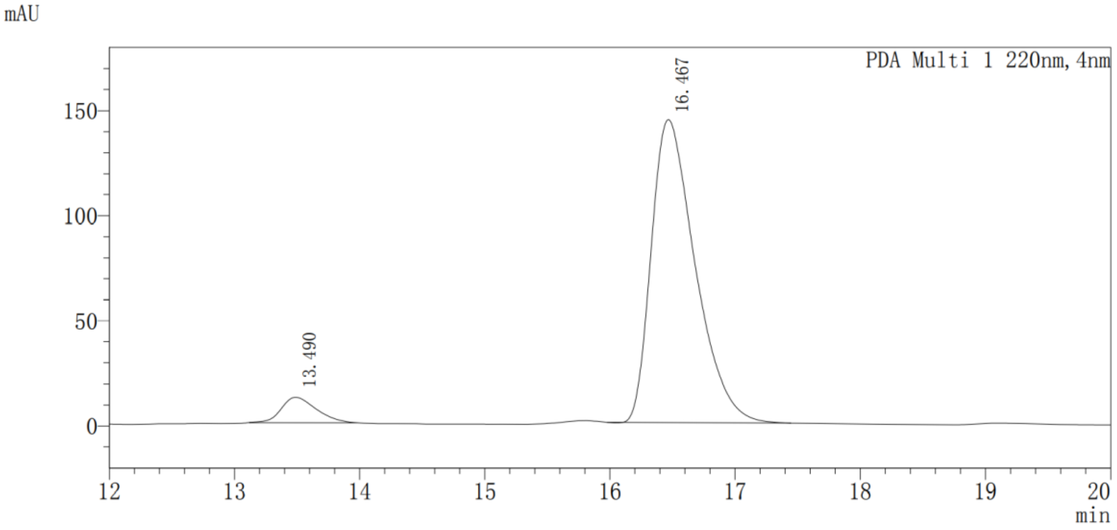

Peak Ret Time[min] Area[mAU*s] Height [mAU] Area %

1	13.490	236269	12098	6.313
2	16.467	3506600	144121	93.687
Total		3742869	156219	100.000

Supplementary Figure 74. HPLC chromatography for 3ax

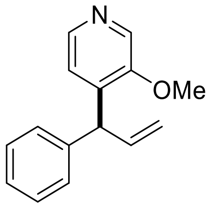

(R)-3-methoxy-4-(1-phenylallyl)pyridine **(3ay)**

Following the general procedure, the product **3ay** was isolated by chromatography on silica gel (PE/EA = 10/1 ~ 5/1, eluent) as a yellow oil (35%, 15.8 mg); $[\alpha]_D^{25}$ = 21.2 (c 0.7, $CHCl_3$); ^1H NMR (400 MHz, $CDCl_3$) δ 8.24 (s, 1H), 8.20 (d, *J* = 4.8 Hz, 1H), 7.29 (t, *J* = 7.3 Hz, 2H), 7.22 (d, *J* = 7.2 Hz, 1H), 7.19 - 7.14 (m, 2H), 7.05 (d, *J* = 4.8 Hz, 1H), 6.24 (ddd, *J* = 17.0, 10.2, 6.7 Hz, 1H), 5.24 (dd, *J* = 10.2, 1.1 Hz, 1H), 5.13 (d, *J* = 6.7 Hz, 1H), 4.94 (dt, *J* = 17.1, 1.3 Hz, 1H), 3.86 (s, 3H).^{13}C NMR (100 MHz, $CDCl_3$) δ 153.30, 142.69, 141.35, 140.24, 138.64, 133.53, 128.54, 128.35, 126.56, 123.59, 117.03, 77.32, 77.00, 76.68, 56.19, 47.05. **HRMS (ESI)** m/z: $[M+H]^+$ Calcd For $C_{15}H_{16}NO$: 226.1232; Found: 226.1231.

Analysis of Stereochemistry:

Enantiomeric excess: 91%, determined by HPLC (Daicel Chiralpak ADH, hexane/isopropanol = 98/2, flow rate 1.0 mL/min, T = 25 °C, 220nm): t_R= 15.15 min (minor), t_R= 17.24 min (major).

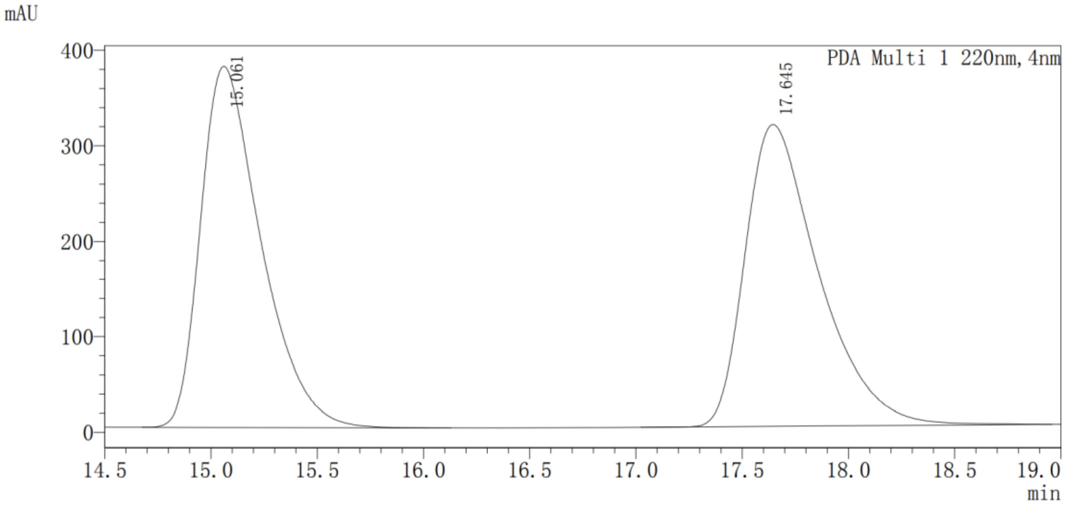

Peak	Ret Time[min]	Area[mAU*s]	Height [mAU]	Area %
1	15.061	7478337	378318	49.526
2	17.645	7621347	315867	50.474
Total		15099684	694185	100.000

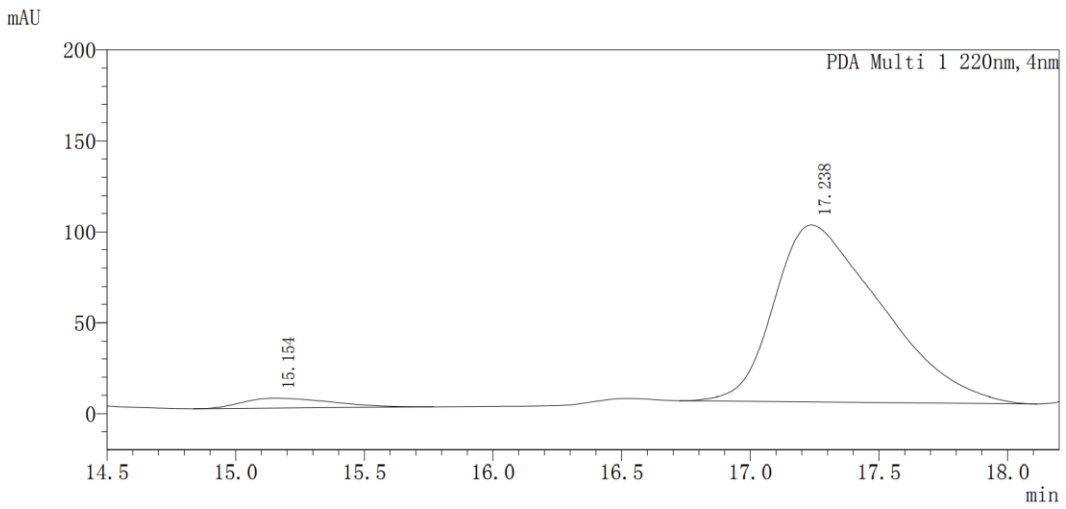

Peak	Ret Time[min]	Area[mAU*s]	Height [mAU]	Area %
1	15.154	130470	5458	4.413
2	17.238	2825699	97363	95.587
Total		2956169	102821	100.000

Supplementary Figure 75. **HPLC chromatography for 3ay**

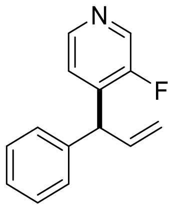

(R)-3-fluoro-4-(1-phenylallyl)pyridine **(3az)**

Following the general procedure, the product **3az** was isolated by chromatography on silica gel (PE/EA = 10/1 ~ 5/1, eluent) as a yellow oil (52%, 22.2 mg); $[\alpha]_D^{25}$ = 22.7 (c 0.6, $CHCl_3$); ^{1}H NMR (400 MHz, $CDCl_3$) δ 8.42 (d, J = 1.7 Hz, 1H), 8.37 (d, J = 4.9 Hz, 1H), 7.38 - 7.32 (m, 2H), 7.29 - 7.26 (m, 1H), 7.24 - 7.18 (m, 2H), 7.14 (dd, J = 5.9, 5.3 Hz, 1H), 6.27 (ddd, J = 17.1, 10.2, 6.8 Hz, 1H), 5.34 (d, J = 10.2 Hz, 1H), 5.06 (dd, J = 13.9, 12.8 Hz, 2H). ^{13}C NMR (100 MHz, $CDCl_3$) δ 157.7 (d, $J_{C\text{-}F}$ = 258.2Hz), 145.78 (d, $J_{C\text{-}F}$ = 5.1 Hz), 140.19, 138.84 (d, $J_{C\text{-}F}$ = 14.3 Hz), 138.11 (d, $J_{C\text{-}F}$ = 24.9 Hz), 137.40, 128.68, 128.36, 127.10, 124.21 (d, $J_{C\text{-}F}$ = 1.3 Hz), 117.88, 47.16 (d, $J_{C\text{-}F}$ = 2.0 Hz). ^{19}F NMR (376 MHz, $CDCl_3$) δ -131.53. **HRMS (ESI)** m/z: $[M+H]^+$ Calcd For $C_{14}H_{13}FN$: 214.1032; Found: 214.1032.

Analysis of Stereochemistry:

Enantiomeric excess: 91%, determined by HPLC (Daicel Chiralpak ADH, hexane/isopropanol = 98/2, flow rate 1.0 mL/min, T = 25 °C, 220nm): t_R= 7.36 min (minor), t_R= 7.88 min (major).

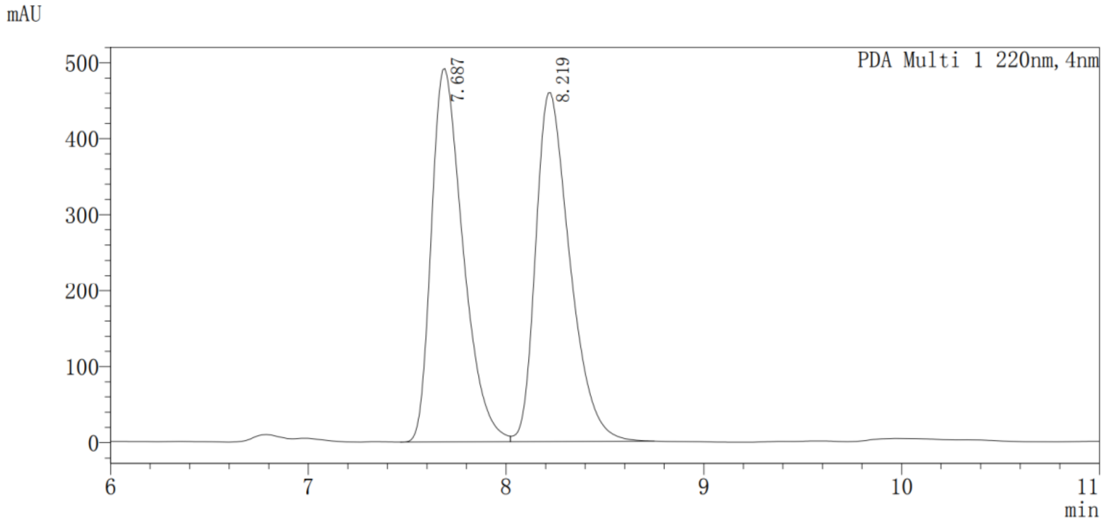

Peak	Ret Time[min]	Area[mAU*s]	Height [mAU]	Area %
1	7. 687	5474345	491813	49. 748
2	8. 219	5529829	459697	50. 252
Total		11004174	951510	100. 000

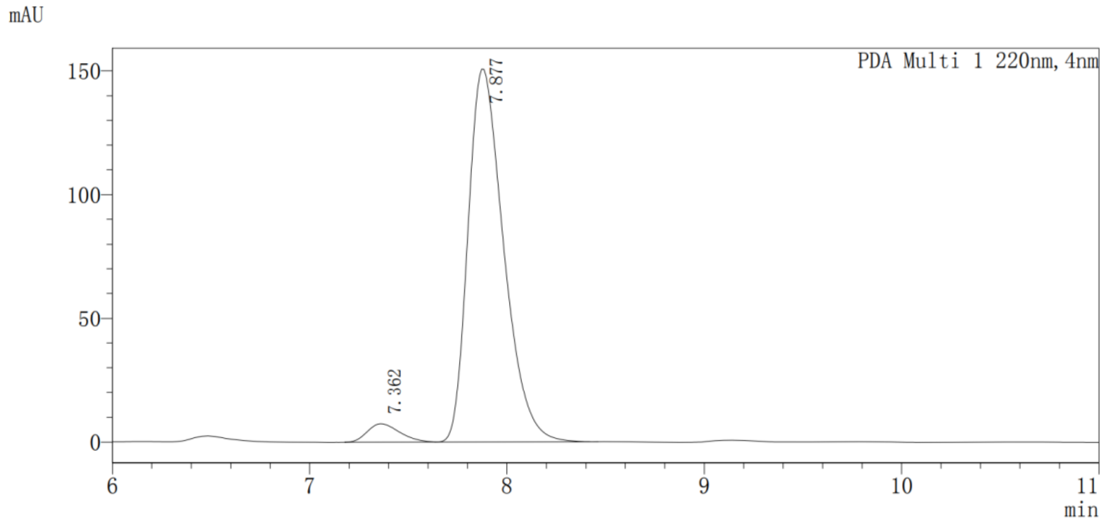

Peak	Ret Time[min]	Area[mAU*s]	Height [mAU]	Area %
1	7. 362	85200	7452	4. 312
2	7. 877	1890879	150649	95. 688
Total		1976080	158100	100. 000

Supplementary Figure 76. **HPLC chromatography for 3az**

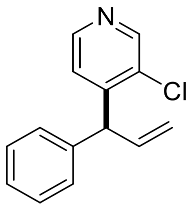

(R)-3-chloro-4-(1-phenylallyl)pyridine **(3ba)**

Following the general procedure, the product **3ba** was isolated by chromatography on silica gel (PE/EA = 10/1 ~ 5/1, eluent) as a pale yellow oil (50%, 22.9 mg); $[\alpha]_D^{25}$ = 22.8 (c 0.8, $CHCl_3$); ^1H NMR (400 MHz, $CDCl_3$) δ 8.58 (s, 1H), 8.44 (d, *J* = 5.0 Hz, 1H), 7.35 (t, *J* = 7.3 Hz, 2H), 7.27 (d, *J* = 9.0 Hz, 1H), 7.19 (d, *J* = 7.2 Hz, 2H), 7.14 (d, *J* = 5.0 Hz, 1H), 6.24 (ddd, *J* = 16.9, 10.2, 6.5 Hz, 1H), 5.35 (d, *J* = 10.2 Hz, 1H), 5.20 (d, *J* = 6.2 Hz, 1H), 4.98 (d, *J* = 17.2 Hz, 1H). ^{13}C NMR (100 MHz, $CDCl_3$) δ 149.67, 149.33, 147.79, 139.94, 137.50, 132.19, 128.71, 128.62, 127.06, 124.49, 118.18, 50.47. **HRMS (ESI)** m/z: $[M+H]^+$ Calcd For $C_{14}H_{13}ClN$: 230.0737; Found: 230.0735.

Analysis of Stereochemistry:

Enantiomeric excess: 92%, determined by HPLC (Daicel Chiralpak ADH hexane/isopropanol = 98/2, flow rate 1.0 mL/min, T = 25 °C, 220nm): t_R= 7.83 min (minor), t_R= 8.91 min (major).

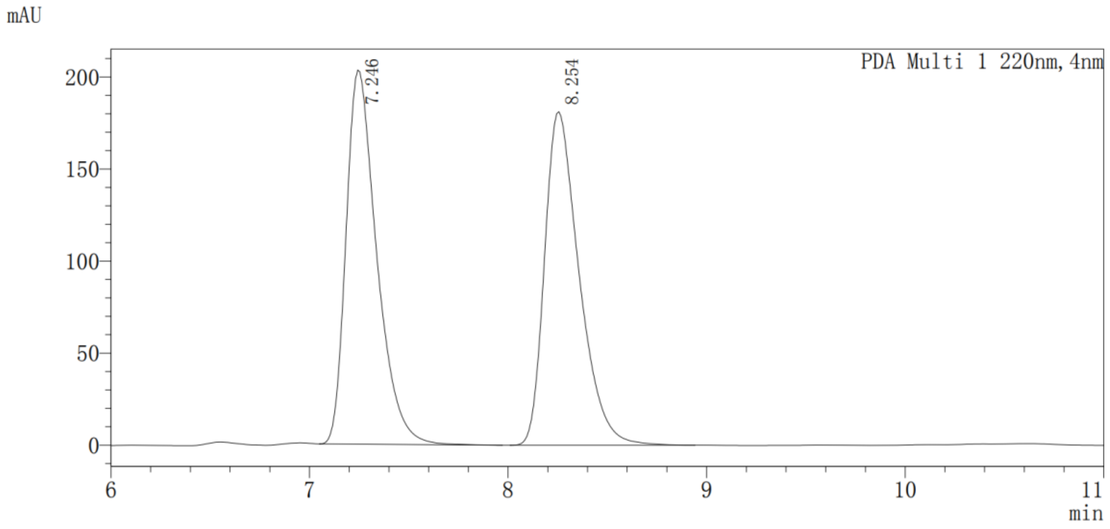

Peak	Ret Time[min]	Area[mAU*s]	Height [mAU]	Area %
1	7.246	2185393	203233	49.931
2	8.254	2191421	181148	50.069
Total		4376814	384381	100.000

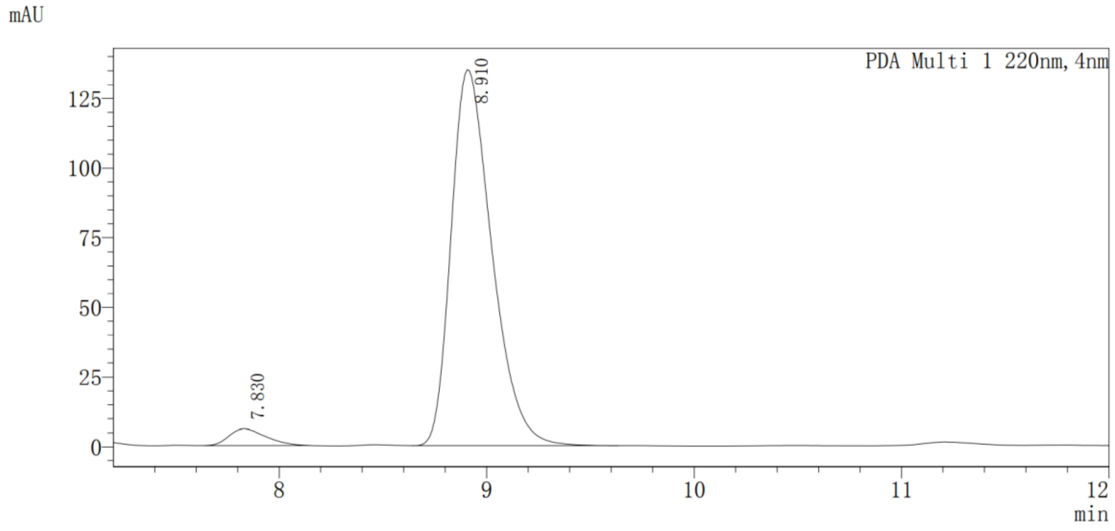

Peak	Ret Time[min]	Area[mAU*s]	Height [mAU]	Area %

1	7.830	73552	5975	3.842
2	8.910	1840857	135024	96.158
Total		1914409	140999	100.000

Supplementary Figure 77. **HPLC chromatography for 3ba**

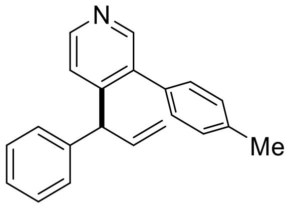

(R)-4-(1-phenylallyl)-3-(p-tolyl)pyridine **(3bb)**

Following the general procedure, the product **3bb** was isolated by chromatography on silica gel (PE/EA = 10/1 ~ 5/1, eluent) as a yellow oil (40%, 22.8 mg); $[\alpha]_D^{25}$ = 22.2 (c 0.8, $CHCl_3$); ^{1}H NMR (400 MHz, $CDCl_3$) δ 8.49 (d, J = 5.2 Hz, 1H), 8.47 (s, 1H), 7.26 (s, 2H), 7.21 (t, J = 8.3 Hz, 3H), 7.14 (dd, J = 6.4, 3.5 Hz, 3H), 7.03 (d, J = 7.3 Hz, 2H), 6.19 (ddd, J = 16.9, 10.2, 6.5 Hz, 1H), 5.27 (d, J = 10.2 Hz, 1H), 4.86 (t, J = 12.7 Hz, 2H), 2.41 (s, 3H). ^{13}C NMR (100 MHz, $CDCl_3$) δ 150.67, 149.38, 148.41, 142.05, 139.53, 137.73, 137.57, 134.54, 129.33, 129.02, 128.45, 128.41, 126.60, 123.72, 117.59, 50.02, 21.19. **HRMS (ESI)** m/z: $[M+H]^+$ Calcd For $C_{21}H_{20}N$: 286.1596; Found: 286.1595.

Analysis of Stereochemistry:

Enantiomeric excess: 80%, determined by HPLC (Daicel Chiralpak ADH, hexane/isopropanol = 95/5, flow rate 1.0 mL/min, T = 25 °C, 220nm): t_R= 16.21 min (minor), t_R= 17.17 min (major).

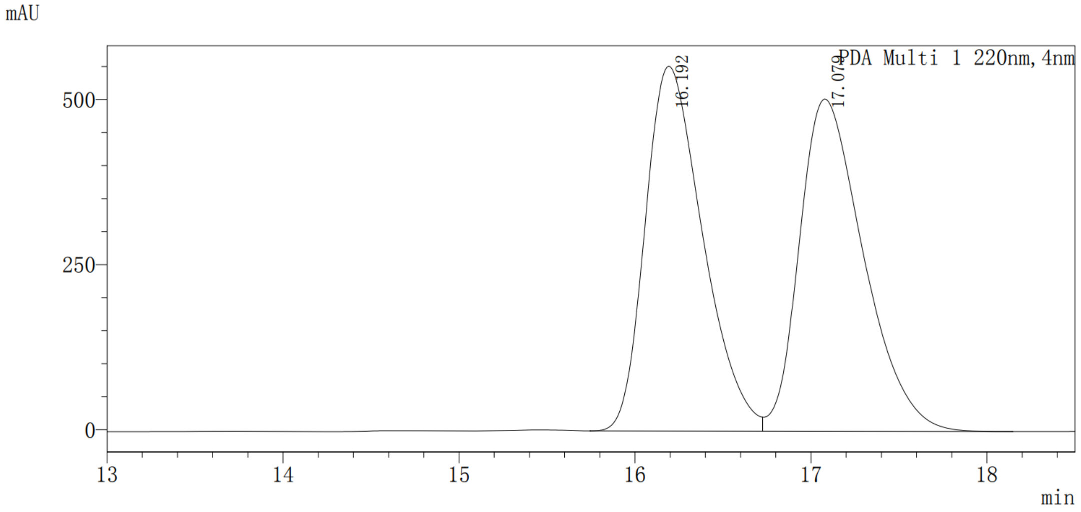

Peak	Ret Time[min]	Area[mAU*s]	Height [mAU]	Area %
1	16.192	12589614	552677	49.665
2	17.079	12759448	503164	50.335
Total		25349063	1055840	100.000

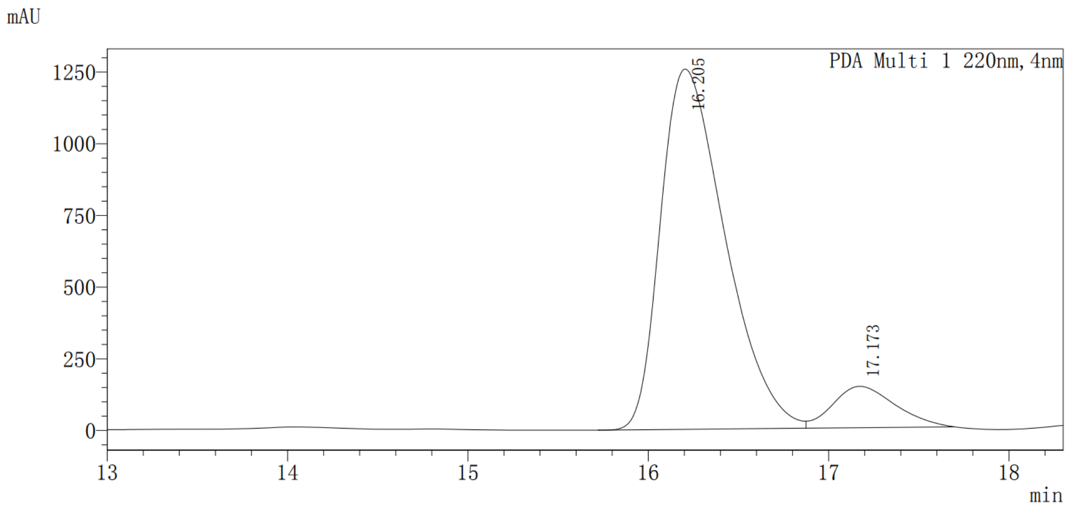

Peak	Ret Time[min]	Area[mAU*s]	Height [mAU]	Area %
1	16. 205	31313061	1256641	90. 010
2	17. 173	3475271	144353	9. 990
Total		34788331	1400994	100. 000

Supplementary Figure 78**. HPLC chromatography for 3bb**

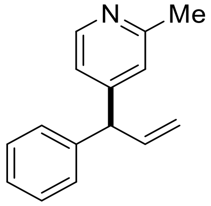

(R)-2-methyl-4-(1-phenylallyl)pyridine **(3bc)**

Following the general procedure, the product **3bc** was isolated by chromatography on silica gel (PE/EA = 10/1 ~ 5/1, eluent) as a yellow oil (45%, 18.8 mg); $[\alpha]_D^{25}$ = 21.0 (c 1.3, $CHCl_3$); ^1H NMR (400 MHz, $CDCl_3$) δ 8.40 (d, *J* = 4.9 Hz, 1H), 7.35 - 7.29 (m, 2H), 7.26 - 7.21 (m, 1H), 7.17 (dd, *J* = 5.3, 3.4 Hz, 2H), 6.98 (s, 1H), 6.92 (d, *J* = 5.0 Hz, 1H), 6.24 (ddd, *J* = 17.3, 10.2, 7.3 Hz, 1H), 5.27 (d, *J* = 10.2 Hz, 1H), 5.02 (d, *J* = 17.1 Hz, 1H), 4.65 (d, *J* = 7.3 Hz, 1H), 2.51 (s, 3H). ^{13}C NMR (100 MHz, $CDCl_3$) δ 158.48, 152.42, 149.12, 141.68, 139.06, 128.63, 128.52, 126.85, 123.31, 120.98, 117.37, 54.37, 24.40. **HRMS (ESI)** m/z: $[M+H]^+$ Calcd For $C_{15}H_{16}N$: 210.1283; Found: 210.1277.

Analysis of Stereochemistry:

Enantiomeric excess: 86%, determined by HPLC (Daicel Chiralpak ADH, hexane/isopropanol = 99.5/0.5, flow rate 1.0 mL/min, T = 25 °C, 220nm): t_R= 19.24 min (minor), t_R= 21.30 min (major).

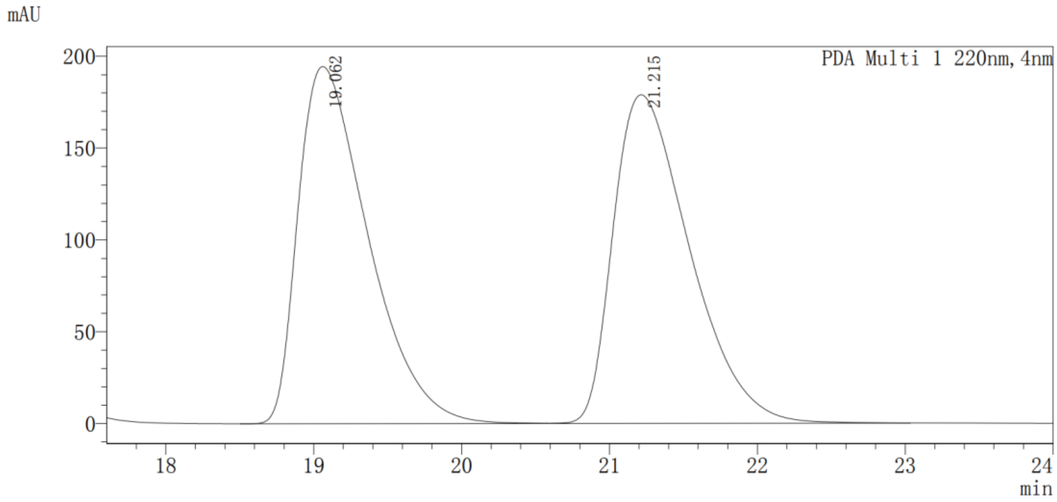

Peak	Ret Time[min]	Area[mAU*s]	Height [mAU]	Area %
1	19. 062	6409318	194588	49. 882
2	21. 215	6439651	178922	50. 118
Total		12848969	373511	100. 000

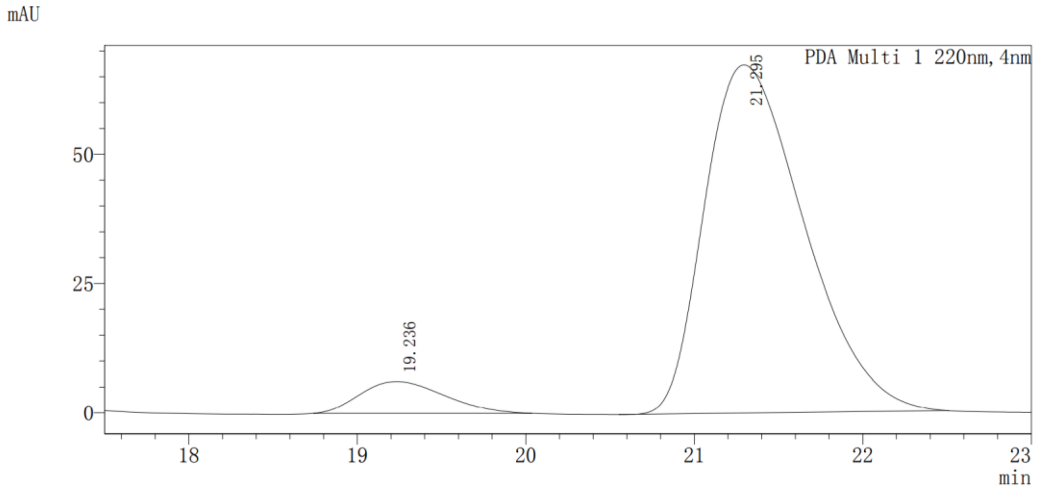

Peak	Ret Time[min]	Area[mAU*s]	Height [mAU]	Area %
1	19. 236	216070	6186	7. 234
2	21. 295	2770787	67364	92. 766
Total		2986856	73549	100. 000

Supplementary Figure 79**. HPLC chromatography for 3bc**

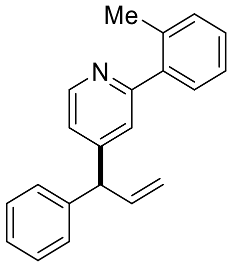

***(R)-4-(1-phenylallyl)-2-(o-tolyl) pyridine* (3bd)**

Following the general procedure, the product **3bd** was isolated by chromatography on silica gel (PE/EA = 10/1 ~ 5/1, eluent) as a yellow oil (42%, 23.9 mg); $[\alpha]_D^{25}$ = 20.7 (c 2.0, $CHCl_3$); ^1H NMR (400 MHz, $CDCl_3$) δ 8.60 (d, *J* = 5.0 Hz, 1H), 7.39 - 7.32 (m, 3H), 7.27 (dd, *J* = 5.8, 2.8 Hz, 3H), 7.25 - 7.19 (m, 4H), 7.08 (dd, *J* = 5.1, 1.3 Hz, 1H), 6.29 (ddd, *J* = 17.2, 10.2, 7.3 Hz, 1H), 5.31 (d, *J* = 10.2 Hz, 1H), 5.08 (d, *J* = 17.1 Hz, 1H), 4.75 (d, *J* = 7.2 Hz, 1H), 2.31 (s, 3H). ^{13}C NMR (100 MHz, $CDCl_3$) δ 160.01, 152.26, 149.23, 141.57, 140.45, 138.98, 135.71, 130.68, 129.60, 128.69, 128.54, 128.20, 126.92, 125.81, 124.26, 121.81, 117.53, 54.47, 20.24. **HRMS (ESI)** m/z: $[M+H]^+$ Calcd For $C_{21}H_{20}N$: 286.1596; Found: 286.1594.

Analysis of Stereochemistry:

Enantiomeric excess: 85%, determined by HPLC (Daicel Chiralpak ADH, hexane/isopropanol = 99/1, flow rate 1.0 mL/min, T = 25 °C, 220nm): t_R= 11.04 min (minor), t_R= 11.70 min (major).

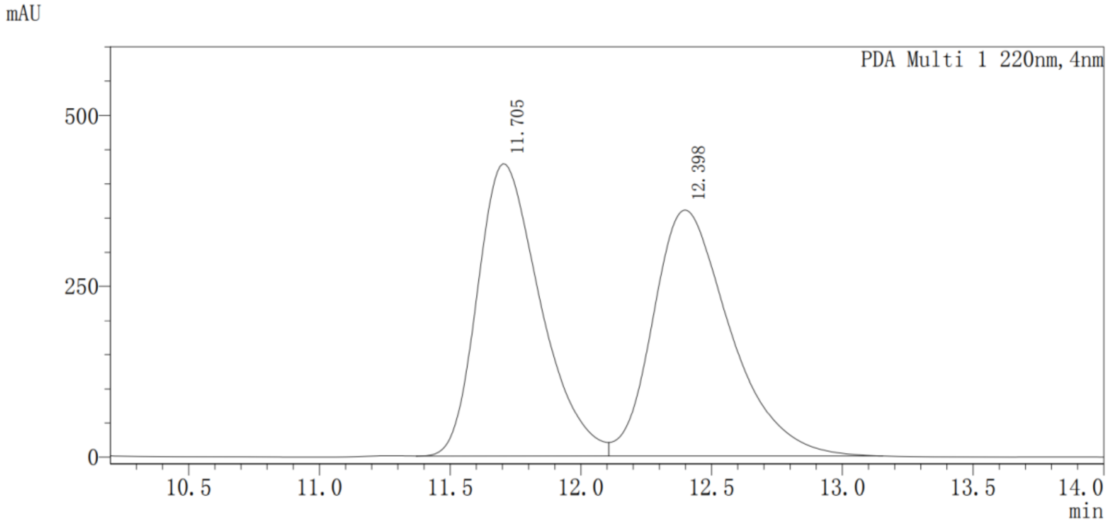

Peak	Ret Time[min]	Area[mAU*s]	Height [mAU]	Area %
1	11.705	7180351	427926	49.191
2	12.398	7416545	360278	50.809
Total		14596896	788204	100.000

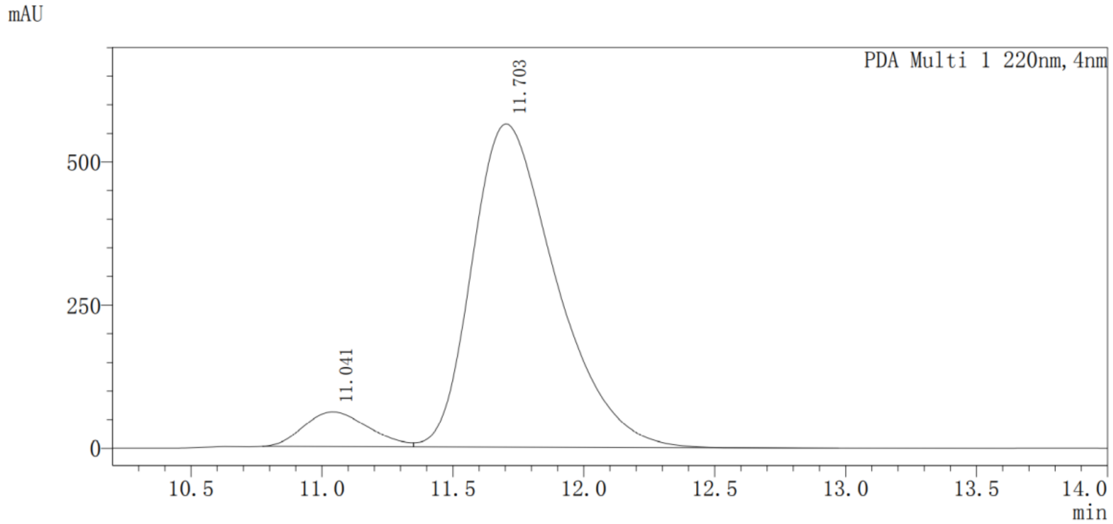

Peak	Ret Time[min]	Area[mAU*s]	Height [mAU]	Area %
1	11.041	1028992	59907	7.562
2	11.703	12578531	564480	92.438
Total		13607523	624387	100.000

Supplementary Figure 80. **HPLC chromatography for 3bd**

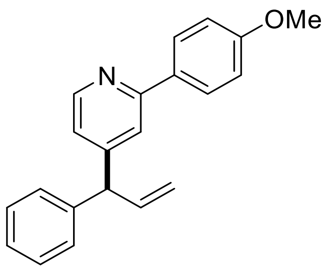

(R)-2-(4-methoxyphenyl)-4-(1-phenylallyl) pyridine **(3be)**

Following the general procedure, the product **3be** was isolated by chromatography on silica gel (PE/EA = 10/1 ~ 5/1, eluent) as a yellow oil (40%, 24.1 mg); $[\alpha]_D^{25}$ = 16.1 (c 1.4, $CHCl_3$); ^{1}H NMR (400 MHz, $CDCl_3$) δ 8.55 (d, *J* = 5.1 Hz, 1H), 7.93 - 7.86 (m, 2H), 7.50 (s, 1H), 7.34 (t, *J* = 7.3 Hz, 2H), 7.28 - 7.26 (m, 1H), 7.24 - 7.18 (m, 2H), 7.00 (dd, *J* = 5.2, 1.5 Hz, 1H), 6.99 - 6.95 (m, 2H), 6.34 - 6.25 (m, 1H), 5.31 (d, *J* = 10.2 Hz, 1H), 5.08 (d, *J* = 17.1 Hz, 1H), 4.75 (d, *J* = 7.2 Hz, 1H), 3.85 (s, 3H). ^{13}C NMR (100 MHz, $CDCl_3$) δ 160.46, 157.32, 152.74, 149.56, 141.63, 139.06, 132.09, 128.67, 128.57, 128.24, 126.89, 121.69, 119.99, 117.52, 114.08, 55.34, 54.59. **HRMS (ESI)** m/z: $[M+H]^+$ Calcd For $C_{21}H_{20}NO$: 302.1545; Found: 302.1536.

Analysis of Stereochemistry:

Enantiomeric excess: 85%, determined by HPLC (Agela Technologies Venusil CJ, hexane/isopropanol = 90/10, flow rate 1.0 mL/min, T = 25 °C, 220nm): t_R= 22.02 min (minor), t_R= 25.05 min (major).

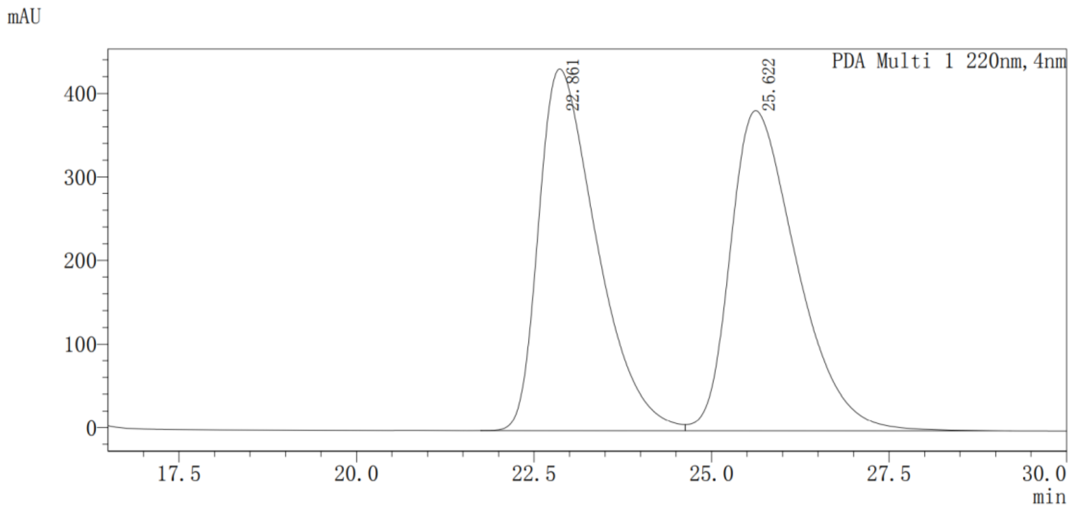

Peak Ret Time[min] Area[mAU*s] Height [mAU] Area %

1	22.861	24652906	432742	49.653
2	25.622	24997155	383351	50.347
Total		49650061	816093	100.000

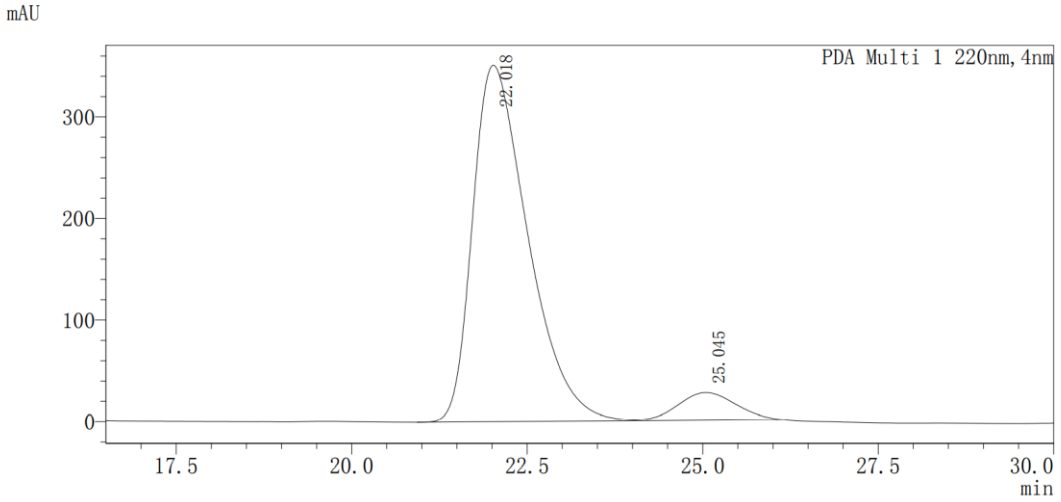

Peak	Ret Time[min]	Area[mAU*s]	Height [mAU]	Area %
1	22.018	19208668	350986	92.534
2	25.045	1549940	27181	7.466
Total		20758607	378167	100.000

Supplementary Figure 81. **HPLC chromatography for 3be**

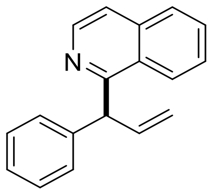

(R)-1-(1-phenylallyl)isoquinoline **(3bf)**

Following the general procedure, the product **3bf** was isolated by chromatography on silica gel (PE/EA = 10/1 ~ 5/1, eluent) as a yellow oil (49%, 24.0 mg); $[\alpha]_D^{25}$ = 31.8 (c 1.2, $CHCl_3$); ^{1}H NMR (400 MHz, $CDCl_3$) δ 8.57 (d, J = 5.7 Hz, 1H), 8.14 (d, J = 8.5 Hz, 1H), 7.80 (d, J = 8.2 Hz, 1H), 7.64 - 7.59 (m, 1H), 7.56 (d, J = 5.7 Hz, 1H), 7.52 - 7.46 (m, 1H), 7.31 - 7.26 (m, 4H), 7.18 (td, J = 5.6, 2.7 Hz, 1H), 6.78 - 6.66 (m, 1H), 5.64 (d, J = 7.3 Hz, 1H), 5.32 - 5.24 (m, 1H), 5.07 (dt, J = 17.2, 1.3 Hz, 1H). ^{13}C NMR (100 MHz, $CDCl_3$) δ 161.77, 142.31, 141.89, 140.37, 136.68, 129.66, 128.54, 128.37, 127.48, 127.14, 126.52, 125.24, 119.73, 115.71, 53.70. **HRMS (ESI)** m/z: $[M+H]^+$ Calcd For $C_{18}H_{16}N$: 246.1283; Found: 246.1279.

Analysis of Stereochemistry:

Enantiomeric excess: 87%, determined by HPLC (Agela Technologies Venusil CJ, hexane/isopropanol = 99/1, flow rate 1.0 mL/min, T = 25 °C, 220nm): t_R= 20.59 min (major), t_R= 26.37 min (minor).

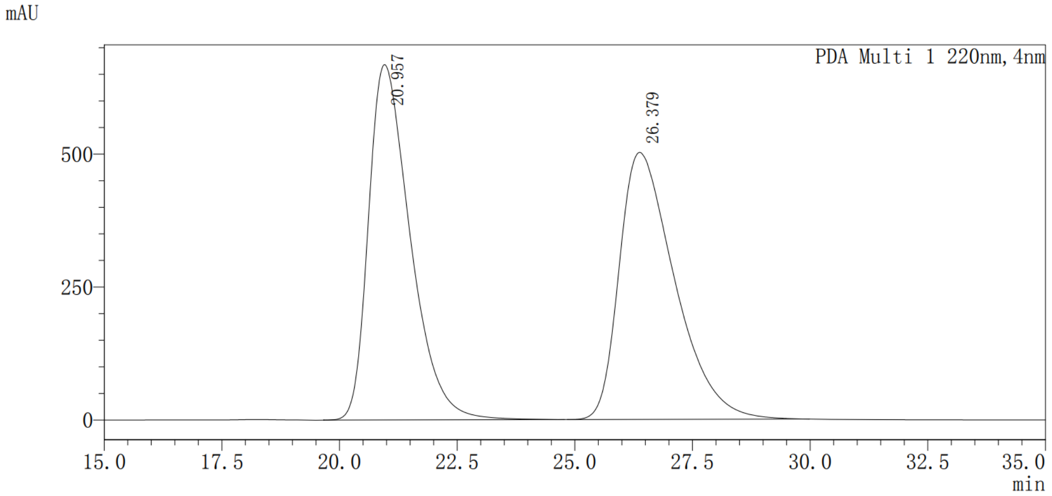

Peak	Ret Time[min]	Area[mAU*s]	Height [mAU]	Area %
1	20.957	41216805	668033	50.011
2	26.379	41199407	502144	49.989
Total		82416212	1170178	100.000

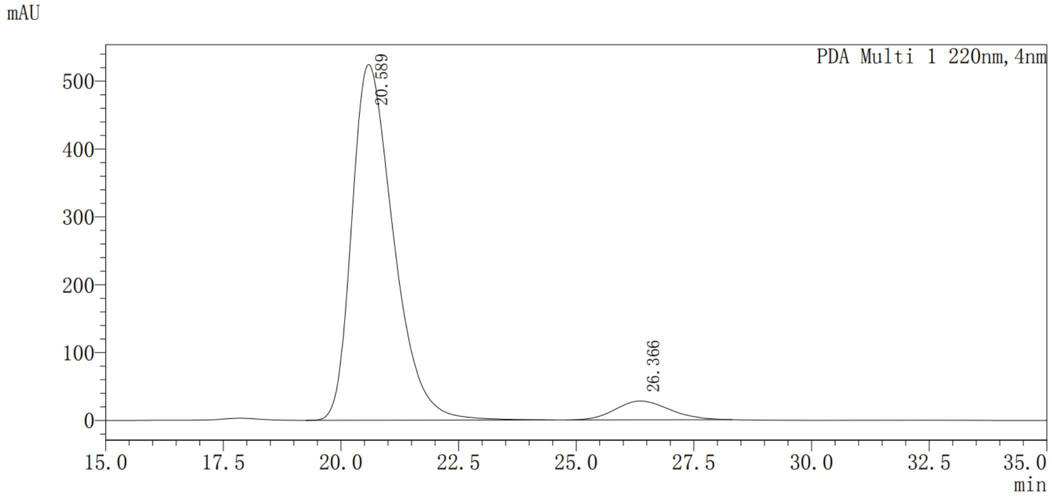

Peak	Ret Time[min]	Area[mAU*s]	Height [mAU]	Area %
1	20.589	32299373	524299	93.549
2	26.366	2227365	27739	6.451
Total		34526738	552038	100.000

Supplementary Figure 82. **HPLC chromatography for 3bf**

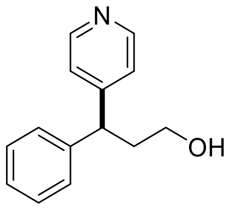

(R)-3-phenyl-3-(pyridin-4-yl)propan-1-ol **(S3)**

Following the general procedure, the product **S3** was isolated by chromatography on silica gel (PE/EA = 1/1 ~ 1/2, eluent) as a yellow oil (24.0 mg); $[\alpha]_D^{25}$ = 21.8 (c 1.1, $CHCl_3$); ^1H NMR (400

MHz, Chloroform-*d*) δ 8.50 – 8.42 (m, 2H), 7.30 (dd, *J* = 8.0, 6.8 Hz, 2H), 7.22 (dt, *J* = 5.9, 1.4 Hz, 3H), 7.19 – 7.14 (m, 2H), 4.16 (t, *J* = 7.9 Hz, 1H), 3.59 (t, *J* = 6.3 Hz, 2H), 2.30 (dt, *J* = 7.9, 6.3 Hz, 2H), 2.00 (s, 1H). ^{13}C NMR (100 MHz, Chloroform-*d*) δ 153.65, 149.77, 142.51, 128.77, 127.94, 126.88, 123.24, 60.30, 46.62, 37.42. **HRMS (ESI)** m/z: $[M+H]^+$ Calcd For $C_{14}H_{16}NO$: 214.1232; Found: 214.1234.

Analysis of Stereochemistry:

Enantiomeric excess: 91%, determined by HPLC (Agela Technologies Venusil CJ, hexane/isopropanol = 90/10, flow rate 1.0 mL/min, T = 25 °C, 220nm): t_R= 21.86 min (minor), t_R= 26.44 min (major).

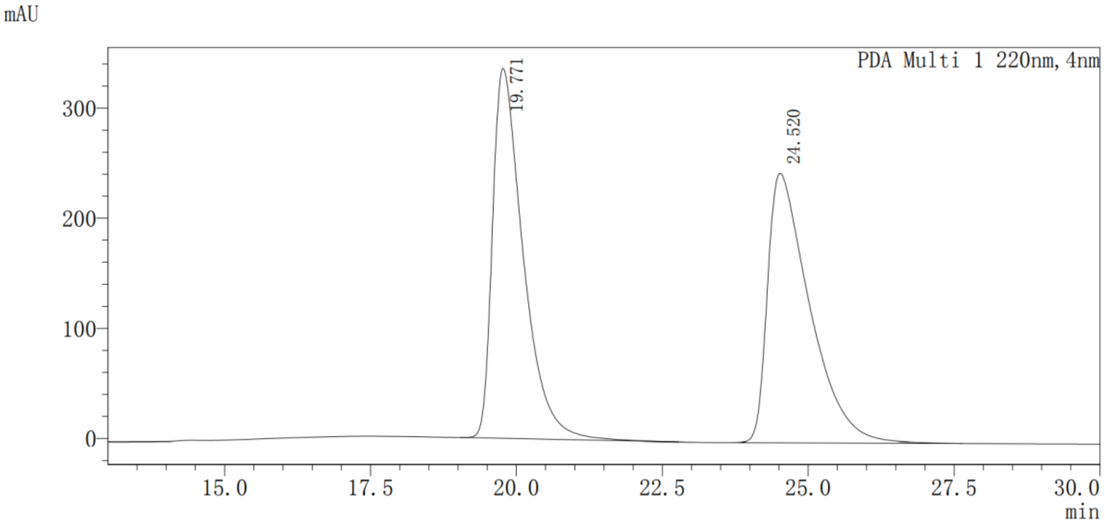

Peak	Ret Time[min]	Area[mAU*s]	Height [mAU]	Area %
1	19.771	12298789	335804	50.064
2	24.520	12267556	244403	49.936
Total		24566346	580208	100.000

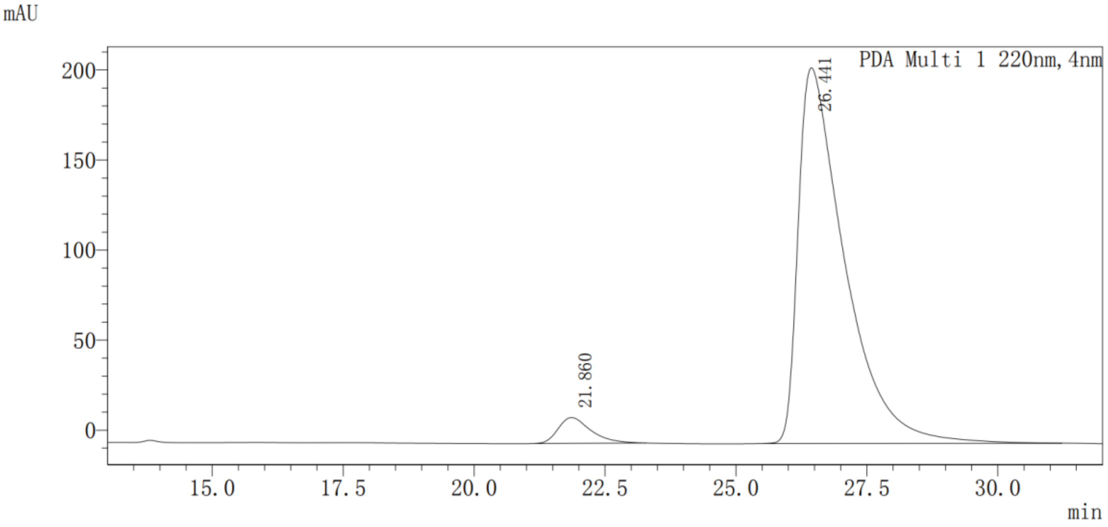

Peak	Ret Time[min]	Area[mAU*s]	Height [mAU]	Area %
1	21.860	627159	14375	4.631
2	26.441	12915179	208680	95.369
Total		13542338	223055	100.000

Supplementary Figure 83. **HPLC chromatography for S3**

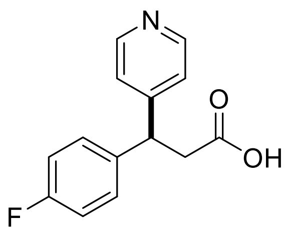

***(R)-3-(4-fluorophenyl)-3-(pyridin-4-yl) propanoic acid* (5)**

Following the above procedure, the product **5** was obtained as a white solid (49 mg); $[\alpha]_D^{25}$ = 33.9 (c 0.9, $CHCl_3$); ^{1}H NMR (400 MHz, DMSO) δ 8.44 (d, *J* = 6.0 Hz, 2H), 7.38 (dd, *J* = 8.6, 5.5 Hz, 2H), 7.35 (d, *J* = 6.0 Hz, 2H), 7.11 (t, *J* = 8.9 Hz, 2H), 4.45 (t, *J* = 7.9 Hz, 1H), 3.05 (ddd, *J* = 35.0, 16.2, 8.0 Hz, 2H). ^{13}C NMR (100 MHz, DMSO) δ 172.83, 162.40 (d, $J_{C\text{-}F}$ = 243.4 Hz), 153.20, 150.09, 139.49 (d, $J_{C\text{-}F}$ = 3.0 Hz), 129.99 (d, $J_{C\text{-}F}$ = 8.0 Hz), 123.35, 115.79, 115.58, 45.67, 39.47. ^{19}F NMR (376 MHz, DMSO) δ -116.30. **HRMS (ESI)** m/z: $[M+H]^+$ Calcd for $C_{14}H_{13}FNO_2$ 246.0930; Found 246.0931.

Analysis of Stereochemistry:

Enantiomeric excess: 87%, determined by HPLC (Daicel Chiralpak ADH, hexane/isopropanol = 80/20, flow rate 1.0 mL/min, T = 25 °C, 220nm): t_R= 8.52 min (minor), t_R= 13.79 min (major).

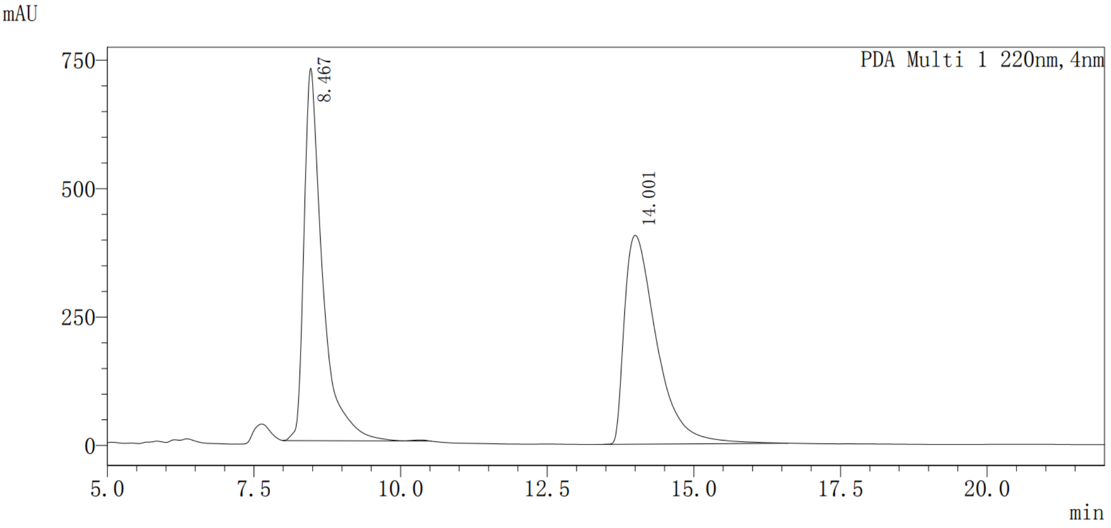

Peak	Ret Time[min]	Area[mAU*s]	Height [mAU]	Area %
1	8.467	725021	15919170	50.541
2	14.001	406519	15578593	49.459
Total		1131540	31497763	100.000

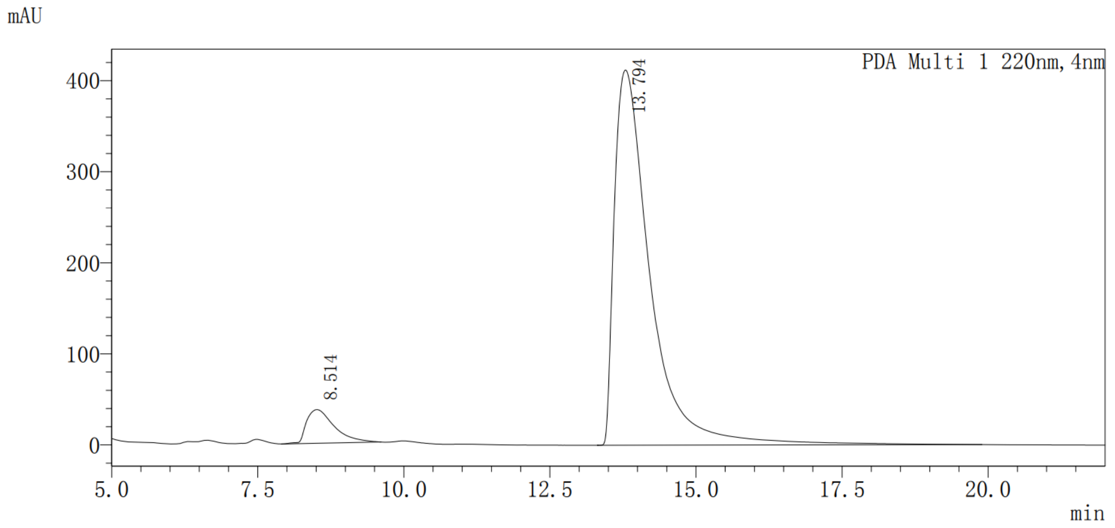

Peak	Ret Time[min]	Area[mAU*s]	Height [mAU]	Area %
1	8.514	36968	1224170	6.487
2	13.794	412026	17645732	93.513
Total		448993	18869902	100.000

Supplementary Figure 84. **HPLC chromatography for 5**

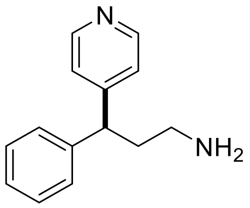

***(R)-3-phenyl-3-(pyridin-4-yl) propan-1-amine* (6)**

Following the general procedure, the product **6** obtained as a pale yellow oil (70 mg); a pale yellow oil; $[\alpha]_D^{25}$ = 32.7 (c 1.2, $CHCl_3$); ^{1}H NMR (400 MHz, $CDCl_3$) δ 8.47 (d, J = 5.9 Hz, 2H), 7.32 – 7.27 (m, 2H), 7.23 – 7.19 (m, 3H), 7.16 (d, J = 6.0 Hz, 2H), 4.02 (t, J = 7.8 Hz, 1H), 2.66 (t, J = 7.0 Hz, 2H), 2.19 (dd, J = 14.5, 7.6 Hz, 2H), 1.93 (s, 2H). ^{13}C NMR (100 MHz, $CDCl_3$) δ 153.67, 149.84, 142.69, 128.74, 127.83, 126.82, 123.11, 48.04, 40.14, 38.44. **HRMS (ESI)** m/z: $[M+H]^+$ Calcd For $C_{14}H_{17}N_2$: 213.1392; Found: 213.1392.

Analysis of Stereochemistry:

The title compound and the analogous racemic material were subjected to acetylation for HPLC analysis as shown below.

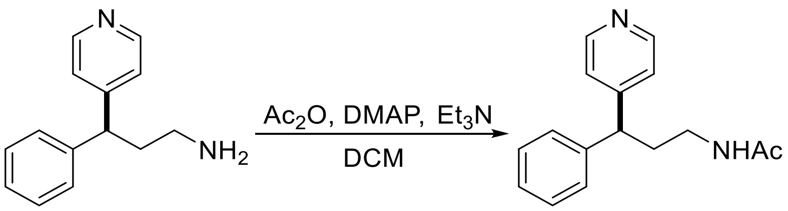

Enantiomeric excess: 92%, determined by HPLC (Daicel Chiralpak ASH, hexane/isopropanol = 90/10, flow rate 1.0 mL/min, T = 25 °C, 220nm): t_R= 22.76 min (minor), t_R= 28.08 min (major).

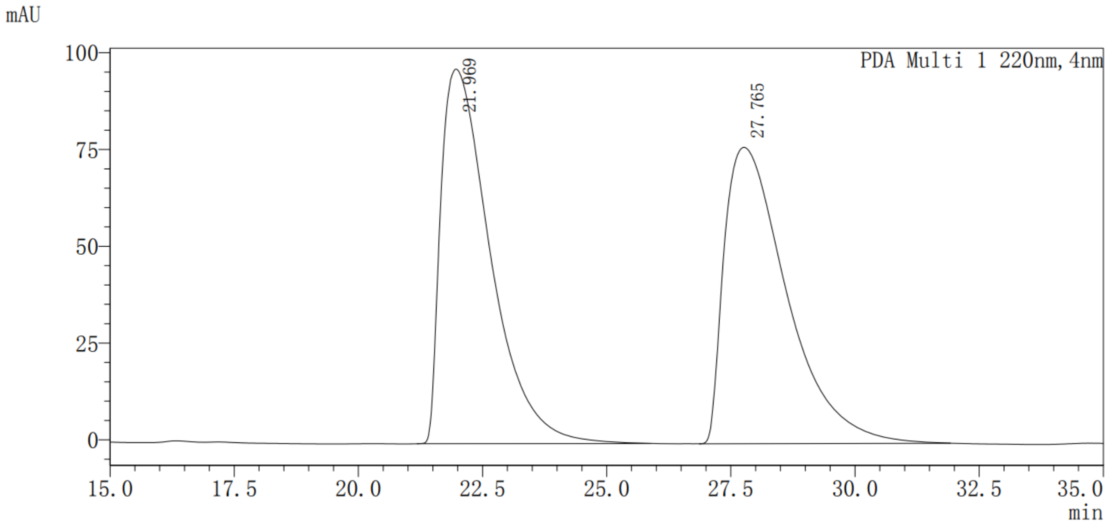

Peak	Ret Time[min]	Area[mAU*s]	Height [mAU]	Area %
1	21.969	96745	6724801	49.985
2	27.765	76552	6728738	50.015
Total		173297	13453539	100.000

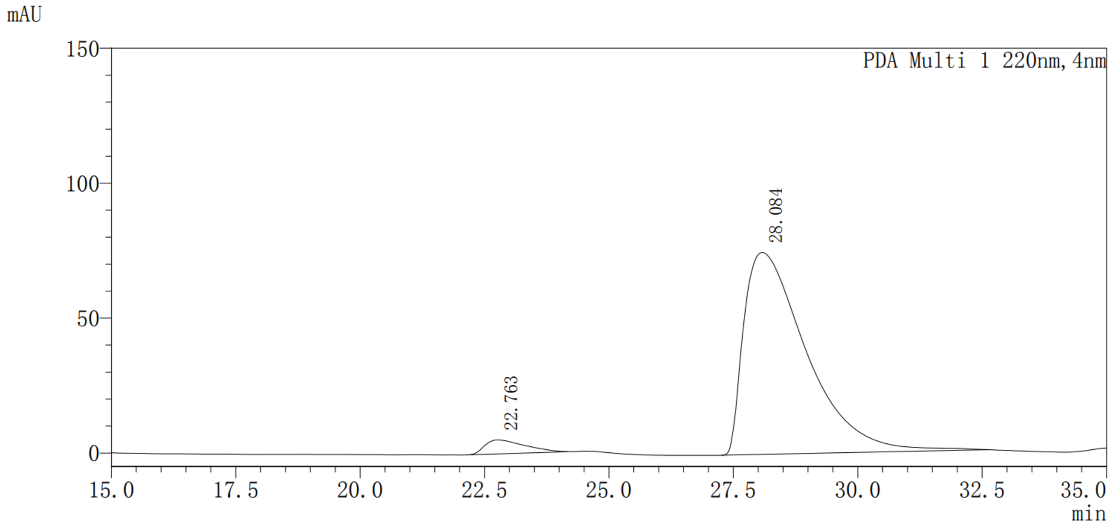

Peak	Ret Time[min]	Area[mAU*s]	Height [mAU]	Area %
1	22.763	5215	288151	4.195
2	28.084	74810	6581011	95.805
Total		80025	6869162	100.000

Supplementary Figure 85. **HPLC chromatography for S5**

3.2 NMR spectra for new compounds

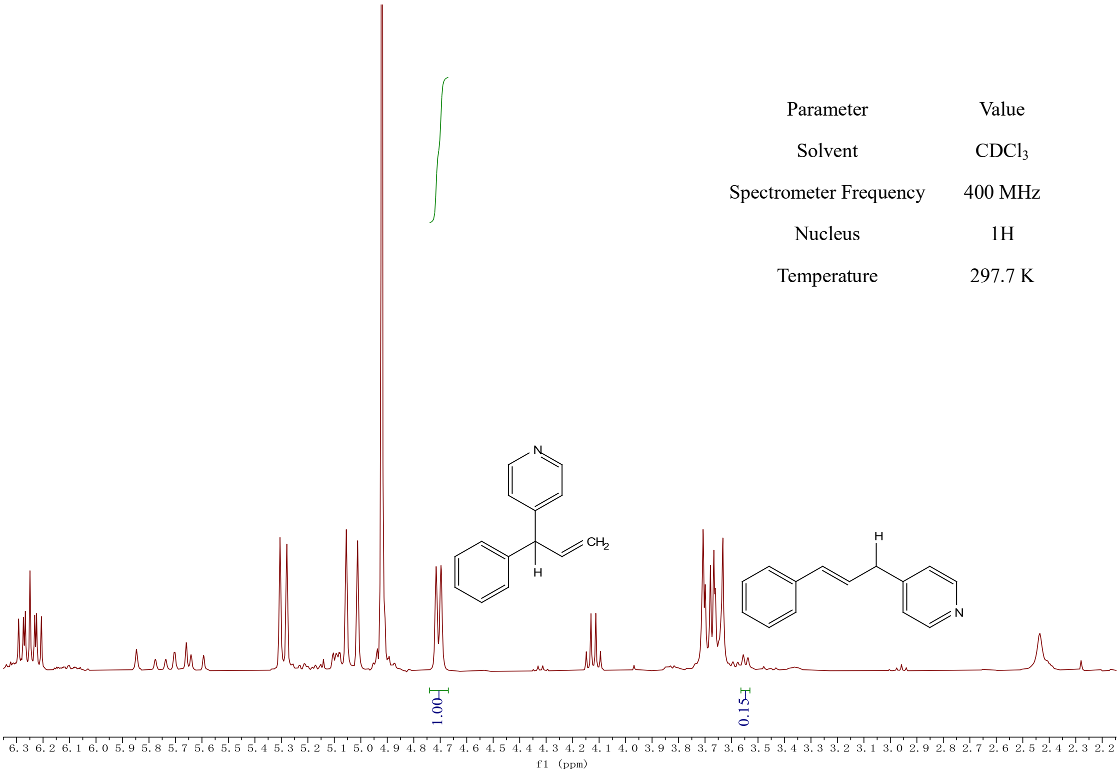

^{1}H NMR Spectrum of Crude Product **3a**

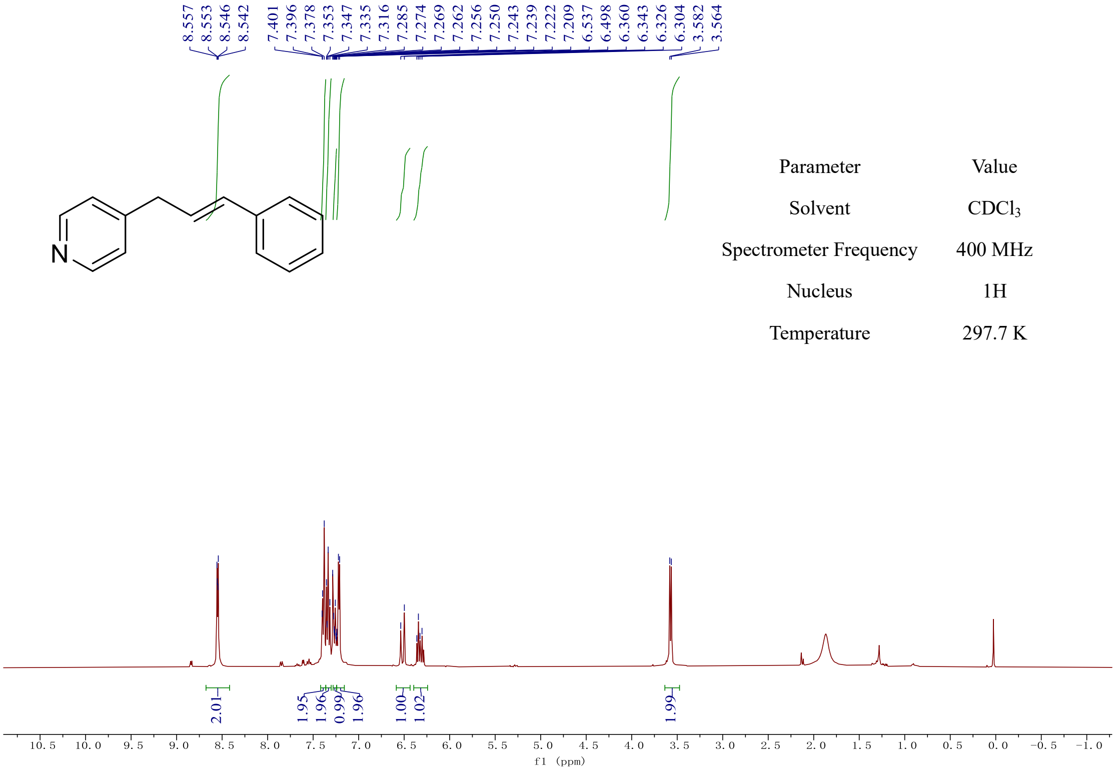

^{1}H NMR Spectrum of Pure *Linear* Product **3a'**

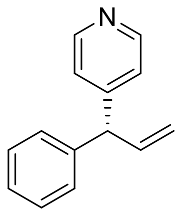

3a

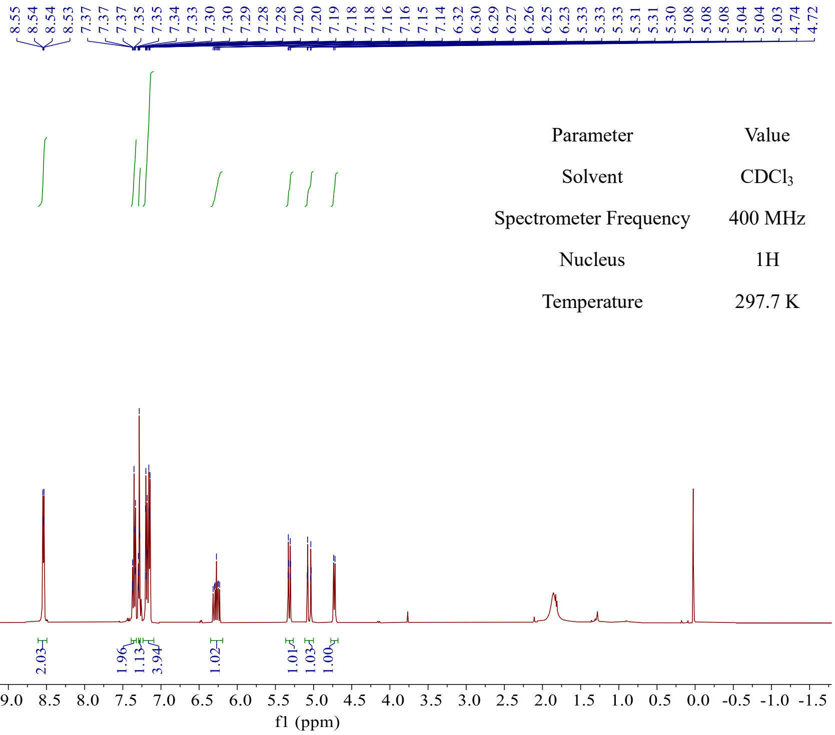

Parameter	Value
Solvent	$CDCl_3$
Spectrometer Frequency	400 MHz
Nucleus	1H
Temperature	297.7 K

^{1}H NMR Spectrum of Compound **3a**

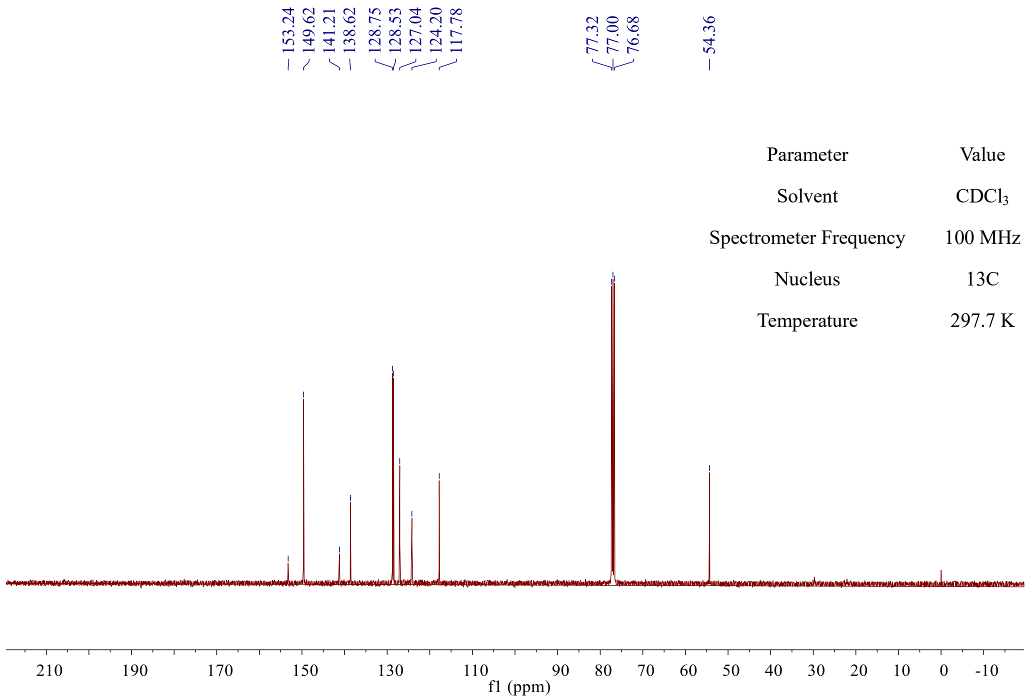

Parameter	Value
Solvent	$CDCl_3$
Spectrometer Frequency	100 MHz
Nucleus	13C
Temperature	297.7 K

^{13}C NMR Spectrum of Compound **3a**

Supplementary Figure 86**. NMR spectra of 3a**

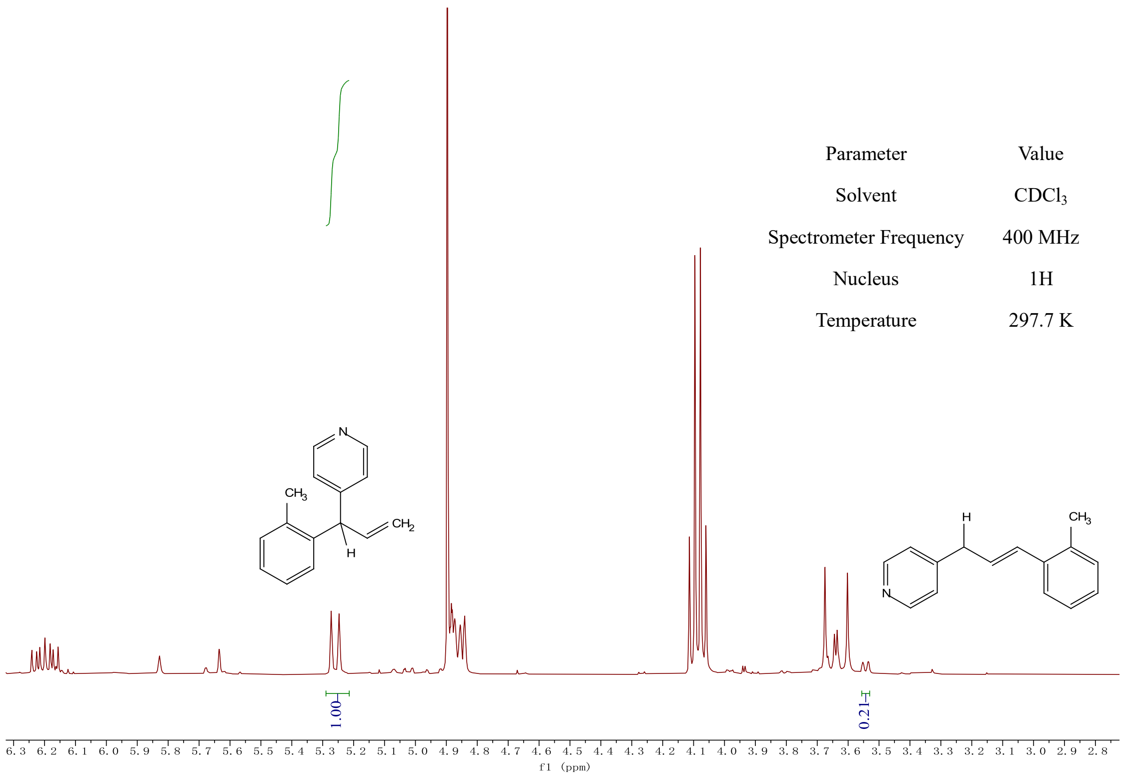

Parameter	Value
Solvent	$CDCl_3$
Spectrometer Frequency	400 MHz
Nucleus	1H
Temperature	297.7 K

^{1}H NMR Spectrum of Crude Product **3b**

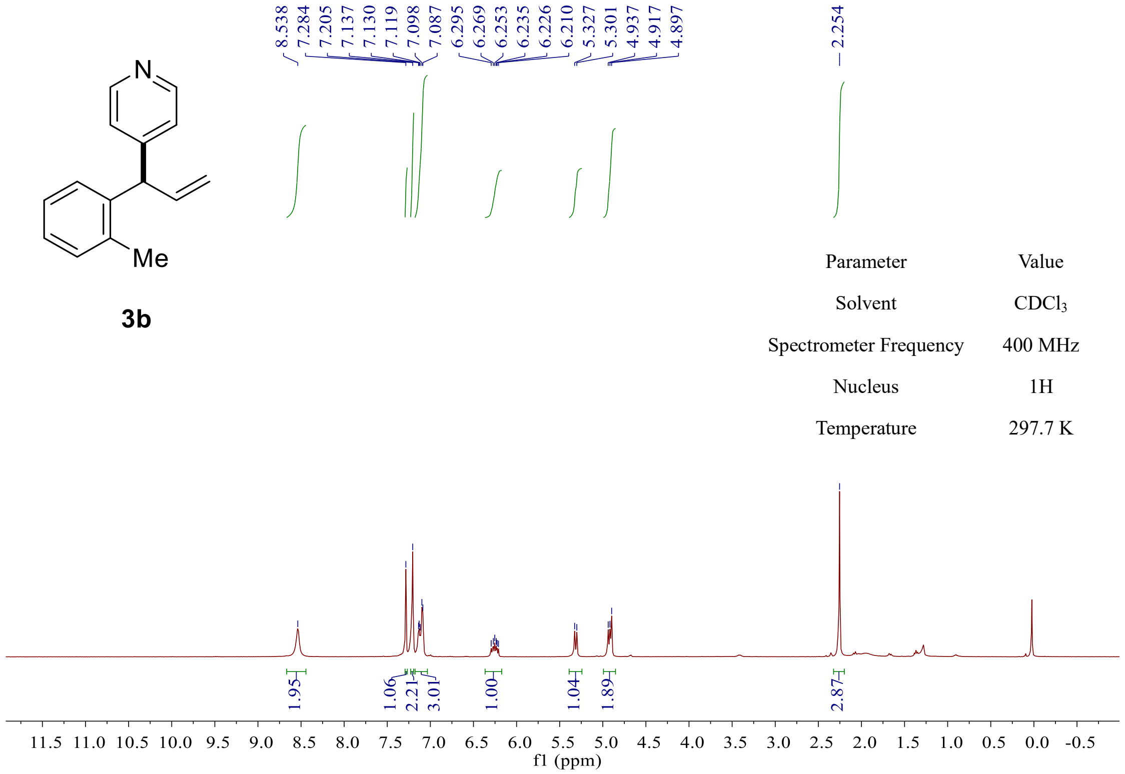

3b

Parameter	Value
Solvent	$CDCl_3$
Spectrometer Frequency	400 MHz
Nucleus	1H
Temperature	297.7 K

^{1}H NMR Spectrum of Compound **3b**

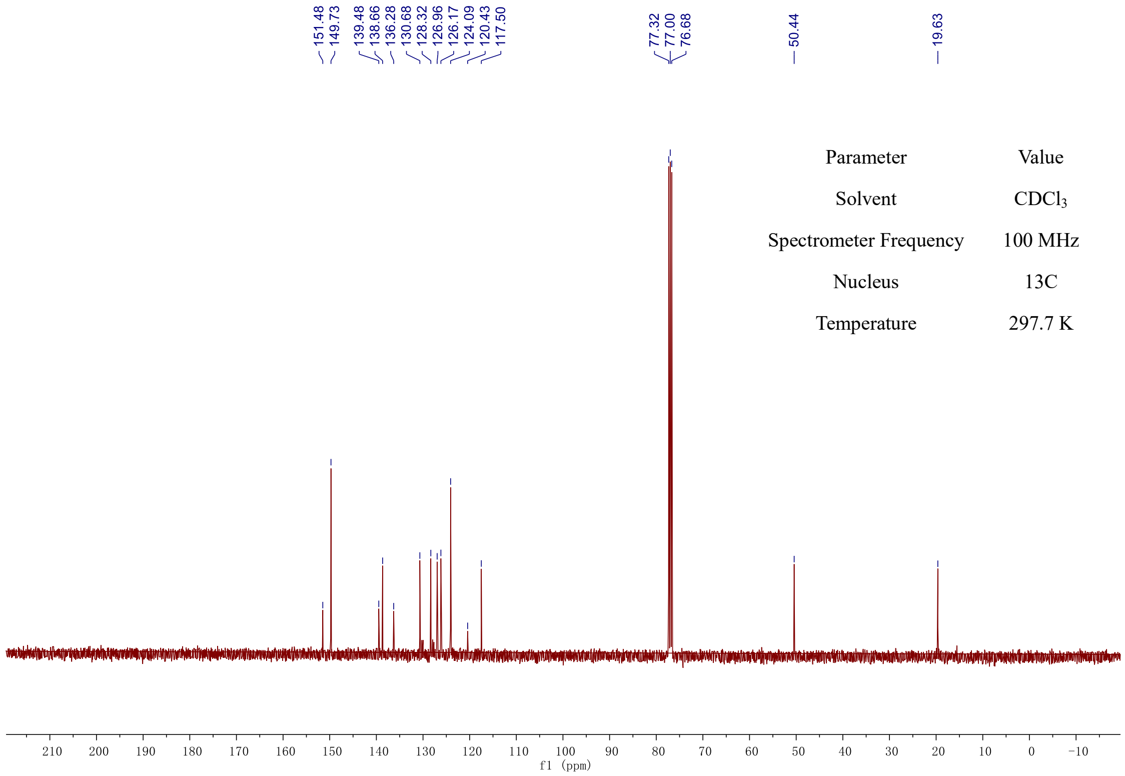

^{13}C NMR Spectrum of Compound **3b**

Supplementary Figure 87**. NMR spectra of 3b**

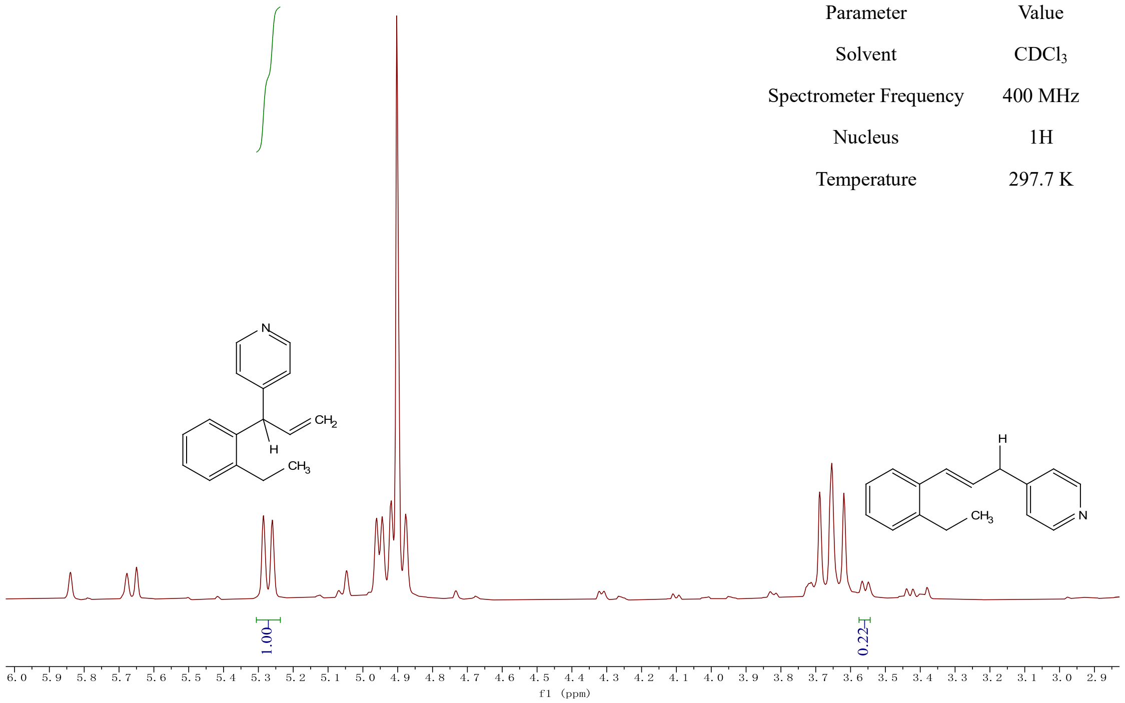

^{1}H NMR Spectrum of Crude Product **3c**

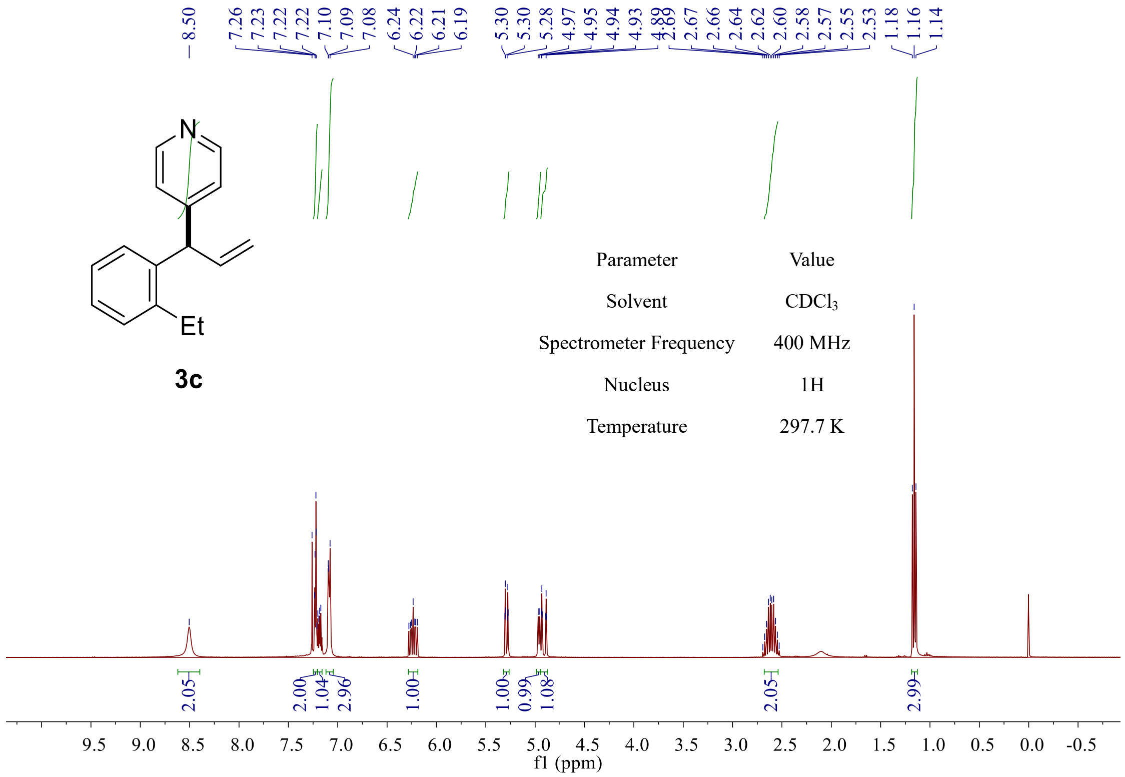

^{1}H NMR Spectrum of Compound **3c**

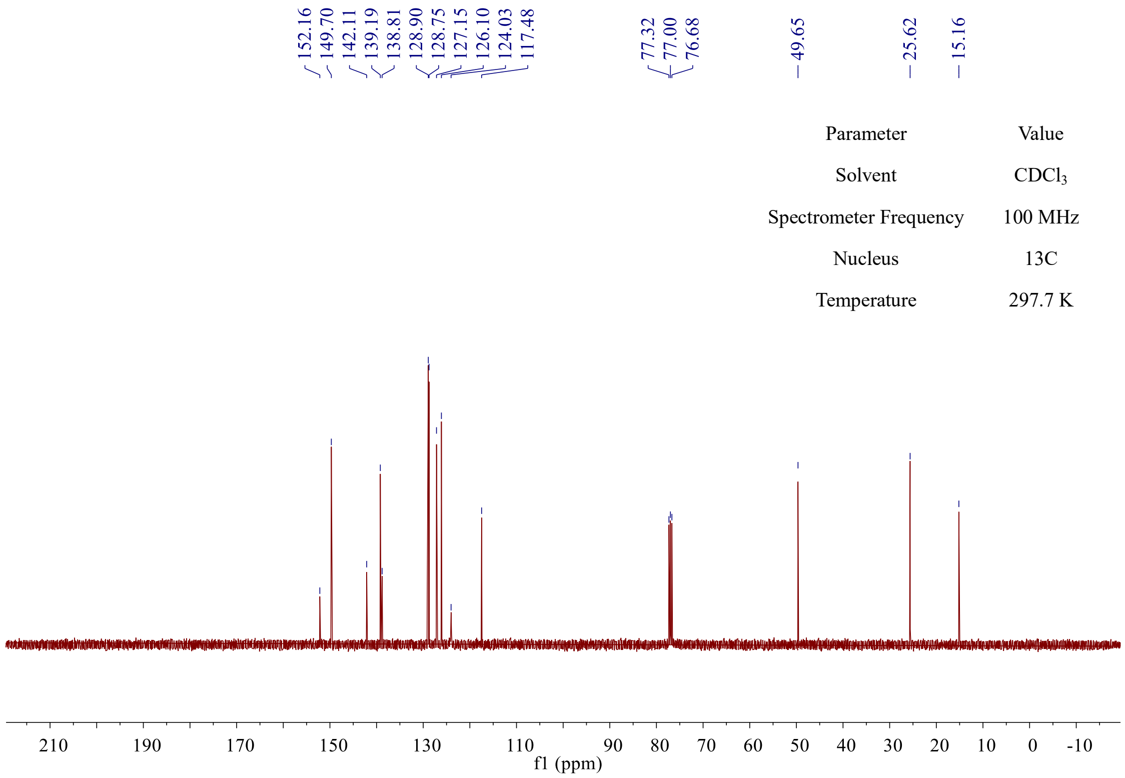

^{13}C NMR Spectrum of Compound **3c**

Supplementary Figure 88**. NMR spectra of 3c**

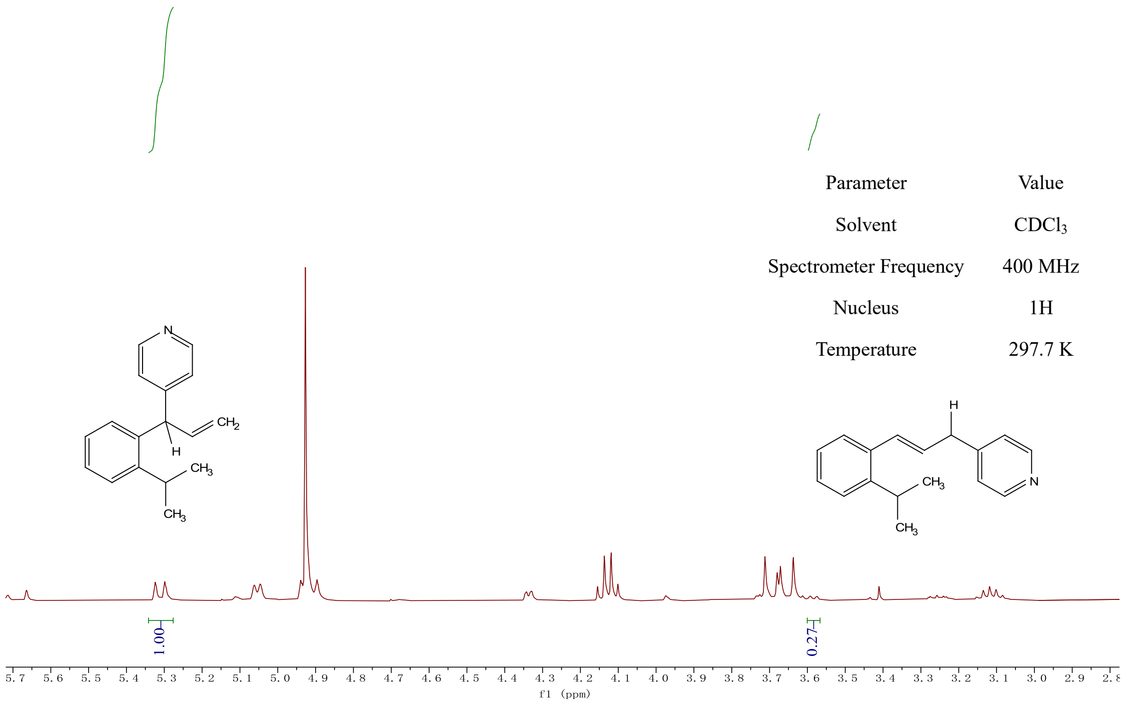

^{1}H NMR Spectrum of Crude Product **3d**

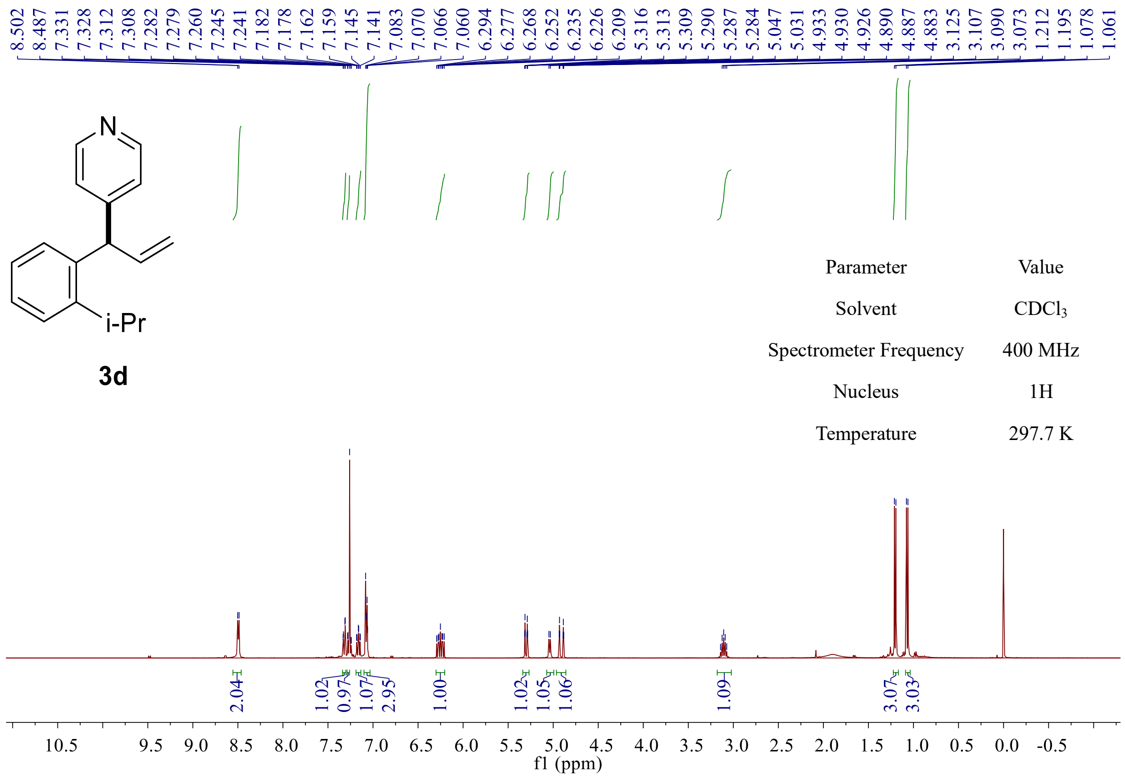

^{1}H NMR Spectrum of Compound **3d**

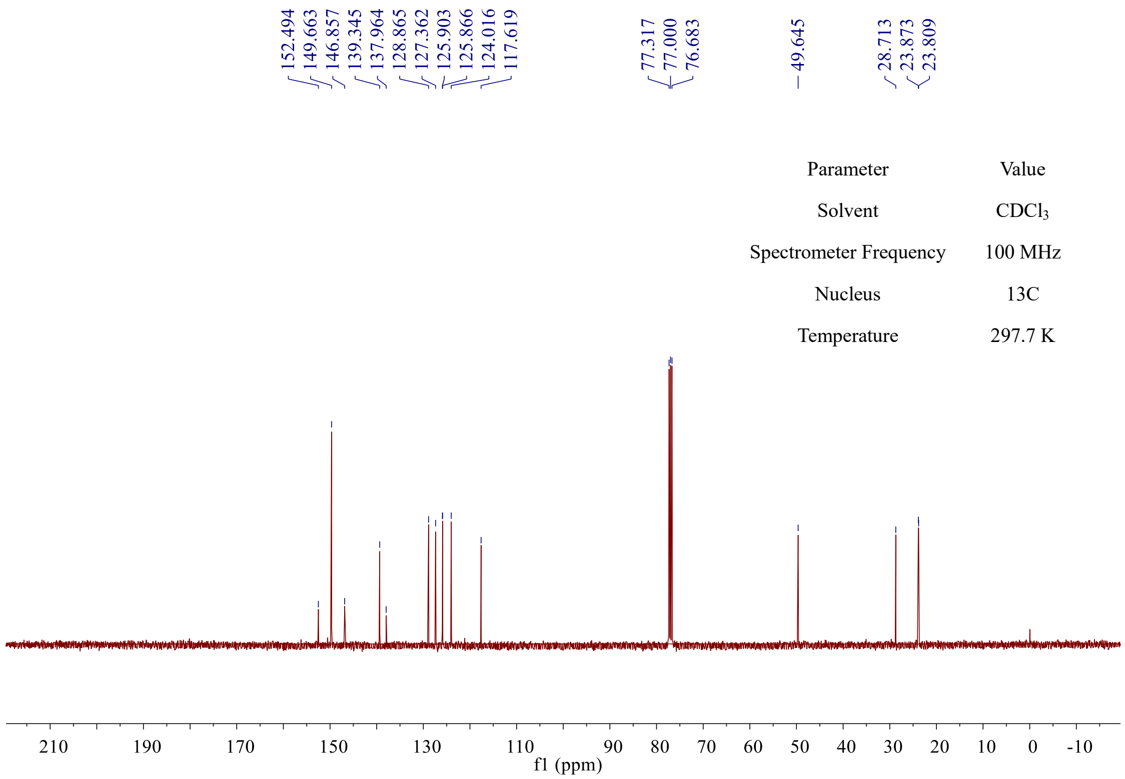

^{13}C NMR Spectrum of Compound **3d**

Supplementary Figure 89. **NMR spectra of 3d**

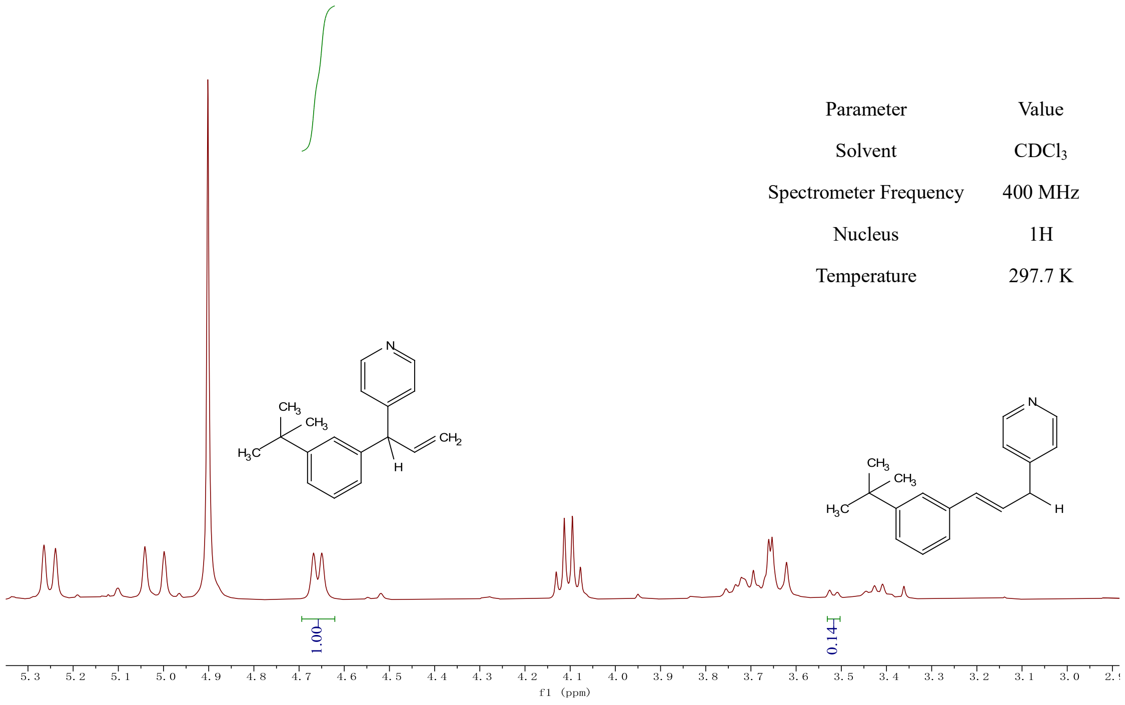

^{1}H NMR Spectrum of Crude Product **3e**

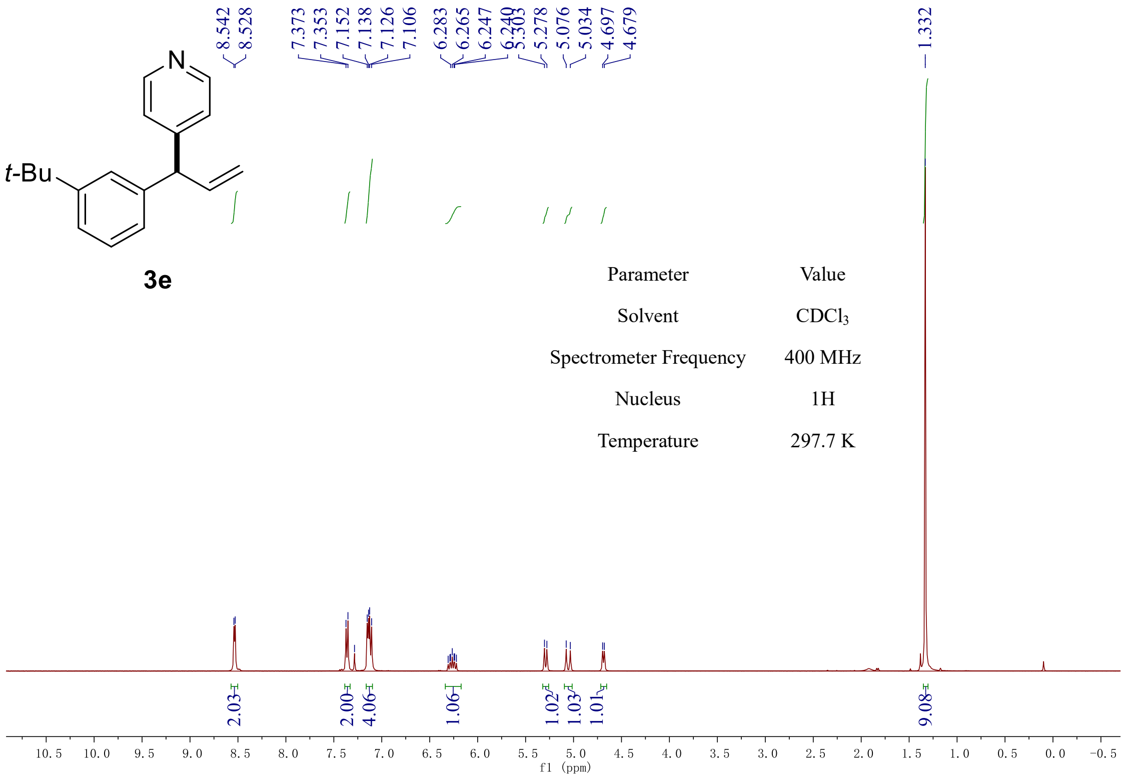

^{1}H NMR Spectrum of Compound **3e**

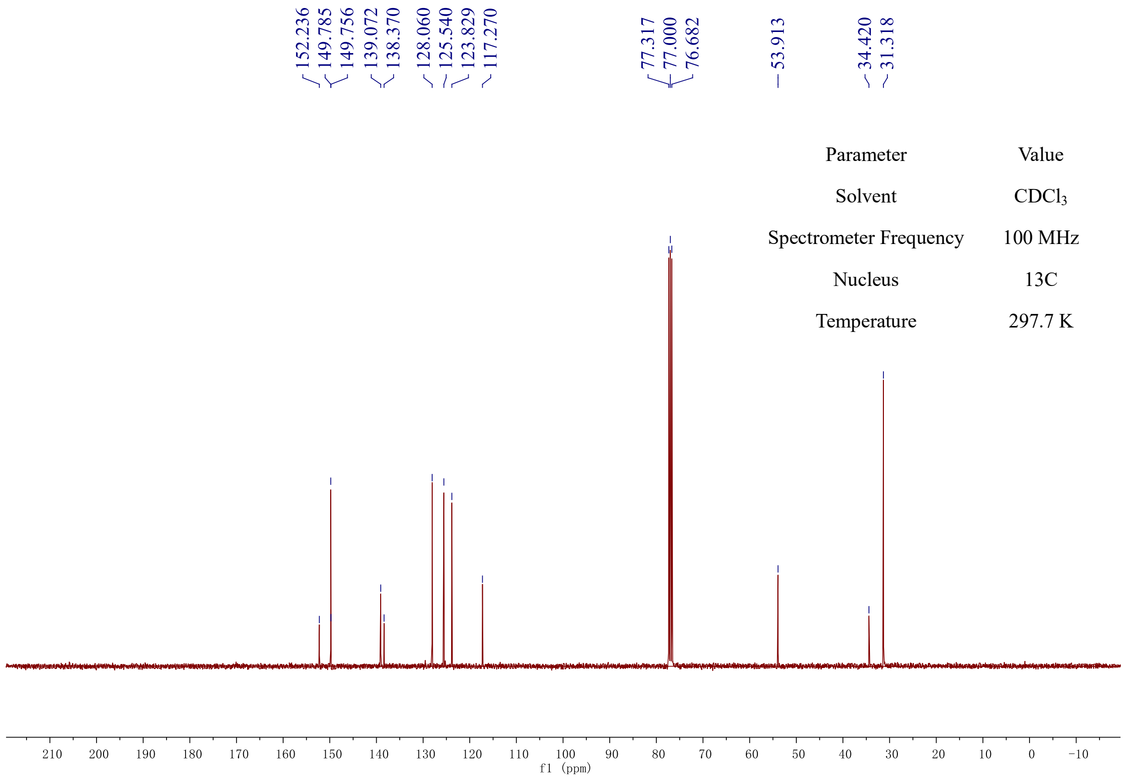

^{13}C NMR Spectrum of Compound **3e**

Supplementary Figure 90**. NMR spectra of 3e**

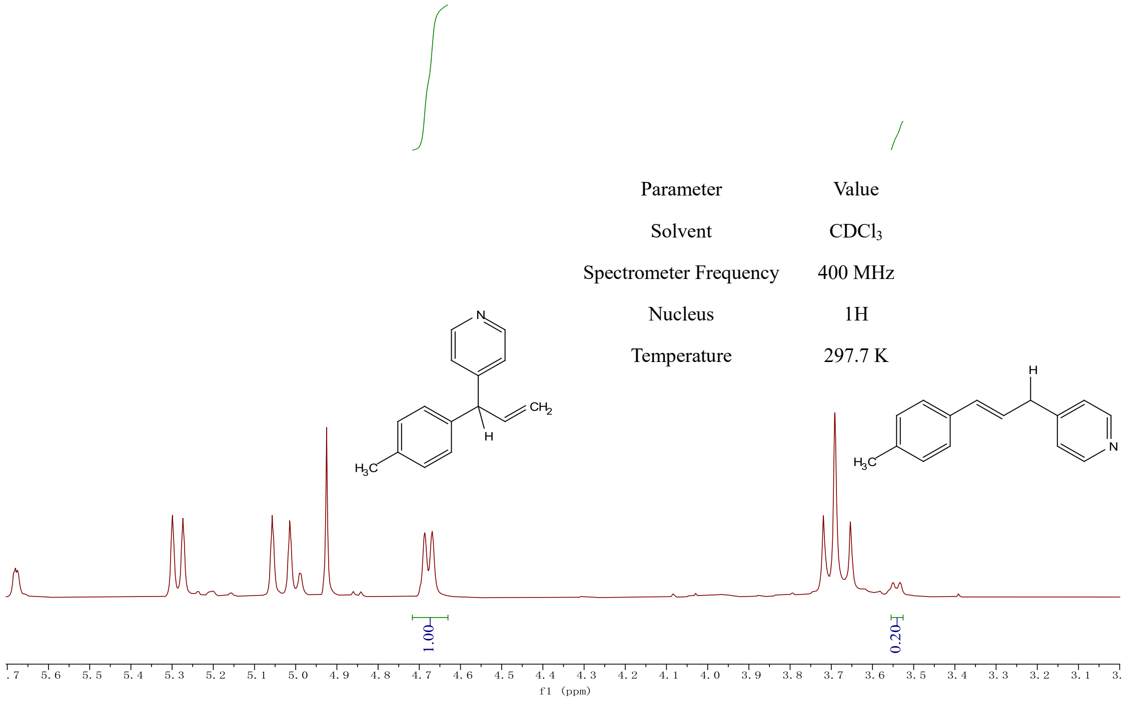

^{1}H NMR Spectrum of Crude Product **3f**

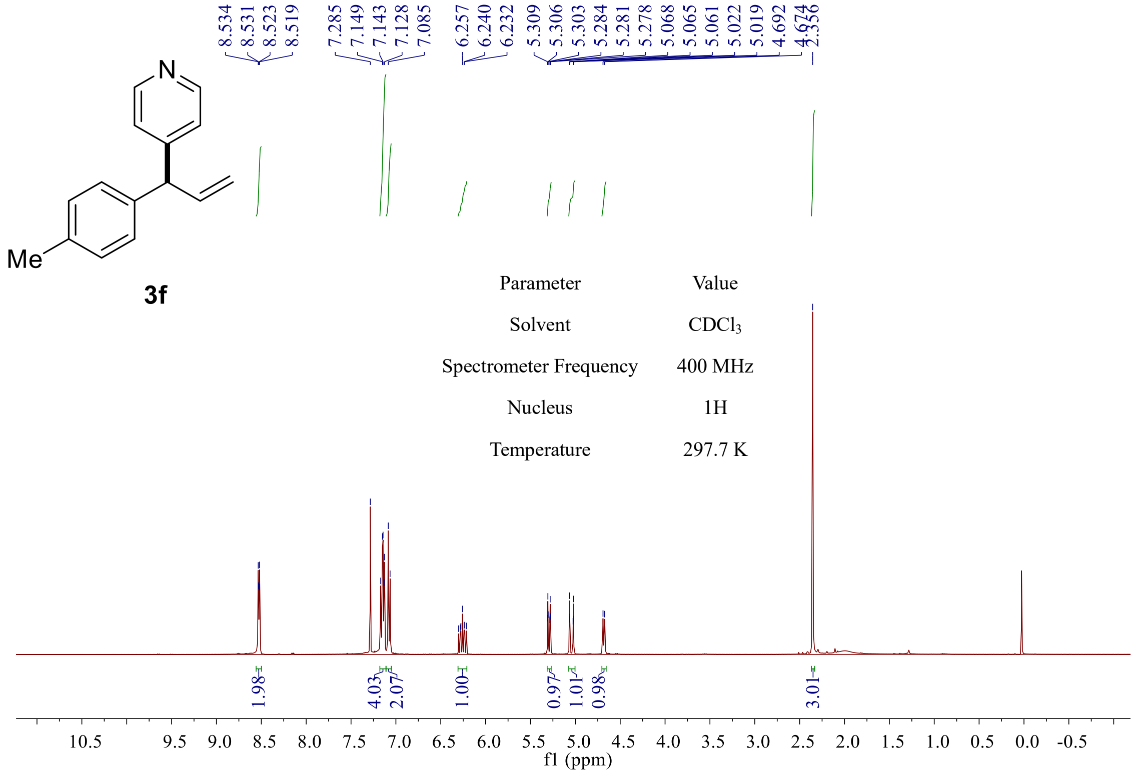

^{1}H NMR Spectrum of Compound **3f**

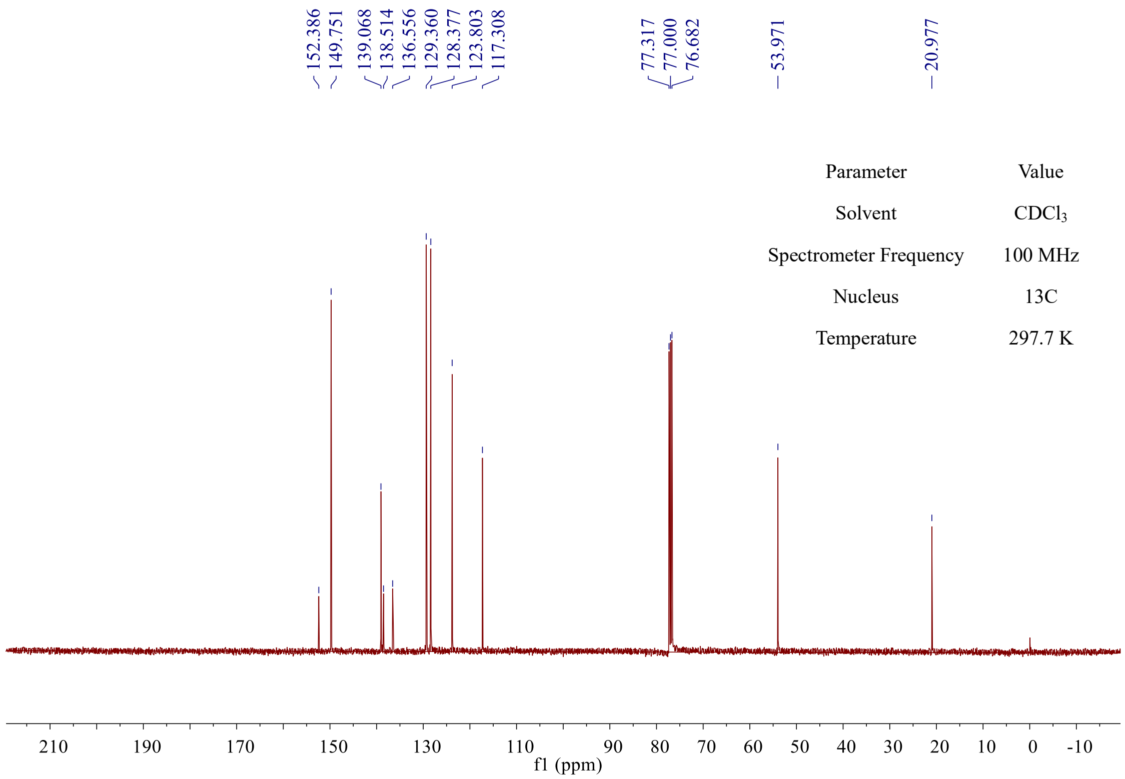

^{13}C NMR Spectrum of Compound **3f**

Supplementary Figure 91. **NMR spectra of 3f**

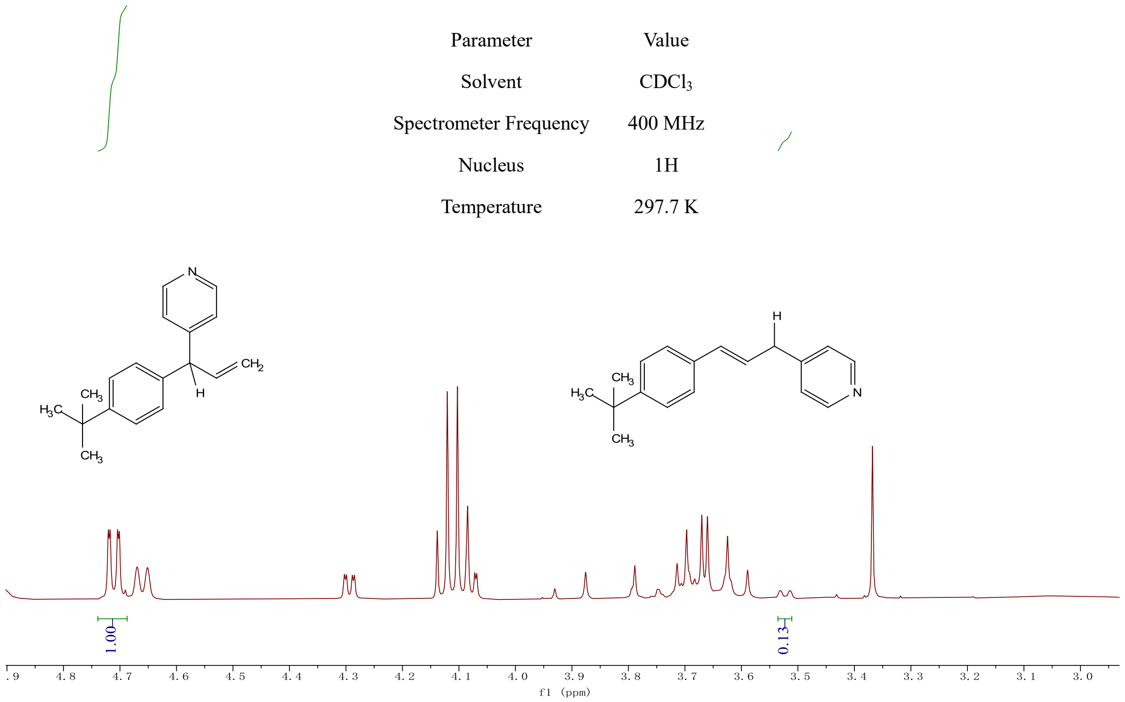

^{1}H NMR Spectrum of Crude Product **3g**

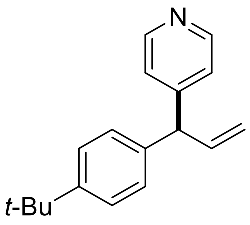

3g

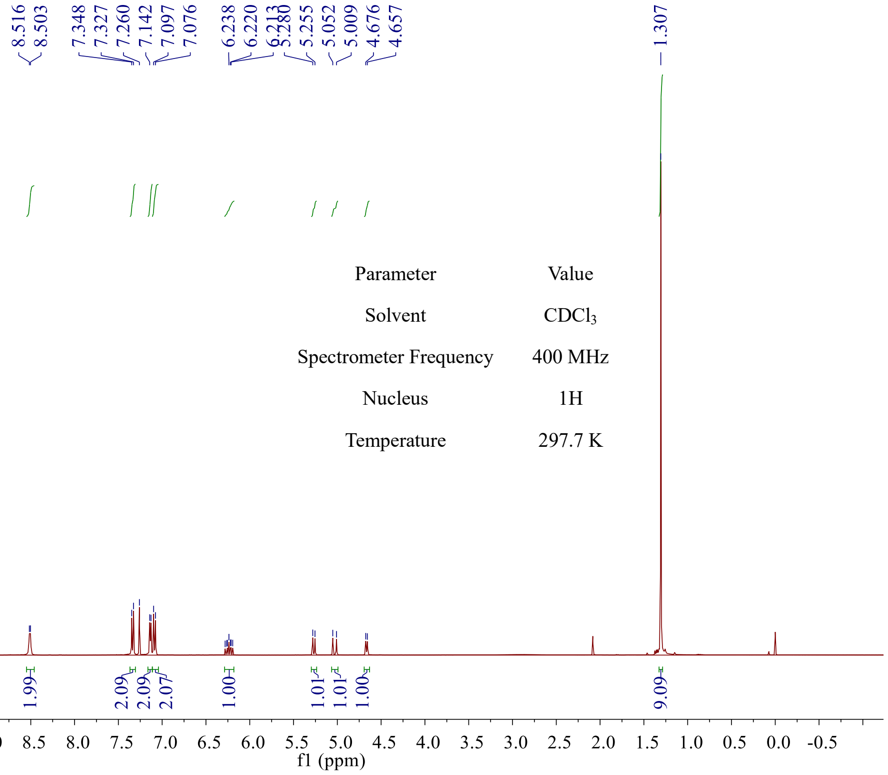

^{1}H NMR Spectrum of Compound **3g**

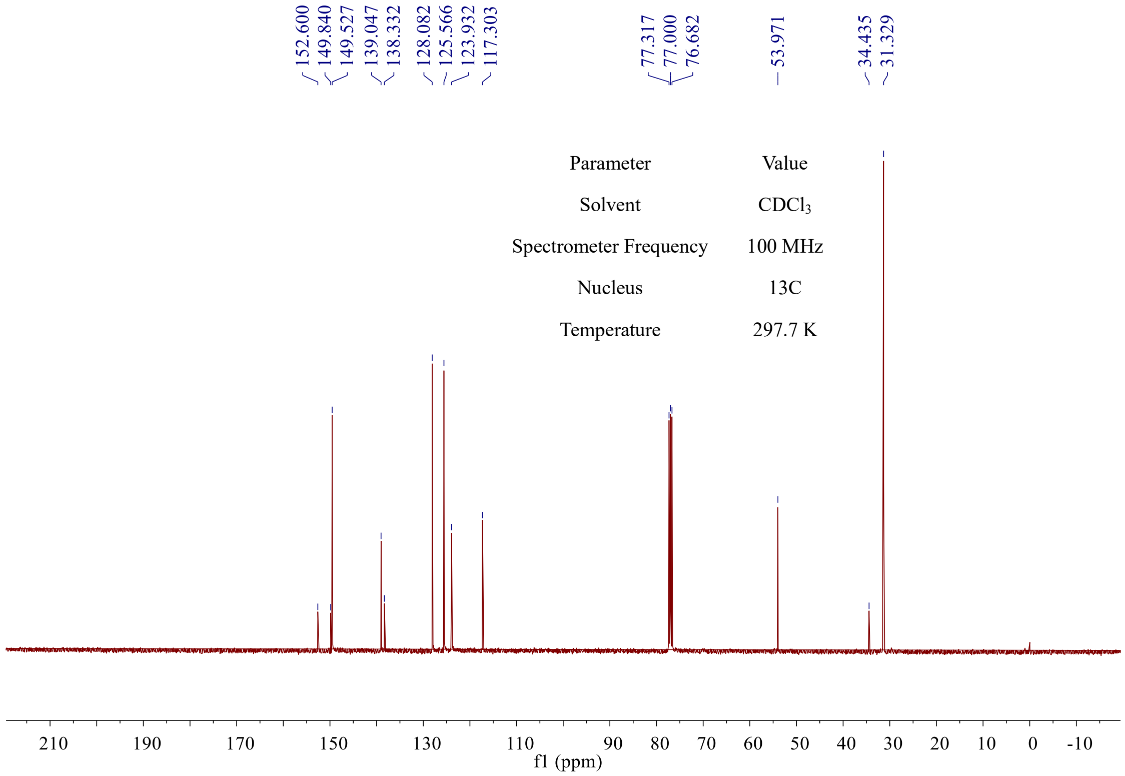

^{13}C NMR Spectrum of Compound **3g**

Supplementary Figure 92. **NMR spectra of 3g**

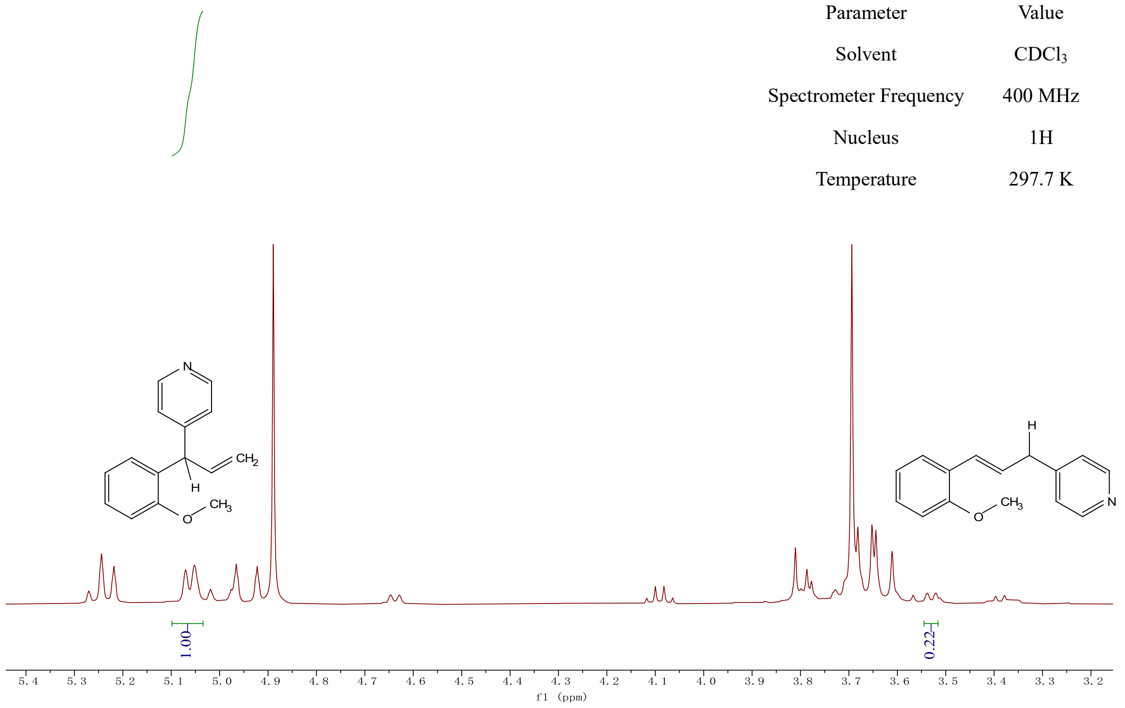

Parameter	Value
Solvent	$CDCl_3$
Spectrometer Frequency	400 MHz
Nucleus	1H
Temperature	297.7 K

^{1}H NMR Spectrum of Crude Product **3h**

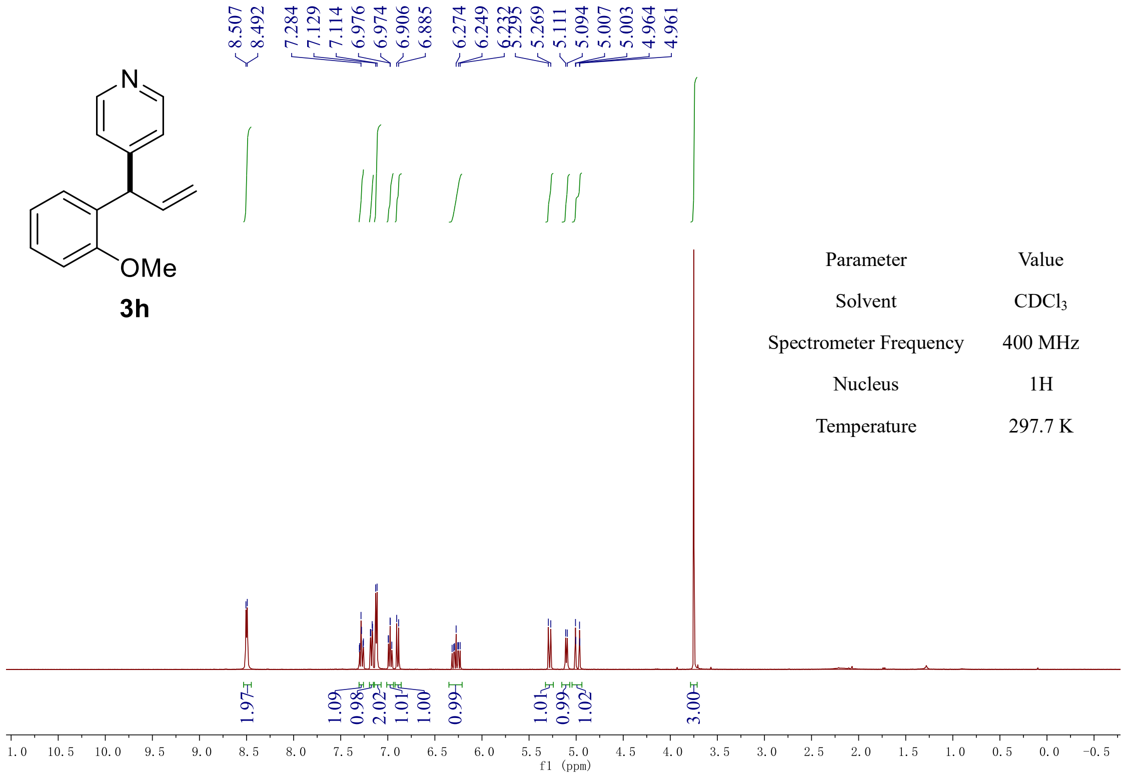

Parameter	Value
Solvent	$CDCl_3$
Spectrometer Frequency	400 MHz
Nucleus	1H
Temperature	297.7 K

^{1}H NMR Spectrum of Compound **3h**

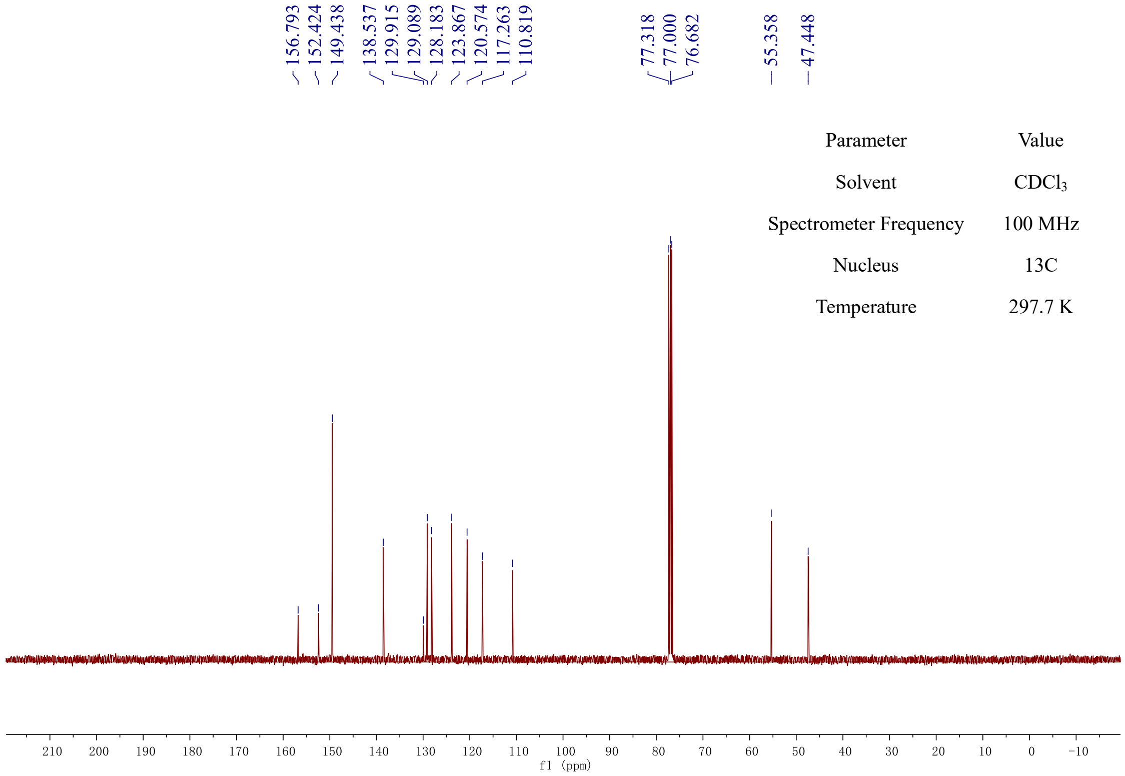

^{13}C NMR Spectrum of Compound **3h**

Supplementary Figure 93. **NMR spectra of 3h**

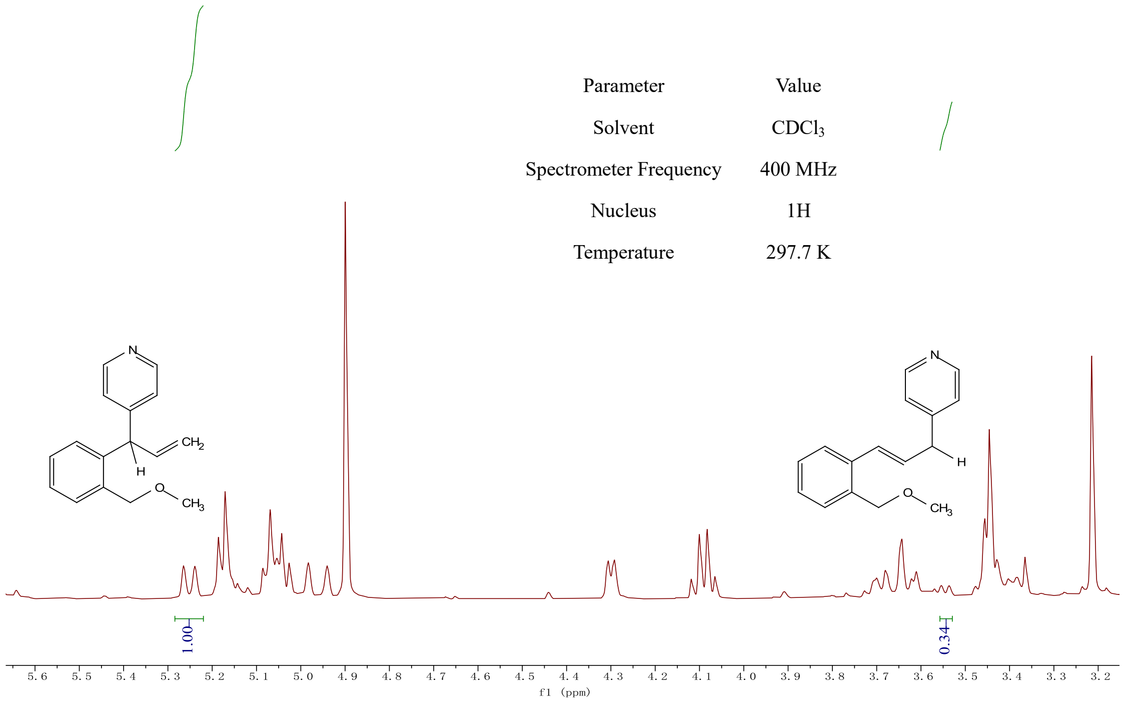

^{1}H NMR Spectrum of Crude **3i**

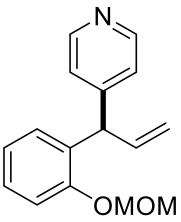

3i

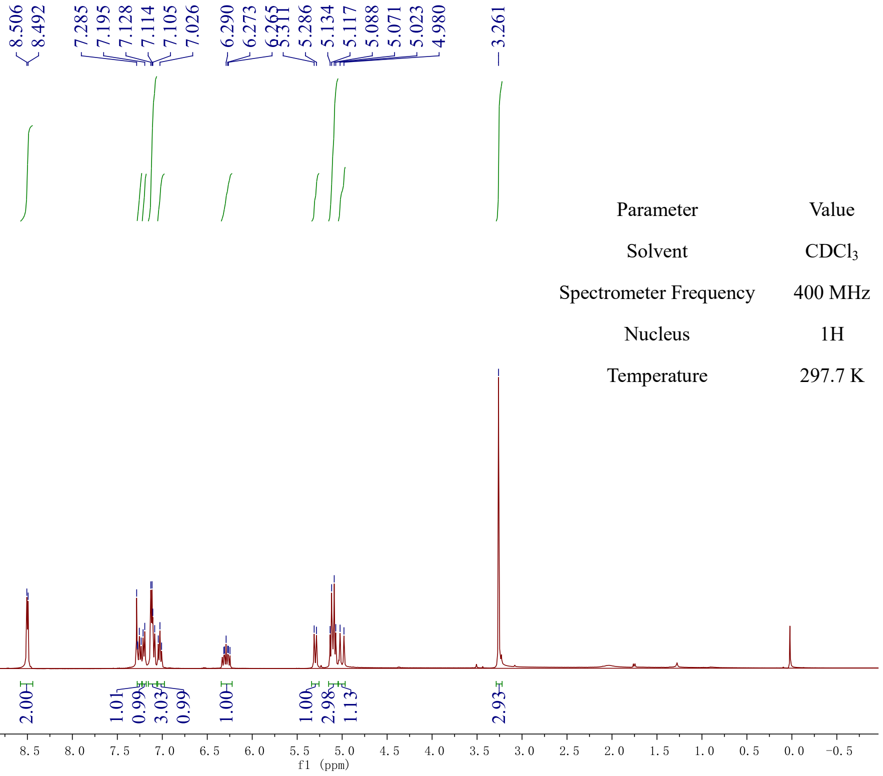

^{1}H NMR Spectrum of Compound **3i**

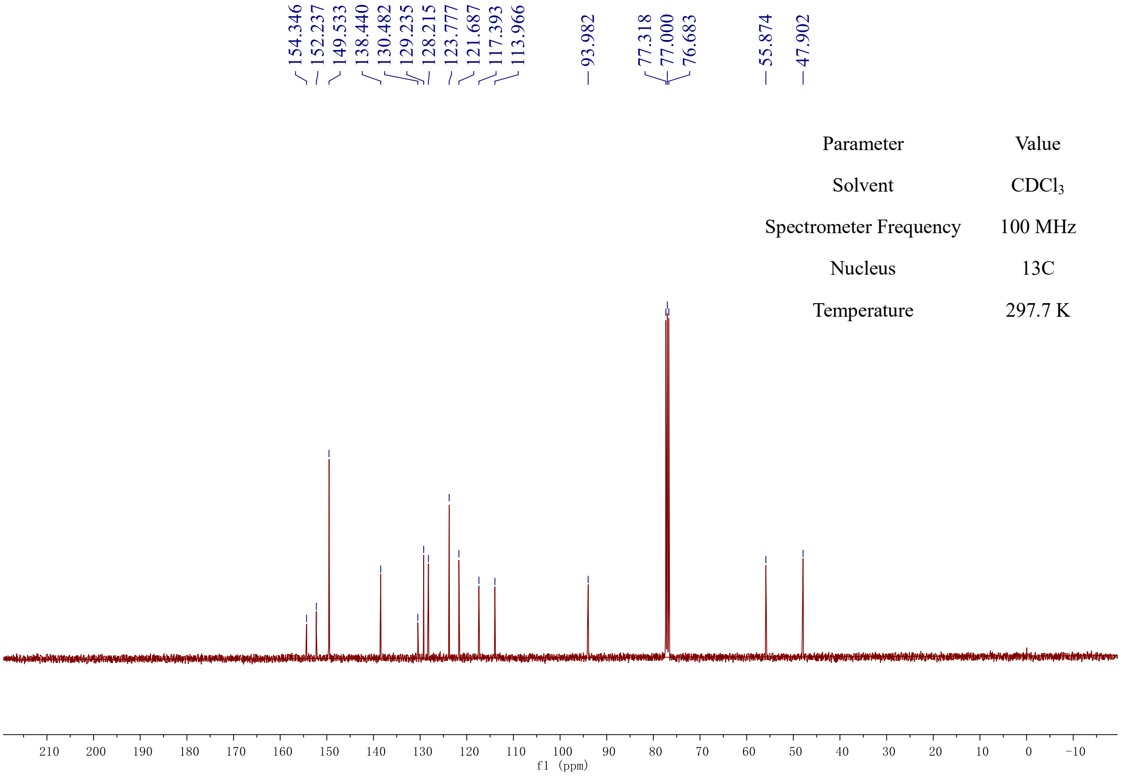

^{13}C NMR Spectrum of Compound **3i**

Supplementary Figure 94**. NMR spectra of 3i**

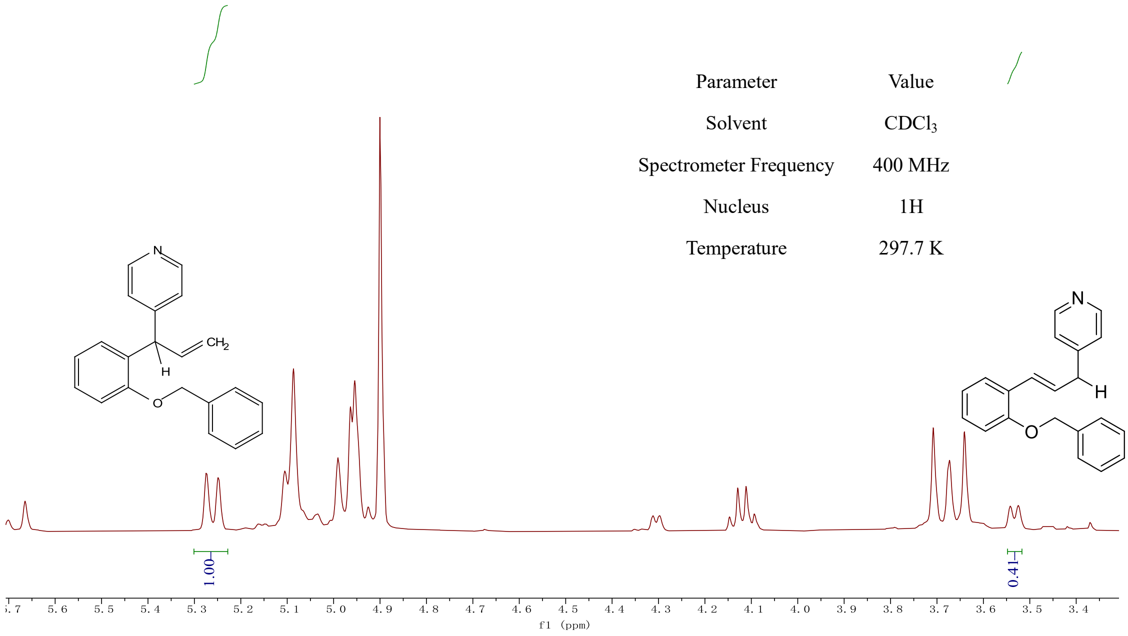

^{1}H NMR Spectrum of Crude Product **3j**

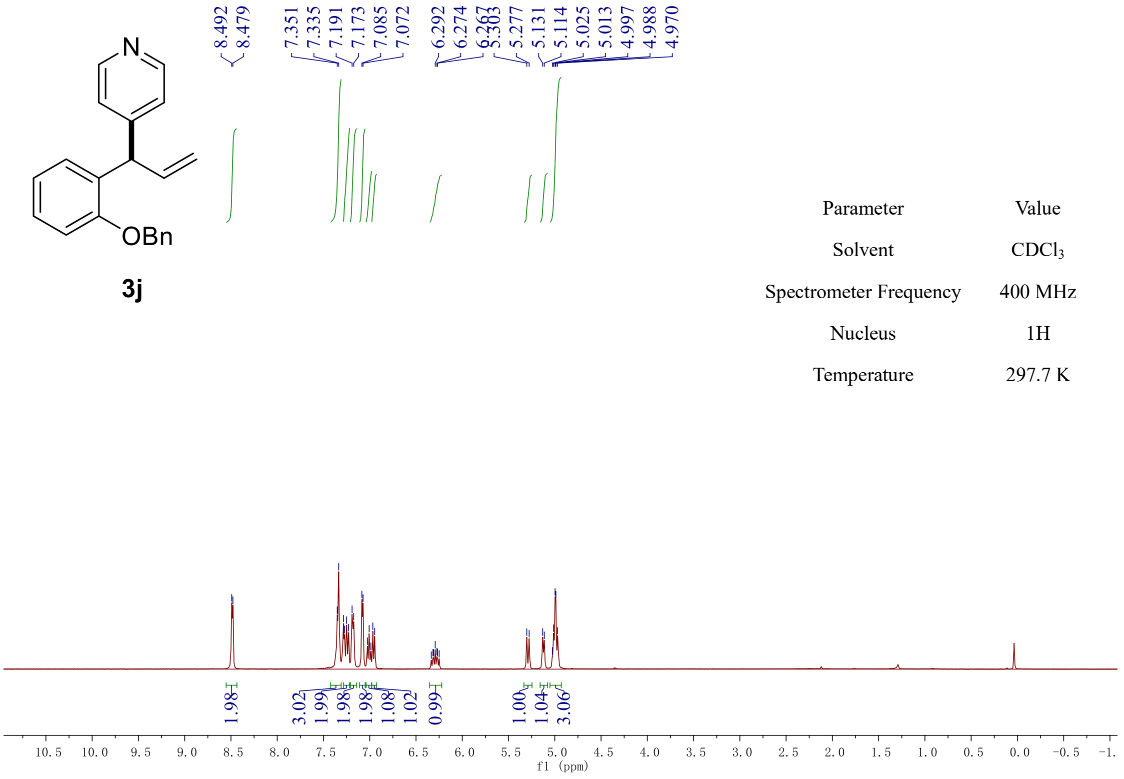

^{1}H NMR Spectrum of Compound **3j**

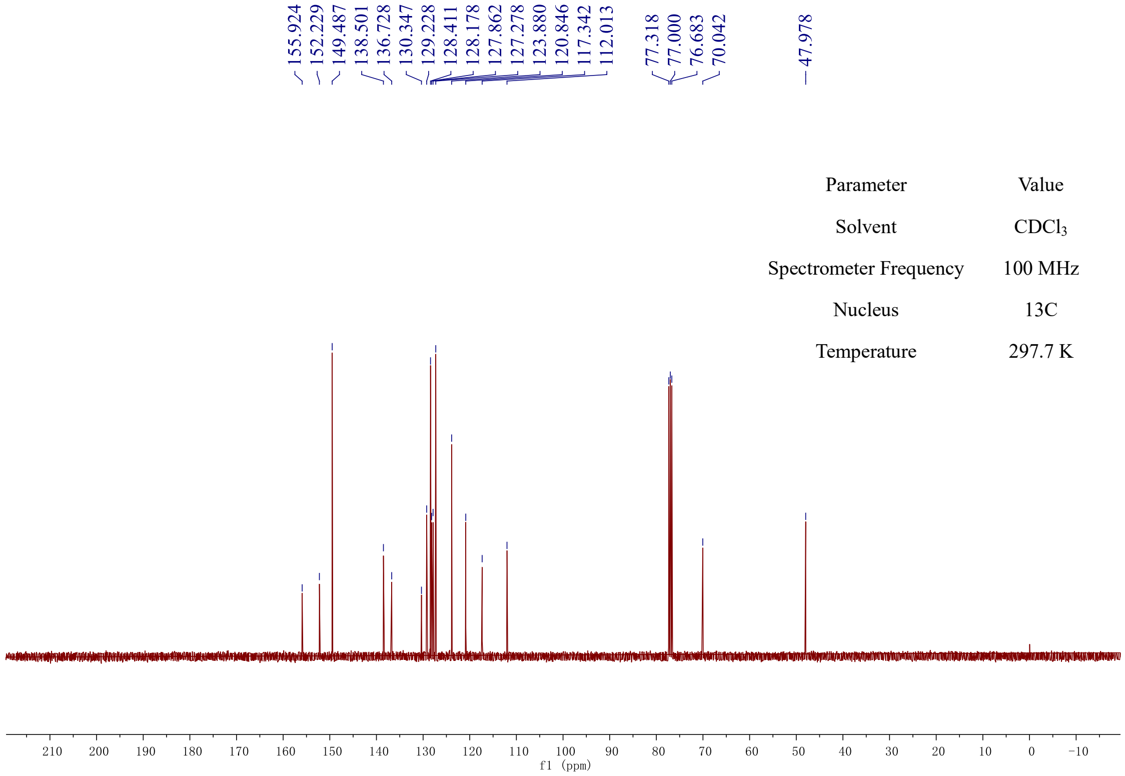

^{13}C NMR Spectrum of Compound **3j**

Supplementary Figure 95. NMR spectra of **3j**

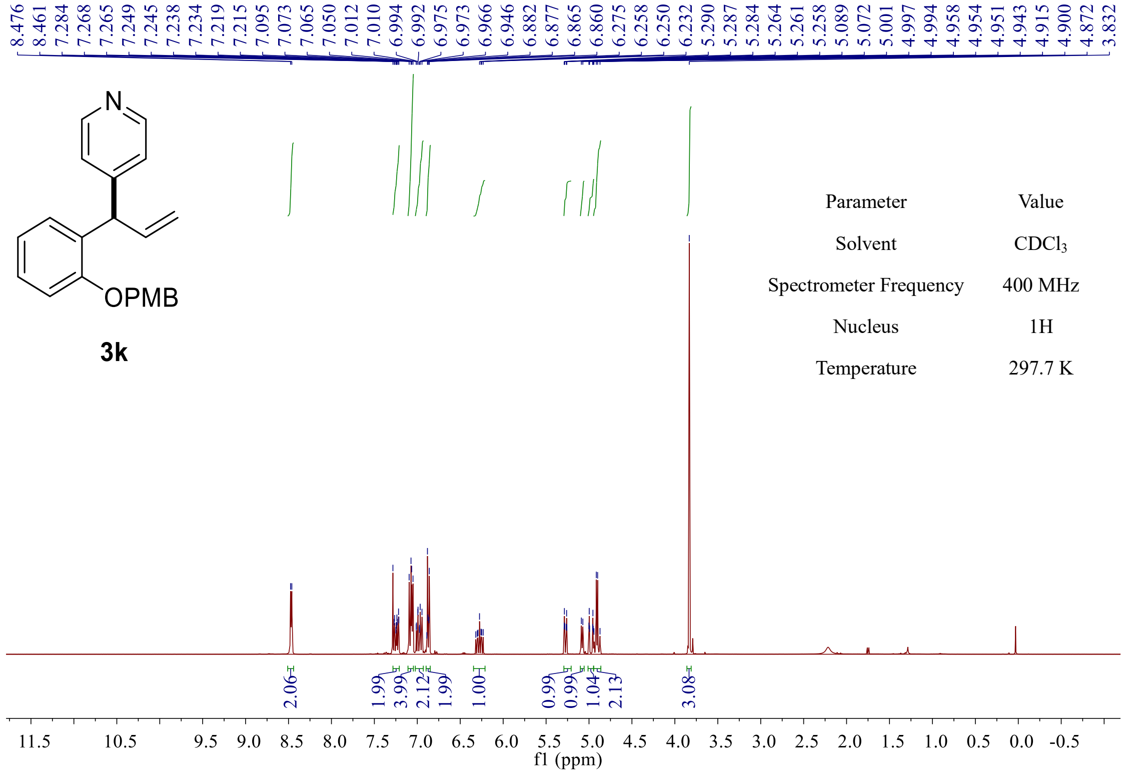

^{1}H NMR Spectrum of Compound **3k**

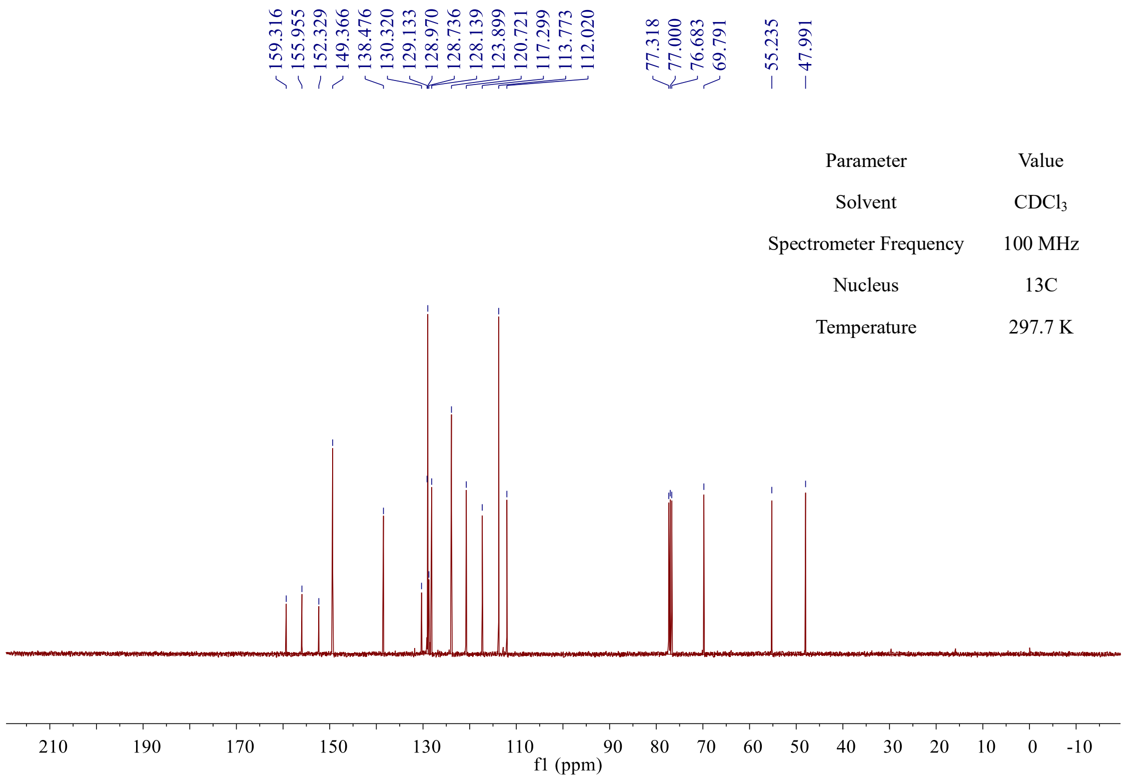

Parameter	Value
Solvent	$CDCl_3$
Spectrometer Frequency	100 MHz
Nucleus	13C
Temperature	297.7 K

^{13}C NMR Spectrum of Compound **3k**

Supplementary Figure 96. **NMR spectra of 3k**

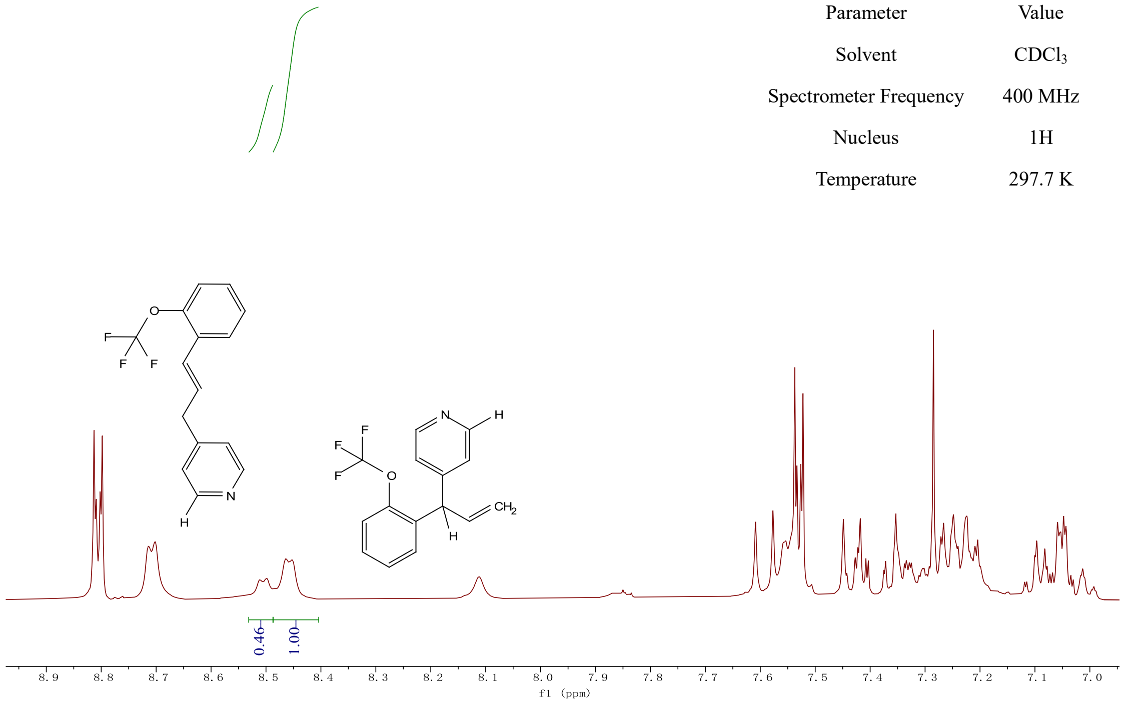

Parameter	Value
Solvent	$CDCl_3$
Spectrometer Frequency	400 MHz
Nucleus	1H
Temperature	297.7 K

^{1}H NMR Spectrum of Crude Product **3l**

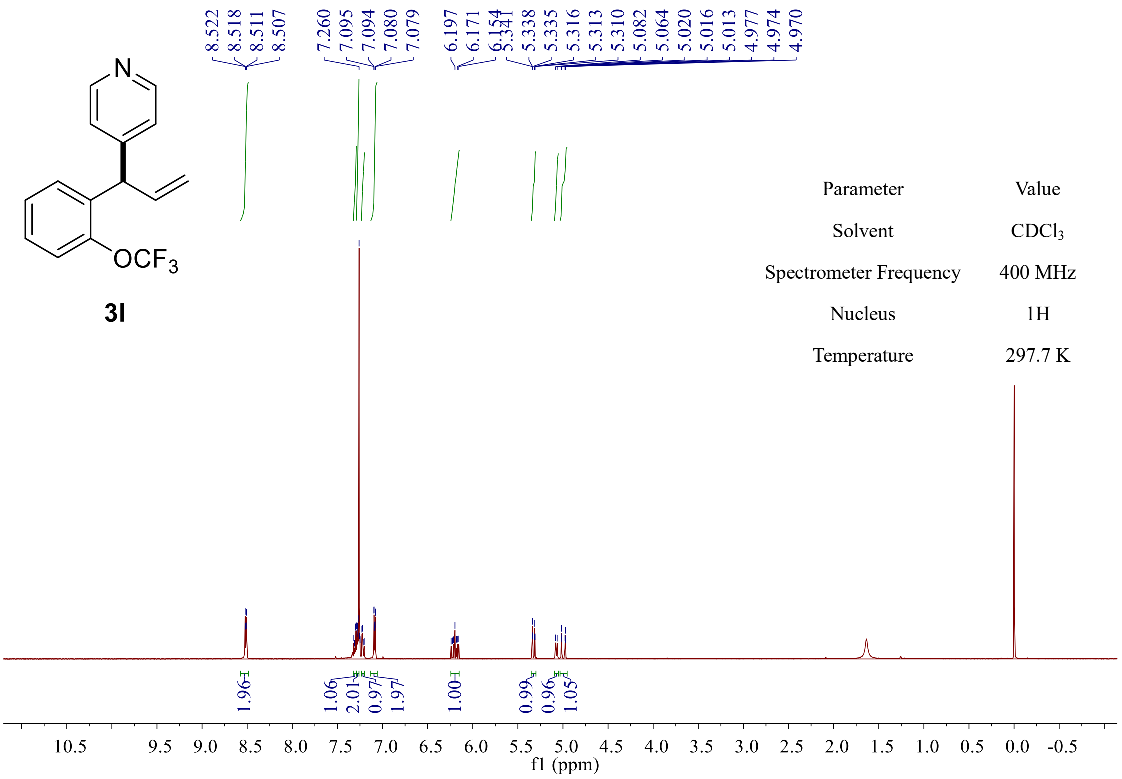

^{1}H NMR Spectrum of Compound **3l**

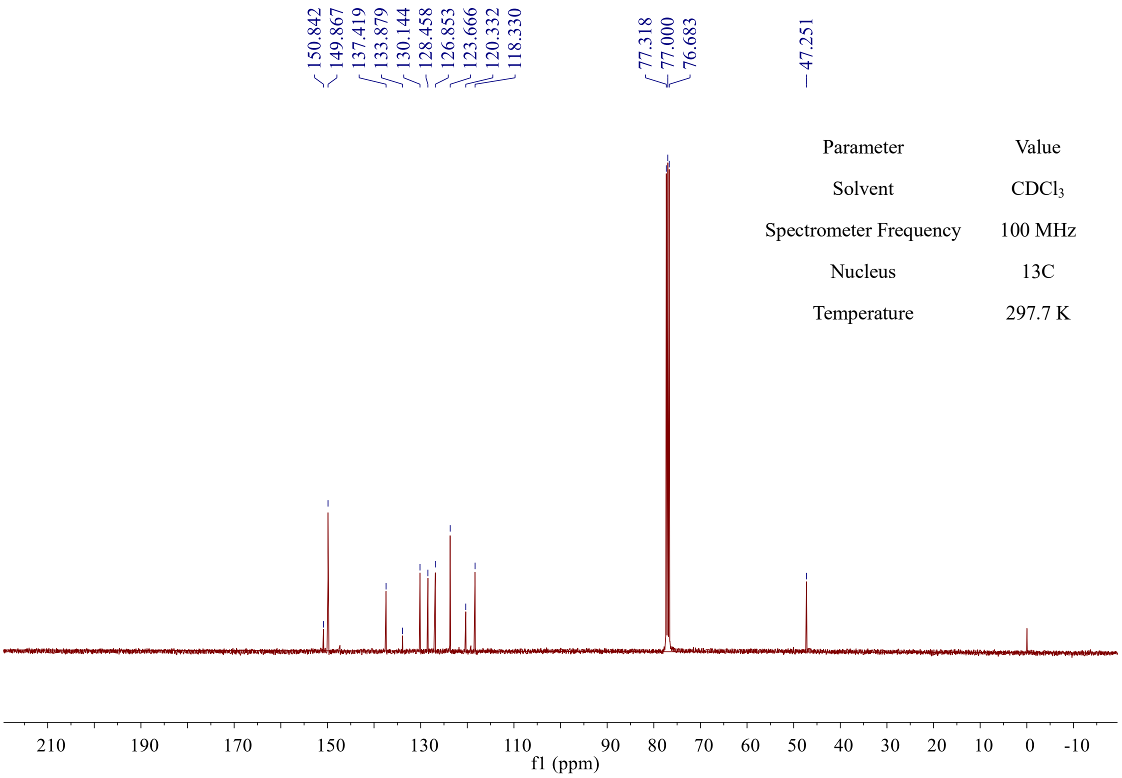

^{13}C NMR Spectrum of Compound **3l**

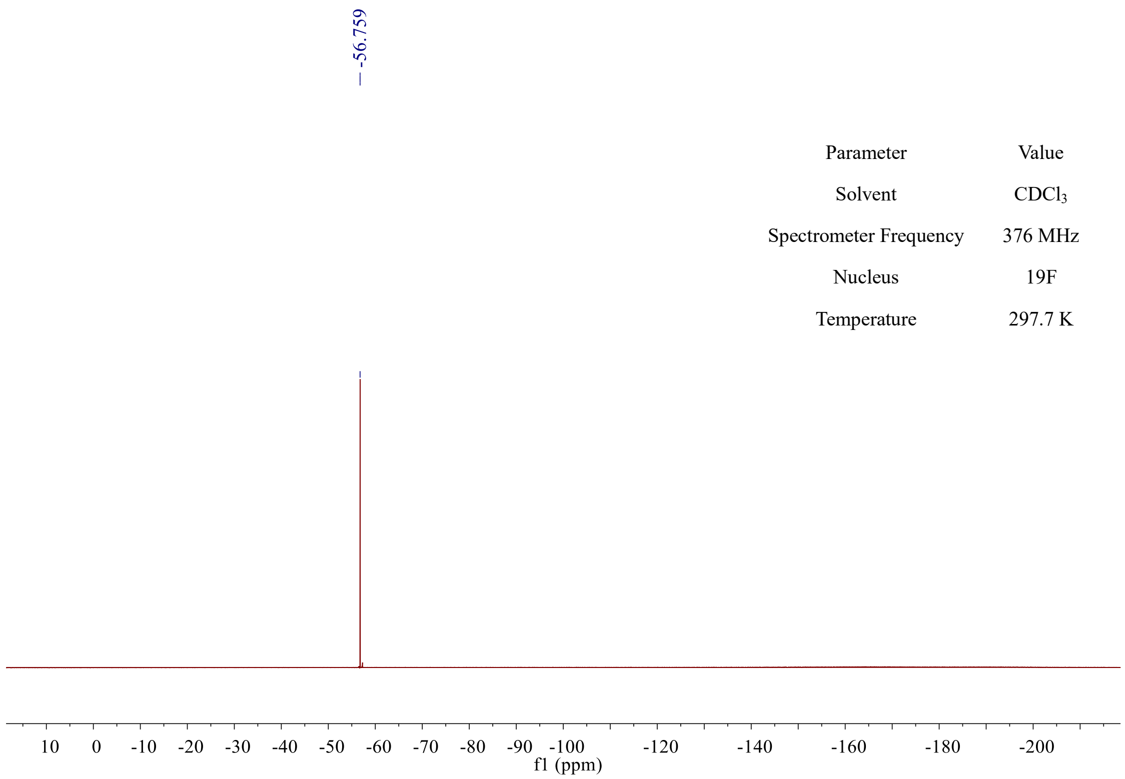

^{19}F NMR Spectrum of Compound **3l**

Supplementary Figure 97. **NMR spectra of 3l**

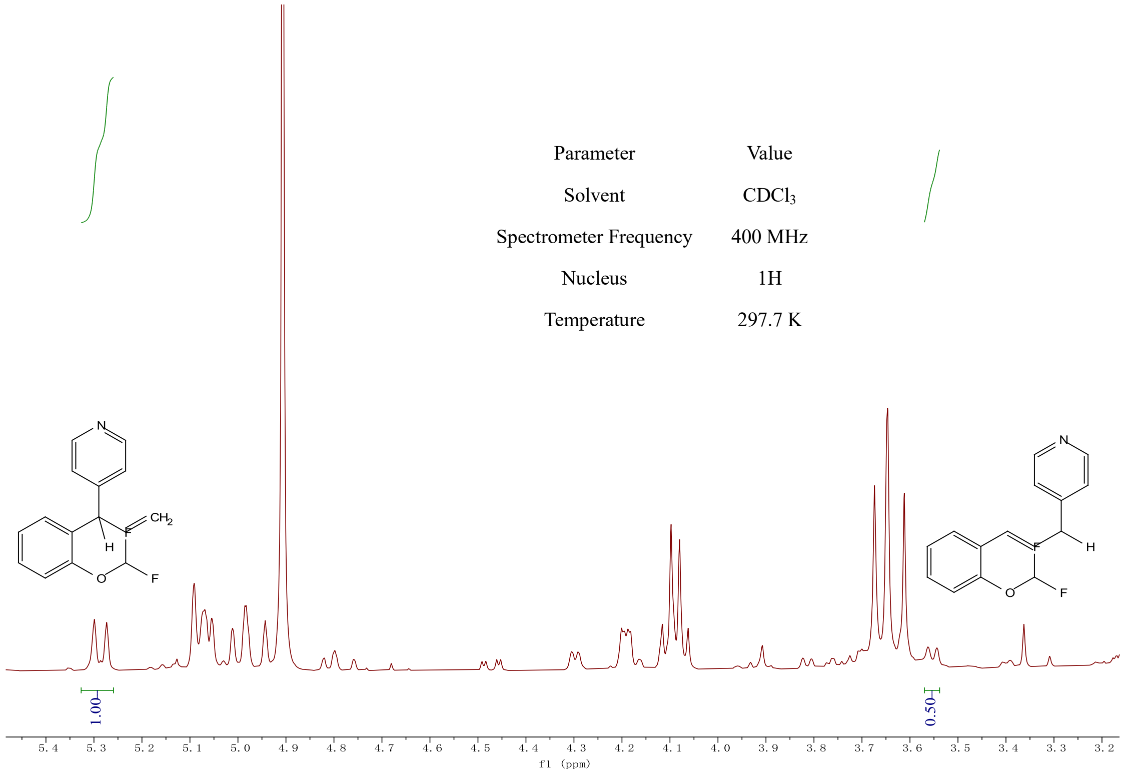

^{1}H NMR Spectrum of Crude Product **3m**

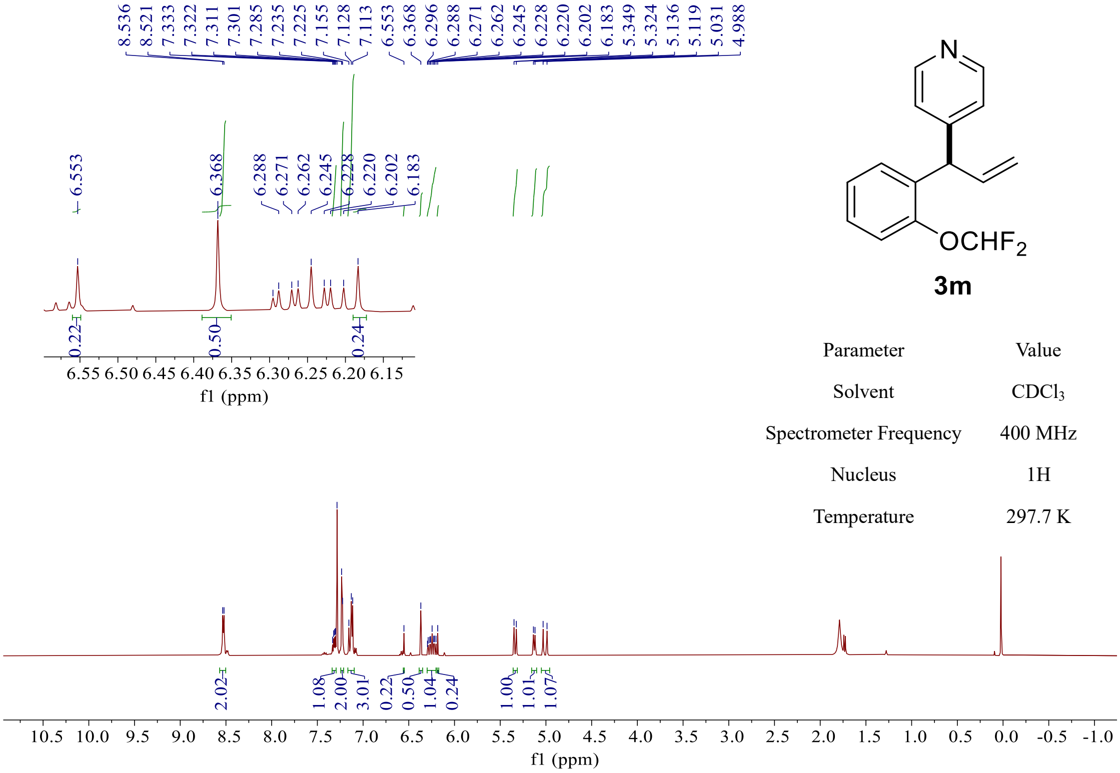

3m

Parameter	Value
Solvent	$CDCl_3$
Spectrometer Frequency	400 MHz
Nucleus	1H
Temperature	297.7 K

^{1}H NMR Spectrum of Compound **3m**

Parameter	Value
Solvent	$CDCl_3$
Spectrometer Frequency	100 MHz
Nucleus	13C
Temperature	297.7 K

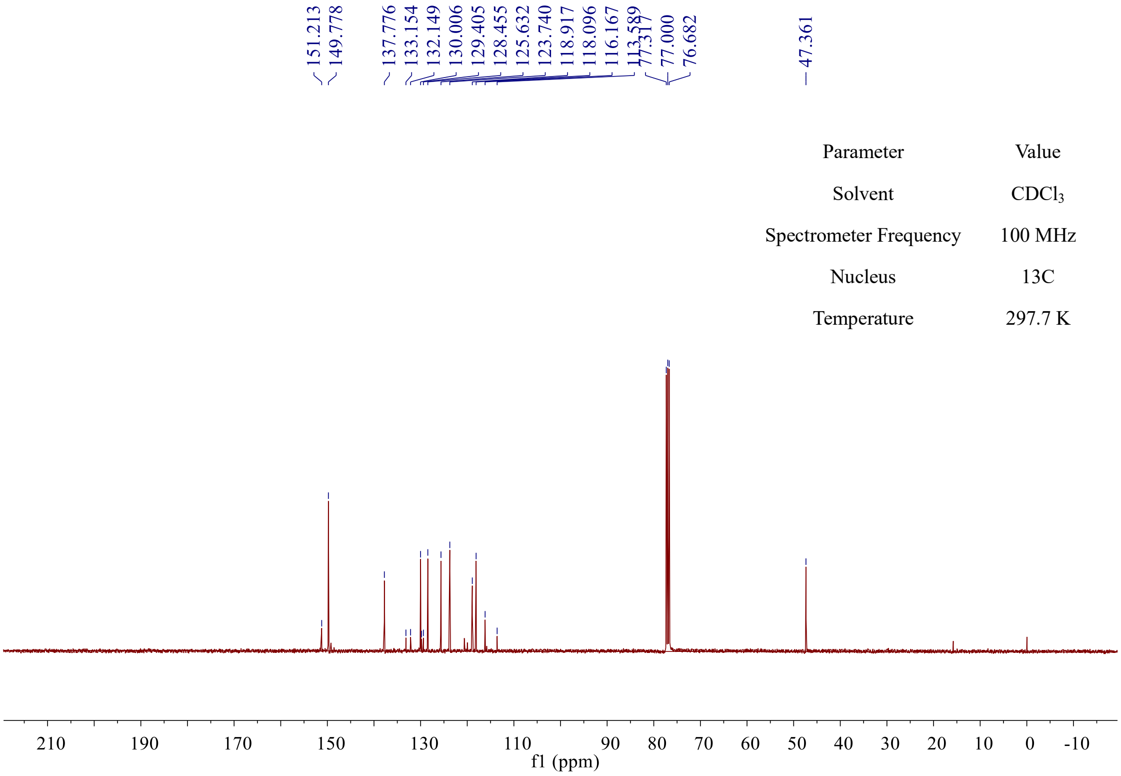

^{13}C NMR Spectrum of Compound **3m**

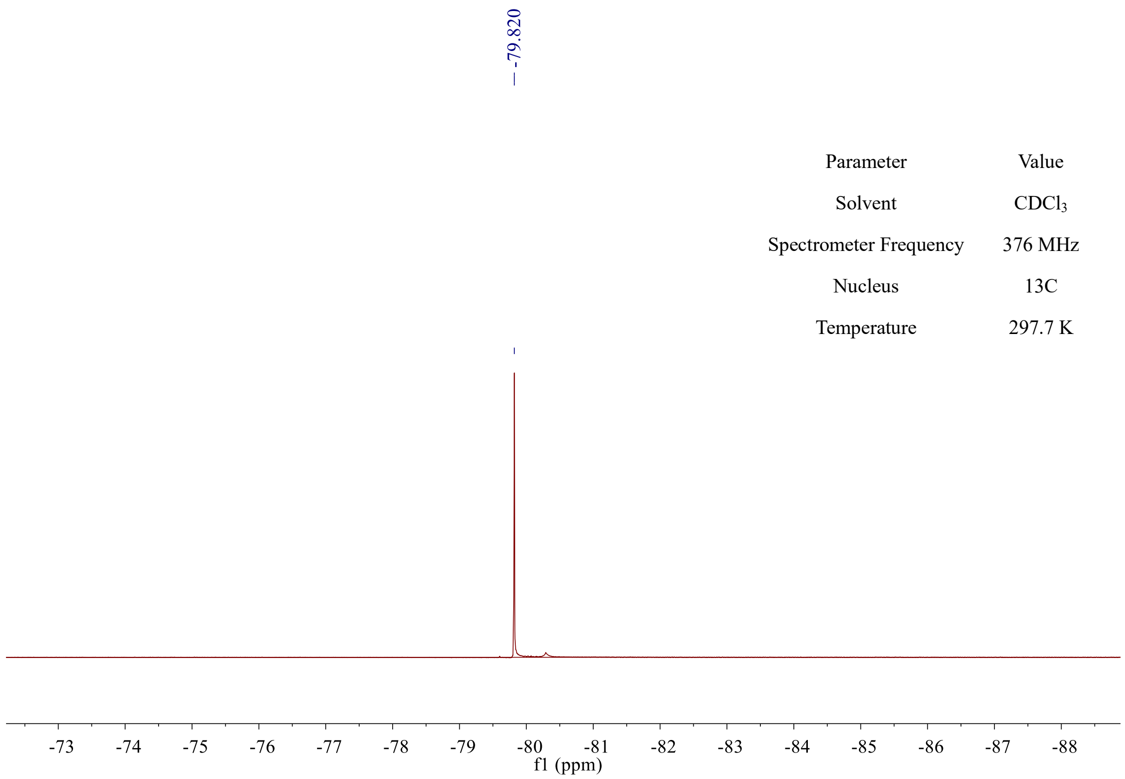

^{19}F NMR Spectrum of Compound **3m**

Supplementary Figure 98**. NMR spectra of 3m**

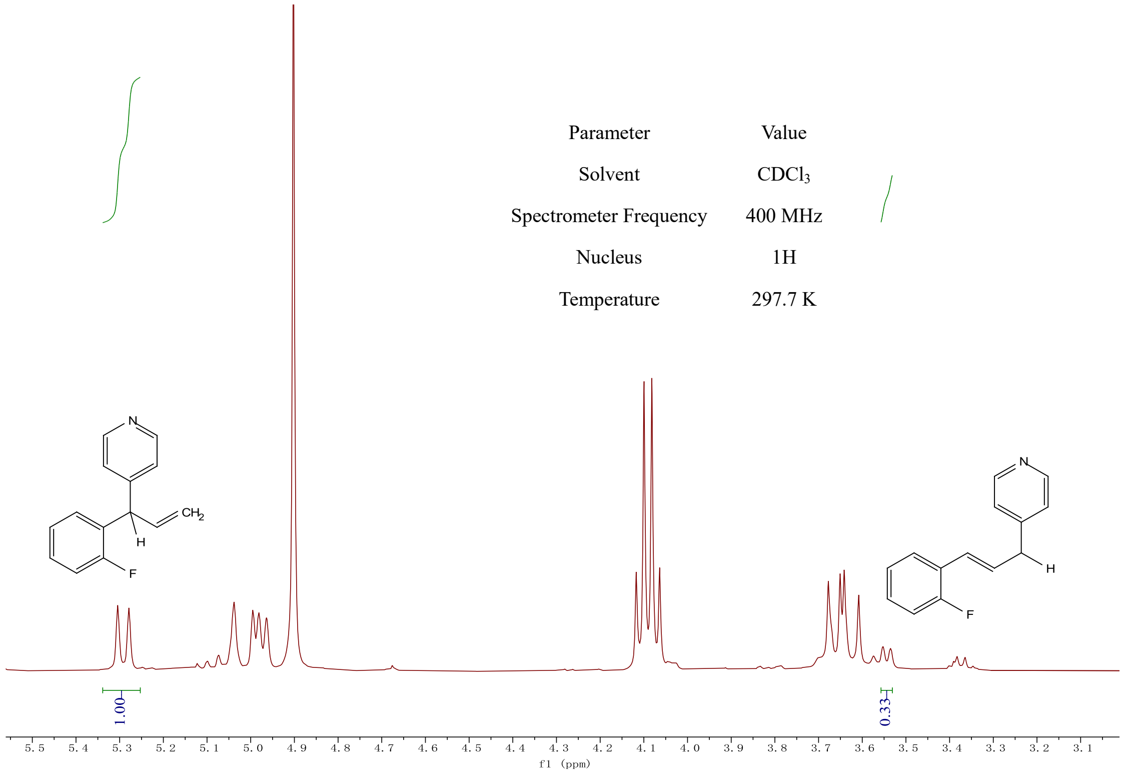

^{1}H NMR Spectrum of Crude Product **3n**

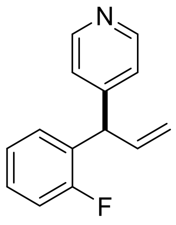

3n

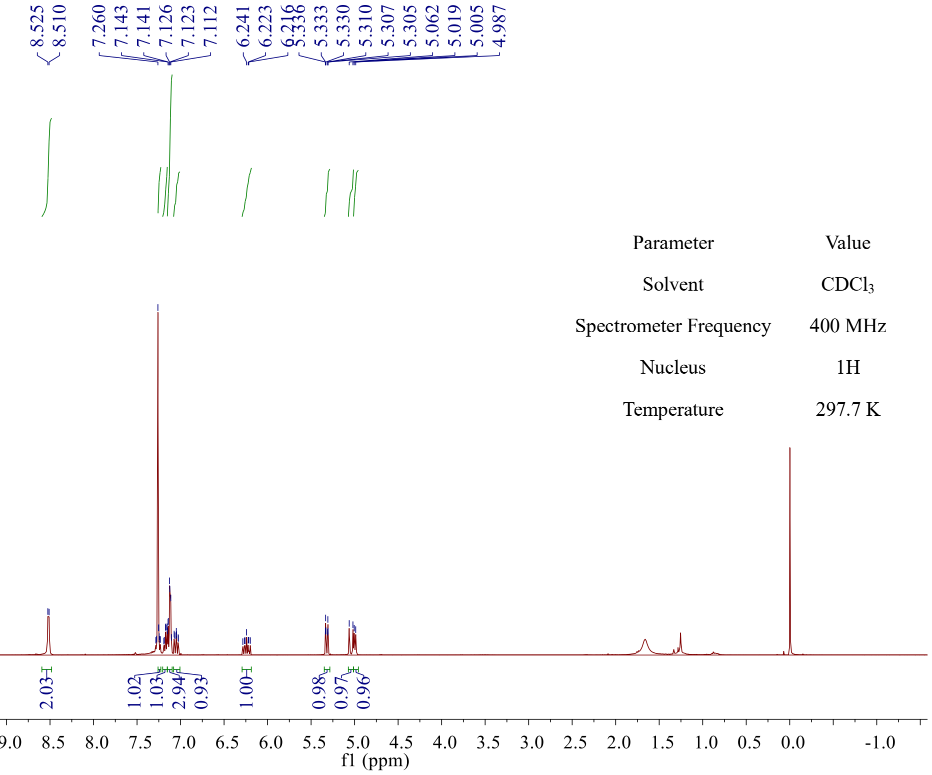

Parameter	Value
Solvent	$CDCl_3$
Spectrometer Frequency	400 MHz
Nucleus	1H
Temperature	297.7 K

^{1}H NMR Spectrum of Compound **3n**

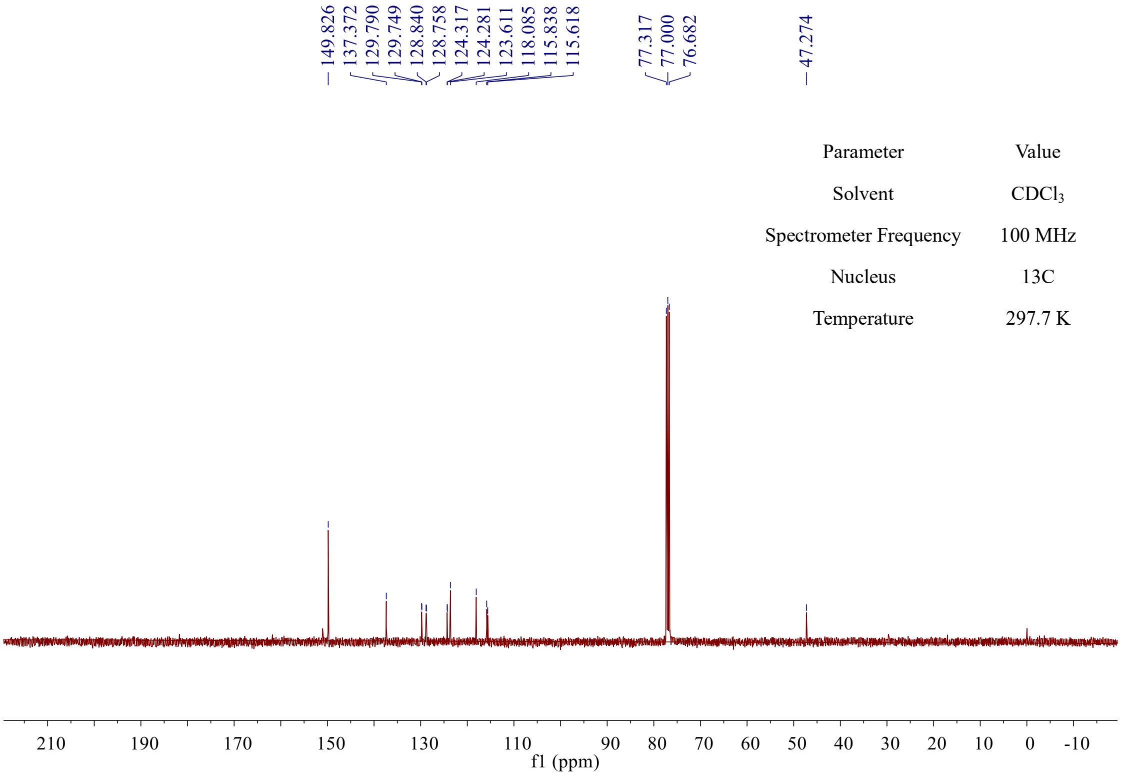

Parameter	Value
Solvent	$CDCl_3$
Spectrometer Frequency	100 MHz
Nucleus	13C
Temperature	297.7 K

^{13}C NMR Spectrum of Compound **3n**

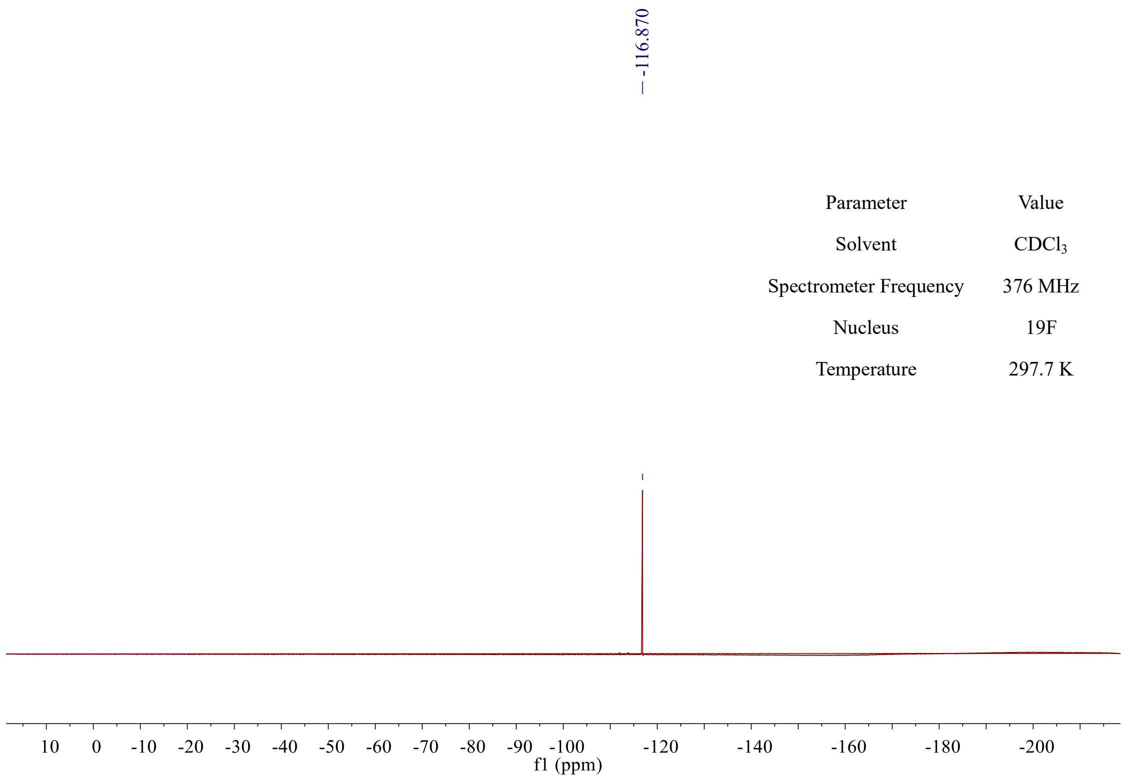

^{19}F NMR Spectrum of Compound **3n**

Supplementary Figure 99**. NMR spectra of 3n**

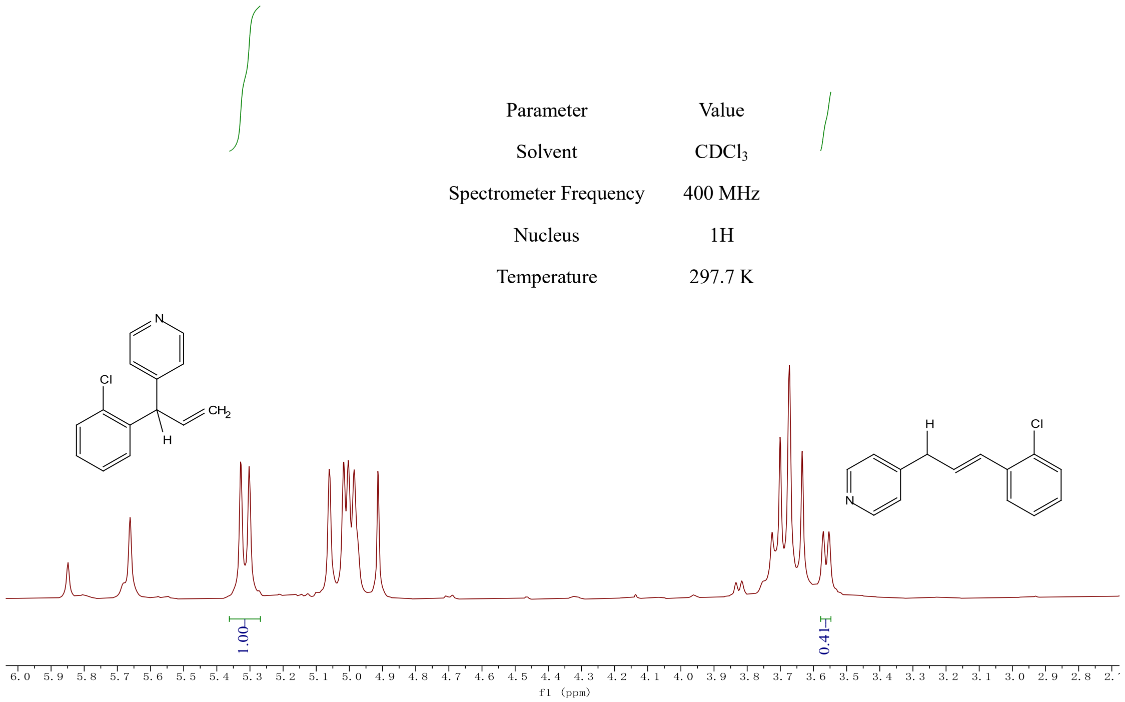

^{1}H NMR Spectrum of Crude Product **3o**

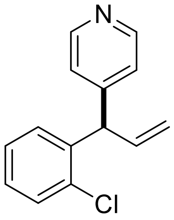

3o

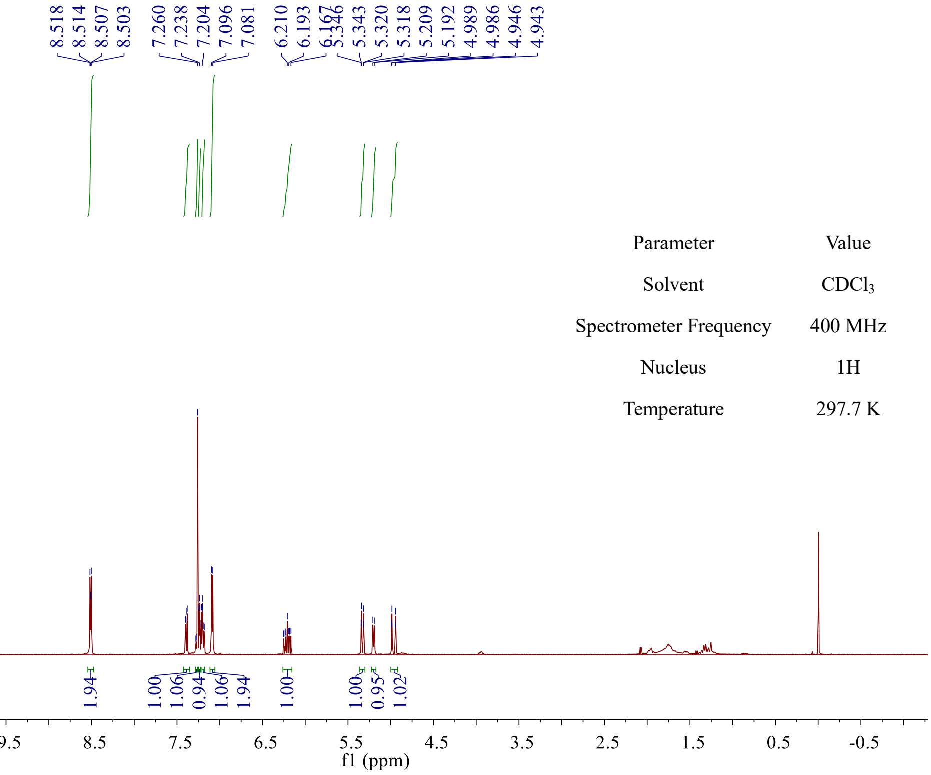

Parameter	Value
Solvent	$CDCl_3$
Spectrometer Frequency	400 MHz
Nucleus	1H
Temperature	297.7 K

^{1}H NMR Spectrum of Compound **3o**

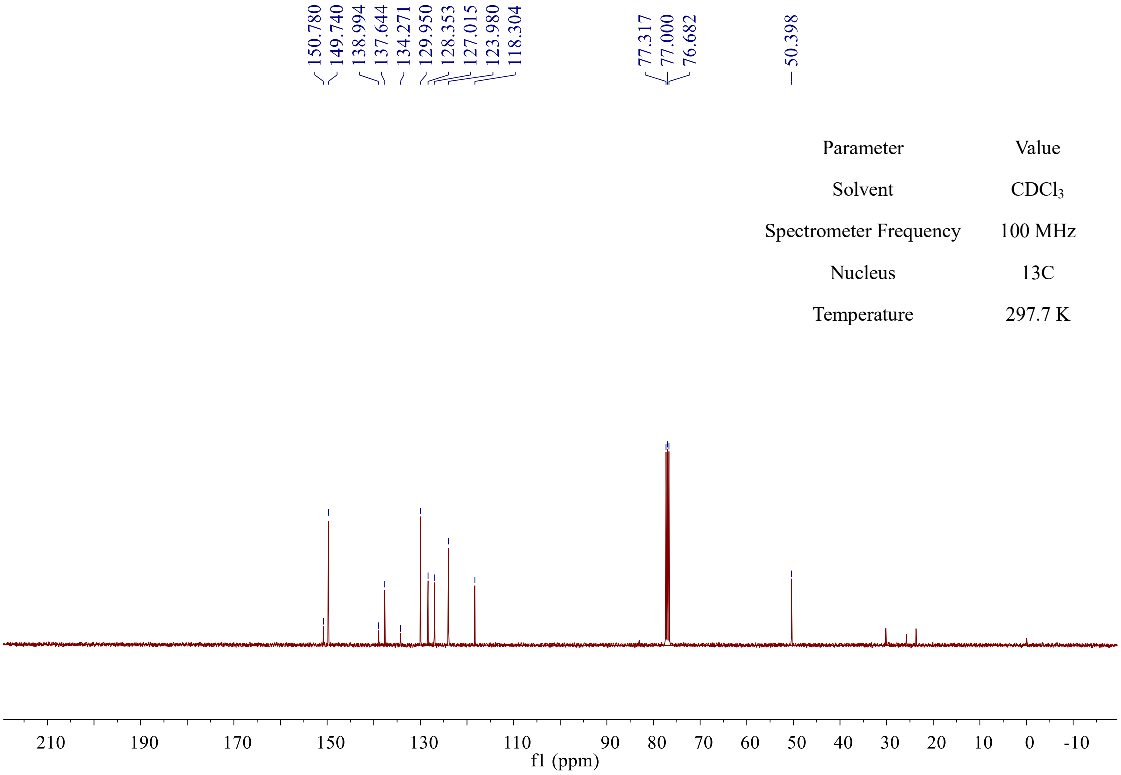

Parameter	Value
Solvent	$CDCl_3$
Spectrometer Frequency	100 MHz
Nucleus	13C
Temperature	297.7 K

^{13}C NMR Spectrum of Compound **3o**

Supplementary Figure 100**. NMR spectra of 3o**

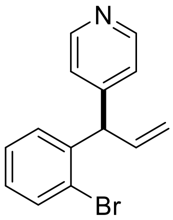

3p

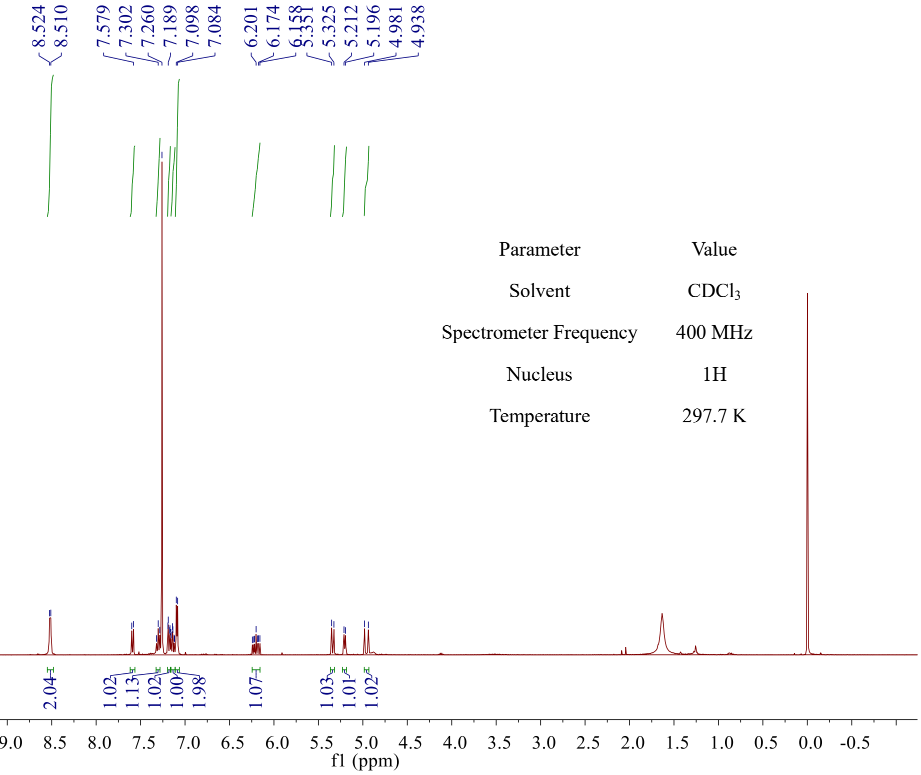

^{1}H NMR Spectrum of Compound **3p**

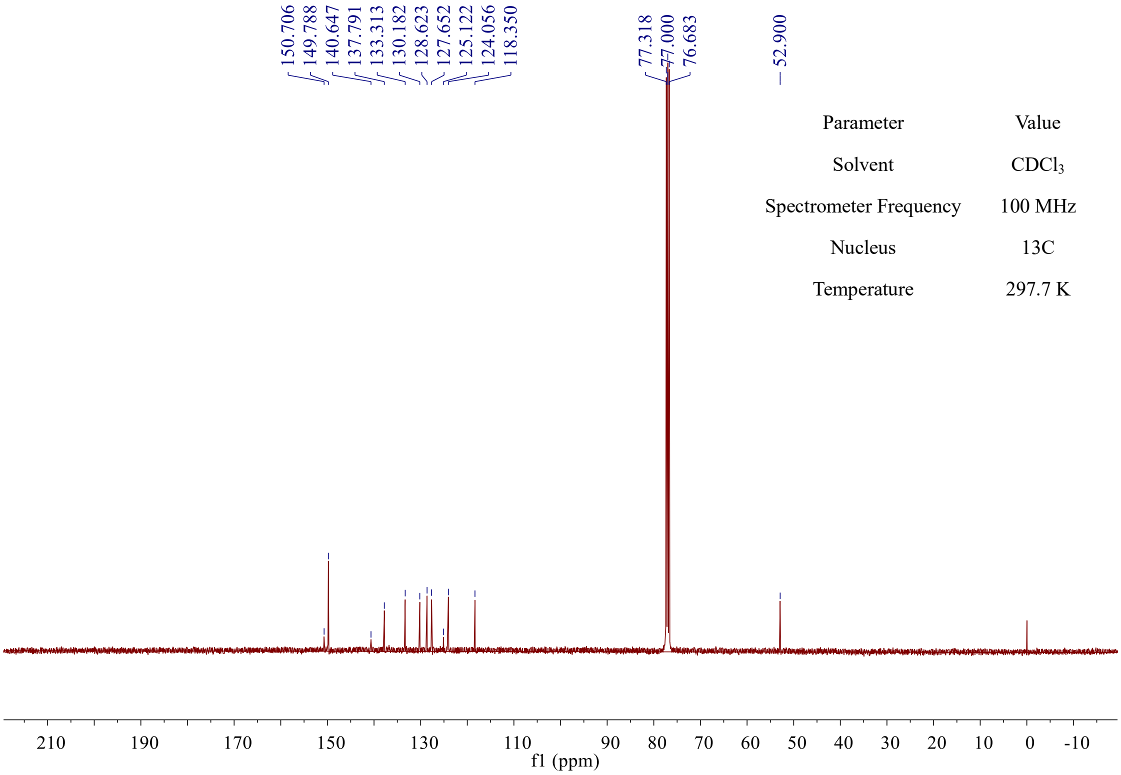

^{13}C NMR Spectrum of Compound **3p**

Supplementary Figure 101**. NMR spectra of 3p**

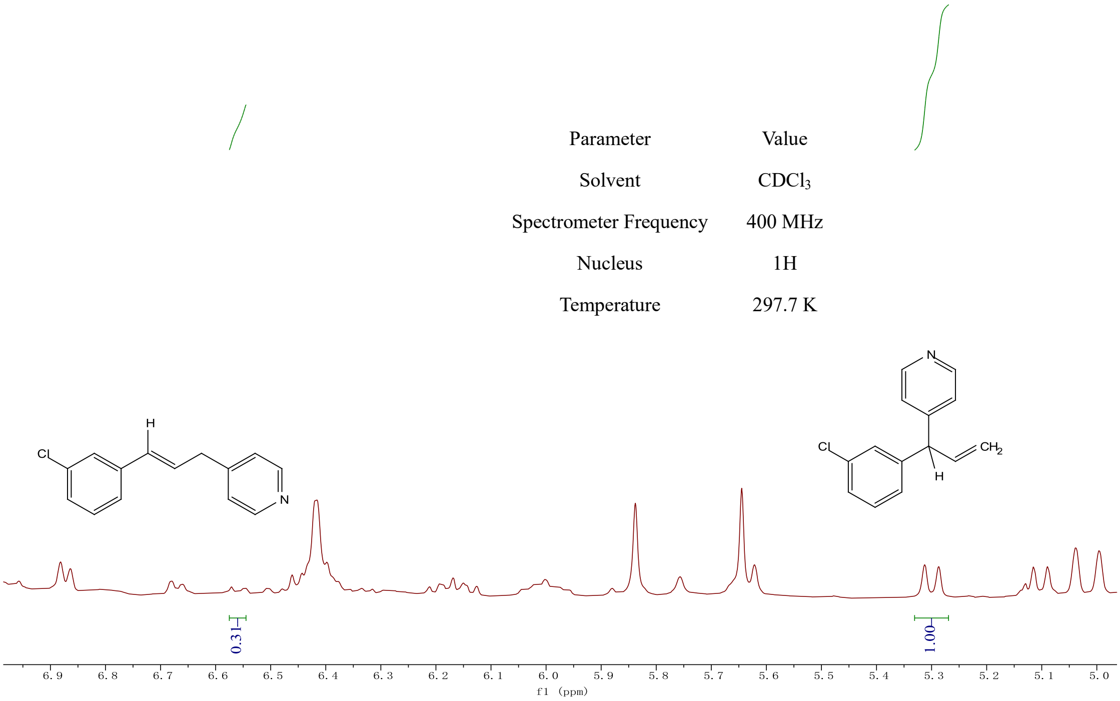

^{1}H NMR Spectrum of Crude Product **3q**

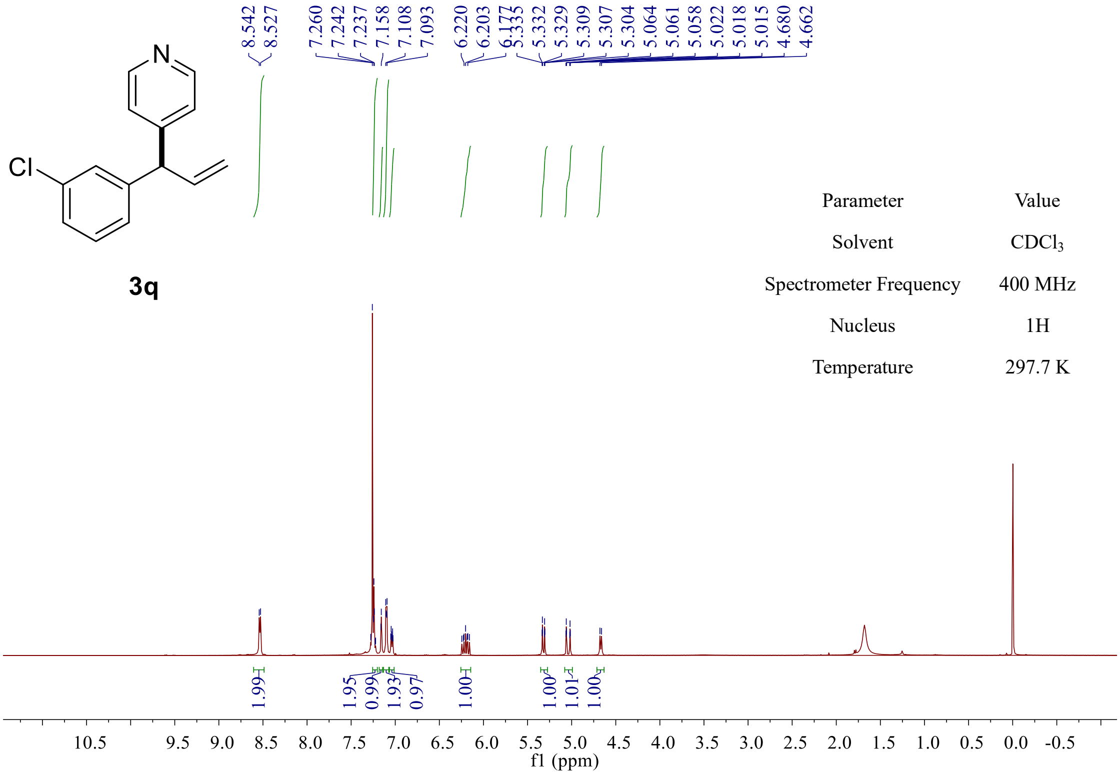

^{1}H NMR Spectrum of Compound **3q**

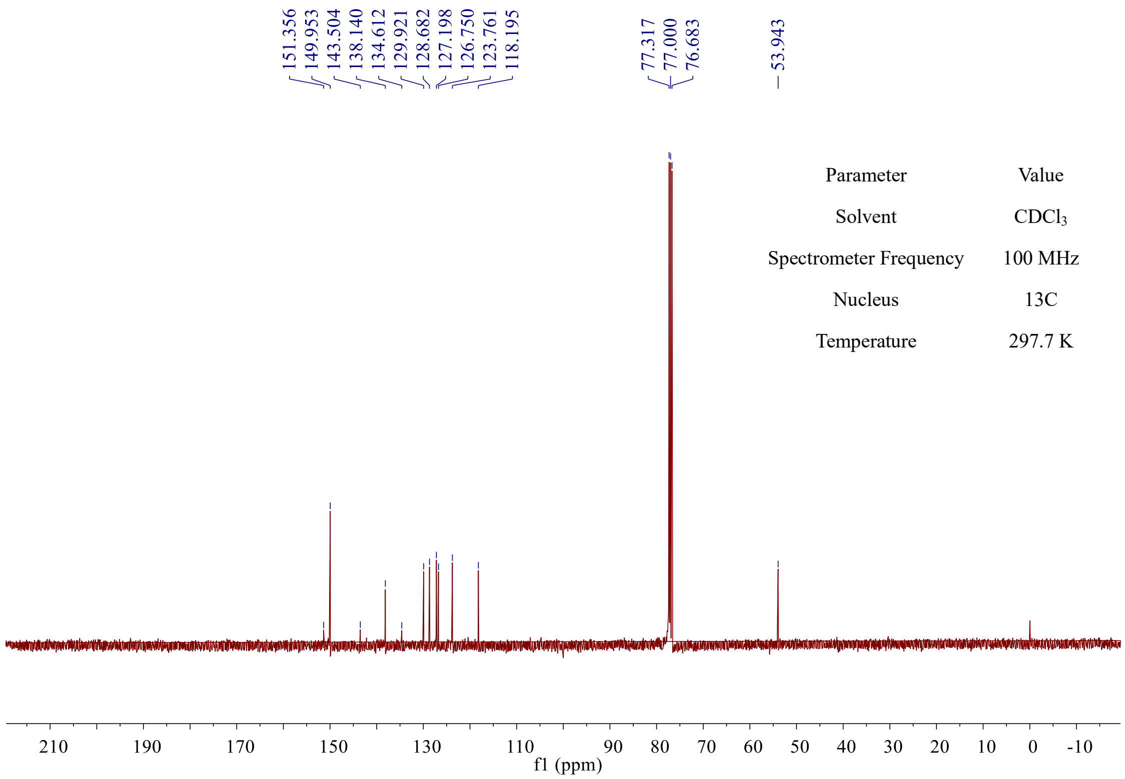

^{13}C NMR Spectrum of Compound **3q**

Supplementary Figure 102**. NMR spectra of 3q**

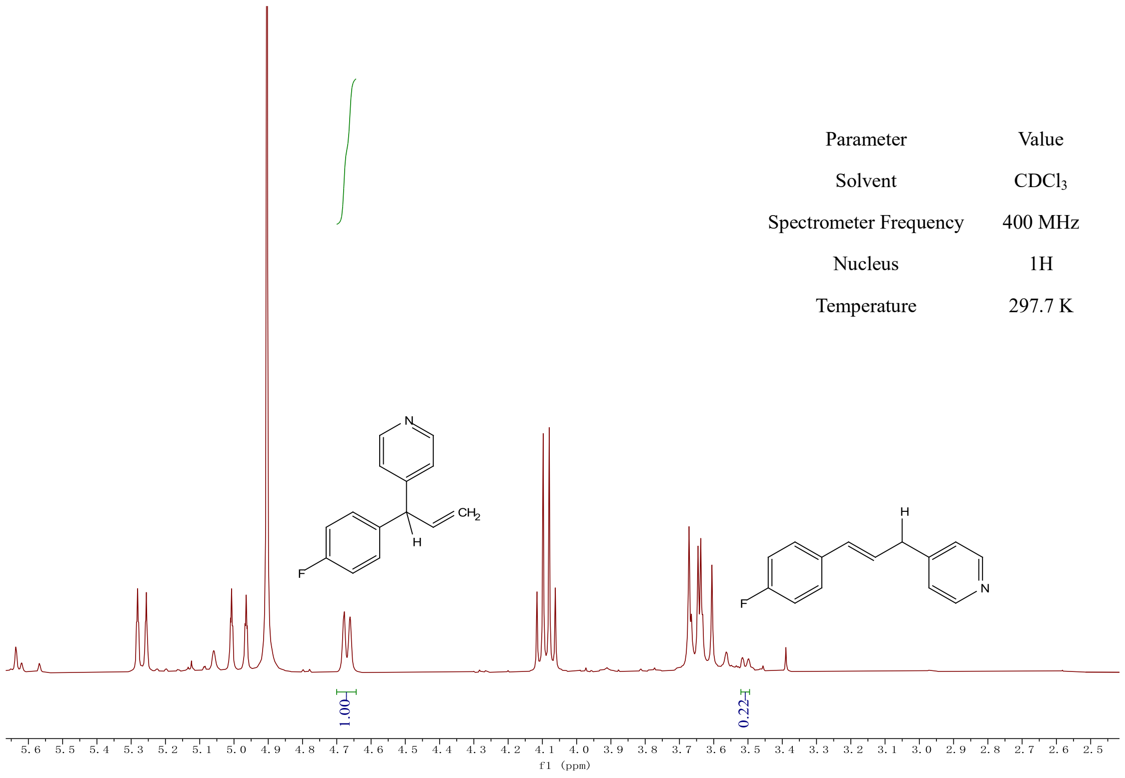

^{1}H NMR Spectrum of Crude Product **3r**

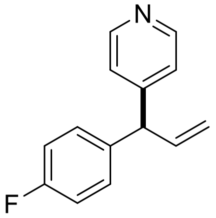

3r

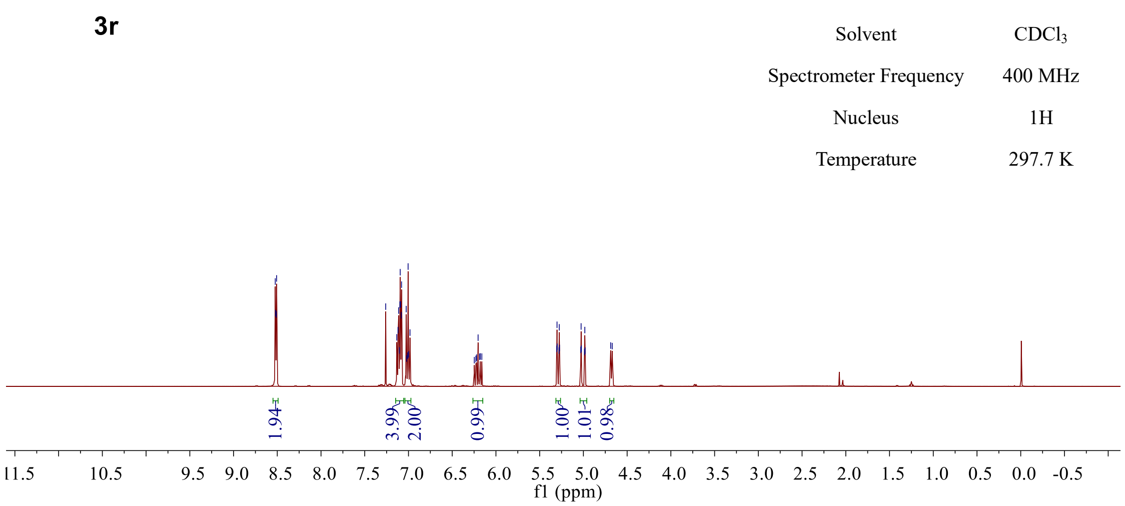

Parameter	Value
Solvent	$CDCl_3$
Spectrometer Frequency	400 MHz
Nucleus	1H
Temperature	297.7 K

^{1}H NMR Spectrum of Compound **3r**

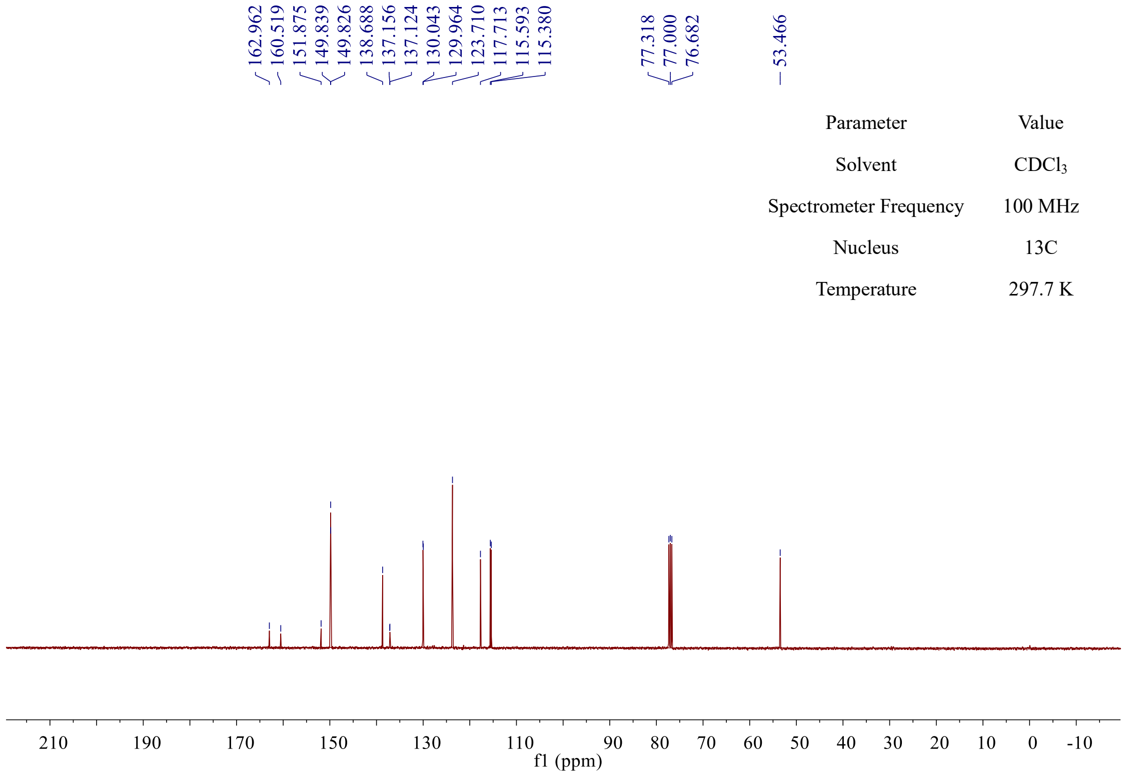

Parameter	Value
Solvent	$CDCl_3$
Spectrometer Frequency	100 MHz
Nucleus	13C
Temperature	297.7 K

^{13}C NMR Spectrum of Compound **3r**

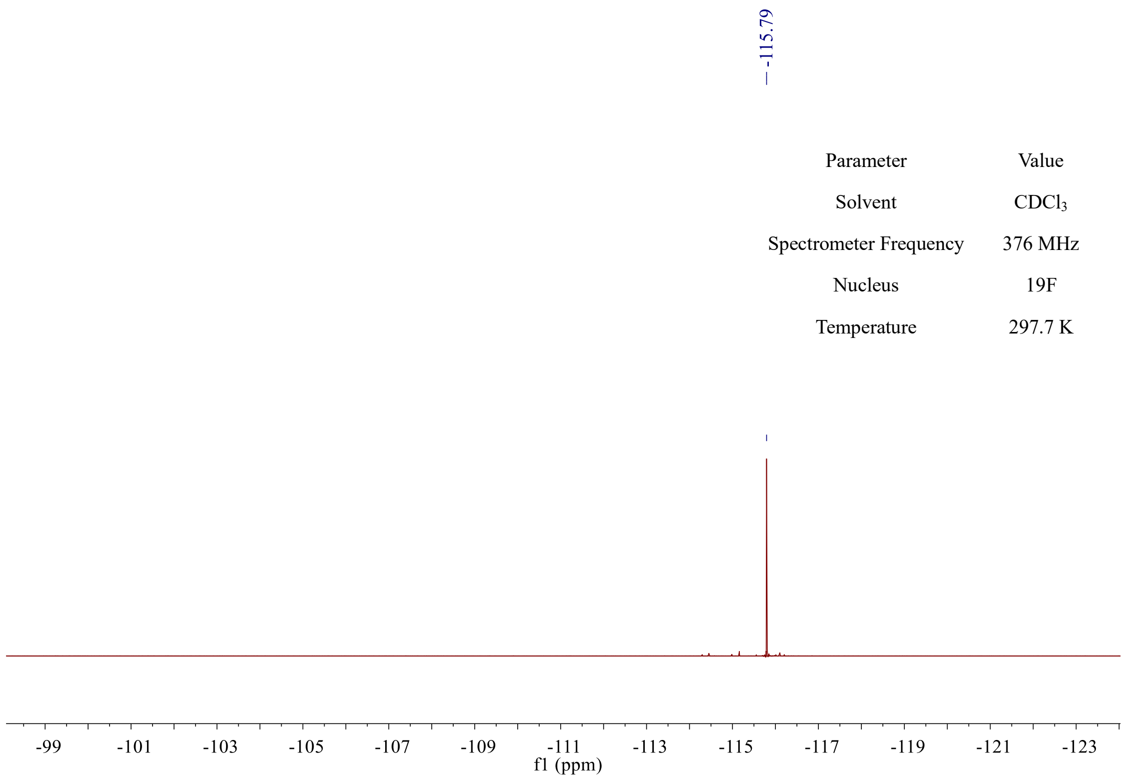

^{19}F NMR Spectrum of Compound **3r**

Supplementary Figure 103**. NMR spectra of 3r**

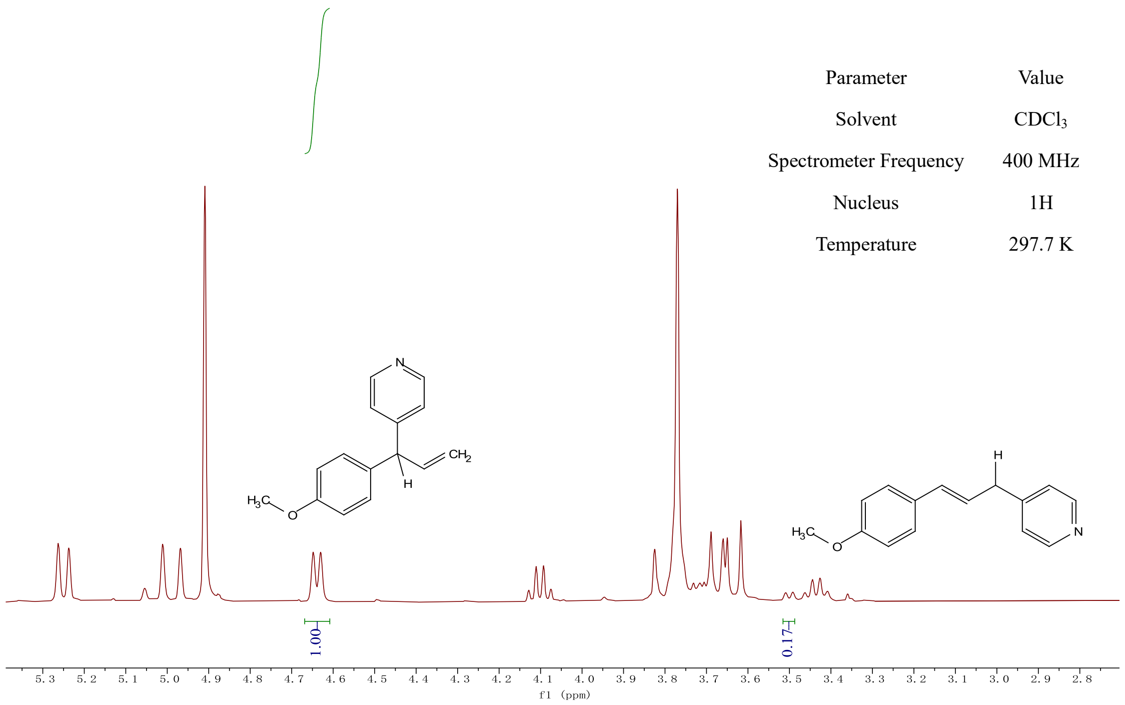

^{1}H NMR Spectrum of Crude Product **3s**

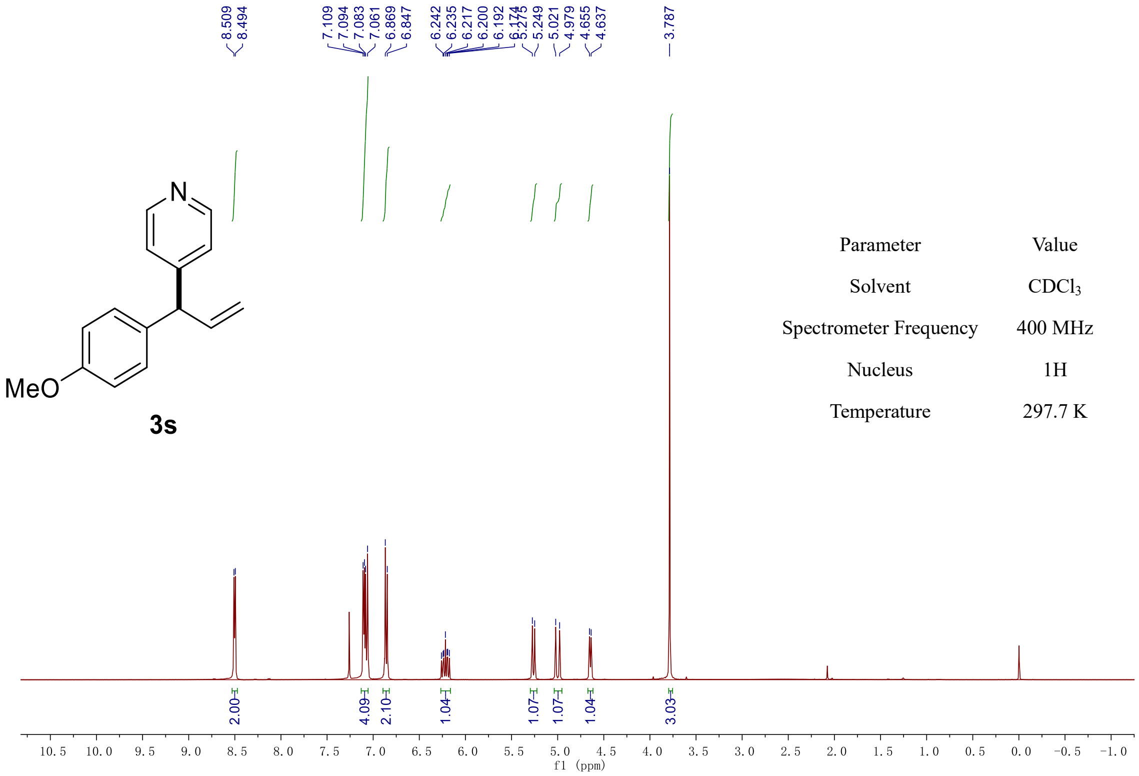

^{1}H NMR Spectrum of Compound **3s**

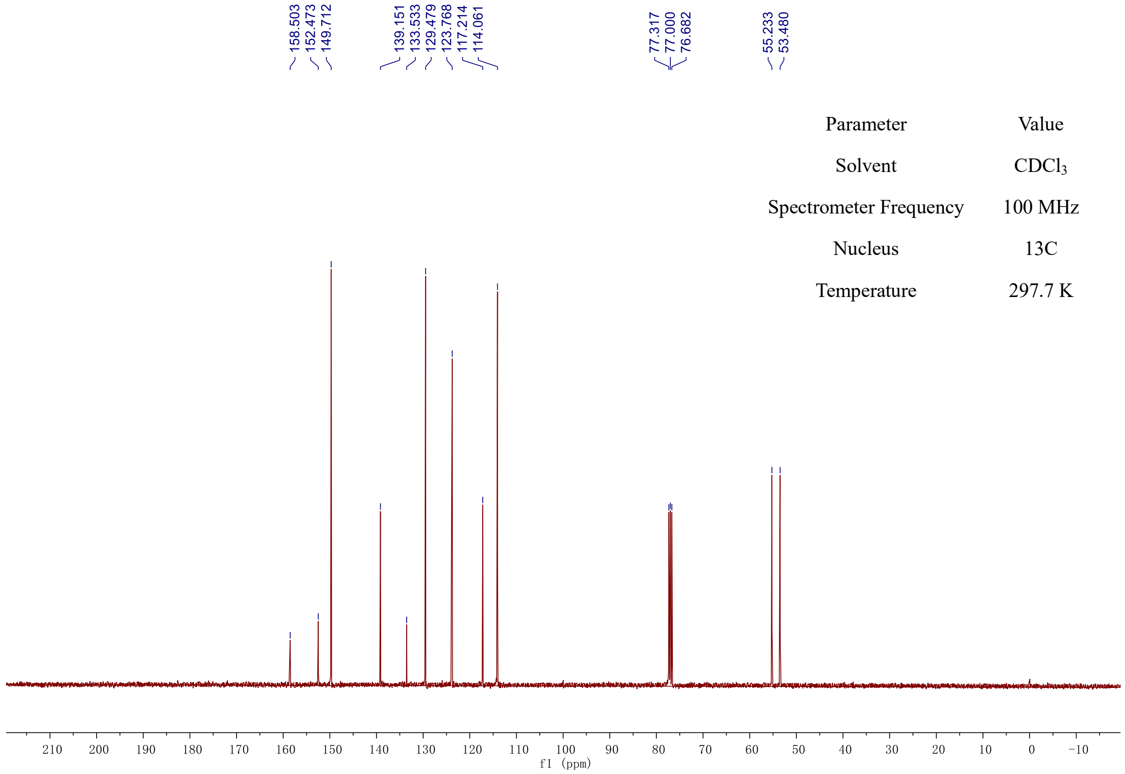

^{13}C NMR Spectrum of Compound **3s**

Supplementary Figure 104**. NMR spectra of 3s**

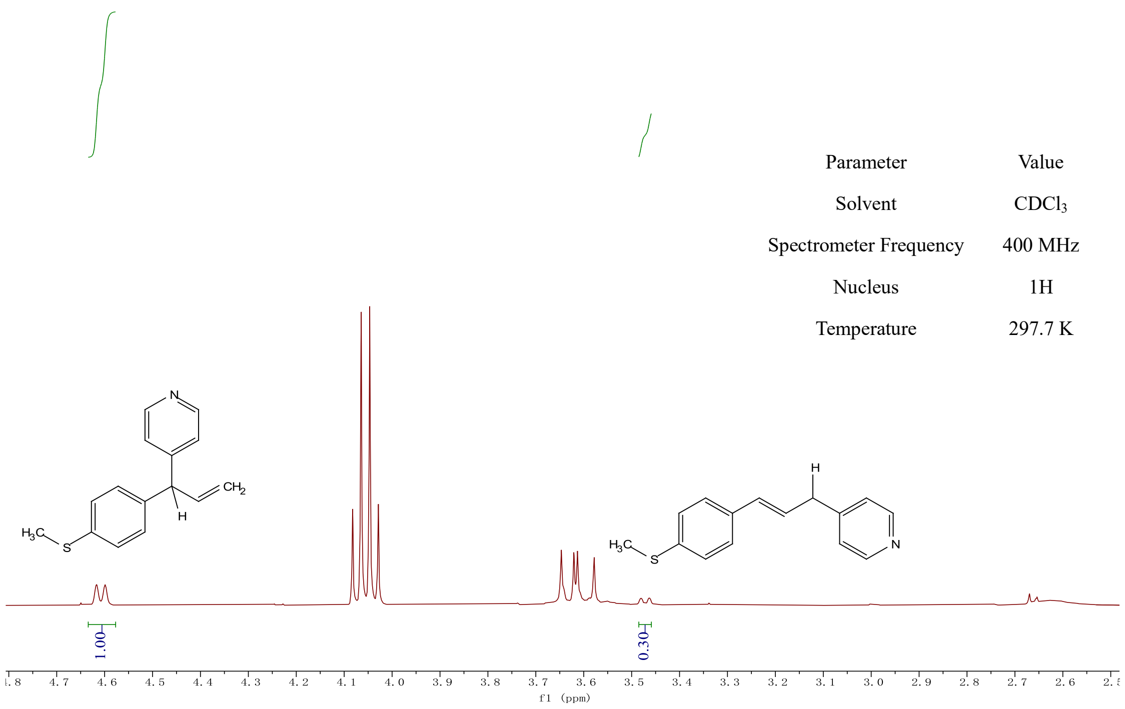

^{1}H NMR Spectrum of Crude Product **3t**

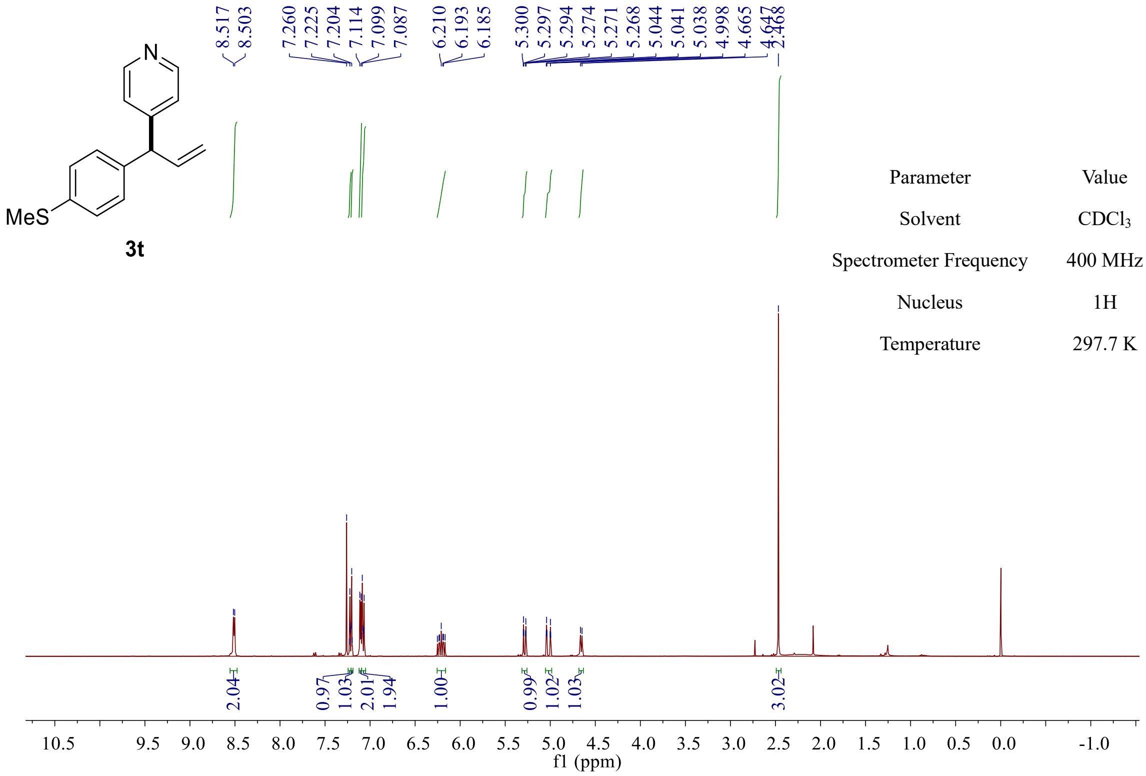

^{1}H NMR Spectrum of Compound **3t**

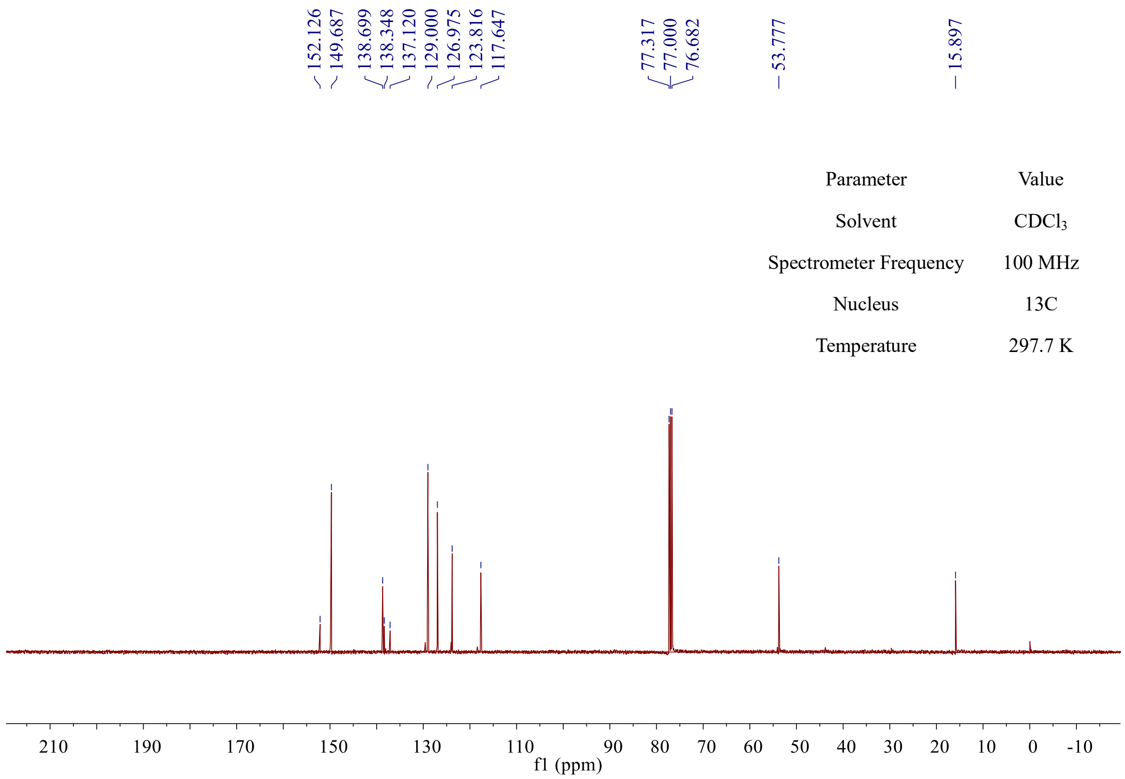

^{13}C NMR Spectrum of Compound **3t**

Supplementary Figure 105**. NMR spectra of 3t**

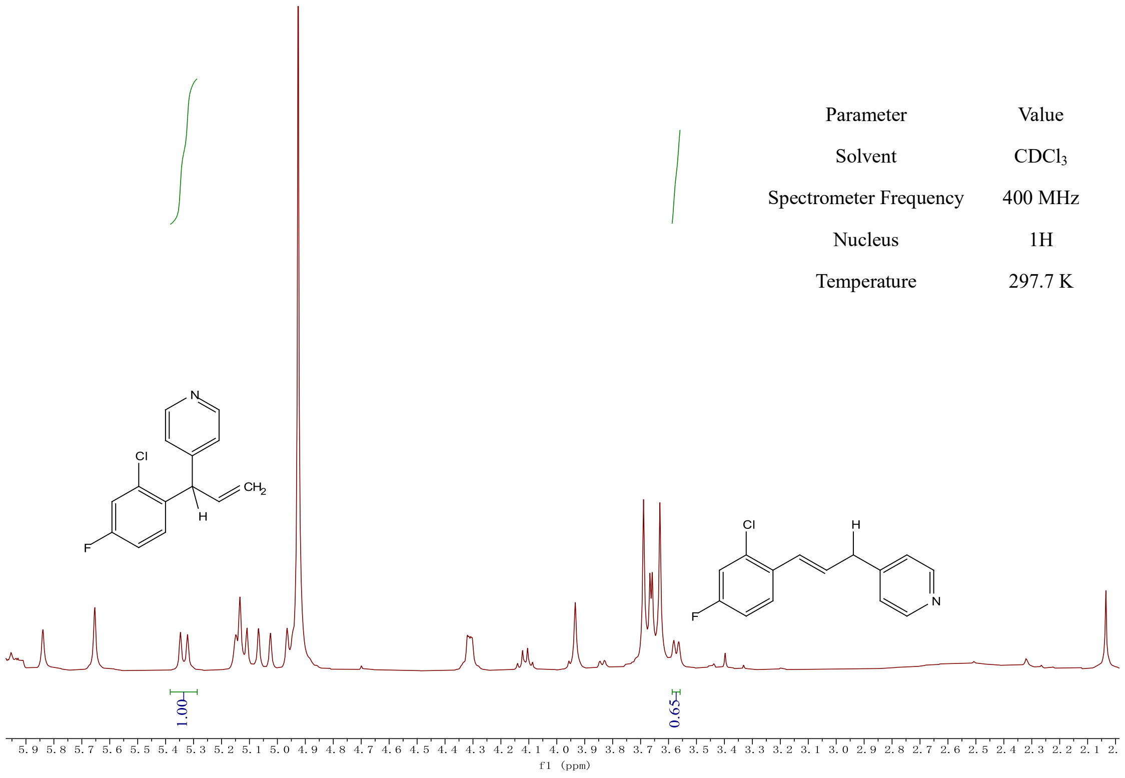

^{1}H NMR Spectrum of Crude Product **3u**

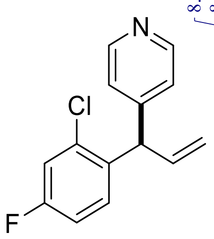

3u

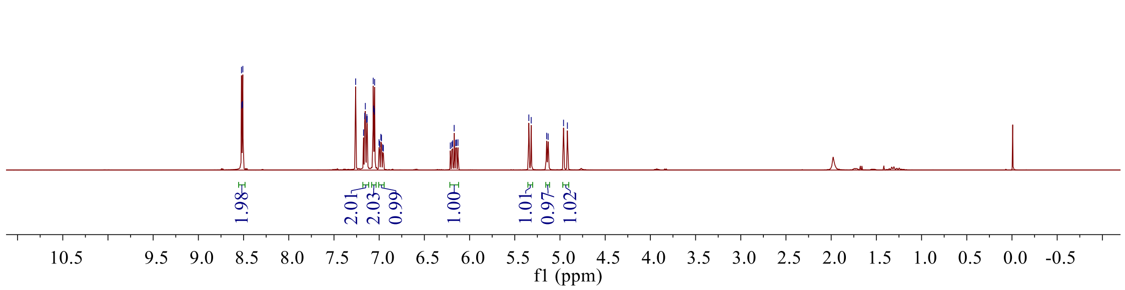

Parameter	Value
Solvent	$CDCl_3$
Spectrometer Frequency	400 MHz
Nucleus	1H
Temperature	297.7 K

^{1}H NMR Spectrum of Compound **3u**

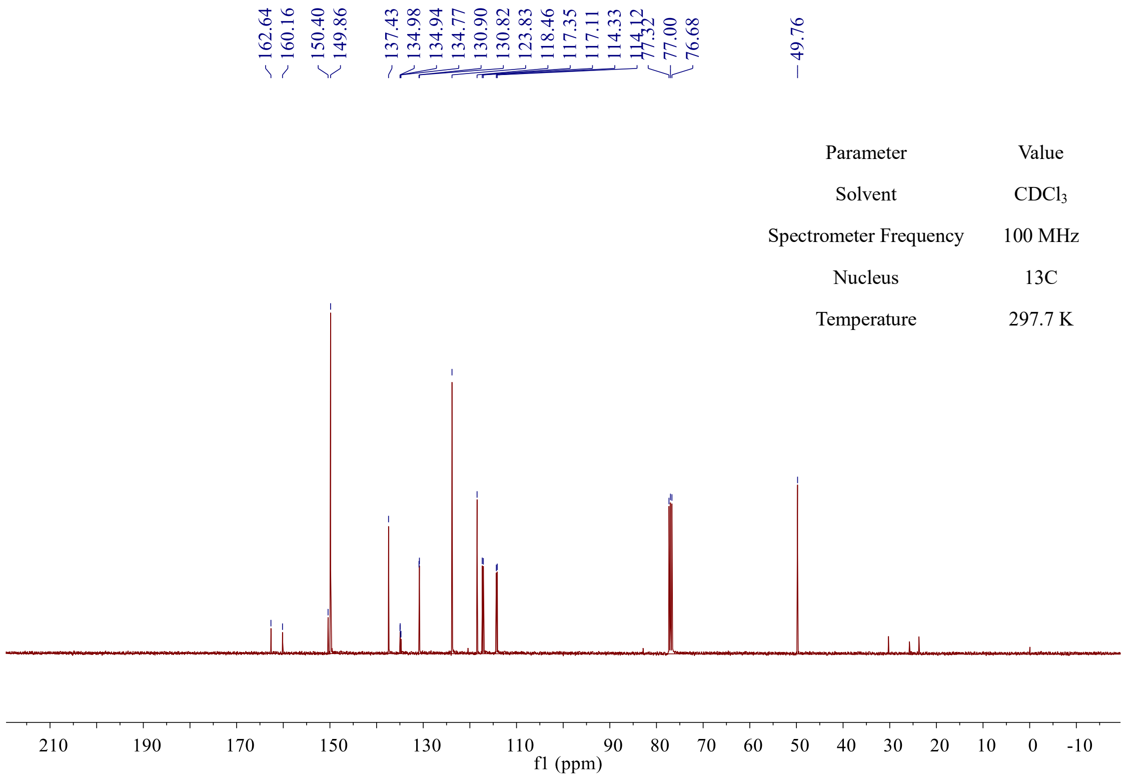

Parameter	Value
Solvent	$CDCl_3$
Spectrometer Frequency	100 MHz
Nucleus	13C
Temperature	297.7 K

^{13}C NMR Spectrum of Compound **3u**

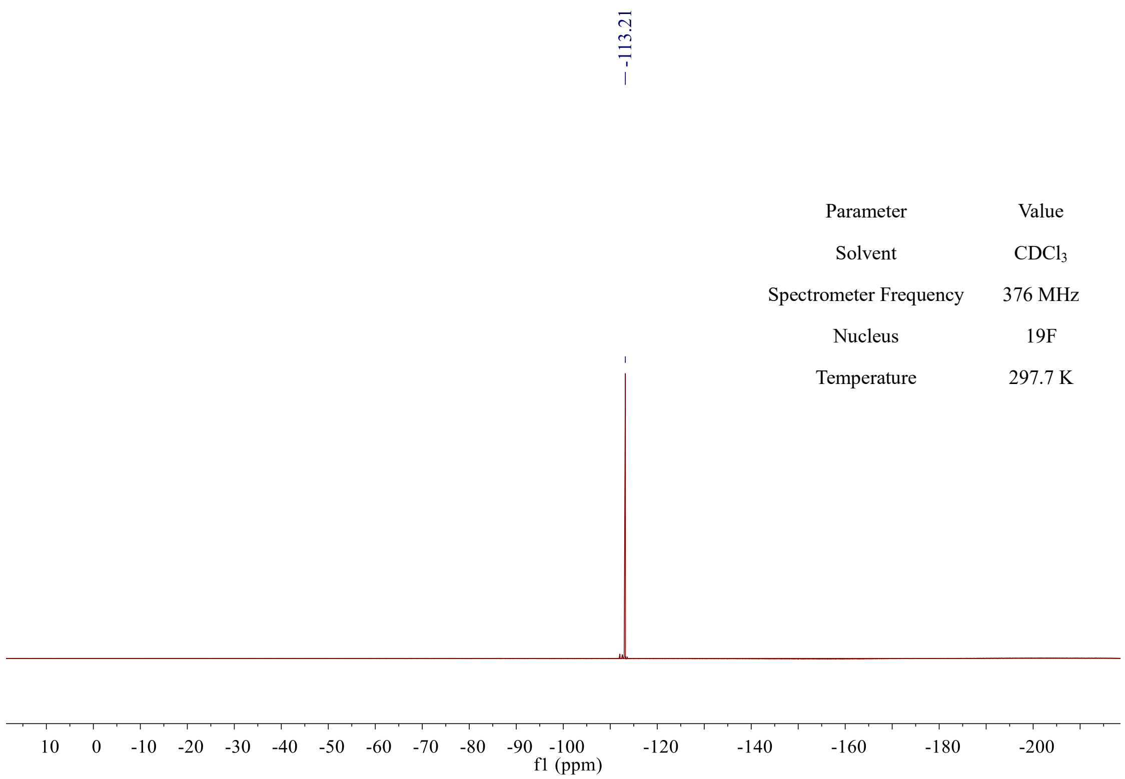

^{19}F NMR Spectrum of Compound **3u**

Supplementary Figure 106**. NMR spectra of 3u**

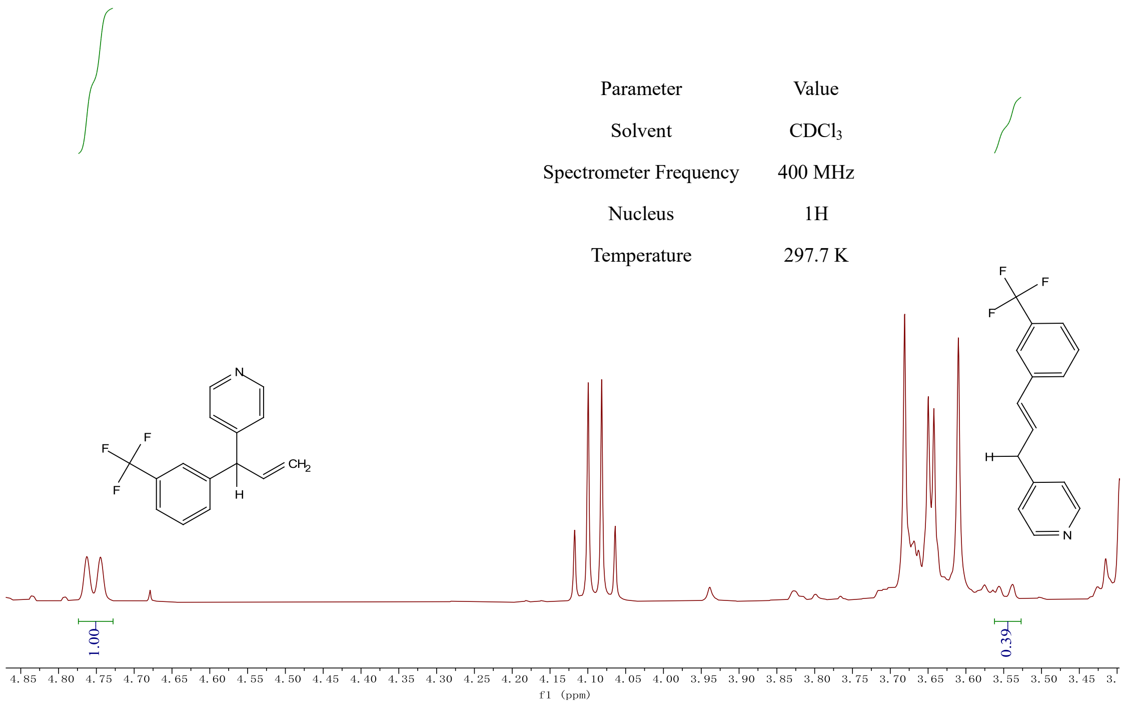

^{1}H NMR Spectrum of Crude Product **3v**

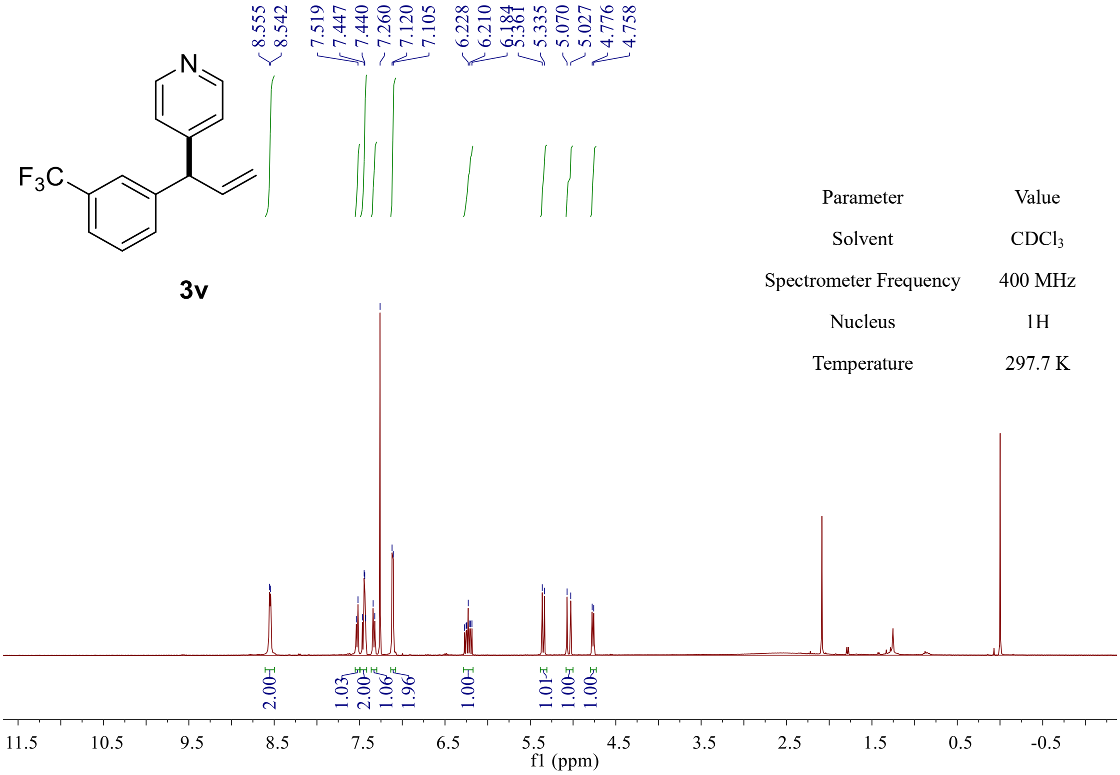

3v

Parameter	Value
Solvent	$CDCl_3$
Spectrometer Frequency	400 MHz
Nucleus	1H
Temperature	297.7 K

^{1}H NMR Spectrum of Compound **3v**

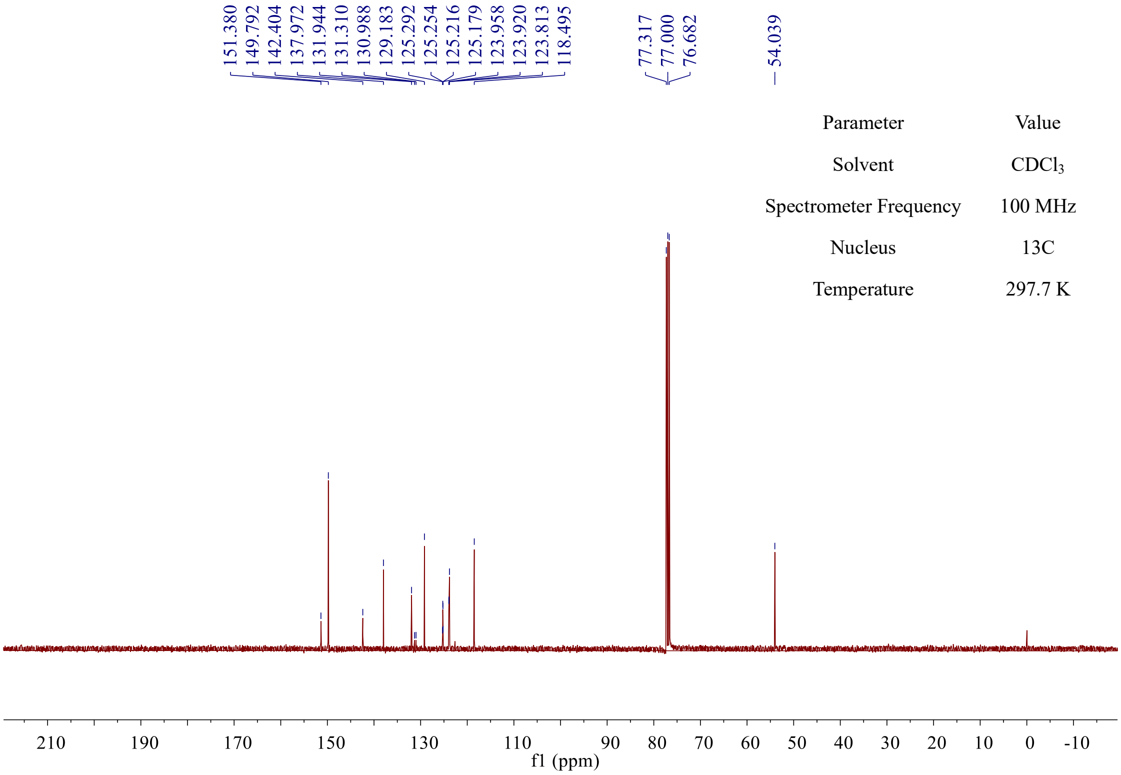

Parameter	Value
Solvent	$CDCl_3$
Spectrometer Frequency	100 MHz
Nucleus	13C
Temperature	297.7 K

^{13}C NMR Spectrum of Compound **3v**

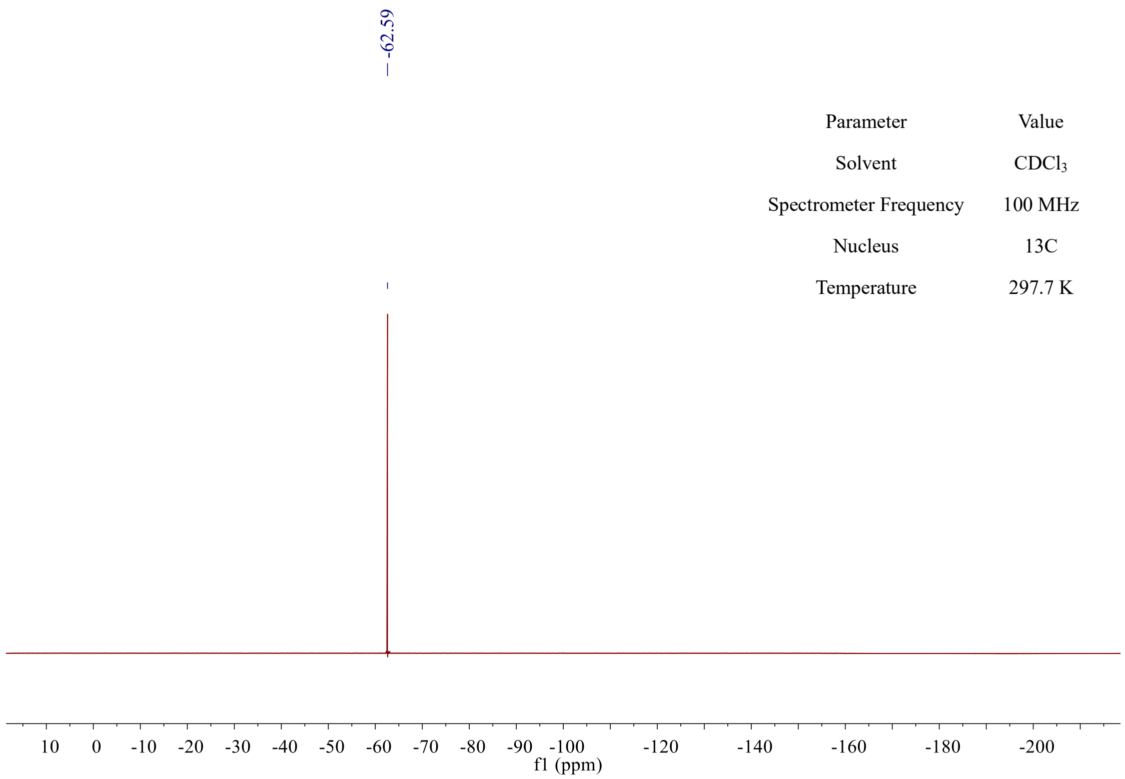

^{19}F NMR Spectrum of Compound **3v**

Supplementary Figure 107. **NMR spectra of 3v**

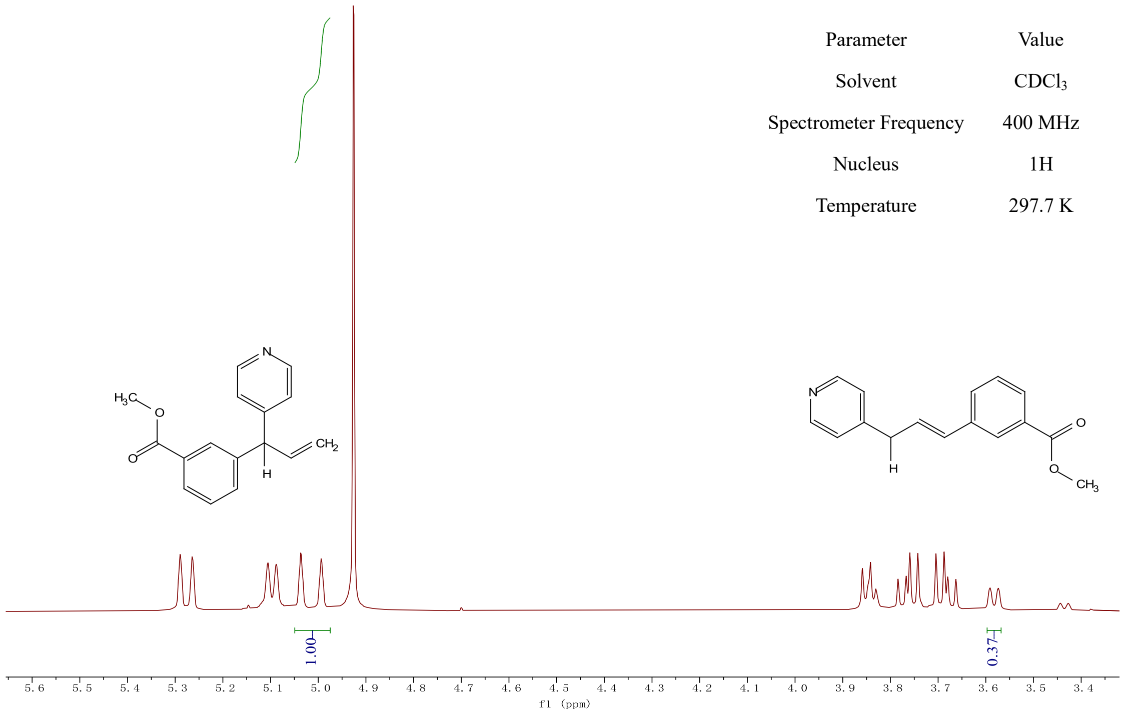

^{1}H NMR Spectrum of Crude Product **3w**

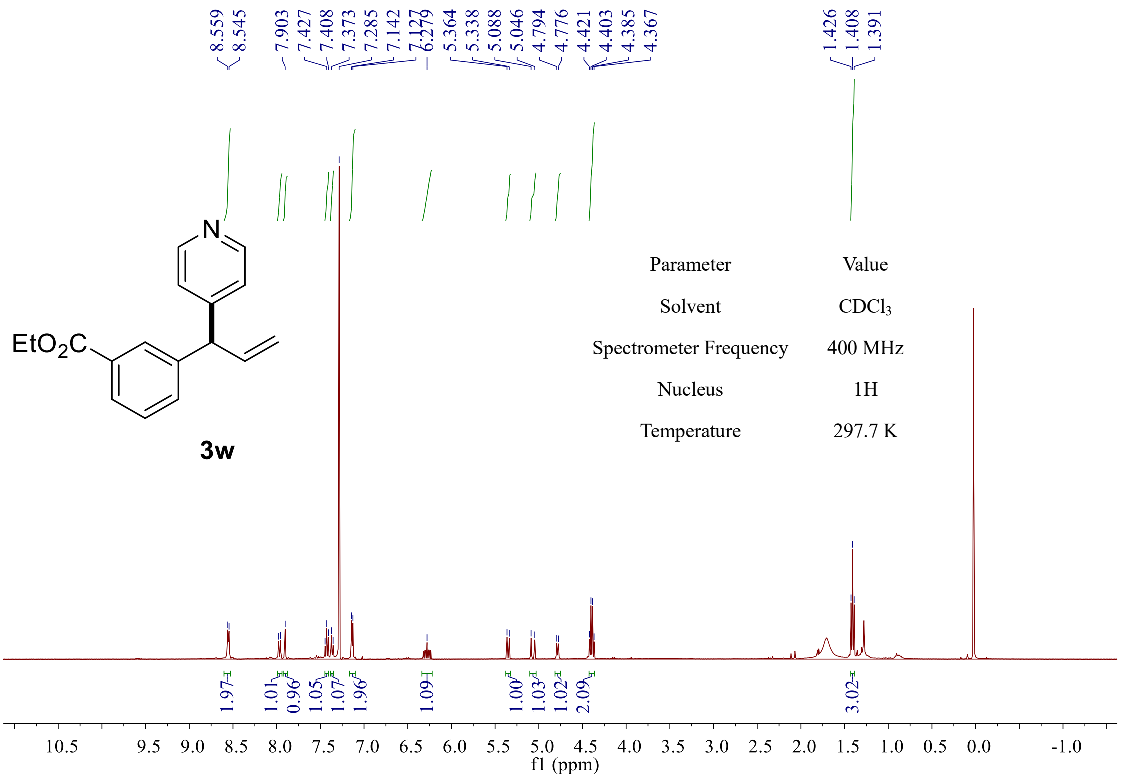

^{1}H NMR Spectrum of Compound **3w**

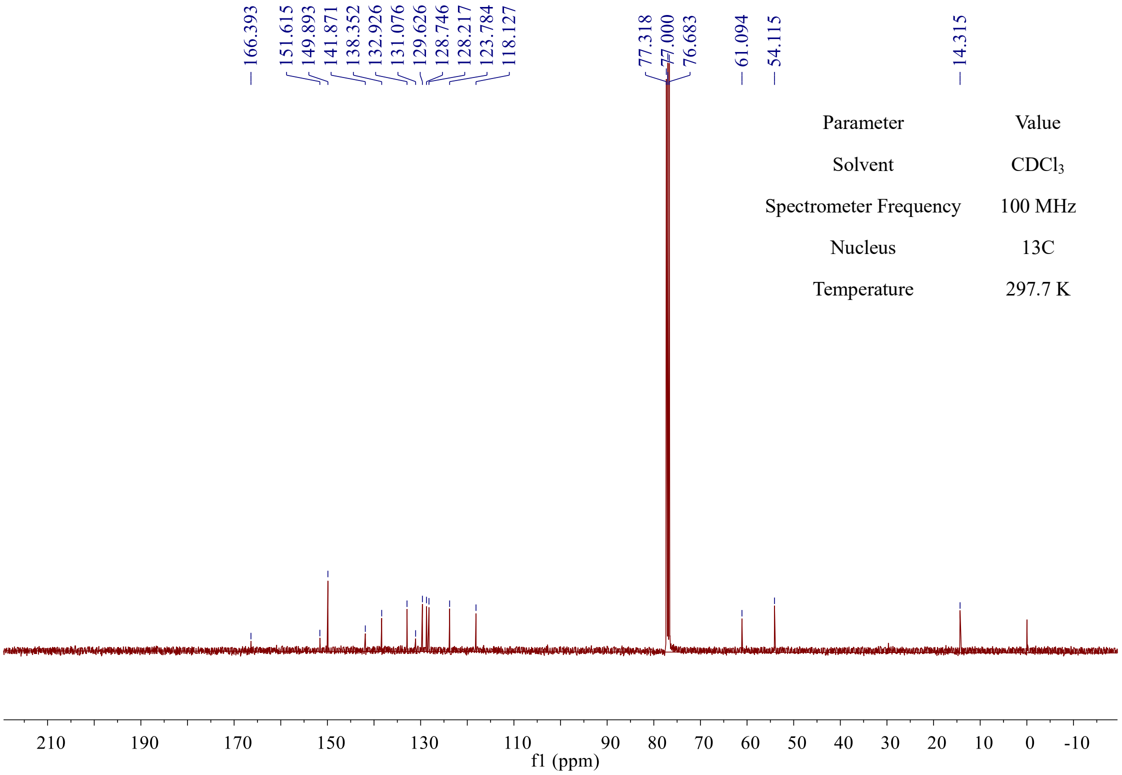

^{13}C NMR Spectrum of Compound **3w**

Supplementary Figure 108**. NMR spectra of 3w**

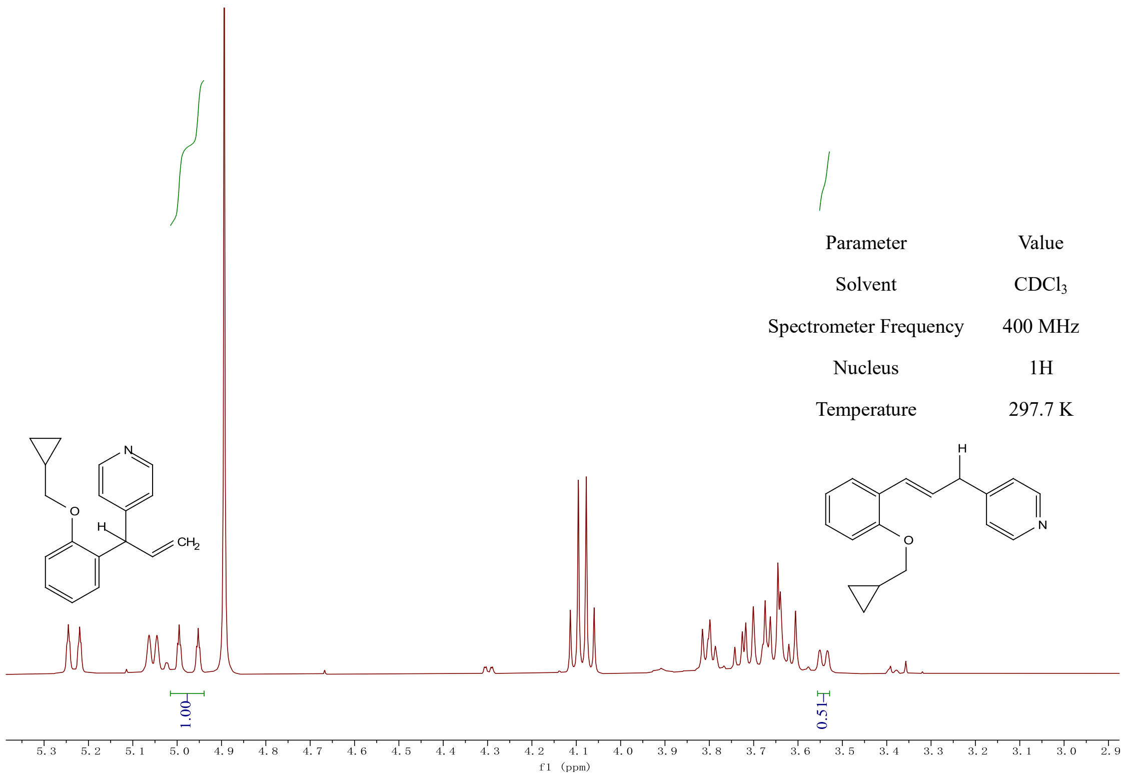

^{1}H NMR Spectrum of Crude Product **3x**

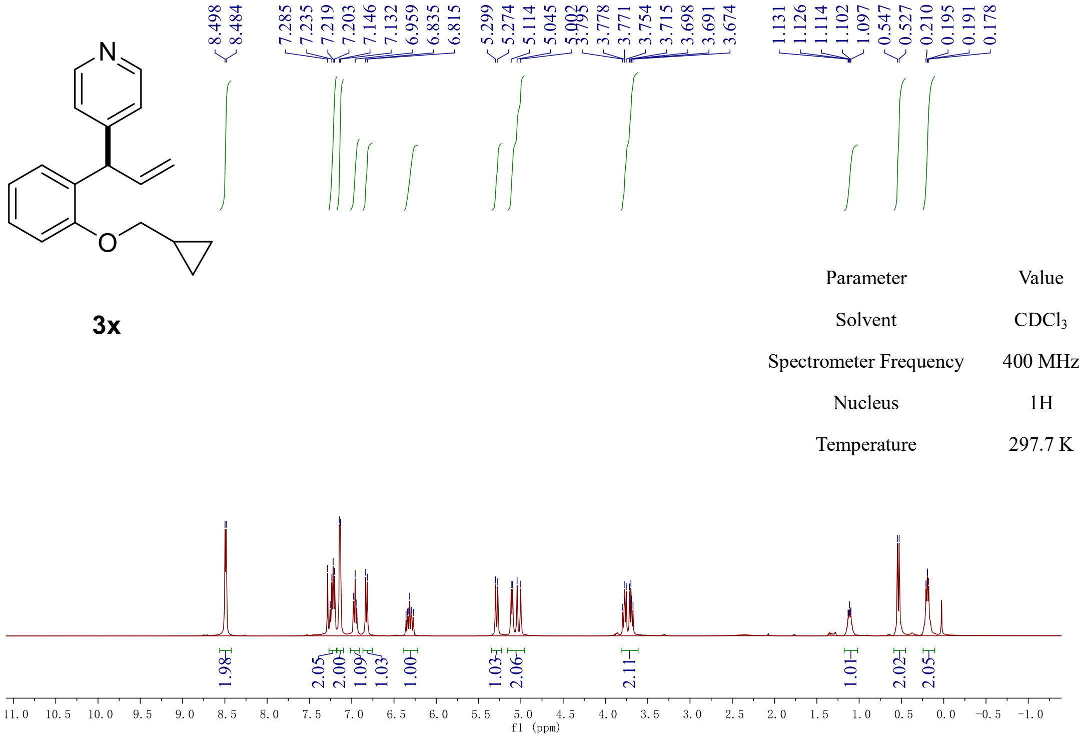

^{1}H NMR Spectrum of Compound **3x**

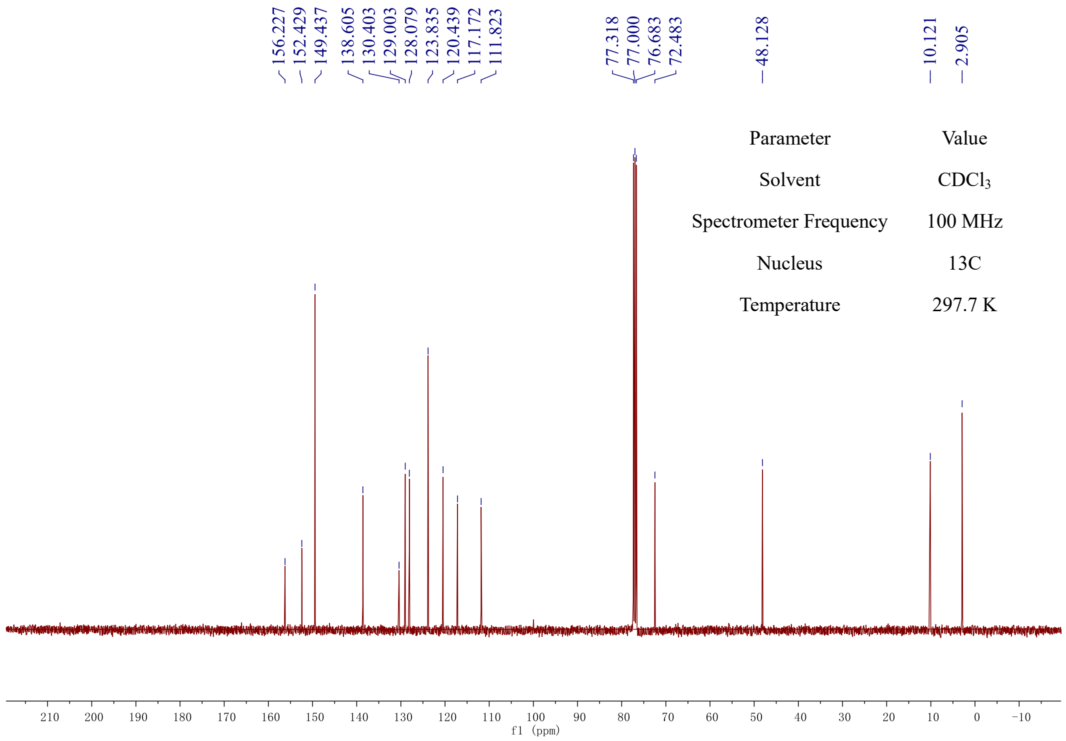

Parameter	Value
Solvent	$CDCl_3$
Spectrometer Frequency	100 MHz
Nucleus	13C
Temperature	297.7 K

^{13}C NMR Spectrum of Compound **3x**

Supplementary Figure 109**. NMR spectra of 3x**

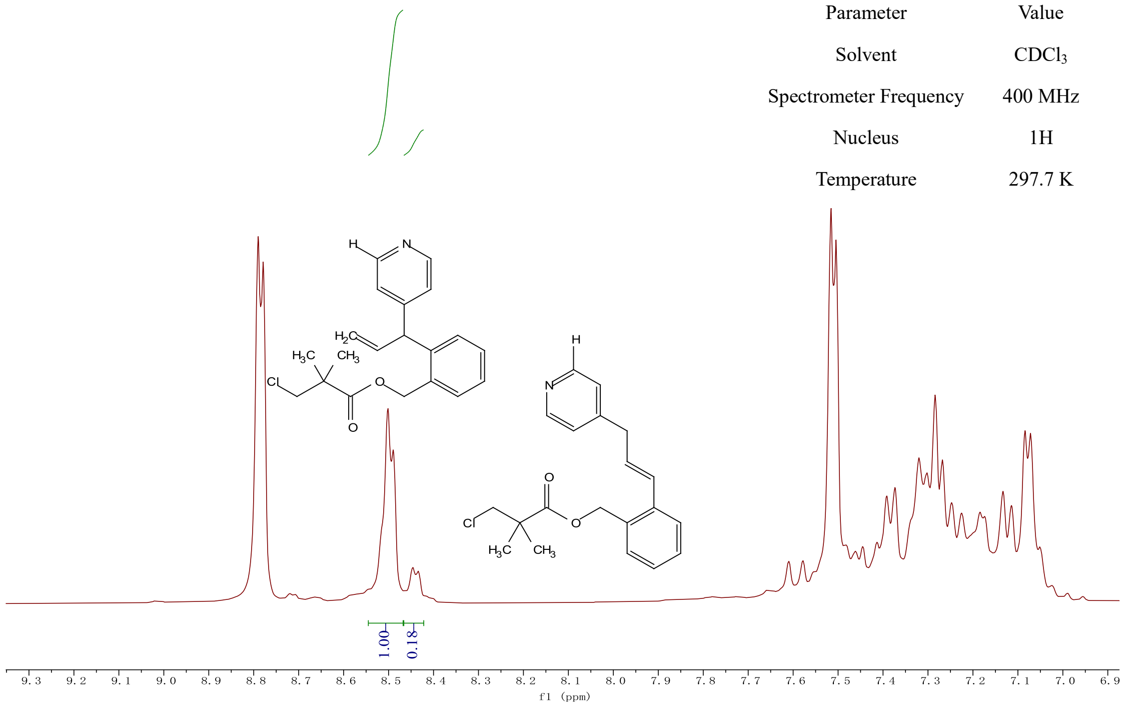

Parameter	Value
Solvent	$CDCl_3$
Spectrometer Frequency	400 MHz
Nucleus	1H
Temperature	297.7 K

^{1}H NMR Spectrum of Crude Product **3y**

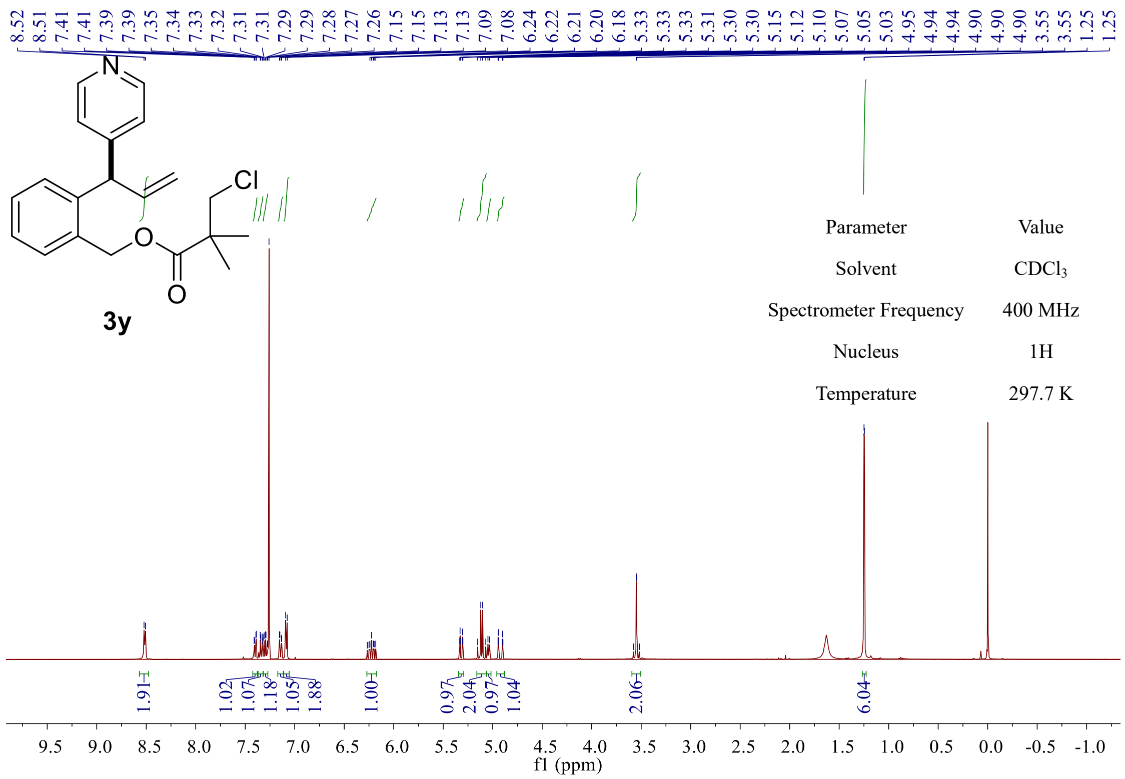

^{1}H NMR Spectrum of Compound **3y**

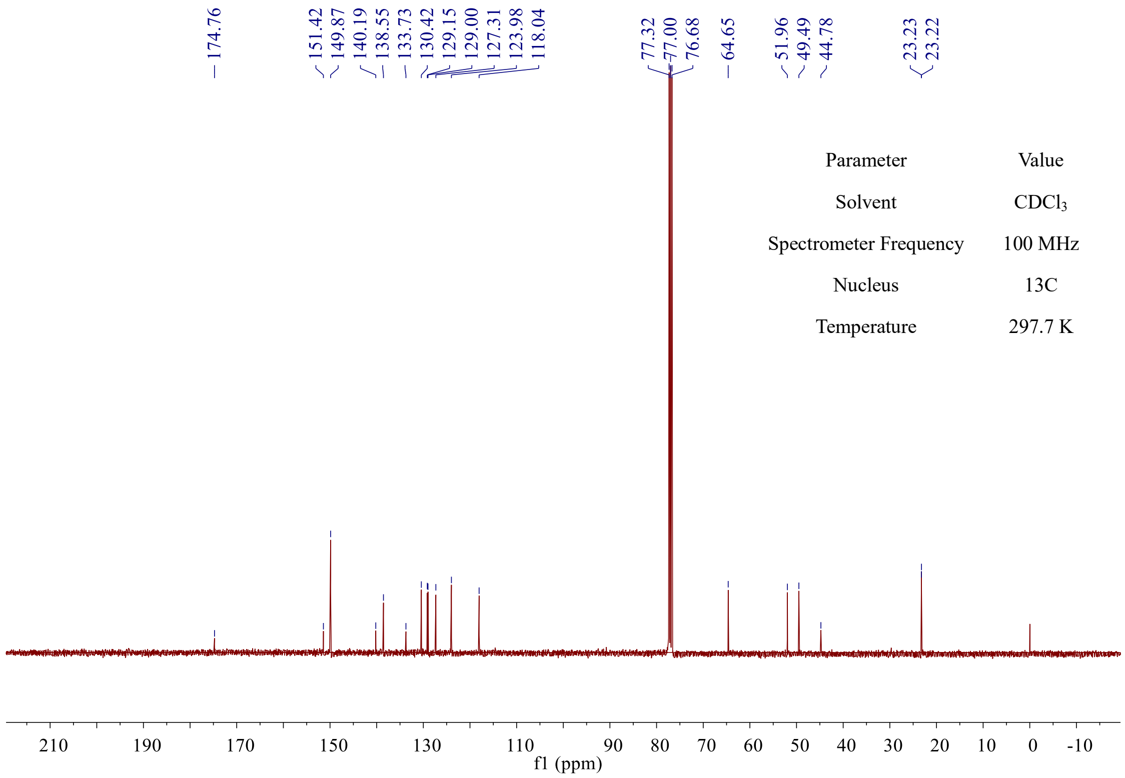

^{13}C NMR Spectrum of Compound **3y**

Supplementary Figure 110**. NMR spectra of 3y**

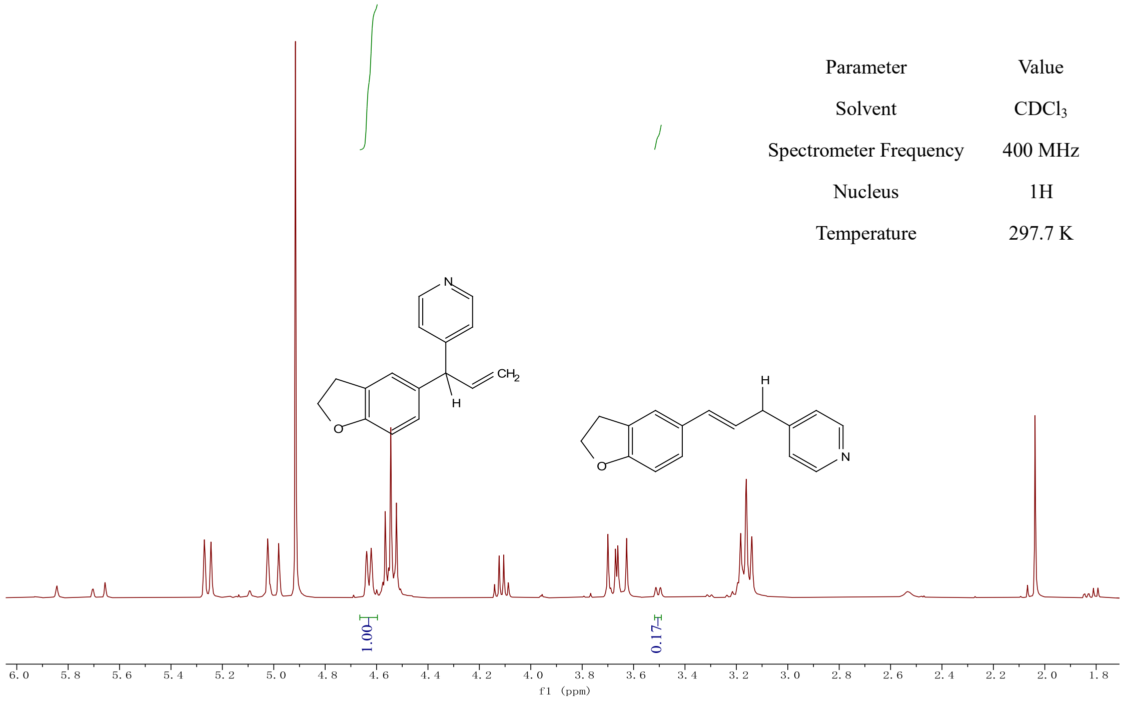

^{1}H NMR Spectrum of Crude Product **3z**

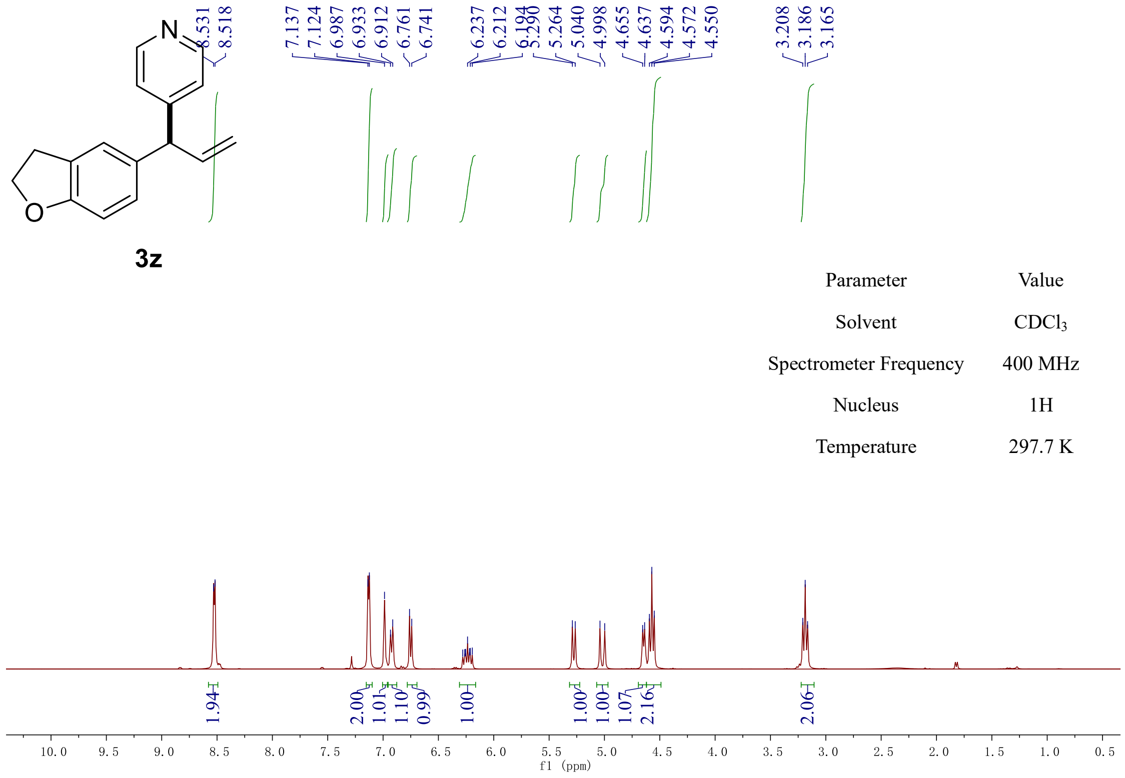

^{1}H NMR Spectrum of Compound **3z**

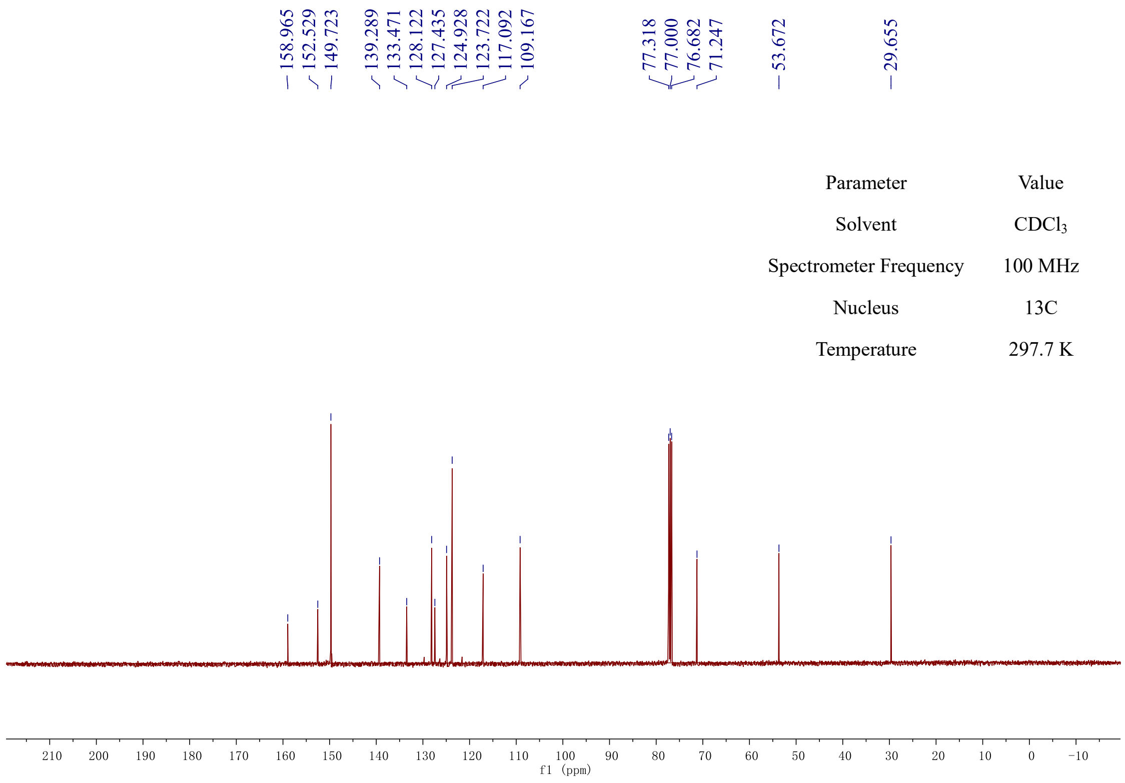

Parameter	Value
Solvent	$CDCl_3$
Spectrometer Frequency	100 MHz
Nucleus	13C
Temperature	297.7 K

^{13}C NMR Spectrum of Compound **3z**

Supplementary Figure 111**. NMR spectra of 3z**

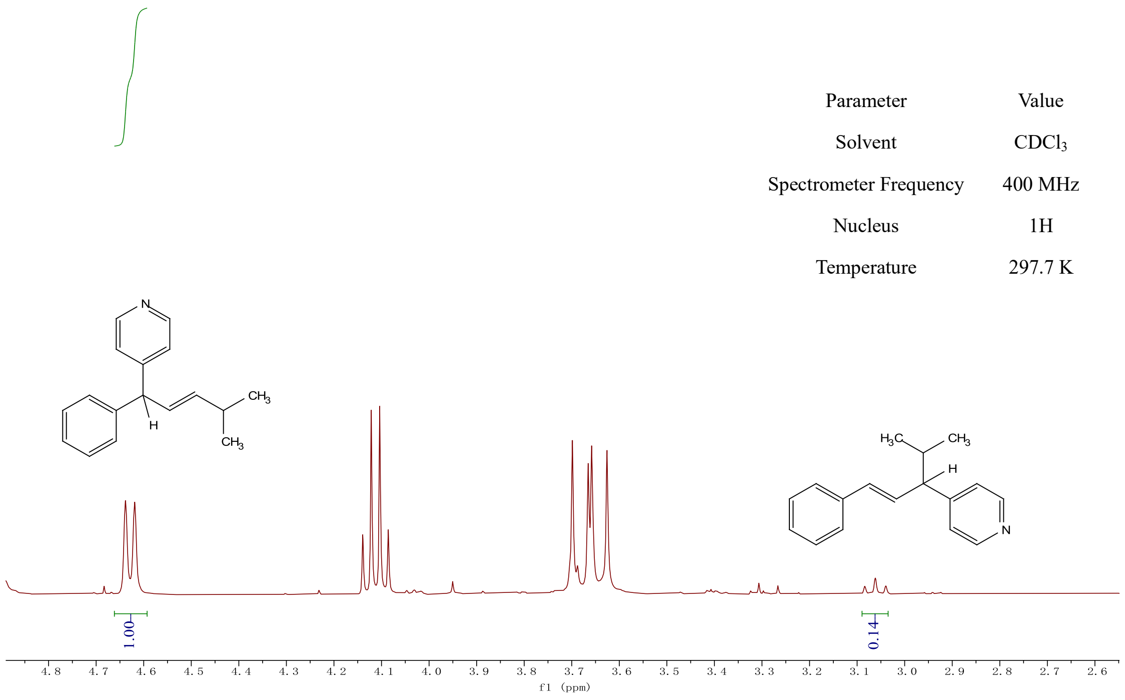

Parameter	Value
Solvent	$CDCl_3$
Spectrometer Frequency	400 MHz
Nucleus	1H
Temperature	297.7 K

^{1}H NMR Spectrum of Crude Product **3aa**

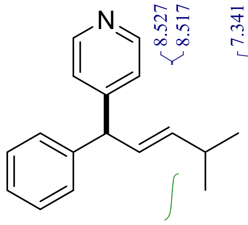

3aa

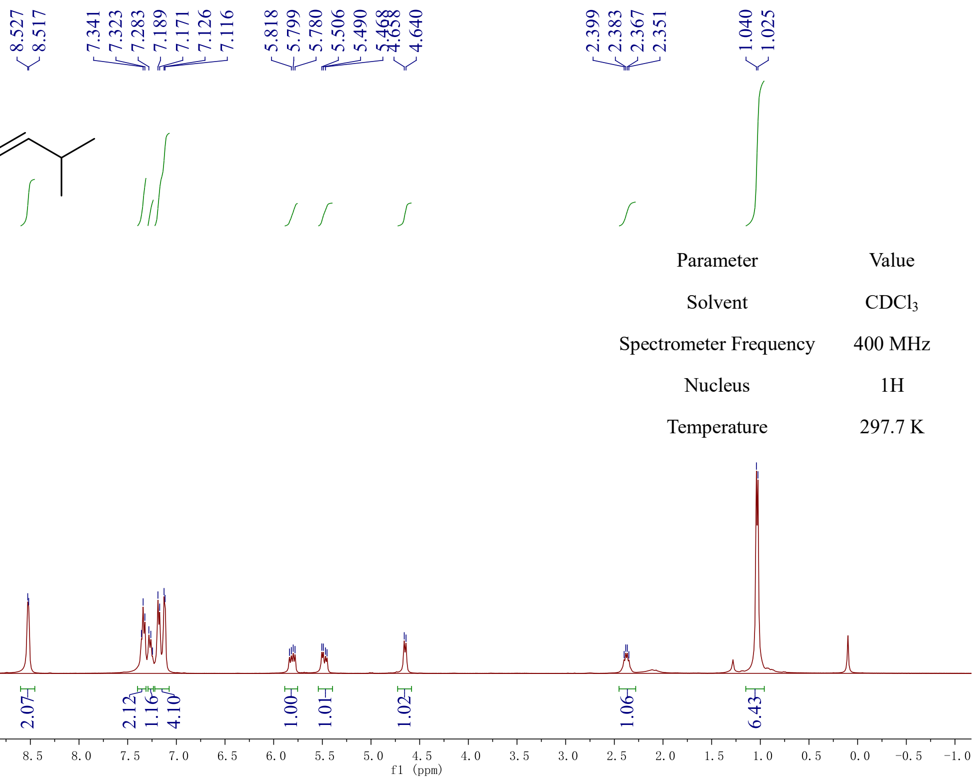

Parameter	Value
Solvent	$CDCl_3$
Spectrometer Frequency	400 MHz
Nucleus	1H
Temperature	297.7 K

^{1}H NMR Spectrum of Compound **3aa**

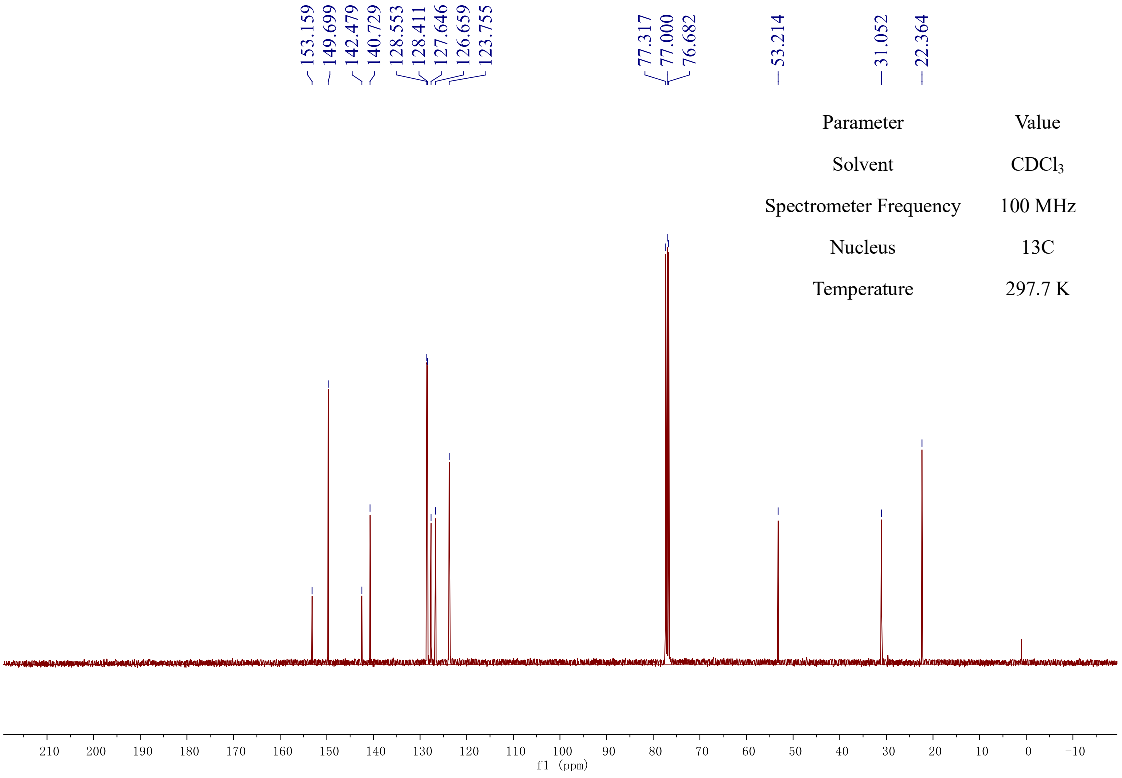

Parameter	Value
Solvent	$CDCl_3$
Spectrometer Frequency	100 MHz
Nucleus	13C
Temperature	297.7 K

^{13}C NMR Spectrum of Compound **3aa**

Supplementary Figure 112**. NMR spectra of 3aa**

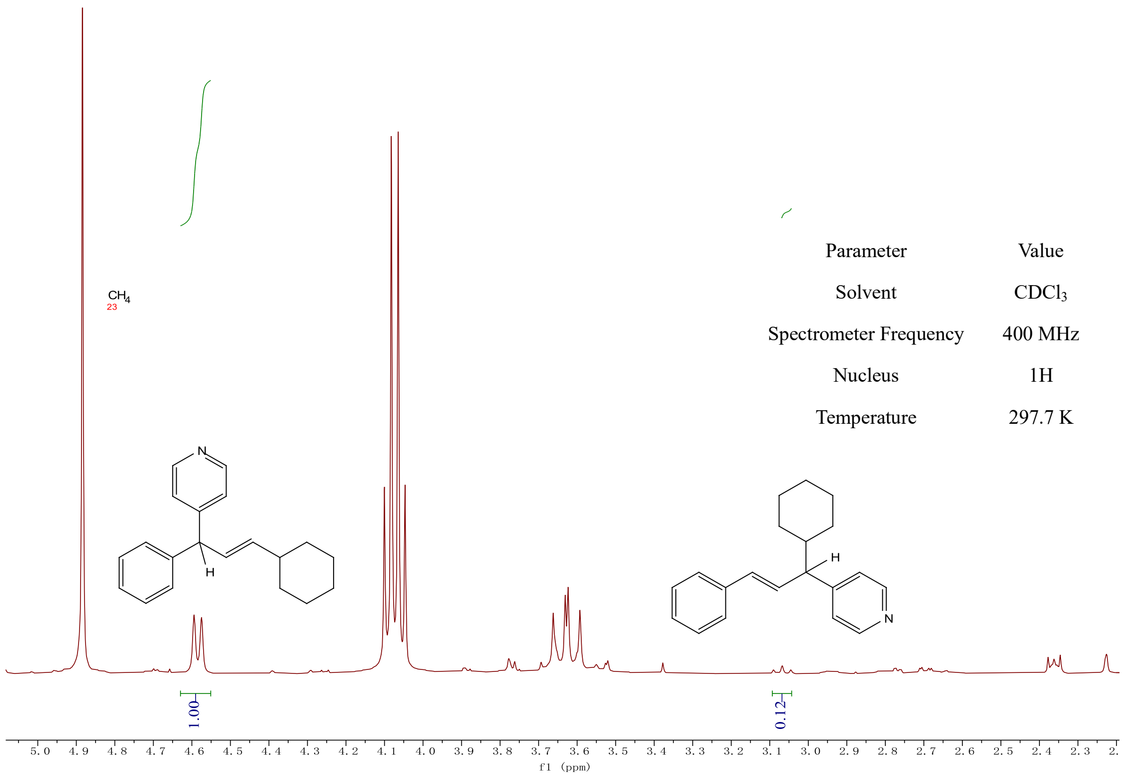

^{1}H NMR Spectrum of Crude Product **3ab**

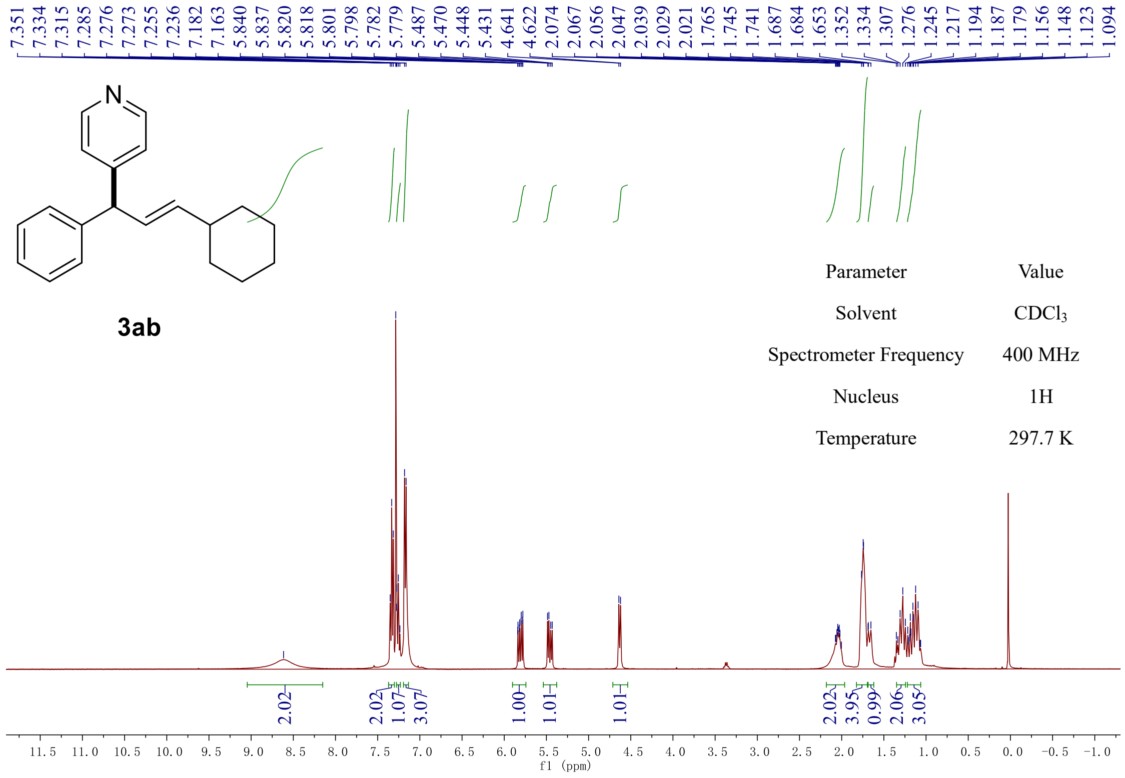

^{1}H NMR Spectrum of Compound **3ab**

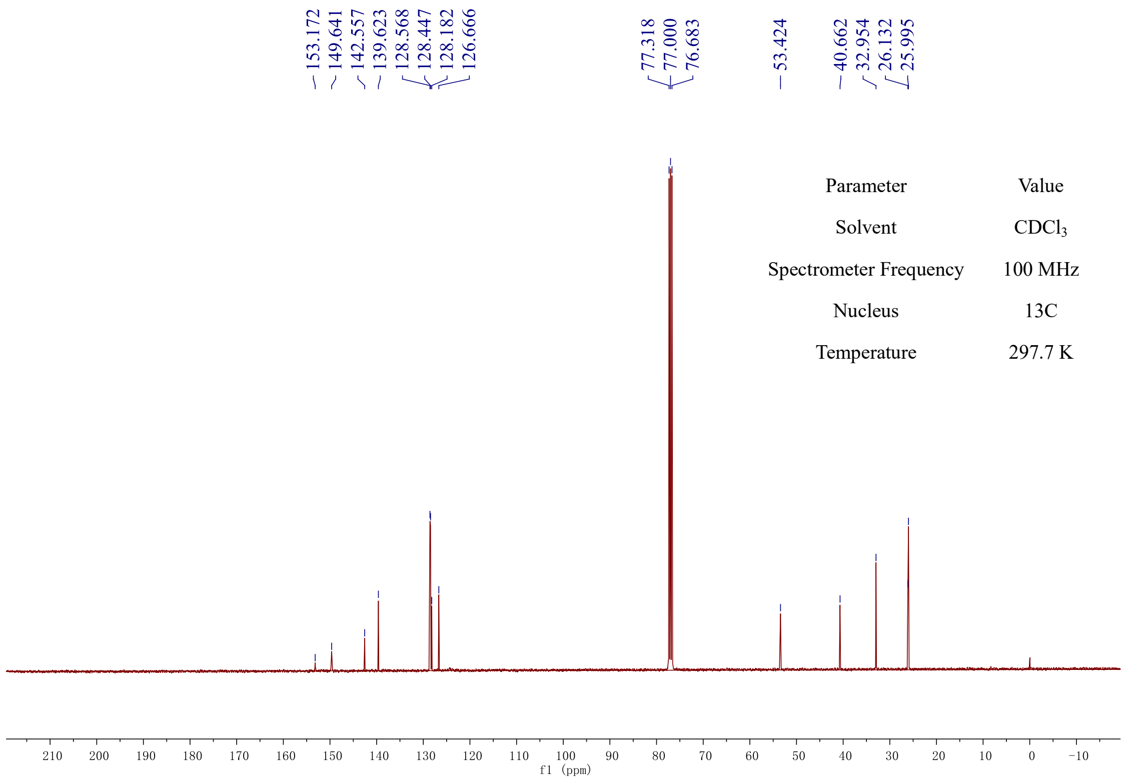

^{13}C NMR Spectrum of Compound **3ab**

Supplementary Figure 113**. NMR spectra of 3ab**

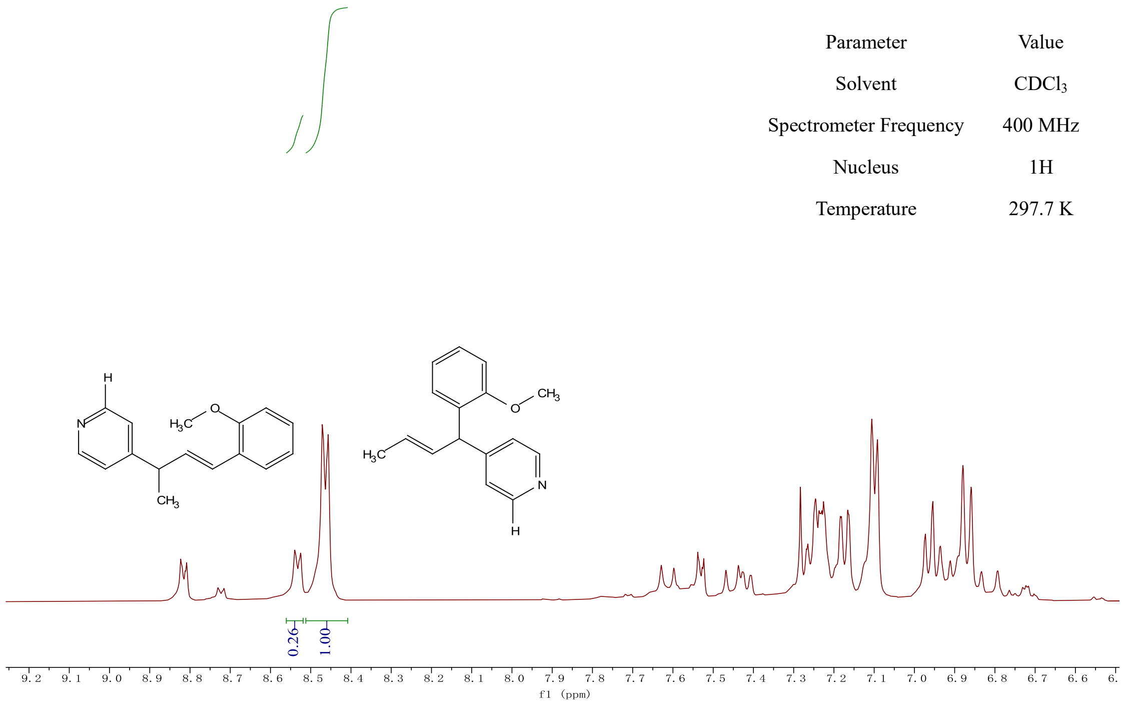

^{1}H NMR Spectrum of Crude Product **3ac**

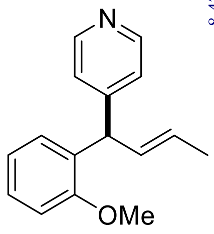

3ac

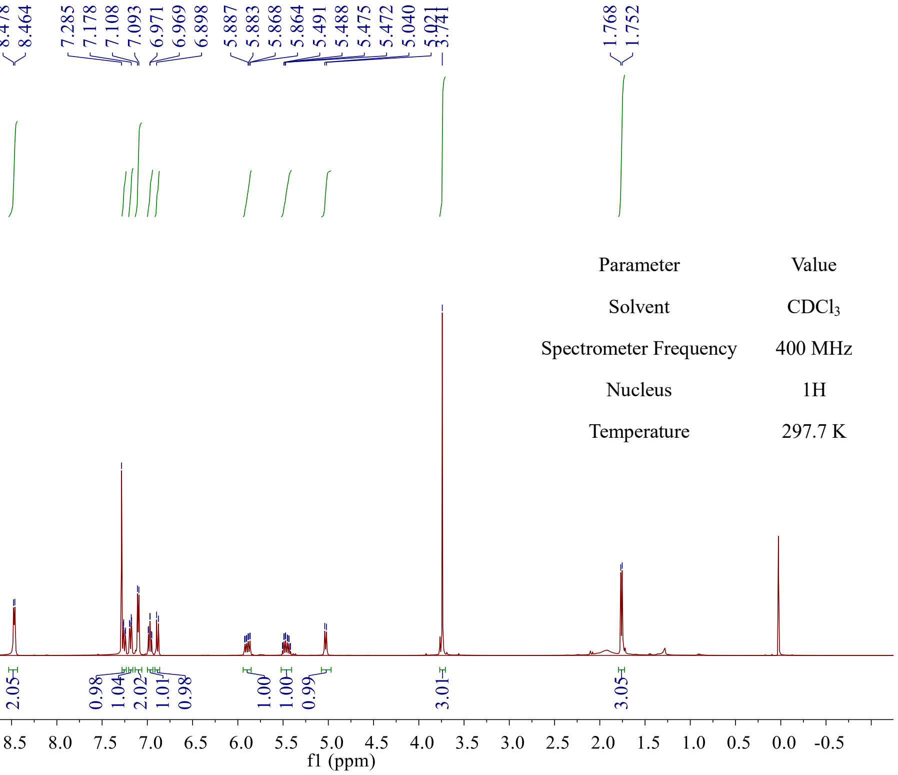

^{1}H NMR Spectrum of Compound **3ac**

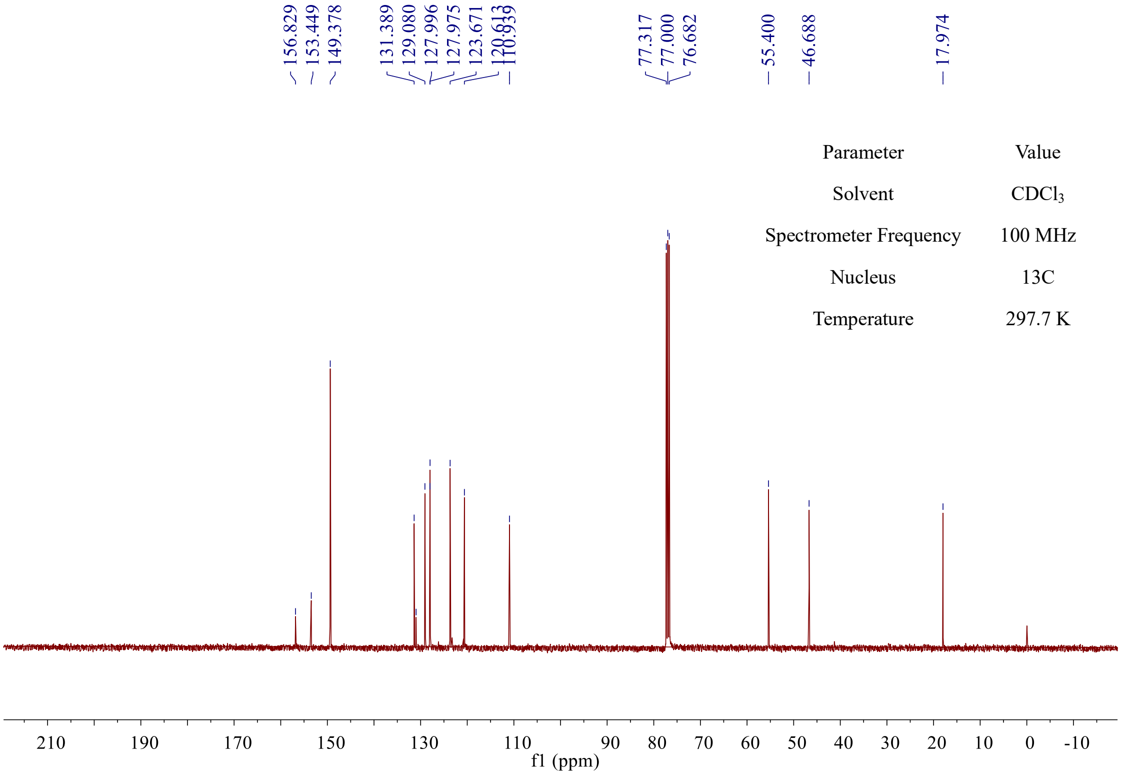

^{13}C NMR Spectrum of Compound **3ac**

Supplementary Figure 114**. NMR spectra of 3ac**

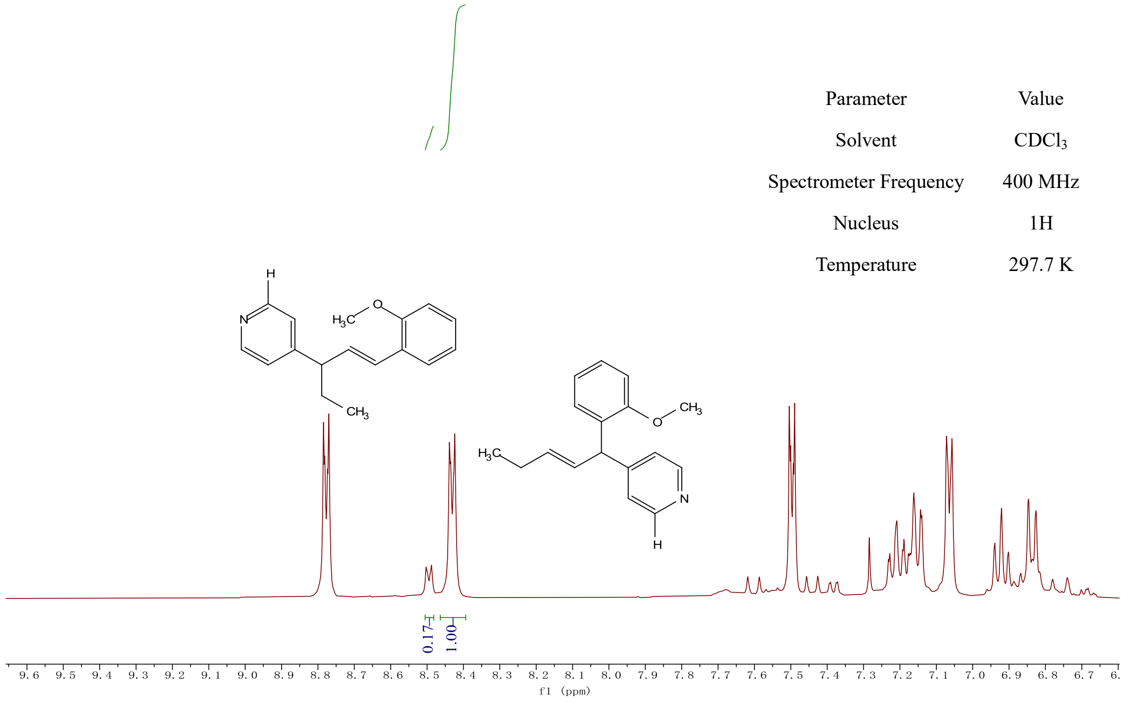

Parameter	Value
Solvent	$CDCl_3$
Spectrometer Frequency	400 MHz
Nucleus	1H
Temperature	297.7 K

^{1}H NMR Spectrum of Crude Product **3ad**

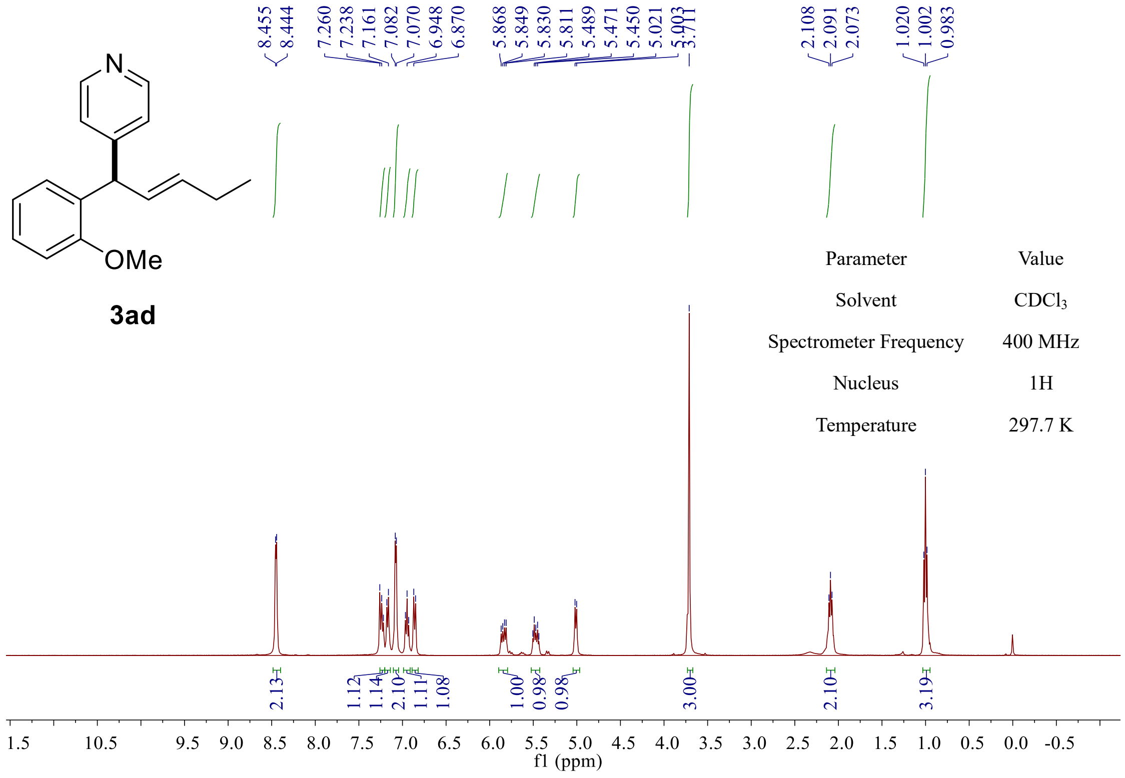

3ad

Parameter	Value
Solvent	$CDCl_3$
Spectrometer Frequency	400 MHz
Nucleus	1H
Temperature	297.7 K

. ^{1}H NMR Spectrum of Compound **3ad**

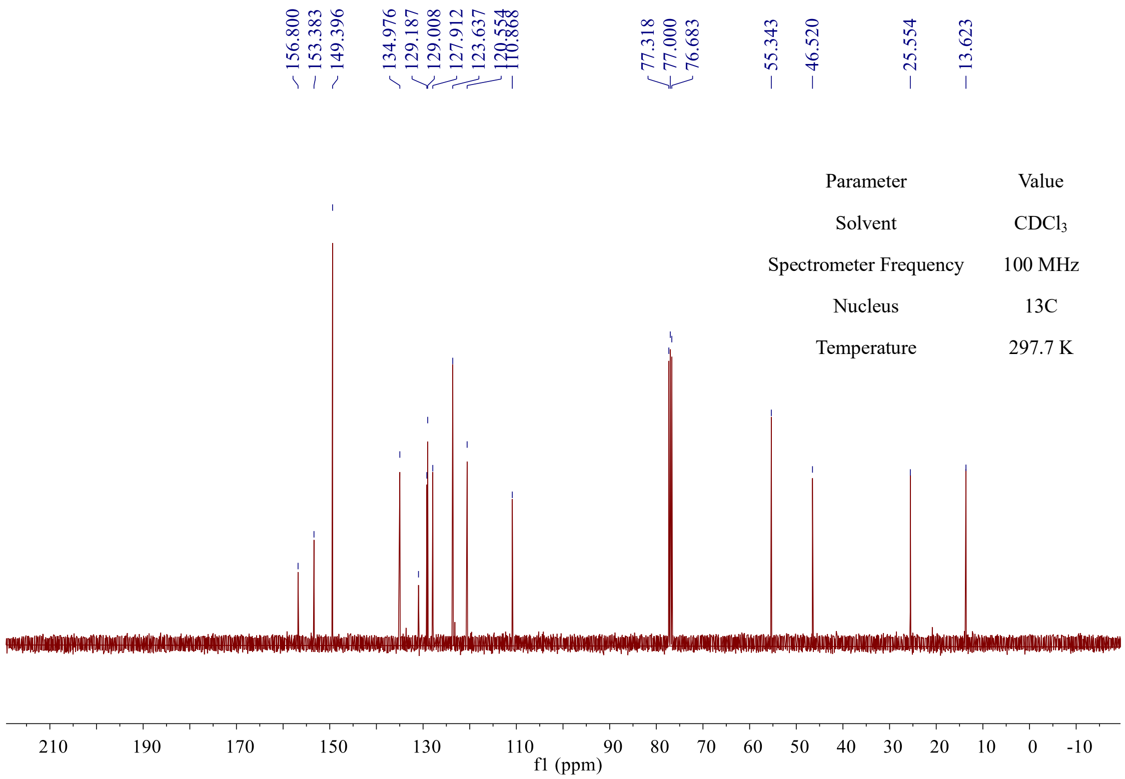

^{13}C NMR Spectrum of Compound **3ad**

Supplementary Figure 115**. NMR spectra of 3ad**

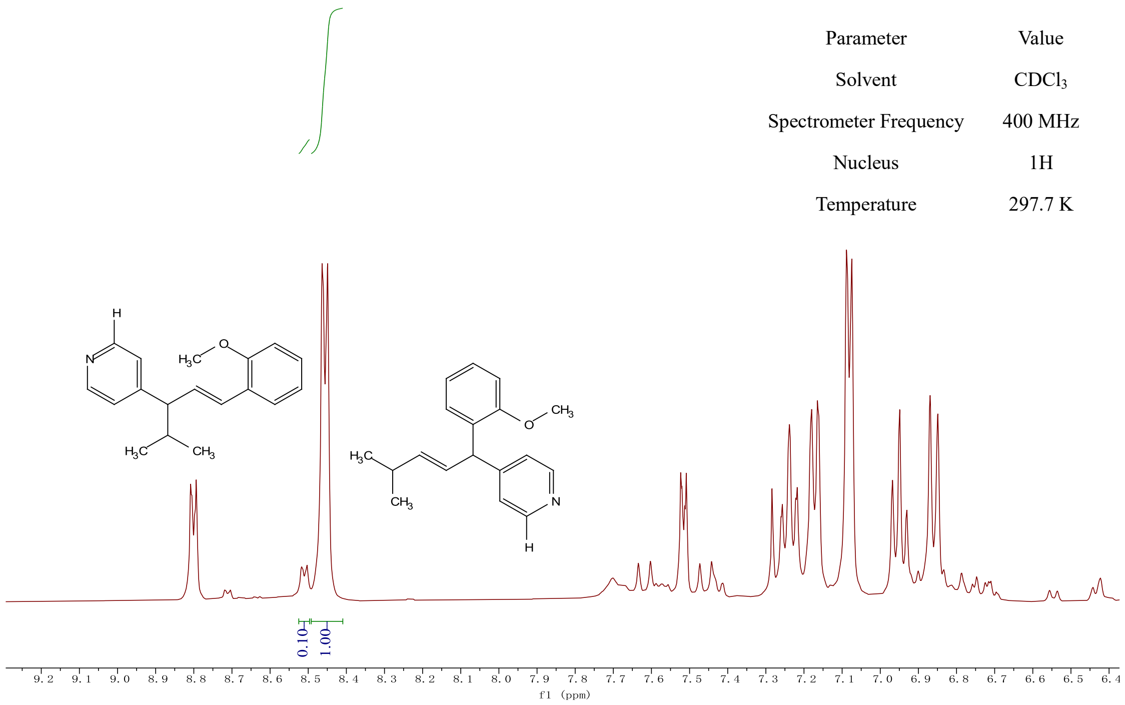

^{1}H NMR Spectrum of Crude Product **3ae**

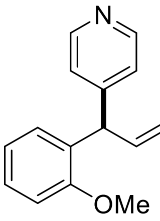

3ae

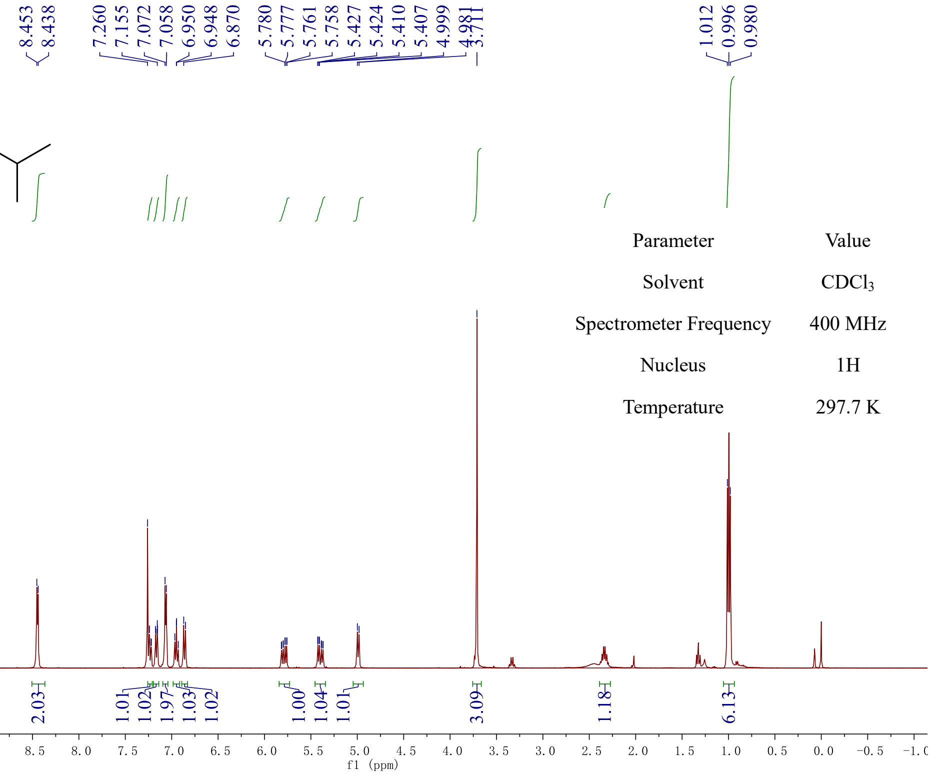

Parameter	Value
Solvent	$CDCl_3$
Spectrometer Frequency	400 MHz
Nucleus	1H
Temperature	297.7 K

^{1}H NMR Spectrum of Compound **3ae**

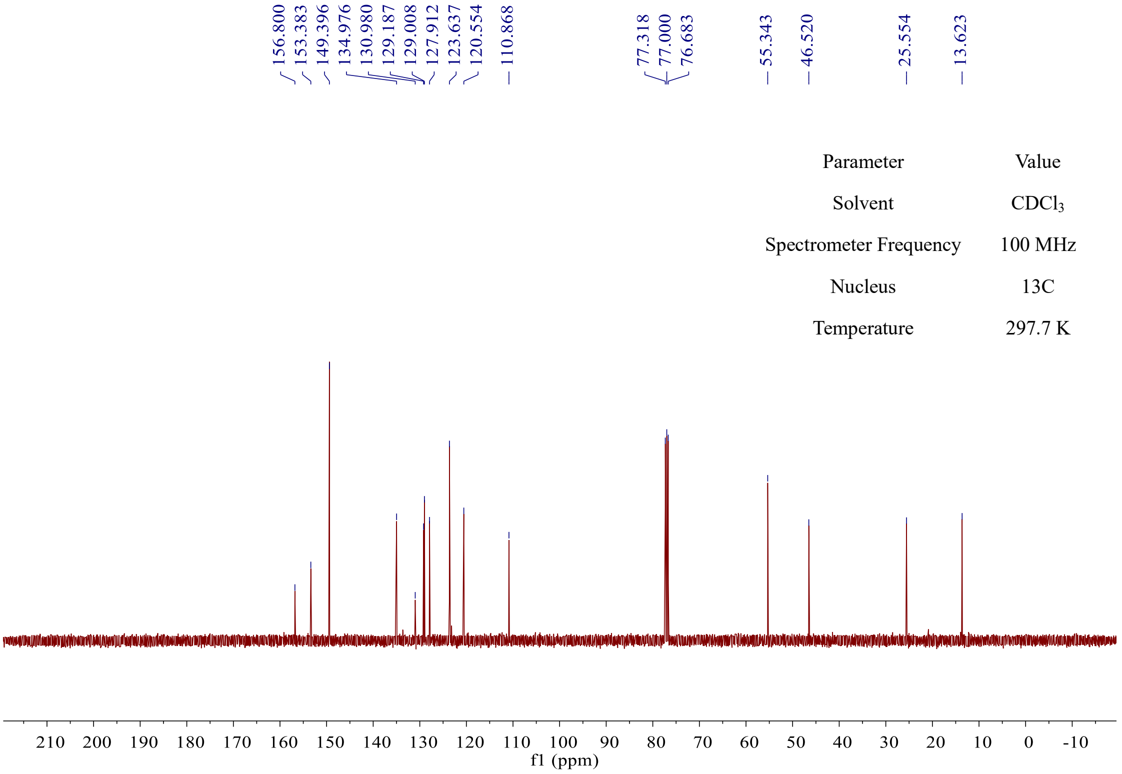

Parameter	Value
Solvent	$CDCl_3$
Spectrometer Frequency	100 MHz
Nucleus	13C
Temperature	297.7 K

^{13}C NMR Spectrum of Compound **3ae**

Supplementary Figure 116**. NMR spectra of 3ae**

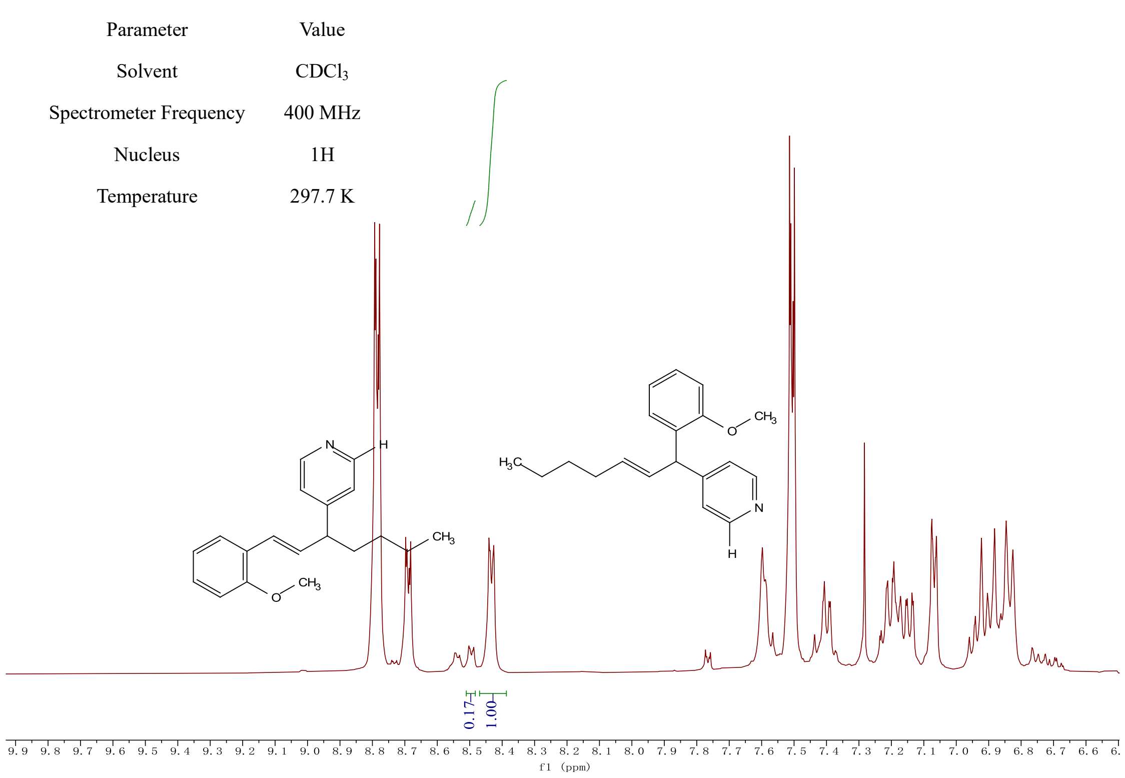

^{1}H NMR Spectrum of Crude Product **3af**

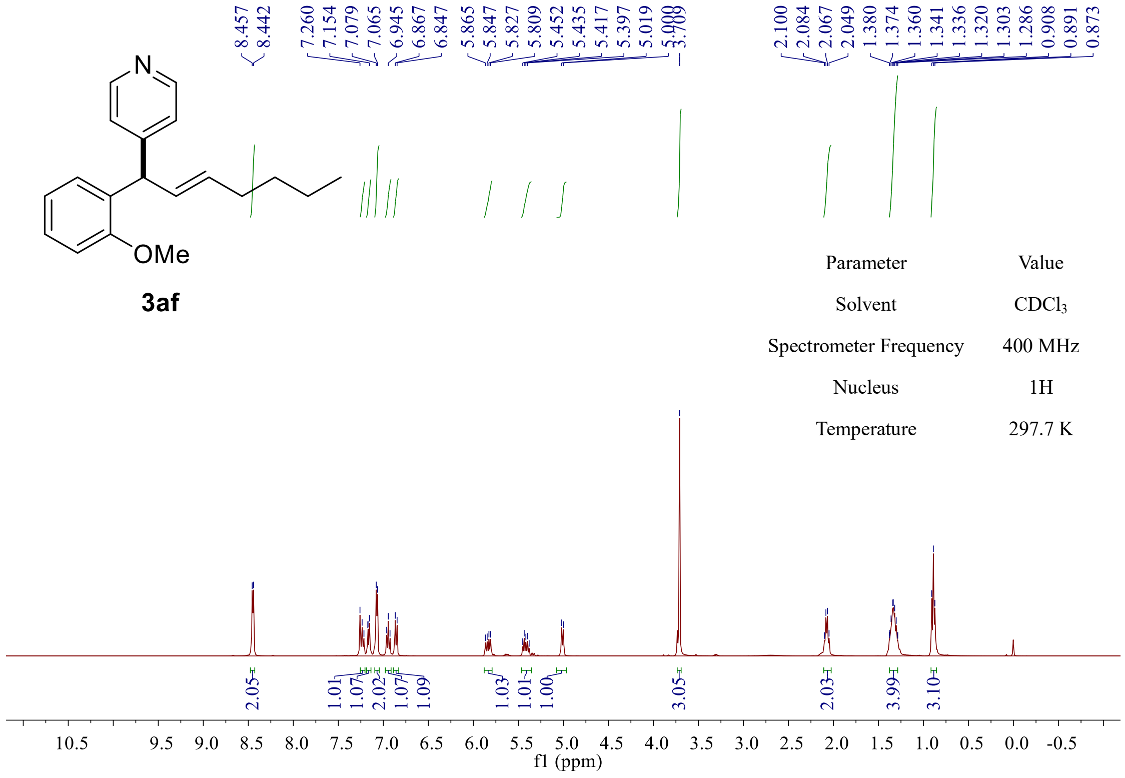

^{1}H NMR Spectrum of Compound **3af**

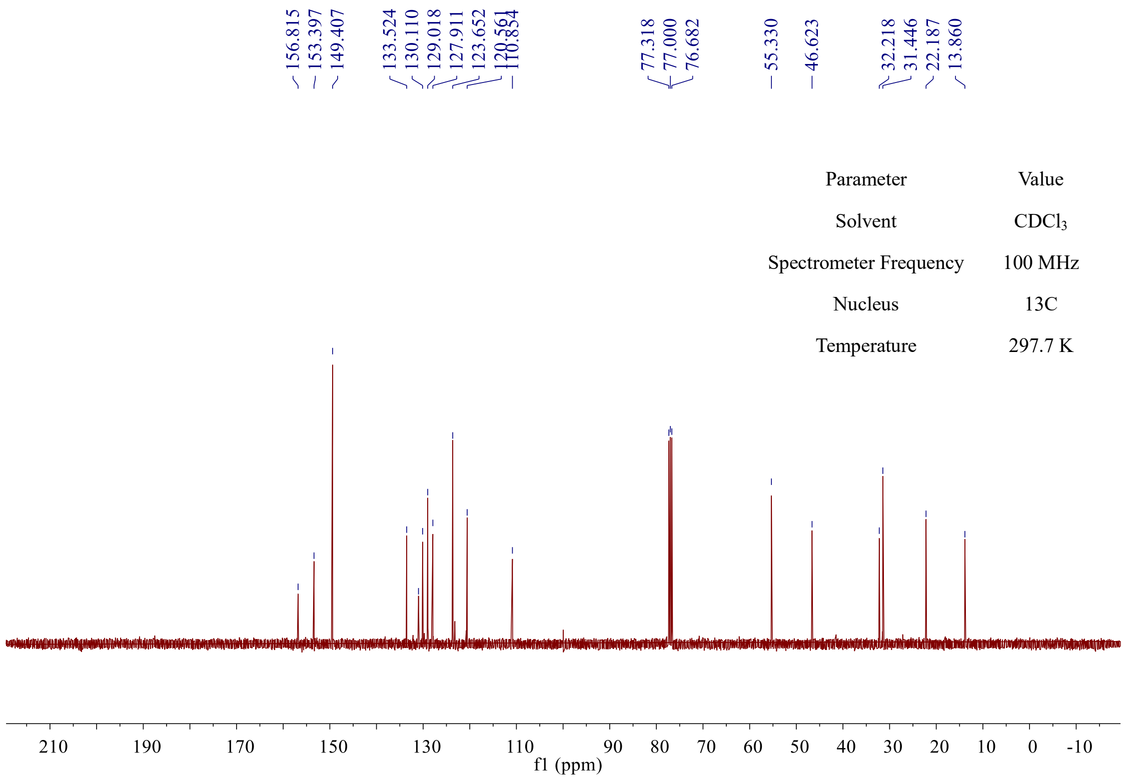

Parameter	Value
Solvent	$CDCl_3$
Spectrometer Frequency	100 MHz
Nucleus	13C
Temperature	297.7 K

^{13}C NMR Spectrum of Compound **3af**

Supplementary Figure 117. **NMR spectra of 3af**

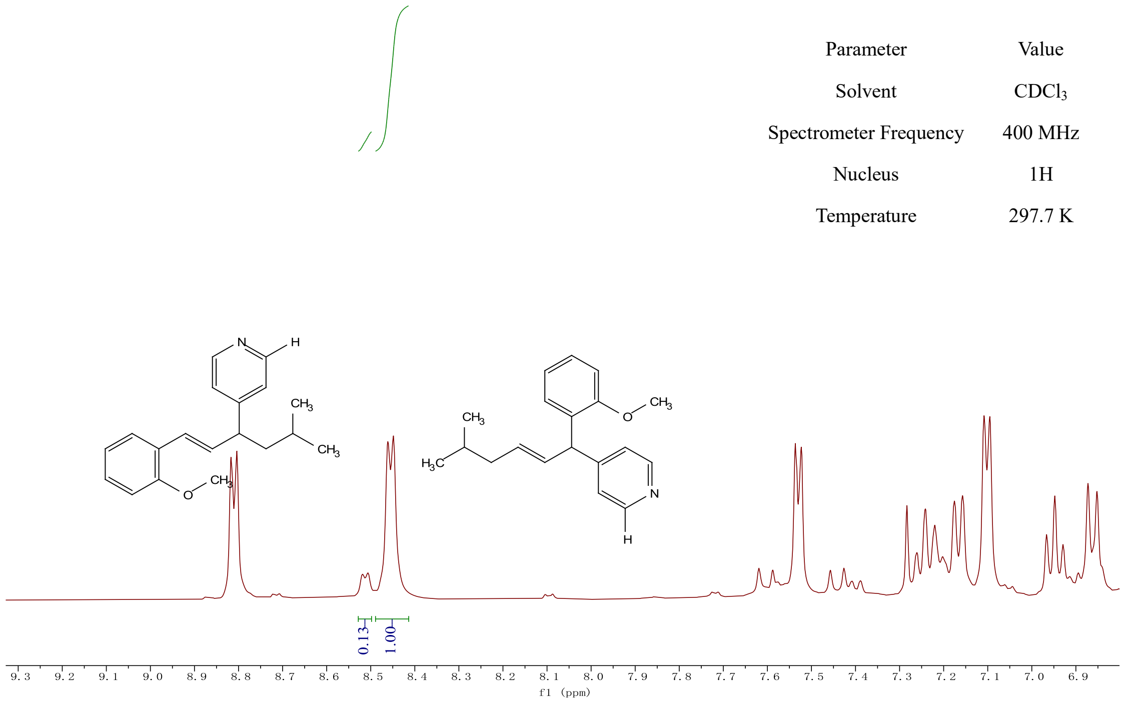

Parameter	Value
Solvent	$CDCl_3$
Spectrometer Frequency	400 MHz
Nucleus	1H
Temperature	297.7 K

^{1}H NMR Spectrum of Crude Product **3ag**

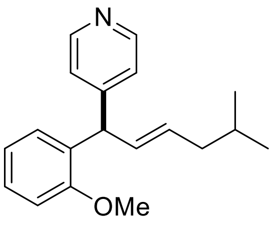

3ag

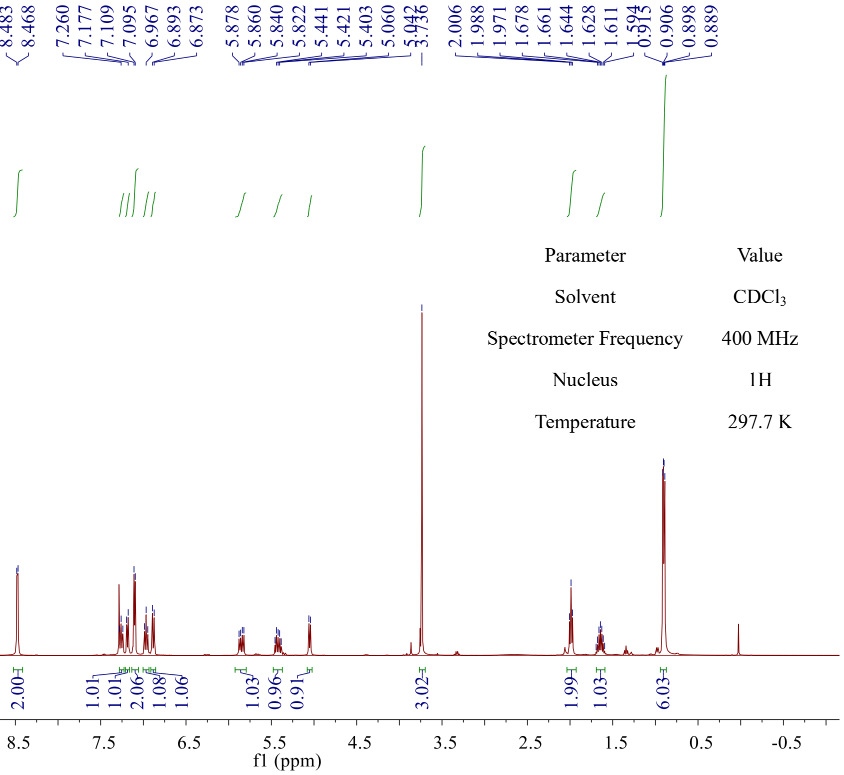

^{1}H NMR Spectrum of Compound **3ag**

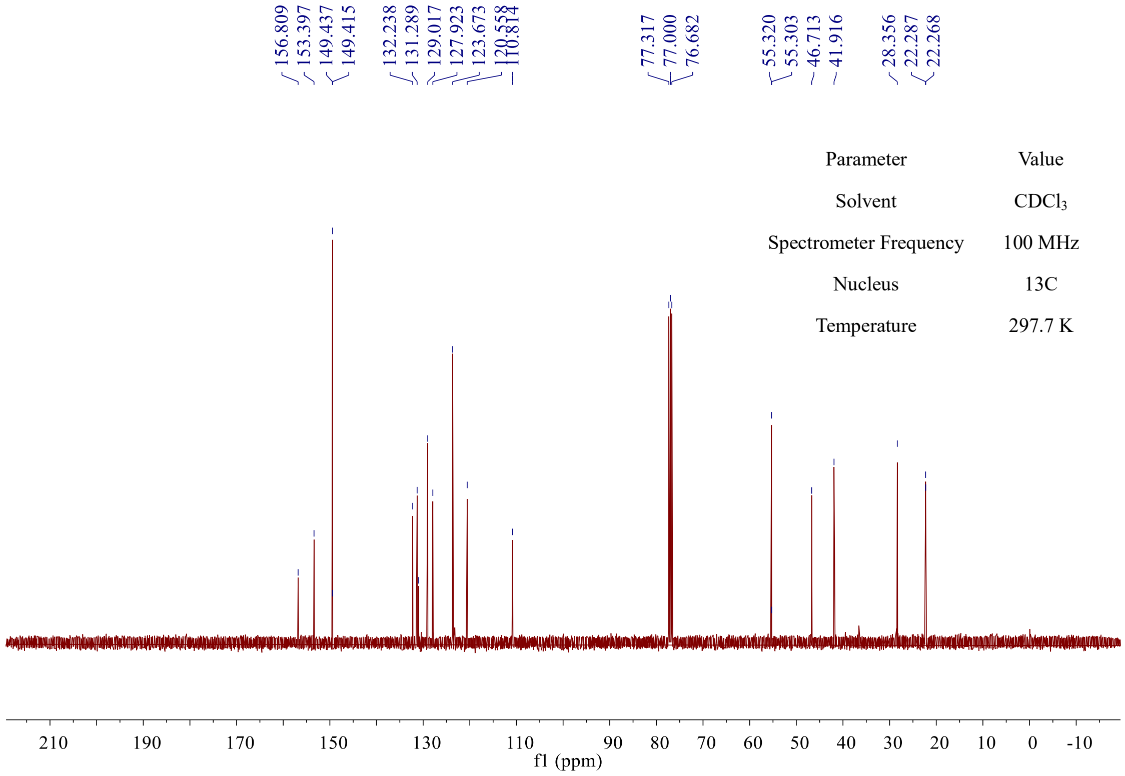

^{13}C NMR Spectrum of Compound **3ag**

Supplementary Figure 118**. NMR spectra of 3ag**

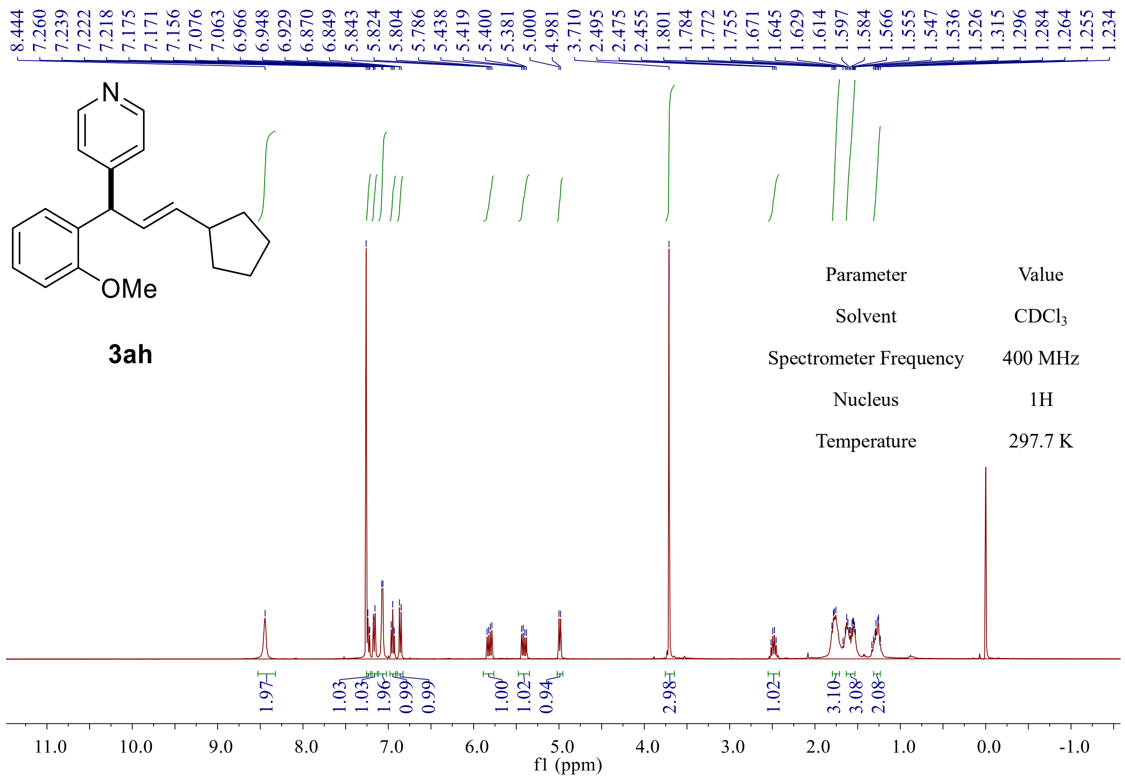

^{1}H NMR Spectrum of Compound **3ah**

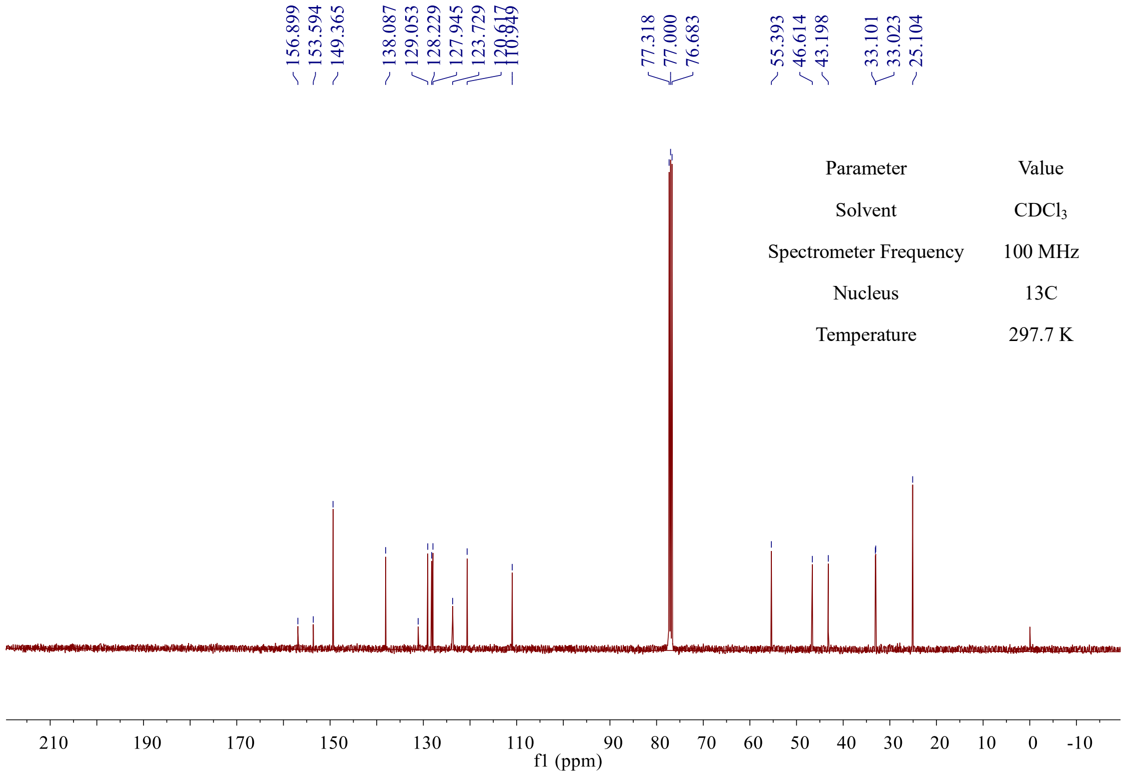

^{13}C NMR Spectrum of Compound **3ah**

Supplementary Figure 119**. NMR spectra of 3ah**

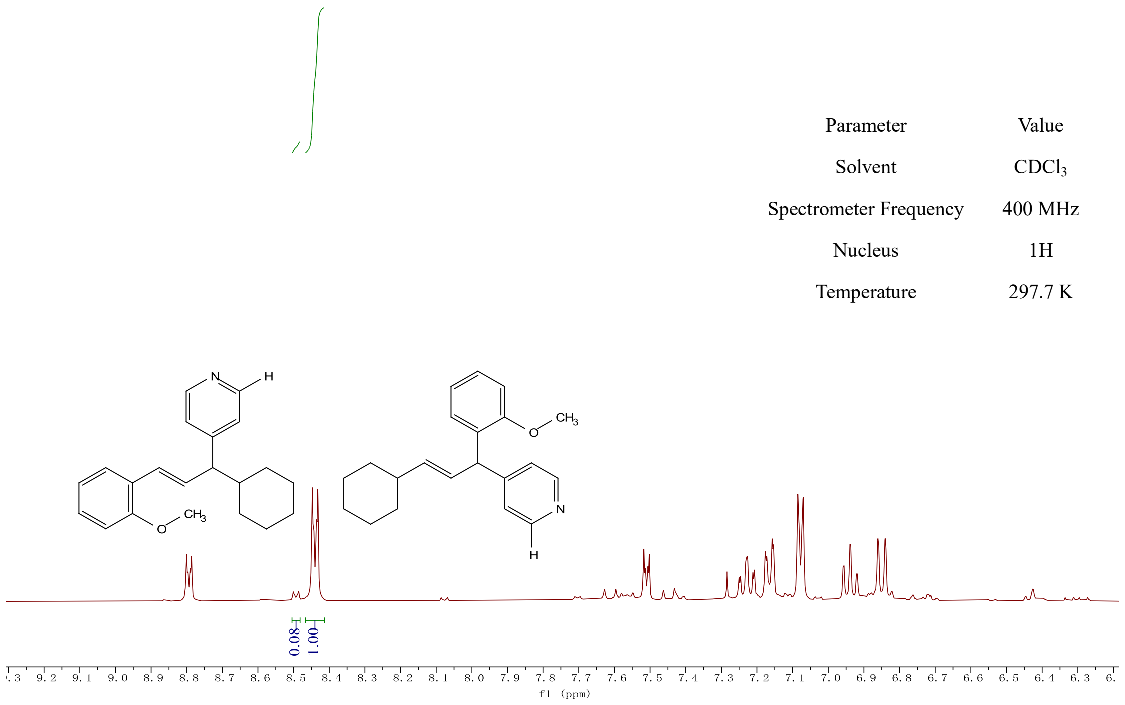

^{1}H NMR Spectrum of Crude Product **3ai**

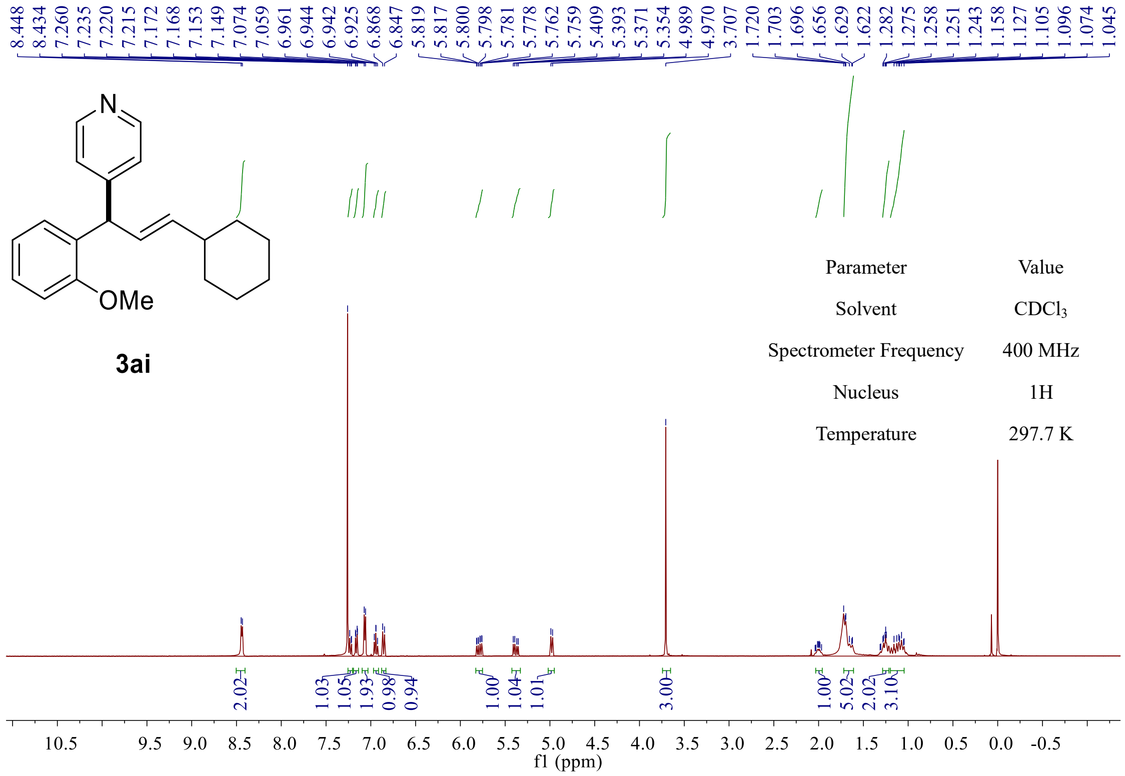

^{1}H NMR Spectrum of Compound **3ai**

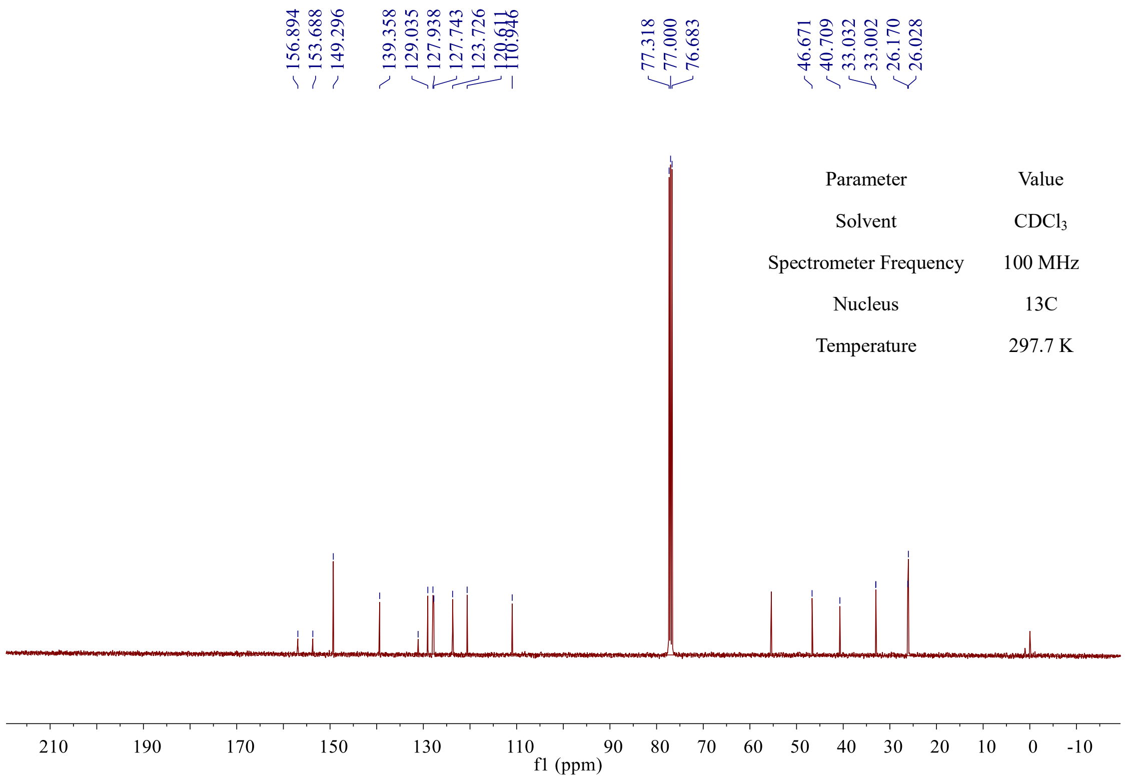

^{13}C NMR Spectrum of Compound **3ai**

Supplementary Figure 120**. NMR spectra of 3ai**

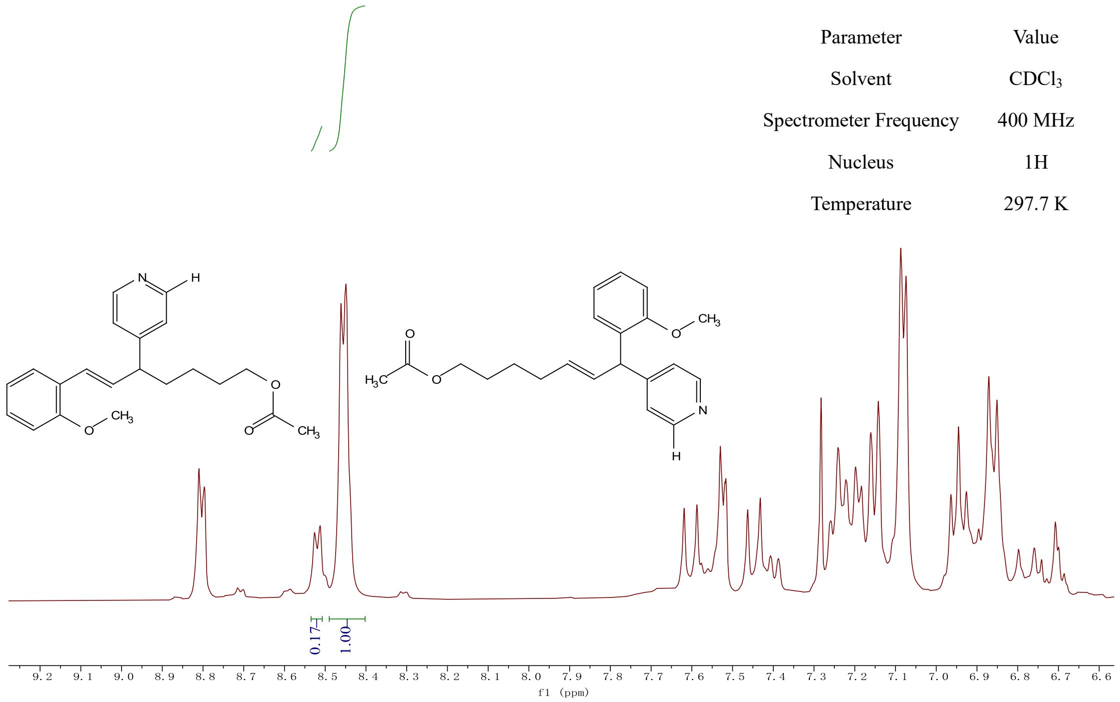

^{1}H NMR Spectrum of Crude Product **3aj**

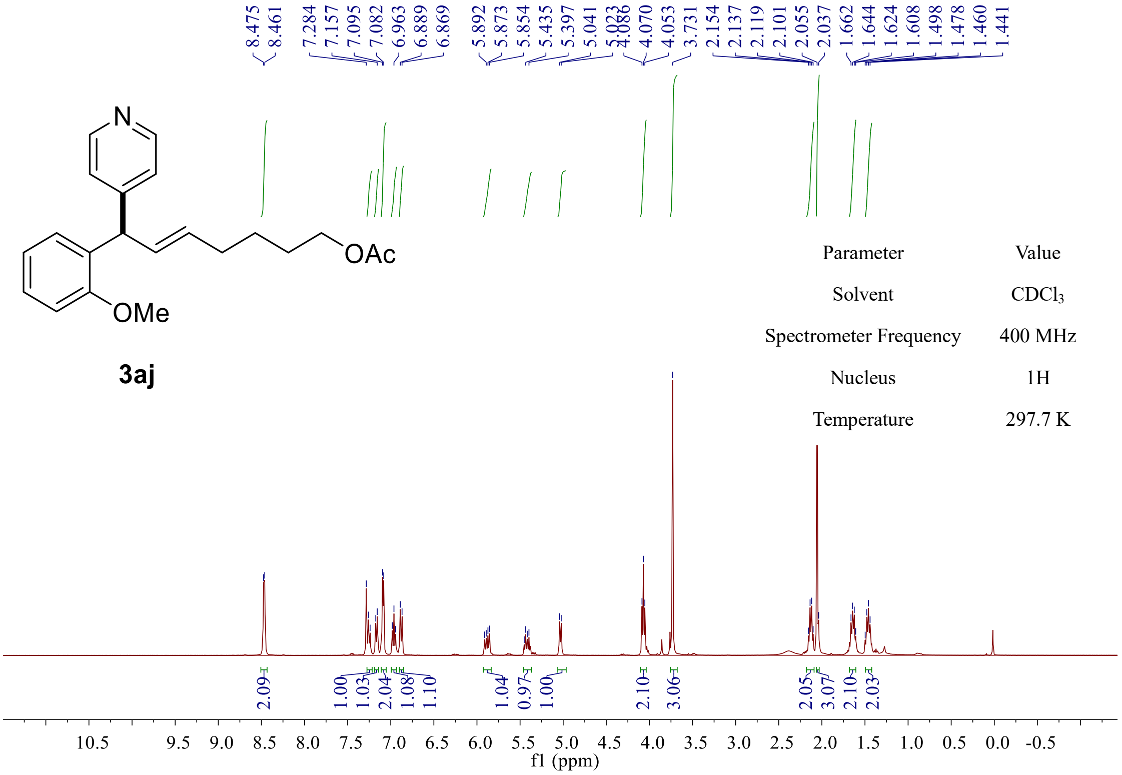

3aj

^{1}H NMR Spectrum of Compound **3aj**

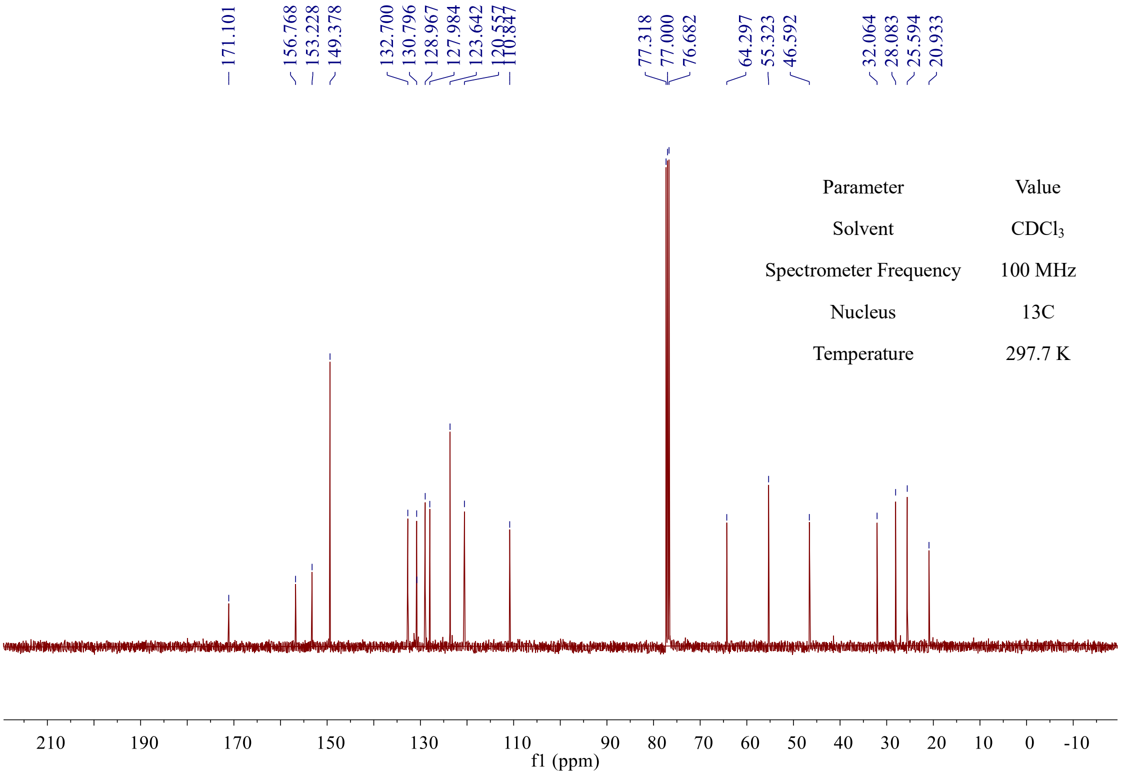

^{13}C NMR Spectrum of Compound **3aj**

Supplementary Figure 121**. NMR spectra of 3aj**

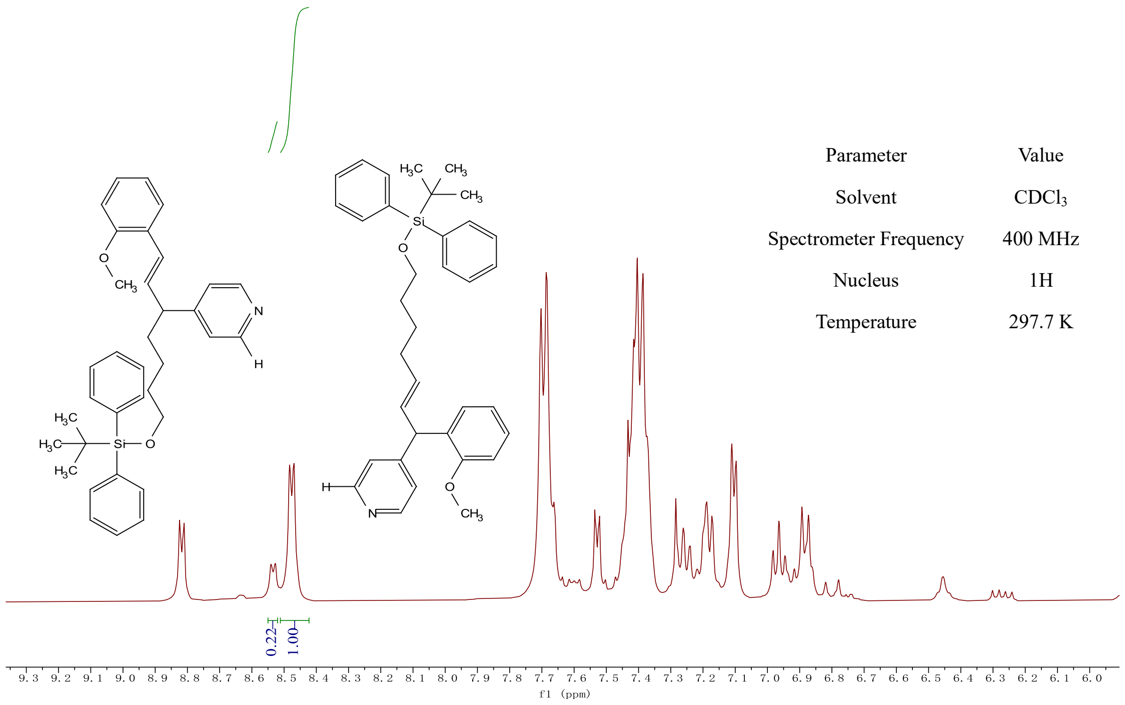

Parameter	Value
Solvent	$CDCl_3$
Spectrometer Frequency	400 MHz
Nucleus	1H
Temperature	297.7 K

^{1}H NMR Spectrum of Crude Product **3ak**

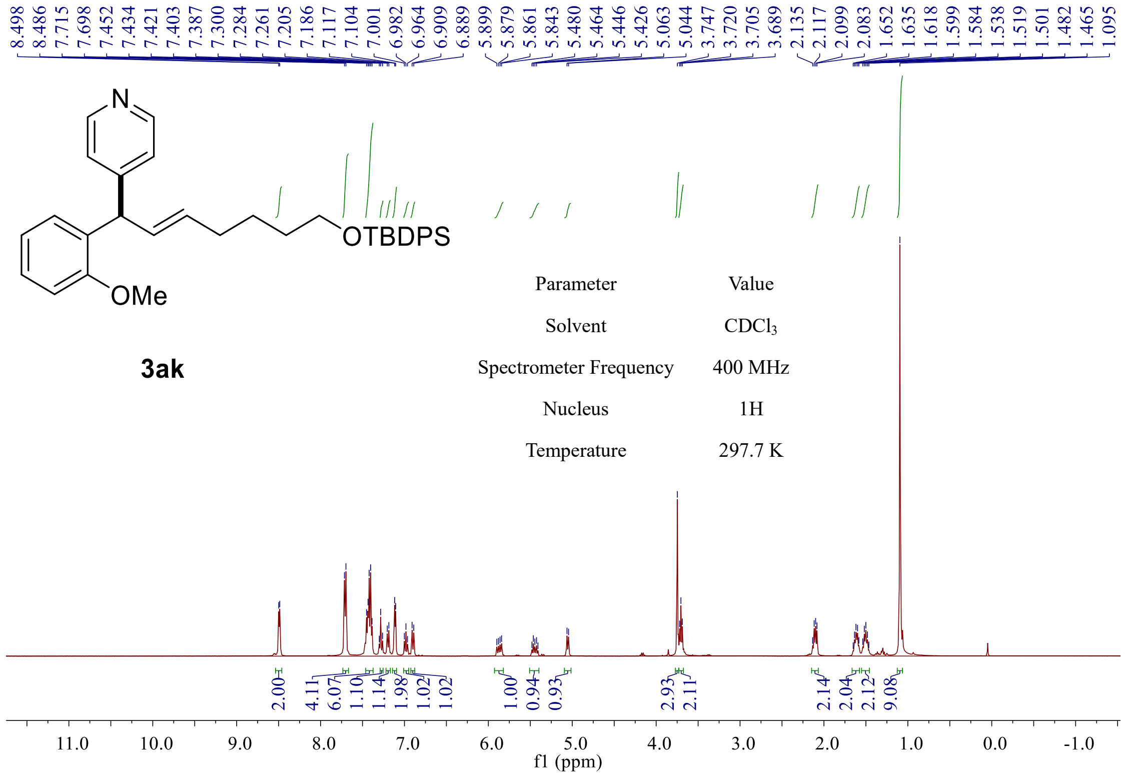

Parameter	Value
Solvent	$CDCl_3$
Spectrometer Frequency	400 MHz
Nucleus	1H
Temperature	297.7 K

^{1}H NMR Spectrum of Compound **3ak**

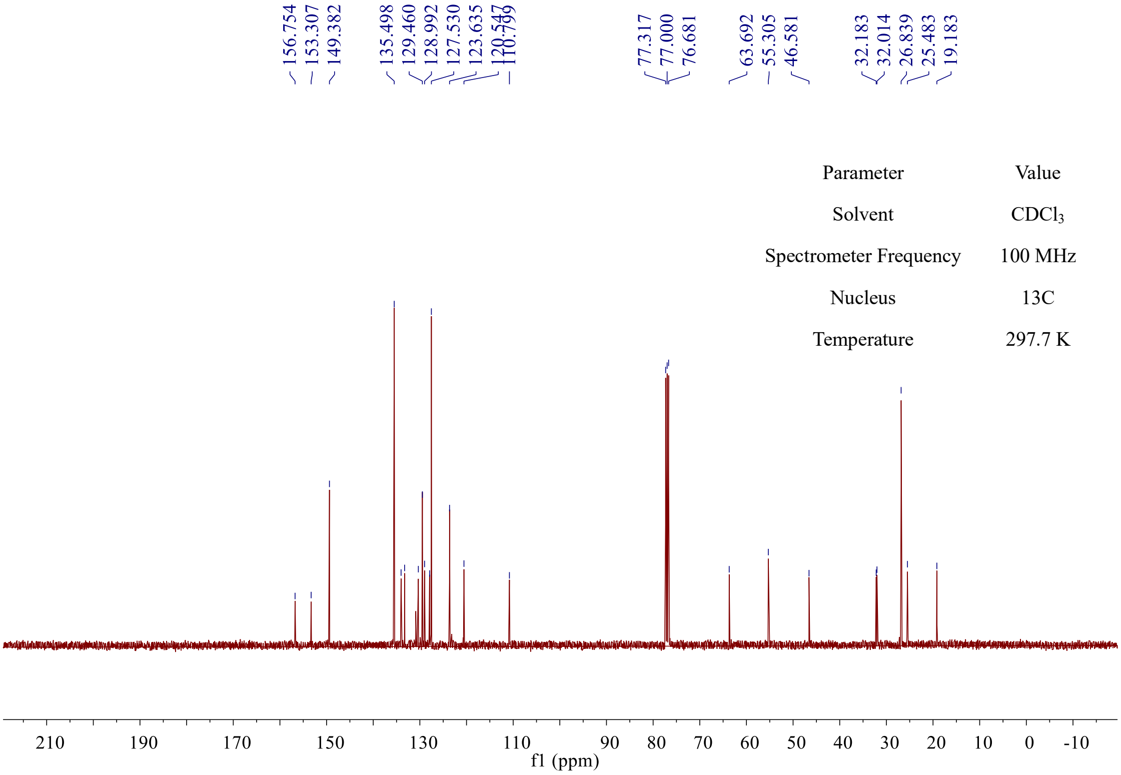

^{13}C NMR Spectrum of Compound **3ak**

Supplementary Figure 122**. NMR spectra of 3ak**

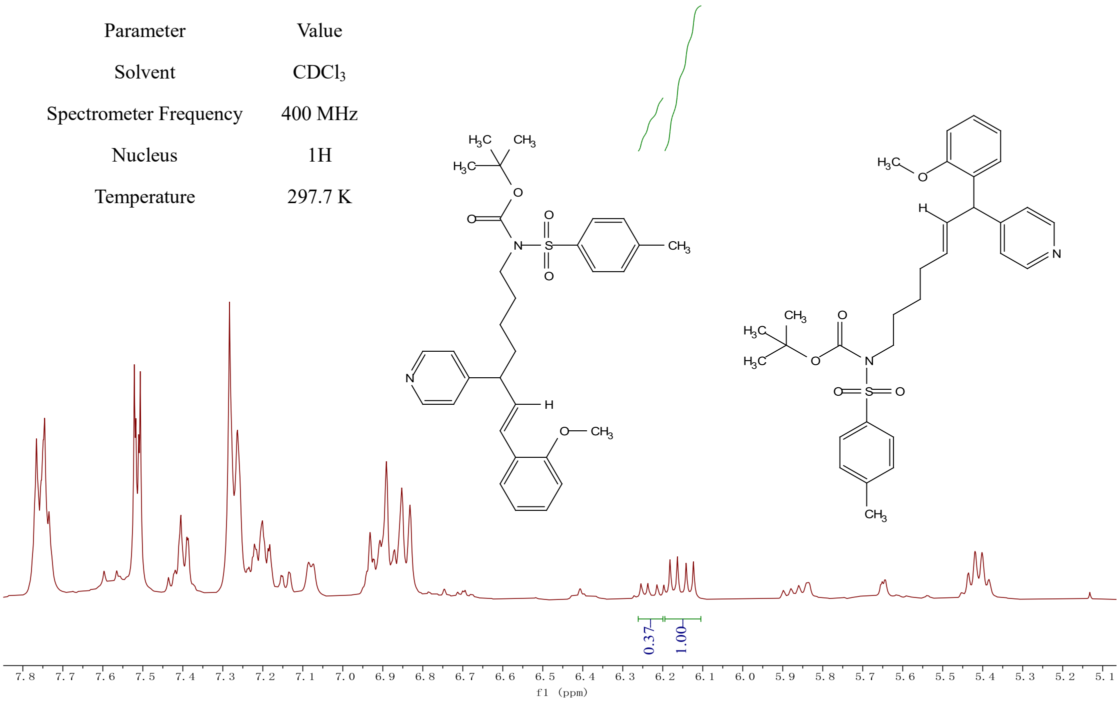

^{1}H NMR Spectrum of Crude Product **3al**

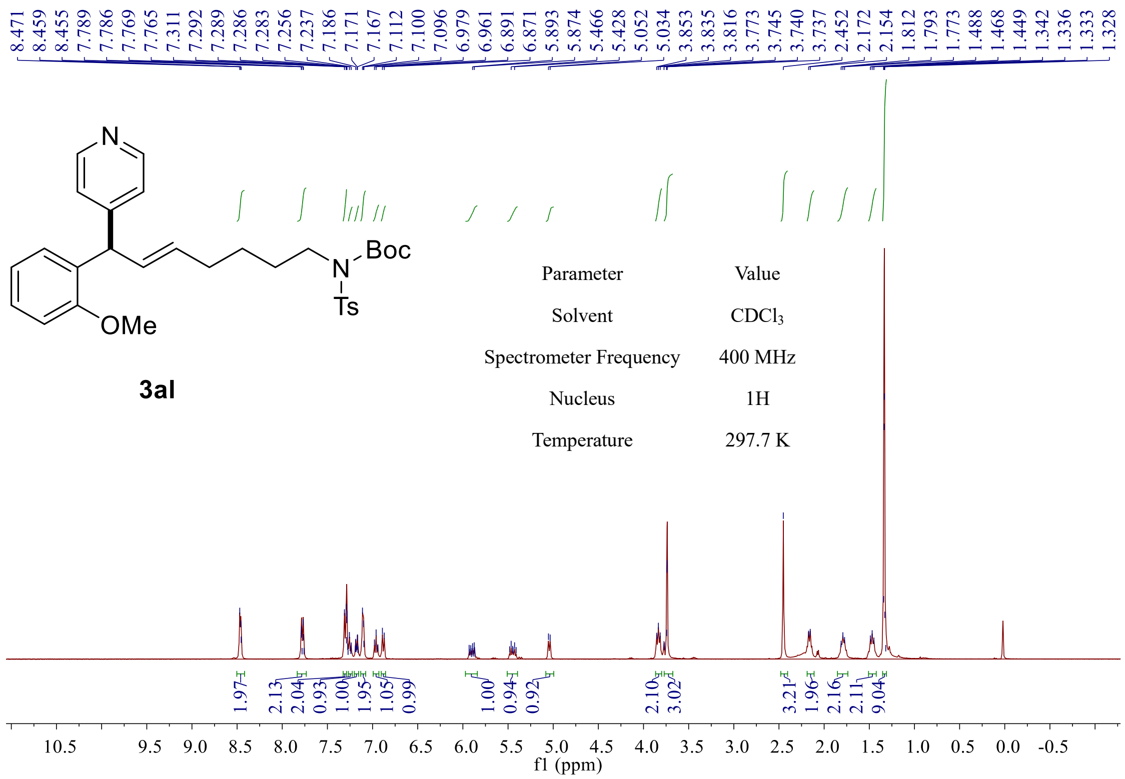

^{1}H NMR Spectrum of Compound **3al**

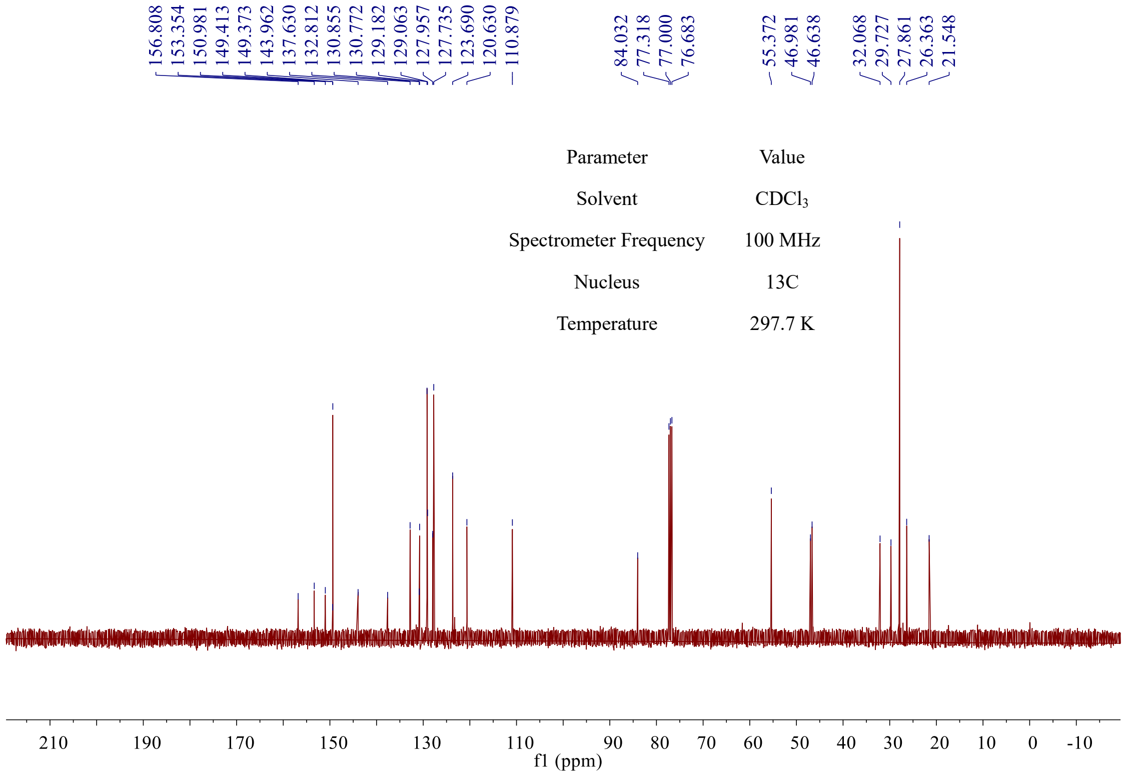

^{13}C NMR Spectrum of Compound **3al**

Supplementary Figure 123**. NMR spectra of 3al**

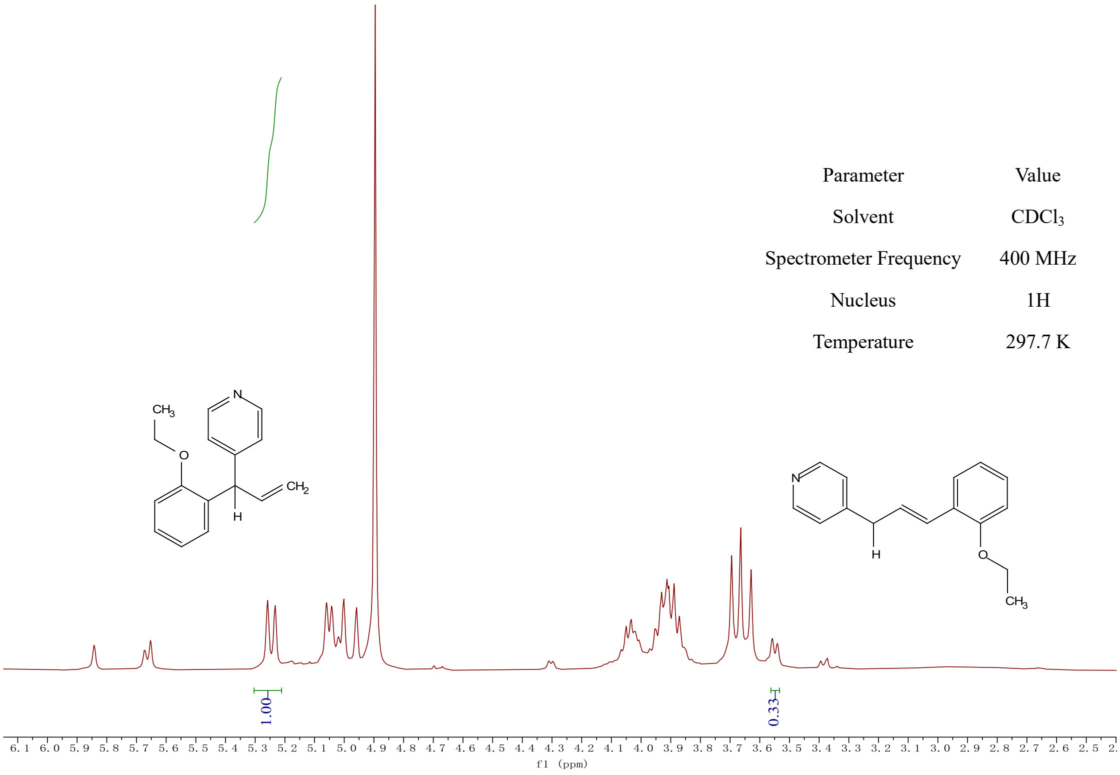

^{1}H NMR Spectrum of Crude Product **3am**

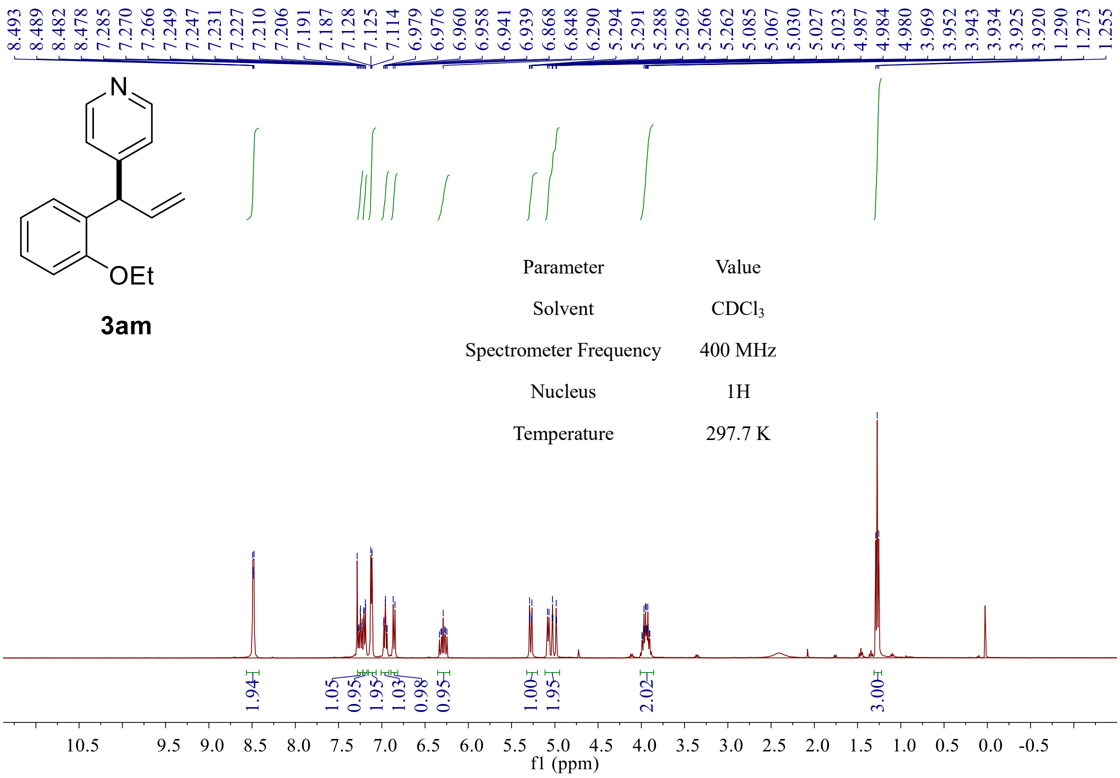

^{1}H NMR Spectrum of Compound **3am**

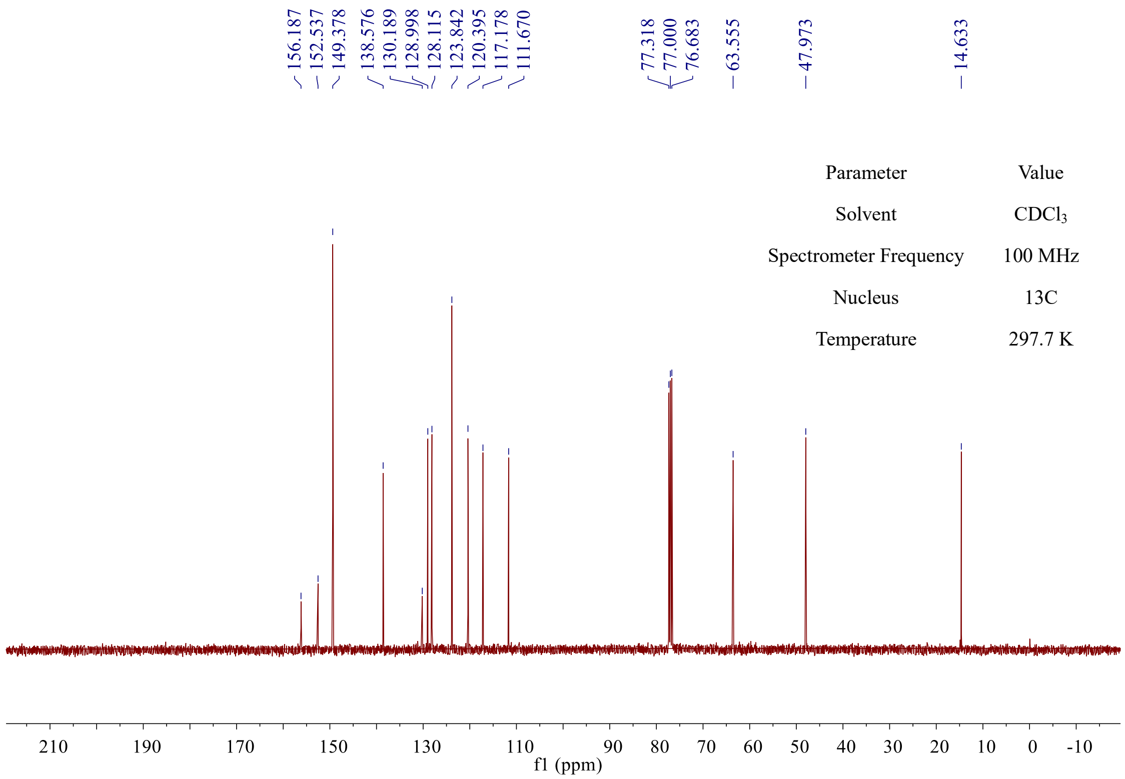

^{13}C NMR Spectrum of Compound **3am**

Supplementary Figure 124**. NMR spectra of 3am**

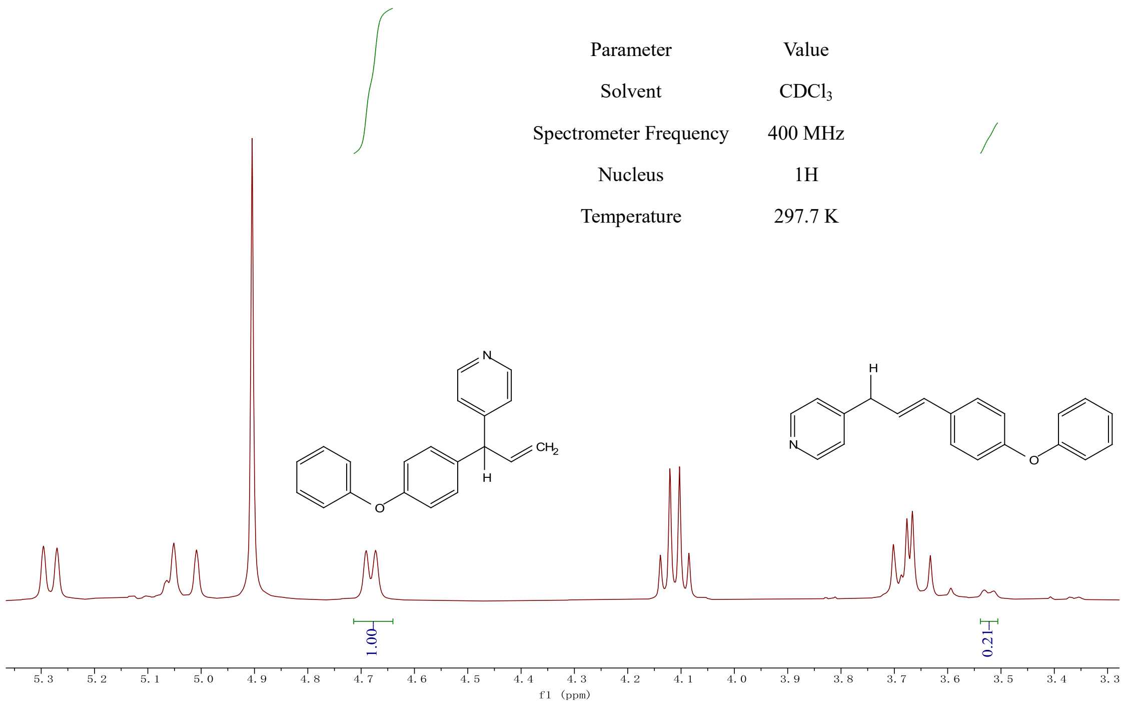

^{1}H NMR Spectrum of Crude Product **3an**

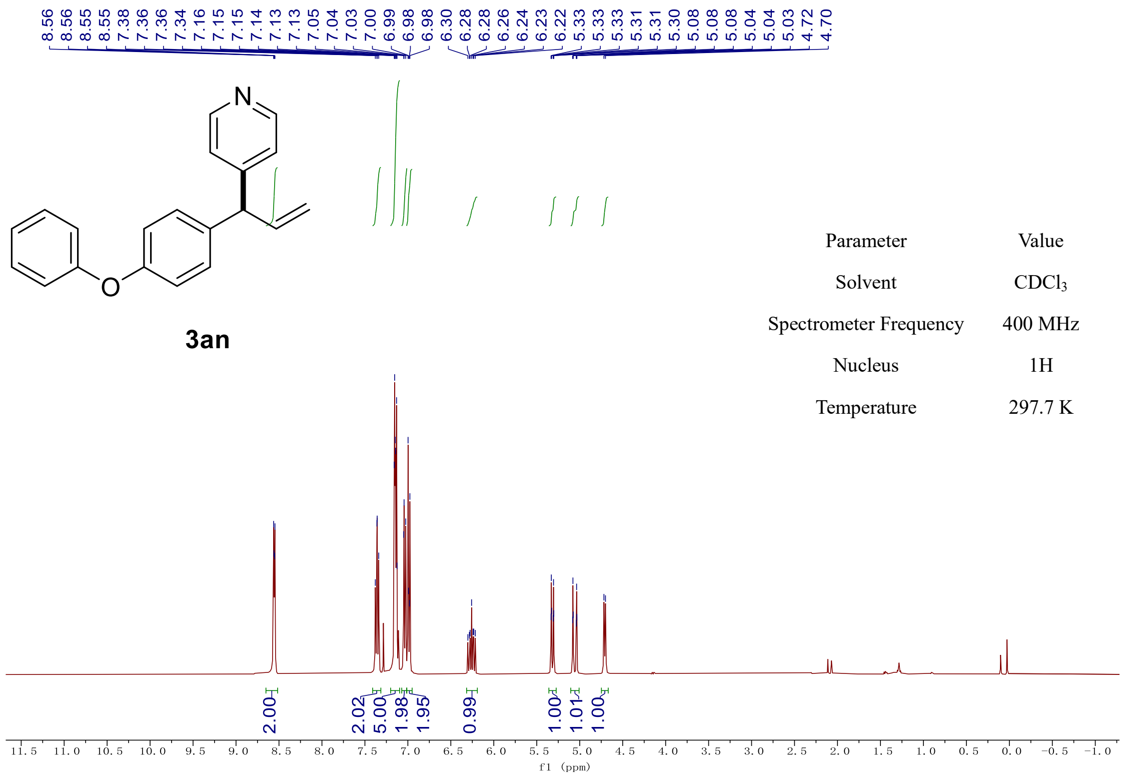

^{1}H NMR Spectrum of Compound **3an**

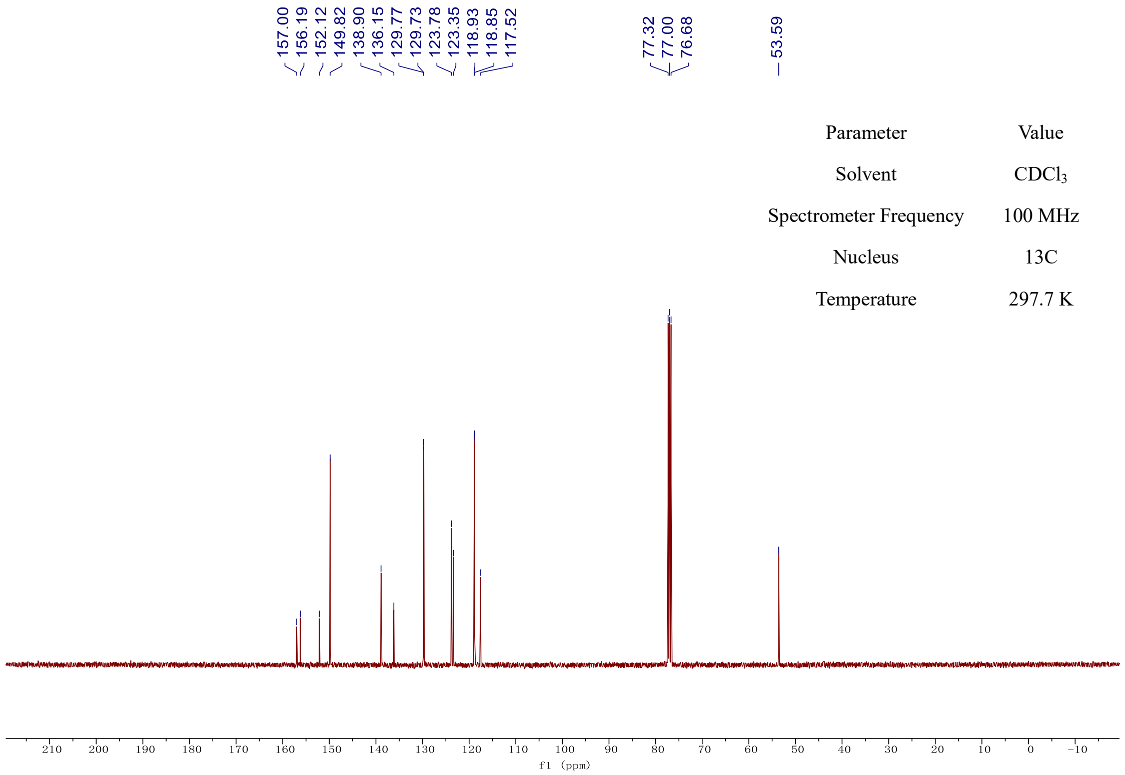

^{13}C NMR Spectrum of Compound **3an**

Supplementary Figure 125**. NMR spectra of 3an**

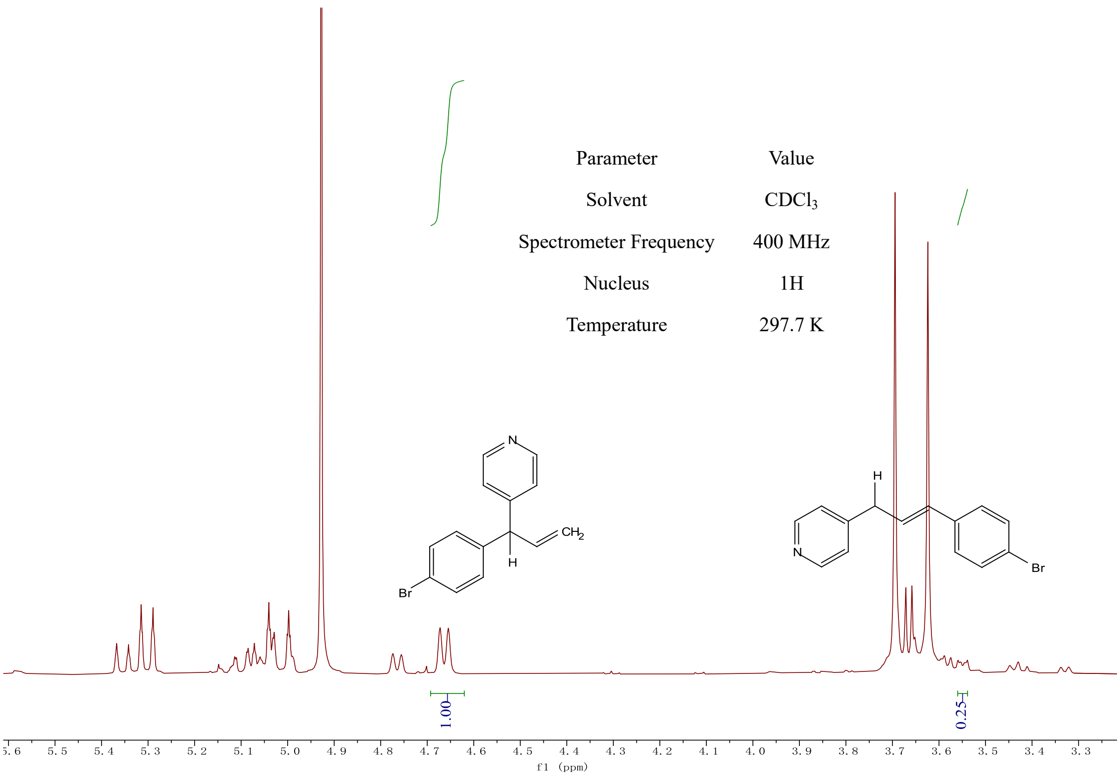

^{1}H NMR Spectrum of Crude Product **3ao**

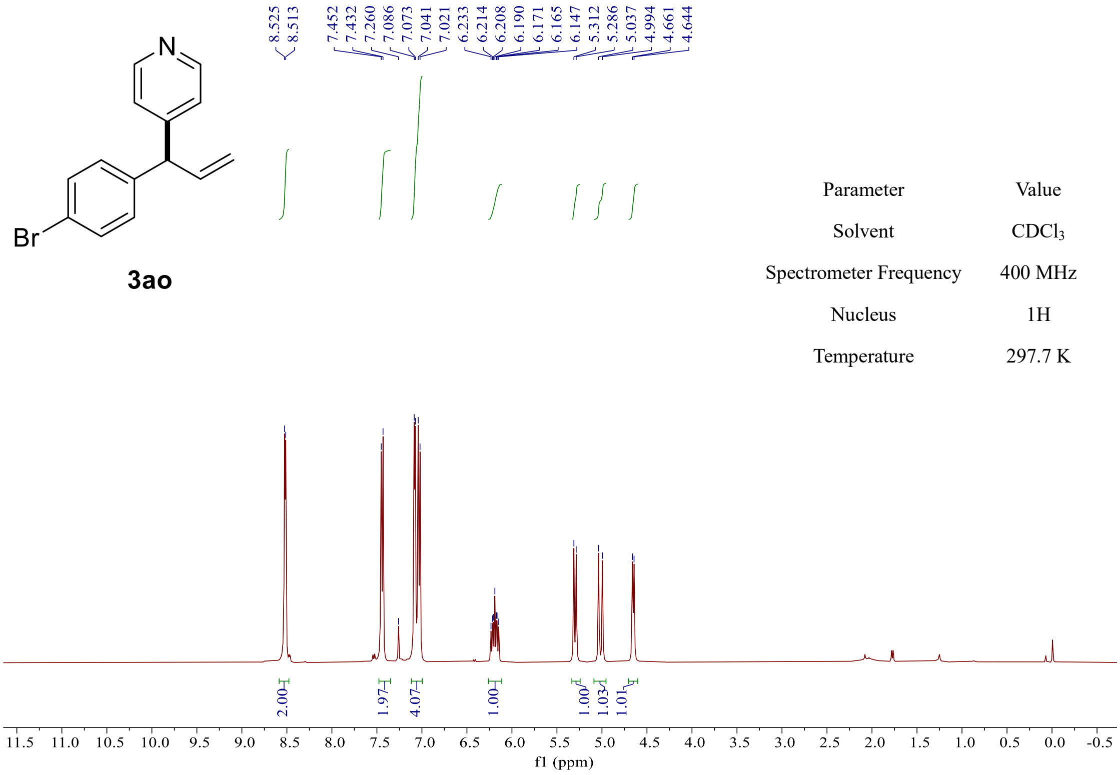

^{1}H NMR Spectrum of Compound **3ao**

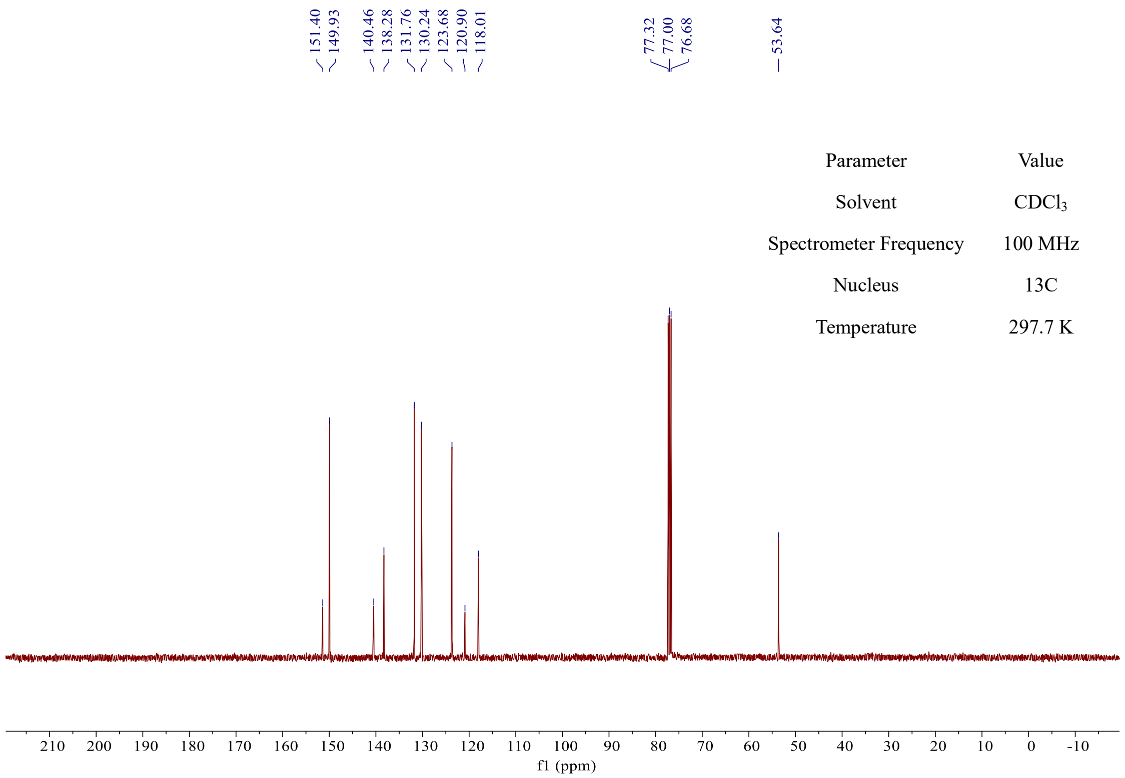

^{13}C NMR Spectrum of Compound **3ao**

Supplementary Figure 126**. NMR spectra of 3ao**

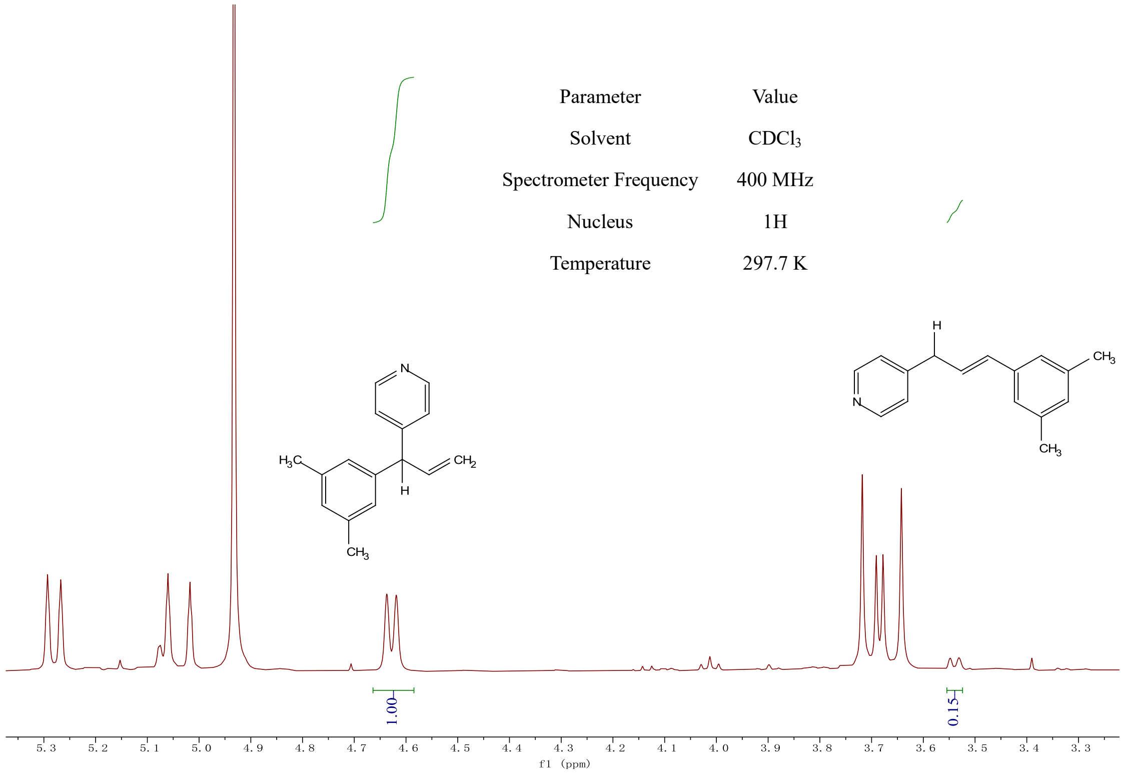

^{1}H NMR Spectrum of Crude Product **3ap**

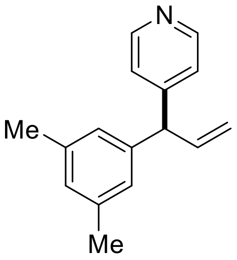

3ap

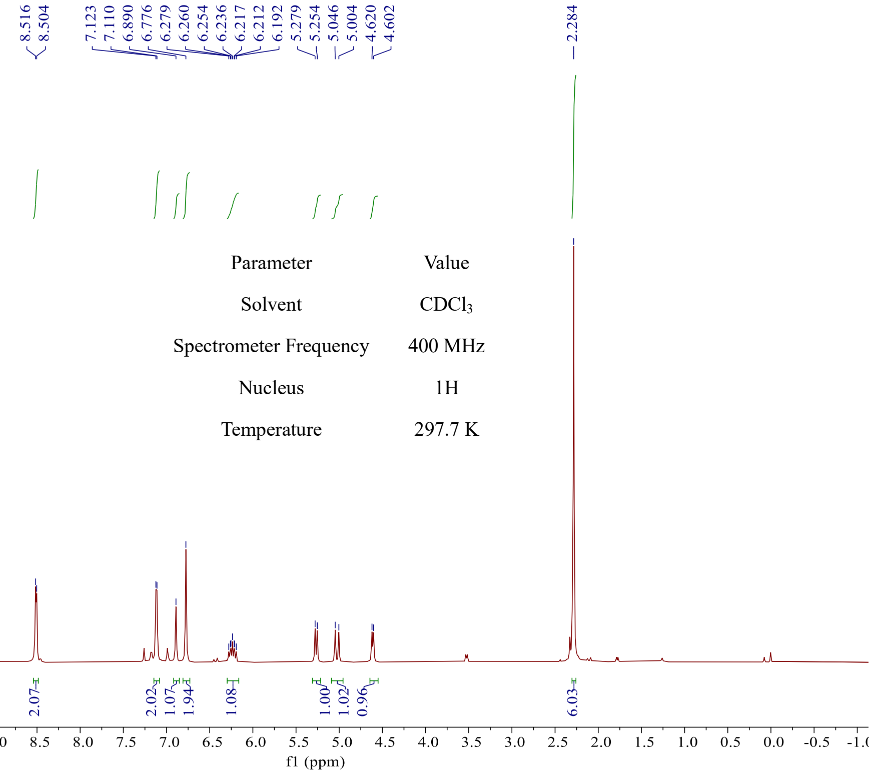

^{1}H NMR Spectrum of Compound **3ap**

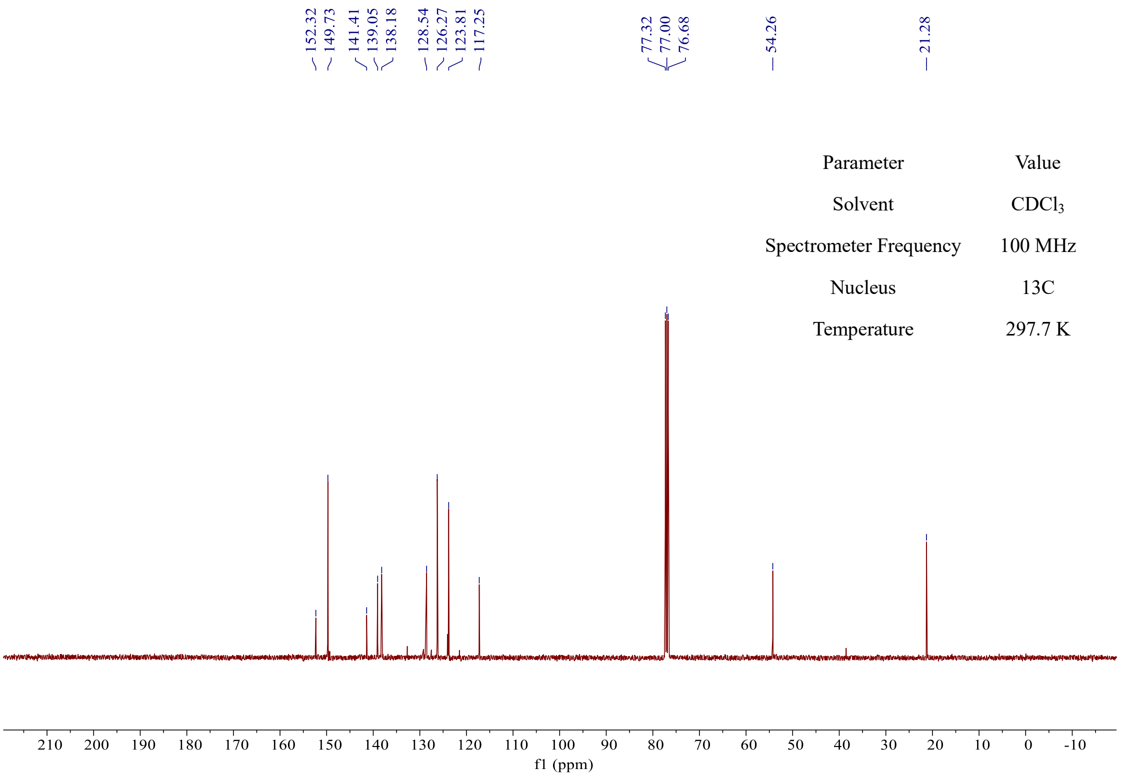

^{13}C NMR Spectrum of Compound **3ap**

Supplementary Figure 127**. NMR spectra of 3ap**

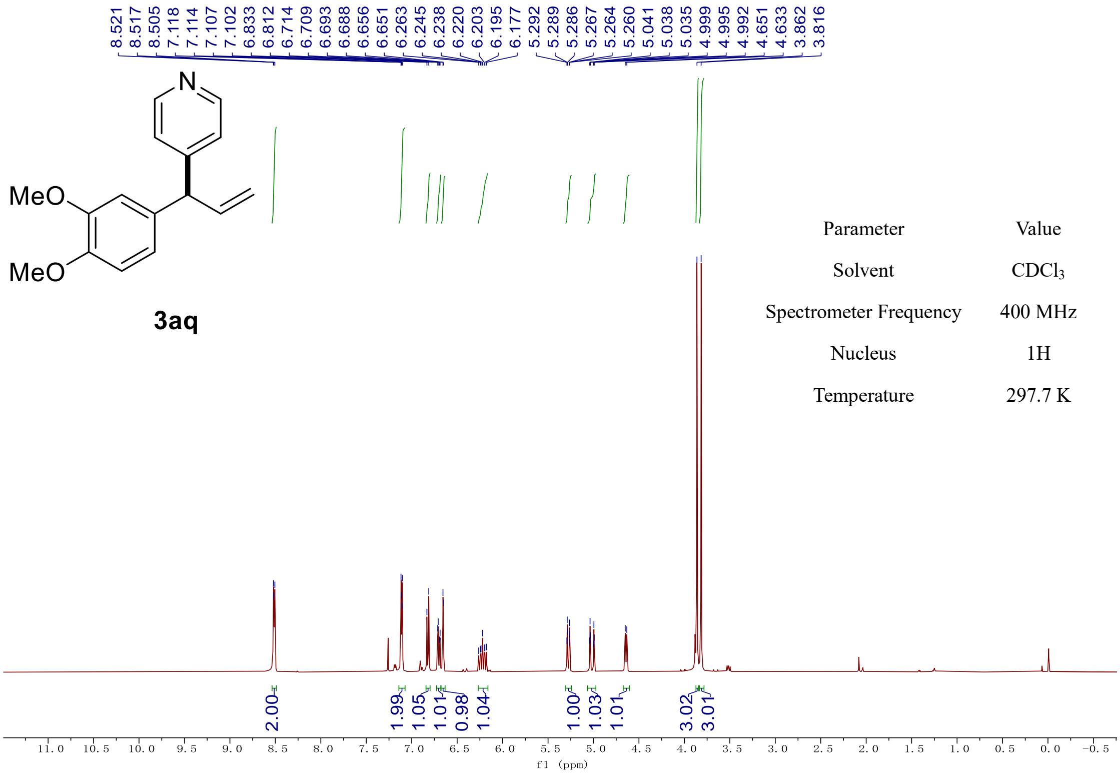

^{1}H NMR Spectrum of Compound **3aq**

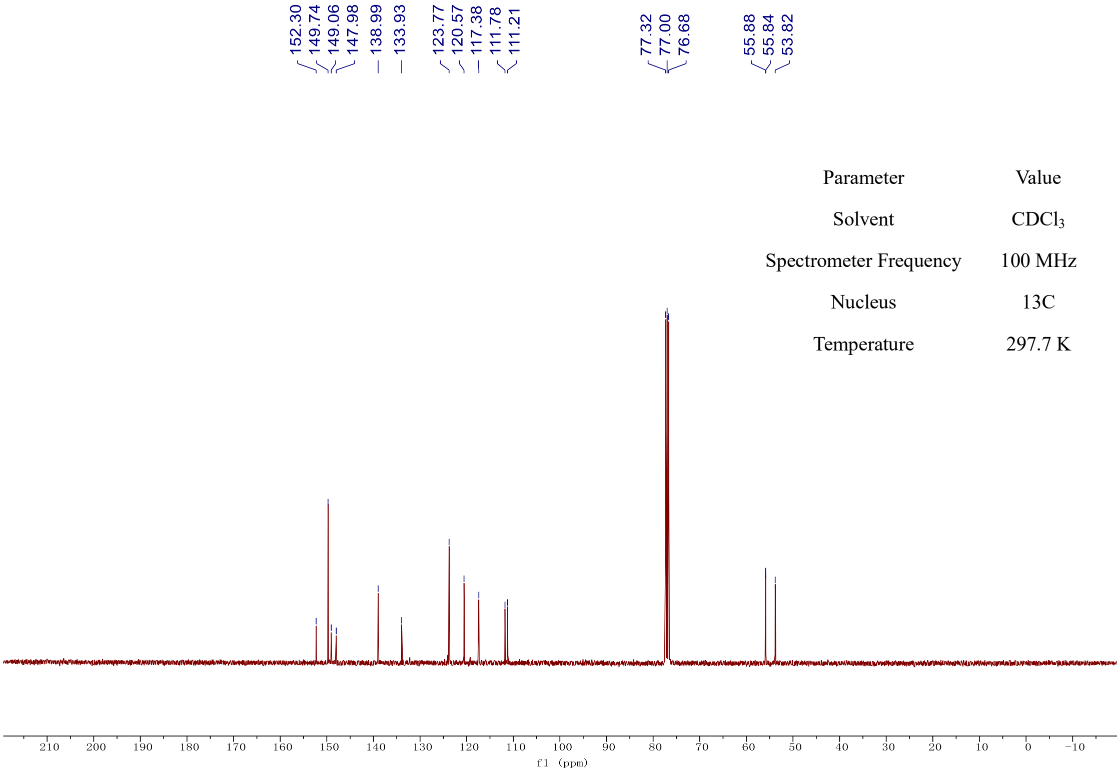

^{13}C NMR Spectrum of Compound **3aq**

Supplementary Figure 128**. NMR spectra of 3aq**

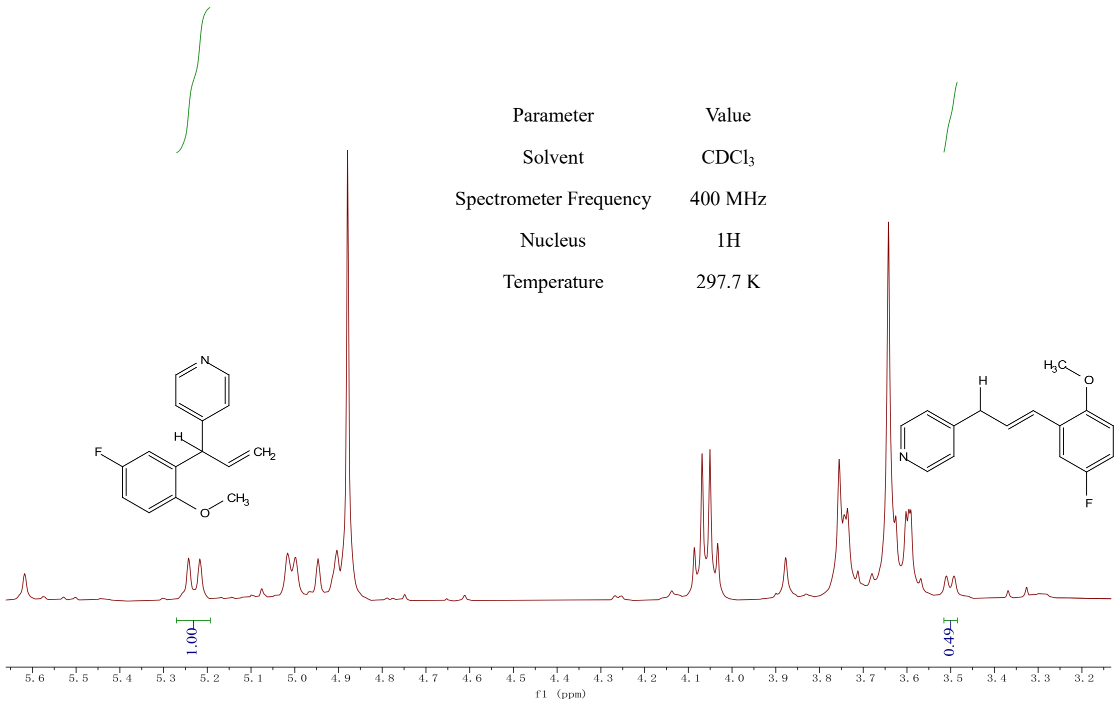

^{1}H NMR Spectrum of Crude Product **3ar**

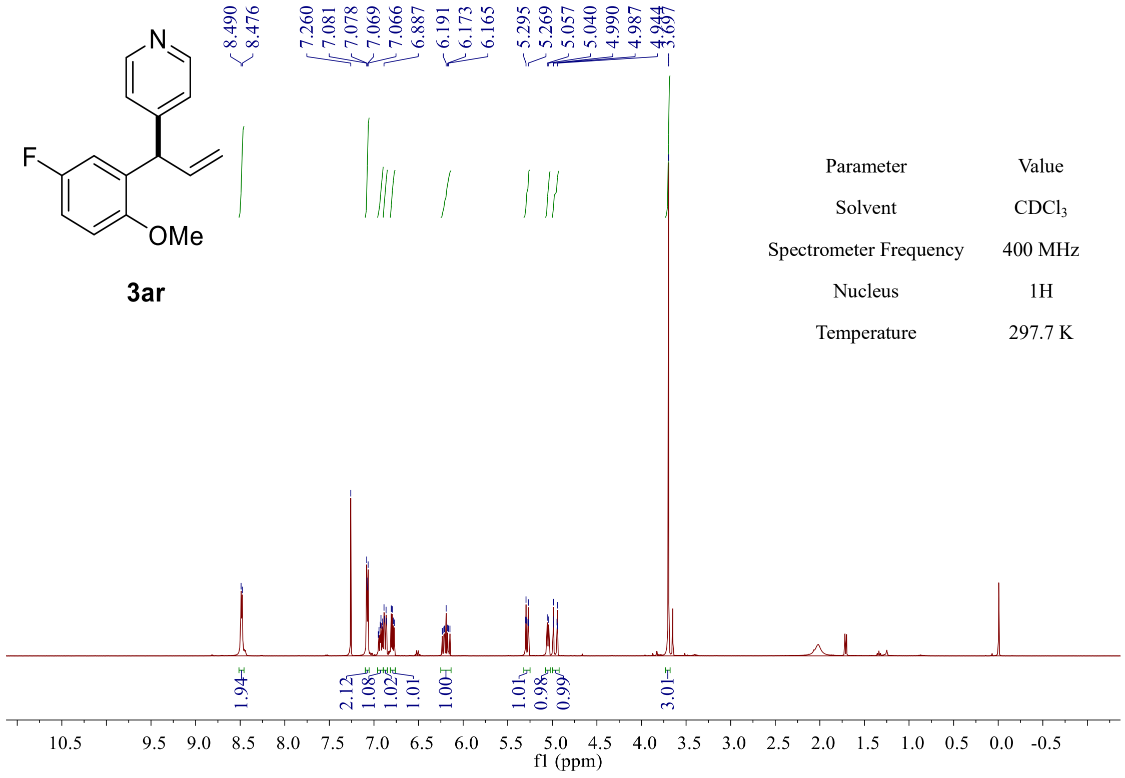

^{1}H NMR Spectrum of Compound **3ar**

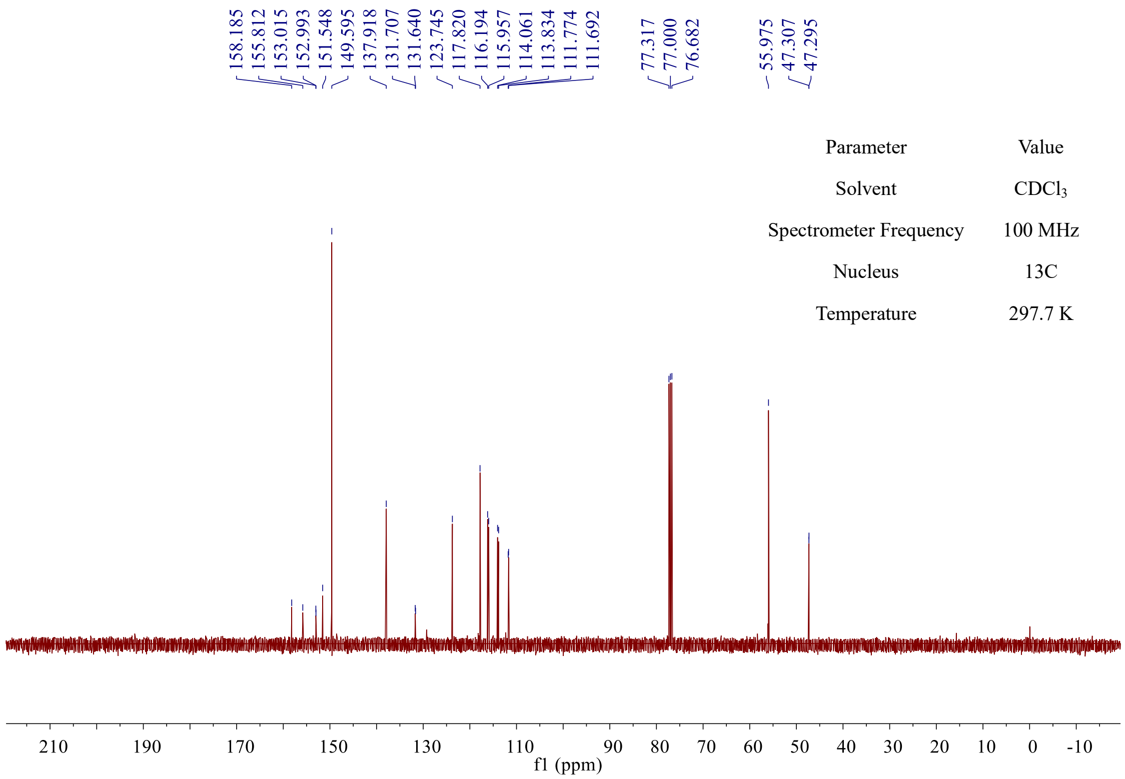

^{13}C NMR Spectrum of Compound **3ar**

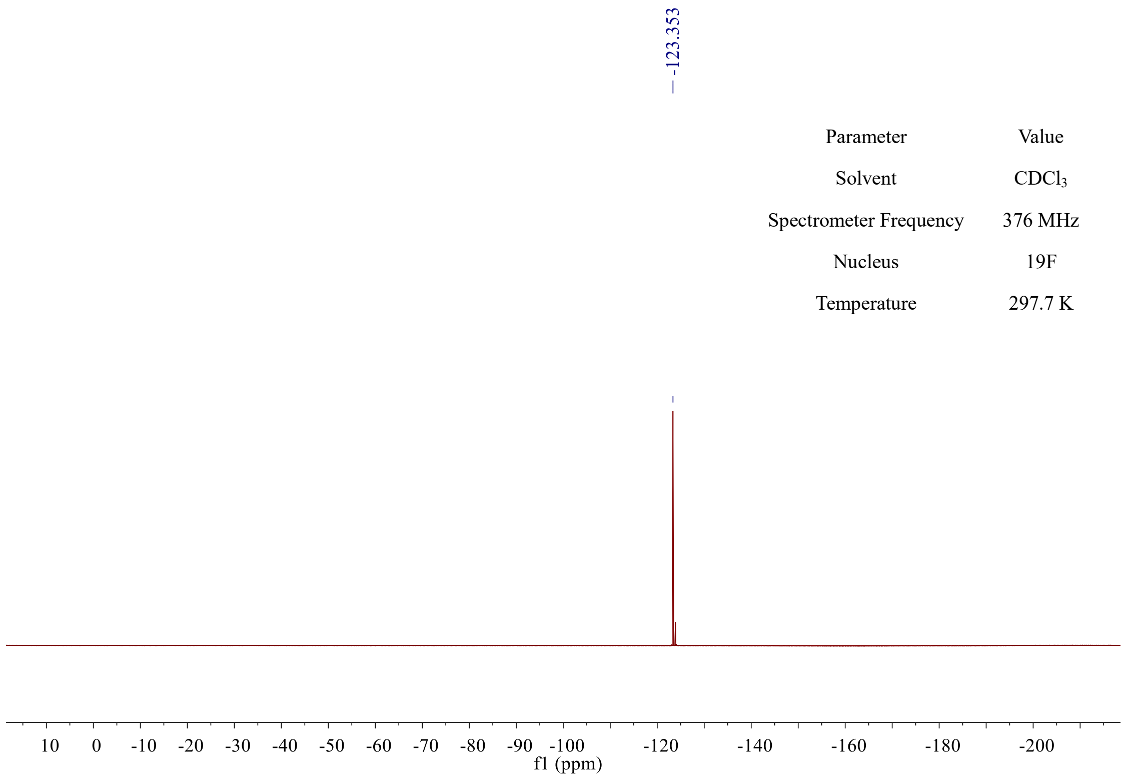

^{19}F NMR Spectrum of Compound **3ar**

Supplementary Figure 129. **NMR spectra of 3ar**

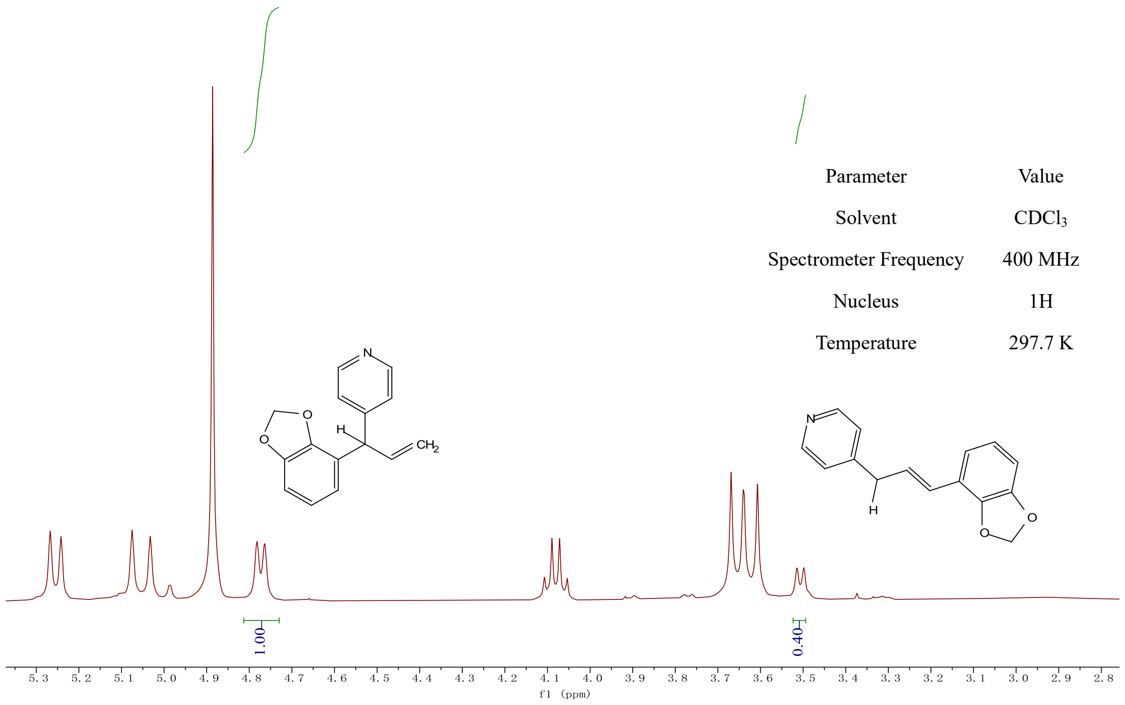

^{1}H NMR Spectrum of Crude Product **3as**

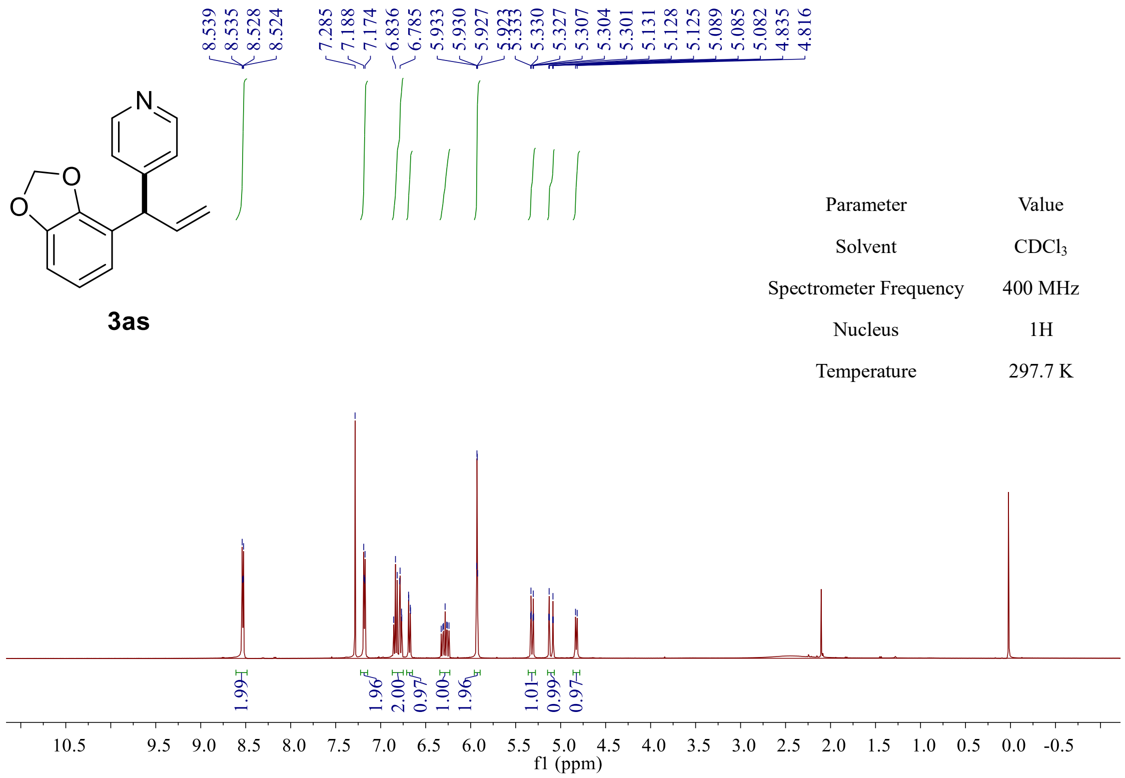

^{1}H NMR Spectrum of Compound **3as**

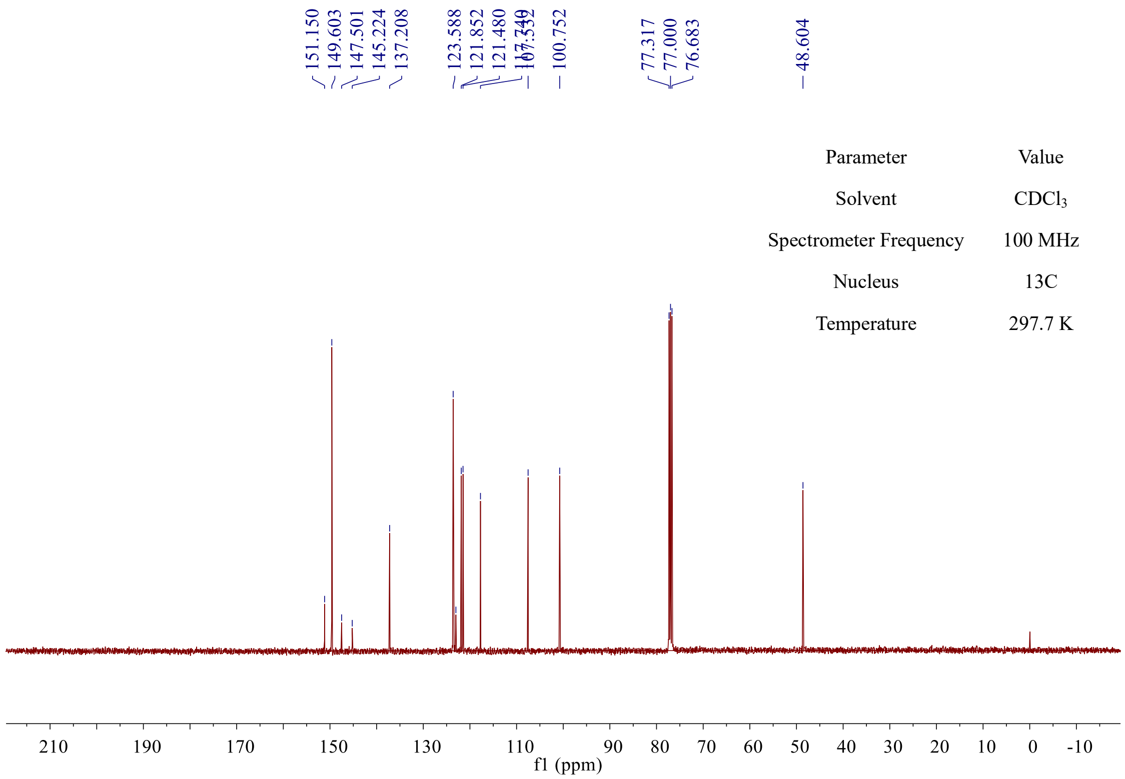

^{13}C NMR Spectrum of Compound **3as**

Supplementary Figure 130. **NMR spectra of 3as**

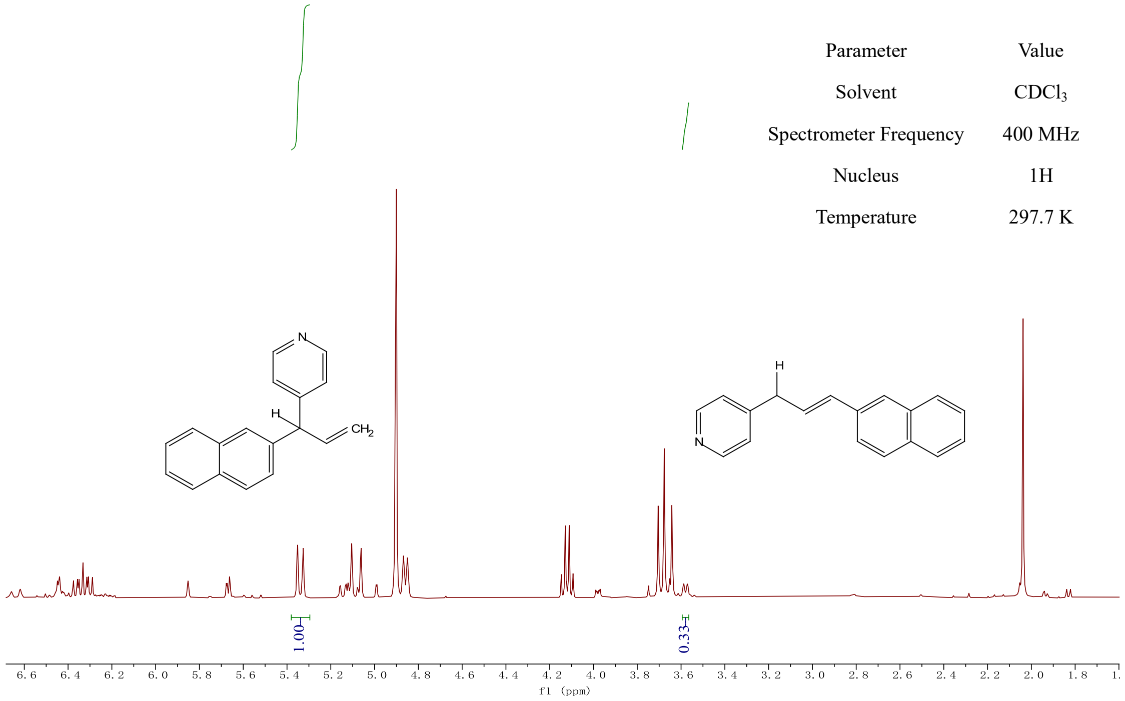

^{1}H NMR Spectrum of Crude Product **3at**

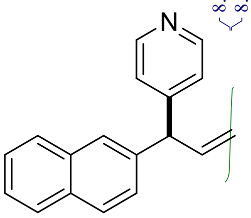

3at

Parameter	Value
Solvent	$CDCl_3$
Spectrometer Frequency	400 MHz
Nucleus	1H
Temperature	297.7 K

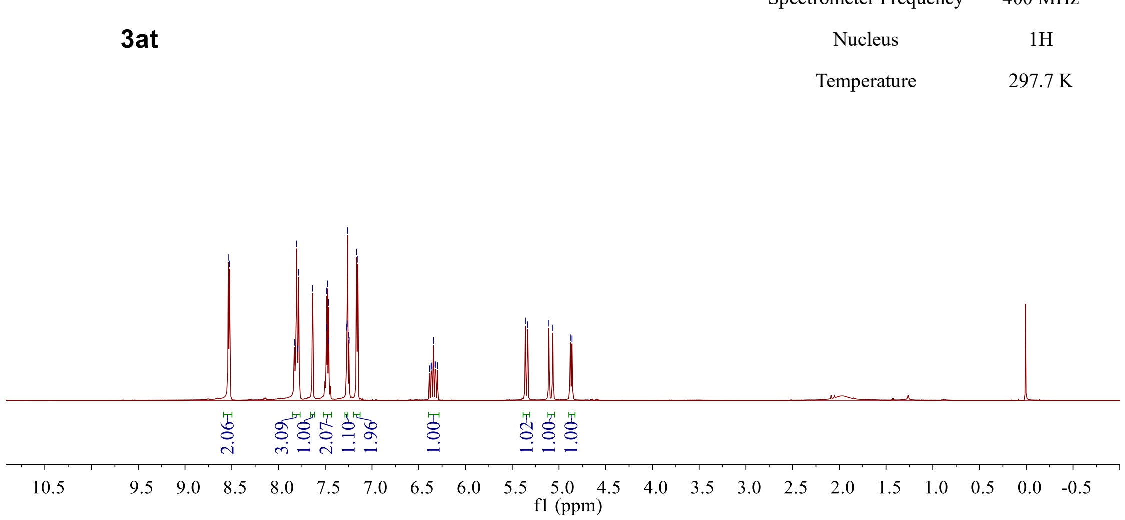

^{1}H NMR Spectrum of Compound **3at**

Parameter	Value
Solvent	$CDCl_3$
Spectrometer Frequency	100 MHz
Nucleus	13C
Temperature	297.7 K

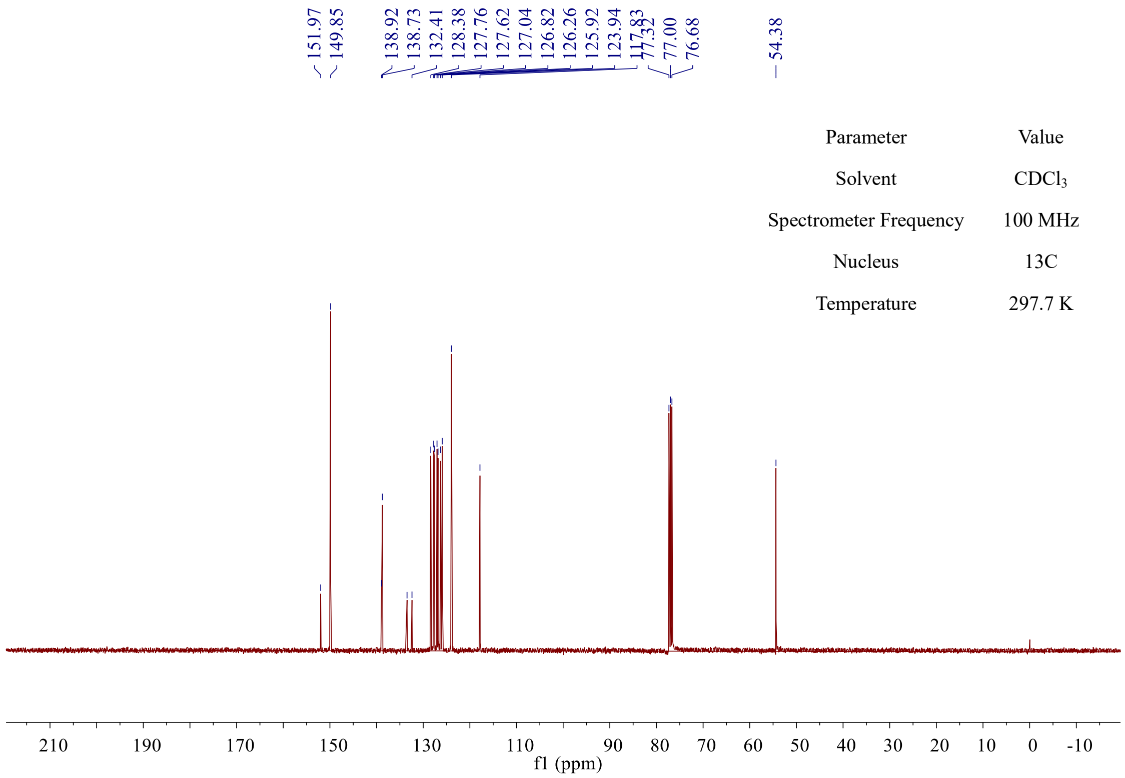

^{13}C NMR Spectrum of Compound **3at**

Supplementary Figure 131**. NMR spectra of 3at**

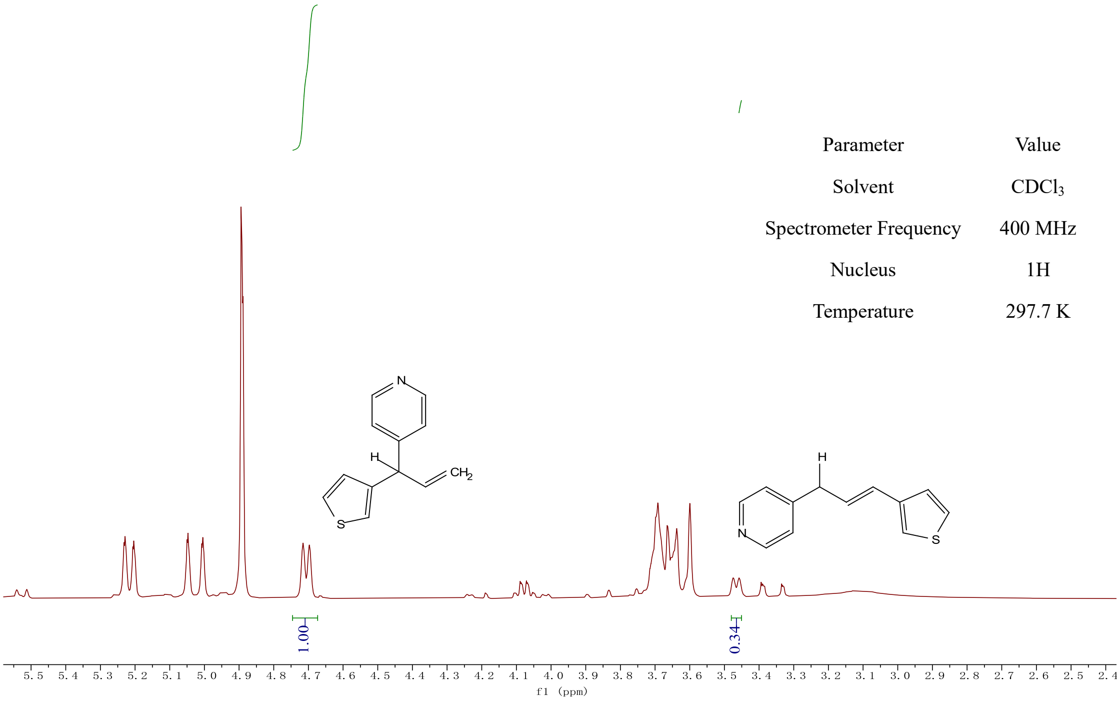

Parameter	Value
Solvent	$CDCl_3$
Spectrometer Frequency	400 MHz
Nucleus	1H
Temperature	297.7 K

^{1}H NMR Spectrum of Crude Product **3au**

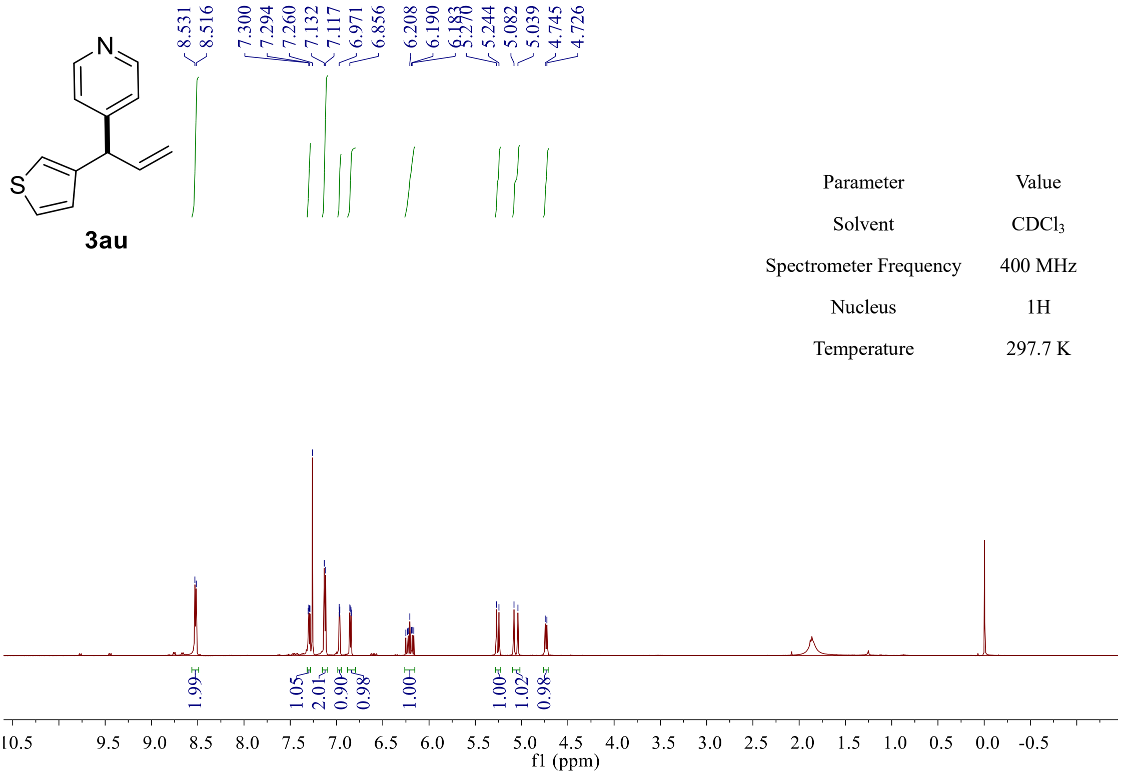

Parameter	Value
Solvent	$CDCl_3$
Spectrometer Frequency	400 MHz
Nucleus	1H
Temperature	297.7 K

^{1}H NMR Spectrum of Compound **3au**

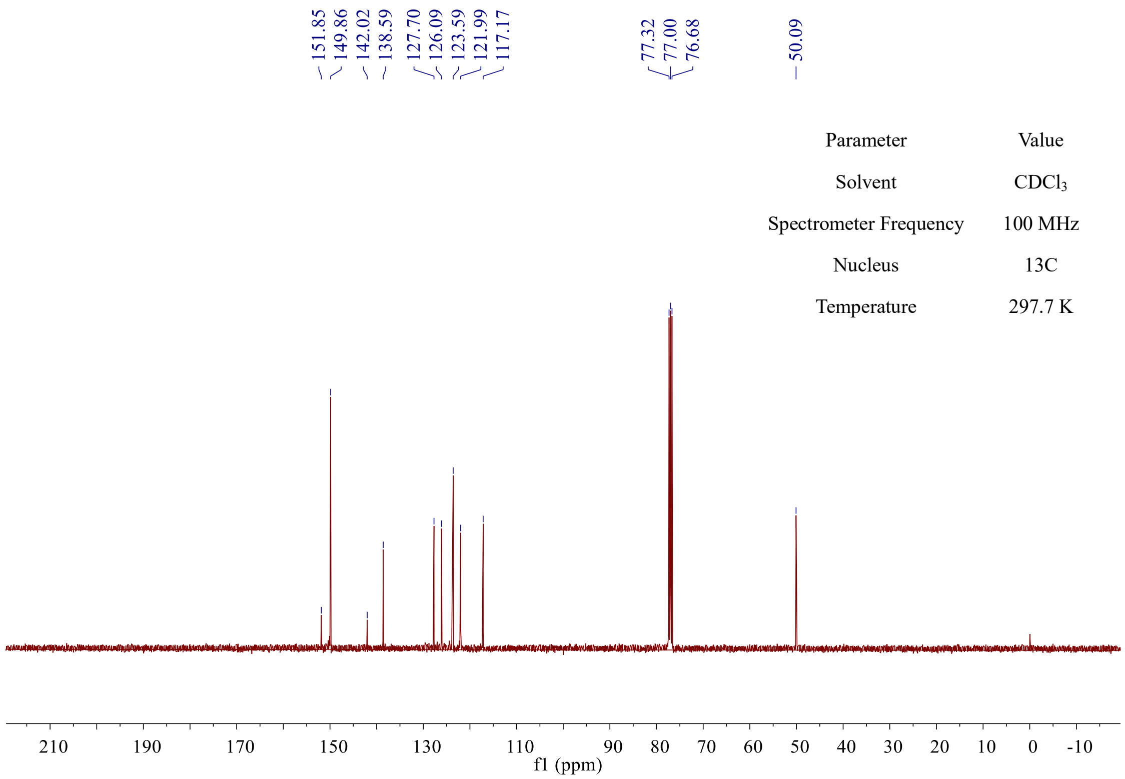

^{13}C NMR Spectrum of Compound **3au**

Supplementary Figure 132**. NMR spectra of 3au**

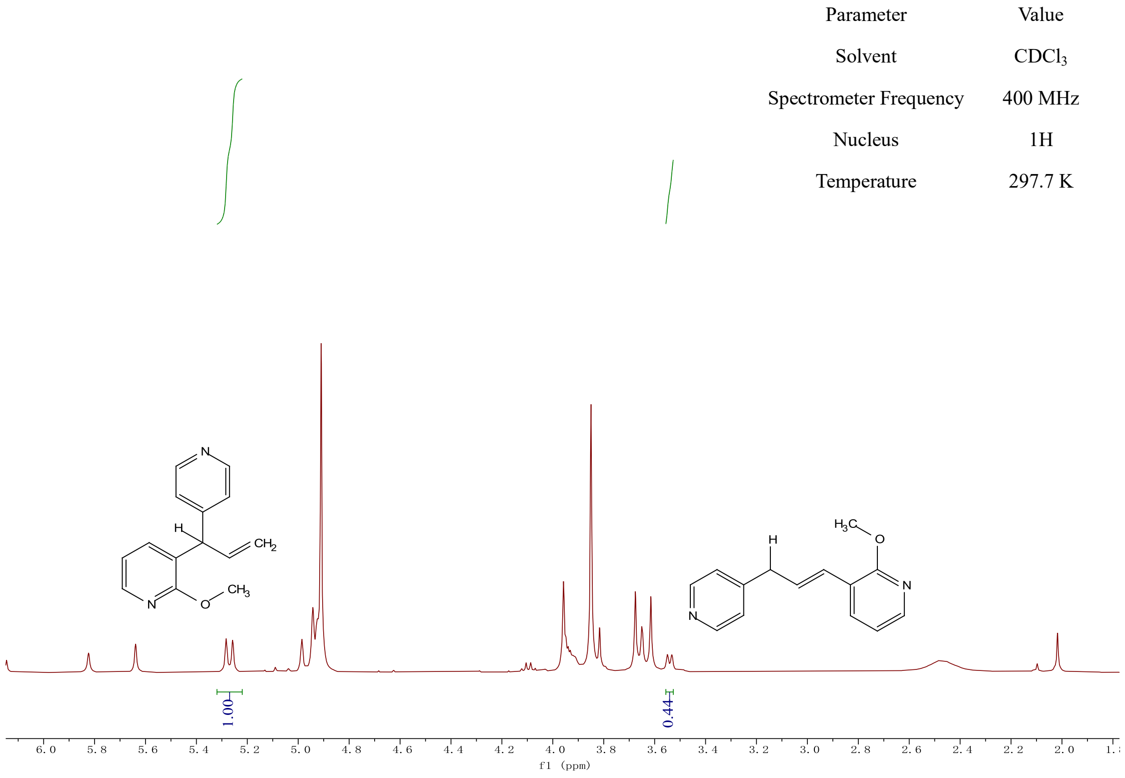

^{1}H NMR Spectrum of Crude Product **3av**

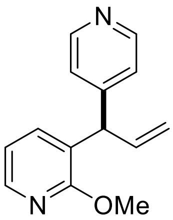

3av

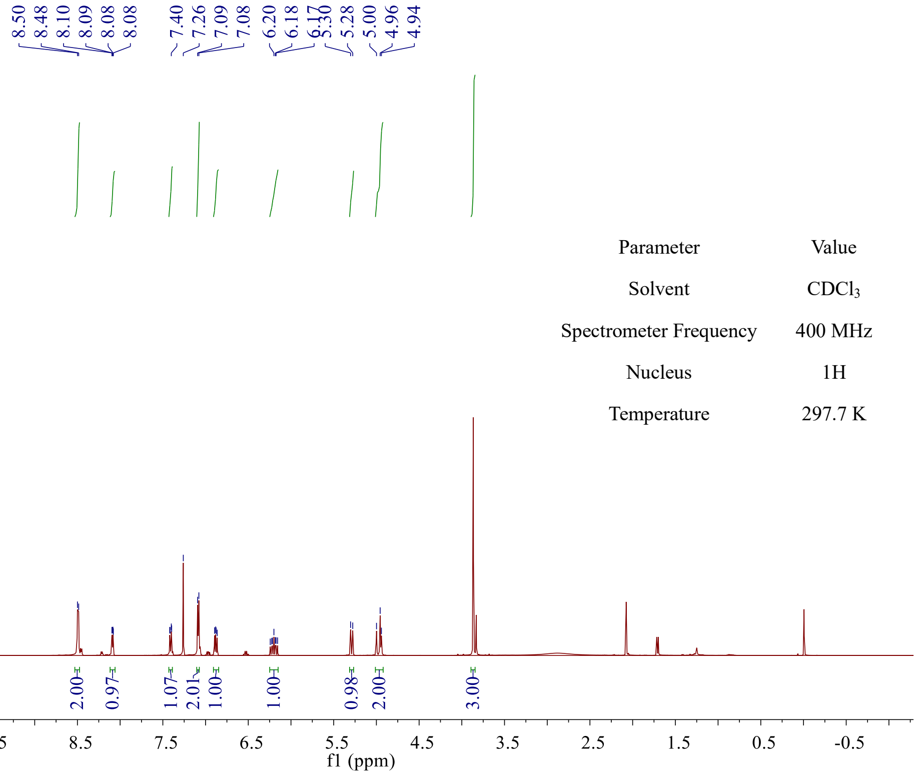

Parameter	Value
Solvent	$CDCl_3$
Spectrometer Frequency	400 MHz
Nucleus	1H
Temperature	297.7 K

^{1}H NMR Spectrum of Compound **3av**

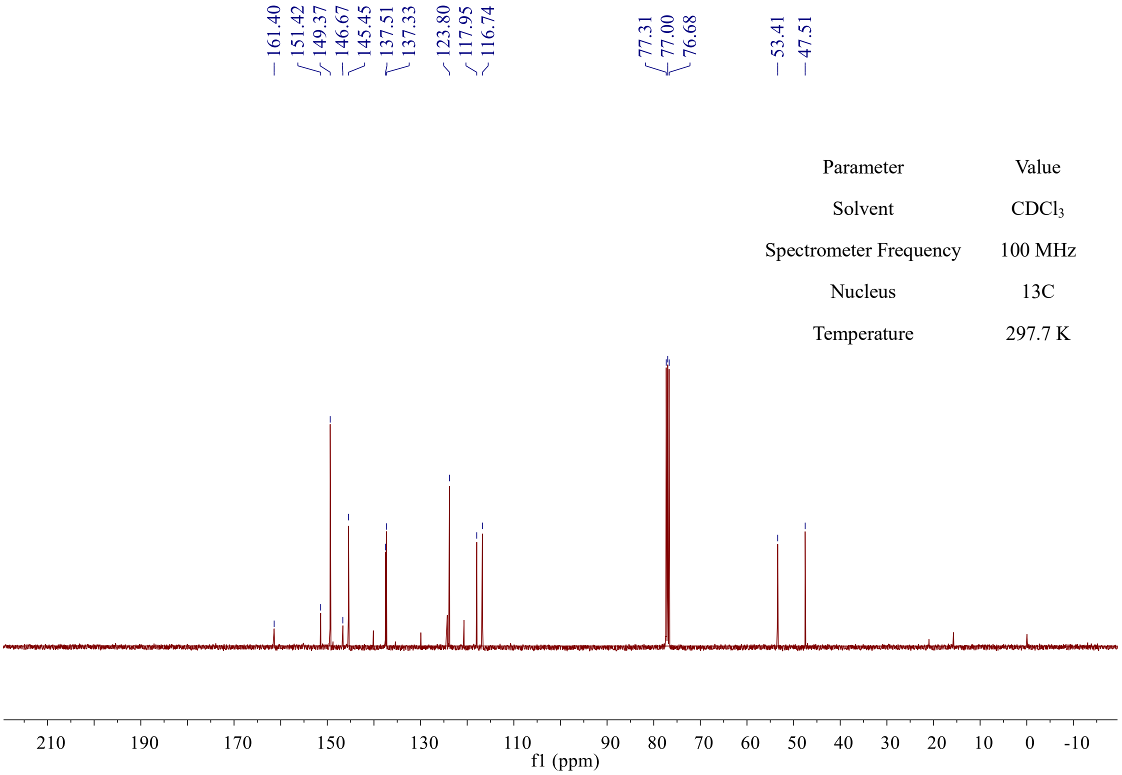

Parameter	Value
Solvent	$CDCl_3$
Spectrometer Frequency	100 MHz
Nucleus	13C
Temperature	297.7 K

^{13}C NMR Spectrum of Compound **3av**

Supplementary Figure 133. **NMR spectra of 3av**

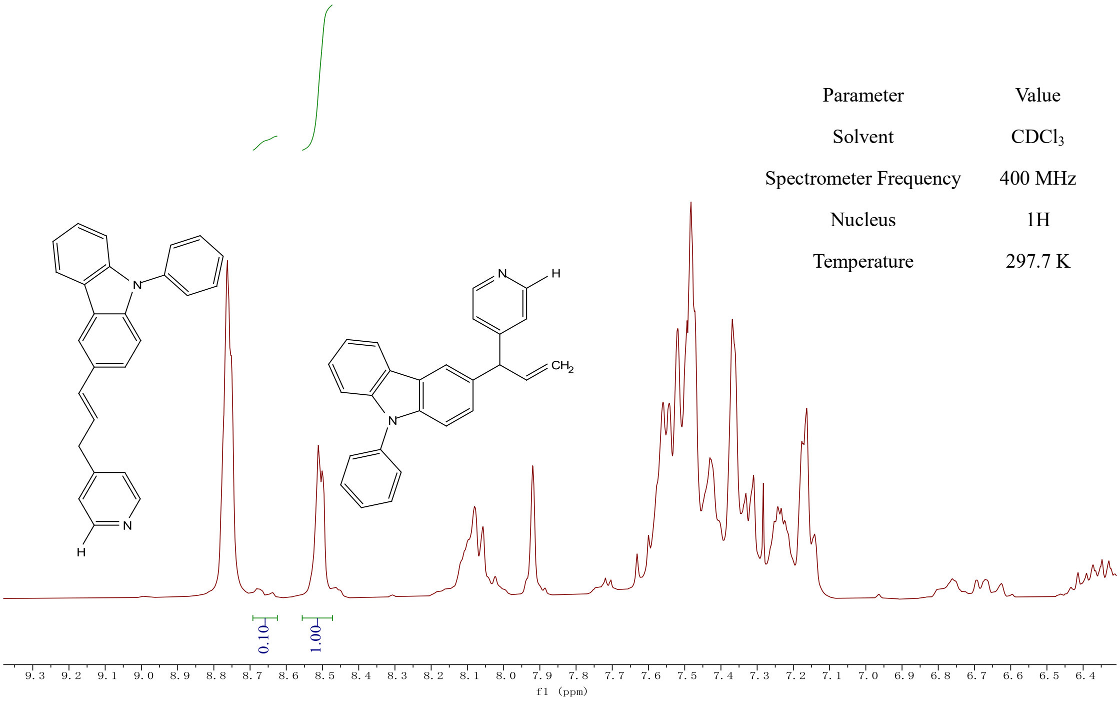

Parameter	Value
Solvent	$CDCl_3$
Spectrometer Frequency	400 MHz
Nucleus	1H
Temperature	297.7 K

^{1}H NMR Spectrum of Crude Product **3aw**

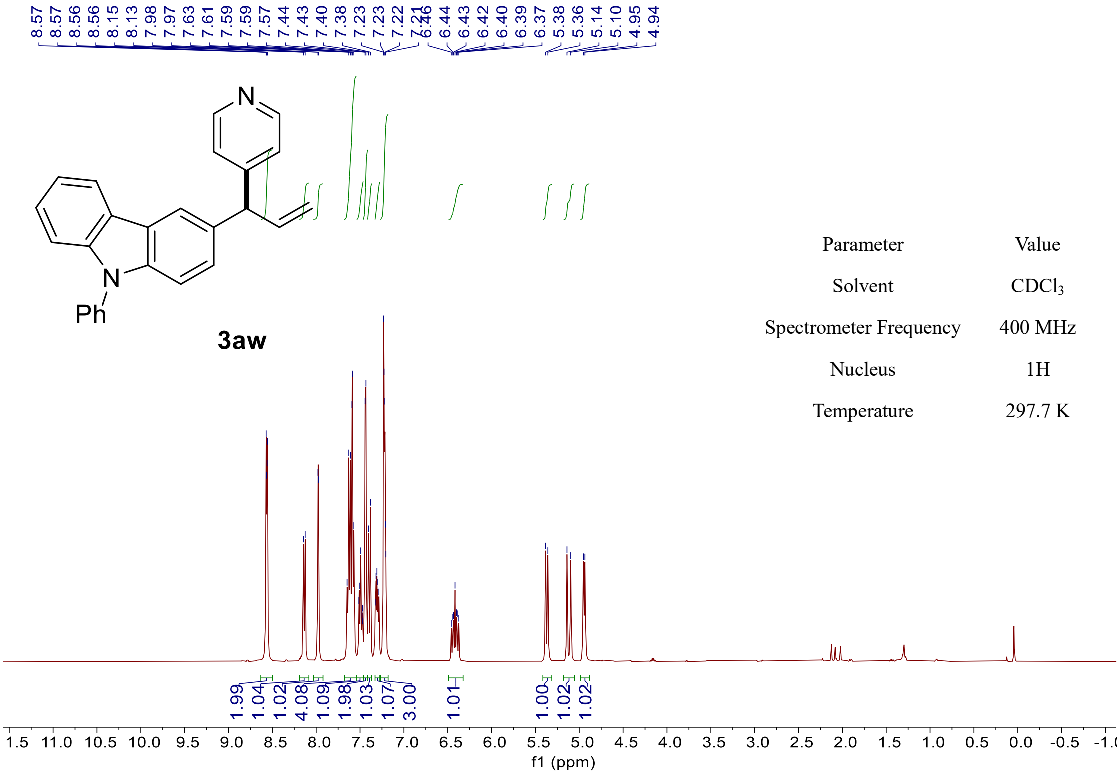

Parameter	Value
Solvent	$CDCl_3$
Spectrometer Frequency	400 MHz
Nucleus	1H
Temperature	297.7 K

^{1}H NMR Spectrum of Compound **3aw**

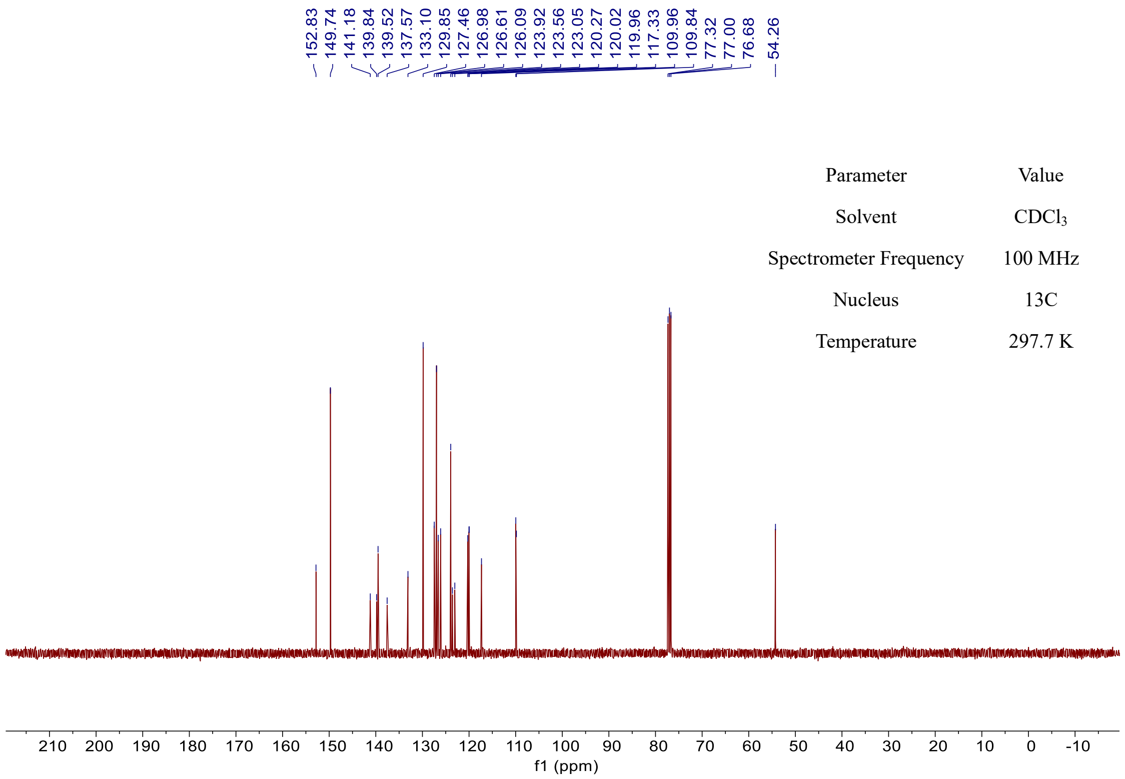

^{13}C NMR Spectrum of Compound **3aw**

Supplementary Figure 134**. NMR spectra of 3aw**

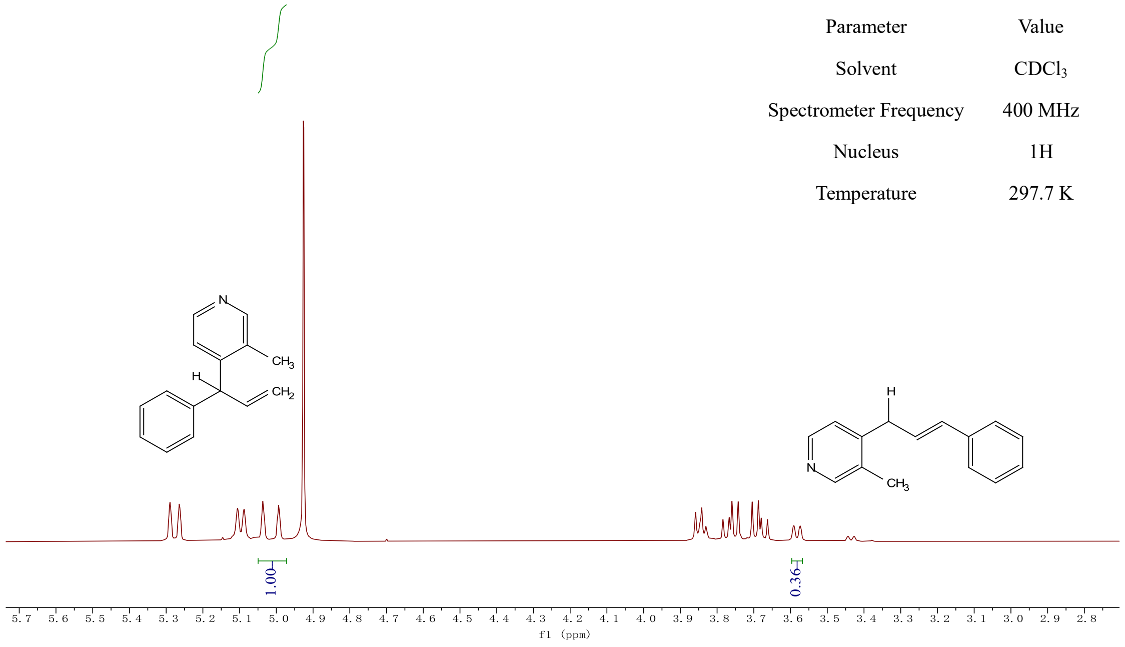

^{1}H NMR Spectrum of Crude Product **3ax**

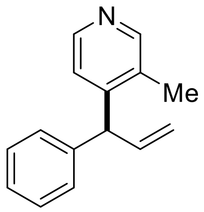

3ax

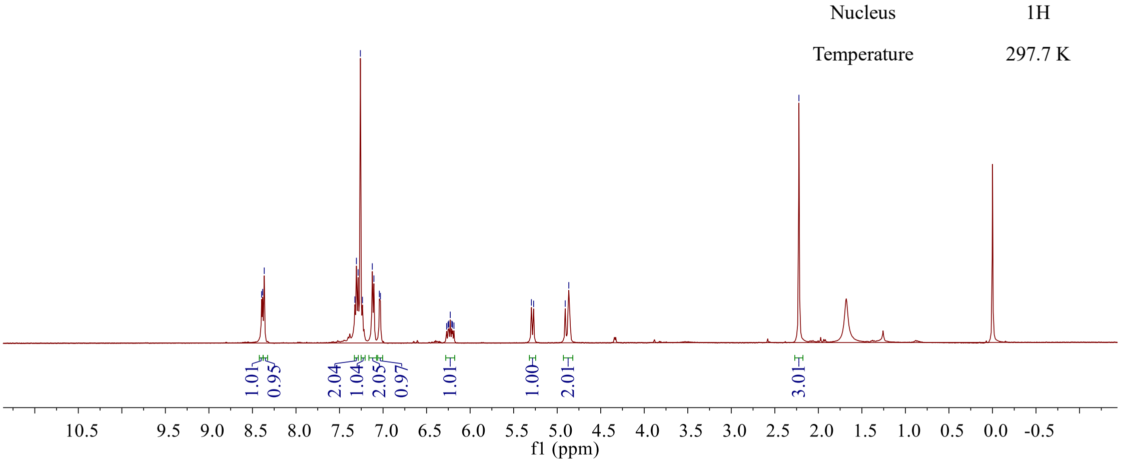

Parameter	Value
Solvent	$CDCl_3$
Spectrometer Frequency	400 MHz
Nucleus	1H
Temperature	297.7 K

^{1}H NMR Spectrum of Compound **3ax**

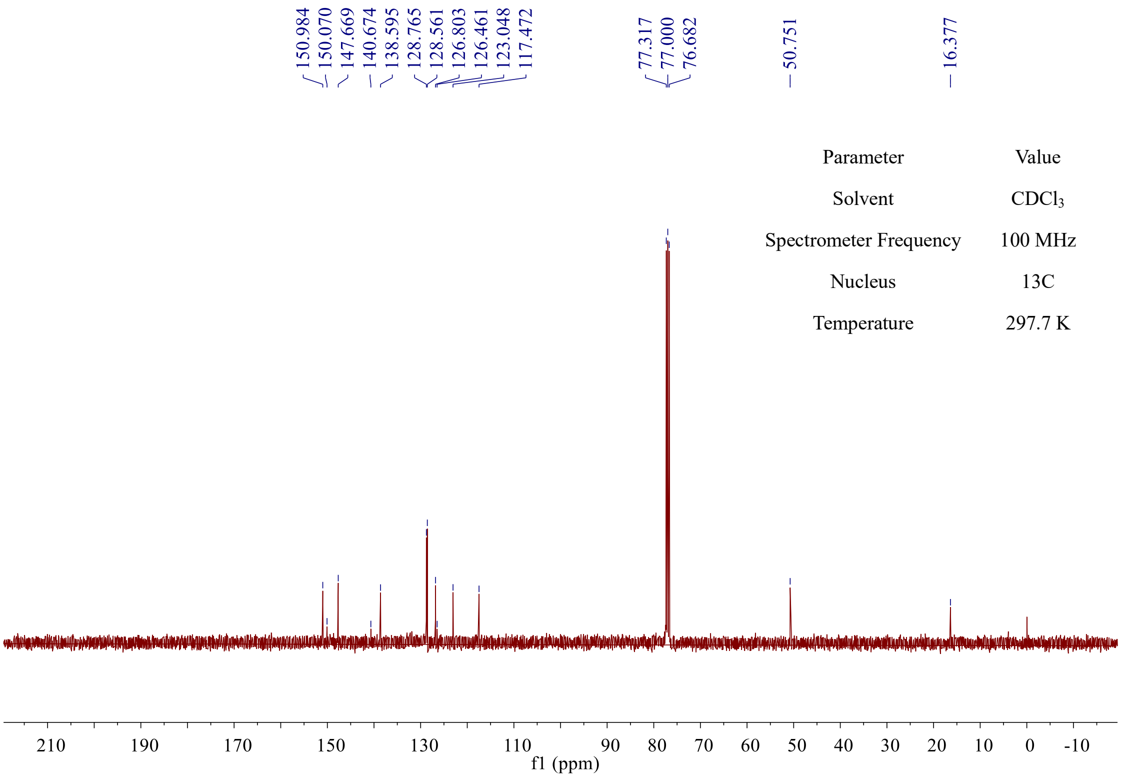

Parameter	Value
Solvent	$CDCl_3$
Spectrometer Frequency	100 MHz
Nucleus	13C
Temperature	297.7 K

^{13}C NMR Spectrum of Compound **3ax**

Supplementary Figure 135**. NMR spectra of 3ax**

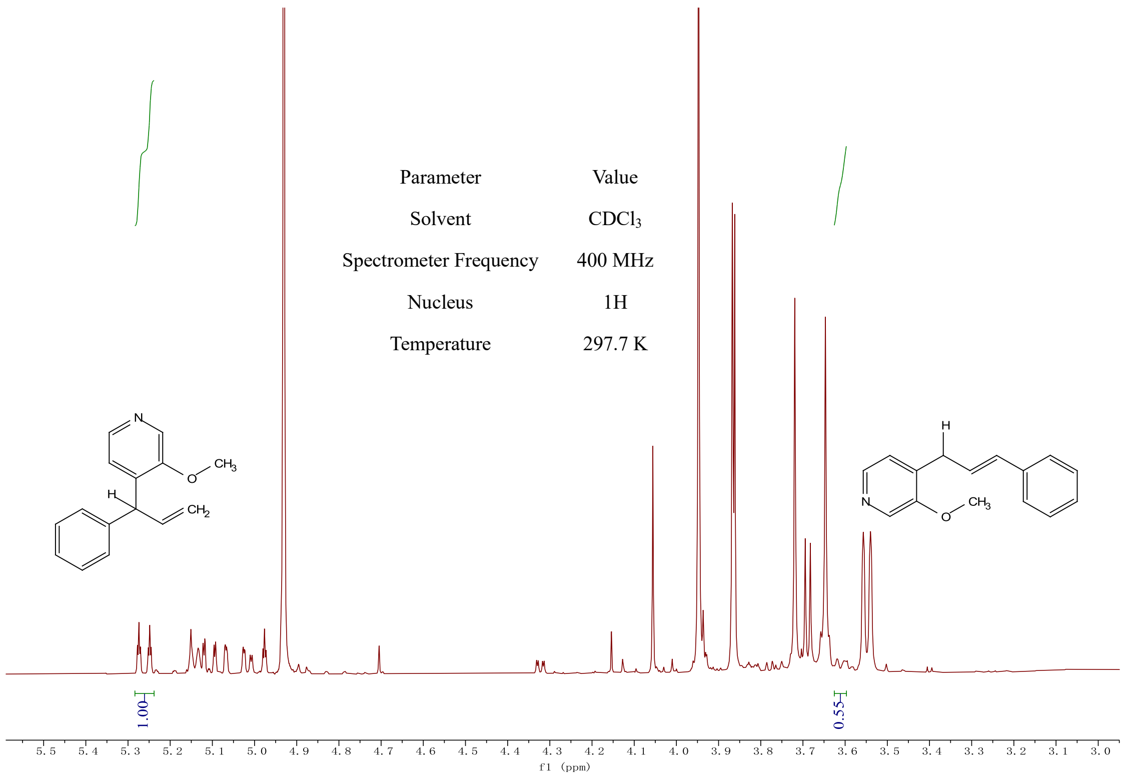

^{1}H NMR Spectrum of Crude Product **3ay**

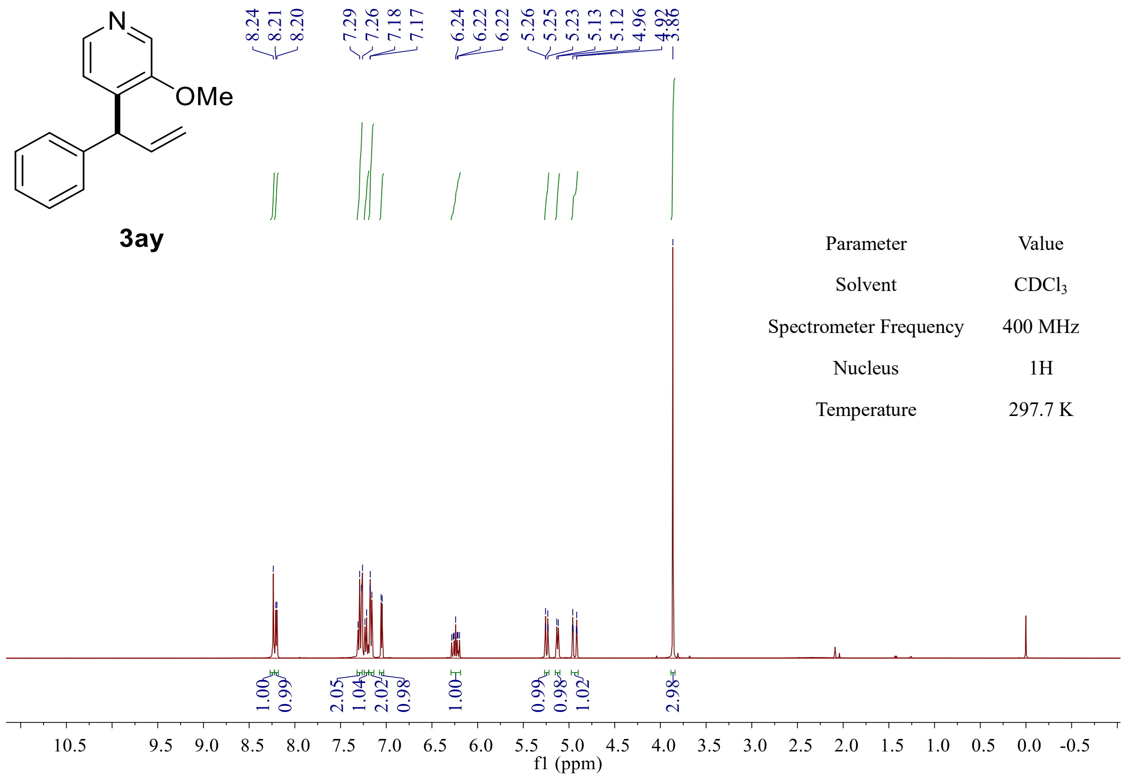

^{1}H NMR Spectrum of Compound **3ay**

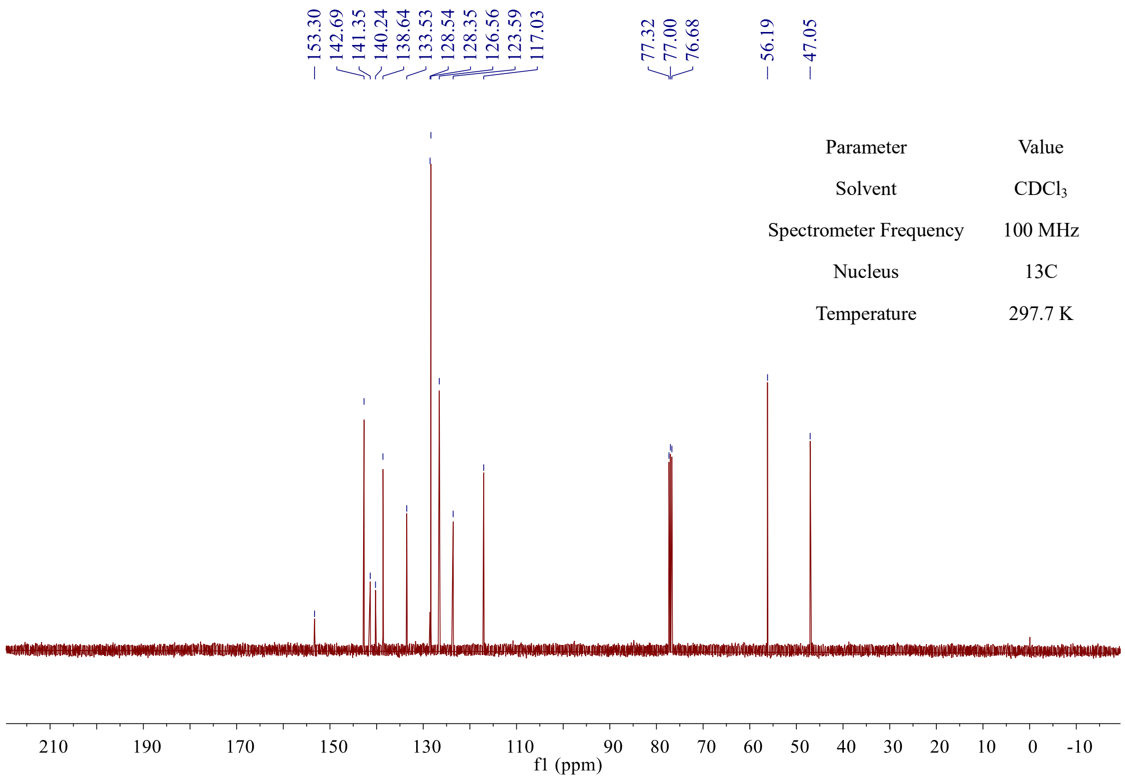

^{13}C NMR Spectrum of Compound **3ay**

Supplementary Figure 136**. NMR spectra of 3ay**

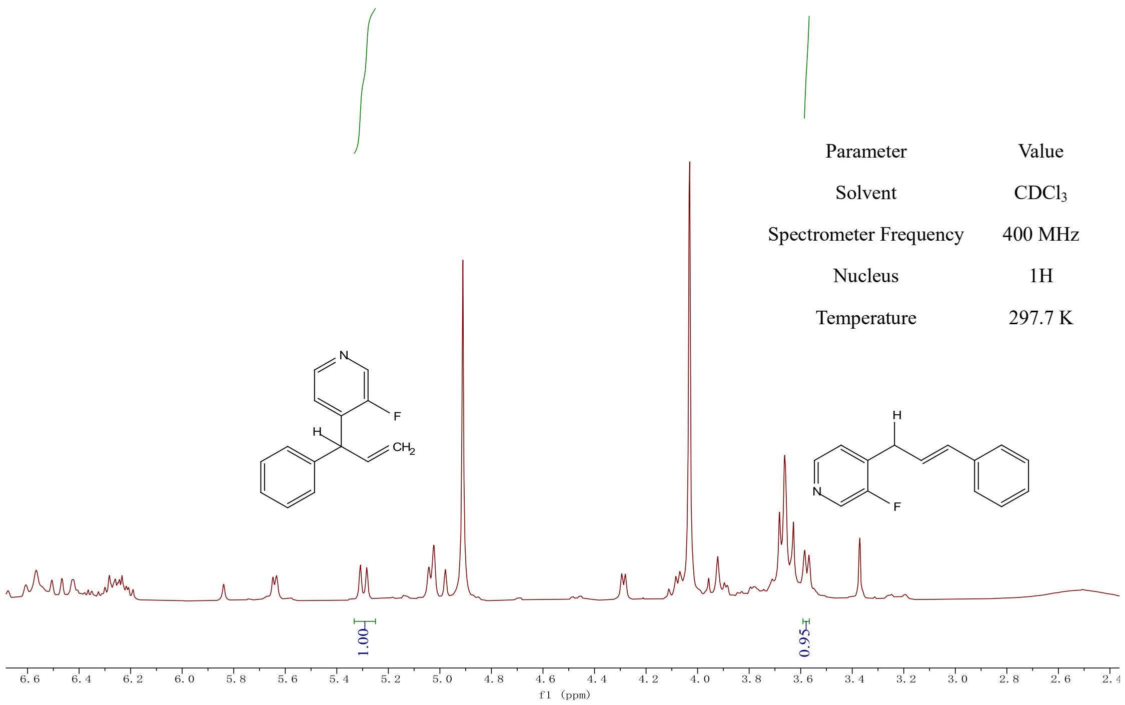

^{1}H NMR Spectrum of Crude Product **3az**

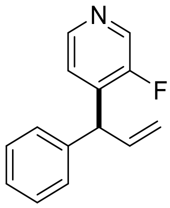

3az

Parameter	Value
Solvent	$CDCl_3$
Spectrometer Frequency	400 MHz
Nucleus	1H
Temperature	297.7 K

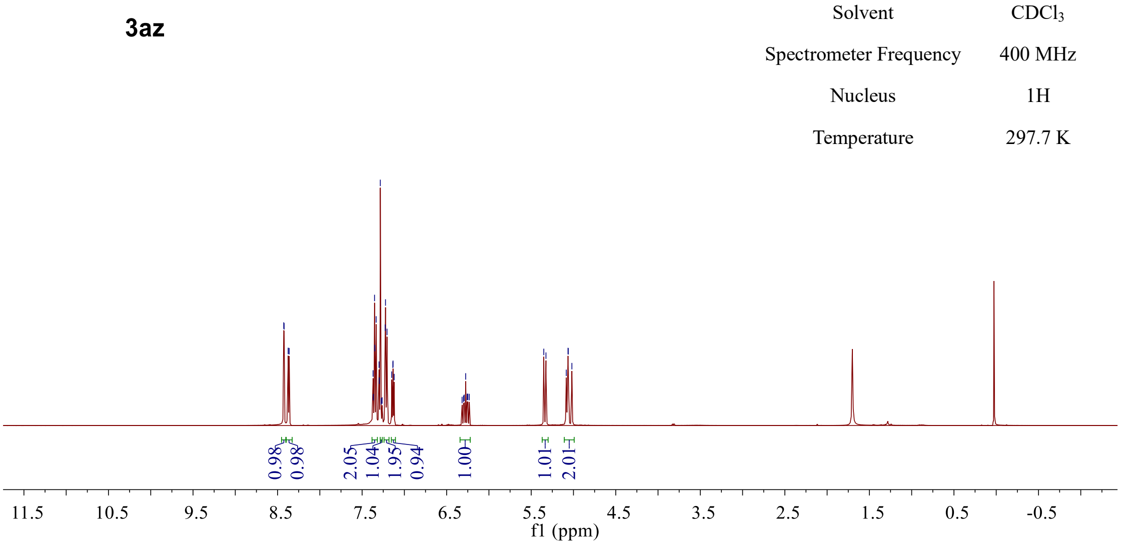

^{1}H NMR Spectrum of Compound **3az**

Parameter	Value
Solvent	$CDCl_3$
Spectrometer Frequency	100 MHz
Nucleus	13C
Temperature	297.7 K

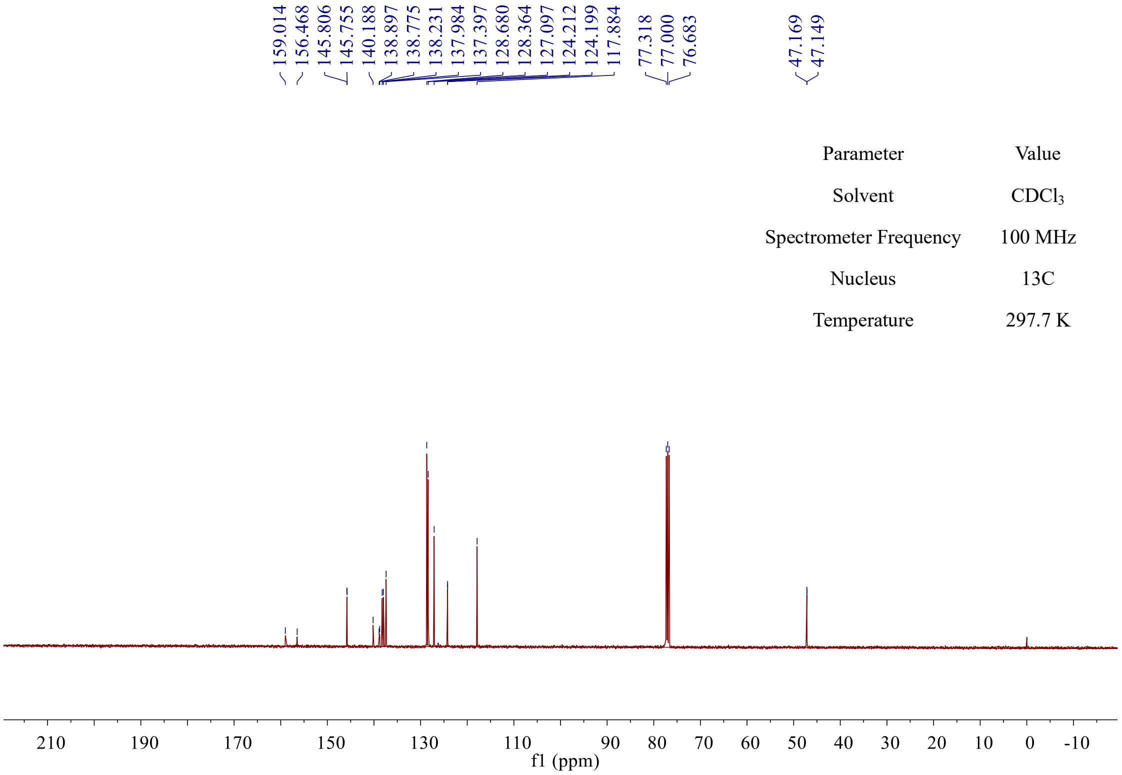

^{13}C NMR Spectrum of Compound **3az**

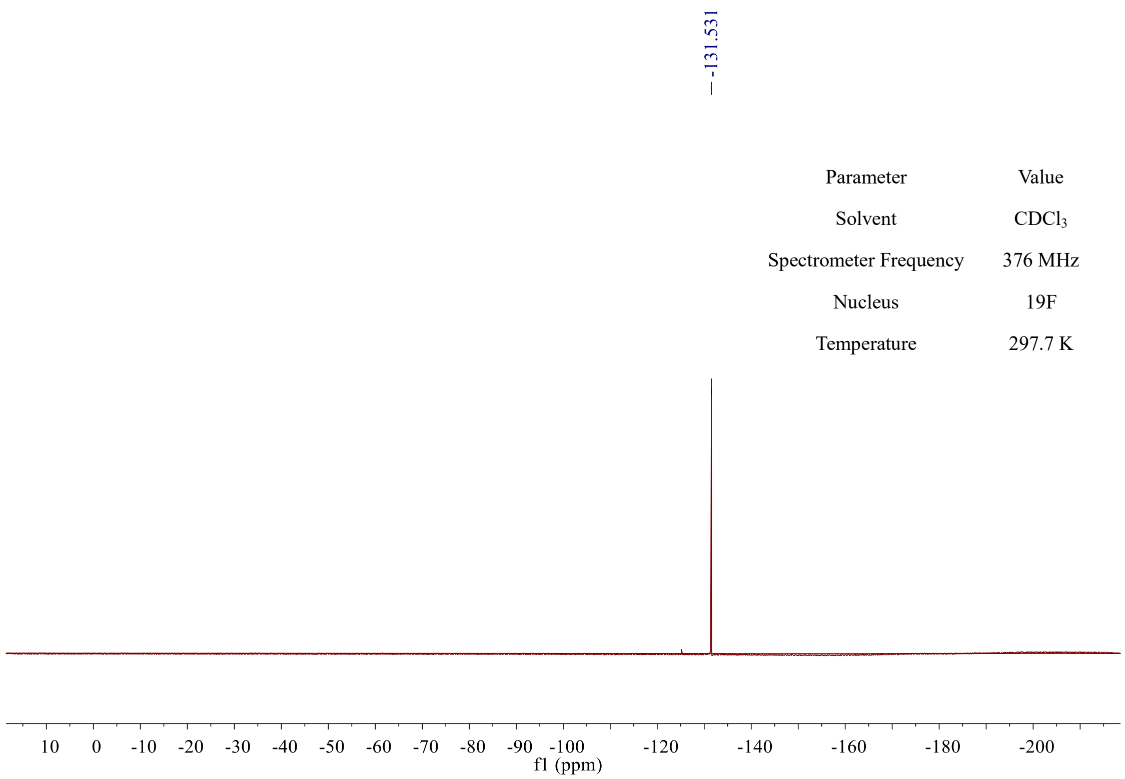

^{19}F NMR Spectrum of Compound **3az**

Supplementary Figure 137**. NMR spectra of 3az**

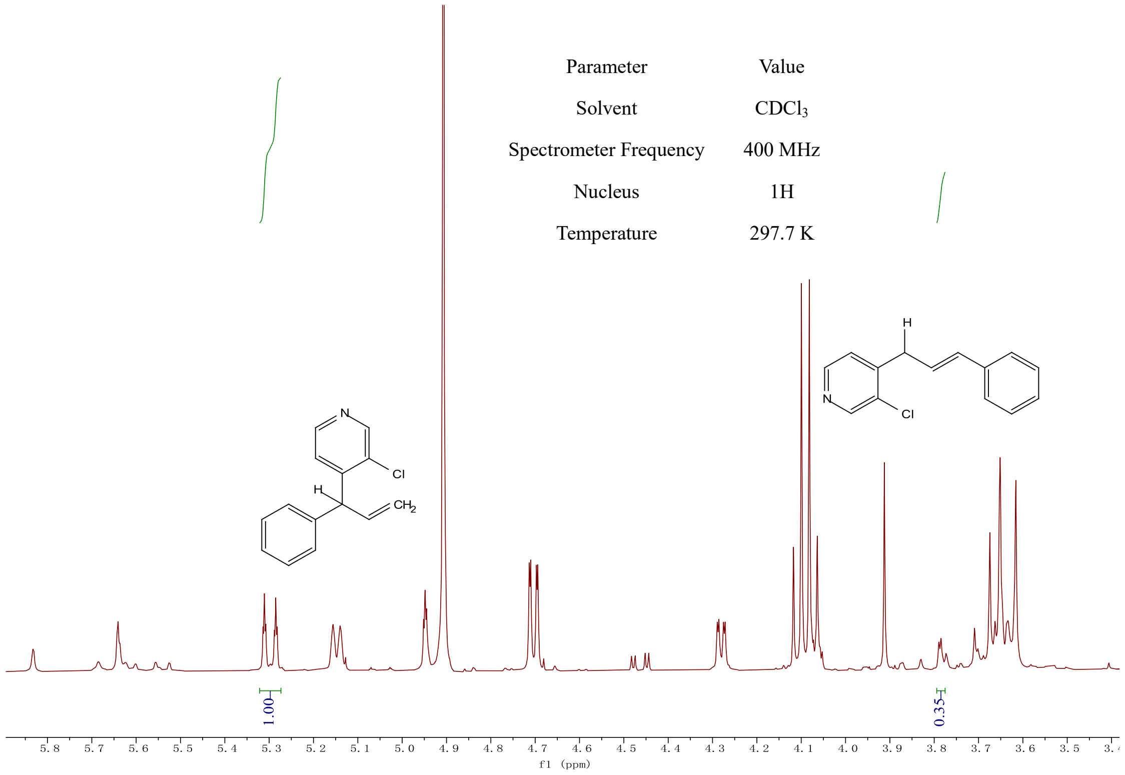

^{1}H NMR Spectrum of Crude Product **3ba**

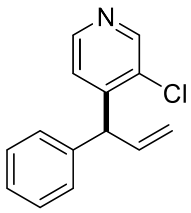

3ba

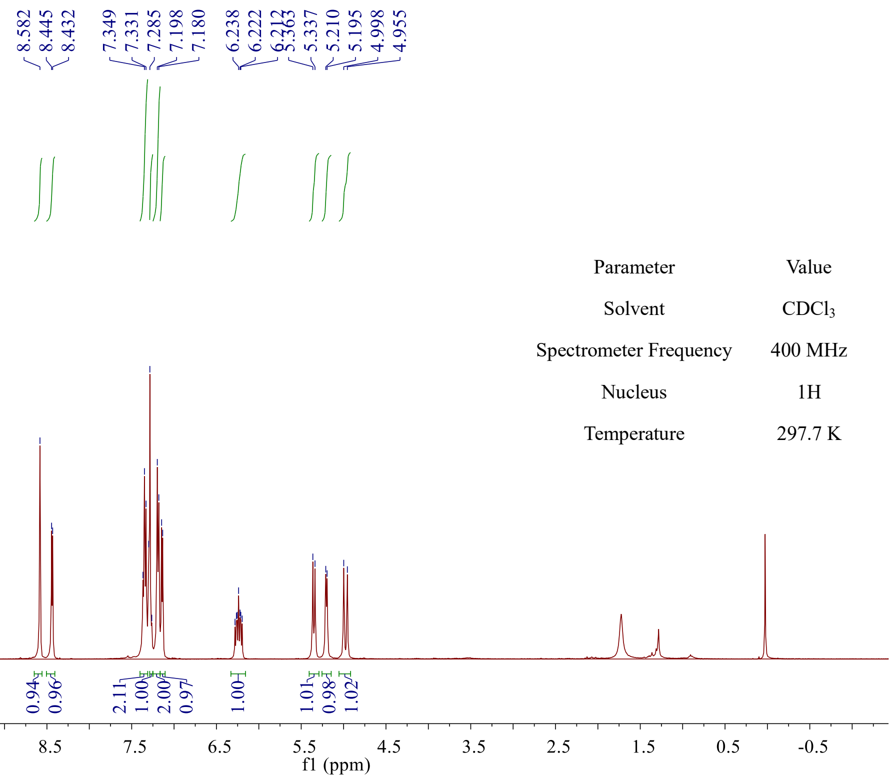

Parameter	Value
Solvent	$CDCl_3$
Spectrometer Frequency	400 MHz
Nucleus	1H
Temperature	297.7 K

^{1}H NMR Spectrum of Compound **3ba**

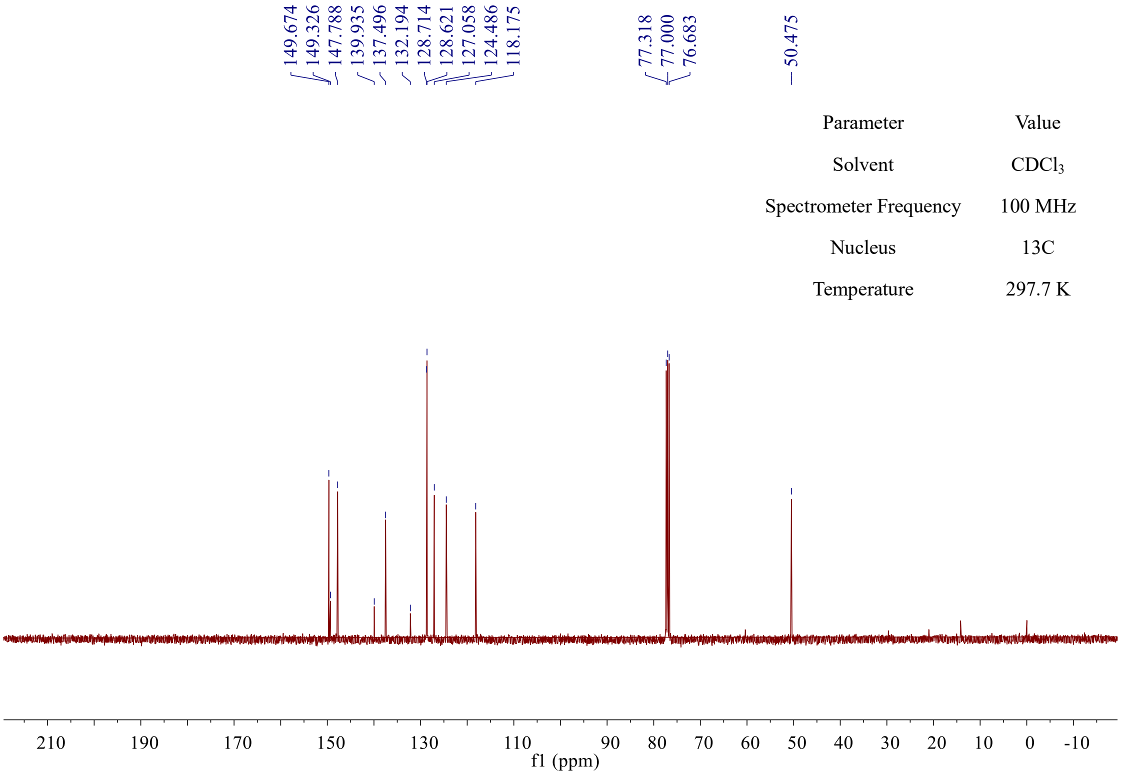

Parameter	Value
Solvent	$CDCl_3$
Spectrometer Frequency	100 MHz
Nucleus	13C
Temperature	297.7 K

^{13}C NMR Spectrum of Compound **3ba**

Supplementary Figure 138**. NMR spectra of 3ba**

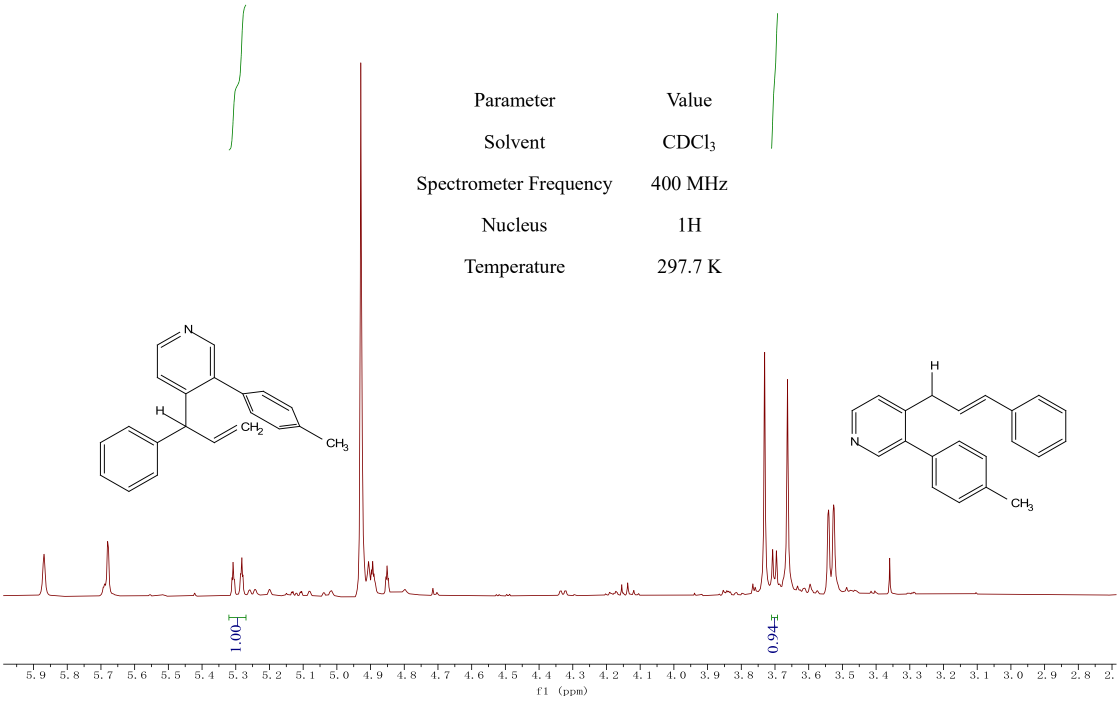

^{1}H NMR Spectrum of Crude Product **3bb**

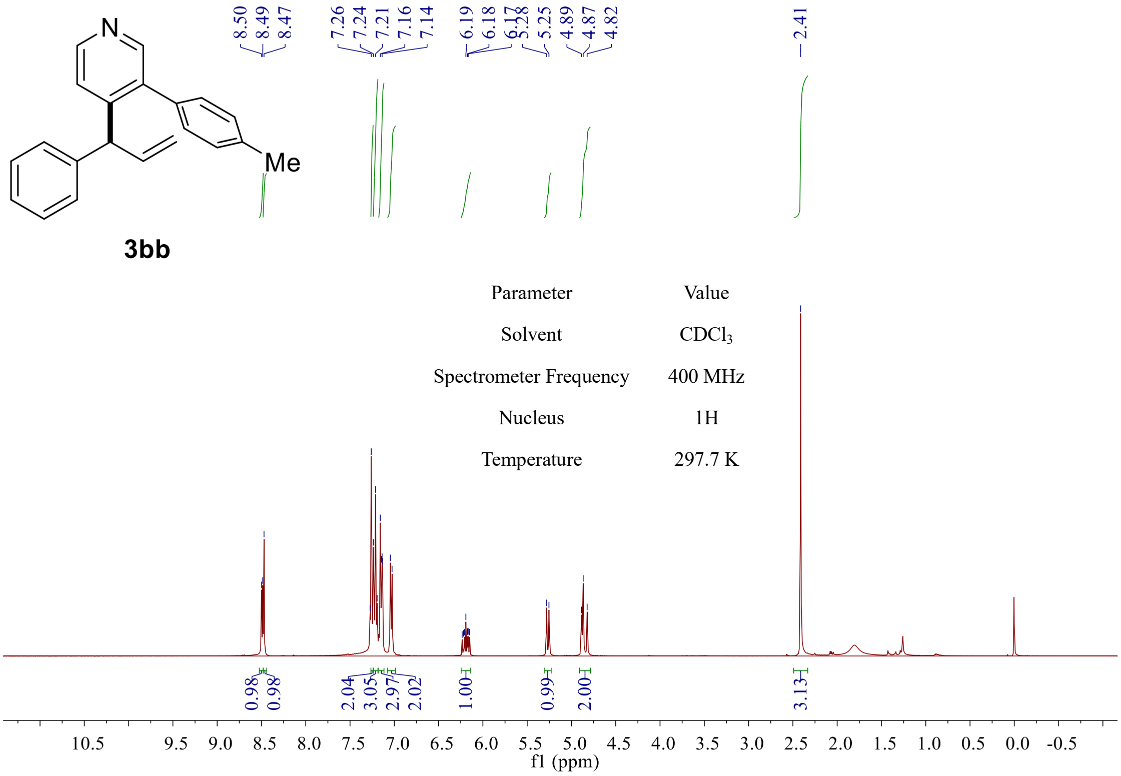

^{1}H NMR Spectrum of Compound **3bb**

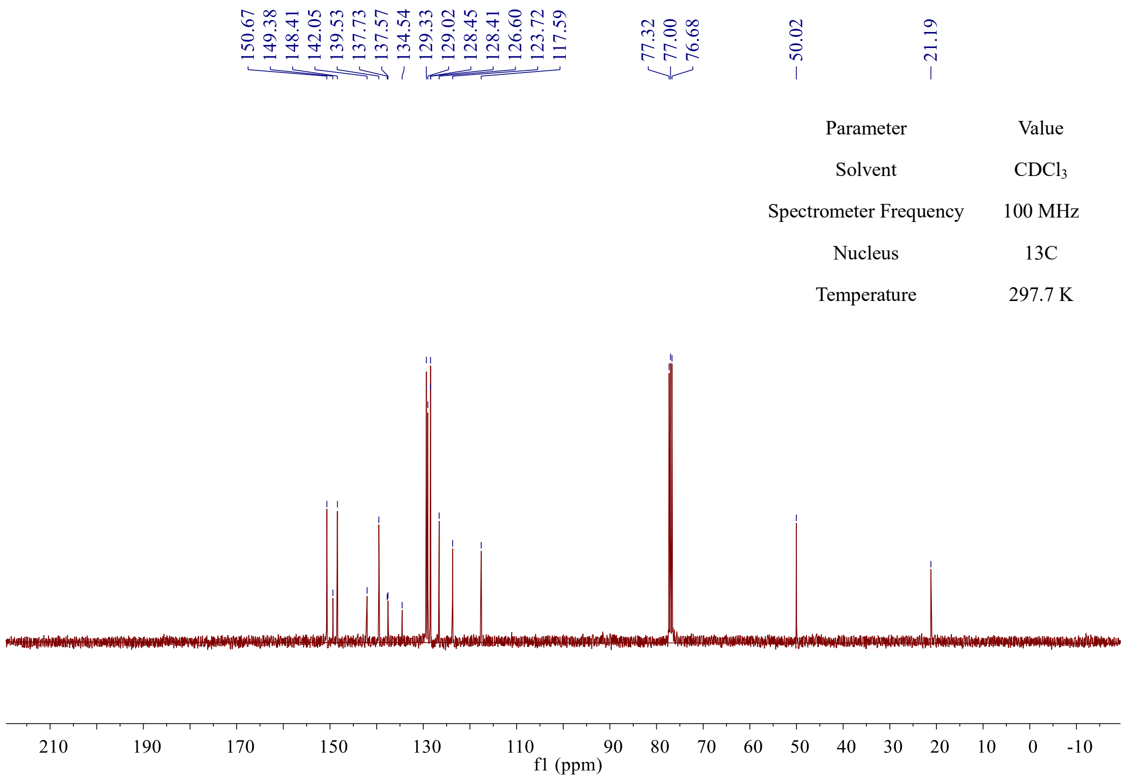

^{13}C NMR Spectrum of Compound **3bb**

Supplementary Figure 139**. NMR spectra of 3bb**

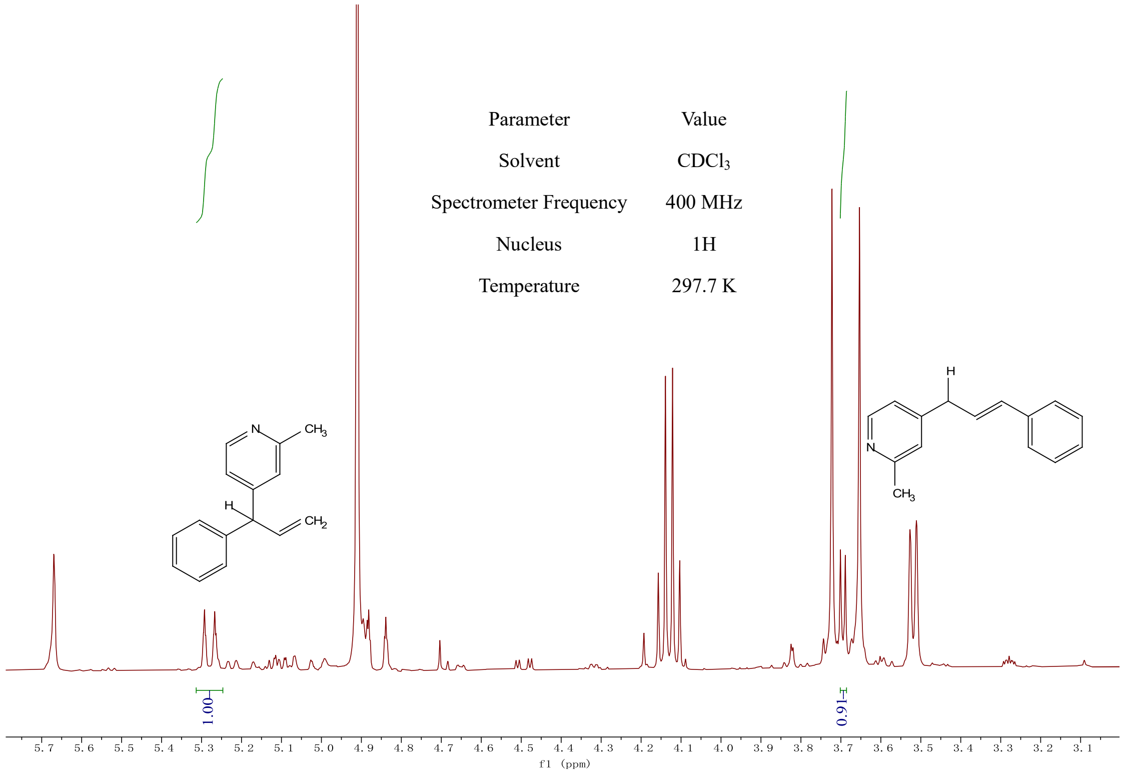

^{1}H NMR Spectrum of Crude Product **3bc**

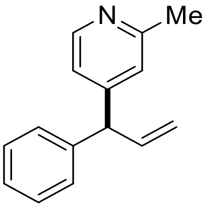

3bc

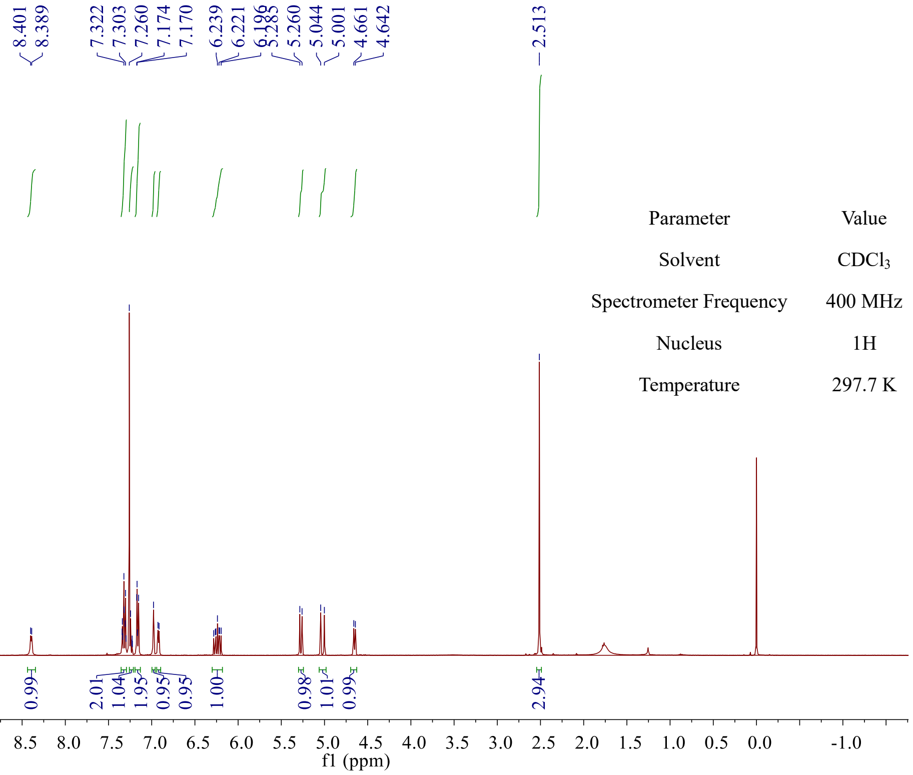

^{1}H NMR Spectrum of Compound **3bc**

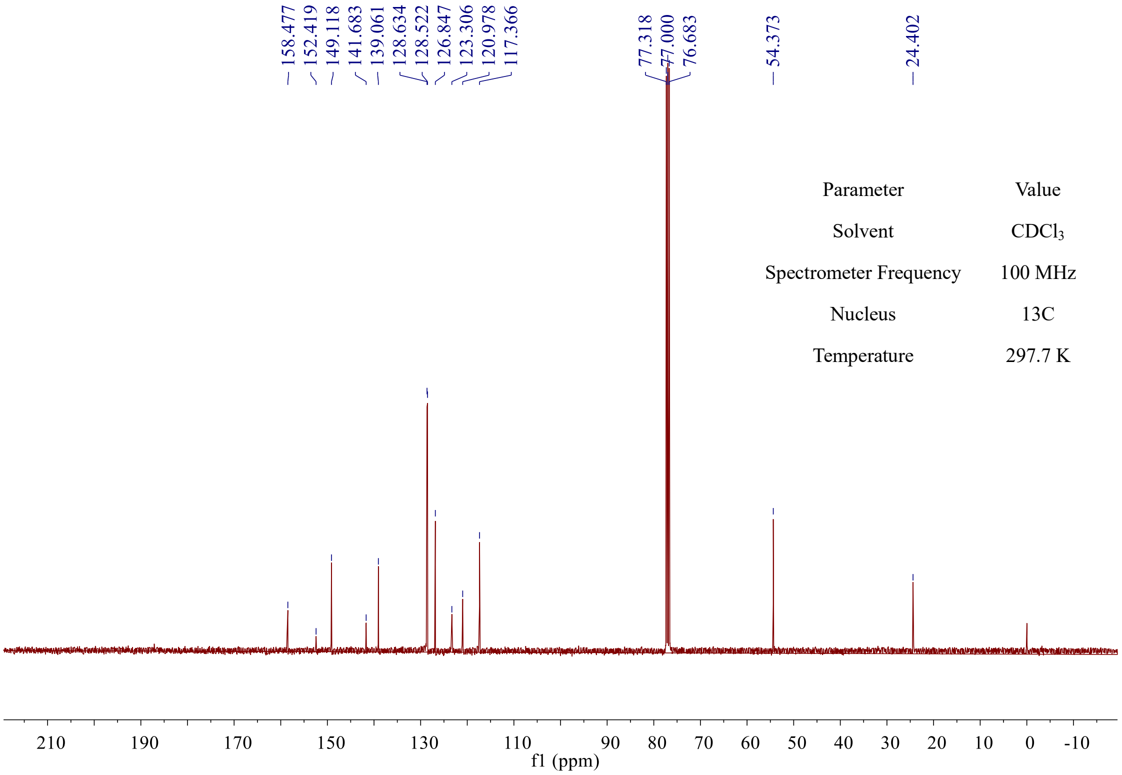

^{13}C NMR Spectrum of Compound **3bc**

Supplementary Figure 140. **NMR spectra of 3bc**

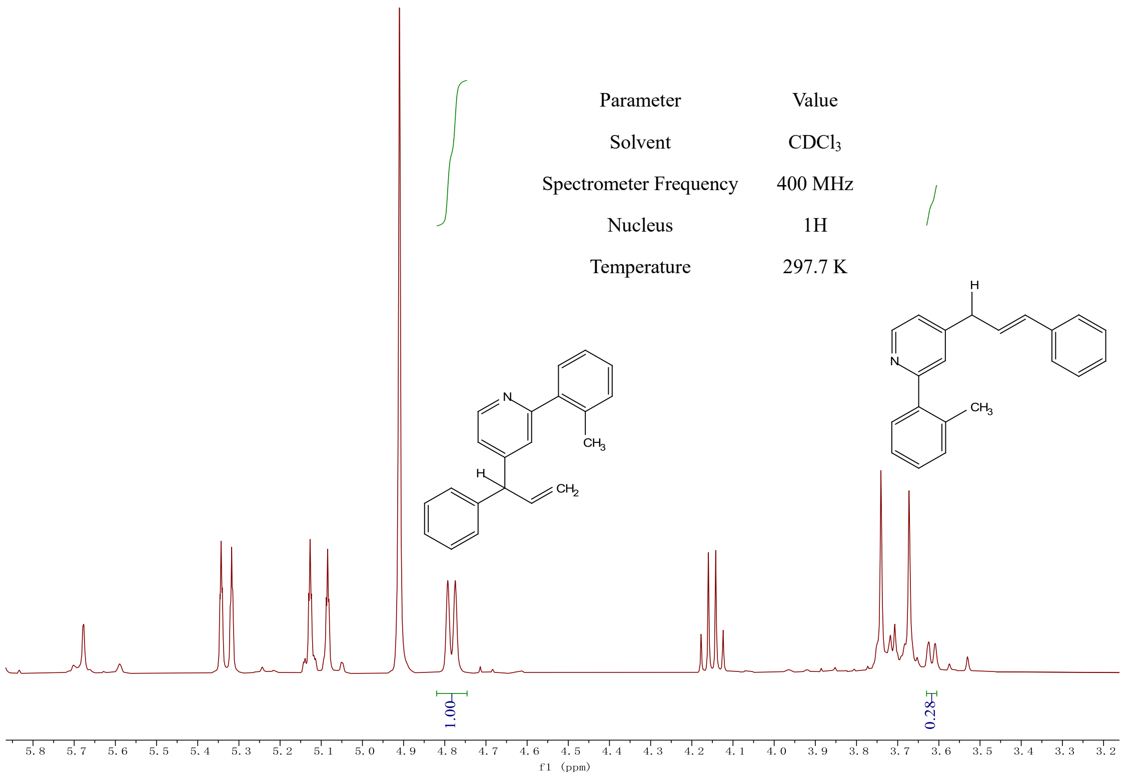

^{1}H NMR Spectrum of Crude Product **3bd**

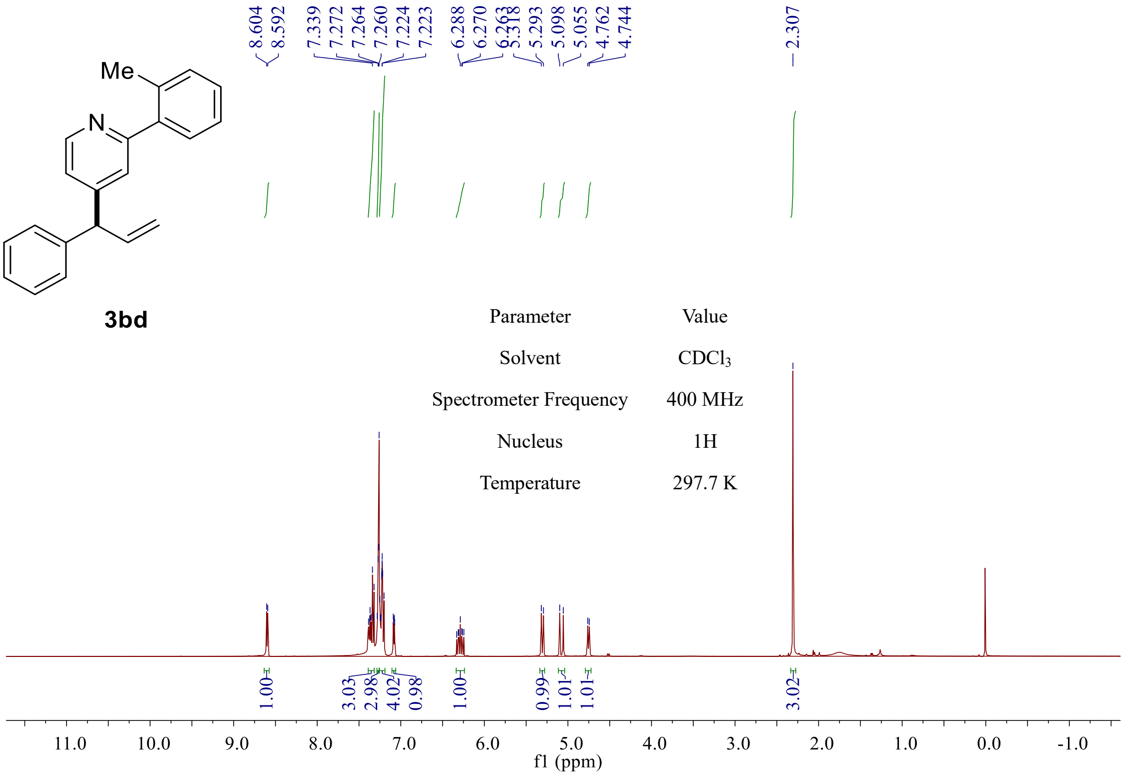

^{1}H NMR Spectrum of Compound **3bd**

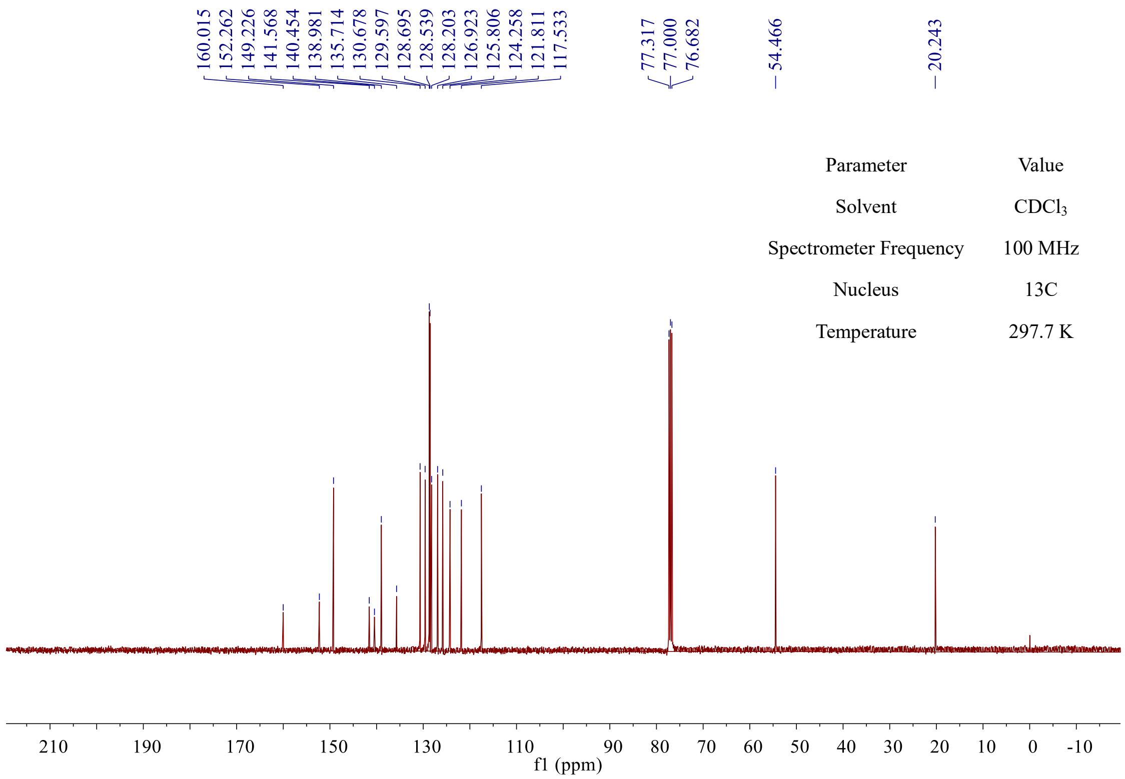

^{13}C NMR Spectrum of Compound **3bd**

Supplementary Figure 141. **NMR spectra of 3bd**

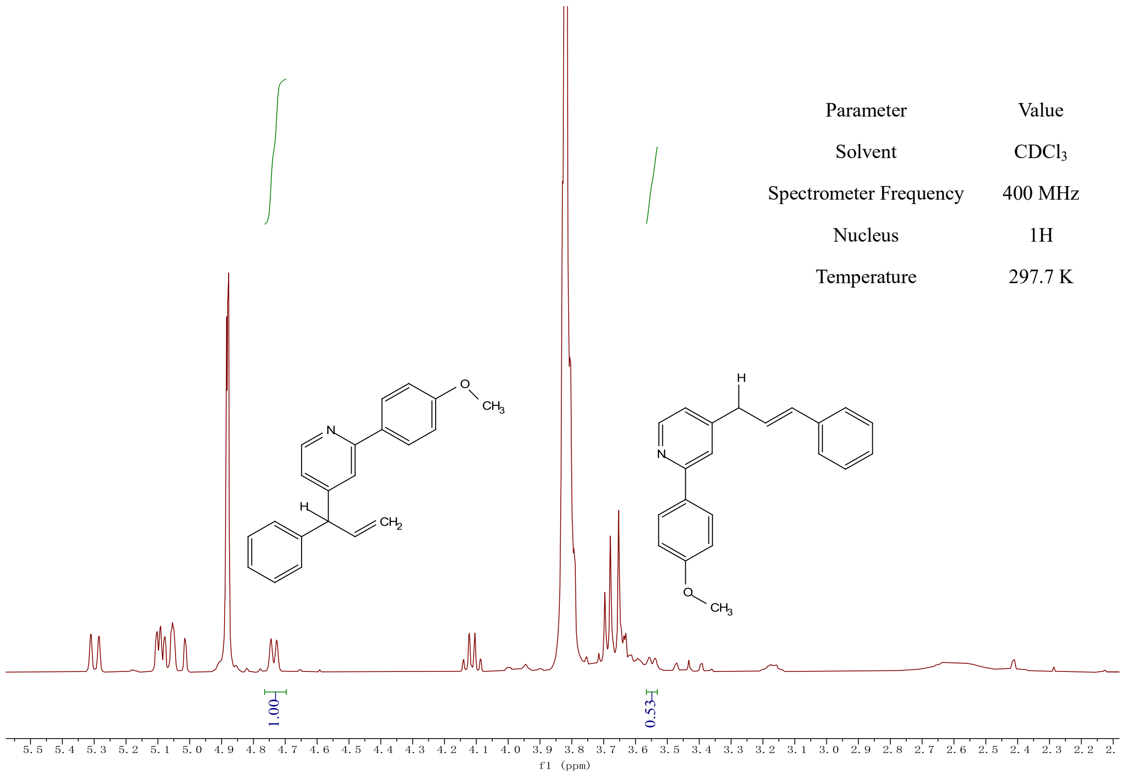

^{1}H NMR Spectrum of Crude Product **3be**

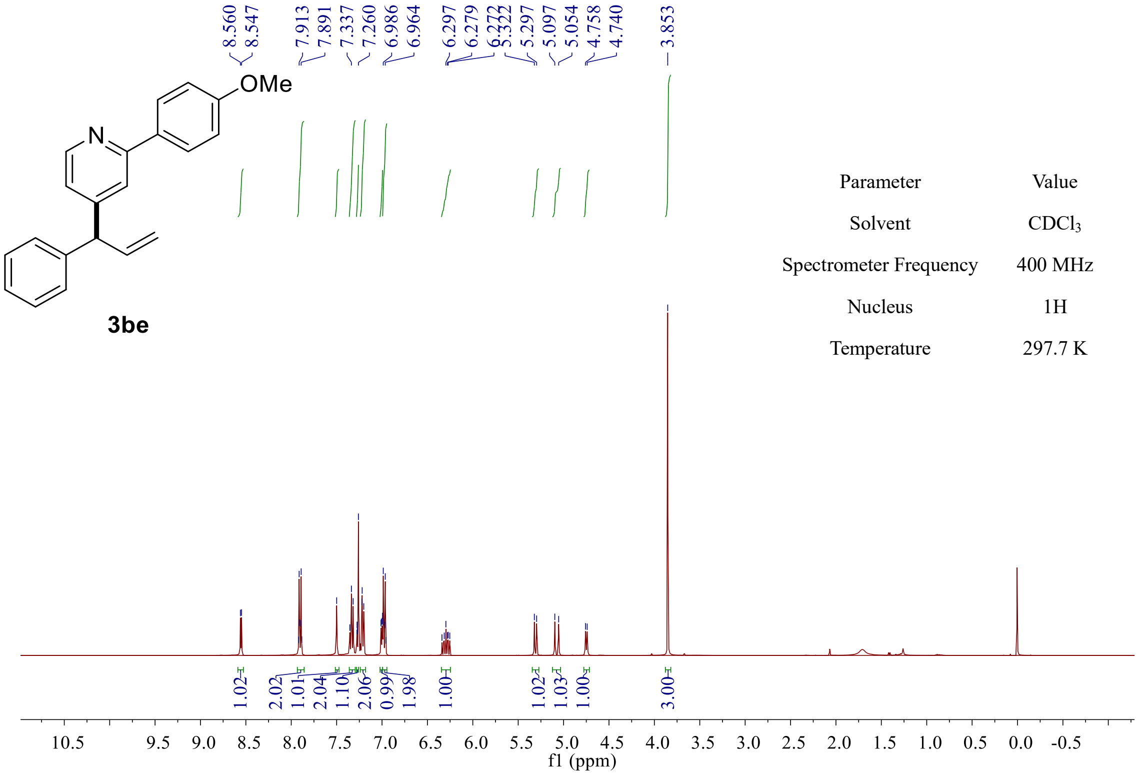

^{1}H NMR Spectrum of Compound **3be**

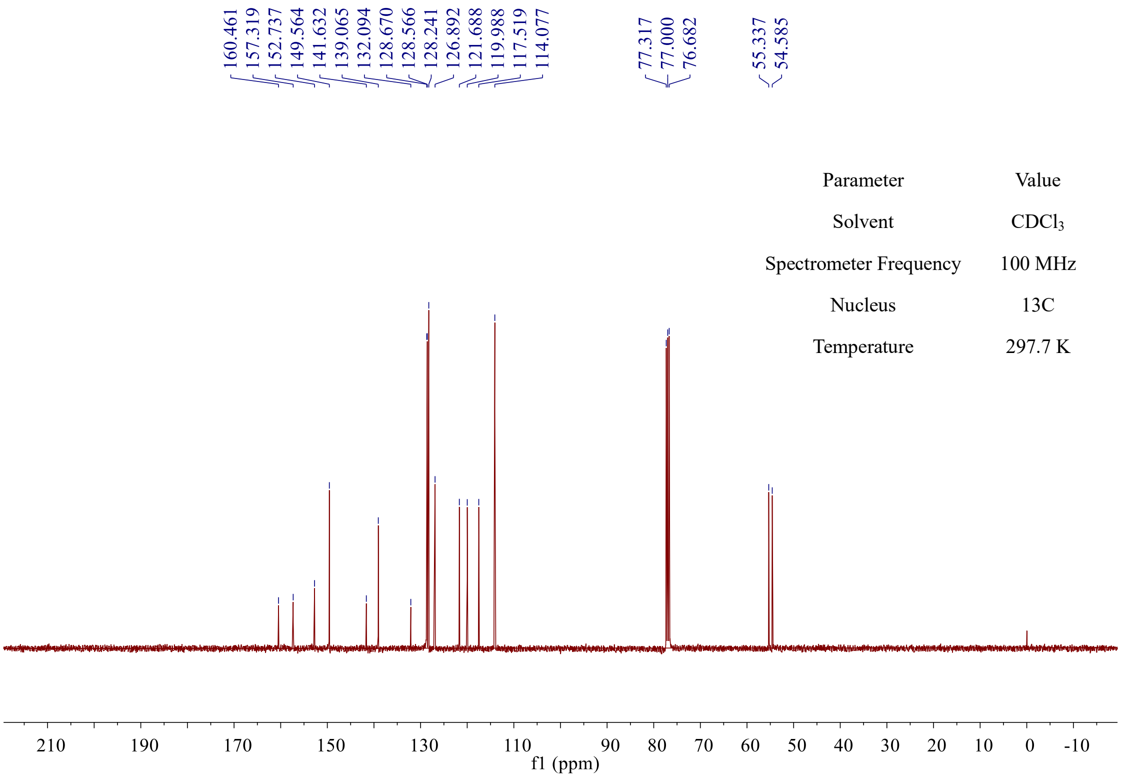

^{13}C NMR Spectrum of Compound **3be**

Supplementary Figure 142**. NMR spectra of 3be**

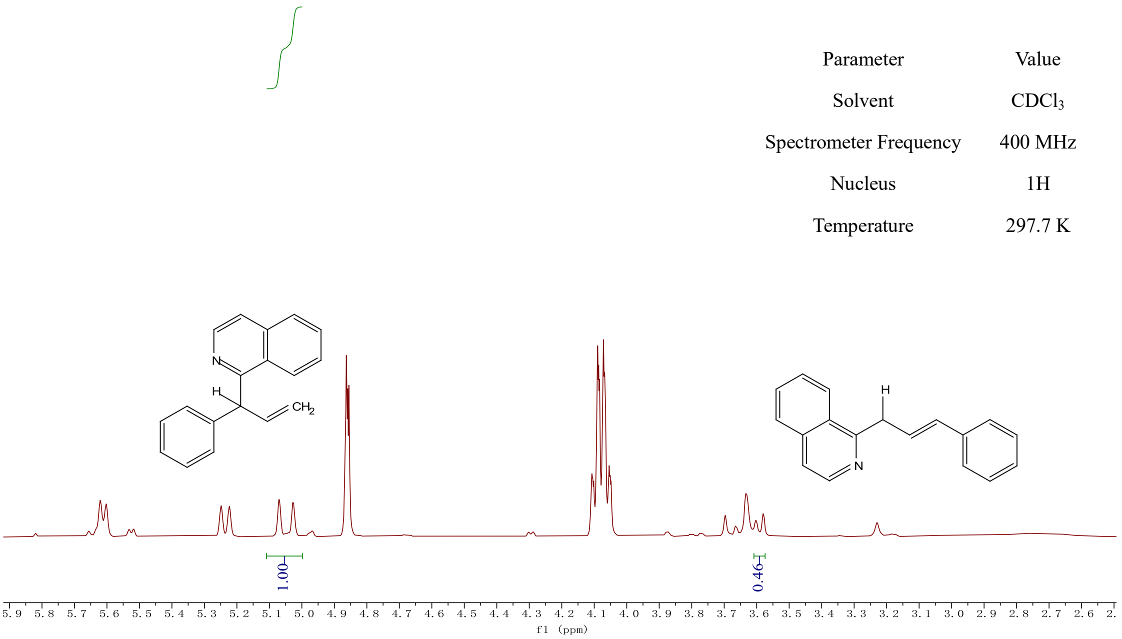

Parameter	Value
Solvent	$CDCl_3$
Spectrometer Frequency	400 MHz
Nucleus	1H
Temperature	297.7 K

^{1}H NMR Spectrum of Crude Product **3bf**

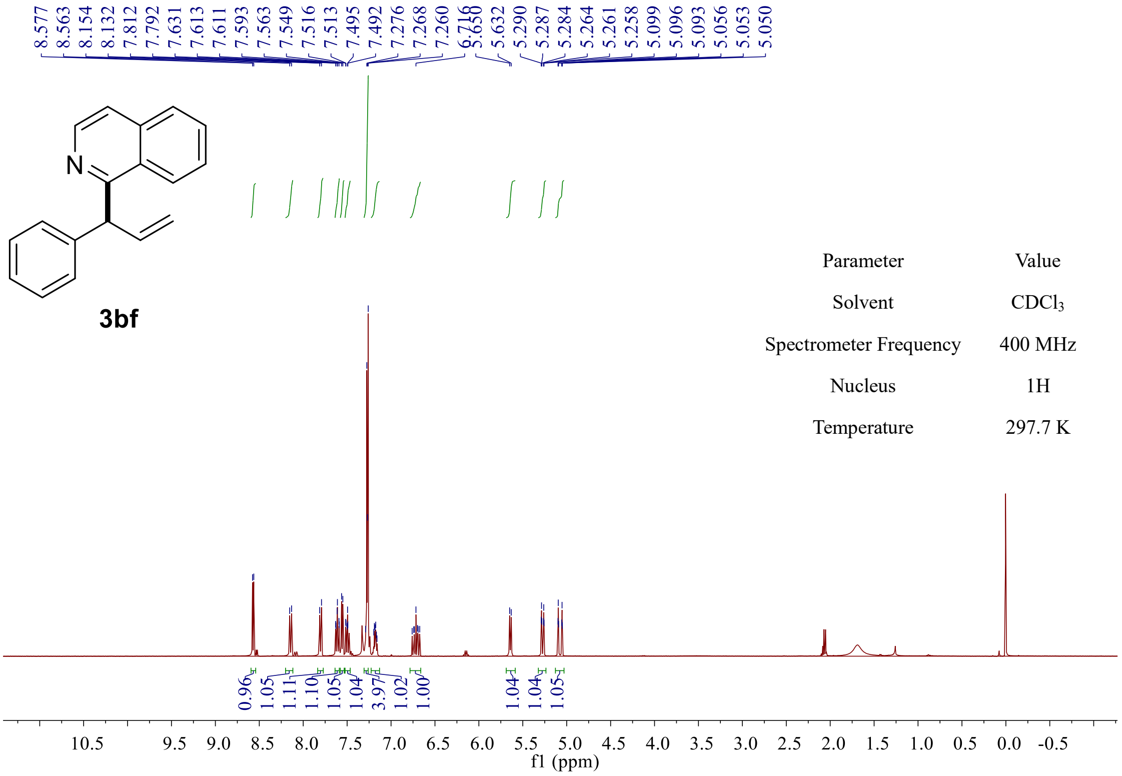

Parameter	Value
Solvent	$CDCl_3$
Spectrometer Frequency	400 MHz
Nucleus	1H
Temperature	297.7 K

^{1}H NMR Spectrum of Compound **3bf**

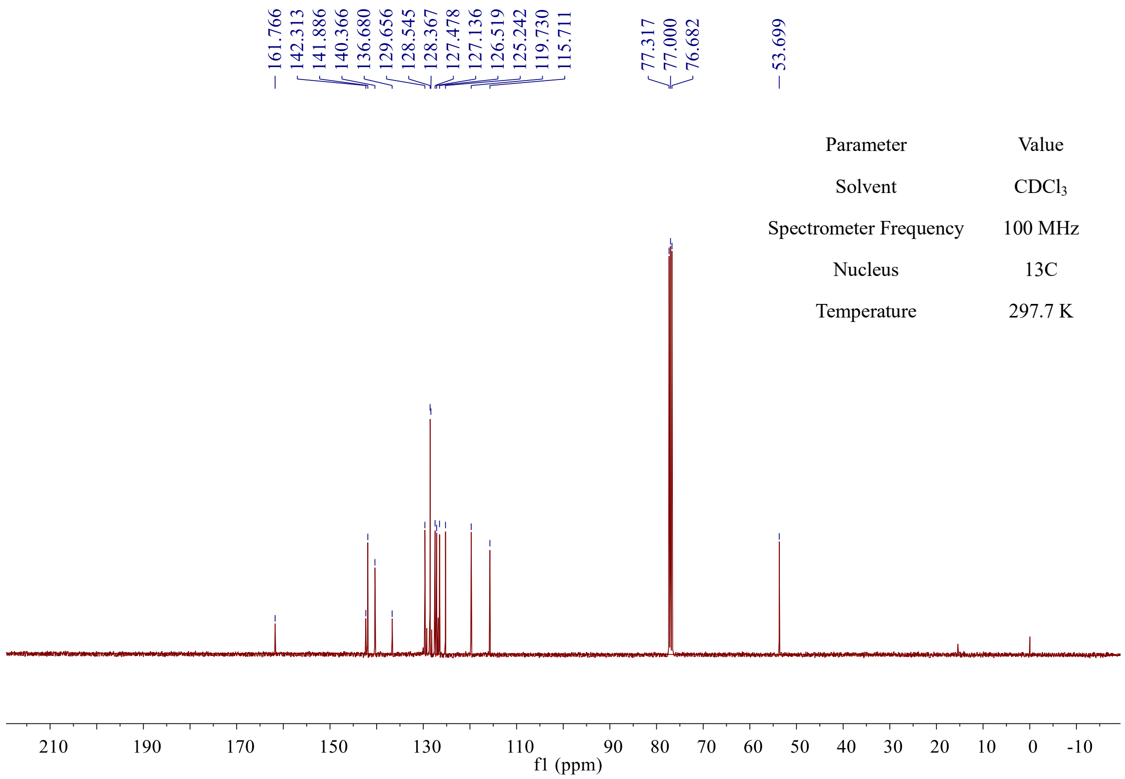

^{13}C NMR Spectrum of Compound **3bf**

Supplementary Figure 143**. NMR spectra of 3bf**

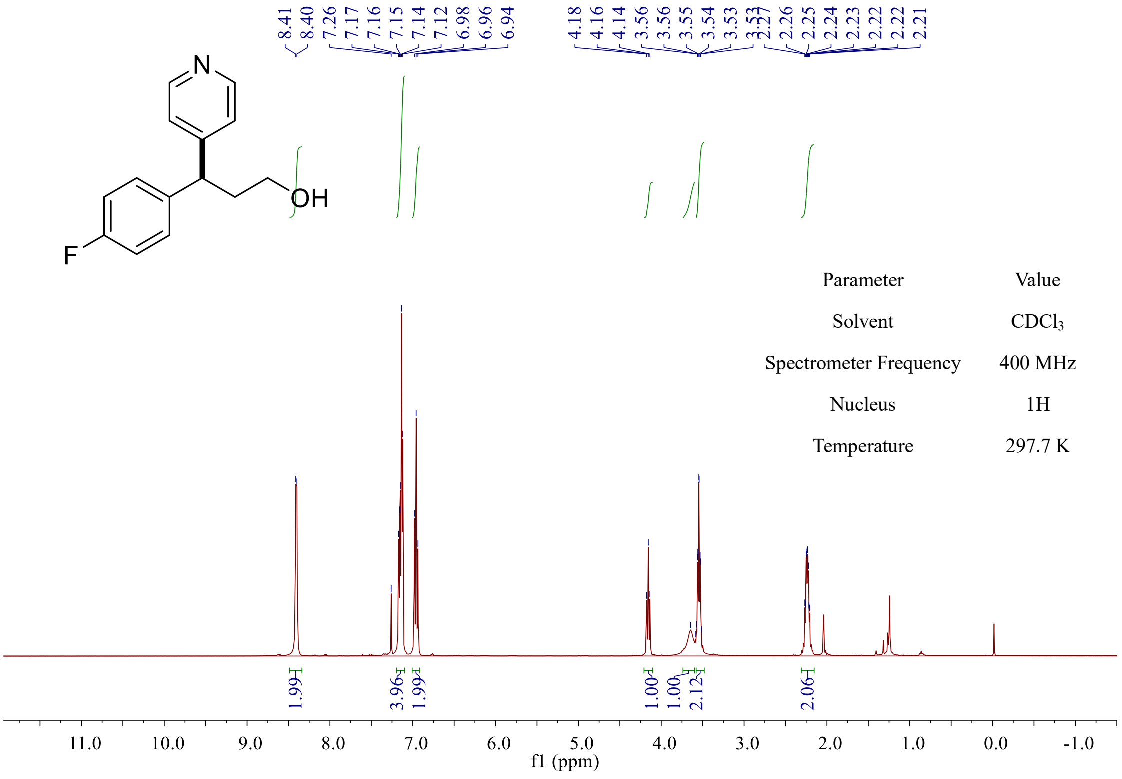

^{1}H NMR Spectrum of Compound **S1**

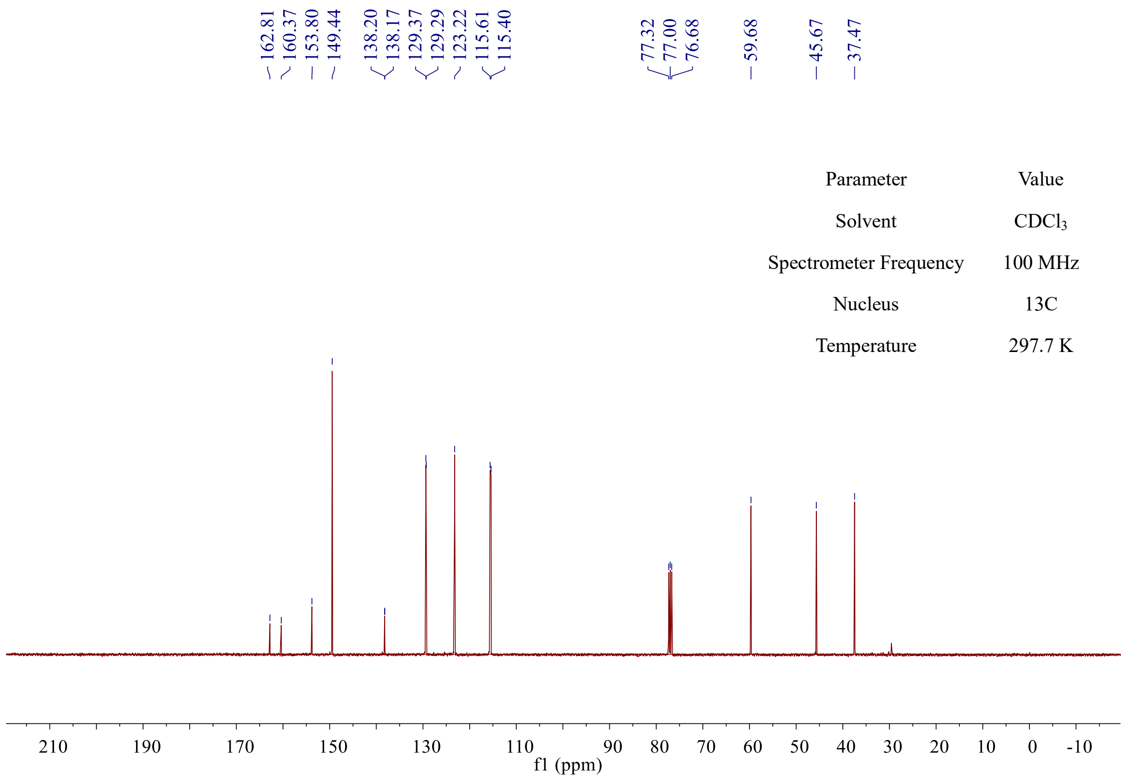

Parameter	Value
Solvent	$CDCl_3$
Spectrometer Frequency	100 MHz
Nucleus	13C
Temperature	297.7 K

^{13}C NMR Spectrum of Compound **S1**

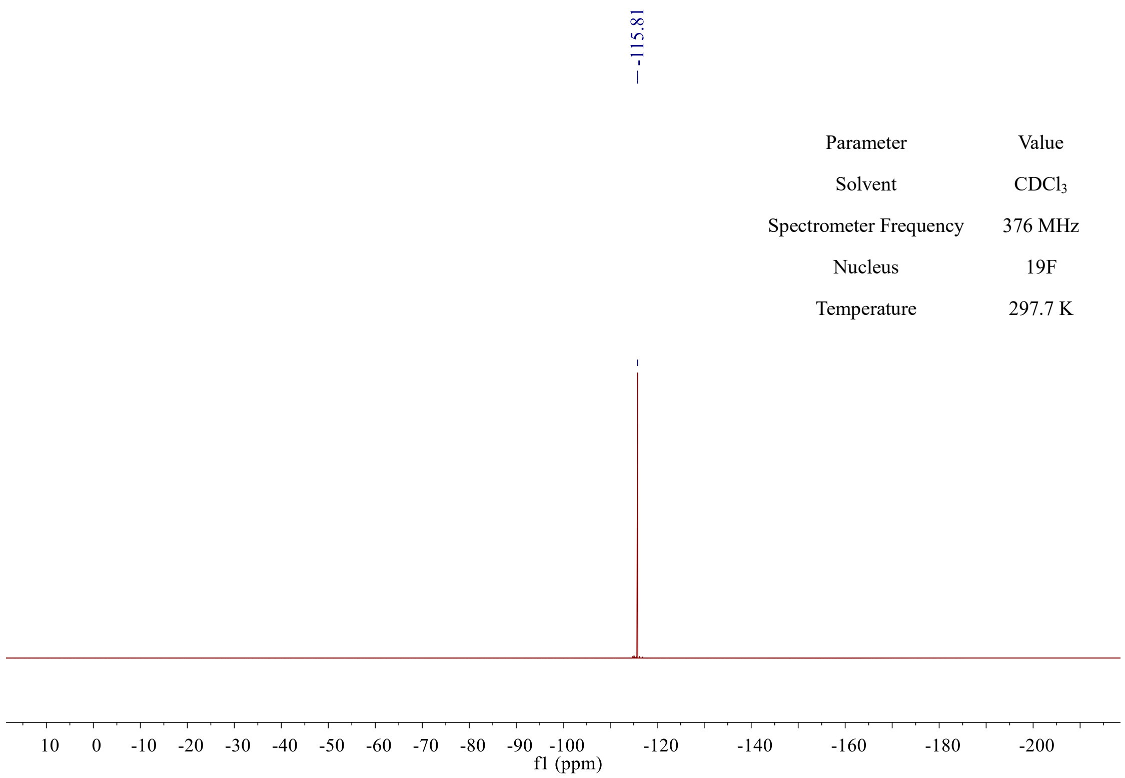

Parameter	Value
Solvent	$CDCl_3$
Spectrometer Frequency	376 MHz
Nucleus	19F
Temperature	297.7 K

^{19}F NMR Spectrum of Compound **S1**

Supplementary Figure 144**. NMR spectra of S1**

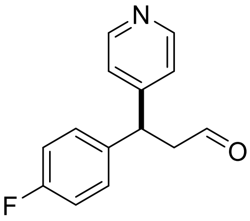

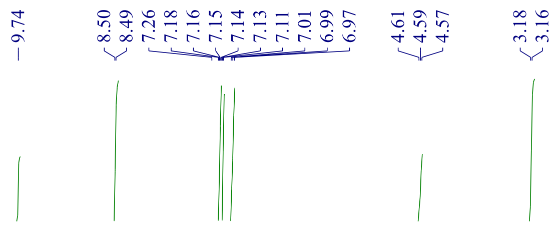

Parameter	Value
Solvent	$CDCl_3$
Spectrometer Frequency	400 MHz
Nucleus	1H
Temperature	297.7 K

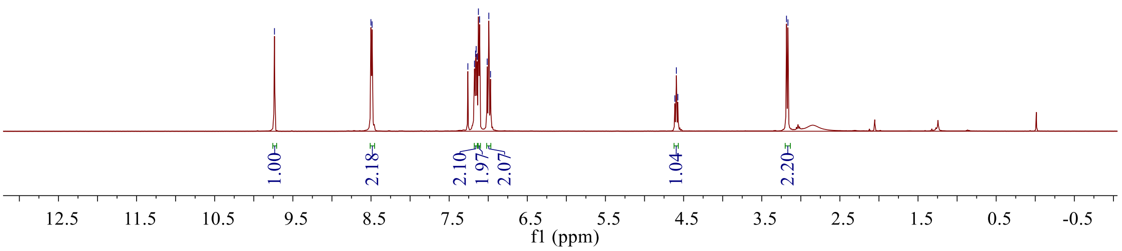

^{1}H NMR Spectrum of Compound **S2**

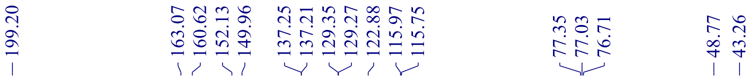

Parameter	Value
Solvent	$CDCl_3$
Spectrometer Frequency	100 MHz
Nucleus	13C
Temperature	297.7 K

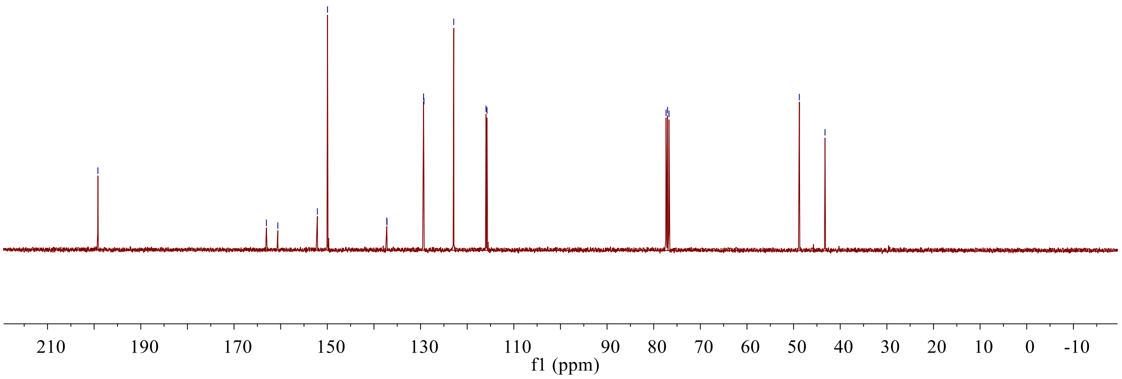

^{13}C NMR Spectrum of Compound **S2**

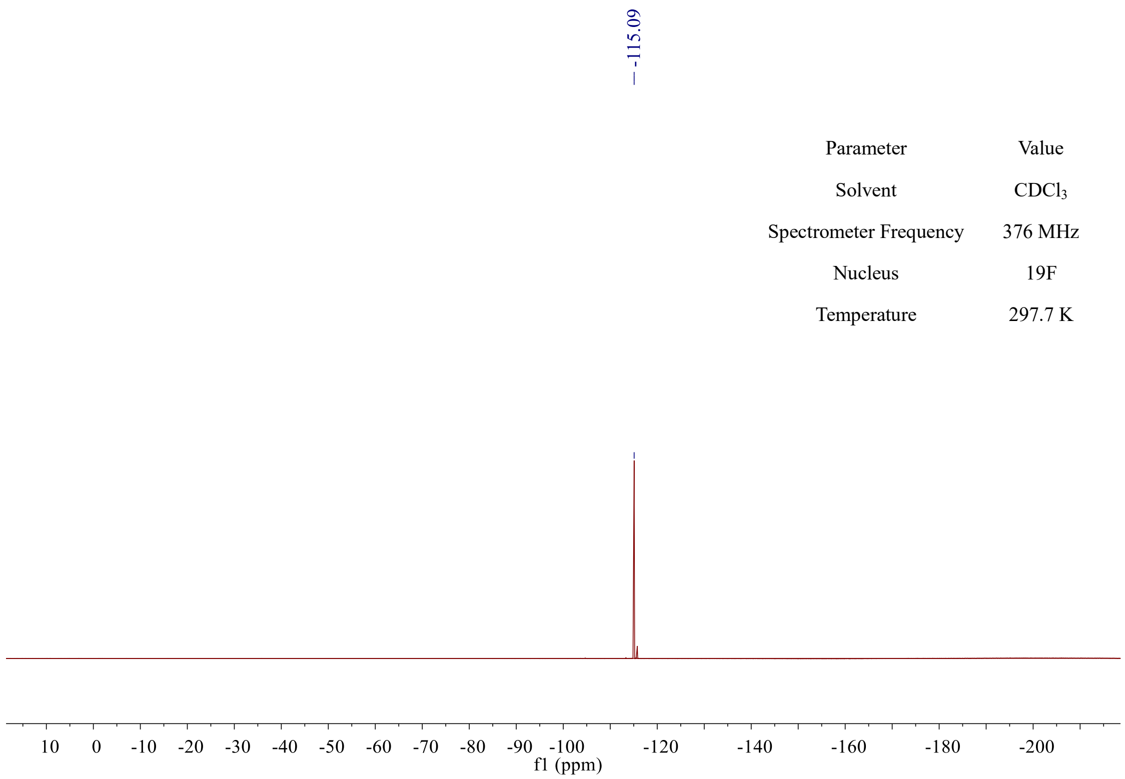

^{19}F NMR Spectrum of Compound **S2**

Supplementary Figure 145**. NMR spectra of S2**

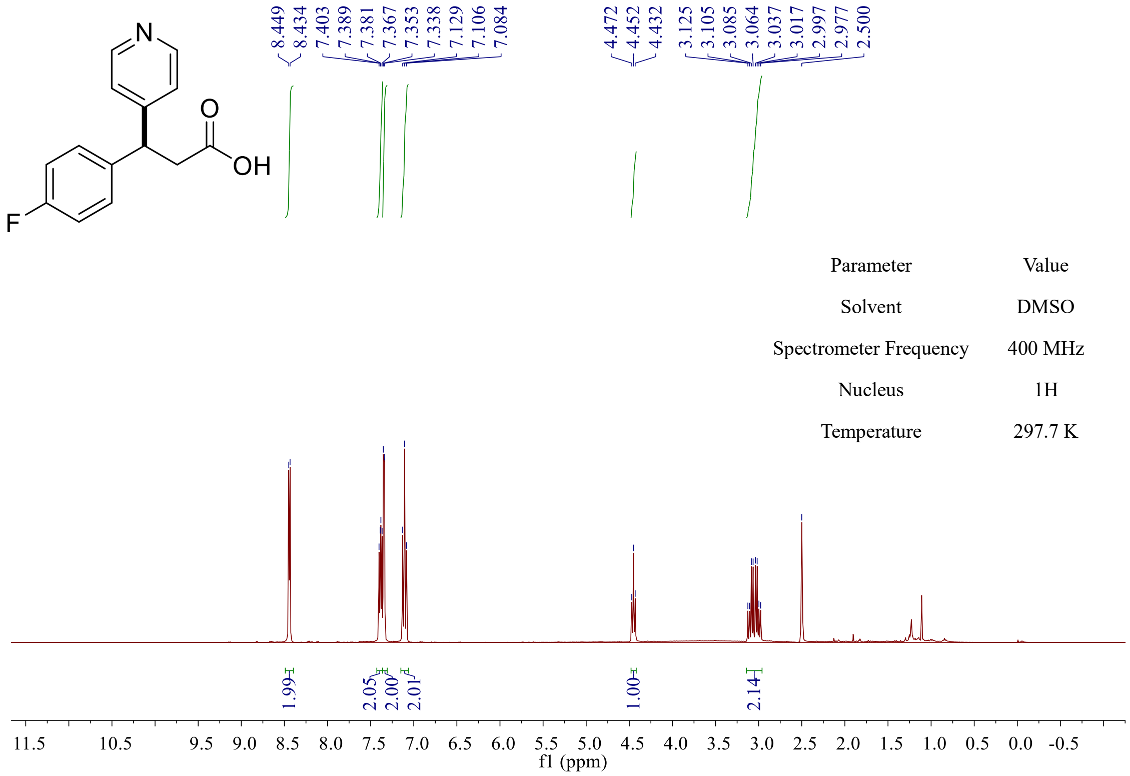

^{1}H NMR Spectrum of Compound **5**

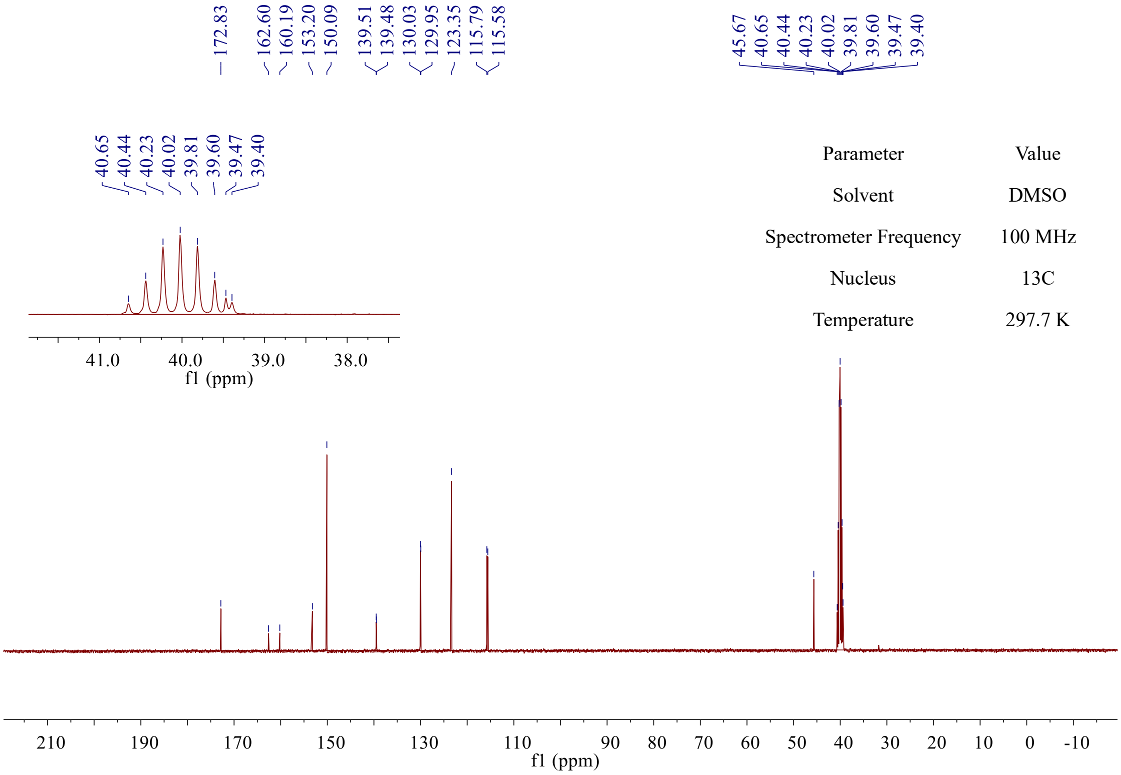

Parameter	Value
Solvent	DMSO
Spectrometer Frequency	100 MHz
Nucleus	13C
Temperature	297.7 K

^{13}C NMR Spectrum of Compound **5**

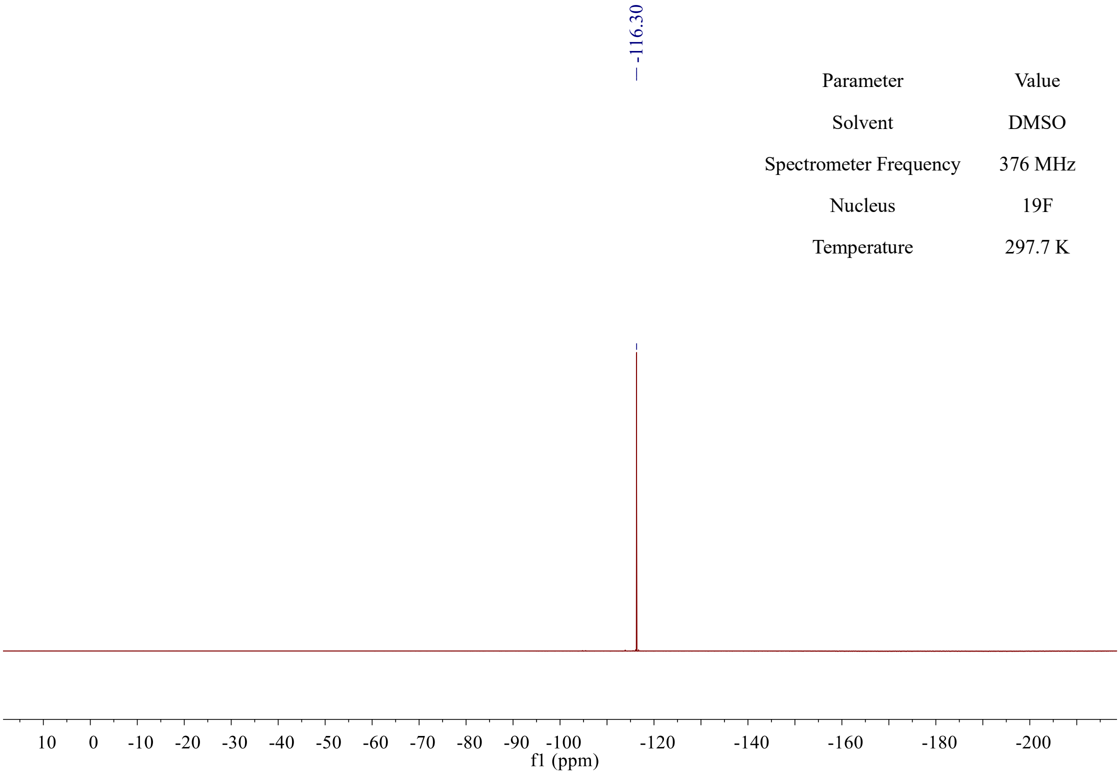

Parameter	Value
Solvent	DMSO
Spectrometer Frequency	376 MHz
Nucleus	19F
Temperature	297.7 K

^{19}F NMR Spectrum of Compound **5**

Supplementary Figure 146**. NMR spectra of 5**

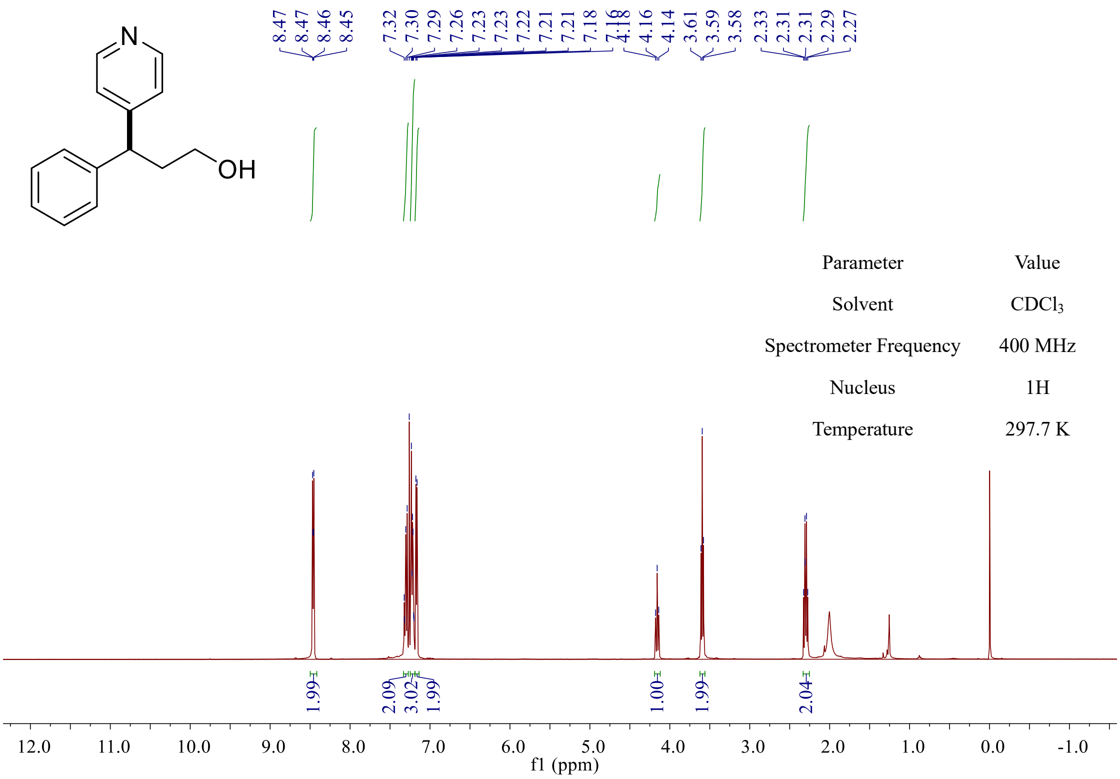

^{1}H NMR Spectrum of Compound **S3**

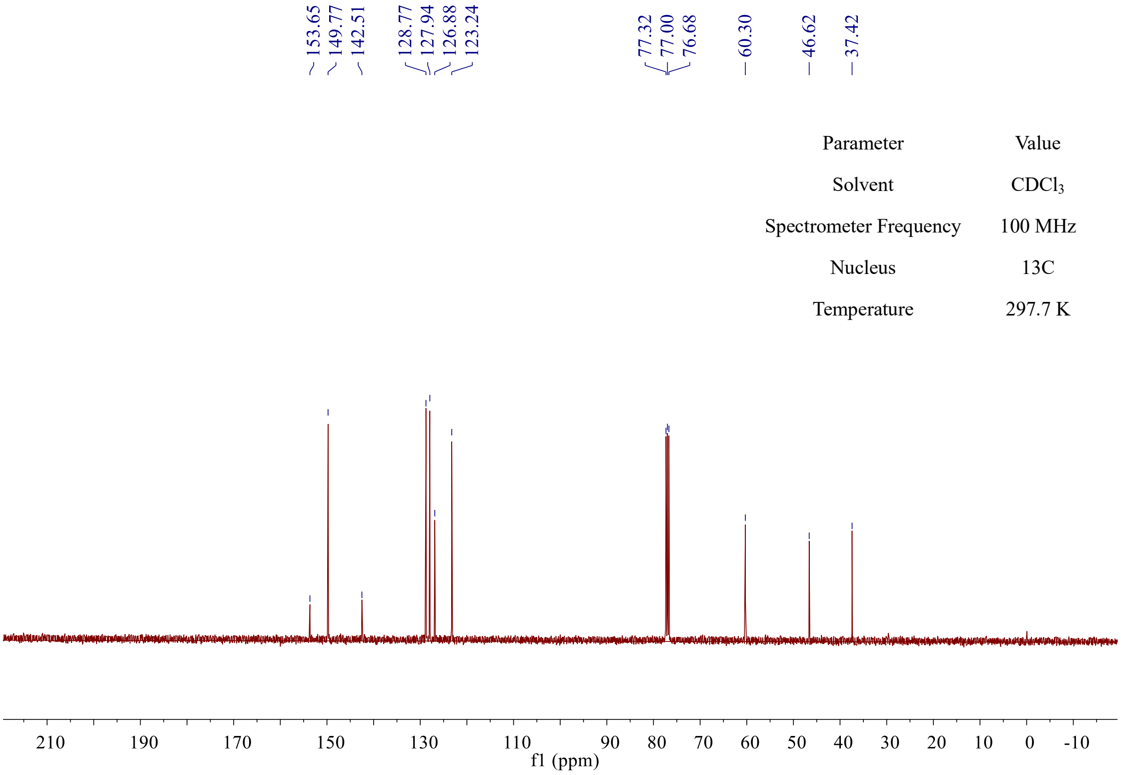

^{13}C NMR Spectrum of Compound **S3**

Supplementary Figure 147**. NMR spectra of S3**

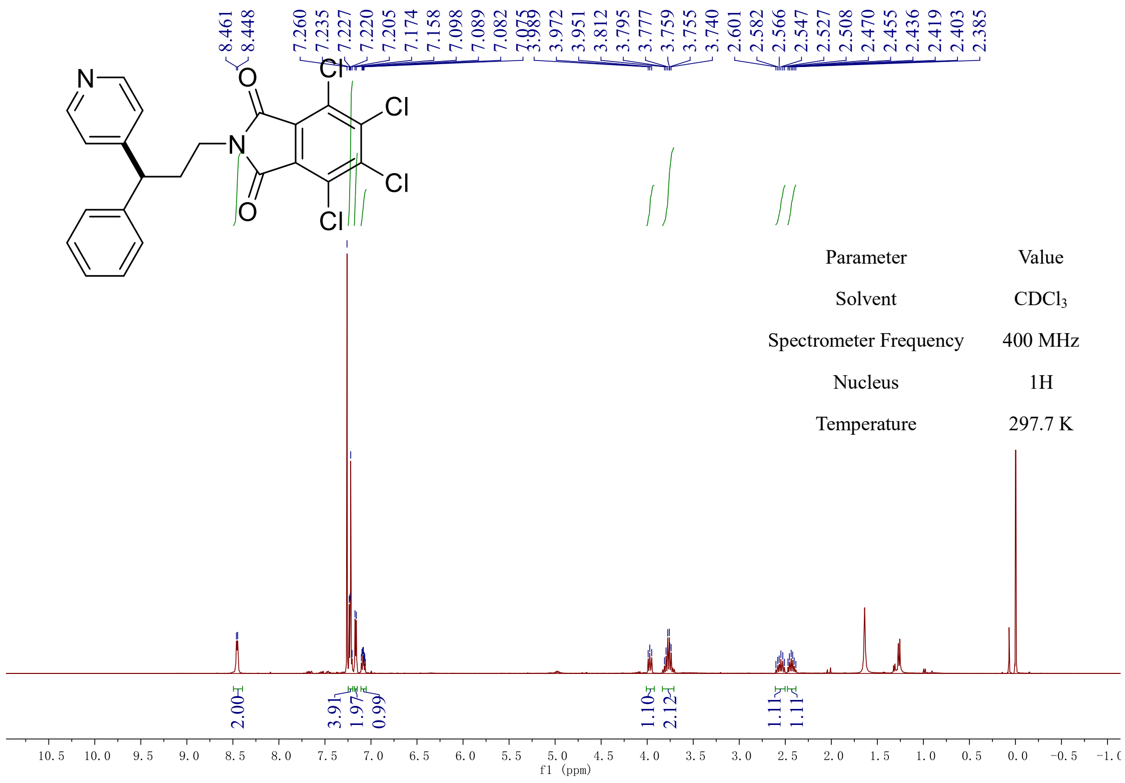

^{1}H NMR Spectrum of Compound **S4**

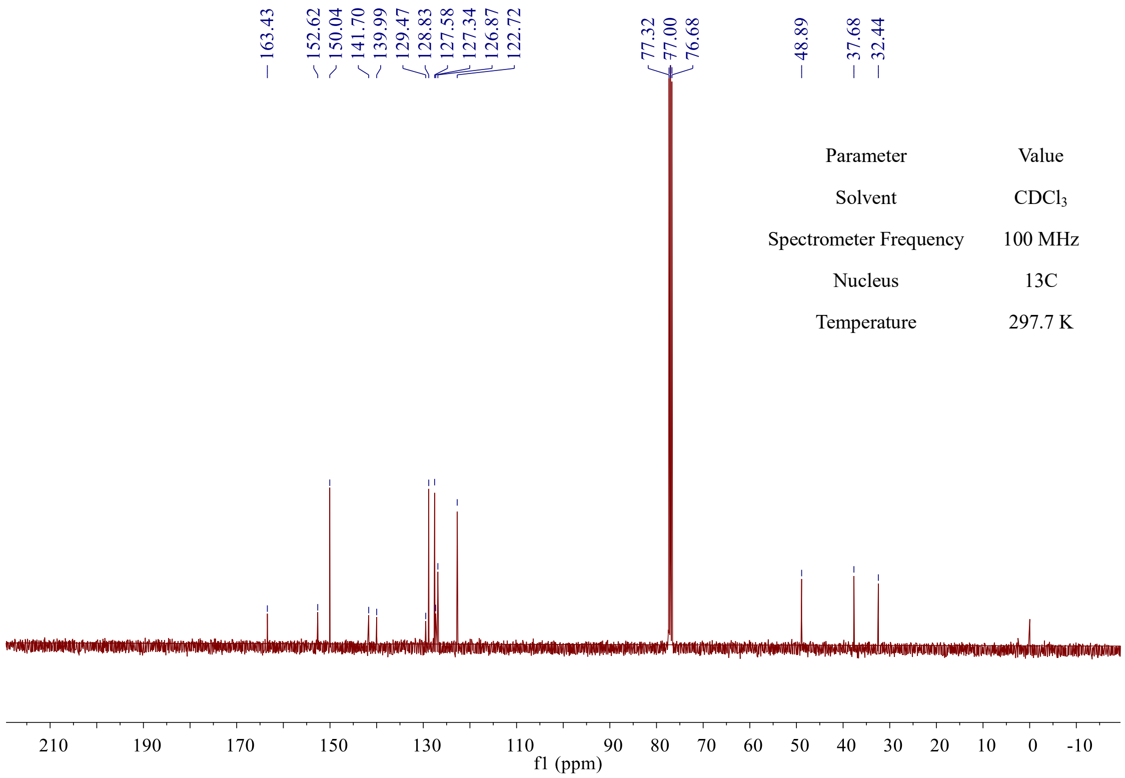

^{13}C NMR Spectrum of Compound **S4**

Supplementary Figure 148**. NMR spectra of S4**

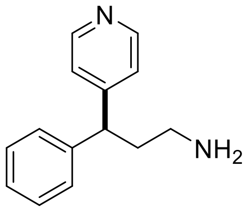

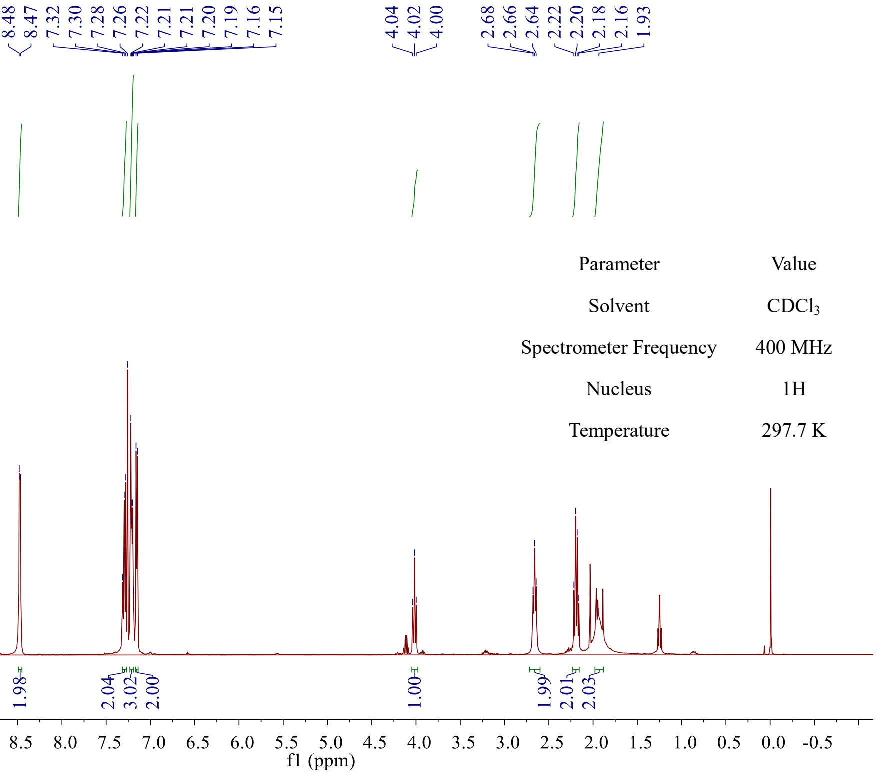

Parameter	Value
Solvent	$CDCl_3$
Spectrometer Frequency	400 MHz
Nucleus	1H
Temperature	297.7 K

^{1}H NMR Spectrum of Compound **6**

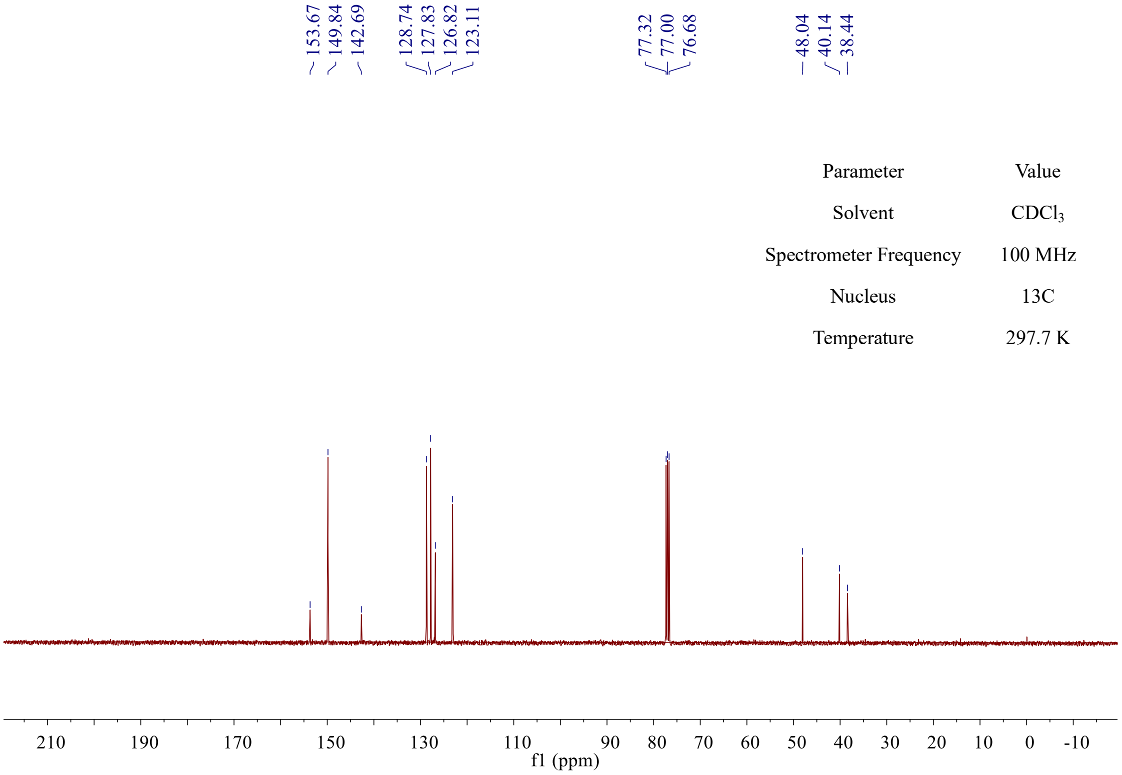

Parameter	Value
Solvent	$CDCl_3$
Spectrometer Frequency	100 MHz
Nucleus	13C
Temperature	297.7 K

^{13}C NMR Spectrum of Compound **6**

Supplementary Figure 149**. NMR spectra of 6**

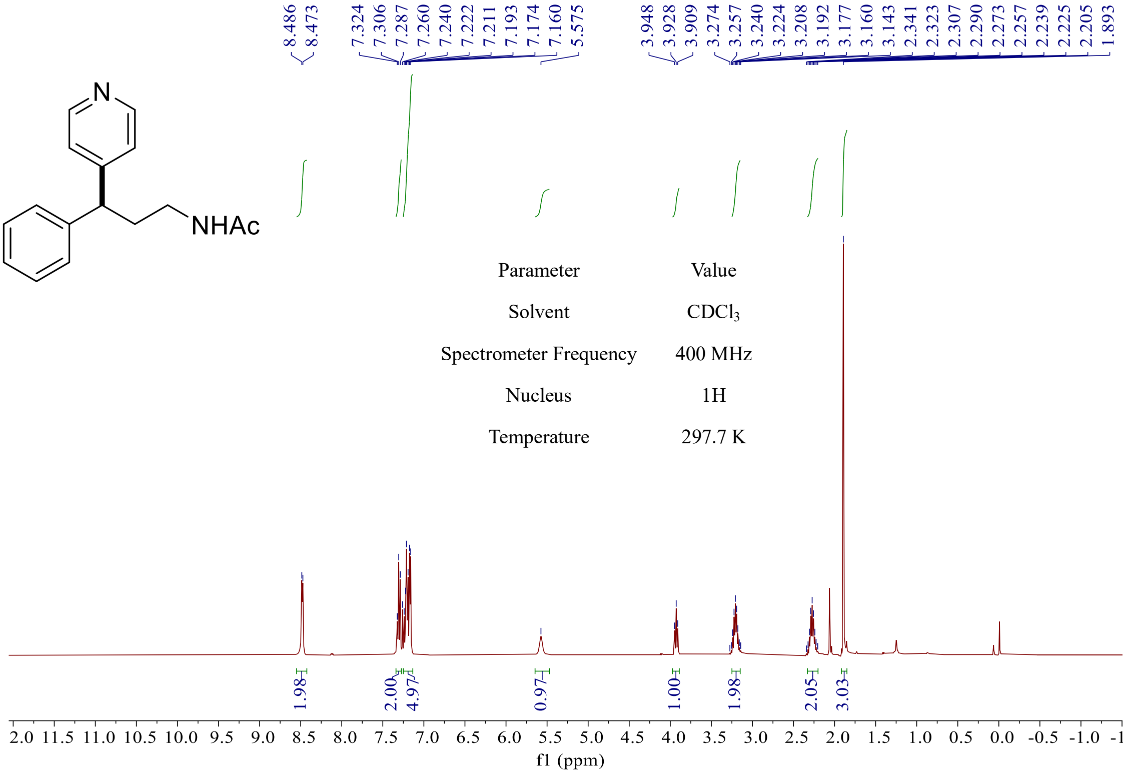

^{1}H NMR Spectrum of Compound **S5**

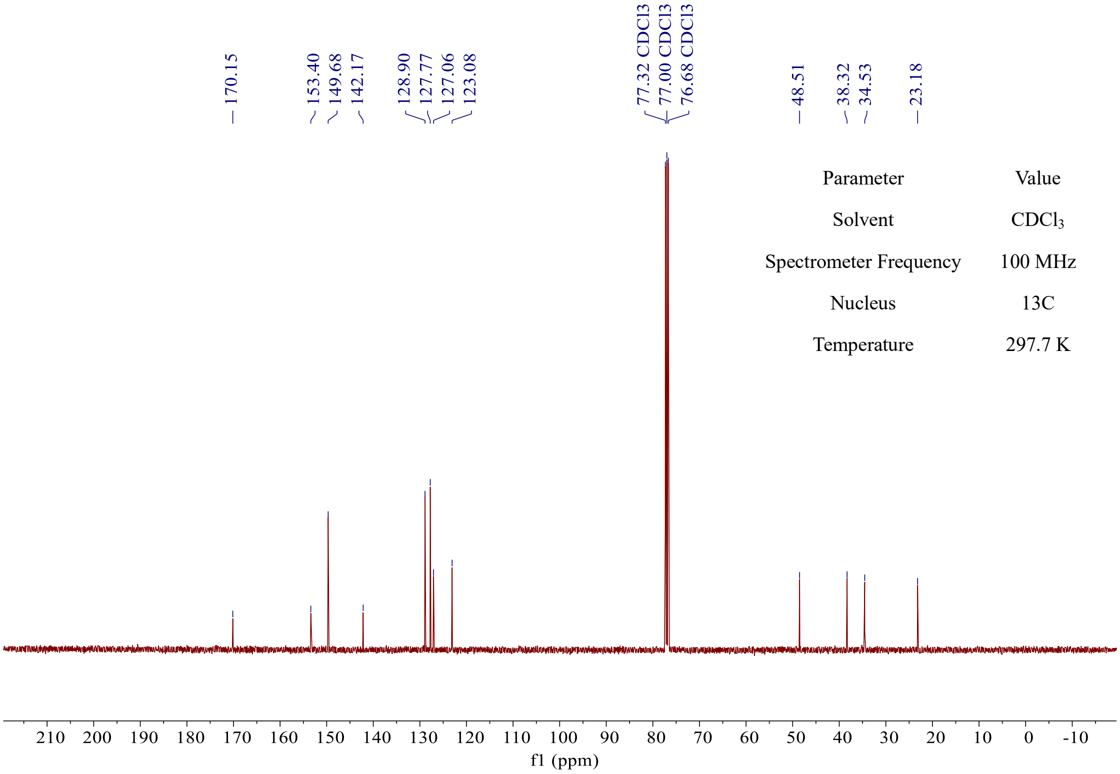

^{13}C NMR Spectrum of Compound **S5**

Supplementary Figure 150**. NMR spectra of S5**

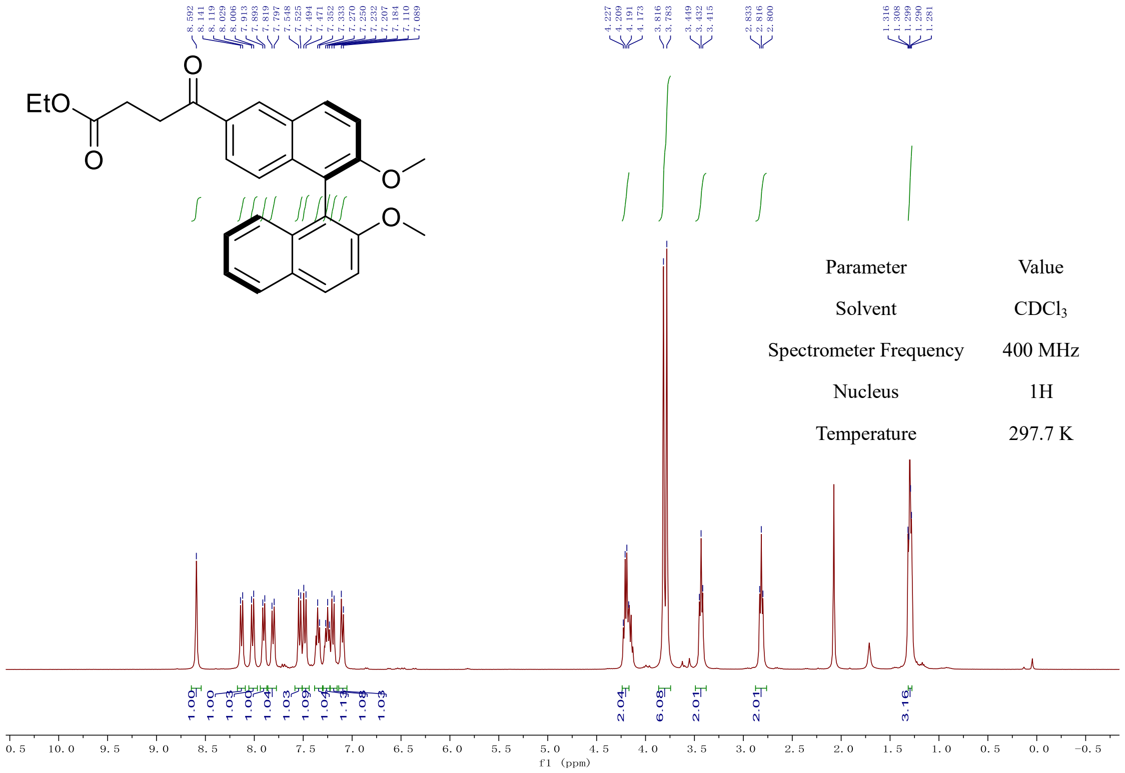

Parameter	Value
Solvent	$CDCl_3$
Spectrometer Frequency	400 MHz
Nucleus	1H
Temperature	297.7 K

Supplementary Figure 151. **^{1}H NMR Spectrum of Compound S8**

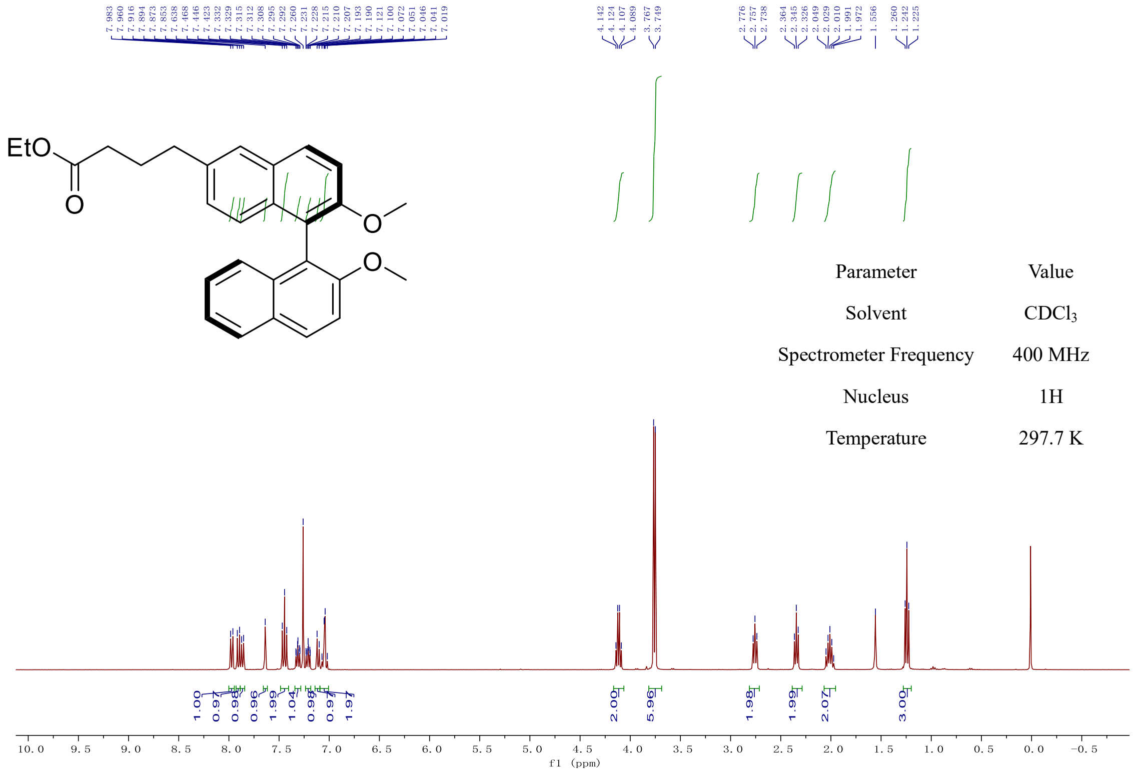

Parameter	Value
Solvent	$CDCl_3$
Spectrometer Frequency	400 MHz
Nucleus	1H
Temperature	297.7 K

Supplementary Figure 152. **^{1}H NMR Spectrum of Compound S9**

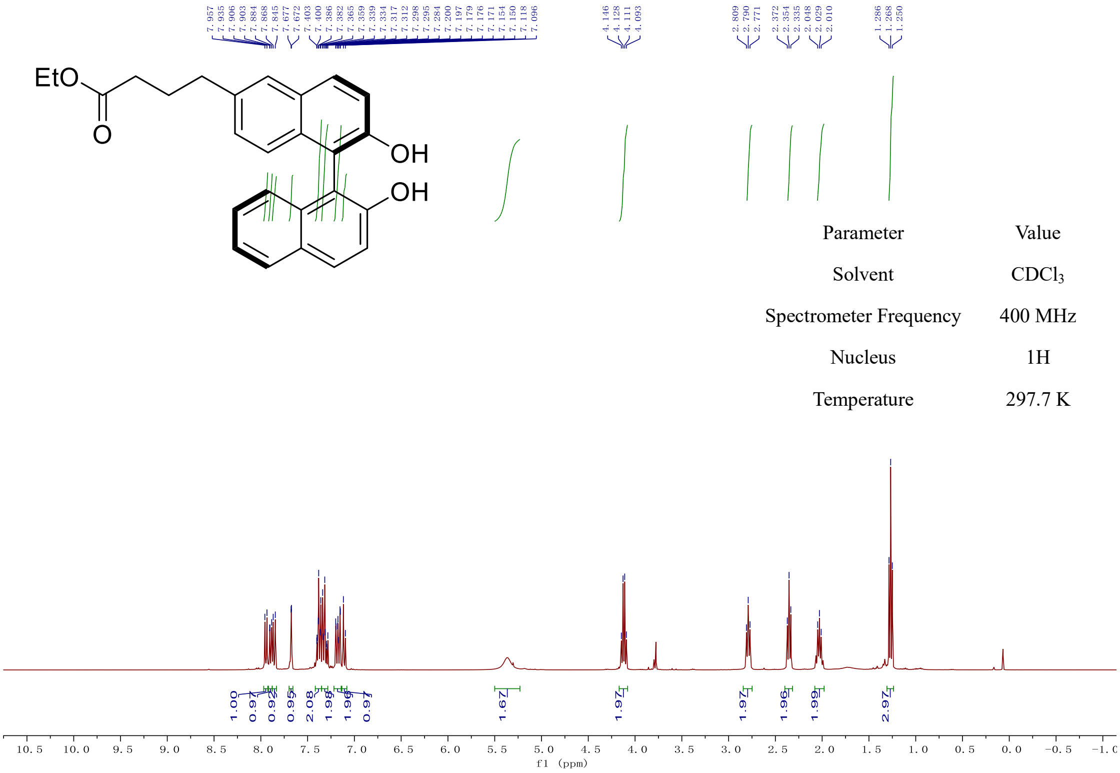

Supplementary Figure 153. **^{1}H NMR Spectrum of Compound S10**

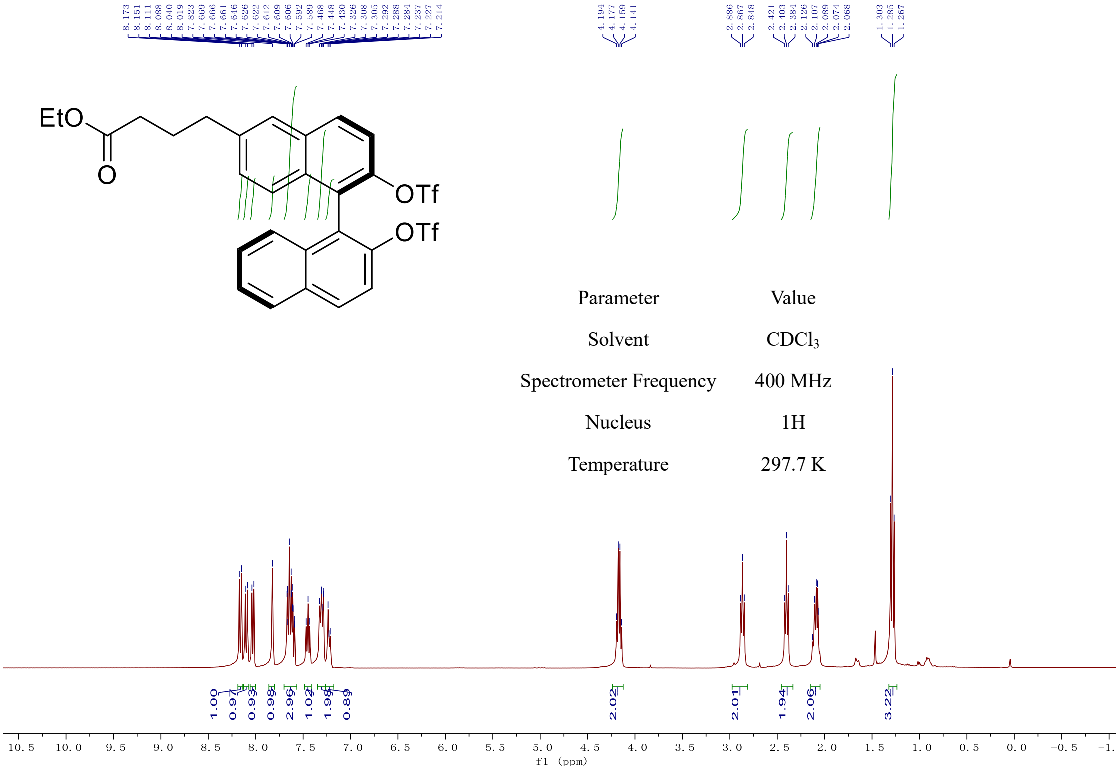

Supplementary Figure 154. **^{1}H NMR Spectrum of Compound S11**

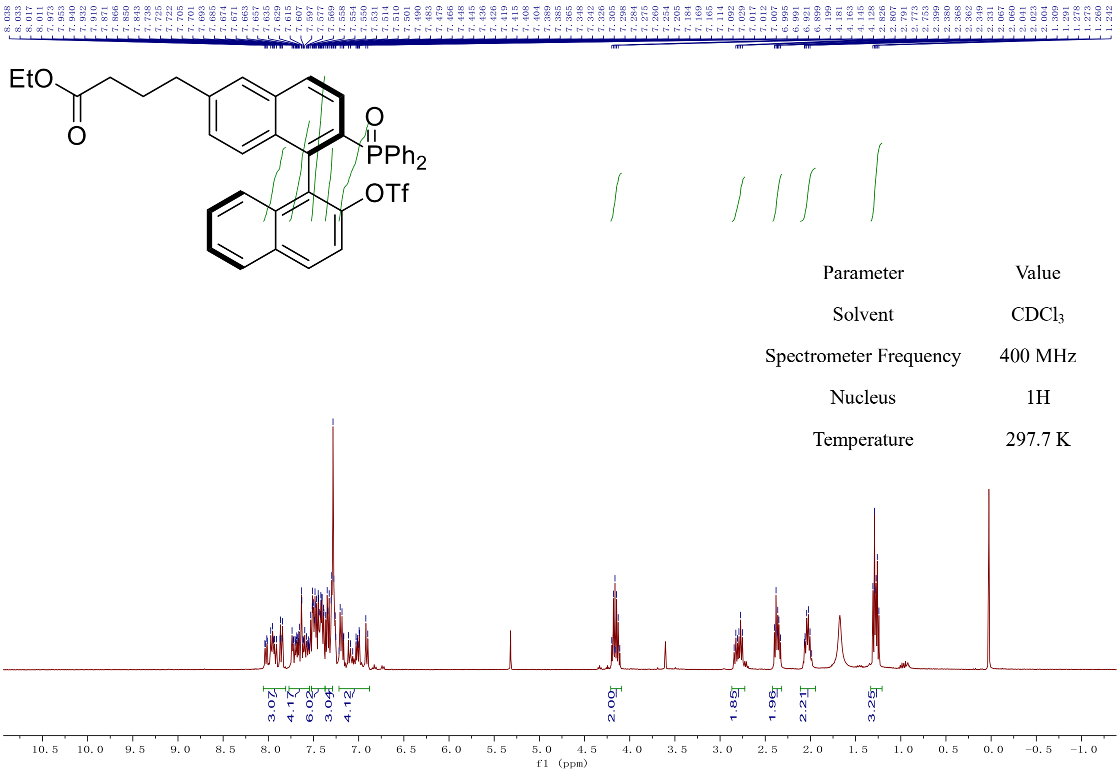

Supplementary Figure 155. **^{1}H NMR Spectrum of Compound S12**

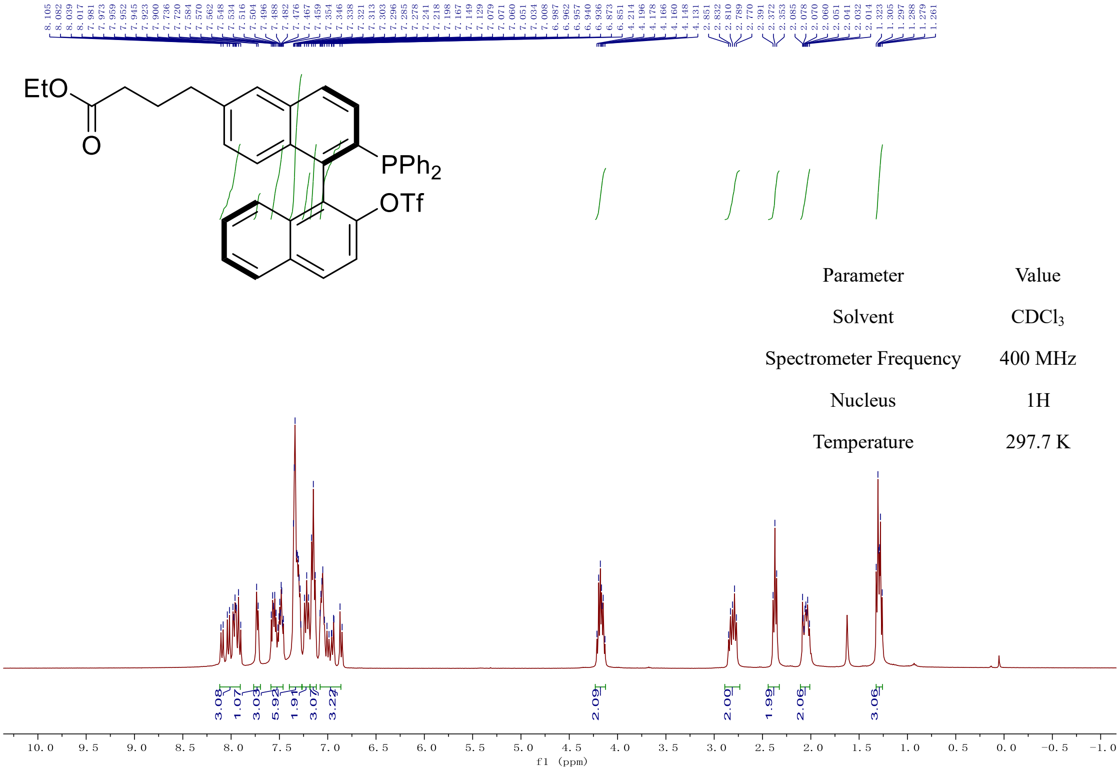

Supplementary Figure 156. **^{1}H NMR Spectrum of Compound S13**

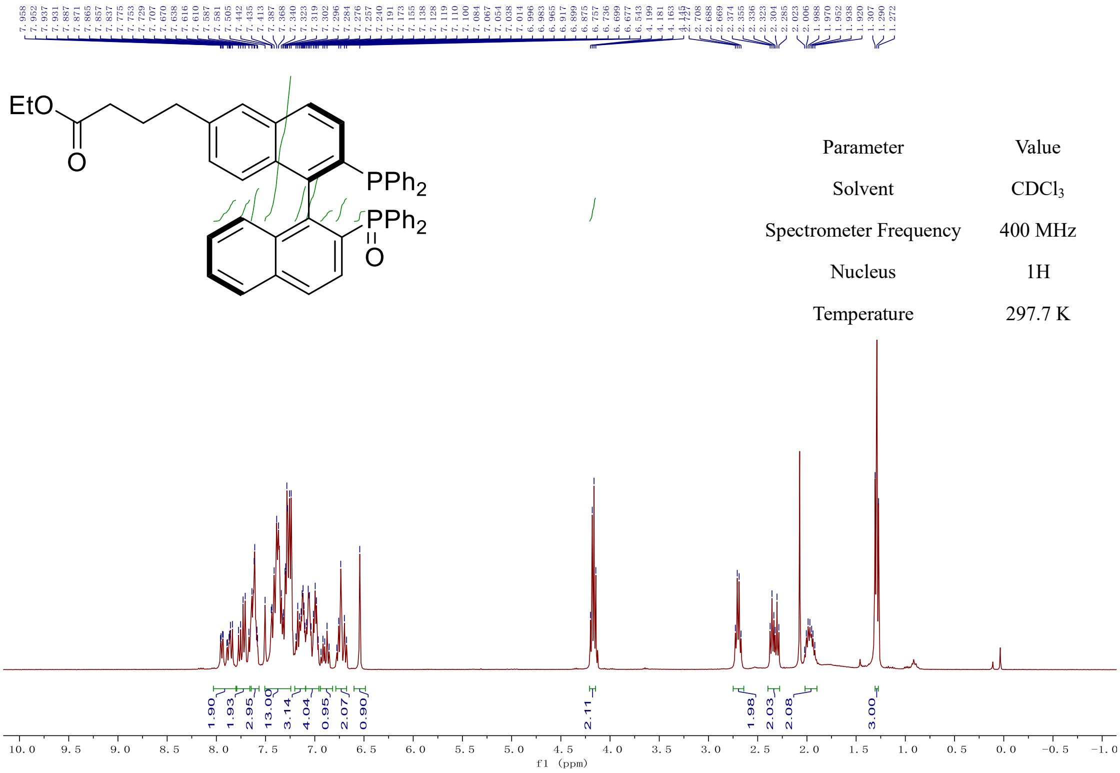

Supplementary Figure 157. **^{1}H NMR Spectrum of Compound S14**

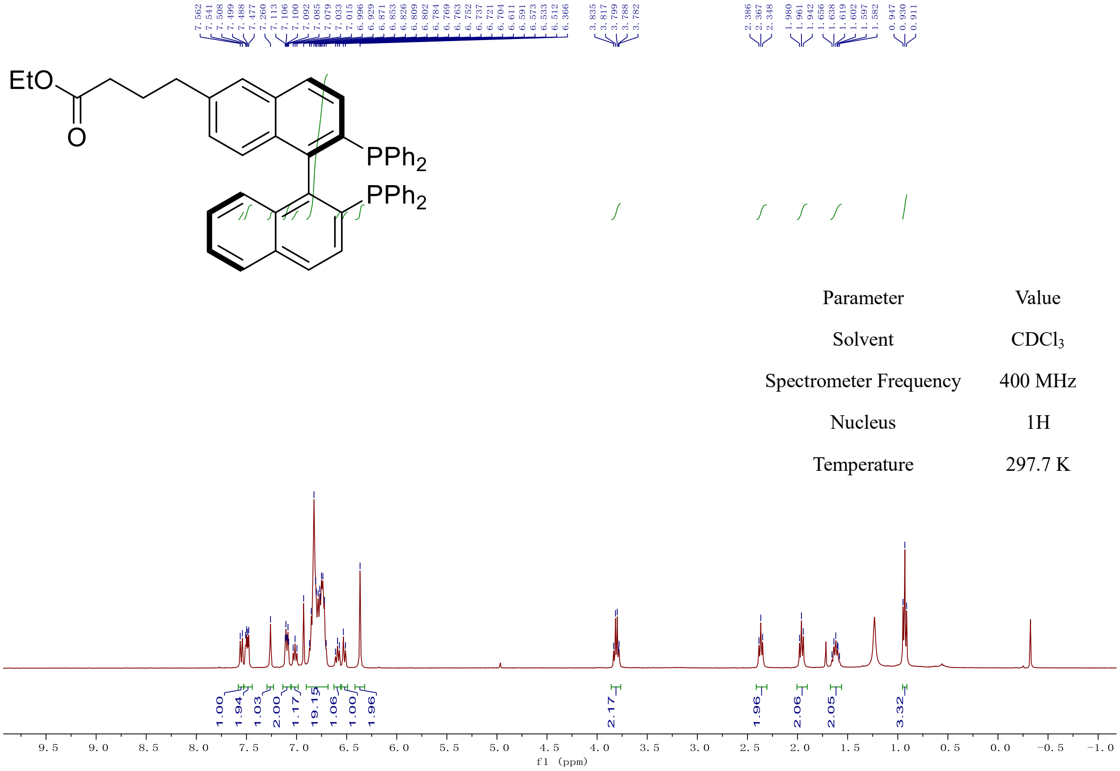

Supplementary Figure 158. **^{1}H NMR Spectrum of Compound S15**

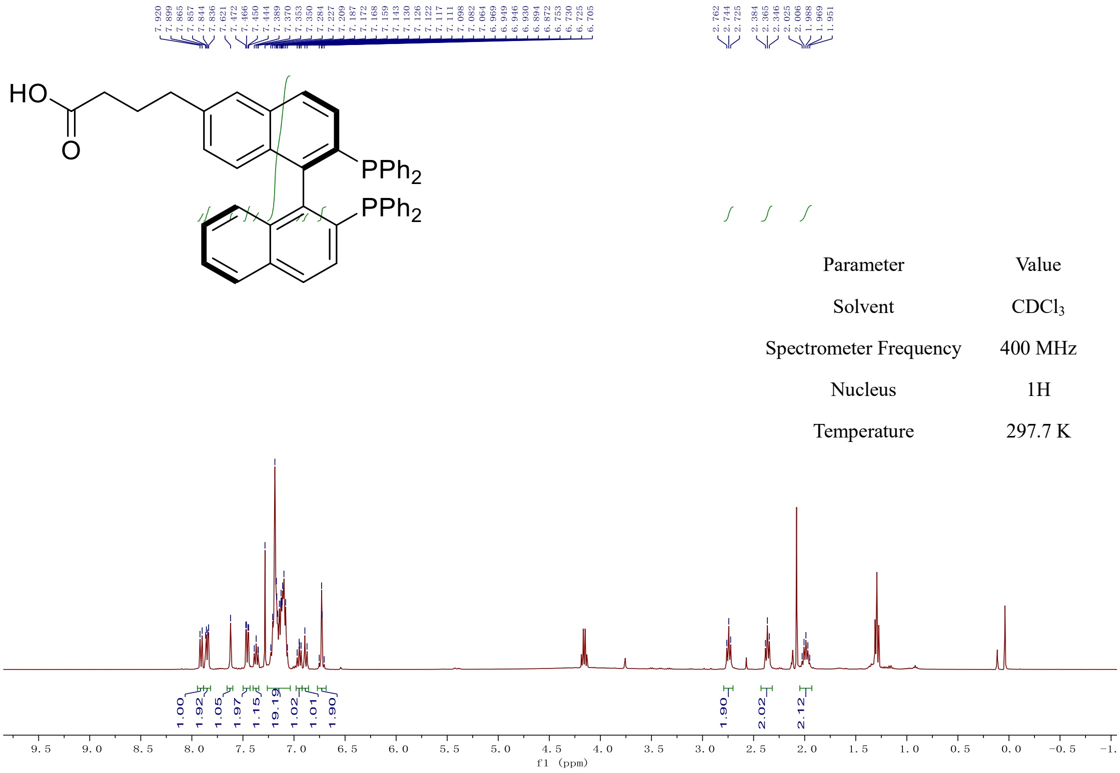

Parameter	Value
Solvent	$CDCl_3$
Spectrometer Frequency	400 MHz
Nucleus	1H
Temperature	297.7 K

Supplementary Figure 159.· ^{1}H NMR Spectrum of Compound **S16**

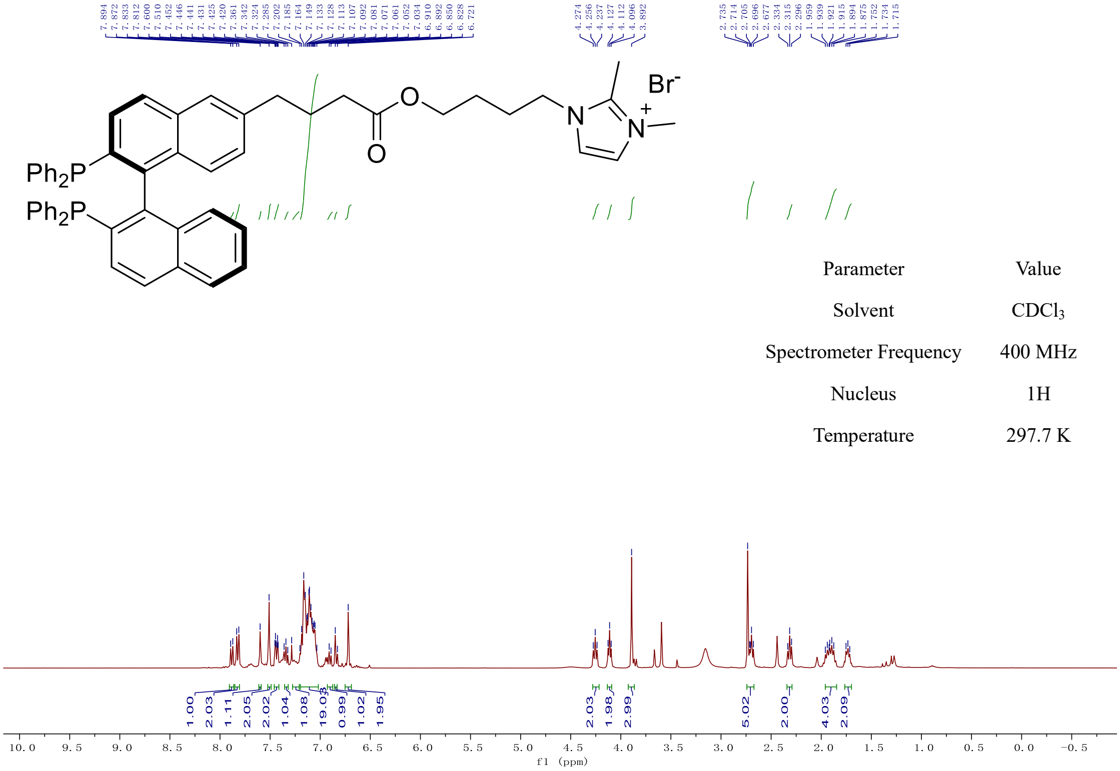

Parameter	Value
Solvent	$CDCl_3$
Spectrometer Frequency	400 MHz
Nucleus	1H
Temperature	297.7 K

^{1}H NMR Spectrum of Compound **L9**

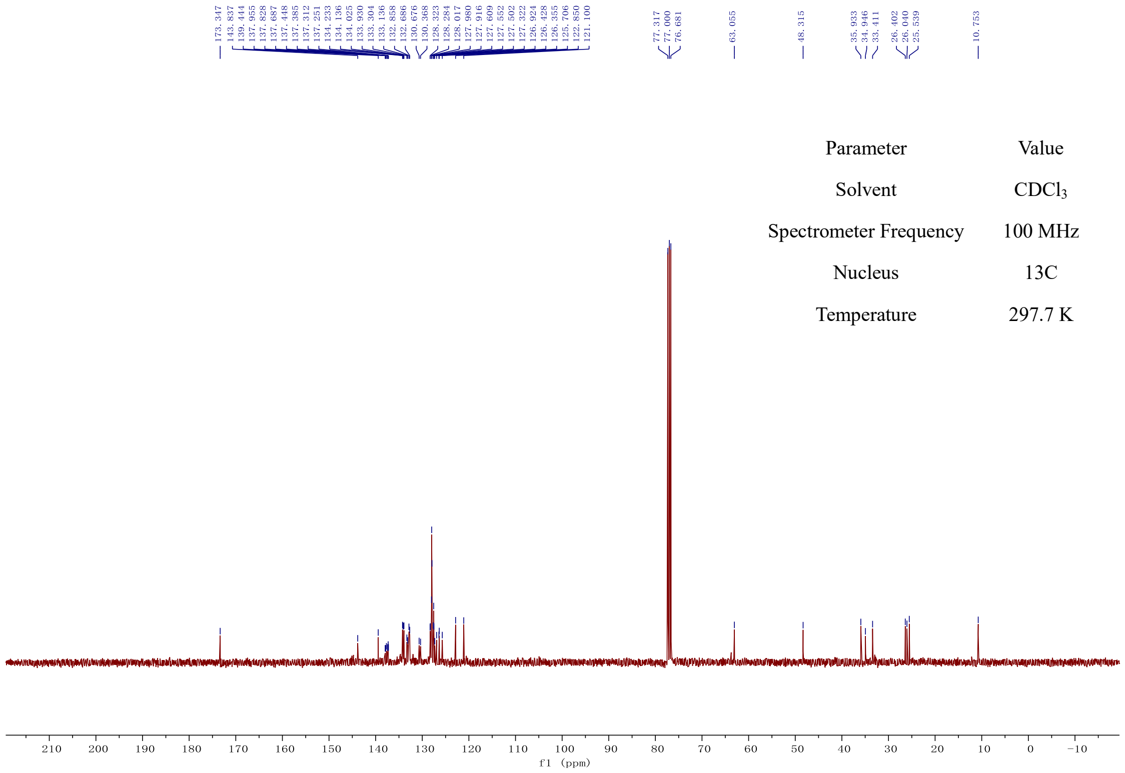

Parameter	Value
Solvent	$CDCl_3$
Spectrometer Frequency	100 MHz
Nucleus	13C
Temperature	297.7 K

^{13}C NMR Spectrum of Compound **L9**

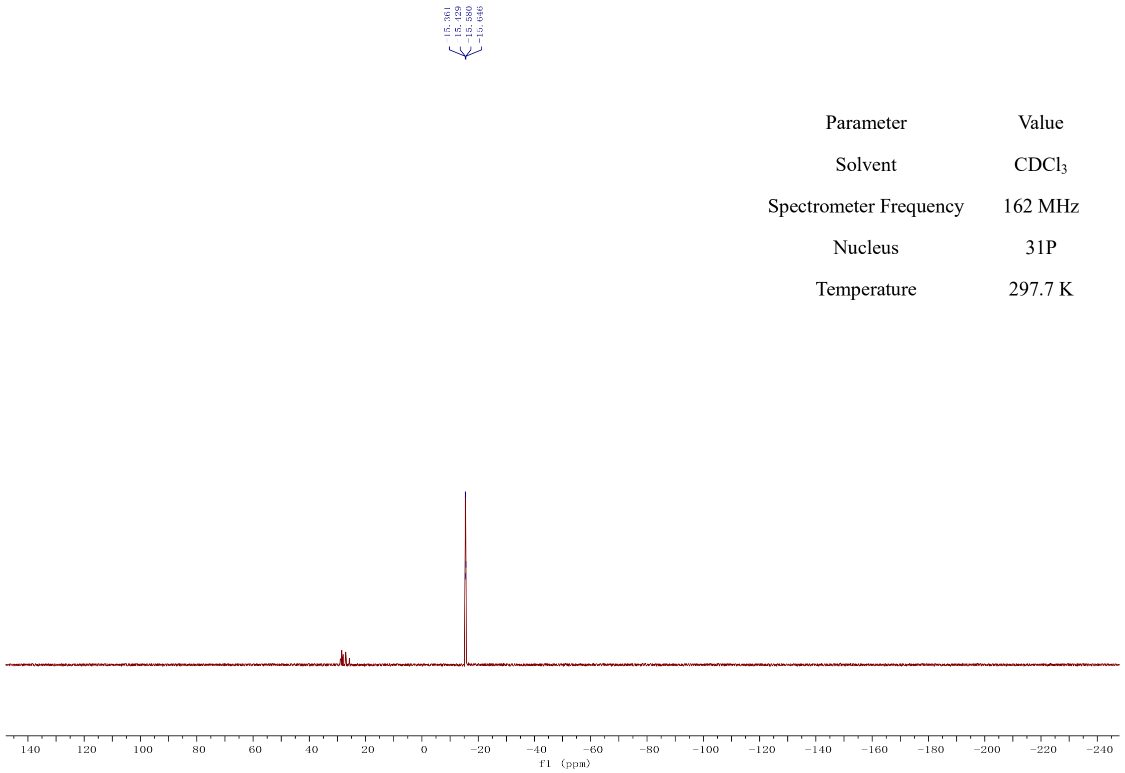

Parameter	Value
Solvent	$CDCl_3$
Spectrometer Frequency	162 MHz
Nucleus	31P
Temperature	297.7 K

^{31}P NMR Spectrum of Compound **L9**

Supplementary Figure 160**. NMR spectra of L9**

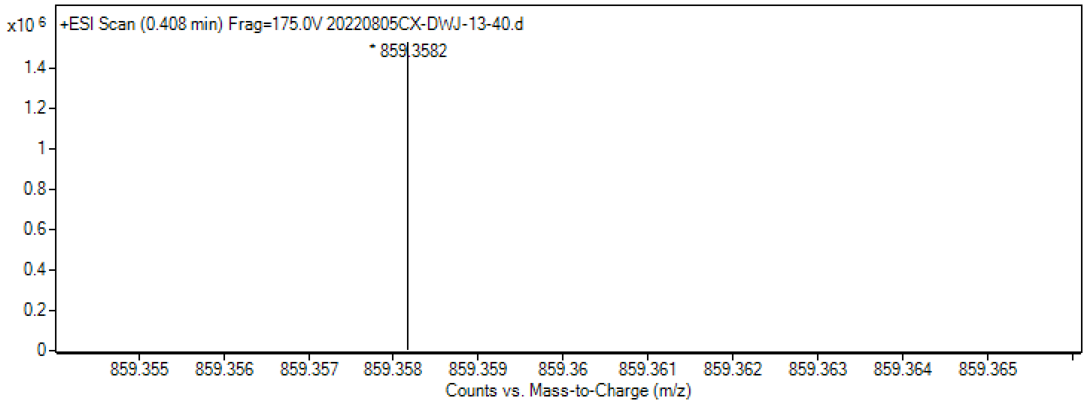

Supplementary Figure 161. **HRMS Spectrum of Compound L9**

3.3 X-ray single crystal data for the derivative compounds 3a

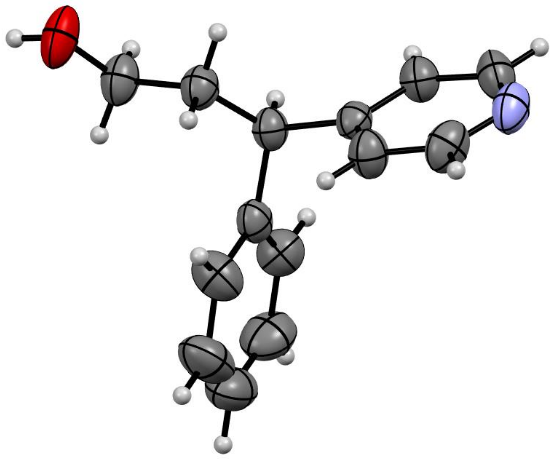

Supplementary Figure 162**. Crystal structure (CCDC 2118567)**

Table 1. Crystal data and structure refinement for 2118567.

Identification code	2118567	
Empirical formula	C14 H15NO	
Formula weight	213.27	
Temperature	293(2) K	
Wavelength	1.54178 Å	
Crystal system	Monoclinic	
Space group	P2(1)	
Unit cell dimensions	a = 7.8584(11) Å	= 90 °.
	b = 6.4170(7) Å	= 103.667(2) °.
	c = 11.9651(15) Å	= 90 °.
Volume	586.28(13) $Å^3$	
Z	2	
Density (calculated)	1.208 Mg/m^3	
Absorption coefficient	0.595 mm^{-1}	
F(000)	228	
Crystal size	0.27 x 0.15 x 0.12 mm^3	
Theta range for data collection	3.80 to 66.03 °.	
Index ranges	-9<=h<=7, -4<=k<=7, -14<=l<=11	
Reflections collected	1955	
Independent reflections	1370 [R(int) = 0.0130]	
Completeness to theta = 66.03 °	99.8 %	
Absorption correction	Semi-empirical from equivalents	
Max. and min. transmission	0.9320 and 0.8558	
Refinement method	Full-matrix least-squares on F^2	

Data / restraints / parameters	1370 / 1 / 148
Goodness-of-fit on F^2	1.042
Final R indices [I>2sigma(I)]	R1 = 0.0344, wR2 = 0.0842
R indices (all data)	R1 = 0.0379, wR2 = 0.0873
Absolute structure parameter	0.0(5)
Extinction coefficient	0.0131(15)
Largest diff. peak and hole	0.103 and -0.117 e.$Å^{-3}$

Supplementary Table 14. **Atomic coordinates (x 10^4) and equivalent isotropic displacement parameters (Å^2 x 10^3) for 2118567. U(equiv) is defined as one third of the trace of the orthogonalized tensor.**

	x	y	z	U(equiv)
N(1)	11818(3)	-284(4)	1401(2)	57(1)
O(1)	4900(2)	7647(3)	1100(2)	68(1)
C(1)	5107(3)	5676(5)	1615(2)	54(1)
C(2)	6898(3)	4834(4)	1589(2)	47(1)
C(3)	7205(3)	2636(4)	2074(2)	42(1)
C(4)	7290(3)	2478(4)	3363(2)	44(1)
C(5)	6576(3)	783(4)	3785(2)	58(1)
C(6)	6655(4)	582(5)	4950(2)	75(1)
C(7)	7471(4)	2056(6)	5701(2)	77(1)
C(8)	8208(4)	3727(6)	5306(2)	80(1)
C(9)	8112(3)	3955(5)	4142(2)	65(1)
C(10)	11924(3)	1462(5)	2003(2)	58(1)
C(11)	10491(3)	2487(4)	2226(2)	53(1)
C(12)	8844(3)	1658(4)	1820(2)	39(1)
C(13)	8727(3)	-130(4)	1179(2)	48(1)
C(14)	10234(3)	-1037(4)	998(2)	57(1)

Supplementary Table 15. **Bond lengths [Å] and angles [°] for 2118567.**

N(1)-C(14)	1.316(3)
N(1)-C(10)	1.324(4)
O(1)-C(1)	1.399(3)
O(1)-H(1)	0.8200
C(1)-C(2)	1.515(3)
C(1)-H(1A)	0.9700
C(1)-H(1B)	0.9700
C(2)-C(3)	1.523(3)
C(2)-H(2A)	0.9700
C(2)-H(2B)	0.9700
C(3)-C(12)	1.527(3)
C(3)-C(4)	1.531(3)
C(3)-H(3)	0.9800
C(4)-C(5)	1.373(3)
C(4)-C(9)	1.378(3)
C(5)-C(6)	1.387(3)
C(5)-H(5)	0.9300
C(6)-C(7)	1.357(4)
C(6)-H(6)	0.9300
C(7)-C(8)	1.356(5)
C(7)-H(7)	0.9300
C(8)-C(9)	1.384(4)
C(8)-H(8)	0.9300
C(9)-H(9)	0.9300
C(10)-C(11)	1.384(3)
C(10)-H(10)	0.9300
C(11)-C(12)	1.377(3)
C(11)-H(11)	0.9300
C(12)-C(13)	1.371(3)
C(13)-C(14)	1.383(3)
C(13)-H(13)	0.9300
C(14)-H(14)	0.9300
C(14)-N(1)-C(10)	116.3(2)
C(1)-O(1)-H(1)	109.5
O(1)-C(1)-C(2)	109.0(2)

O(1)-C(1)-H(1A)	109.9
C(2)-C(1)-H(1A)	109.9
O(1)-C(1)-H(1B)	109.9
C(2)-C(1)-H(1B)	109.9
H(1A)-C(1)-H(1B)	108.3
C(1)-C(2)-C(3)	112.8(2)
C(1)-C(2)-H(2A)	109.0
C(3)-C(2)-H(2A)	109.0
C(1)-C(2)-H(2B)	109.0
C(3)-C(2)-H(2B)	109.0
H(2A)-C(2)-H(2B)	107.8
C(2)-C(3)-C(12)	111.70(18)
C(2)-C(3)-C(4)	114.23(19)
C(12)-C(3)-C(4)	109.60(17)
C(2)-C(3)-H(3)	107.0
C(12)-C(3)-H(3)	107.0
C(4)-C(3)-H(3)	107.0
C(5)-C(4)-C(9)	117.6(2)
C(5)-C(4)-C(3)	119.9(2)
C(9)-C(4)-C(3)	122.5(2)
C(4)-C(5)-C(6)	121.2(3)
C(4)-C(5)-H(5)	119.4
C(6)-C(5)-H(5)	119.4
C(7)-C(6)-C(5)	120.2(3)
C(7)-C(6)-H(6)	119.9
C(5)-C(6)-H(6)	119.9
C(8)-C(7)-C(6)	119.6(3)
C(8)-C(7)-H(7)	120.2
C(6)-C(7)-H(7)	120.2
C(7)-C(8)-C(9)	120.6(3)
C(7)-C(8)-H(8)	119.7
C(9)-C(8)-H(8)	119.7
C(4)-C(9)-C(8)	120.9(3)
C(4)-C(9)-H(9)	119.6
C(8)-C(9)-H(9)	119.6
N(1)-C(10)-C(11)	123.9(2)
N(1)-C(10)-H(10)	118.1
C(11)-C(10)-H(10)	118.1

C(12)-C(11)-C(10)	119.2(3)
C(12)-C(11)-H(11)	120.4
C(10)-C(11)-H(11)	120.4
C(13)-C(12)-C(11)	117.0(2)
C(13)-C(12)-C(3)	120.5(2)
C(11)-C(12)-C(3)	122.5(2)
C(12)-C(13)-C(14)	119.6(2)
C(12)-C(13)-H(13)	120.2
C(14)-C(13)-H(13)	120.2
N(1)-C(14)-C(13)	124.0(3)
N(1)-C(14)-H(14)	118.0
C(13)-C(14)-H(14)	118.0

Symmetry transformations used to generate equivalent atoms:

Supplementary Table 16. **Anisotropic displacement parameters (Å^2 x 10^3) for 2118567.**

	U^{11}	U^{22}	U^{33}	U^{23}	U^{13}	U^{12}
N(1)	51(1)	56(1)	72(1)	14(1)	29(1)	17(1)
O(1)	48(1)	54(1)	109(1)	18(1)	31(1)	16(1)
C(1)	45(1)	48(1)	73(2)	5(1)	21(1)	7(1)
C(2)	43(1)	47(1)	54(1)	3(1)	16(1)	5(1)
C(3)	36(1)	43(1)	49(1)	-2(1)	13(1)	3(1)
C(4)	35(1)	48(1)	51(1)	-4(1)	13(1)	6(1)
C(5)	55(1)	58(2)	60(2)	2(1)	13(1)	-2(1)
C(6)	74(2)	87(2)	69(2)	26(2)	27(2)	3(2)
C(7)	73(2)	108(3)	52(2)	7(2)	20(1)	20(2)
C(8)	78(2)	102(3)	58(2)	-26(2)	12(1)	-5(2)
C(9)	71(2)	65(2)	64(2)	-12(1)	26(1)	-12(2)
C(10)	38(1)	62(2)	76(2)	10(2)	17(1)	2(1)
C(11)	44(1)	49(1)	66(1)	-7(1)	14(1)	1(1)
C(12)	39(1)	40(1)	41(1)	7(1)	13(1)	5(1)
C(13)	46(1)	45(1)	55(1)	-3(1)	15(1)	2(1)
C(14)	66(2)	45(1)	68(2)	-4(1)	31(1)	8(1)

Supplementary Table 17. **Hydrogen coordinates (x 10^4) and isotropic displacement parameters (Å^2 x 10^3) for 2118567.**

	x	y	z	U(equiv)
H(1)	3992	8178	1189	102
H(1A)	4205	4742	1202	65
H(1B)	4997	5777	2404	65
H(2A)	7788	5752	2031	57
H(2B)	7021	4832	802	57
H(3)	6206	1790	1677	50
H(5)	6030	-248	3280	69
H(6)	6148	-567	5217	90
H(7)	7524	1921	6483	92
H(8)	8783	4728	5821	96
H(9)	8609	5120	3883	78
H(10)	13028	2033	2294	70
H(11)	10638	3722	2645	63
H(13)	7640	-729	868	58
H(14)	10123	-2254	564	69

Supplementary Table 18. **Torsion angles [°] for 2118567**.

O(1)-C(1)-C(2)-C(3)	-176.9(2)
C(1)-C(2)-C(3)-C(12)	168.43(19)
C(1)-C(2)-C(3)-C(4)	-66.5(2)
C(2)-C(3)-C(4)-C(5)	142.5(2)
C(12)-C(3)-C(4)-C(5)	-91.3(2)
C(2)-C(3)-C(4)-C(9)	-39.5(3)
C(12)-C(3)-C(4)-C(9)	86.7(3)
C(9)-C(4)-C(5)-C(6)	1.0(4)
C(3)-C(4)-C(5)-C(6)	179.1(2)
C(4)-C(5)-C(6)-C(7)	-1.0(4)
C(5)-C(6)-C(7)-C(8)	0.0(5)
C(6)-C(7)-C(8)-C(9)	1.0(5)
C(5)-C(4)-C(9)-C(8)	0.0(4)
C(3)-C(4)-C(9)-C(8)	-178.1(2)
C(7)-C(8)-C(9)-C(4)	-0.9(5)
C(14)-N(1)-C(10)-C(11)	0.4(4)
N(1)-C(10)-C(11)-C(12)	0.9(4)
C(10)-C(11)-C(12)-C(13)	-2.0(3)
C(10)-C(11)-C(12)-C(3)	177.1(2)
C(2)-C(3)-C(12)-C(13)	-119.2(2)
C(4)-C(3)-C(12)-C(13)	113.1(2)
C(2)-C(3)-C(12)-C(11)	61.7(3)
C(4)-C(3)-C(12)-C(11)	-65.9(3)
C(11)-C(12)-C(13)-C(14)	1.7(3)
C(3)-C(12)-C(13)-C(14)	-177.4(2)
C(10)-N(1)-C(14)-C(13)	-0.7(4)
C(12)-C(13)-C(14)-N(1)	-0.4(4)

Symmetry transformations used to generate equivalent atoms:

Supplementary Table 19. **Hydrogen bonds for 2118567 [Å and °]**.

D-H	d(D-H)	d(H..A)	<DHA	d(D..A)	A
O1-H1	0.820	2.040	175.64	2.858	N1 [x-1, y+1, z]

4. Supplementary References

[1] Pan, D., Chen, A., Su, Y., Zhou, W., Li, S., Jia, W., Xiao, J., Liu, Q., Zhang, L., & Jiao, N. *Angew. Chem. In.t Ed. Engl.*, **47**, 4729-4732 (2008).
[2] Battistuzzi, G., Cacchi, S. & Fabrizi, G. *Org. Let.t*, **5**, 777-780 (2003).
[3] Wang, J., Wang, S., Wang, G., Zhang, J. & Yu, X. Q. *Chem. Commun.*, **48**, 11769-11771 (2012).
[4] Osborn, J. A. & Schrock, R. R., *J. Am. Chem. Soc.*, 93, 3089-3091 (2002)
[5] Onyeagusi, C. I., Shao, X. & Malcolmson, S. J. *Org. Lett.*, **22**, 1681-1685 (2020)
[6] Aakermark, B., Krakenberger, B., Hansson, S. & Vitagliano, A. *Organometallics*, **6**, 620-628 (2002).
[7] Faller, J. W., Thomsen, M. E., & Mattina, M. J., *J. Am. Chem. Soc.*, **93**, 2642-2653 (1971).
[8] Kazmaier, U. & Zumpe, F. L. *Angew. Chem. Int. Ed.*, **39**, 802-804 (2000).
[10] Ammann, C. J., Pregosin, P. S. & Rueegger, H. *J. Organomet. Chem.* **423**, 415-430 (1992)
[11] Gaussian 16, Revision C.01, Frisch, M. J., Trucks, G. W., Schlegel, H. B., Scuseria, G. E., Robb, M. A., Cheeseman, J. R., Scalmani, G., Barone, V., Petersson, G. A., Nakatsuji, H., Li, X., Caricato, M., Marenich, A. V., Bloino, J., Janesko, B. G., Gomperts, R., Mennucci, B., Hratchian, H. P., Ortiz, J. V., Izmaylov, A. F., Sonnenberg, J. L., Williams-Young, D., Ding, F., Lipparini, F., Egidi, F., Goings, J., Peng, B., Petrone, A., Henderson, T., Ranasinghe, D., Zakrzewski, V. G., Gao, J., Rega, N., Zheng, G., Liang, W., Hada, M., Ehara, M., Toyota, K., Fukuda, R., Hasegawa, J., Ishida, M., Nakajima, T., Honda, Y., Kitao, O., Nakai, H., Vreven, T., Throssell, K., Montgomery, J. A., Jr., Peralta, J. E., Ogliaro, F., Bearpark, M. J., Heyd, J. J., Brothers, E. N., Kudin, K. N., Staroverov, V. N., Keith, T. A., Kobayashi, R., Normand, J., Raghavachari, K., Rendell, A. P., Burant, J. C., Iyengar, S. S., Tomasi, J., Cossi, M., Millam, J. M., Klene, M., Adamo, C., Cammi, R., Ochterski, J. W., Martin, R. L., Morokuma, K., Farkas, O., Foresman, J. B. & Fox, D. J. Gaussian, Inc., Wallingford CT, 2016.
[12] Stevens, P. J., Devlin, F. J., Chablowski, C. F. & Frisch, M. J. *J. Phys. Chem.*, **98,** 11623 (1994).
[13] Grimme, S., Ehrlich, S. & Goerigk, L. *J. Comp. Chem.* **32**, 1456 (2011).
[14] Hehre, W. J., Ditchfield, R. & Pople, J.A. *J. Chem. Phys.*, **56**, 2257 (1972).
[15] Francl, M. M., Pietro, W. J., Hehre, W. J., Binkley, J. S., Gordon, M. S., DeFrees, D. J. & Pople, J. A. *J. Chem. Phys.*, **77**, 3654 (1982).
[16] Dolg, M., Wedig, U., Stoll, H. & Preuss, H. *J. Chem. Phys.*, **86**, 866 (1987).
[17] Fukui, K. *J. Phys. Chem.*, **74**, 4161 (1970).
[18] Fukui, K. *Acc. Chem. Res.*, **14**, 363 (1981).
[19] Weigend, F. & Ahlrichs, R. *Phys. Chem. Chem. Phys.*, **7**, 3297 (2005).
[20] Weigend, F. *Phys. Chem. Chem. Phys.*, 8, 1057 (2006).
[21] Andrae, D., Haeussermann, U., Dolg, M., Stoll, H. & Preuss, H. *Theor. Chim. Acta.*, **77**, 123 (1990).
[21] Marenich, A. V., Cramer, C. J. & Truhlar, D. G. *J. Phys. Chem. B*, **113**, 6378 (2009).
