# Supplementary material for: Palladium-catalyzed asymmetric allylic 4-pyridinylation via electroreductive substitution reaction
Source: Nat Commun. 2022 Sep 26;13:5642. doi: 10.1038/s41467-022-33452-0 (PMC9512896; doi:10.1038/s41467-022-33452-0)
Supplement: Supplementary file 2 — Description of Additional Supplementary Files [file 41467_2022_33452_MOESM2_ESM.docx]

**Description of Additional Supplementary Files**

File Name: Supplementary Data 1

Description: Cartessian coordinates of DFT optimized structures.
