# Supplementary material for: Palladium-catalyzed asymmetric allylic 4-pyridinylation via electroreductive substitution reaction
Source: Nat Commun. 2022 Sep 26;13:5642. doi: 10.1038/s41467-022-33452-0 (PMC9512896; doi:10.1038/s41467-022-33452-0)
Supplement: Supplementary file 3 — Supplementary Data 1 [file 41467_2022_33452_MOESM3_ESM.docx]

**Cartesian coordinates and energies of the optimized structures:**

The Cartesian coordinates of optimized structures are given below in the standard XYZ format,

and units are in Å.

**INT-C (*E*)**

| Center Number | Atomic  Number | Atomic  Type | Coordinates (Angstroms) | | |
| --- | --- | --- | --- | --- | --- |
|  |  |  | X | Y | Z |
| 1 | 46 | 0 | 0.932556 | 1.263228 | -0.51223 |
| 2 | 15 | 0 | 1.065052 | -1.05767 | -0.68343 |
| 3 | 15 | 0 | -1.23367 | 1.302215 | 0.391371 |
| 4 | 8 | 0 | -4.27532 | -2.687 | -2.72753 |
| 5 | 8 | 0 | -4.07749 | -2.20302 | -0.46892 |
| 6 | 8 | 0 | -1.84889 | -3.47367 | 3.88406 |
| 7 | 8 | 0 | -1.91156 | -3.78215 | 1.587548 |
| 8 | 6 | 0 | -1.66615 | -1.71377 | -0.48689 |
| 9 | 6 | 0 | -0.52452 | -1.68734 | -1.34462 |
| 10 | 6 | 0 | -0.65175 | -2.01441 | -2.69852 |
| 11 | 1 | 0 | 0.224552 | -2.01277 | -3.33406 |
| 12 | 6 | 0 | -1.886 | -2.36668 | -3.27318 |
| 13 | 1 | 0 | -1.97186 | -2.61777 | -4.32393 |
| 14 | 6 | 0 | -5.00826 | -2.56771 | -1.49857 |
| 15 | 1 | 0 | -5.4652 | -3.53066 | -1.25182 |
| 16 | 1 | 0 | -5.75872 | -1.77864 | -1.60194 |
| 17 | 6 | 0 | -2.85807 | -2.08991 | -1.07533 |
| 18 | 6 | 0 | -2.97834 | -2.38732 | -2.43305 |
| 19 | 6 | 0 | -1.63981 | -1.41534 | 0.970338 |
| 20 | 6 | 0 | -1.5104 | -0.10952 | 1.529616 |
| 21 | 6 | 0 | -1.50801 | 0.066263 | 2.916833 |
| 22 | 1 | 0 | -1.42916 | 1.062759 | 3.33104 |
| 23 | 6 | 0 | -1.61647 | -1.0138 | 3.810536 |
| 24 | 1 | 0 | -1.60578 | -0.86562 | 4.884008 |
| 25 | 6 | 0 | -2.01471 | -4.45585 | 2.850906 |
| 26 | 1 | 0 | -3.00481 | -4.91548 | 2.938722 |
| 27 | 1 | 0 | -1.21808 | -5.20005 | 2.9299 |
| 28 | 6 | 0 | -1.77739 | -2.45187 | 1.872712 |
| 29 | 6 | 0 | -1.74321 | -2.26823 | 3.254984 |
| 30 | 6 | 0 | 2.342178 | -1.58599 | -1.88078 |
| 31 | 6 | 0 | 2.444738 | -0.89572 | -3.10082 |
| 32 | 1 | 0 | 1.743654 | -0.09634 | -3.32725 |
| 33 | 6 | 0 | 3.442331 | -1.21966 | -4.01725 |
| 34 | 1 | 0 | 3.508032 | -0.68167 | -4.95819 |
| 35 | 6 | 0 | 4.362468 | -2.2294 | -3.71856 |
| 36 | 1 | 0 | 5.14665 | -2.47682 | -4.42742 |
| 37 | 6 | 0 | 4.272335 | -2.91243 | -2.50615 |
| 38 | 1 | 0 | 4.988234 | -3.69276 | -2.2661 |
| 39 | 6 | 0 | 3.268641 | -2.59385 | -1.58892 |
| 40 | 1 | 0 | 3.222661 | -3.11807 | -0.64205 |
| 41 | 6 | 0 | 1.373053 | -2.01532 | 0.832014 |
| 42 | 6 | 0 | 1.156356 | -3.40118 | 0.863822 |
| 43 | 1 | 0 | 0.785504 | -3.91361 | -0.01841 |
| 44 | 6 | 0 | 1.391183 | -4.11312 | 2.038197 |
| 45 | 1 | 0 | 1.232047 | -5.18739 | 2.057684 |
| 46 | 6 | 0 | 1.824818 | -3.44645 | 3.187832 |
| 47 | 1 | 0 | 1.994966 | -4.00219 | 4.10495 |
| 48 | 6 | 0 | 2.040146 | -2.06772 | 3.157166 |
| 49 | 1 | 0 | 2.379405 | -1.54841 | 4.048122 |
| 50 | 6 | 0 | 1.820239 | -1.35266 | 1.980713 |
| 51 | 1 | 0 | 1.992258 | -0.28279 | 1.949787 |
| 52 | 6 | 0 | -2.65116 | 1.240258 | -0.75008 |
| 53 | 6 | 0 | -2.41035 | 1.308511 | -2.12859 |
| 54 | 1 | 0 | -1.39137 | 1.426378 | -2.48436 |
| 55 | 6 | 0 | -3.46497 | 1.190056 | -3.03167 |
| 56 | 1 | 0 | -3.27142 | 1.226437 | -4.09929 |
| 57 | 6 | 0 | -4.76854 | 1.016247 | -2.56127 |
| 58 | 1 | 0 | -5.59083 | 0.927328 | -3.26486 |
| 59 | 6 | 0 | -5.01496 | 0.952042 | -1.18698 |
| 60 | 1 | 0 | -6.02829 | 0.816891 | -0.82013 |
| 61 | 6 | 0 | -3.95912 | 1.048775 | -0.28182 |
| 62 | 1 | 0 | -4.14565 | 0.95604 | 0.783723 |
| 63 | 6 | 0 | -1.43199 | 2.776777 | 1.453345 |
| 64 | 6 | 0 | -2.54355 | 3.624567 | 1.391702 |
| 65 | 1 | 0 | -3.35331 | 3.411475 | 0.702553 |
| 66 | 6 | 0 | -2.60821 | 4.755211 | 2.209184 |
| 67 | 1 | 0 | -3.4705 | 5.412161 | 2.147586 |
| 68 | 6 | 0 | -1.57644 | 5.037954 | 3.104212 |
| 69 | 1 | 0 | -1.63381 | 5.914486 | 3.742107 |
| 70 | 6 | 0 | -0.46451 | 4.193681 | 3.173735 |
| 71 | 1 | 0 | 0.345449 | 4.413286 | 3.862943 |
| 72 | 6 | 0 | -0.38506 | 3.079528 | 2.341655 |
| 73 | 1 | 0 | 0.493476 | 2.439392 | 2.375371 |
| 74 | 6 | 0 | 1.359904 | 3.397098 | -1.55472 |
| 75 | 6 | 0 | 2.394228 | 2.963997 | -0.73762 |
| 76 | 6 | 0 | 3.020952 | 1.691873 | -0.95529 |
| 77 | 1 | 0 | 1.229933 | 2.878871 | -2.50851 |
| 78 | 1 | 0 | 2.613732 | 3.523128 | 0.16774 |
| 79 | 1 | 0 | 3.140308 | 1.369854 | -1.98708 |
| 80 | 6 | 0 | 3.970086 | 1.09634 | -0.00239 |
| 81 | 6 | 0 | 4.857984 | 0.101934 | -0.4456 |
| 82 | 6 | 0 | 3.979277 | 1.442465 | 1.362653 |
| 83 | 6 | 0 | 5.710323 | -0.54191 | 0.44842 |
| 84 | 1 | 0 | 4.871406 | -0.16828 | -1.49633 |
| 85 | 6 | 0 | 4.838384 | 0.805293 | 2.252301 |
| 86 | 1 | 0 | 3.296227 | 2.201485 | 1.734341 |
| 87 | 6 | 0 | 5.702109 | -0.19721 | 1.800899 |
| 88 | 1 | 0 | 6.383811 | -1.3127 | 0.08579 |
| 89 | 1 | 0 | 4.832868 | 1.086259 | 3.301574 |
| 90 | 1 | 0 | 6.36728 | -0.69828 | 2.497355 |
| 91 | 6 | 0 | 0.636497 | 4.717076 | -1.47286 |
| 92 | 1 | 0 | 1.101254 | 5.326509 | -2.26728 |
| 93 | 6 | 0 | -0.84544 | 4.563408 | -1.84861 |
| 94 | 1 | 0 | -1.31653 | 5.546355 | -1.94856 |
| 95 | 1 | 0 | -0.96635 | 4.032188 | -2.79911 |
| 96 | 1 | 0 | -1.38398 | 4.00744 | -1.07774 |
| 97 | 6 | 0 | 0.814338 | 5.464609 | -0.14935 |
| 98 | 1 | 0 | 1.868155 | 5.67815 | 0.060008 |
| 99 | 1 | 0 | 0.287432 | 6.422874 | -0.19014 |
| 100 | 1 | 0 | 0.402814 | 4.893078 | 0.685629 |

**INT-C (*Z*)**

| Center Number | Atomic  Number | Atomic  Type | Coordinates (Angstroms) | | |
| --- | --- | --- | --- | --- | --- |
|  |  |  | X | Y | Z |
| 1 | 46 | 0 | -1.10971 | 1.121442 | 0.116398 |
| 2 | 15 | 0 | -0.91096 | -1.21152 | -0.12756 |
| 3 | 15 | 0 | 1.198454 | 1.507872 | 0.018877 |
| 4 | 8 | 0 | 3.413299 | -2.55291 | 3.742725 |
| 5 | 8 | 0 | 4.038566 | -1.77651 | 1.649475 |
| 6 | 8 | 0 | 4.005406 | -2.57734 | -3.27886 |
| 7 | 8 | 0 | 3.180939 | -3.22302 | -1.21225 |
| 8 | 6 | 0 | 1.765109 | -1.54139 | 0.735427 |
| 9 | 6 | 0 | 0.390661 | -1.7549 | 1.052718 |
| 10 | 6 | 0 | 0.031123 | -2.25667 | 2.308552 |
| 11 | 1 | 0 | -1.01169 | -2.43772 | 2.533296 |
| 12 | 6 | 0 | 0.980513 | -2.55768 | 3.300327 |
| 13 | 1 | 0 | 0.685631 | -2.9431 | 4.269329 |
| 14 | 6 | 0 | 4.54023 | -2.21134 | 2.922056 |
| 15 | 1 | 0 | 5.171124 | -3.09472 | 2.781961 |
| 16 | 1 | 0 | 5.092655 | -1.39244 | 3.391344 |
| 17 | 6 | 0 | 2.676696 | -1.87807 | 1.718629 |
| 18 | 6 | 0 | 2.303728 | -2.34994 | 2.976082 |
| 19 | 6 | 0 | 2.270985 | -1.02985 | -0.56463 |
| 20 | 6 | 0 | 2.141944 | 0.31717 | -1.01297 |
| 21 | 6 | 0 | 2.675416 | 0.70067 | -2.24835 |
| 22 | 1 | 0 | 2.597962 | 1.729879 | -2.57085 |
| 23 | 6 | 0 | 3.333069 | -0.20502 | -3.09802 |
| 24 | 1 | 0 | 3.732569 | 0.104326 | -4.05668 |
| 25 | 6 | 0 | 3.941934 | -3.66582 | -2.34569 |
| 26 | 1 | 0 | 4.955109 | -3.93164 | -2.02411 |
| 27 | 1 | 0 | 3.435111 | -4.51323 | -2.81352 |
| 28 | 6 | 0 | 2.94882 | -1.88918 | -1.40715 |
| 29 | 6 | 0 | 3.44729 | -1.50312 | -2.65079 |
| 30 | 6 | 0 | -2.38191 | -2.17945 | 0.361053 |
| 31 | 6 | 0 | -3.10145 | -1.76391 | 1.493287 |
| 32 | 1 | 0 | -2.80803 | -0.85638 | 2.013323 |
| 33 | 6 | 0 | -4.18941 | -2.50216 | 1.949518 |
| 34 | 1 | 0 | -4.73775 | -2.16902 | 2.82437 |
| 35 | 6 | 0 | -4.59141 | -3.64943 | 1.263026 |
| 36 | 1 | 0 | -5.44592 | -4.22017 | 1.613419 |
| 37 | 6 | 0 | -3.9039 | -4.04949 | 0.117821 |
| 38 | 1 | 0 | -4.22101 | -4.93318 | -0.42785 |
| 39 | 6 | 0 | -2.8043 | -3.31986 | -0.3341 |
| 40 | 1 | 0 | -2.28232 | -3.64086 | -1.22729 |
| 41 | 6 | 0 | -0.40395 | -1.86823 | -1.74934 |
| 42 | 6 | 0 | 0.079452 | -3.17822 | -1.88804 |
| 43 | 1 | 0 | 0.180336 | -3.8138 | -1.01407 |
| 44 | 6 | 0 | 0.462983 | -3.65026 | -3.1413 |
| 45 | 1 | 0 | 0.829296 | -4.66745 | -3.24486 |
| 46 | 6 | 0 | 0.384738 | -2.81572 | -4.25961 |
| 47 | 1 | 0 | 0.697573 | -3.18174 | -5.23265 |
| 48 | 6 | 0 | -0.08816 | -1.50949 | -4.12483 |
| 49 | 1 | 0 | -0.1404 | -0.85708 | -4.99113 |
| 50 | 6 | 0 | -0.48684 | -1.03835 | -2.87389 |
| 51 | 1 | 0 | -0.84077 | -0.01719 | -2.7581 |
| 52 | 6 | 0 | 2.005926 | 1.414916 | 1.650103 |
| 53 | 6 | 0 | 1.215381 | 1.323329 | 2.803038 |
| 54 | 1 | 0 | 0.133301 | 1.328073 | 2.708417 |
| 55 | 6 | 0 | 1.814541 | 1.18835 | 4.054702 |
| 56 | 1 | 0 | 1.197411 | 1.099331 | 4.943414 |
| 57 | 6 | 0 | 3.206358 | 1.159874 | 4.162405 |
| 58 | 1 | 0 | 3.672528 | 1.055899 | 5.137493 |
| 59 | 6 | 0 | 3.999184 | 1.261835 | 3.015982 |
| 60 | 1 | 0 | 5.082062 | 1.243772 | 3.097814 |
| 61 | 6 | 0 | 3.403722 | 1.37516 | 1.761167 |
| 62 | 1 | 0 | 4.020168 | 1.410738 | 0.86842 |
| 63 | 6 | 0 | 1.636064 | 3.124882 | -0.70703 |
| 64 | 6 | 0 | 2.530749 | 4.012513 | -0.09664 |
| 65 | 1 | 0 | 2.984406 | 3.761181 | 0.855128 |
| 66 | 6 | 0 | 2.835465 | 5.229921 | -0.70847 |
| 67 | 1 | 0 | 3.527274 | 5.913548 | -0.2259 |
| 68 | 6 | 0 | 2.257418 | 5.567644 | -1.93233 |
| 69 | 1 | 0 | 2.498315 | 6.514628 | -2.40533 |
| 70 | 6 | 0 | 1.362436 | 4.687551 | -2.54579 |
| 71 | 1 | 0 | 0.904573 | 4.947788 | -3.49538 |
| 72 | 6 | 0 | 1.047228 | 3.476988 | -1.93292 |
| 73 | 1 | 0 | 0.347692 | 2.7953 | -2.40971 |
| 74 | 6 | 0 | -1.81602 | 3.166243 | 0.738352 |
| 75 | 6 | 0 | -2.85369 | 2.214614 | 0.920154 |
| 76 | 6 | 0 | -3.40066 | 1.496205 | -0.15321 |
| 77 | 1 | 0 | -1.34178 | 3.494885 | 1.662424 |
| 78 | 1 | 0 | -3.10257 | 1.891133 | 1.929501 |
| 79 | 1 | 0 | -3.17693 | 1.840225 | -1.16304 |
| 80 | 6 | 0 | -4.5278 | 0.568298 | -0.05275 |
| 81 | 6 | 0 | -4.74462 | -0.37854 | -1.06834 |
| 82 | 6 | 0 | -5.44581 | 0.644507 | 1.008575 |
| 83 | 6 | 0 | -5.83399 | -1.23996 | -1.01341 |
| 84 | 1 | 0 | -4.03652 | -0.44631 | -1.89018 |
| 85 | 6 | 0 | -6.54486 | -0.20863 | 1.054868 |
| 86 | 1 | 0 | -5.31968 | 1.40017 | 1.77795 |
| 87 | 6 | 0 | -6.73829 | -1.15702 | 0.0488 |
| 88 | 1 | 0 | -5.9787 | -1.97838 | -1.79549 |
| 89 | 1 | 0 | -7.25663 | -0.12701 | 1.870884 |
| 90 | 1 | 0 | -7.59503 | -1.82289 | 0.086037 |
| 91 | 6 | 0 | -1.81146 | 4.234273 | -0.36251 |
| 92 | 1 | 0 | -1.42124 | 3.816562 | -1.29757 |
| 93 | 6 | 0 | -3.22785 | 4.772935 | -0.64221 |
| 94 | 1 | 0 | -3.9047 | 4.002102 | -1.02024 |
| 95 | 1 | 0 | -3.66958 | 5.186619 | 0.271744 |
| 96 | 1 | 0 | -3.18101 | 5.574876 | -1.38698 |
| 97 | 6 | 0 | -0.89735 | 5.391674 | 0.059988 |
| 98 | 1 | 0 | 0.106077 | 5.04466 | 0.312738 |
| 99 | 1 | 0 | -0.8083 | 6.128807 | -0.74324 |
| 100 | 1 | 0 | -1.3148 | 5.899035 | 0.938593 |

**INT-D**

| Center Number | Atomic  Number | Atomic  Type | Coordinates (Angstroms) | | |
| --- | --- | --- | --- | --- | --- |
|  |  |  | X | Y | Z |
| 1 | 46 | 0 | 1.028465 | 1.107902 | -0.48342 |
| 2 | 15 | 0 | 0.966686 | -1.186 | -0.83522 |
| 3 | 15 | 0 | -1.05878 | 1.404445 | 0.459221 |
| 4 | 8 | 0 | -4.64241 | -2.40331 | -2.43101 |
| 5 | 8 | 0 | -4.22452 | -1.89089 | -0.20859 |
| 6 | 8 | 0 | -1.87653 | -3.36186 | 3.952925 |
| 7 | 8 | 0 | -2.04228 | -3.64878 | 1.658569 |
| 8 | 6 | 0 | -1.78392 | -1.61057 | -0.42047 |
| 9 | 6 | 0 | -0.71136 | -1.71526 | -1.3605 |
| 10 | 6 | 0 | -0.97468 | -2.05442 | -2.69171 |
| 11 | 1 | 0 | -0.14909 | -2.13217 | -3.38954 |
| 12 | 6 | 0 | -2.27612 | -2.30103 | -3.16469 |
| 13 | 1 | 0 | -2.46597 | -2.55924 | -4.20046 |
| 14 | 6 | 0 | -5.25782 | -2.16286 | -1.15923 |
| 15 | 1 | 0 | -5.81153 | -3.05535 | -0.84805 |
| 16 | 1 | 0 | -5.91643 | -1.29113 | -1.23636 |
| 17 | 6 | 0 | -3.04523 | -1.89223 | -0.91171 |
| 18 | 6 | 0 | -3.29691 | -2.20719 | -2.24429 |
| 19 | 6 | 0 | -1.6406 | -1.29731 | 1.03313 |
| 20 | 6 | 0 | -1.42635 | 0.000514 | 1.593961 |
| 21 | 6 | 0 | -1.3604 | 0.162086 | 2.981162 |
| 22 | 1 | 0 | -1.20605 | 1.151999 | 3.392301 |
| 23 | 6 | 0 | -1.48863 | -0.91526 | 3.875849 |
| 24 | 1 | 0 | -1.42973 | -0.77365 | 4.949056 |
| 25 | 6 | 0 | -2.01805 | -4.33713 | 2.912418 |
| 26 | 1 | 0 | -2.96033 | -4.87835 | 3.046856 |
| 27 | 1 | 0 | -1.15926 | -5.01677 | 2.935921 |
| 28 | 6 | 0 | -1.79435 | -2.32815 | 1.94207 |
| 29 | 6 | 0 | -1.70089 | -2.15748 | 3.320409 |
| 30 | 6 | 0 | 2.10276 | -1.79019 | -2.14244 |
| 31 | 6 | 0 | 2.226515 | -0.99766 | -3.29706 |
| 32 | 1 | 0 | 1.615516 | -0.10316 | -3.39182 |
| 33 | 6 | 0 | 3.138089 | -1.33166 | -4.29601 |
| 34 | 1 | 0 | 3.21783 | -0.71123 | -5.1844 |
| 35 | 6 | 0 | 3.965468 | -2.44754 | -4.14159 |
| 36 | 1 | 0 | 4.690303 | -2.69949 | -4.91047 |
| 37 | 6 | 0 | 3.865662 | -3.22729 | -2.98883 |
| 38 | 1 | 0 | 4.513266 | -4.09 | -2.85791 |
| 39 | 6 | 0 | 2.937862 | -2.90533 | -1.99624 |
| 40 | 1 | 0 | 2.876763 | -3.51282 | -1.1005 |
| 41 | 6 | 0 | 1.30998 | -2.26407 | 0.603564 |
| 42 | 6 | 0 | 0.959625 | -3.62208 | 0.616998 |
| 43 | 1 | 0 | 0.451349 | -4.0562 | -0.2387 |
| 44 | 6 | 0 | 1.243571 | -4.4069 | 1.733681 |
| 45 | 1 | 0 | 0.983244 | -5.4623 | 1.73209 |
| 46 | 6 | 0 | 1.858577 | -3.83696 | 2.852636 |
| 47 | 1 | 0 | 2.069775 | -4.44793 | 3.726032 |
| 48 | 6 | 0 | 2.204427 | -2.48497 | 2.844136 |
| 49 | 1 | 0 | 2.69175 | -2.03648 | 3.70435 |
| 50 | 6 | 0 | 1.937075 | -1.70125 | 1.721959 |
| 51 | 1 | 0 | 2.211875 | -0.6524 | 1.71024 |
| 52 | 6 | 0 | -2.53297 | 1.415887 | -0.63094 |
| 53 | 6 | 0 | -2.32986 | 1.450897 | -2.0165 |
| 54 | 1 | 0 | -1.31322 | 1.494188 | -2.39588 |
| 55 | 6 | 0 | -3.41535 | 1.394015 | -2.88972 |
| 56 | 1 | 0 | -3.2478 | 1.400813 | -3.96279 |
| 57 | 6 | 0 | -4.71452 | 1.318472 | -2.38394 |
| 58 | 1 | 0 | -5.56086 | 1.275488 | -3.06379 |
| 59 | 6 | 0 | -4.92513 | 1.290975 | -1.00245 |
| 60 | 1 | 0 | -5.93532 | 1.232425 | -0.60573 |
| 61 | 6 | 0 | -3.8387 | 1.323887 | -0.12914 |
| 62 | 1 | 0 | -4.00009 | 1.259496 | 0.942802 |
| 63 | 6 | 0 | -1.21683 | 2.907157 | 1.502871 |
| 64 | 6 | 0 | -2.26081 | 3.834094 | 1.393502 |
| 65 | 1 | 0 | -3.08866 | 3.641573 | 0.720321 |
| 66 | 6 | 0 | -2.23523 | 5.017896 | 2.135275 |
| 67 | 1 | 0 | -3.04662 | 5.732992 | 2.030281 |
| 68 | 6 | 0 | -1.17929 | 5.283043 | 3.006066 |
| 69 | 1 | 0 | -1.16334 | 6.204003 | 3.581724 |
| 70 | 6 | 0 | -0.13147 | 4.364197 | 3.121889 |
| 71 | 1 | 0 | 0.70409 | 4.569468 | 3.78533 |
| 72 | 6 | 0 | -0.14075 | 3.197898 | 2.362077 |
| 73 | 1 | 0 | 0.700294 | 2.510107 | 2.409842 |
| 74 | 6 | 0 | 1.879509 | 3.528691 | -1.81378 |
| 75 | 6 | 0 | 2.51619 | 2.892605 | -0.75248 |
| 76 | 6 | 0 | 3.169017 | 1.622898 | -0.85614 |
| 77 | 1 | 0 | 1.900527 | 3.036793 | -2.78724 |
| 78 | 1 | 0 | 2.545035 | 3.4119 | 0.201936 |
| 79 | 1 | 0 | 3.389676 | 1.257601 | -1.85802 |
| 80 | 6 | 0 | 3.924106 | 0.981188 | 0.217019 |
| 81 | 6 | 0 | 4.649789 | -0.19889 | -0.06189 |
| 82 | 6 | 0 | 3.91769 | 1.434399 | 1.555916 |
| 83 | 6 | 0 | 5.306721 | -0.90009 | 0.942947 |
| 84 | 1 | 0 | 4.6643 | -0.57402 | -1.08154 |
| 85 | 6 | 0 | 4.583659 | 0.733157 | 2.557398 |
| 86 | 1 | 0 | 3.372337 | 2.337272 | 1.81455 |
| 87 | 6 | 0 | 5.279126 | -0.4442 | 2.264372 |
| 88 | 1 | 0 | 5.841273 | -1.81344 | 0.694251 |
| 89 | 1 | 0 | 4.557861 | 1.108343 | 3.577866 |
| 90 | 1 | 0 | 5.791732 | -0.99264 | 3.049188 |
| 91 | 6 | 0 | 1.107688 | 4.819735 | -1.74899 |
| 92 | 1 | 0 | 1.546188 | 5.506293 | -2.49381 |
| 93 | 6 | 0 | -0.35914 | 4.590381 | -2.16827 |
| 94 | 1 | 0 | -0.42201 | 4.07841 | -3.13561 |
| 95 | 1 | 0 | -0.86994 | 3.96898 | -1.42622 |
| 96 | 1 | 0 | -0.89866 | 5.542195 | -2.25082 |
| 97 | 6 | 0 | 1.164179 | 5.514933 | -0.38436 |
| 98 | 1 | 0 | 2.195415 | 5.733172 | -0.08437 |
| 99 | 1 | 0 | 0.615612 | 6.462845 | -0.41806 |
| 100 | 1 | 0 | 0.70523 | 4.894573 | 0.391477 |

**INT-E**

| Center Number | Atomic  Number | Atomic  Type | Coordinates (Angstroms) | | |
| --- | --- | --- | --- | --- | --- |
|  |  |  | X | Y | Z |
| 1 | 46 | 0 | -1.4536 | 1.415542 | -0.87171 |
| 2 | 15 | 0 | -2.83019 | -0.02639 | 0.397249 |
| 3 | 15 | 0 | 0.371595 | -0.06798 | -0.84606 |
| 4 | 8 | 0 | 0.440589 | -2.94805 | 4.404681 |
| 5 | 8 | 0 | 0.578362 | -3.60985 | 2.186898 |
| 6 | 8 | 0 | -2.21316 | -5.33733 | -1.80855 |
| 7 | 8 | 0 | -2.14099 | -4.5653 | 0.376016 |
| 8 | 6 | 0 | -1.05495 | -1.9996 | 1.306527 |
| 9 | 6 | 0 | -1.93685 | -0.95003 | 1.702964 |
| 10 | 6 | 0 | -2.04246 | -0.58623 | 3.048675 |
| 11 | 1 | 0 | -2.72856 | 0.198188 | 3.341024 |
| 12 | 6 | 0 | -1.2826 | -1.21178 | 4.053259 |
| 13 | 1 | 0 | -1.36499 | -0.91752 | 5.092962 |
| 14 | 6 | 0 | 1.091136 | -3.86112 | 3.503679 |
| 15 | 1 | 0 | 0.857254 | -4.88918 | 3.796058 |
| 16 | 1 | 0 | 2.167882 | -3.67356 | 3.513707 |
| 17 | 6 | 0 | -0.33501 | -2.60526 | 2.314828 |
| 18 | 6 | 0 | -0.42167 | -2.21042 | 3.651363 |
| 19 | 6 | 0 | -0.9341 | -2.47284 | -0.0956 |
| 20 | 6 | 0 | -0.32661 | -1.73453 | -1.15023 |
| 21 | 6 | 0 | -0.38715 | -2.20718 | -2.46805 |
| 22 | 1 | 0 | 0.061694 | -1.62651 | -3.26521 |
| 23 | 6 | 0 | -1.00034 | -3.42615 | -2.80107 |
| 24 | 1 | 0 | -1.04425 | -3.77941 | -3.82458 |
| 25 | 6 | 0 | -2.55502 | -5.66252 | -0.45231 |
| 26 | 1 | 0 | -2.02258 | -6.56977 | -0.1487 |
| 27 | 1 | 0 | -3.63836 | -5.7841 | -0.37396 |
| 28 | 6 | 0 | -1.49707 | -3.68611 | -0.4478 |
| 29 | 6 | 0 | -1.54384 | -4.1509 | -1.76132 |
| 30 | 6 | 0 | -4.0121 | 1.021647 | 1.31529 |
| 31 | 6 | 0 | -3.50674 | 2.171325 | 1.948216 |
| 32 | 1 | 0 | -2.44066 | 2.380949 | 1.909159 |
| 33 | 6 | 0 | -4.36098 | 3.043852 | 2.617874 |
| 34 | 1 | 0 | -3.95905 | 3.924697 | 3.109647 |
| 35 | 6 | 0 | -5.73566 | 2.789434 | 2.643834 |
| 36 | 1 | 0 | -6.40528 | 3.474364 | 3.154693 |
| 37 | 6 | 0 | -6.24423 | 1.655555 | 2.010332 |
| 38 | 1 | 0 | -7.31133 | 1.455745 | 2.026571 |
| 39 | 6 | 0 | -5.38741 | 0.769496 | 1.353063 |
| 40 | 1 | 0 | -5.79398 | -0.1058 | 0.859205 |
| 41 | 6 | 0 | -3.78853 | -1.30166 | -0.48013 |
| 42 | 6 | 0 | -4.47705 | -2.29975 | 0.225688 |
| 43 | 1 | 0 | -4.45054 | -2.31257 | 1.310925 |
| 44 | 6 | 0 | -5.17121 | -3.28885 | -0.4686 |
| 45 | 1 | 0 | -5.71237 | -4.05356 | 0.080905 |
| 46 | 6 | 0 | -5.16234 | -3.30206 | -1.86645 |
| 47 | 1 | 0 | -5.69204 | -4.08185 | -2.40505 |
| 48 | 6 | 0 | -4.46749 | -2.31762 | -2.57136 |
| 49 | 1 | 0 | -4.44841 | -2.33525 | -3.65658 |
| 50 | 6 | 0 | -3.78518 | -1.31728 | -1.8807 |
| 51 | 1 | 0 | -3.22488 | -0.56057 | -2.42142 |
| 52 | 6 | 0 | 1.253622 | -0.13087 | 0.755309 |
| 53 | 6 | 0 | 0.898487 | 0.777277 | 1.760598 |
| 54 | 1 | 0 | 0.122318 | 1.509562 | 1.567522 |
| 55 | 6 | 0 | 1.518561 | 0.72314 | 3.008152 |
| 56 | 1 | 0 | 1.233567 | 1.424854 | 3.785181 |
| 57 | 6 | 0 | 2.510773 | -0.22844 | 3.25167 |
| 58 | 1 | 0 | 2.990644 | -0.27155 | 4.22497 |
| 59 | 6 | 0 | 2.891452 | -1.11465 | 2.240663 |
| 60 | 1 | 0 | 3.679045 | -1.84437 | 2.401889 |
| 61 | 6 | 0 | 2.263959 | -1.07177 | 0.997744 |
| 62 | 1 | 0 | 2.570401 | -1.78334 | 0.242266 |
| 63 | 6 | 0 | 1.704879 | 0.038019 | -2.10532 |
| 64 | 6 | 0 | 2.530124 | -1.064 | -2.39066 |
| 65 | 1 | 0 | 2.375542 | -2.01829 | -1.90265 |
| 66 | 6 | 0 | 3.556454 | -0.94712 | -3.32827 |
| 67 | 1 | 0 | 4.166029 | -1.81531 | -3.55961 |
| 68 | 6 | 0 | 3.781017 | 0.26869 | -3.98435 |
| 69 | 1 | 0 | 4.581226 | 0.35213 | -4.71328 |
| 70 | 6 | 0 | 2.969043 | 1.367255 | -3.70039 |
| 71 | 1 | 0 | 3.134893 | 2.315299 | -4.20346 |
| 72 | 6 | 0 | 1.933209 | 1.247116 | -2.77085 |
| 73 | 1 | 0 | 1.308877 | 2.097353 | -2.5479 |
| 74 | 6 | 0 | -3.06675 | 2.846077 | -1.75553 |
| 75 | 6 | 0 | -1.99918 | 3.540404 | -1.18965 |
| 76 | 6 | 0 | -0.66061 | 3.352912 | -1.64525 |
| 77 | 1 | 0 | -2.91755 | 2.413457 | -2.74874 |
| 78 | 1 | 0 | -2.17668 | 4.109697 | -0.27867 |
| 79 | 1 | 0 | -0.53273 | 3.170581 | -2.71148 |
| 80 | 6 | 0 | 0.470319 | 4.007757 | -0.95959 |
| 81 | 6 | 0 | 0.485645 | 4.17159 | 0.437603 |
| 82 | 6 | 0 | 1.551709 | 4.515592 | -1.69893 |
| 83 | 6 | 0 | 1.534742 | 4.829567 | 1.071758 |
| 84 | 1 | 0 | -0.33004 | 3.769457 | 1.031761 |
| 85 | 6 | 0 | 2.607887 | 5.170642 | -1.06559 |
| 86 | 1 | 0 | 1.550059 | 4.418939 | -2.78178 |
| 87 | 6 | 0 | 2.603073 | 5.331495 | 0.321459 |
| 88 | 1 | 0 | 1.53687 | 4.935739 | 2.150909 |
| 89 | 1 | 0 | 3.429125 | 5.563573 | -1.65757 |
| 90 | 1 | 0 | 3.423604 | 5.838386 | 0.819368 |
| 91 | 6 | 0 | -4.50411 | 3.061049 | -1.35404 |
| 92 | 1 | 0 | -4.56535 | 3.09285 | -0.26203 |
| 93 | 6 | 0 | -4.96595 | 4.433964 | -1.89198 |
| 94 | 1 | 0 | -4.35826 | 5.248214 | -1.48416 |
| 95 | 1 | 0 | -4.89702 | 4.472725 | -2.98533 |
| 96 | 1 | 0 | -6.01011 | 4.611978 | -1.61349 |
| 97 | 6 | 0 | -5.41485 | 1.946923 | -1.87838 |
| 98 | 1 | 0 | -5.39747 | 1.916779 | -2.97534 |
| 99 | 1 | 0 | -5.11313 | 0.964596 | -1.51051 |
| 100 | 1 | 0 | -6.44888 | 2.120353 | -1.5652 |
| 101 | 6 | 0 | 3.924786 | 2.986564 | 2.75956 |
| 102 | 7 | 0 | 3.463481 | 3.717261 | 3.536131 |
| 103 | 6 | 0 | 4.481664 | 2.070356 | 1.808756 |
| 104 | 6 | 0 | 4.002623 | 2.029884 | 0.494224 |
| 105 | 6 | 0 | 5.497087 | 1.184576 | 2.197559 |
| 106 | 6 | 0 | 4.556245 | 1.097429 | -0.37481 |
| 107 | 1 | 0 | 3.220113 | 2.699414 | 0.162107 |
| 108 | 6 | 0 | 5.972246 | 0.27618 | 1.26239 |
| 109 | 1 | 0 | 5.887028 | 1.194031 | 3.208513 |
| 110 | 7 | 0 | 5.508714 | 0.233163 | 0.003639 |
| 111 | 1 | 0 | 4.225351 | 1.032155 | -1.40324 |
| 112 | 1 | 0 | 6.735349 | -0.45121 | 1.521888 |
| 113 | 30 | 0 | 6.057087 | -1.46981 | -1.30404 |
| 114 | 17 | 0 | 4.838779 | -3.10991 | -0.32735 |

**INT-F**

| Center Number | Atomic  Number | Atomic  Type | Coordinates (Angstroms) | | |
| --- | --- | --- | --- | --- | --- |
|  |  |  | X | Y | Z |
| 1 | 46 | 0 | 0.37499 | 1.445872 | -0.08457 |
| 2 | 15 | 0 | 2.631405 | 0.740564 | -0.27286 |
| 3 | 15 | 0 | -0.57497 | -0.65096 | 0.362308 |
| 4 | 8 | 0 | 2.866772 | -4.86612 | -2.28082 |
| 5 | 8 | 0 | 1.940616 | -4.48831 | -0.19541 |
| 6 | 8 | 0 | 3.346465 | -2.55235 | 4.451922 |
| 7 | 8 | 0 | 3.911381 | -2.847 | 2.221752 |
| 8 | 6 | 0 | 2.374896 | -2.06137 | -0.11635 |
| 9 | 6 | 0 | 2.941502 | -0.98314 | -0.86363 |
| 10 | 6 | 0 | 3.540166 | -1.23583 | -2.10198 |
| 11 | 1 | 0 | 3.978065 | -0.41635 | -2.65781 |
| 12 | 6 | 0 | 3.582066 | -2.52201 | -2.67287 |
| 13 | 1 | 0 | 4.043842 | -2.69895 | -3.63776 |
| 14 | 6 | 0 | 1.994711 | -5.41716 | -1.28294 |
| 15 | 1 | 0 | 2.403108 | -6.36649 | -0.93051 |
| 16 | 1 | 0 | 0.988973 | -5.53641 | -1.70649 |
| 17 | 6 | 0 | 2.426969 | -3.30882 | -0.70269 |
| 18 | 6 | 0 | 2.990822 | -3.53915 | -1.95668 |
| 19 | 6 | 0 | 1.837563 | -1.91632 | 1.264029 |
| 20 | 6 | 0 | 0.569111 | -1.38071 | 1.619006 |
| 21 | 6 | 0 | 0.24584 | -1.20221 | 2.972323 |
| 22 | 1 | 0 | -0.70903 | -0.7634 | 3.234648 |
| 23 | 6 | 0 | 1.113492 | -1.56558 | 4.01521 |
| 24 | 1 | 0 | 0.848282 | -1.40964 | 5.054724 |
| 25 | 6 | 0 | 4.359819 | -3.04609 | 3.566251 |
| 26 | 1 | 0 | 4.510397 | -4.11732 | 3.744817 |
| 27 | 1 | 0 | 5.283257 | -2.48138 | 3.725998 |
| 28 | 6 | 0 | 2.65498 | -2.3015 | 2.31377 |
| 29 | 6 | 0 | 2.318975 | -2.12507 | 3.650824 |
| 30 | 6 | 0 | 3.697358 | 1.760425 | -1.36235 |
| 31 | 6 | 0 | 3.257705 | 1.974048 | -2.68056 |
| 32 | 1 | 0 | 2.332265 | 1.522389 | -3.0256 |
| 33 | 6 | 0 | 3.987959 | 2.781899 | -3.5497 |
| 34 | 1 | 0 | 3.629602 | 2.935947 | -4.56334 |
| 35 | 6 | 0 | 5.156508 | 3.407845 | -3.10927 |
| 36 | 1 | 0 | 5.719783 | 4.047726 | -3.78252 |
| 37 | 6 | 0 | 5.589374 | 3.217572 | -1.79689 |
| 38 | 1 | 0 | 6.492267 | 3.708425 | -1.44423 |
| 39 | 6 | 0 | 4.867062 | 2.397655 | -0.92743 |
| 40 | 1 | 0 | 5.213913 | 2.259666 | 0.090328 |
| 41 | 6 | 0 | 3.482684 | 0.752243 | 1.352618 |
| 42 | 6 | 0 | 4.746955 | 0.171315 | 1.529023 |
| 43 | 1 | 0 | 5.246291 | -0.29544 | 0.685503 |
| 44 | 6 | 0 | 5.350521 | 0.172635 | 2.785498 |
| 45 | 1 | 0 | 6.335511 | -0.26918 | 2.911319 |
| 46 | 6 | 0 | 4.687294 | 0.731598 | 3.881704 |
| 47 | 1 | 0 | 5.151474 | 0.716339 | 4.863624 |
| 48 | 6 | 0 | 3.424705 | 1.301853 | 3.714185 |
| 49 | 1 | 0 | 2.899664 | 1.723892 | 4.56616 |
| 50 | 6 | 0 | 2.82716 | 1.317345 | 2.453681 |
| 51 | 1 | 0 | 1.833715 | 1.734378 | 2.315855 |
| 52 | 6 | 0 | -0.62688 | -1.81913 | -1.07006 |
| 53 | 6 | 0 | 0.1266 | -1.46843 | -2.20081 |
| 54 | 1 | 0 | 0.687926 | -0.54283 | -2.19292 |
| 55 | 6 | 0 | 0.16247 | -2.29108 | -3.32396 |
| 56 | 1 | 0 | 0.777093 | -2.01033 | -4.17384 |
| 57 | 6 | 0 | -0.59799 | -3.46335 | -3.35346 |
| 58 | 1 | 0 | -0.58224 | -4.10369 | -4.23112 |
| 59 | 6 | 0 | -1.39574 | -3.7947 | -2.25565 |
| 60 | 1 | 0 | -2.02607 | -4.67887 | -2.28049 |
| 61 | 6 | 0 | -1.40556 | -2.98297 | -1.11842 |
| 62 | 1 | 0 | -2.05762 | -3.25003 | -0.29757 |
| 63 | 6 | 0 | -2.193 | -0.90429 | 1.200412 |
| 64 | 6 | 0 | -2.55887 | -2.1063 | 1.832203 |
| 65 | 1 | 0 | -1.86721 | -2.942 | 1.848464 |
| 66 | 6 | 0 | -3.7899 | -2.22268 | 2.47163 |
| 67 | 1 | 0 | -4.06876 | -3.16132 | 2.939965 |
| 68 | 6 | 0 | -4.66766 | -1.13552 | 2.503135 |
| 69 | 1 | 0 | -5.62483 | -1.22342 | 3.008568 |
| 70 | 6 | 0 | -4.30694 | 0.070979 | 1.895565 |
| 71 | 1 | 0 | -4.95625 | 0.936774 | 1.960313 |
| 72 | 6 | 0 | -3.06832 | 0.182093 | 1.255073 |
| 73 | 1 | 0 | -2.76318 | 1.124329 | 0.820038 |
| 74 | 6 | 0 | 0.680137 | 3.627953 | -0.11347 |
| 75 | 6 | 0 | -0.66275 | 3.488932 | 0.310979 |
| 76 | 6 | 0 | -1.09959 | 3.343604 | 1.653327 |
| 77 | 1 | 0 | 1.41925 | 3.782492 | 0.67445 |
| 78 | 1 | 0 | -1.43342 | 3.657421 | -0.43299 |
| 79 | 1 | 0 | -0.33955 | 3.199534 | 2.419334 |
| 80 | 6 | 0 | -2.45995 | 3.378277 | 2.093677 |
| 81 | 6 | 0 | -3.55198 | 3.695382 | 1.238262 |
| 82 | 6 | 0 | -2.7747 | 3.086875 | 3.447283 |
| 83 | 6 | 0 | -4.85903 | 3.711193 | 1.710057 |
| 84 | 1 | 0 | -3.36203 | 3.965835 | 0.204218 |
| 85 | 6 | 0 | -4.08175 | 3.104677 | 3.912136 |
| 86 | 1 | 0 | -1.9621 | 2.839295 | 4.126267 |
| 87 | 6 | 0 | -5.1416 | 3.415225 | 3.049832 |
| 88 | 1 | 0 | -5.66848 | 3.97241 | 1.032315 |
| 89 | 1 | 0 | -4.28251 | 2.873126 | 4.954879 |
| 90 | 1 | 0 | -6.16405 | 3.433621 | 3.414563 |
| 91 | 6 | 0 | 1.068081 | 4.245417 | -1.45963 |
| 92 | 1 | 0 | 1.426874 | 3.459262 | -2.13006 |
| 93 | 6 | 0 | -0.09249 | 4.938564 | -2.18517 |
| 94 | 1 | 0 | -0.8873 | 4.232599 | -2.44932 |
| 95 | 1 | 0 | -0.53032 | 5.735918 | -1.57223 |
| 96 | 1 | 0 | 0.26253 | 5.38108 | -3.12267 |
| 97 | 6 | 0 | 2.237322 | 5.219862 | -1.25909 |
| 98 | 1 | 0 | 1.942191 | 6.057095 | -0.61327 |
| 99 | 1 | 0 | 3.089093 | 4.712482 | -0.79461 |
| 100 | 1 | 0 | 2.575777 | 5.628493 | -2.21787 |
| 101 | 6 | 0 | -0.97011 | 1.42659 | -3.6223 |
| 102 | 7 | 0 | 0.034459 | 1.786993 | -4.08227 |
| 103 | 6 | 0 | -2.19305 | 0.944656 | -3.04864 |
| 104 | 6 | 0 | -2.94172 | 1.746164 | -2.17669 |
| 105 | 6 | 0 | -2.62056 | -0.36112 | -3.31228 |
| 106 | 6 | 0 | -4.06017 | 1.189955 | -1.56982 |
| 107 | 1 | 0 | -2.63902 | 2.760922 | -1.95691 |
| 108 | 6 | 0 | -3.73658 | -0.83984 | -2.63692 |
| 109 | 1 | 0 | -2.06299 | -1.01017 | -3.97299 |
| 110 | 7 | 0 | -4.43217 | -0.08253 | -1.77567 |
| 111 | 1 | 0 | -4.65351 | 1.759918 | -0.86189 |
| 112 | 1 | 0 | -4.06607 | -1.86781 | -2.74046 |
| 113 | 30 | 0 | -5.88286 | -1.13312 | -0.42953 |
| 114 | 17 | 0 | -5.19978 | -3.27228 | -0.77166 |

**INT-G**

| Center Number | Atomic  Number | Atomic  Type | Coordinates (Angstroms) | | |
| --- | --- | --- | --- | --- | --- |
|  |  |  | X | Y | Z |
| 1 | 46 | 0 | 1.190189 | 0.426895 | -0.01968 |
| 2 | 15 | 0 | 0.014616 | -1.4297 | -0.86296 |
| 3 | 15 | 0 | -0.68532 | 1.502215 | 0.880287 |
| 4 | 8 | 0 | -3.83645 | 1.78737 | -4.08013 |
| 5 | 8 | 0 | -4.29164 | 1.472609 | -1.83163 |
| 6 | 8 | 0 | -5.44111 | -1.9967 | 1.625172 |
| 7 | 8 | 0 | -4.64408 | -1.65269 | -0.52507 |
| 8 | 6 | 0 | -2.36534 | 0.015317 | -1.33559 |
| 9 | 6 | 0 | -1.2396 | -0.62069 | -1.949 |
| 10 | 6 | 0 | -1.00072 | -0.45309 | -3.31585 |
| 11 | 1 | 0 | -0.1526 | -0.95052 | -3.77016 |
| 12 | 6 | 0 | -1.81804 | 0.346879 | -4.13544 |
| 13 | 1 | 0 | -1.6174 | 0.466465 | -5.19415 |
| 14 | 6 | 0 | -4.63504 | 2.251312 | -2.98232 |
| 15 | 1 | 0 | -5.69248 | 2.103437 | -3.21715 |
| 16 | 1 | 0 | -4.40588 | 3.304975 | -2.7847 |
| 17 | 6 | 0 | -3.15313 | 0.785787 | -2.17136 |
| 18 | 6 | 0 | -2.88482 | 0.970125 | -3.52578 |
| 19 | 6 | 0 | -2.79449 | -0.14131 | 0.085694 |
| 20 | 6 | 0 | -2.19401 | 0.514558 | 1.20239 |
| 21 | 6 | 0 | -2.70462 | 0.324465 | 2.490999 |
| 22 | 1 | 0 | -2.24334 | 0.838489 | 3.325162 |
| 23 | 6 | 0 | -3.80659 | -0.51019 | 2.74643 |
| 24 | 1 | 0 | -4.18961 | -0.65645 | 3.75007 |
| 25 | 6 | 0 | -5.62334 | -2.352 | 0.248197 |
| 26 | 1 | 0 | -6.62578 | -2.0511 | -0.07626 |
| 27 | 1 | 0 | -5.47443 | -3.43046 | 0.131871 |
| 28 | 6 | 0 | -3.89273 | -0.93072 | 0.36755 |
| 29 | 6 | 0 | -4.37693 | -1.13285 | 1.657678 |
| 30 | 6 | 0 | 1.031028 | -2.49711 | -1.95873 |
| 31 | 6 | 0 | 1.965667 | -1.86093 | -2.79372 |
| 32 | 1 | 0 | 2.029188 | -0.77659 | -2.78794 |
| 33 | 6 | 0 | 2.83211 | -2.60419 | -3.59136 |
| 34 | 1 | 0 | 3.537506 | -2.09255 | -4.23957 |
| 35 | 6 | 0 | 2.812381 | -4.00038 | -3.53186 |
| 36 | 1 | 0 | 3.500869 | -4.58236 | -4.13773 |
| 37 | 6 | 0 | 1.914403 | -4.64135 | -2.67804 |
| 38 | 1 | 0 | 1.902349 | -5.72607 | -2.61577 |
| 39 | 6 | 0 | 1.022831 | -3.89744 | -1.90156 |
| 40 | 1 | 0 | 0.330509 | -4.4085 | -1.24246 |
| 41 | 6 | 0 | -1.01528 | -2.60078 | 0.097396 |
| 42 | 6 | 0 | -1.93589 | -3.44853 | -0.53763 |
| 43 | 1 | 0 | -2.02834 | -3.42978 | -1.61945 |
| 44 | 6 | 0 | -2.74748 | -4.29298 | 0.216903 |
| 45 | 1 | 0 | -3.44909 | -4.95621 | -0.2822 |
| 46 | 6 | 0 | -2.6685 | -4.27428 | 1.612751 |
| 47 | 1 | 0 | -3.31622 | -4.91696 | 2.202361 |
| 48 | 6 | 0 | -1.76089 | -3.4259 | 2.24717 |
| 49 | 1 | 0 | -1.69315 | -3.40598 | 3.329968 |
| 50 | 6 | 0 | -0.92814 | -2.60036 | 1.492588 |
| 51 | 1 | 0 | -0.21794 | -1.94556 | 1.984129 |
| 52 | 6 | 0 | -1.34906 | 2.873598 | -0.13543 |
| 53 | 6 | 0 | -0.6834 | 3.238215 | -1.31203 |
| 54 | 1 | 0 | 0.229883 | 2.721086 | -1.58099 |
| 55 | 6 | 0 | -1.19844 | 4.24659 | -2.12664 |
| 56 | 1 | 0 | -0.6785 | 4.519887 | -3.04016 |
| 57 | 6 | 0 | -2.37715 | 4.902709 | -1.76675 |
| 58 | 1 | 0 | -2.77274 | 5.695123 | -2.39654 |
| 59 | 6 | 0 | -3.04981 | 4.538534 | -0.59613 |
| 60 | 1 | 0 | -3.9708 | 5.042838 | -0.31643 |
| 61 | 6 | 0 | -2.5434 | 3.521678 | 0.211718 |
| 62 | 1 | 0 | -3.07743 | 3.221919 | 1.108506 |
| 63 | 6 | 0 | -0.23583 | 2.231695 | 2.489847 |
| 64 | 6 | 0 | -0.17544 | 3.612842 | 2.704269 |
| 65 | 1 | 0 | -0.51716 | 4.295353 | 1.933966 |
| 66 | 6 | 0 | 0.34638 | 4.116397 | 3.897892 |
| 67 | 1 | 0 | 0.39906 | 5.190952 | 4.047934 |
| 68 | 6 | 0 | 0.805647 | 3.249241 | 4.888518 |
| 69 | 1 | 0 | 1.21365 | 3.644896 | 5.814133 |
| 70 | 6 | 0 | 0.749797 | 1.86718 | 4.680265 |
| 71 | 1 | 0 | 1.114534 | 1.184289 | 5.443008 |
| 72 | 6 | 0 | 0.246375 | 1.362411 | 3.48479 |
| 73 | 1 | 0 | 0.247227 | 0.290704 | 3.308667 |
| 74 | 6 | 0 | 4.69272 | -0.2573 | -0.62654 |
| 75 | 6 | 0 | 3.928366 | -1.20078 | 0.014105 |
| 76 | 6 | 0 | 3.374383 | -1.0267 | 1.29865 |
| 77 | 1 | 0 | 4.849747 | 0.705916 | -0.14398 |
| 78 | 1 | 0 | 3.75934 | -2.14385 | -0.50226 |
| 79 | 1 | 0 | 3.557129 | -0.07633 | 1.792463 |
| 80 | 6 | 0 | 2.669471 | -2.02005 | 2.058878 |
| 81 | 6 | 0 | 2.239031 | -1.70519 | 3.372599 |
| 82 | 6 | 0 | 2.392387 | -3.32448 | 1.577117 |
| 83 | 6 | 0 | 1.584813 | -2.64194 | 4.163635 |
| 84 | 1 | 0 | 2.441204 | -0.7097 | 3.758722 |
| 85 | 6 | 0 | 1.744887 | -4.25724 | 2.375654 |
| 86 | 1 | 0 | 2.684216 | -3.60024 | 0.569683 |
| 87 | 6 | 0 | 1.338931 | -3.9302 | 3.674565 |
| 88 | 1 | 0 | 1.272519 | -2.37304 | 5.169585 |
| 89 | 1 | 0 | 1.540981 | -5.24851 | 1.980104 |
| 90 | 1 | 0 | 0.832003 | -4.66557 | 4.29206 |
| 91 | 6 | 0 | 5.35393 | -0.45864 | -1.95694 |
| 92 | 1 | 0 | 5.072682 | -1.45011 | -2.33547 |
| 93 | 6 | 0 | 4.89453 | 0.601478 | -2.97456 |
| 94 | 1 | 0 | 3.811926 | 0.558928 | -3.12972 |
| 95 | 1 | 0 | 5.131604 | 1.609234 | -2.61668 |
| 96 | 1 | 0 | 5.392081 | 0.453405 | -3.94099 |
| 97 | 6 | 0 | 6.887267 | -0.41917 | -1.80134 |
| 98 | 1 | 0 | 7.210492 | 0.552542 | -1.4087 |
| 99 | 1 | 0 | 7.233735 | -1.19463 | -1.11006 |
| 100 | 1 | 0 | 7.380771 | -0.57253 | -2.76879 |
| 101 | 6 | 0 | 2.417588 | 2.107714 | 0.130405 |
| 102 | 6 | 0 | 2.926774 | 2.590412 | 1.348997 |
| 103 | 6 | 0 | 2.87276 | 2.800169 | -1.00764 |
| 104 | 6 | 0 | 3.834549 | 3.651311 | 1.37294 |
| 105 | 1 | 0 | 2.623479 | 2.147878 | 2.294765 |
| 106 | 6 | 0 | 3.781009 | 3.853329 | -0.88806 |
| 107 | 1 | 0 | 2.542336 | 2.513896 | -2.00436 |
| 108 | 7 | 0 | 4.274964 | 4.289029 | 0.279547 |
| 109 | 1 | 0 | 4.227858 | 4.011462 | 2.324633 |
| 110 | 1 | 0 | 4.135714 | 4.373858 | -1.77925 |

**TS-R**

| Center Number | Atomic  Number | Atomic  Type | Coordinates (Angstroms) | | |
| --- | --- | --- | --- | --- | --- |
|  |  |  | X | Y | Z |
| 1 | 46 | 0 | 0.833151 | 0.529858 | -0.16664 |
| 2 | 15 | 0 | -1.14108 | 1.654231 | 0.296399 |
| 3 | 15 | 0 | -0.17768 | -1.53216 | -0.60718 |
| 4 | 8 | 0 | -3.97729 | -2.12221 | 3.924506 |
| 5 | 8 | 0 | -4.07206 | -2.53034 | 1.646604 |
| 6 | 8 | 0 | -5.6343 | -0.67265 | -2.81973 |
| 7 | 8 | 0 | -5.28594 | -0.23777 | -0.56858 |
| 8 | 6 | 0 | -2.77883 | -0.55197 | 0.938134 |
| 9 | 6 | 0 | -2.12907 | 0.614646 | 1.446932 |
| 10 | 6 | 0 | -2.10157 | 0.860328 | 2.82312 |
| 11 | 1 | 0 | -1.60554 | 1.74919 | 3.193067 |
| 12 | 6 | 0 | -2.69136 | -0.01078 | 3.756065 |
| 13 | 1 | 0 | -2.65953 | 0.19047 | 4.820742 |
| 14 | 6 | 0 | -4.32629 | -3.09967 | 2.935622 |
| 15 | 1 | 0 | -5.38957 | -3.33915 | 3.021897 |
| 16 | 1 | 0 | -3.69808 | -3.98809 | 3.067889 |
| 17 | 6 | 0 | -3.36655 | -1.37617 | 1.879378 |
| 18 | 6 | 0 | -3.31292 | -1.1313 | 3.249909 |
| 19 | 6 | 0 | -2.91031 | -0.89333 | -0.50684 |
| 20 | 6 | 0 | -1.86055 | -1.41821 | -1.31989 |
| 21 | 6 | 0 | -2.09122 | -1.70008 | -2.67162 |
| 22 | 1 | 0 | -1.28469 | -2.10193 | -3.27299 |
| 23 | 6 | 0 | -3.33822 | -1.48226 | -3.28291 |
| 24 | 1 | 0 | -3.50258 | -1.69696 | -4.33276 |
| 25 | 6 | 0 | -6.25879 | -0.20454 | -1.61754 |
| 26 | 1 | 0 | -7.09487 | -0.86457 | -1.35953 |
| 27 | 1 | 0 | -6.59661 | 0.82615 | -1.76503 |
| 28 | 6 | 0 | -4.13137 | -0.72149 | -1.13028 |
| 29 | 6 | 0 | -4.34285 | -0.98496 | -2.48136 |
| 30 | 6 | 0 | -0.81786 | 3.208549 | 1.213413 |
| 31 | 6 | 0 | 0.225097 | 3.185045 | 2.154083 |
| 32 | 1 | 0 | 0.782015 | 2.265291 | 2.309466 |
| 33 | 6 | 0 | 0.566284 | 4.3347 | 2.861639 |
| 34 | 1 | 0 | 1.375849 | 4.300512 | 3.584953 |
| 35 | 6 | 0 | -0.10969 | 5.532351 | 2.61771 |
| 36 | 1 | 0 | 0.165536 | 6.433756 | 3.15792 |
| 37 | 6 | 0 | -1.13121 | 5.569389 | 1.667997 |
| 38 | 1 | 0 | -1.65394 | 6.500359 | 1.466181 |
| 39 | 6 | 0 | -1.48829 | 4.4135 | 0.971284 |
| 40 | 1 | 0 | -2.27872 | 4.457274 | 0.230682 |
| 41 | 6 | 0 | -2.3379 | 2.097815 | -1.01495 |
| 42 | 6 | 0 | -3.64244 | 2.520244 | -0.7224 |
| 43 | 1 | 0 | -3.96584 | 2.594113 | 0.311416 |
| 44 | 6 | 0 | -4.53141 | 2.815115 | -1.75508 |
| 45 | 1 | 0 | -5.53739 | 3.153088 | -1.521 |
| 46 | 6 | 0 | -4.13391 | 2.665318 | -3.08637 |
| 47 | 1 | 0 | -4.8333 | 2.878242 | -3.88966 |
| 48 | 6 | 0 | -2.84008 | 2.232772 | -3.38304 |
| 49 | 1 | 0 | -2.53328 | 2.100705 | -4.41644 |
| 50 | 6 | 0 | -1.9434 | 1.956751 | -2.35125 |
| 51 | 1 | 0 | -0.94174 | 1.598266 | -2.5686 |
| 52 | 6 | 0 | -0.37205 | -2.65961 | 0.815151 |
| 53 | 6 | 0 | 0.376704 | -2.40583 | 1.972446 |
| 54 | 1 | 0 | 1.040426 | -1.54918 | 2.006484 |
| 55 | 6 | 0 | 0.267466 | -3.25325 | 3.074635 |
| 56 | 1 | 0 | 0.860821 | -3.05328 | 3.961407 |
| 57 | 6 | 0 | -0.58691 | -4.35622 | 3.027825 |
| 58 | 1 | 0 | -0.6668 | -5.01901 | 3.885114 |
| 59 | 6 | 0 | -1.33728 | -4.61165 | 1.875786 |
| 60 | 1 | 0 | -1.99813 | -5.47366 | 1.834091 |
| 61 | 6 | 0 | -1.23791 | -3.76222 | 0.774442 |
| 62 | 1 | 0 | -1.83936 | -3.94508 | -0.11078 |
| 63 | 6 | 0 | 0.781624 | -2.46377 | -1.85578 |
| 64 | 6 | 0 | 1.054907 | -3.8328 | -1.75675 |
| 65 | 1 | 0 | 0.646044 | -4.40801 | -0.93342 |
| 66 | 6 | 0 | 1.875863 | -4.45412 | -2.69934 |
| 67 | 1 | 0 | 2.091603 | -5.51486 | -2.60701 |
| 68 | 6 | 0 | 2.424744 | -3.71828 | -3.75072 |
| 69 | 1 | 0 | 3.06885 | -4.20413 | -4.47796 |
| 70 | 6 | 0 | 2.153043 | -2.35164 | -3.85848 |
| 71 | 1 | 0 | 2.586344 | -1.77026 | -4.66746 |
| 72 | 6 | 0 | 1.344225 | -1.72743 | -2.91163 |
| 73 | 1 | 0 | 1.166574 | -0.65632 | -2.96412 |
| 74 | 6 | 0 | 6.140558 | -0.09596 | -0.72223 |
| 75 | 6 | 0 | 5.30224 | 0.898339 | -0.37771 |
| 76 | 6 | 0 | 3.936888 | 1.024556 | -0.86812 |
| 77 | 1 | 0 | 5.821452 | -0.81955 | -1.47583 |
| 78 | 1 | 0 | 5.622118 | 1.58644 | 0.403543 |
| 79 | 1 | 0 | 3.739864 | 0.449555 | -1.77257 |
| 80 | 6 | 0 | 3.220021 | 2.304659 | -0.83089 |
| 81 | 6 | 0 | 2.098117 | 2.488498 | -1.6763 |
| 82 | 6 | 0 | 3.505498 | 3.329009 | 0.092186 |
| 83 | 6 | 0 | 1.331932 | 3.657223 | -1.62696 |
| 84 | 1 | 0 | 1.883782 | 1.733721 | -2.43001 |
| 85 | 6 | 0 | 2.73107 | 4.485174 | 0.147931 |
| 86 | 1 | 0 | 4.345144 | 3.220796 | 0.770646 |
| 87 | 6 | 0 | 1.640819 | 4.659803 | -0.71067 |
| 88 | 1 | 0 | 0.483787 | 3.771312 | -2.29672 |
| 89 | 1 | 0 | 2.974108 | 5.257272 | 0.872546 |
| 90 | 1 | 0 | 1.033335 | 5.556729 | -0.64964 |
| 91 | 6 | 0 | 7.484227 | -0.34934 | -0.09763 |
| 92 | 1 | 0 | 7.701669 | 0.479232 | 0.591485 |
| 93 | 6 | 0 | 7.451923 | -1.65418 | 0.72184 |
| 94 | 1 | 0 | 6.685812 | -1.6109 | 1.501788 |
| 95 | 1 | 0 | 7.217634 | -2.50876 | 0.074053 |
| 96 | 1 | 0 | 8.42529 | -1.84693 | 1.190895 |
| 97 | 6 | 0 | 8.595786 | -0.39824 | -1.15986 |
| 98 | 1 | 0 | 8.648373 | 0.541368 | -1.72087 |
| 99 | 1 | 0 | 9.574618 | -0.58149 | -0.69999 |
| 100 | 1 | 0 | 8.408413 | -1.20731 | -1.8778 |
| 101 | 6 | 0 | 3.775195 | -2.27553 | 1.182098 |
| 102 | 6 | 0 | 3.264589 | -1.50149 | 0.151641 |
| 103 | 6 | 0 | 2.868479 | -0.14403 | 0.368979 |
| 104 | 6 | 0 | 3.072724 | 0.290303 | 1.722791 |
| 105 | 6 | 0 | 3.586908 | -0.56556 | 2.682535 |
| 106 | 7 | 0 | 3.938341 | -1.85203 | 2.451975 |
| 107 | 1 | 0 | 4.058151 | -3.31157 | 0.992347 |
| 108 | 1 | 0 | 3.178613 | -1.94172 | -0.83703 |
| 109 | 1 | 0 | 2.855835 | 1.321523 | 1.997445 |
| 110 | 1 | 0 | 3.722503 | -0.2185 | 3.707924 |

**TS-R-L**

| Center Number | Atomic  Number | Atomic  Type | Coordinates (Angstroms) | | |
| --- | --- | --- | --- | --- | --- |
|  |  |  | X | Y | Z |
| 1 | 46 | 0 | -1.25431 | 0.120929 | 0.190286 |
| 2 | 15 | 0 | 0.366914 | 1.390913 | -0.91536 |
| 3 | 15 | 0 | 0.236111 | -1.47019 | 1.0773 |
| 4 | 8 | 0 | 3.033348 | -3.1497 | -3.69225 |
| 5 | 8 | 0 | 3.677588 | -2.67574 | -1.51946 |
| 6 | 8 | 0 | 5.683837 | 0.859083 | 1.621081 |
| 7 | 8 | 0 | 4.879419 | 0.40407 | -0.5059 |
| 8 | 6 | 0 | 2.223396 | -0.70958 | -1.20186 |
| 9 | 6 | 0 | 1.276566 | 0.118522 | -1.88294 |
| 10 | 6 | 0 | 0.91749 | -0.16201 | -3.20401 |
| 11 | 1 | 0 | 0.195753 | 0.474276 | -3.70274 |
| 12 | 6 | 0 | 1.450155 | -1.25475 | -3.91252 |
| 13 | 1 | 0 | 1.159825 | -1.46498 | -4.93563 |
| 14 | 6 | 0 | 3.732987 | -3.66717 | -2.55162 |
| 15 | 1 | 0 | 4.776379 | -3.85019 | -2.82079 |
| 16 | 1 | 0 | 3.23565 | -4.57904 | -2.20135 |
| 17 | 6 | 0 | 2.742267 | -1.76172 | -1.93183 |
| 18 | 6 | 0 | 2.358127 | -2.0452 | -3.24197 |
| 19 | 6 | 0 | 2.690694 | -0.48847 | 0.196815 |
| 20 | 6 | 0 | 1.920651 | -0.80815 | 1.355364 |
| 21 | 6 | 0 | 2.424729 | -0.55154 | 2.633606 |
| 22 | 1 | 0 | 1.826414 | -0.80577 | 3.500509 |
| 23 | 6 | 0 | 3.692981 | 0.022801 | 2.835351 |
| 24 | 1 | 0 | 4.073644 | 0.227804 | 3.829546 |
| 25 | 6 | 0 | 5.989713 | 0.937036 | 0.222843 |
| 26 | 1 | 0 | 6.883474 | 0.339373 | 0.011161 |
| 27 | 1 | 0 | 6.135285 | 1.985485 | -0.05567 |
| 28 | 6 | 0 | 3.947607 | 0.034094 | 0.429857 |
| 29 | 6 | 0 | 4.43311 | 0.304327 | 1.707529 |
| 30 | 6 | 0 | -0.43221 | 2.484404 | -2.15608 |
| 31 | 6 | 0 | -1.66193 | 2.055625 | -2.68393 |
| 32 | 1 | 0 | -2.0538 | 1.087846 | -2.38789 |
| 33 | 6 | 0 | -2.39546 | 2.873257 | -3.54096 |
| 34 | 1 | 0 | -3.34588 | 2.52428 | -3.93459 |
| 35 | 6 | 0 | -1.91777 | 4.144918 | -3.8683 |
| 36 | 1 | 0 | -2.49458 | 4.792914 | -4.52194 |
| 37 | 6 | 0 | -0.70095 | 4.582786 | -3.34276 |
| 38 | 1 | 0 | -0.3278 | 5.573328 | -3.58822 |
| 39 | 6 | 0 | 0.040635 | 3.758711 | -2.49439 |
| 40 | 1 | 0 | 0.971744 | 4.120911 | -2.07472 |
| 41 | 6 | 0 | 1.71537 | 2.401925 | -0.20276 |
| 42 | 6 | 0 | 2.824596 | 2.796389 | -0.96616 |
| 43 | 1 | 0 | 2.901639 | 2.490875 | -2.00528 |
| 44 | 6 | 0 | 3.837921 | 3.556507 | -0.38516 |
| 45 | 1 | 0 | 4.688254 | 3.870361 | -0.98496 |
| 46 | 6 | 0 | 3.76506 | 3.904549 | 0.967141 |
| 47 | 1 | 0 | 4.562742 | 4.484841 | 1.42238 |
| 48 | 6 | 0 | 2.667752 | 3.504656 | 1.73036 |
| 49 | 1 | 0 | 2.602907 | 3.773767 | 2.780145 |
| 50 | 6 | 0 | 1.643181 | 2.761121 | 1.146099 |
| 51 | 1 | 0 | 0.785746 | 2.455938 | 1.733391 |
| 52 | 6 | 0 | 0.572145 | -3.09135 | 0.302718 |
| 53 | 6 | 0 | -0.25878 | -3.51653 | -0.73905 |
| 54 | 1 | 0 | -1.05667 | -2.87066 | -1.08187 |
| 55 | 6 | 0 | -0.05788 | -4.76161 | -1.33465 |
| 56 | 1 | 0 | -0.72539 | -5.08665 | -2.12641 |
| 57 | 6 | 0 | 0.981346 | -5.58406 | -0.89639 |
| 58 | 1 | 0 | 1.133882 | -6.55842 | -1.35307 |
| 59 | 6 | 0 | 1.826861 | -5.15609 | 0.13341 |
| 60 | 1 | 0 | 2.640271 | -5.79263 | 0.471611 |
| 61 | 6 | 0 | 1.627662 | -3.91155 | 0.728984 |
| 62 | 1 | 0 | 2.289484 | -3.57286 | 1.520469 |
| 63 | 6 | 0 | -0.41301 | -1.85574 | 2.743189 |
| 64 | 6 | 0 | -0.64506 | -3.16218 | 3.189654 |
| 65 | 1 | 0 | -0.35236 | -4.00389 | 2.571875 |
| 66 | 6 | 0 | -1.26784 | -3.3844 | 4.419684 |
| 67 | 1 | 0 | -1.44925 | -4.40266 | 4.752288 |
| 68 | 6 | 0 | -1.65916 | -2.30853 | 5.21722 |
| 69 | 1 | 0 | -2.14583 | -2.48556 | 6.17205 |
| 70 | 6 | 0 | -1.42916 | -1.00079 | 4.779181 |
| 71 | 1 | 0 | -1.736 | -0.15766 | 5.392215 |
| 72 | 6 | 0 | -0.8213 | -0.77537 | 3.546698 |
| 73 | 1 | 0 | -0.6715 | 0.2406 | 3.190799 |
| 74 | 6 | 0 | -4.38187 | 0.343113 | 0.346936 |
| 75 | 6 | 0 | -3.62853 | 1.56956 | 0.343119 |
| 76 | 6 | 0 | -2.76882 | 1.894747 | 1.374177 |
| 77 | 1 | 0 | -3.67013 | 2.21093 | -0.53362 |
| 78 | 1 | 0 | -2.81008 | 1.259206 | 2.259666 |
| 79 | 6 | 0 | -1.97938 | 3.10896 | 1.534001 |
| 80 | 6 | 0 | -1.41176 | 3.393301 | 2.794207 |
| 81 | 6 | 0 | -1.75068 | 4.031119 | 0.491312 |
| 82 | 6 | 0 | -0.65006 | 4.539711 | 3.004335 |
| 83 | 1 | 0 | -1.58543 | 2.700103 | 3.614603 |
| 84 | 6 | 0 | -0.9774 | 5.167303 | 0.699955 |
| 85 | 1 | 0 | -2.16629 | 3.843517 | -0.49178 |
| 86 | 6 | 0 | -0.42032 | 5.432562 | 1.954964 |
| 87 | 1 | 0 | -0.22984 | 4.73414 | 3.987873 |
| 88 | 1 | 0 | -0.80156 | 5.847429 | -0.12892 |
| 89 | 1 | 0 | 0.185894 | 6.319959 | 2.110442 |
| 90 | 6 | 0 | -5.69135 | 0.264192 | -0.43867 |
| 91 | 1 | 0 | -6.37293 | 0.966027 | 0.073346 |
| 92 | 6 | 0 | -5.60052 | 0.731297 | -1.89864 |
| 93 | 1 | 0 | -5.16265 | 1.731556 | -1.98837 |
| 94 | 1 | 0 | -4.99997 | 0.038682 | -2.49419 |
| 95 | 1 | 0 | -6.60202 | 0.769961 | -2.34237 |
| 96 | 6 | 0 | -6.34548 | -1.12187 | -0.34929 |
| 97 | 1 | 0 | -5.77613 | -1.87167 | -0.90388 |
| 98 | 1 | 0 | -6.41593 | -1.45762 | 0.691478 |
| 99 | 1 | 0 | -7.3601 | -1.0902 | -0.76366 |
| 100 | 6 | 0 | -3.10652 | -1.14621 | -0.18339 |
| 101 | 6 | 0 | -3.32179 | -2.26282 | 0.679297 |
| 102 | 6 | 0 | -3.15102 | -1.49268 | -1.56877 |
| 103 | 6 | 0 | -3.55527 | -3.53355 | 0.171714 |
| 104 | 1 | 0 | -3.29449 | -2.12639 | 1.758964 |
| 105 | 6 | 0 | -3.41121 | -2.79448 | -1.98045 |
| 106 | 1 | 0 | -2.97451 | -0.74033 | -2.33308 |
| 107 | 7 | 0 | -3.61654 | -3.82946 | -1.14266 |
| 108 | 1 | 0 | -3.69138 | -4.37534 | 0.851234 |
| 109 | 1 | 0 | -3.44027 | -3.03388 | -3.04452 |
| 110 | 1 | 0 | -4.50429 | -0.06138 | 1.352836 |

**TS-S**

| Center Number | Atomic  Number | Atomic  Type | Coordinates (Angstroms) | | |
| --- | --- | --- | --- | --- | --- |
|  |  |  | X | Y | Z |
| 1 | 46 | 0 | -0.82636 | 0.548625 | -0.04961 |
| 2 | 15 | 0 | 1.094974 | 1.645406 | -0.74106 |
| 3 | 15 | 0 | 0.275501 | -1.32553 | 0.832492 |
| 4 | 8 | 0 | 3.539549 | -2.77899 | -3.86895 |
| 5 | 8 | 0 | 3.864403 | -2.76013 | -1.57894 |
| 6 | 8 | 0 | 5.905601 | -0.00668 | 2.252927 |
| 7 | 8 | 0 | 5.321381 | -0.04771 | 0.009225 |
| 8 | 6 | 0 | 2.676065 | -0.64415 | -1.129 |
| 9 | 6 | 0 | 1.990601 | 0.426577 | -1.78223 |
| 10 | 6 | 0 | 1.831767 | 0.420396 | -3.17074 |
| 11 | 1 | 0 | 1.308456 | 1.240114 | -3.64847 |
| 12 | 6 | 0 | 2.31537 | -0.62567 | -3.97745 |
| 13 | 1 | 0 | 2.18472 | -0.62083 | -5.05359 |
| 14 | 6 | 0 | 3.912464 | -3.58908 | -2.74566 |
| 15 | 1 | 0 | 4.931565 | -3.95575 | -2.88671 |
| 16 | 1 | 0 | 3.193185 | -4.4097 | -2.63333 |
| 17 | 6 | 0 | 3.149015 | -1.64946 | -1.95011 |
| 18 | 6 | 0 | 2.960337 | -1.6584 | -3.33218 |
| 19 | 6 | 0 | 2.963821 | -0.6926 | 0.332813 |
| 20 | 6 | 0 | 2.010812 | -1.0242 | 1.34127 |
| 21 | 6 | 0 | 2.38293 | -1.01981 | 2.690113 |
| 22 | 1 | 0 | 1.647845 | -1.27862 | 3.442126 |
| 23 | 6 | 0 | 3.683281 | -0.68906 | 3.109937 |
| 24 | 1 | 0 | 3.956877 | -0.67733 | 4.158863 |
| 25 | 6 | 0 | 6.401415 | 0.177584 | 0.920699 |
| 26 | 1 | 0 | 7.200541 | -0.54701 | 0.724935 |
| 27 | 1 | 0 | 6.760771 | 1.205175 | 0.809033 |
| 28 | 6 | 0 | 4.240079 | -0.40276 | 0.775521 |
| 29 | 6 | 0 | 4.592982 | -0.37931 | 2.122615 |
| 30 | 6 | 0 | 0.639974 | 3.028659 | -1.85836 |
| 31 | 6 | 0 | -0.48659 | 2.842729 | -2.68065 |
| 32 | 1 | 0 | -1.01424 | 1.891914 | -2.65005 |
| 33 | 6 | 0 | -0.93962 | 3.868554 | -3.50661 |
| 34 | 1 | 0 | -1.80786 | 3.707624 | -4.13926 |
| 35 | 6 | 0 | -0.29079 | 5.106583 | -3.50322 |
| 36 | 1 | 0 | -0.65212 | 5.912744 | -4.13511 |
| 37 | 6 | 0 | 0.813838 | 5.306392 | -2.67561 |
| 38 | 1 | 0 | 1.31612 | 6.269664 | -2.66121 |
| 39 | 6 | 0 | 1.281155 | 4.27371 | -1.85937 |
| 40 | 1 | 0 | 2.138326 | 4.443774 | -1.21777 |
| 41 | 6 | 0 | 2.392511 | 2.327224 | 0.354707 |
| 42 | 6 | 0 | 3.65655 | 2.689746 | -0.13327 |
| 43 | 1 | 0 | 3.882401 | 2.567281 | -1.18831 |
| 44 | 6 | 0 | 4.629836 | 3.176842 | 0.737562 |
| 45 | 1 | 0 | 5.603926 | 3.465627 | 0.351772 |
| 46 | 6 | 0 | 4.356542 | 3.283339 | 2.104051 |
| 47 | 1 | 0 | 5.121618 | 3.646324 | 2.784268 |
| 48 | 6 | 0 | 3.102809 | 2.915053 | 2.594821 |
| 49 | 1 | 0 | 2.891767 | 2.985153 | 3.657934 |
| 50 | 6 | 0 | 2.120685 | 2.444136 | 1.723401 |
| 51 | 1 | 0 | 1.147078 | 2.146944 | 2.098796 |
| 52 | 6 | 0 | 0.391931 | -2.84237 | -0.17842 |
| 53 | 6 | 0 | -0.50607 | -3.00739 | -1.24056 |
| 54 | 1 | 0 | -1.22866 | -2.22994 | -1.45874 |
| 55 | 6 | 0 | -0.47055 | -4.16838 | -2.01233 |
| 56 | 1 | 0 | -1.18064 | -4.29083 | -2.82443 |
| 57 | 6 | 0 | 0.463384 | -5.1674 | -1.73156 |
| 58 | 1 | 0 | 0.486743 | -6.07442 | -2.32964 |
| 59 | 6 | 0 | 1.366572 | -5.00333 | -0.67643 |
| 60 | 1 | 0 | 2.093944 | -5.77983 | -0.45424 |
| 61 | 6 | 0 | 1.335682 | -3.84309 | 0.096488 |
| 62 | 1 | 0 | 2.045624 | -3.70913 | 0.90656 |
| 63 | 6 | 0 | -0.55559 | -1.83071 | 2.379831 |
| 64 | 6 | 0 | -0.87675 | -3.15917 | 2.681046 |
| 65 | 1 | 0 | -0.5833 | -3.95407 | 2.0042 |
| 66 | 6 | 0 | -1.59503 | -3.45955 | 3.839583 |
| 67 | 1 | 0 | -1.84987 | -4.49225 | 4.060672 |
| 68 | 6 | 0 | -1.99088 | -2.44013 | 4.707278 |
| 69 | 1 | 0 | -2.55459 | -2.67769 | 5.605019 |
| 70 | 6 | 0 | -1.67183 | -1.11229 | 4.410729 |
| 71 | 1 | 0 | -1.99205 | -0.31049 | 5.069651 |
| 72 | 6 | 0 | -0.96705 | -0.80686 | 3.2491 |
| 73 | 1 | 0 | -0.76938 | 0.230128 | 2.993196 |
| 74 | 6 | 0 | -6.17839 | 0.649932 | -0.71158 |
| 75 | 6 | 0 | -5.28052 | 0.866961 | 0.265496 |
| 76 | 6 | 0 | -3.93441 | 1.376642 | 0.036411 |
| 77 | 1 | 0 | -5.92778 | 0.944953 | -1.73301 |
| 78 | 1 | 0 | -5.52932 | 0.538124 | 1.273795 |
| 79 | 1 | 0 | -3.81038 | 1.84747 | -0.93864 |
| 80 | 6 | 0 | -3.16552 | 2.053814 | 1.08625 |
| 81 | 6 | 0 | -2.10638 | 2.911726 | 0.697128 |
| 82 | 6 | 0 | -3.33078 | 1.803413 | 2.461076 |
| 83 | 6 | 0 | -1.26313 | 3.496838 | 1.645742 |
| 84 | 1 | 0 | -1.99841 | 3.175079 | -0.35139 |
| 85 | 6 | 0 | -2.48446 | 2.38543 | 3.40215 |
| 86 | 1 | 0 | -4.12241 | 1.141299 | 2.795809 |
| 87 | 6 | 0 | -1.43988 | 3.22959 | 3.00352 |
| 88 | 1 | 0 | -0.46711 | 4.158387 | 1.314438 |
| 89 | 1 | 0 | -2.64221 | 2.183652 | 4.45889 |
| 90 | 1 | 0 | -0.78782 | 3.685099 | 3.743675 |
| 91 | 6 | 0 | -7.50837 | -0.02769 | -0.53422 |
| 92 | 1 | 0 | -7.61579 | -0.29446 | 0.526883 |
| 93 | 6 | 0 | -8.668 | 0.909702 | -0.91499 |
| 94 | 1 | 0 | -8.66184 | 1.816103 | -0.29968 |
| 95 | 1 | 0 | -8.58718 | 1.217129 | -1.96565 |
| 96 | 1 | 0 | -9.63681 | 0.411153 | -0.78743 |
| 97 | 6 | 0 | -7.55992 | -1.32483 | -1.36364 |
| 98 | 1 | 0 | -7.46292 | -1.09864 | -2.43354 |
| 99 | 1 | 0 | -6.74346 | -2.00164 | -1.09467 |
| 100 | 1 | 0 | -8.51558 | -1.84425 | -1.21843 |
| 101 | 6 | 0 | -2.85968 | -0.26522 | -0.37534 |
| 102 | 6 | 0 | -3.23276 | -1.30461 | 0.541269 |
| 103 | 6 | 0 | -3.15341 | -0.59792 | -1.7419 |
| 104 | 6 | 0 | -3.81174 | -2.48226 | 0.099943 |
| 105 | 1 | 0 | -3.07866 | -1.16371 | 1.60691 |
| 106 | 6 | 0 | -3.76934 | -1.79197 | -2.07694 |
| 107 | 1 | 0 | -2.92353 | 0.112505 | -2.53556 |
| 108 | 7 | 0 | -4.09842 | -2.75877 | -1.19098 |
| 109 | 1 | 0 | -4.06014 | -3.26653 | 0.815587 |
| 110 | 1 | 0 | -4.00047 | -2.01325 | -3.11997 |

**E_branched_product**

| Center Number | Atomic  Number | Atomic  Type | Coordinates (Angstroms) | | |
| --- | --- | --- | --- | --- | --- |
|  |  |  | X | Y | Z |
| 1 | 6 | 0 | -2.41916 | 0.014484 | -0.35402 |
| 2 | 6 | 0 | -1.21654 | 0.008885 | 0.222281 |
| 3 | 6 | 0 | 0.097166 | 0.020454 | -0.52572 |
| 4 | 1 | 0 | -2.48438 | 0.048416 | -1.44456 |
| 5 | 1 | 0 | -1.14024 | -0.02907 | 1.309568 |
| 6 | 1 | 0 | -0.13416 | 0.002892 | -1.59992 |
| 7 | 6 | 0 | 0.873031 | -1.25072 | -0.20274 |
| 8 | 6 | 0 | 0.945007 | -2.29324 | -1.13075 |
| 9 | 6 | 0 | 1.48442 | -1.41747 | 1.047065 |
| 10 | 6 | 0 | 1.615002 | -3.47877 | -0.82288 |
| 11 | 1 | 0 | 0.470486 | -2.17575 | -2.10202 |
| 12 | 6 | 0 | 2.15187 | -2.60032 | 1.35931 |
| 13 | 1 | 0 | 1.447796 | -0.60886 | 1.772182 |
| 14 | 6 | 0 | 2.219635 | -3.63607 | 0.42425 |
| 15 | 1 | 0 | 1.664717 | -4.27705 | -1.55824 |
| 16 | 1 | 0 | 2.622674 | -2.71301 | 2.332002 |
| 17 | 1 | 0 | 2.742617 | -4.55691 | 0.666138 |
| 18 | 6 | 0 | -3.73588 | -0.03048 | 0.375157 |
| 19 | 1 | 0 | -3.52868 | -0.05858 | 1.453788 |
| 20 | 6 | 0 | -4.56564 | 1.229592 | 0.074576 |
| 21 | 1 | 0 | -4.03061 | 2.136533 | 0.376315 |
| 22 | 1 | 0 | -4.77993 | 1.307487 | -0.99881 |
| 23 | 1 | 0 | -5.52438 | 1.202669 | 0.605489 |
| 24 | 6 | 0 | -4.51657 | -1.3023 | 0.00044 |
| 25 | 1 | 0 | -4.73336 | -1.32249 | -1.07514 |
| 26 | 1 | 0 | -3.94326 | -2.20269 | 0.244733 |
| 27 | 1 | 0 | -5.4729 | -1.34554 | 0.534739 |
| 28 | 6 | 0 | 0.911744 | 1.289274 | -0.27266 |
| 29 | 6 | 0 | 2.285784 | 1.333262 | -0.53684 |
| 30 | 6 | 0 | 0.315347 | 2.468817 | 0.179958 |
| 31 | 6 | 0 | 2.980031 | 2.524722 | -0.33861 |
| 32 | 1 | 0 | 2.809787 | 0.448699 | -0.88396 |
| 33 | 6 | 0 | 1.101409 | 3.609441 | 0.35038 |
| 34 | 1 | 0 | -0.74796 | 2.500001 | 0.393233 |
| 35 | 7 | 0 | 2.414889 | 3.657183 | 0.101851 |
| 36 | 1 | 0 | 4.048841 | 2.572198 | -0.5411 |
| 37 | 1 | 0 | 0.648697 | 4.533349 | 0.707389 |
